# Genetic Recovery of Two Wild Seatrout Populations Following Long‐Term Stocking With Non‐Native Conspecifics

**DOI:** 10.1111/mec.70036

**Published:** 2025-07-12

**Authors:** Dorte Bekkevold, Kevin A. Glover, Belén Jimenez‐Mena, Francois Besnier, Einar E. Nielsen

**Affiliations:** ^1^ National Institute of Aquatic Resources, Technical University of Denmark Silkeborg Denmark; ^2^ Institute of Marine Research Bergen Norway

**Keywords:** admixture, brown trout, genetic effects of stocking, introgression, maturation genes, *six6*

## Abstract

Predicting the long‐term impact of releases and introgression from non‐native strains into wild populations remains an important conservation issue, particularly in fishes where stocking and aquaculture escapes have led to widespread genetic admixture between wild and cultured conspecifics. Here, we investigated the genetic response of two wild sea trout, *Salmo trutta* L., populations following long‐term stocking programmes with non‐native conspecifics. This included temporal sampling spanning 23 years and genome‐wide SNP data from two neighbouring Danish rivers that from the 1970's to 1990's were heavily stocked with partially domesticated, non‐local hatchery strains. To estimate wild‐hatchery admixture we analysed 3656 SNPs in 195 wild‐caught fish, 74 hatchery strain fish, and expanded collections temporally by analysing a subset 288 SNPs in 489 additional fish. Admixture estimates decreased from 46% to 62% input from the stocked strains to 25% seven generations after the last stocking. Introgression varied across the genome, indicative of selection for and against specific hatchery gene variants under wild conditions. For the first time in trout, strong temporal allele frequency changes were observed in a gene region harbouring the maturation gene *six6* likely associated with divergent selection on age‐at‐maturity under hatchery versus wild conditions. The two populations showed low overlap between SNPs identified as under negative (or purifying) selection. Results point to selection against hatchery fish and partial recovery of both populations but also emphasise the role of local dynamics in shaping genetic responses to anthropogenic pressure and support the notion that introgression is likely to incur long‐lasting changes to the genetic make‐up of wild populations.

## 1 Introduction

Evaluating to which extent populations' genetic status and evolutionary trajectories are altered as a result of releases of non‐native strains (e.g., to support threatened wild populations) has been a central objective in conservation and evolutionary genetics (Rhymer and Simberloff 1996; Allendorf et al. 2001). Interbreeding between local populations and non‐native strains and their backcrosses results in genetic admixture that may lead to introgressive hybridisation, potentially causing break‐up of co‐adapted gene complexes and loss of local adaptation in recipient populations (Edmands 2007). This concern has led to extensive study of genetic and demographic impacts of introgression (Todesco et al. 2016). There has been less focus on genetic recovery of populations that have previously undergone introgression, sometimes referred to as de‐introgression, via natural selection and genetic drift. Salmonid fishes (Salmonidae) have been extensively used as models to study introgressive hybridisation, from both an interspecific and, especially, an intra‐specific perspective. Reasons for this include their generally well‐described population structure and remarkable ecological variance reflecting a general capacity for adapting to local environments (Fraser et al. 2011). Additionally, the widespread tradition of deliberate releases (and in recent decades large‐scale unintentional escapes of farmed fish) of genetically divergent conspecifics has allowed testing of a suite of hypotheses relating to outcomes of unidirectional gene‐flow from non‐native individuals into wild gene pools (see Glover et al. 2017 for a review in Atlantic salmon). Releases typically represent a range of scenarios associated with increasing expected severity to fitness effects in wild populations (Baskett and Waples 2013); from supplementation of hatchery produced fish of indigenous brood‐stock, over stocking wild fish with ‘non‐indigenous’ genetic makeup, to stocking partially or fully domesticated hatchery strains exhibiting strong genetic divergence from the wild population.

Stocking and escapes of non‐native fish are repeatedly shown to cause changes to native gene pools, as assessed with molecular markers (e.g., Marie et al. 2010; Hansen et al. 2009; Glover et al. 2012) and by comparing phenotype data and reaction norms in genetically wild versus introgressed individuals (Besnier et al. 2022; Bolstad et al. 2017, 2021; Bekkevold, Besnier, et al. 2024). Salmonid studies have reported additive effects of introgression on a suite of fitness traits under wild (McGinnity et al. 2003; Reed et al. 2015; Skaala et al. 2012, 2019) and experimental (Glover et al. 2009; Harvey et al. 2016; Solberg et al. 2013; Bekkevold, Besnier, et al. 2024) conditions. However, whereas most studies report effects of on‐going gene flow (e.g., Glover et al. 2020) knowledge is scarce about genetic and functional responses once recipient populations are released from directed gene flow from non‐native strains (Harbicht et al. 2014; Wacker et al. 2021).

Population recovery from introgression by mal‐adapted genotypes is expected to vary, depending on drivers including the ratio of immigrant to wild individuals (to which extent immigrants are able to swamp local gene pools; Ryman and Laikre 1991; Baskett et al. 2013; Tufto 2017; Castellani et al. 2018; Diserud et al. 2022), specific fitness consequences of introgression (to which degree non‐native gene pools are mal‐adaptive; Baskett and Waples 2013), and to which extent the recipient population is large enough to prevent genetic drift from overriding natural selection against mal‐adapted traits (Hwang et al. 2016). Generally, it is expected that introgression can occur more unhindered when donor and recipient gene pools are weakly diverged (Castellani et al. 2015). Introgression is unlikely to be uniform across the genome as selective responses are expected, either against gene complexes that are mal‐adapted in the wild or through positive selection on introduced advantageous gene variants (Rieseberg et al. 1999) meaning introgression signals may vary accordingly.

In this study, we investigated the temporal development of genomic signatures of introgression following the cessation of releases of non‐native strains in two natural Sea trout *Salmo trutta* (L.) populations. During the 1970–1990's all major rivers draining into the North Sea on the west coast of Denmark were annually stocked with one or two partially domesticated, closely related, hatchery strains (Hansen et al. 2009). Wild populations and stocked strains (hereafter HAT) belong to the Atlantic lineage (Bernatchez 2001) but HAT came from mixed‐origin broodstock, chiefly originating in the river Kolding in the Western Baltic Sea and separated by more than 700 km least waterway distance from the North Sea (Hansen et al. 1997, 2001, Figure 1). Between 1991 and 2000, depending on the river, stocking with HAT was discontinued and all stocking has since been based on supplementary breeding of annually wild‐caught broodstock. We monitored introgression in two strongly impacted rivers, Varde and Skjern, which flow into the North Sea ca. 40 km apart. Genetic differentiation is moderate between HAT and both wild populations (*F*_ST_s estimated at 0.026 for both with 3.7 K genome‐spanning SNPs; Bekkevold et al. 2020), and a microsatellite marker study estimated that, respectively, 45% (Varde) and 64% (Skjern) of gene pools across spawning‐run fish originated from HAT approximately one generation (3.5 years) after HAT stocking ended (Hansen et al. 2009). We take advantage of long‐erm temporal sampling, low‐density genome‐wide SNP data in a total of 758 fish, and knowledge about the stocking history of trout in the two rivers, to address the following three questions: (1) does hatchery introgression decrease over time following cessation of hatchery stocking; (2) is introgression uniform across the genome; and (3) do weakly diverged populations within an eco‐zone vary with respect to which genomic regions show introgression?

**FIGURE 1:**
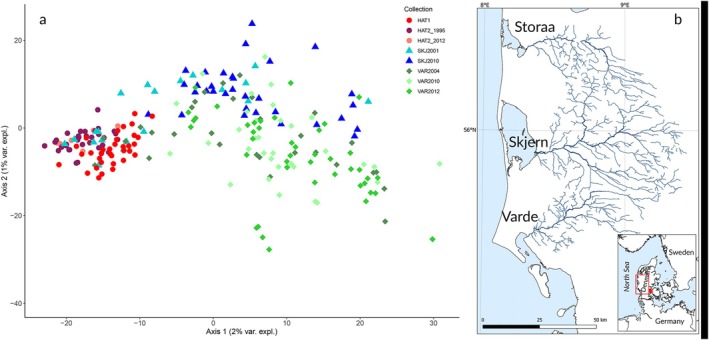
PCA plot of *S. trutta* samples typed for 3656 SNPs, showing the first two axes and their respective variance explained (a). Locations of study rivers all of which flow into the North Sea via lagoons (b). The location in the Western Baltic Sea of the river of origin for the stocked hatchery strain is indicated by an asterisk.

## 2 Materials and Methods

### 2.1 Temporal Samples

In the river Varde, temporal replicate samples of wild spawning run trout were collected over 20 years: 2004–2024. In the river Skjern, samples were collected over 23 years: 2001–2024 (Table 1). Fish were collected by electrofishing downstream spawning areas immediately prior to spawning time (December). Samples consisted of adipose fin clips removed from adult fish under anaesthesia. Procedures were conducted in accordance with the Danish Experimental Animal Inspectorate permit for DTU (Licence no. 2017‐15‐0201‐01164). For both rivers, collections of wild‐caught spawning run fish in 2001 and 2004 potentially represented a broad mix of genetic backgrounds, ranging from pure wild non‐admixed origin, over wild/HAT admixed genotypes resulting from several generations of interbreeding, to pure HAT, representing stocked hatchery fish. In the period 1977–1996 River Skjern was stocked with a total of ca. 1 million, mainly juvenile, HAT fish (Hansen et al. 2009). Broodstock used for stocking after 1996 represented wild‐caught spawning run fish. However, natural matings between wild and HAT fish resulted in River Skjern wild‐caught broodstock consisting of strongly admixed fish at least until 2000 (Hansen et al. 2006; Hansen et al. 2009). Thus, although direct stocking with HAT had ended by 2000, anthropogenic input from HAT continued through stocking with HAT admixed fish for at least a couple of generations. Genetic admixture analyses in fish from three tributaries, however, also indicated that non‐admixed wild contingents persisted in upper parts of the river (Hansen et al. 2006). To avoid inclusion of HAT admixed broodstock in stocking material, all broodstock candidates captured on spawning run in 2005–2011 were genetically screened using a method detailed in Hansen et al. (2006). Stocking was discontinued 2012–2019 and reinstated in 2020, following observations suggesting decreasing population size.

**TABLE 1:** *S. trutta* Collections (Adult Fish on Spawning Run) From HAT Stocked Populations Skjern and Varde, the Neighbouring River Storaa, and the Two Hatchery Strains (HAT 1–2) Used for Stocking.

River	Sample id	Collection year	*N*	3656 SNP *q* [range] (CI)	288 SNP *q* [range] (CI)	*F*_st_ with HAT (CI) estimated with 288/3656 SNPs	% individuals classified with low or no HAT admixture
Skjern	SKJ_2001_	2001	20	0.62 [0.01–0.99] (0.15)	0.73 [0.06–0.97] (0.32)	0.019 (0.010–0.027)/0.007 (0.004–0.015)	5
	SKJ_2007_ ^a^	2007	30	—	0.61 [0.12–0.94] (0.34)	0.027 (0.020–0.033)/na	17
	SKJ_2010_	2010	32	0.24 [0.01–0.63] (0.14)	0.42 [0.03–0.87] (0.35)	0.041 (0.034–0.052)/0.021 (0.016–0.026)	31
	SKJ_2019_ ^a^	2019	31	—	0.29 [0.06–0.53] (0.35)	0.056 (0.044–0.068)/na	68
	SKJ_2021_ ^a^	2021	97	—	0.26 [0.04–0.68] (0.36)	0.052 (0.039–0.067)/na	77
	SKJ_2022_ ^a^	2022	79	—	0.28 [0.04–0.79] (0.36)	0.052 (0.040–0.064)/na	71
	SKJ_2023_ ^a^	2023	91	—	0.26 [0.04–0.86] (0.36)	0.053 (0.040–0.065)/na	74
	SKJ_2024_ ^a^	2024	58	—	0.25 [0.05–0.65] (0.30)	0.054 (0.041–0.070)/na	79
Varde	VAR_2004_	2004	30	0.46 [0.02–0.93] (0.12)	0.47 [0.04–0.87] (0.31)	0.037 (0.026–0.048)/0.021 (0.012–0.034)	33
	VAR_2010_	2010	34	0.36 [0.00–0.70] (0.13)	0.39 [0.05–0.74] (0.34)	0.046 (0.036–0.057)/0.032 (0.025–0.041)	29
	VAR_2012_	2012	45	0.28 [0.00–0.66] (0.12)	0.30 [0.04–0.83] (0.30)	0.051 (0.042–0.059)/0.042 (0.032–0.053)	64
	VAR_2016_ ^a^	2016	48	—	0.21 [0.02–0.49] (0.27)	0.067 (0.052–0.074)/na	78
	VAR_2021_ ^a^	2021	15	—	0.23 [0.07–0.49] (0.27)	0.063 (0.051–0.075)/na	87
	VAR_2024_ ^a^	2024	40	—	0.21 [0.04–0.44] (0.23)	0.064 (0.049–0.074)/na	93
Storaa	STO_2010_	2010	34	0.14 [0.01–0.33] (0.15)	—	0.041 (0.032–0.051)/0.029 (0.024–0.039)	—
Hatchery strain 1	HAT1	1991–1993	34	0.88 [0.76–1.00] (0.12)	0.86 [0.63–0.98] (0.26)	0.018 (0.012–0.023)/0.017 (0.016–0.019)	—
Hatchery strain 2	HAT2	1995	34	0.96 [0.82–1.00] (0.08)	0.95 [0.86–0.99] (0.16)	—	—
	^a^	2012	6	0.95 [0.83–1.00] (0.07)	0.92 [0.82–0.98] (0.21)	—	—

*Note:* Average individual HAT admixture estimate, *q*, range and 90% confidence intervals (CI) are reported per collection both for 3656 SNPs and for the 288 SNP subset. *F*_st_ differentiation from HAT estimated with 288 SNPs is shown with 1000 95% bootstrap CI values in brackets. For HAT1, *F*_st_ gives the estimated differentiation from HAT2. The percentage of individuals showing no or low wild‐HAT admixture (*q* < 0.34, see text) are shown per collection.

^a^ Underlying SNP data are from Bekkevold et al. (2020) unless.

River Varde was stocked with a total of > 0.7 mil. 1/2−1y + old hatchery strain fish in 1977–2000 (details in Hansen et al. 2009). Since then, stocking of juveniles from wild broodstock has been conducted at varying intensity, ranging from 0 to ca. 50,000 fish per year.

For both rivers, collections from 2010 to 2024 (ca. seven generations after direct stocking with HAT ceased) were not expected to contain HAT but could contain *n*th generation backcrosses of HAT fish to wild fish. All hatchery production with wild‐caught broodstock adhered to the principle of using a minimum of 25:25 males: females crossed individually with gametes in equal proportions to attain a minimum genetically effective size of 50 in the stocked material. Neither spawning run size nor ratios between natural and hatchery‐produced offspring are known for the rivers.

Genotypes in samples from a third river, Storaa, were included in a subset of the analyses. The river Storaa is the neighbouring river of Skjern (river mouths separated by approximately 50 km, Figure 1), and demographic exchange between the two rivers is estimated to be substantial, as suggested by the lack of statistically significant population differentiation (Bekkevold et al. 2020). As strong admixture in Skjern led to relatively few sampled genotypes representing non‐admixed fish, we increased sample size by initially including genotypes from the Storaa in admixture analyses to represent river Skjern gene pools (see below). Although Storaa was also previously stocked with HAT, admixture is estimated to have been relatively low compared with river Skjern (Hansen et al. 2009), and samples were expected to represent unadmixed wild genotypes. The two closely related HAT strains used for stocking were represented by samples collected in 1991–1995, as well as by a small sample from 2012; that is, closely tracking HAT gene pools towards and after the final stages of stocking in the wild.

### 2.2 SNP Analysis

Genetic marker data were obtained in two ways. First, 235 genotypes from a 3.8 K candidate Illumina iSelect SNP array were selected from a population study describing genetic variation in trout populations across the Northeast Atlantic (Bekkevold et al. 2020). They identified regionally strong population structure and used a genotype‐environment association approach to demonstrate widespread correlations between SNP markers and a suite of parameters related to climate, hydrography and other environmental variables. A subset of 3656 of those SNPs was selected for the current study as they were polymorphic in HAT, Varde and Skjern river samples (at MAF > 0.05), and showed moderate linkage disequilibrium (here defined as *r* ^2^ between pairs of markers < 0.8). The resulting dataset represented the 40 trout linkage groups, LG, at varying densities (average is 94 SNPs per LG, range 13–162). One sample of 34 fish (VAR_2010_) was genotyped in connection with the current study, using the same Illumina iSelect SNP array as in Bekkevold et al. (2020), thus bringing the number of fish genotyped for 3656 SNPs to 269. To increase temporal inference for recent years, nine additional collections, three from Varde and six from Skjern (489 fish in total), were genotyped for a subset of 288 SNPs. This subset represented all 40 LG (1–16 SNPs per LG, average 5.9, Table S1), and was selected based on maximising statistical power to assign population of origin in Northeast Atlantic trout populations (Bekkevold, Knutsen, et al. 2024). PCR amplification and genotyping for the 288 SNPs typed in samples here were performed in 96.96 Dynamic Arrays using a Fluidigm IFC thermal cycler and BioMark instruments with SNPtypeTM chemistry. Genotypes were called using the BioMark Genotyping Analysis software (Fluidigm, San Francisco, California, USA). Validation of genotypes by genotyping 30 fish on both platforms showed 99% correspondence across all loci and individuals (7020 genotypes).

### 2.3 Multi‐Locus Admixture Analyses

Summary statistics (tests for deviation from Hardy–Weinberg proportions, sample and locus *F*_st_ differentiation estimated by Weir and Cockerham's Ɵ, exact *G* tests for sample differentiation) were calculated using GENEPOP v.4.1.4 (Raymond and Rousset 1995). 90% confidence intervals (CI) for Ɵ were generated by bootstrapping 1000 times over individuals, using the R package Hierfstat (Goudet 2005). Sample‐specific *H*_exp_ was estimated using the function *H*s in the R package *adegenet* (Jombart 2008).

Genetic relationships among samples were examined using PCA, and clustering of samples was visualised using DAPC implemented in *adegenet*. A genome‐wide admixture coefficient (*q*, the proportion of an individual's genome being of HAT origin) was estimated for wild‐caught fish and for HAT samples using *structure* v.2.3.4 (Pritchard et al. 2000). Both PCA and examination of *structure* trial runs for 1–5 K clusters suggested that within river all genotypes could be described by two clusters corresponding to HAT and the local wild genotypes (Skjern and Storaa samples made up a single cluster; see the (3) Results), justifying separate admixture analyses for, respectively, Varde and Skjern–Storaa populations. Allele frequencies were assumed to be correlated, and five replicate runs (to check consistency) were performed using 100 K MCMC (discarding the first 50 K MCMC as burn‐in). Pre‐stocking samples of fish of non‐admixed pure wild origin were not available. Individuals were therefore initially classified as pure wild or admixed based on their *q* estimate and 90% CI based on structure analyses of 3656 SNPs. In both rivers, individuals with *q* < 0.20 were initially classified as pure wild (‘WILD’). Analyses showed that HAT samples always exhibited lower 90% CI for *q* > 0.80. Individuals with *q* ranging 0.20–0.80 were therefore initially classified as being of admixed origin. The validity of this classification and the potential to classify individuals displaying different levels of admixture were tested by simulation. Using allele frequency estimates for 74 HAT and for, respectively, 24 Varde and 34 Skjern‐Storaa WILD fish (with *q* < 0.20), multi‐locus genotypes were simulated using the function ‘hybridise’ in *adegenet* to correspond with five admixture classes with increasing HAT admixture: (1) WILD; (2) Back‐crossed to wild, BC_WILD_ (F1 × WILD cross); (3) F1 (WILD × HAT cross); (4) Back‐crossed to HAT, BC_HAT_ (F1 × HAT cross); and (5) HAT. A total of 50 genotypes were simulated per admixture class, resulting in 250 in total per river. Simulated genotypes were examined in a clustering analysis using the same settings as above, and range and mean *q* values together with 90% CI were compared among classes. Finally, based on specific *q* thresholds observed in simulated data, all wild‐caught fish were classified as either ‘WILD’, ‘low admixture’ (*n*th generation back‐crossed to wild), ‘medium admixture’ (*n*th generation admixed cross), ‘high admixture’ (*n*th generation back‐crossed to HAT), or ‘HAT’ (see detailed criteria below).

A separate clustering analysis was performed for the dataset with the 288 SNPs only, to allow temporal comparison across all samples. There was correspondence between multi‐locus *q* values estimated with the two panels (see (3) Results) and these fish were therefore assigned to an admixture class, using the same criteria as for the full 3656 SNP dataset. Locus specific analyses suggested strong temporal change for the SNP ‘*Gdist:S499800_4765*’ which is associated with the maturation candidate gene *six6* (see below). This SNP was represented on both panels, and we tested whether admixture estimates might be biassed by inclusion of this locus by preforming a separate admixture analysis excluding information for this SNP in the reduced SNP data set (i.e., using 287 SNPs).

### 2.4 Locus‐Specific Admixture

Outlier tests were used to examine whether specific loci showed overall sample differentiation above or below expectations under neutrality. Using the Bayesian model implemented in *BayeScan* (Foll and Gaggiotti 2008) analyses were performed using settings recommended by the authors. Rivers were tested separately, pooling genotypes within rivers for collections from 2010 to 2012 and testing against the two HAT samples pooled. Loci returning a log_10_ Bayes factor above 0.5 and showing posterior probability *q* values below 0.05 (corrected for multiple sampling using false discovery rate procedure, FDR; Benjamini and Hochberg 1995) were considered statistical outliers. In addition to the *Bayescan* outlier test, we applied a complementary method, *signasel*, in an attempt to resolve whether temporal development in allele frequencies may reflect short‐term selection or random genetic drift (Hubert et al. 2018). The method uses temporal data and compares a model of pure genetic drift acting on the population versus a model including both genetic drift and directional selection. It thereafter establishes a likelihood ratio test (LRT) between the two models on a locus‐by‐locus basis. As we wanted to detect candidates of ongoing selection against hatchery introgressed regions, we based the analyses on selected genotypes showing a low to moderate level of genome‐wide introgression (individuals categorised as wild, low‐admixed or medium‐admixed by *structure q*, as described above) for each river by year. We analysed each river separately for both marker sets (6–8 temporal samples per river typed for 288 SNPs, or 2–3 temporal samples per river typed for 3656 SNPs). The strength of *signasel* relies on the availability of multiple temporal samples. We hence expected results generated with fewer temporal replicates (3656 SNP data) to exhibit more false positives than tests with more replicates (288 SNP data). Using a temporal method, Hansen et al. (2009) estimated river‐specific effective population sizes, *N*_*e*_, at *N*_*e_*Skjern_ = 133 (95% CI 91–193) and *N*_*e_*Varde_ = 224 (95% CI 55–452). We assumed a generation time of 3.5 years and tested two different *N*_*e*_ scenarios, assuming a constant *N*_*e*_ of either 100 or 200, representing, respectively, values closer to point estimates/upper versus lower estimates in the two rivers. The lower *N*_e_ scenario also represents a model with more conservative assumptions about selection, that is, when drift is expected to play a relatively large role. We considered temporal outlier loci as those with a *p*‐value lower than 0.05 after FDR correction.

A genomic cline approach implemented in the programme INTROGRESS (Gompert and Buerkle 2010) was applied to test the hypothesis that introgression was uniform across genomic regions and loci. INTROGRESS uses multinomial regression to evaluate whether individual loci show either higher than expected introgression rates (i.e., indicative of increased introgression) or lower introgression than expected under neutrality (i.e., indicative of reduced introgression) among individuals of varying genome‐wide admixture. Genome‐wide admixture was estimated using hybrid index estimation built into the programme, calculated as the proportion of HAT genes of an individual. Index calculation requires information about allele frequencies in non‐admixed individuals from both contributing gene pools, which was available for HAT only. We therefore used the subsamples of individuals classified as WILD, based on *structure q* values, as proxies for pure Varde and Skjern allele frequencies. No locus was differentially fixed between WILD and HAT samples and the majority of loci showed relatively weak or lacking differentiation. Loci exhibiting weak differentiation between source gene pools are non‐informative and are commonly deselected in hybridisation marker studies (Gompert et al. 2012). Data were therefore restricted to including 765 markers selected by meeting one or more of the criteria: (1) loci driving the main differentiation between HAT and wild‐caught samples (SNP loading at upper 5% for PC1 in the DAPC analysis); (2) loci exhibiting global *F*_st_ > 0.03 (average multi‐locus *F*_st_ between HAT and wild caught samples); and (3) loci exhibiting outlier behaviour in either river. A hybrid index was first calculated for all wild‐caught samples. Null models were then generated using simulations as described in Gompert and Buerkle (2010). Briefly, a large simulated admixed population was generated based on expected genotype frequency distributions estimated by hybrid indices and heterozygosity values equal to the observed data. A total of 2000 admixed individuals were simulated using a permutation model. Deviation from neutrality was assessed by *p*‐values and comparison of observed versus expected genotype distributions. Allele frequency clines that are steeper than expectations under neutral processes or display heterozygote deficit indicate reduced introgression, whereas shallower clines and heterozygote excess indicate increased introgression (Gompert and Buerkle 2009). All significance thresholds were adjusted using FDR.

The frequencies of outlier loci and of loci exhibiting trends for reduced introgression were compared across temporal samples by river. Locus specific associations with environmental variables reported in Bekkevold et al. (2020) were examined and, using the BWA‐mem algorithm (Li and Durbin 2009) loci were mapped to the reference genome assemblies of *S. salar* (Lien et al. 2016) and *S. trutta* (fSalTru1.2). FASTA sequences from the outlier loci that overlapped between *signasel* and *Bayescan* results were blasted using online blastn suite at NCBI (Altschul et al. 1990).

## 3 Results

### 3.1 Sample Differentiation

A total of 758 fish were included, respectively 269/489 fish typed for 3656/288 SNPs. *H*_exp_ estimated with 3656 SNPs varied little across collections for both SNP datasets, with SKJ_2001_ and HAT exhibiting slightly lower estimates than all remaining samples (Table S1). Twelve deviations from HWE were observed across 3656 loci following FDR correction (equalling 0.3% of all tests; Table S2), and all loci were retained in the analyses. Except for temporal comparisons within Varde, all pairwise tests for sample differentiation were significant at *p* < 0.01 following FDR correction. Estimated with 3656 SNPs, differentiation between the two HAT strains was moderate (*F*_st_ 0.017, Table 1) and in subsequent analyses HAT genotypes were pooled to reduce complexity. Differentiation between HAT and wild fish was low (all *F*_st_ < 0.05) and increased with time since stocking (Table 1). *F*_st_ between rivers increased from 0.008 in the 2001–2004 samples to 0.012 in the 2010 samples, with no overlap in bootstrap CI (respectively, 0.007–0.009 and 0.011–0.013). When including more recent collections genotyped for the 288 SNPs, *F*_st_ differentiation from HAT increased further; in Varde from 0.037 to 0.065 from 2004 to 2024, and in Skjern from 0.019 to 0.054 from the year 2001 to 2024.

In the PCA analysis with 3656 SNPs (Figure 1) fish were differentiated by HAT versus wild, with PC1 (explaining 2% variance). HAT samples collected ca. 20 years apart clustered together. PC2 (1% variance explained) split populations Varde vs Skjern–Storaa. The DAPC analysis clustered samples by HAT versus wild/HAT‐wild admixed (PC1 explaining 100% variance, Figure S1).

### 3.2 Genome‐Wide Admixture Over Time

Admixture analysis for 3656 SNPs with *structure* and using Evanno's delta *K* indicated that *K* = 2–3 were the most likely models (Table S3), where *K* = 2 corresponded with wild versus HAT fish. The *K* = 3 clusters corresponded with respectively HAT, Varde and Skjern–Storaa genotypes and mixtures thereof (Figure S2). To control for population structure, subsequent admixture analyses were carried out separately for Varde and Skjern–Storaa. When analyses were repeated for individual rivers, setting *K* = 2 and including simulated genotypes, *q* values ranged from 0 to 1 in both rivers and for simulated fish. There was a consistent decrease in average *q* with generations since stocking with HAT ended (Table 1). In simulations, admixture samples showed *q* values close to theoretical expectations for each admixture class (Table 2, Figure S3). Simulated *q* estimates did not overlap between admixture classes, but 90% CI often overlapped. While acknowledging that real genotypes would represent a mosaic of backcross admixture classes, criteria used for classifying the admixture status of wild trout were set based on minimising overlap in 90% CI. Individuals were classified based on the same *q* criteria in both rivers, as simulations indicated that this would not bias inference.

**TABLE 2:** Trout Admixture Estimates, *q* (Proportion Genome From HAT Estimated With 3656 SNPs) for Simulated Genotypes of Five Admixture Classes (Each *N* = 50).

Simulated admixture class	Expected mean *q*	SIM_Skjern observed average *q* (range) [90% CI]	SIM_Varde observed average *q* (range) [90% CI]	Classification criteria for wild‐caught fish in both rivers (*q* range)
Non‐admixed pure wild	0	0.03 (0.01–0.12) [0.00–0.19]	0.02 (0.01–0.06) [0.00–0.12]	WILD (0.00–0.12)^a^
BC_WILD_	0.25	0.22 (0.15–0.33) [0.09–0.40]	0.21 (0.13–0.28) [0.07–0.34]	Low admixture (0.13–0.34)^b^
F1	0.50	0.51 (0.43–0.60) [0.37–0.67]	0.47 (0.41–0.54) [0.35–0.60]	Medium admixture (0.35–0.60)
BC_HAT_	0.75	0.76 (0.66–0.82) [0.60–0.89]	0.74 (0.67–0.80) [0.61–0.85]	High admixture (0.61–0.84)
HAT	1	0.98 (0.92–1.00) [0.86–1.00]	0.98 (0.93–0.99) [0.84–1.00]	HAT (0.84–1.00)

^a^ Range was set to minimise erroneous categorisation of lightly admixed fish as pure wild; criterion is based on lower *q* for simulated back‐crosses to pure wild being 0.15 and 0.13 in Skjern and Varde.

^b^ Range was set to minimise false categorisation of F1 as weakly admixed; criterion is based on lower *q* for simulated F1 genotypes being ≥ 0.35 in both Skjern and Varde.

Admixture estimates based on 288 SNPs exhibited slightly inflated *q* and twice as broad CI compared to estimates generated with 3656 SNPs (Table 1), suggesting that estimates based on fewer loci were less precise and may have slightly overestimated HAT admixture. Nonetheless, there was an overall positive correlation between *q* estimates with the two marker sets estimated at *r* ^2^ = 0.80 (Figure S4) and admixture class frequencies were tentatively based on estimates generated with 288 SNPs.

Individual admixture estimates were not affected by inclusion or exclusion of the SNP associated with the candidate gene *six6* (*y* = 0.993*x*–0.004; *r* ^2^ = 0.995) and in no case did classifications change category, showing that this candidate locus did not drive admixture estimates.

**FIGURE 2:**
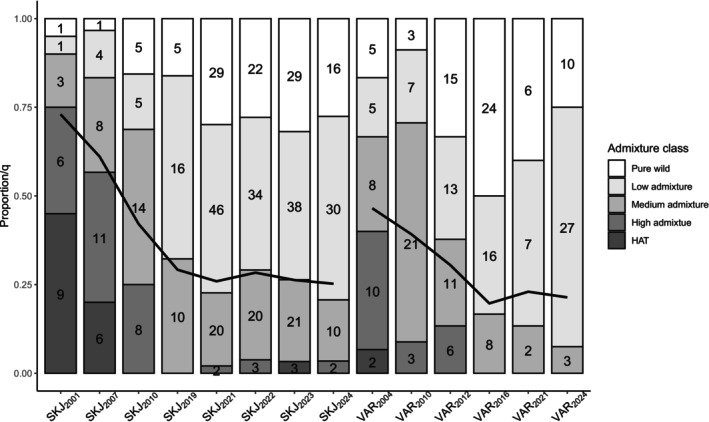
Temporal development in frequencies of five admixture classes of *S. trutta* from rivers Skjern and Varde, as assessed by admixture analysis based on 288 SNPs. Numbers of fish per class are indicated inside bars. Lines show average HAT admixture, *q*, per collection.

Individual classification of samples collected the first generation after HAT stocking ended showed that both rivers contained spawning run fish with HAT genotypes as well as individuals of pure wild and broad spectra of admixed origins (Figure 2). Approximately three generations later, no fish with pure HAT genetic profiles were present in collections, and the proportion of fish with genotypes consistent with a pure wild origin had at least doubled. Nonetheless, admixture analyses indicated that substantial proportions of individuals exhibited admixture, and although average *q* initially decreased in both rivers, there was a trend for *q* to reach a plateau after 2016 (Figure 2). Recent samples from Skjern (2019 to 2024) were consistently estimated to harbour 26%–29% HAT input with no indication of a drop. These samples also contained fish with high HAT admixture estimates (2, 3, 3 and 2 fish shown as dark grey bars in Figure 2). While no substantial releases of HAT fish took place after 1996, in 2021–2022 there was suspicion of unsanctioned (numerically small) HAT releases in a local Skjern tributary, which may explain the seeming reappearance of these genotypes.

### 3.3 Locus‐Specific Divergence and Introgression

*Bayescan* outlier tests identified 10 and 2 loci in Varde and Skjern, respectively, as statistical outliers; all exhibiting larger than expected differentiation between HAT and wild fish (Table S1). One locus overlapped between rivers. When using the *signasel* test for detection of short‐term selection, four and one outliers were detected for the 288 SNP panel, and 424 and seven for the 3656 SNP panel for Skjern and Varde, respectively, for *N*_e_ = 200 (Table S1). As expected, overlapping, but fewer, candidate outliers were obtained when assuming *N*_e_ = 100: two and one for the 288 SNP panel, and 172 and two for the 3656 SNP panel for Skjern and Varde, respectively. In Skjern, only three *signasel* outliers overlapped with *Bayescan* outliers, and only one (‘*Gdist:S165349_3716*’) retained statistical significance following FDR correction. In Varde, one locus (*‘Gdist:S499800_4765’*) initially overlapped between *Bayescan* and *signasel* tests, but lost statistical significance following FDR for the latter test.

Genomic cline analyses indicated that introgression rates were variable among loci. After FDR correction, 28 (4%) and 64 (8%) loci showed evidence of a non‐neutral introgression pattern in Skjern and Varde, respectively. Only two loci overlapped between populations, both showing evidence of increased introgression. In Varde, increased introgression was indicated for a total of 28 loci, including two outlier loci, and 36 loci (none of which were outliers) displayed reduced introgression rates. Identified loci were spread across 30 LGs in total, with 1–5 loci per LG (Table S1). In Skjern, increased introgression was indicated in 12 loci. Sixteen loci displayed reduced introgression. Loci were scattered over 15 LGs with 1–5 per LG, and none also exhibited outlier behaviour.

**FIGURE 3:**
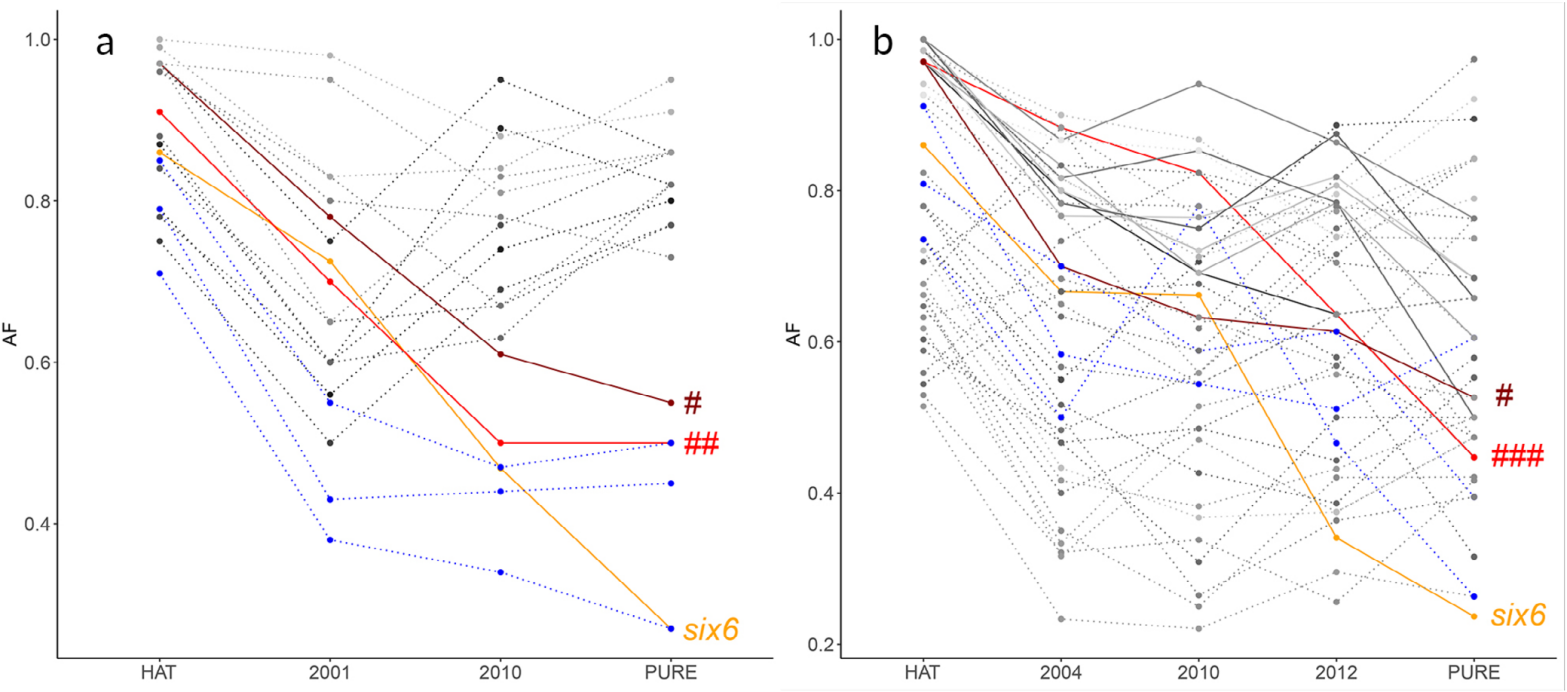
Temporal development in allele frequencies, AF, in *S. trutta* in the rivers Skjern (a), and Varde (b) for SNPs exhibiting reduced introgression (connected by dotted lines) and outlier behaviour (solid lines). AF estimates in HAT and in fish classified as genetically wild (PURE) are shown for comparison. Point estimates are connected by lines to aid interpretation. The locus ‘*Gdist:S307891_8631*’ showing outlier behaviour in both populations is shown in dark red and marked #. The locus ‘*Gdist:S499800_4765*’ associated with the *six6* gene is shown in orange and marked ‘*six6*’. Two loci with marked AF change away from the allele dominating in HAT are shown in red and, respectively, notation ##: ‘Gdist:S165349_3716’, and ###: ‘cDNA:S822390_3958’. Respectively three and three loci (none overlapping between rivers) showing reduced introgression and strong AF difference between HAT and wild collections, suggestive of de‐selection under natural conditions, are indicated in blue.

Twenty‐six of the reduced introgression loci and outlier loci showed association with predicted candidate gene regions (Table S1). All outlier loci, and 15 loci exhibiting reduced (eight loci) or increased (seven loci) introgression also showed association with one or more environmental parameters, as identified across North European geographical scales (Bekkevold et al. 2020; Table S1). Environmental parameters associated with loci showing reduced introgression included salinity at marine entry from river, ambient river temperature and pH at spawning location. When examining loci showing reduced introgression and/or being outlier loci, allele frequencies mostly showed weak or inconsistent temporal changes away from the variant dominating in HAT, suggesting lack of strong selection under wild conditions (Figure 3). Noticeable exceptions to this pattern included three loci with delta AF decreasing by more than 30% in one or both rivers the first decade after HAT stocking ceased. One was the locus (‘*Gdist:S499800_4765*’) mapping to within 2020 bp of the *six6* gene in *S. salar* . In both Varde and Skjern it displayed allele frequency changes away from the variant that was nearly fixed in HAT (Figure 3). In data generated with the 288 SNPs, AF of the HAT‐associated allele had by 2024 decreased by 50% and 34% in Skjern and Varde, respectively (Figure S5). The SNP was initially flagged as an outlier in the *signasel* test for Varde but lost its signal after FDR correction (see above). In Skjern, the outlier ‘*Gdist:S165349_3716*’ showed temporal change in gene frequencies away from those observed in HAT (Figure 3a, ‘##’). The locus maps to an *O*‐acetyl‐ADP‐ribose deacetylase gene in *S. salar* , belonging to a family of protein lysine deacetylases, which regulate epigenetic gene silencing, metabolism, life span and chromatin structure (Chen et al. 2011). Further, the SNP is within a potential ‘supergene’ on chromosome 28 that harbours genetic variation linked to sex specific differences in trait optima (M. Sodeland, M. M. Hansen, D. Bekkevold, T. Haraldstad4, I.K. Mellerud, H. Sannæs, A. Slettan, K. Bleeker, K. Glover, and H. Knutsen. unpublished results). In Varde, this SNP showed no evidence of temporal change and, in contrast to in Skjern, maintained high frequencies (0.67–0.72) of the allele that was nearly fixed in HAT (not shown). Conversely, the outlier SNP ‘*cDNA:S822390_3958*’ showed consistently large delta AF in Varde (see Figure 3b, ‘###’) and not in Skjern, and statistically marginally significant evidence of reduced introgression in Varde (Table S1). The SNP is associated with salinity conditions at river discharge across Northeast European trout populations (Bekkevold et al. 2020), suggestive of being of adaptive significance. Finally, six loci (three in Skjern, three in Varde) showing reduced introgression exhibited consistently strong divergence between HAT and wild fish (Figure 3), suggestive of being de‐selected under wild conditions. None was associated with known candidate gene regions, except ‘*cDNA:S41119_1719*’, which was associated with a C3a anaphylatoxin chemotactic receptor gene, involved in chemotaxis and immune response.

## 4 Discussion

### 4.1 Genome‐Wide Admixture

Despite overall weak genetic differentiation between wild populations and the stocked hatchery strain we were able to show that genome‐wide admixture, estimated by the proportion gene input from HAT, decreased over time, indicative of low fitness of hatchery and strongly admixed fish leading to de‐introgression and partial recovery of genetic profiles. Over the first ca. two and a half generations, average individual admixture dropped by ca. 40%–60% in the two rivers. Thus, in Varde, admixture was estimated to drop 39% after 8 years. When including information for the recent samples typed for 288 SNPs, we observed a drop in admixture of 55% over ~six generations. In Skjern, the drop in admixture was initially even more dramatic, coming out at 61% from 2001 to 2010. When including the most recent samples, the drop was 64% over ~six generations, suggestive of stabilisation. Admixture estimates may be less accurate for samples typed only for a subset of SNP markers, potentially masking continued convergence towards pre‐impact gene profiles. Yet, stabilising admixture estimates may also reflect that recombination gradually disconnects SNP markers from candidate genes for local mal‐adaptations. Over time, an increasing number of SNPs are hence expected to become non‐informative with respect to ongoing population recovery, as they reflect historical admixture events. Nonetheless, seven generations after stocking with HAT ended, proportions of fish with wild or near‐wild genotypes continued to increase in both rivers. The results therefore point to two likely co‐occurring processes. On the one hand, introgressed ‘hybrid swarms’ are sustained with initial dilution of, and selection against HAT genetic input, potentially reaching a plateau in recent years. On the other, contingents of non‐admixed individuals appear to have persisted in both populations, even in the face of strong overall introgression. ‘Subpopulations’ of genetically wild fish may become increasingly prolific, once swamping effects by directed release of HAT are lessened and may be instrumental in speeding up recovery (Baskett et al. 2013). This scenario is consistent with predictions for the Skjern population where diverging peak spawning times between HAT and wild fish may have decreased the probability of wild/HAT matings and ensured the persistence of non‐admixed fish in upper, less stocking‐affected, reaches of the river (Hansen and Mensberg 2009). The observation that a SNP associated with the *six6* gene linked with maturation timing in Atlantic salmon showed consistent allele frequency divergence between HAT and wild fish in both Skjern and Varde, is suggestive of divergent selection pressures related to maturation phenology under hatchery conditions and in the wild (Thorpe 2004). Moreover, a recent study comparing reaction norms for growth in wild Varde and HAT trout shows that HAT and HAT‐admixed fish display divergent (increased) growth rates that may be mal‐adaptive under wild conditions (Bekkevold, Knutsen, et al. 2024). It is thus possible that cascading effects on life‐history traits related to migration and spawning behaviour reduce the scope for interbreeding between genetically indigenous and admixed individuals, as observed in other salmonids (Webb et al. 1991; Pritchard et al. 2018).

Stocking with fish produced from a relatively small broodstock may accelerate genetic drift and decrease *N*_e_ in natural populations (Ryman and Laikre 1991) including those studied here (Hansen et al. 2009). Irrespective of broodstock genetic background (HAT vs. native), stocking could thus have affected allele frequency changes compared to under completely unaltered dynamics. However, broodstocks always included 50–100 wild‐caught fish contributing gametes to offspring in roughly equal proportions, and there was no evidence of relatedness among sampled fish (not shown). We do therefore not expect strong effects of supplementary stocking on the development of gene profiles in recent years and suggest that changes at least mainly reflect natural processes. Individual classification by admixture criteria based on simulated genotypes is a simplistic approach, as real genetic profiles most likely consist of complex hybrid mosaics following decades of hybridisation and introgression. Here, pure wild genotypes were simulated based on a relatively small number of fish (20–34) that could have displayed some (low) degree of HAT admixture. Although this may have biassed inference about the relative proportions of different classes of admixed fish in individual samples, and hence assumptions about *q* in different admixture classes, the main conclusion that collections of wild‐caught spawners over time exhibit both more individuals displaying low admixture and fewer strongly admixed genotypes should be robust. Thus, although statistical accuracy to identify the admixture status of an individual in some cases was relatively low, our approach validates the prospects for monitoring effects of introgression on wild populations (Hansen et al. 2012).

### 4.2 Differential Introgression Among Loci

Genomic cline analyses have provided evidence for ecological divergence in the maintenance of species boundaries in hybrid zones in the face of gene flow, through the identification of reduced introgression at genomic regions associated with candidate genes for functional traits (e.g., Walsh et al. 2016). Locus‐specific introgression processes are more rarely examined within species, as lower genetic differentiation decreases statistical power to identify genomic regions exhibiting divergent introgression patterns (but see Lamaze et al. 2012; Ozerov et al. 2016). Our analyses show that introgression from stocked strains is genome‐wide but also suggest a role for selection in shaping introgression rates at specific genomic regions. Several loci showed signs of being arrested or slowed from entering the wild gene pools, as would be expected under a scenario with introgression from a domesticated strain harbouring gene variants that are deselected under wild conditions. Nonetheless, clines were in all cases relatively shallow, suggesting that the majority of identified loci were not direct targets of selection but could be subject to genomic hitch‐hiking effects, or reflect adaptive alleles of small effect (Gompert et al. 2013). Approximately half of the loci exhibited stronger introgression than expected under neutral admixture, suggesting that gene variants of HAT origin may be positively selected in the wild, perhaps via recovery of fitness following release from hatchery selection pressures (Tufto 2017). Nonetheless, it is also possible that the relatively short generational time scale analysed here cannot accurately capture selective responses (Yang et al. 2019). In genomic cline analysis, rates of false positives are expected to increase under low differentiation and at strong genetic drift, that is, at low *N*_e_ (Gompert and Buerkle 2009), both of which may have affected our results. *N*_*e*_s are estimated at ~250 in these populations (Hansen et al. 2009), and genetic drift could thus have increased rates of false positives.

Outlier SNPs were in some cases associated with genome regions subject to selection in other species. A compelling candidate was a SNP associated with the *six6* gene. *six6* belongs to a class of genes widely expressed across vertebrate taxa (Pritchard et al. 2018; Moustakas‐Verho et al. 2020) involved in eye and brain development. In Atlantic salmon, it shows a strong association with age at maturation (Barson et al. 2015; Besnier et al. 2024) and displays sex‐specific epistasis with the maturation gene *vgll3* (Besnier et al. 2024). *Six6* has not previously been identified as a candidate gene in trout, and as such, this result represents a novel finding. Although the specific pathway is unknown for the HAT strain used here, (inadvertent) selection for early maturation can be prominent under hatchery conditions (Ayllon et al. 2019; Harvey et al. 2018; LaCava et al. 2023). This may have led to near fixation of a variant predisposing for early maturation, whereas fitness optima in the wild are likely to display different, and likely more balanced, adaptive gene frequencies (Barson et al. 2015; Besnier et al. 2024). The fact that the locus showed strong temporal change and outlier behaviour, with no evidence of reduced introgression, and no general outlier behaviour across wild trout populations in the Northeast Atlantic (Bekkevold et al. 2020), is in correspondence with the expectation that maturation genes are subject to balancing selection processes in relation to local adaptation that may be lost in analyses stratifying for population structure (Sinclair‐Waters et al. 2020). In Skjern, the allele frequency change in the *six6*‐associated SNP away from the HAT dominant variant was congruent with an *ad hoc* observation in a recent (December 2022) collection of 22 mature trout from the neighbouring, closely related river Storaa population, which was historically less affected by HAT gene pools. In Storaa, the HAT associated allele was estimated at 0.36 and thus similar to estimates in inferred pure wild trout from Skjern and Varde (0.32–0.35; Figure S5). Further characterisation of the *six6* gene region and association with life history variations is warranted, also for trout. It is nonetheless suggestive that a domesticated hatchery strain used for stocking the British river Glaven (Piper et al. 2025) shows near‐fixation of the same SNP allele as in demographically unrelated Danish hatchery strain trout analysed here.

For the two loci showing (1) association with salinity environment at river mouth on broad geographical scale (Bekkevold et al. 2020); (2) strong allele frequency change over time; and (3) reduced introgression, it can be hypothesised that data reflect mal‐adaptation of HAT strains founded with fish originating from brackish western Baltic Sea populations (cf. Hansen et al. 1997) when exposed to a life‐cycle entailing anadromous migration into fully saline North Sea conditions. Loci potentially under selection were identified across several linkage groups, corresponding with general expectations for wild‐culture diversification to act on multiple traits (Wringe et al. 2016). Quantitative traits are commonly governed by multiple loci, each showing weak differentiation and potentially epistasis, but collectively having strong phenotypic effect (Slate 2005), and the generally low HAT‐wild allele frequency differences therefore need not translate into lack of de‐selection in the wild. Nonetheless, modelling also predicts that upon release into the wild, the fitness of hatchery strains can exhibit a fast initial decline, followed by slower decline in subsequent generations, irrespective of the strength of natural selection on life history traits (Tufto 2017). We did not examine fitness of different admixture classes and our results do hence not allow us to distinguish between these processes. Juvenile growth rates vary positively with the degree of HAT admixture in Varde trout under common‐garden conditions (Bekkevold, Besnier, et al. 2024), indicating divergent HAT‐wild selection pressures. There was, however, no indication that the rate of change in genome‐wide admixture proportion showed a decreasing trend in later generations, in comparison with the first generations after stocking ended.

### 4.3 Variance in Introgression Between Populations

In contrast to studies in species hybrid zones (Lexer et al. 2007) our data suggest that introgression affected loci in dissimilar patterns in the two populations. In spite of roughly concordant temporal changes in genome‐wide admixture in Varde and Skjern, the two populations showed different proportions and identities of loci with deviant introgression patterns and displaying outlier behaviour. This suggests that, even between populations within a common ecoregion, there is intraspecific variation in how specific genomic regions respond to introgression, as seen in other salmonid fishes (e.g., Leitwein et al. 2021). This may point to HAT genes exerting different selective pressures in dissimilar environments and genetic backgrounds (Ozerov et al. 2016; Zueva et al. 2020). Populations with different phylogeographic histories varying in standing genetic variation may respond differently to the same selective pressure, resulting in different loci being associated with selection (Therkildsen et al. 2019; Zueva et al. 2020; Leitwein et al. 2021). Our results hence also point to the fact that different sets of genetic markers yield different power for estimating genome‐wide introgression dynamics across populations.

Releases of domesticated salmonid strains may decrease overall population structure (e.g., Glover et al. 2012). Our results show that admixture can be monitored with relatively small marker panels, even when gene pools are weakly diverged, and that migration‐drift relationships, as estimated with *F*_st_, appear to gradually recover with time since the last impact. However, seven generations after HAT stocking ended, wild populations were still showing manifestation of HAT gene pools. Temporally developing de‐introgression and population‐specific genomic responses to hatchery strain introgression are likely linked with selection against mal‐adaptive gene variants, such as those related to maturation timing. However, monitoring results also point to a cessation of genome‐wide return to pre‐impact gene pools, suggesting that hatchery strain introgression is not fully reversible. The genetic remains from hatchery gene pools may, at least partially, reflect gradual decoupling between SNP markers and fitness traits. Population size is not known for either river in our study, but angler catch data are available from 2002 and onwards in River Skjern (www.skjernaasam.dk) and from 2016 in River Varde (www.varde‐sportsfiskerforening.dk). Catches are not controlled for effort and may therefore not reliably reflect population size differences between the two rivers. From 2002 to 2024, River Skjern catches almost tripled from ca. 240 to 560 trout per year. However, the major increase in catches happened before 2016, and catches plateaued at ca. 550 in the past 8 years. HAT admixture estimates also plateaued during the same period. In comparison, Varde catches increased from ca. 500 to 1800 trout annually over the same 8 years. During this period, HAT admixture estimates also plateaued for this population, albeit at a lower admixture level than in River Skjern. Estimated spawning and nursery habitat in River Skjern is more than twice that of River Varde (Hansen et al. 2009). This would predict overall larger population sizes in Skjern than in Varde, whereas the opposite is indicated in catches. It remains of conservation interest to determine to which extent this could be caused not just by extrinsic drivers of population regulation, such as juvenile habitat quality and predation, adult survival at sea, and stochastic events, but also by intrinsic drivers, such as stocking‐induced mal‐adaptations that still prevent full fitness restoration.

## Author Contributions

Initial study conception by D.B. and E.E.N. with substantial contributions from K.A.G., F.B. and B.J.‐M. on planning, analysis and interpretation of data; Analyses conducted by D.B. and B.J.‐M. The original draft of the manuscript was prepared by D.B. with revisions and edits provided by all authors.

## Disclosure

Benefit Sharing Statement: Benefits from this research include the sharing of our data and results, including on public databases as described below.

## Conflicts of Interest

The authors declare no conflicts of interest.

## Supporting information


Appendix S1.
**Table S1.** List of SNP loci in analysis, with information about BLAST results, outlier and introgression results per locus.
**Table S2.** Locus specific tests for HWE in analysed samples.
**Table S3.** *Structure* delta *K* estimates in collected samples.
**Figure S1.** DAPC plot of *S. trutta* samples typed for 3656 SNPs for *K* = 2.
**Figure S2.** *Structure* plot of *q* estimates for collected samples.
**Figure S3.** Structure plot of *q* estimates for simulated genotypes.
**Figure S4.** Relationship between individual *q* estimates generated with 3656 and 288 loci.
**Figure S5.** Allele frequencies for the SNP ‘*Gdist:S499800_4765*’ associated with the maturation gene *six*6 in temporal collections.

## Data Availability

Data underlying this article are available as online Supporting Information and genotype data for all fish are found in the DTU Data repository at https://data.dtu.dk/ with the DOI: 10.11583/DTU.28675775, and scripts can be found in the public GitHub repository *SeaTrout_Genetic_Recovery*.

## References

[mec70036-bib-0001] Allendorf, F. W. , R. F. Leary , P. Spruell , and J. K. Wenburg . 2001. “The Problems With Hybrids: Setting Conservation Guidelines.” Trends in Ecology & Evolution 16: 613–622. 10.1016/S0169-5347(01)02290-X.

[mec70036-bib-0002] Altschul, S. F. , W. Gish , W. Miller , E. W. Myers , and D. J. Lipman . 1990. “Basic Local Alignment Search Tool.” Journal of Molecular Biology 215: 403–410. 10.1016/S0022-2836(05)80360-2. 2231712

[mec70036-bib-0003] Ayllon, F. , M. F. Solberg , K. A. Glover , et al. 2019. “The Influence of vgll3 Genotypes on Sea Age at Maturity Is Altered in Farmed Mowi Strain Atlantic Salmon.” BMC Genetics 20: 44. 10.1186/s12863-019-0745-9. 31060499 PMC6501413

[mec70036-bib-0004] Barson, N. J. , T. Aykanat , K. Hindar , et al. 2015. “Sex‐Dependent Dominance at a Single Locus Maintains Variation in Age at Maturity in Salmon.” Nature 528: 405–408. 10.1038/nature16062. 26536110

[mec70036-bib-0005] Baskett, M. L. , S. C. Burgess , and R. S. Waples . 2013. “Assessing Strategies to Minimize Unintended Fitness Consequences of Aquaculture on Wild Populations.” Evolutionary Applications 6: 1090–1108. 10.1111/eva.12089. 24187590 PMC3804241

[mec70036-bib-0006] Baskett, M. L. , and R. S. Waples . 2013. “Evaluating Alternative Strategies for Minimizing Unintended Fitness Consequences of Cultured Individuals on Wild Populations.” Conservation Biology 27: 83–94. 10.1111/j.1523-1739.2012.01949.x. 23082984

[mec70036-bib-0007] Bekkevold, D. , F. Besnier , T. Frank‐Gopolos , E. E. Nielsen , and K. A. Glover . 2024. “Introgression Affects *Salmo trutta* Juvenile Life‐History Traits Generations After Stocking With Non‐Native Strains.” Evolutionary Applications 17: e13725. 10.1111/eva.13725. 38962360 PMC11219512

[mec70036-bib-0008] Bekkevold, D. , J. Hojesjo , E. E. Nielsen , et al. 2020. “Northern European *Salmo trutta* (L.) Populations Are Genetically Divergent Across Geographical Regions and Environmental Gradients.” Evolutionary Applications 13: 400–416. 10.1111/eva.12877. 31993085 PMC6976966

[mec70036-bib-0009] Bekkevold, D. , H. Knutsen , J. Hemmer‐Hansen , et al. 2024. “Genetic Monitoring Uncovers Long Distance Marine Feeding Coupled With Strong Spatial Segregation in Seatrout (*Salmo trutta* L.) Consistent at Annual and Decadal Time Scales.” ICES Journal of Marine Science 81: 1655–1668. 10.1093/icesjms/fsae114.

[mec70036-bib-0010] Benjamini, Y. , and Y. Hochberg . 1995. “Controlling the False Discovery Rate: A Practical and Powerful Approach to Multiple Testing.” Journal of the Royal Statistical Society. Series B, Statistical Methodology 57: 289–300. https://www.jstor.org/stable/2346101.

[mec70036-bib-0011] Bernatchez, L. 2001. “The Evolutionary History of Brown Trout ( *Salmo trutta* L.) Inferred From Phylogeographic, Nested Clade, and Mismatch Analyses of Mitochondrial DNA Variation.” Evolution 55: 351–379. 10.1111/j.0014-3820.2001.tb01300.x. 11308093

[mec70036-bib-0012] Besnier, F. , F. Ayllon , Ø. Skaala , et al. 2022. “Introgression of Domesticated Salmon Changes Life History and Phenology of a Wild Salmon Population.” Evolutionary Applications 15: 853–864. 10.1111/eva.13375. 35603027 PMC9108307

[mec70036-bib-0013] Besnier, F. , Ø. Skaala , V. Wennevik , et al. 2024. “Overruled by Nature: A Plastic Response to Environmental Change Disconnects a Gene and Its Trait.” Molecular Ecology 33: e16933. 10.1111/mec.16933. 36942798

[mec70036-bib-0014] Bolstad, G. , K. Hindar , G. Robertsen , et al. 2017. “Gene Flow From Domesticated Escapes Alters the Life History of Wild Atlantic Salmon.” Nature Ecology & Evolution 1: 0124. 10.1038/s41559-017-0124. 28812692

[mec70036-bib-0015] Bolstad, G. H. , S. Karlsson , I. J. Hagen , et al. 2021. “Introgression From Farmed Escapees Affects the Full Life Cycle of Wild Atlantic Salmon.” Science Advances 7: eabj3397. 10.1126/sciadv.abj3397. 34936452 PMC8694624

[mec70036-bib-0016] Castellani, M. , M. Heino , J. Gilbey , H. Araki , T. Svasand , and K. A. Glover . 2015. “IBSEM: An Individual‐Based Atlantic Salmon Population Model.” PLoS One 10: e0138444. 10.1371/journal.pone.0138444. 26383256 PMC4575158

[mec70036-bib-0017] Castellani, M. , M. Heino , J. Gilbey , H. Araki , T. Svåsand , and K. A. Glover . 2018. “Modeling Fitness Changes in Wild Atlantic Salmon Populations Faced by Spawning Intrusion of Domesticated Escapees.” Evolutionary Applications 11: 1010–1025. 10.1111/eva.12615. 29928306 PMC5999203

[mec70036-bib-0018] Chen, D. , M. Vollmar , M. N. Rossi , et al. 2011. “Identification of Macrodomain Proteins as Novel O‐Acetyl‐ADP‐Ribose Deacetylases.” Journal of Biological Chemistry 286: 13261–13271. 10.1074/jbc.M110.206771. 21257746 PMC3075673

[mec70036-bib-0019] Diserud, O. H. , P. Fiske , S. Karlsson , et al. 2022. “Natural and Anthropogenic Drivers of Escaped Farmed Salmon Occurrence and Introgression Into Wild Norwegian Atlantic Salmon Populations.” ICES Journal of Marine Science 79: 1363–1379. 10.1093/icesjms/fsac060.

[mec70036-bib-0020] Edmands, S. 2007. “Between a Rock and a Hard Place: Evaluating the Relative Risks of Inbreeding and Outbreeding for Conservation and Management.” Molecular Ecology 16: 463–475. 10.1111/j.1365-294X.2006.03148.x. 17257106

[mec70036-bib-0021] Foll, M. , and O. Gaggiotti . 2008. “A Genome‐Scan Method to Identify Selected Loci Appropriate for Both Dominant and Codominant Markers: A Bayesian Perspective.” Genetics 180: 977–993. 10.1534/genetics.108.092221. 18780740 PMC2567396

[mec70036-bib-0022] Fraser, D. J. , L. K. Weir , L. Bernatchez , M. M. Hansen , and E. B. Taylor . 2011. “Extent and Scale of Local Adaptation in Salmonid Fishes: Review and Meta‐Analysis.” Heredity 106: 404–420. 10.1038/hdy.2010.167. 21224881 PMC3131967

[mec70036-bib-0023] Glover, K. A. , H. Ottera , R. E. Olsen , E. Slinde , G. L. Taranger , and O. Skaala . 2009. “A Comparison of Farmed, Wild and Hybrid Atlantic Salmon ( *Salmo salar* L.) Reared Under Farming Conditions.” Aquaculture 286: 203–210. 10.1016/j.aquaculture.2008.09.023.

[mec70036-bib-0024] Glover, K. A. , M. Quintela , V. Wennevik , F. Besnier , A. G. E. Sørvik , and Ø. Skaala . 2012. “Three Decades of Farmed Escapees in the Wild: A Spatio‐Temporal Analysis of Atlantic Salmon Population Genetic Structure Throughout Norway.” PLoS One 7: e43129. 10.1371/journal.pone.0043129. 22916215 PMC3419752

[mec70036-bib-0025] Glover, K. A. , M. F. Solberg , P. McGinnity , et al. 2017. “Half a Century of Genetic Interaction Between Farmed and Wild Atlantic Salmon: Status of Knowledge and Unanswered Questions.” Fish and Fisheries 2017: 1–38. 10.1111/faf.12214.

[mec70036-bib-0026] Glover, K. A. , V. Wennevik , K. Hindar , et al. 2020. “The Future Looks Like the Past: Introgression of Domesticated Atlantic Salmon Escapees in a Risk Assessment Framework.” Fish and Fisheries 21: 1077–1091. 10.1111/faf.12478.

[mec70036-bib-0027] Gompert, Z. , and C. A. Buerkle . 2009. “A Powerful Regression‐Based Method for Admixture Mapping of Isolation Across the Genome of Hybrids.” Molecular Ecology 18: 1207–1224. 10.1111/j.1365-294X.2009.04098.x. 19243513

[mec70036-bib-0028] Gompert, Z. , and C. A. Buerkle . 2010. “Introgress: A Software Package for Mapping Components of Isolation in Hybrids.” Molecular Ecology Resources 10: 378–384. 10.1111/j.1755-0998.2009.02733.x. 21565033

[mec70036-bib-0077] Gompert, Z. , L. K. Lucas , C. C. Nice , J. A. Fordyce , M. L. Forister , and C. A. Buerkle . 2012. “Genomic Regions With a History of Divergent Selection Affect Fitness of Hybrids Between Two Butterfly Species.” Evolution 66–67: 2167–2181. 10.1111/j.1558-5646.2012.01587.x 22759293

[mec70036-bib-0029] Gompert, Z. , L. K. Lucas , C. C. Nice , and C. A. Buerkle . 2013. “Genomic Divergence and the Genetic Architecture of Barriers to Gene Flow Between *Lycaeides idas* and *L. melissa* .” Evolution 67: 2498–2514. 10.1111/evo.12021. 24033163

[mec70036-bib-0030] Goudet, J. 2005. “Hierfstat, a Package for R to Compute and Test Hierarchical F‐Statistics.” Molecular Ecology Notes 5: 184–186. 10.1111/j.1471-8286.2004.00828.x.

[mec70036-bib-0032] Hansen, M. M. , D. J. Fraser , K. Meier , and K. L. D. Mensberg . 2009. “Sixty Years of Anthropogenic Pressure: A Spatio‐Temporal Genetic Analysis of Brown Trout Populations Subject to Stocking and Population Declines.” Molecular Ecology 18: 2549–2562. 10.1111/j.1365-294X.2009.04198.x. 19457206

[mec70036-bib-0033] Hansen, M. M. , K. L. Mensberg , G. Rasmussen , and V. Simonsen . 1997. “Genetic Variation Within and Among Danish Brown Trout (*Salmo trutta L*.) Hatchery Strains, Assessed by PCR‐RFLP Analysis of Mitochondrial DNA Segments.” Aquaculture 153: 15–29. 10.1016/S0044-8486(97)00022-7.

[mec70036-bib-0076] Hansen, M. M. , D. Bekkevold , L. F. Jensen , K.‐L. D. Mensberg , and E. Eg Nielsen . 2006. “Genetic Restoration of a Stocked Brown Trout *Salmo trutta* Population Using Microsatellite DNA Analysis of Historical and Contemporary Samples.” Journal of Applied Ecology 43, no. 4: 669–679.

[mec70036-bib-0034] Hansen, M. M. , and K. L. D. Mensberg . 2009. “Admixture Analysis of Stocked Brown Trout Populations Using Mapped Microsatellite DNA Markers: Indigenous Trout Persist in Introgressed Populations.” Biology Letters 5: 656–659. 10.1098/rsbl.2009.0214. 19515653 PMC2781948

[mec70036-bib-0035] Hansen, M. M. , I. Olivieri , D. M. Waller , E. E. Nielsen , and The Genetic Monitoring Working Group . 2012. “Monitoring Adaptive Genetic Responses to Environmental Change.” Molecular Ecology 21: 1311–1329. 10.1111/j.1365-294X.2011.05463.x. 22269082

[mec70036-bib-0036] Hansen, M. M. , D. E. Ruzzante , E. E. Nielsen , and K.‐L. D. Mensberg . 2001. “Microsatellite Polymorphism in Domesticated and Wild Brown Trout (*Salmo trutta*) and Stocking Impact Assessment.” Ecological Applications 11: 148–160. 10.2307/3061063.

[mec70036-bib-0037] Harbicht, A. , C. C. Wilson , and D. J. Fraser . 2014. “Does Human‐Induced Hybridization Have Long‐Term Genetic Effects? Empirical Testing With Domesticated, Wild and Hybridized Fish Populations.” Evolutionary Applications 7: 1180–1191. 10.1111/eva.12199. 25558279 PMC4275090

[mec70036-bib-0038] Harvey, A. C. , K. A. Glover , M. I. Taylor , S. Creer , and G. R. Carvalho . 2016. “A Common Garden Design Reveals Population‐Specific Variability in Potential Impacts of Hybridization Between Populations of Farmed and Wild Atlantic Salmon, *Salmo salar* L.” Evolutionary Applications 9: 435–449. 10.1111/eva.12346. 26989435 PMC4778114

[mec70036-bib-0039] Harvey, A. C. , O. T. Skilbrei , F. Besnier , M. F. Solberg , A.‐G. E. Sørvik , and K. A. Glover . 2018. “Implications for Introgression: Has Selection for Fast Growth Altered the Size Threshold for Precocious Male Maturation in Domesticated Atlantic Salmon?” BMC Evolutionary Biology 18: 188. 10.1186/s12862-018-1294-y. 30558529 PMC6298023

[mec70036-bib-0040] Hubert, J.‐N. , T. Zerjal , and F. Hospital . 2018. “Cancer‐ and Behavior‐Related Genes Are Targeted by Selection in the Tasmanian Devil ( *Sarcophilus harrisii* ).” PLoS One 13, no. 8: e0201838. 10.1371/journal.pone.0201838. 30102725 PMC6089428

[mec70036-bib-0041] Hwang, A. S. , V. L. Pritchard , and S. Edmands . 2016. “Recovery From Hybrid Breakdown in a Marine Invertebrate Is Faster, Stronger and More Repeatable Under Environmental Stress.” Journal of Evolutionary Biology 29: 1793–1803. 10.1111/jeb.12913. 27271820

[mec70036-bib-0042] Jombart, T. 2008. “Adegenet: A R Package for the Multivariate Analysis of Genetic Markers.” Bioinformatics 24: 1403–1405. 10.1093/bioinformatics/btn129. 18397895

[mec70036-bib-0043] LaCava, M. E. F. , J. S. Griffiths , L. Ellison , E. W. Carson , T. C. Hung , and A. J. Finger . 2023. “Loss of Plasticity in Maturation Timing After Ten Years of Captive Spawning in a Delta Smelt Conservation Hatchery.” Evolutionary Applications 16, no. 11: 1845–1857. 10.1111/eva.13611. 38029063 PMC10681455

[mec70036-bib-0044] Lamaze, F. C. , C. Sauvage , A. Marie , D. Garant , and L. Bernatchez . 2012. “Dynamics of Introgressive Hybridization Assessed by SNP Population Genomics of Coding Genes in Stocked Brook Charr ( *Salvelinus fontinalis* ).” Molecular Ecology 21: 2877–2895. 10.1111/j.1365-294X.2012.05579.x. 22548328

[mec70036-bib-0045] Leitwein, M. , H. Cayuela , and L. Bernatchez . 2021. “Associative Overdominance and Negative Epistasis Shape Genome‐Wide Ancestry Landscape in Supplemented Fish Populations.” Genes 12: 524. 10.3390/genes12040524. 33916757 PMC8065892

[mec70036-bib-0046] Lexer, C. , C. A. Buerkle , J. A. Joseph , B. Heinze , and M. F. Fay . 2007. “Admixture in European Populus Hybrid Zones Makes Feasible the Mapping of Loci That Contribute to Reproductive Isolation and Trait Differences.” Heredity 98: 74–84. 10.1038/sj.hdy.6800898. 16985509

[mec70036-bib-0047] Li, H. , and R. Durbin . 2009. “Fast and Accurate Short Read Alignment With Burrows–Wheeler Transform.” Bioinformatics 25: 1754–1760. 10.1093/bioinformatics/btp324. 19451168 PMC2705234

[mec70036-bib-0048] Lien, S. , B. F. Koop , S. R. Sandve , et al. 2016. “The Atlantic Salmon Genome Provides Insights Into Rediploidization.” Nature 533: 200–205. 10.1038/nature17164. 27088604 PMC8127823

[mec70036-bib-0049] Marie, A. D. , L. Bernatchez , and D. Garant . 2010. “Loss of Genetic Integrity Correlates With Stocking Intensity in Brook Charr ( *Salvelinus fontinalis* ).” Molecular Ecology 19: 2025–2037. 10.1111/j.1365-294X.2010.04628.x. 20406382

[mec70036-bib-0050] McGinnity, P. , P. Prodöhl , A. Ferguson , et al. 2003. “Fitness Reduction and Potential Extinction of Wild Populations of Atlantic Salmon *Salmo salar*, as a Result of Interactions With Escaped Farm Salmon.” Proceedings of the Royal Society of London Series B 270: 2443–2450. 10.1098/rspb.2003.2520. 14667333 PMC1691531

[mec70036-bib-0051] Moustakas‐Verho, E. , J. Kurko , A. H. House , J. Erkinaro , P. Debes , and C. R. Primmer . 2020. “Developmental Expression Patterns of six6: A Gene Linked With Spawning Ecotypes in Atlantic Salmon.” Gene Expression Patterns 38: 119149. 10.1016/j.gep.2020.119149. 33007443

[mec70036-bib-0052] Ozerov, M. Y. , R. Gross , M. Bruneaux , et al. 2016. “Genomewide Introgressive Hybridization Patterns in Wild Atlantic Salmon Influenced by Inadvertent Gene Flow From Hatchery Releases.” Molecular Ecology 25: 1275–1293. 10.1111/mec.13570. 26840557

[mec70036-bib-0053] Piper, A. T. , P. J. Rosewarne , D. Bekkevold , J. Grey , A. Nye , and R. M. Wright . 2025. “Migration Patterns, Habitat Use and Genetic Origins of Sea Trout ( *Salmo trutta* ) in Norfolk Chalk Streams: Implications for Management of a Mixed Stock Fishery.” Aquatic Science 87: 7. 10.1007/s00027-024-01135-1.

[mec70036-bib-0054] Pritchard, J. K. , M. Stephens , and P. Donnelly . 2000. “Inference of Population Structure Using Multilocus Genotype Data.” Genetics 155: 945–959. 10.1093/genetics/155.2.945. 10835412 PMC1461096

[mec70036-bib-0055] Pritchard, V. L. , H. Mäkinen , J. P. Vähä , J. Erkinaro , P. Orell , and C. R. Primmer . 2018. “Genomic Signatures of Fine‐Scale Local Selection in Atlantic Salmon Suggest Involvement of Sexual Maturation, Energy Homeostasis and Immune Defence‐Related Genes.” Molecular Ecology 27: 2560–2575. 10.1111/mec.14705. 29691916

[mec70036-bib-0056] Raymond, M. , and F. Rousset . 1995. “GENEPOP (Version 1.2): Population Genetics Software for Exact Tests and Ecumenicism.” Journal of Heredity 86: 248–249. 10.1093/oxfordjournals.jhered.a111573.

[mec70036-bib-0057] Reed, T. E. , P. Prodohl , R. Hynes , T. Cross , A. Ferguson , and P. McGinnity . 2015. “Quantifying Heritable Variation in Fitness‐Related Traits of Wild, Farmed and Hybrid Atlantic Salmon Families in a Wild River Environment.” Heredity 115: 173–184. 10.1038/hdy.2015.29. 25920670 PMC4815444

[mec70036-bib-0058] Rhymer, J. M. , and D. Simberloff . 1996. “Extinction by Hybridization and Introgression.” Annual Review of Ecology and Systematics 27: 83–109. 10.1146/annurev.ecolsys.27.1.83.

[mec70036-bib-0059] Rieseberg, L. H. , M. A. Archer , and R. K. Wayne . 1999. “Transgressive Segregation, Adaptation and Speciation.” Heredity 83: 363–372. 10.1038/sj.hdy.6886170. 10583537

[mec70036-bib-0060] Ryman, N. , and L. Laikre . 1991. “Effects of Supportive Breeding on Genetically Effective Population Size.” Conservation Biology 5: 325–329. https://www.jstor.org/stable/2385902.

[mec70036-bib-0061] Sinclair‐Waters, M. , J. Ødegård , S. A. Korsvoll , et al. 2020. “Beyond Large‐Effect Loci: Large‐Scale GWAS Reveals a Mixed Large‐Effect and Polygenic Architecture for Age at Maturity of Atlantic Salmon.” Genetics, Selection, Evolution 52: 9. 10.1186/s12711-020-0529-8. PMC7017552 32050893

[mec70036-bib-0062] Skaala, Ø. , F. Besnier , R. Borgstrøm , et al. 2019. “An Extensive Common‐Garden Study With Domesticated and Wild Atlantic Salmon in the Wild Reveals Impact on Smolt Production and Shifts in Fitness Traits.” Evolutionary Applications 12, no. 5: 1001–1016. 10.1111/eva.12777. 31080511 PMC6503829

[mec70036-bib-0063] Skaala, O. , K. A. Glover , B. T. Barlaup , et al. 2012. “Performance of Farmed, Hybrid, and Wild Atlantic Salmon ( *Salmo salar* ) Families in a Natural River Environment.” Canadian Journal of Fisheries and Aquatic Sciences 69: 1994–2006.

[mec70036-bib-0064] Slate, J. 2005. “Quantitative Trait Locus Mapping in Natural Populations: Progress, Caveats and Future Directions.” Molecular Ecology 14: 363–379. 10.1111/j.1365-294X.2004.02378.x. 15660931

[mec70036-bib-0065] Solberg, M. F. , O. Skaala , F. Nilsen , and K. A. Glover . 2013. “Does Domestication Cause Changes in Growth Reaction Norms? A Study of Farmed, Wild and Hybrid Atlantic Salmon Families Exposed to Environmental Stress.” PLoS One 8: e54469. 10.1371/journal.pone.0054469. 23382901 PMC3561353

[mec70036-bib-0066] Therkildsen, N. O. , A. P. Wilder , D. O. Conover , S. B. Munch , H. Baumann , and S. R. Palumbi . 2019. “Contrasting Genomic Shifts Underlie Parallel Phenotypic Evolution in Response to Fishing.” Science 365, no. 6452: 487–490. 10.1126/science.aaw7271. 31371613

[mec70036-bib-0067] Thorpe, J. 2004. “Life History Responses of Fishes to Culture.” Journal of Fish Biology 65: 263–285. 10.1111/j.0022-1112.2004.00556.x.

[mec70036-bib-0068] Todesco, M. , M. A. Pascual , G. L. Owens , et al. 2016. “Hybridization and Extinction.” Evolutionary Applications 9: 892–908. 10.1111/eva.12367. 27468307 PMC4947151

[mec70036-bib-0069] Tufto, J. 2017. “Domestication and Fitness in the Wild: A Multivariate View.” Evolution 71: 2262–2270. 10.1111/evo.13307. 28714571

[mec70036-bib-0070] Wacker, S. , T. Aronsen , S. Karlsson , et al. 2021. “Selection Against Individuals From Genetic Introgression of Escaped Farmed Salmon in a Natural Population of Atlantic Salmon.” Evolutionary Applications 14: 1450–1460. 10.1111/eva.13213. 34025778 PMC8127704

[mec70036-bib-0071] Walsh, J. , G. W. Shriver , B. J. Olsen , and A. I. Kovach . 2016. “Differential Introgression and the Maintenance of Species Boundaries in an Advanced Generation Avian Hybrid Zone.” BMC Evolutionary Biology 16: 65. 10.1186/s12862-016-0635-y. 27000833 PMC4802838

[mec70036-bib-0072] Webb, J. H. , D. W. Hay , P. D. Cunningham , and A. F. Youngson . 1991. “The Spawning Behaviour of Escaped Farmed and Wild Adult Atlantic Salmon (* Salmo salar L*.) in a Northern Scottish River.” Aquaculture 98: 97–110. 10.1016/0044-8486(91)90375-H.

[mec70036-bib-0073] Wringe, B. F. , F. Craig , and I. A. Fleming . 2016. “In Search of a “Cultured Fish Phenotype”: A Systematic Review, Meta‐Analysis and Vote‐Counting Analysis.” Reviews in Fish Biology and Fisheries 26: 351–373. 10.1007/s11160-016-9431-4.

[mec70036-bib-0074] Yang, L. , R. S. Waples , and M. L. Baskett . 2019. “Life History and Temporal Variability of Escape Events Interactively Determine the Fitness Consequences of Aquaculture Escapees on Wild Populations.” Theoretical Population Biology 129: 93–102. 10.1016/j.tpb.2018.12.006. 31028784

[mec70036-bib-0075] Zueva, K. J. , J. Lumme , A. E. Veselov , C. R. Primmer , and V. L. Pritchard . 2020. “Population Genomics Reveals Repeated Signals of Adaptive Divergence in the Atlantic Salmon of Northeastern Europe.” Journal of Evolutionary Biology 34: 866–878. 10.1111/jeb.13732. 33147360

