# Supplementary material for: Genetic Recovery of Two Wild Seatrout Populations Following Long‐Term Stocking With Non‐Native Conspecifics
Source: Mol Ecol. 2025 Jul 12;34(17):e70036. doi: 10.1111/mec.70036 (PMC12376961; doi:10.1111/mec.70036)
Supplement: Supplementary file 1 — Appendix S1. Table S1. List of SNP loci in analysis, with information about BLAST results, outlier and introgression results per locus. Table S2. Locus specific tests for HWE in analysed samples. Table S3. Structure delta K estimates in collected samples. Figure S1. DAPC plot of S. trutta samples typed for 3656 SNPs for K = 2. Figure S2. Structure plot of q estimates for collected samples. Figure S3. Structure plot of q estimates for simulated genotypes. Figure S4. Relationship between individual q estimates generated with 3656 and 288 loci. Figure S5. Allele frequencies for the SNP ‘Gdist:S499800_4765’ associated with the maturation gene six6 in temporal collections. [file MEC-34-e70036-s001.pdf]

MOLECULAR ECOLOGY

Supplemental Information for:

Genetic recovery of two wild seatrout populations following long-term stocking with non-native conspecifics

BEKKEVOLD, DORTE
GLOVER, KEVIN A.
JIMENEZ-MENA, BELÉN
BESNIER, FRANCOIS
NIELSEN, EINAR E.

Table of Contents:

Supplementary figure 1	Page 1
Supplementary figure 2	Page 2
Supplementary figure 3	Page 3
Supplementary figure 4	Page 4
Supplementary figure 5	Page 5
Supplementary table 1	Pages 7-35
Supplementary table 2	Pages 36-112
Supplementary table 3	Page 113

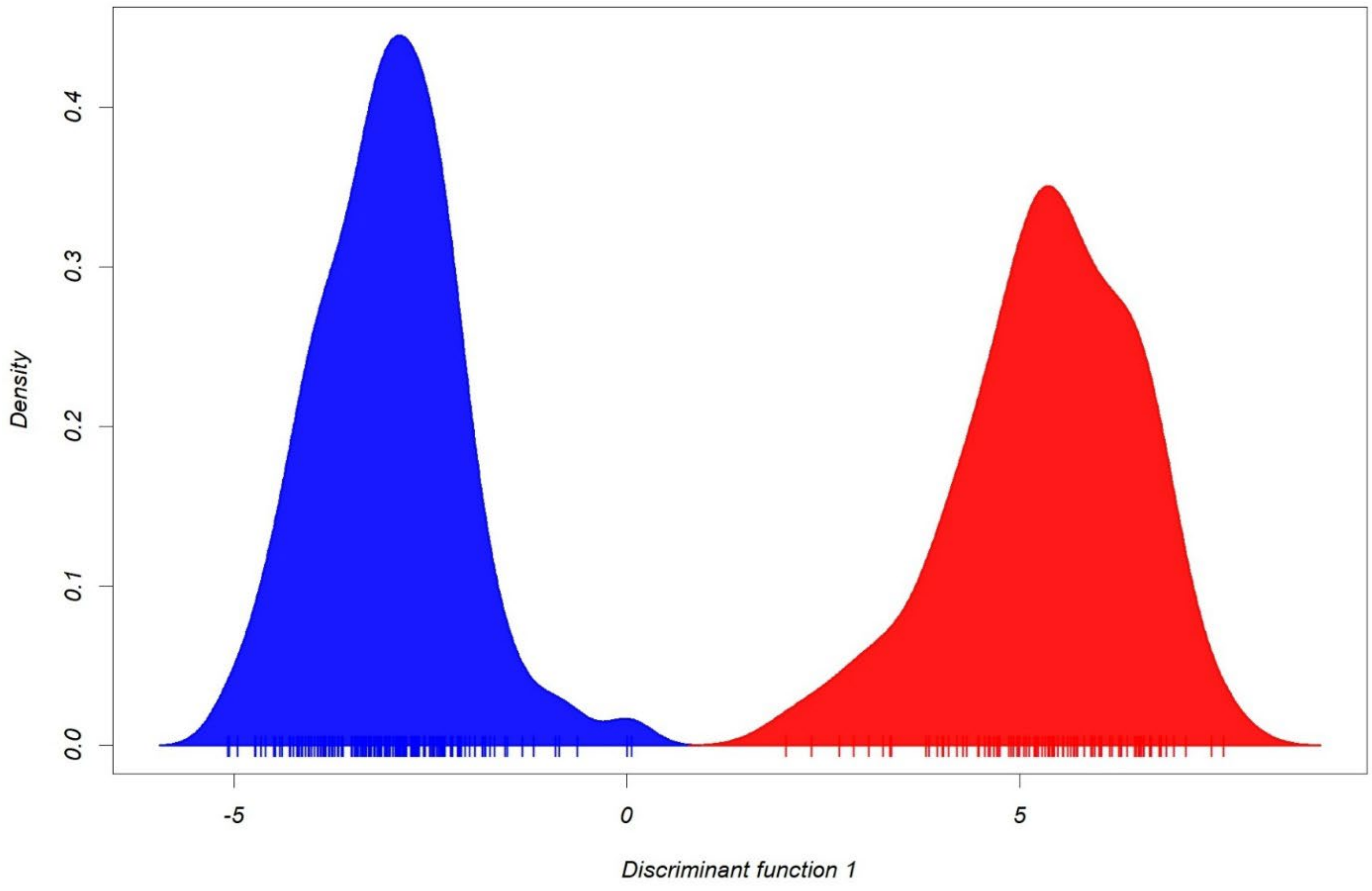

Supplementary Figure S1. DAPC plot of S. trutta samples typed for 3656 SNPs, showing individual fish principal component values for the PC1 (100% var. explained) as notches below and the density plotsabove, grouping all hatchery strain fish (shown in red) into one cluster and all fish with wild or wild-hatchery admixed genotypes into the other cluster (shown in blue).

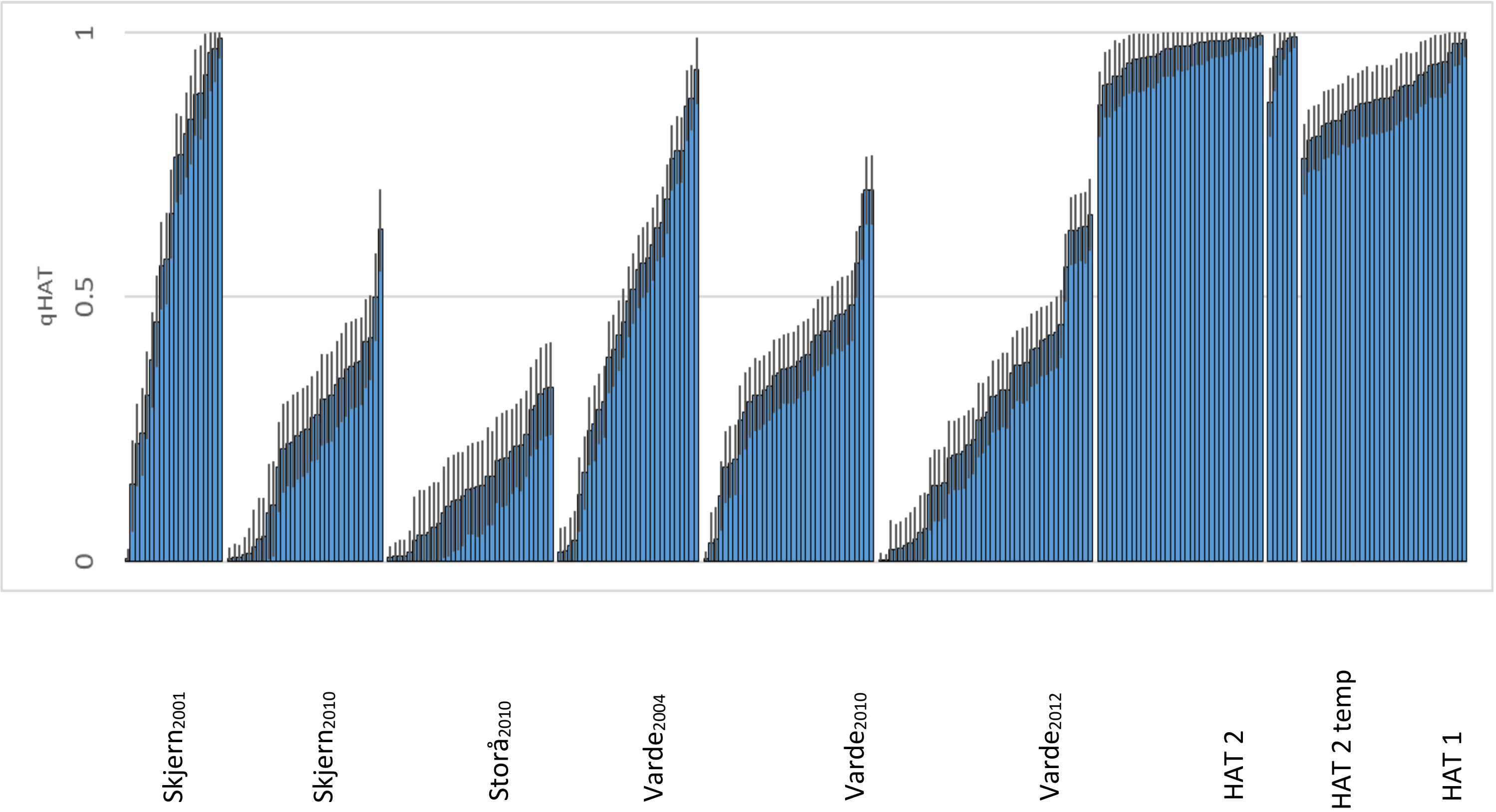

Supplementary Figure S2. Structure plots (q admixture proportion from HAT) for samples of wild caught and hatchery strain trout. Error bars show 90% confidence intervals.

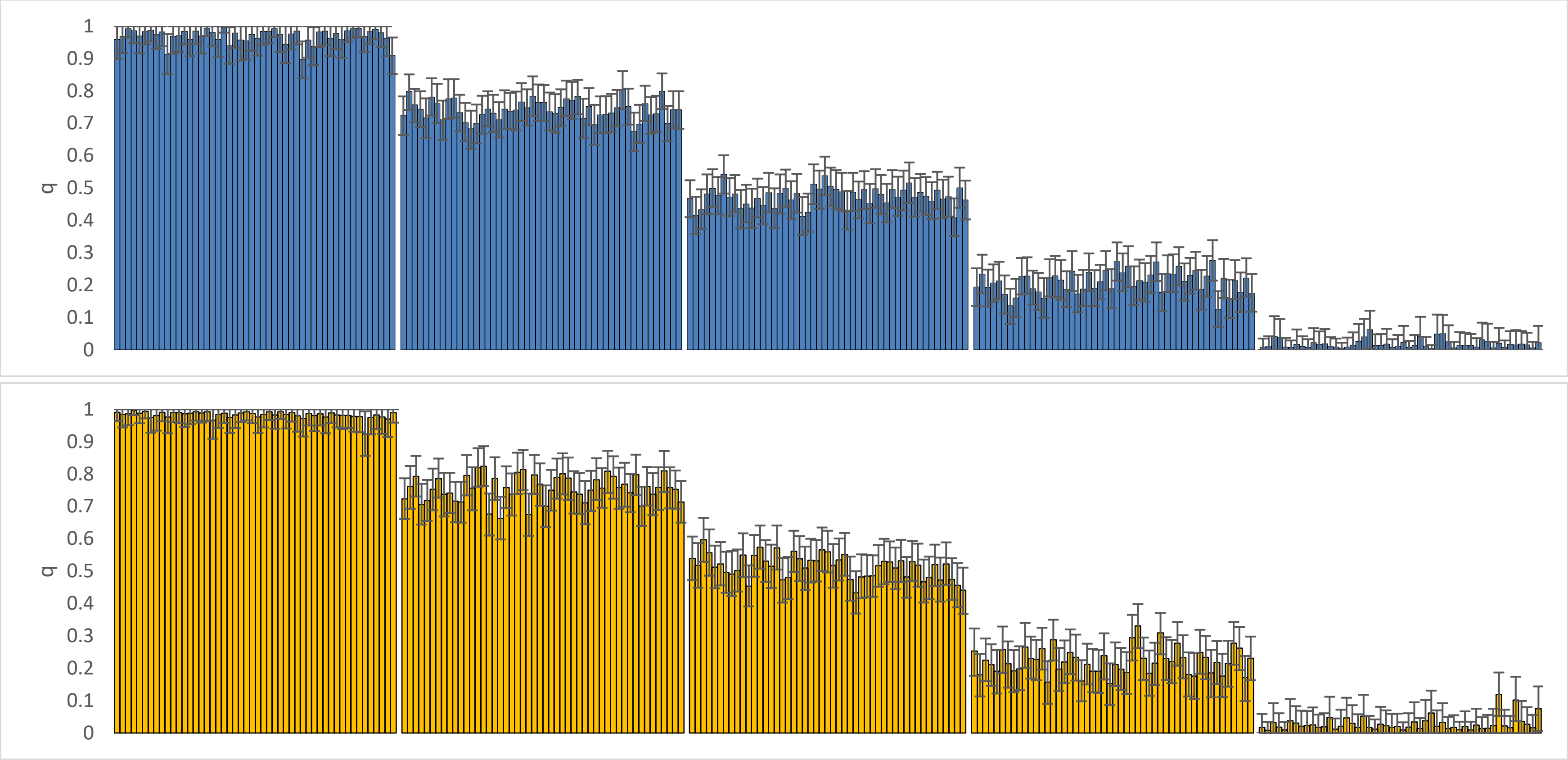

Supplementary Figure S3. *Structure* plots (q admixture proportion from HAT) for simulated genotypes representing, respectively, River Varde (blue bars), and River Skjern (yellow bars). Individual blocks from left to right represent, respectively, simulated HAT, BC_{HAT}, F1, BC_{WILD}, and Pure WILD genotypes. Error bars show 90% confidence intervals.

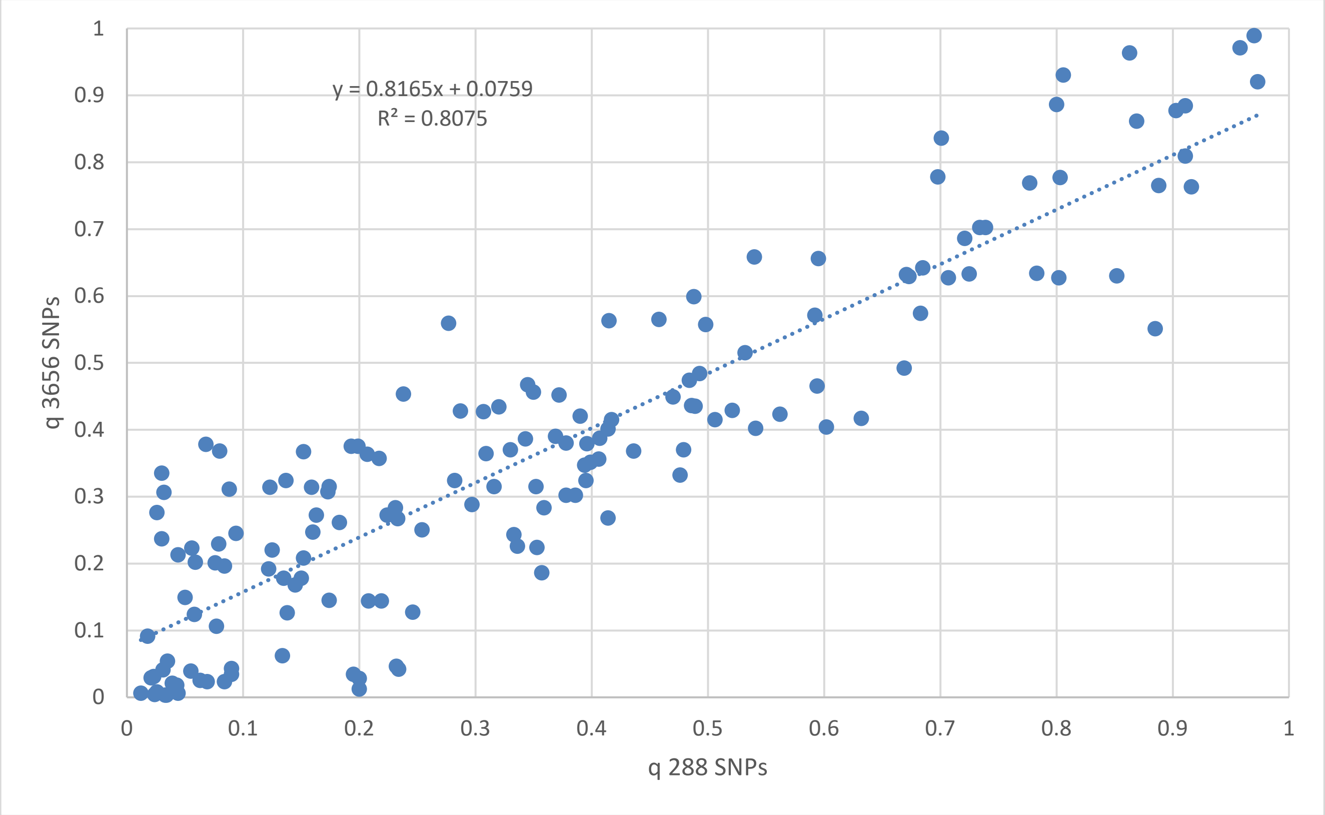

Supplementary Figure S4. Relationship between individual *Structure* q estimates (admixture proportion from HAT) estimated with respectively 288 and 3656 SNPs.

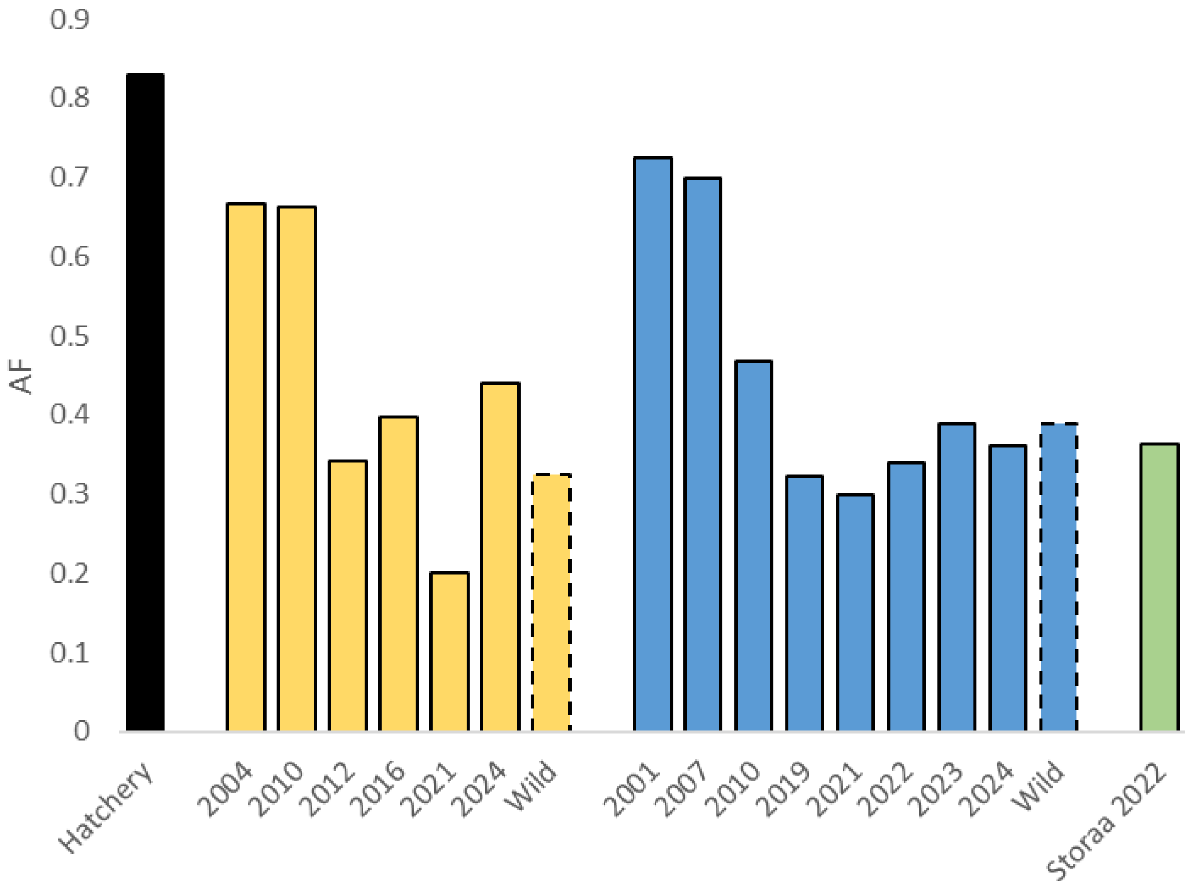

Supplementary figure S5. Allele frequencies (AF) for the SNP ‘Gdist:S499800_4765’ associated with the maturation candidate gene *six6* in temporal samples from 2001-2024. AF for the hatchery strain (HAT) is shown in black, AF for river Varde in yellow, AF for river Skjern in blue, and AF for a contemporary sample from the latter’s neighbouring river Storaa, which has shown low genetic impact of HAT stocking, in green. AF in individuals inferred as having non-admixed (Wild) ancestry, based on structure clustering analysis of 3656 SNPs, are shown by bars with stippled edges.

Locus ID (from Bekkevold et al. 2020)	LG (1-40) in Bekkevold et al. (2020)	Female_map _position	Global Fst	BLAST	Locus also on 288 panel?	VAR outlier bayescan	SKJ outlier Bayescan	VAR outlier signasel Ne=200 (P-value following FDR)	VAR outlier signasel Ne=100 (P-value following FDR)	SKJ outlier signasel Ne=200 (P-value following FDR)	SKJ outlier signasel Ne=100 (P-value following FDR)	INTROGRESS VARDE P value	INTROGRESS SKJERN P value	LOCUS in genomic cline analysis	Type of locus behaviour in genomic cline analysis	Varde behaviour in genomic cline analysis	Skjern behaviour in genomic cline analysis	Locus showing GEA in Bekkevold et al. (2020)
Gdist:S372553_4889	BT01	0	-0.0059	NA	No	No	No	No	No	No	No	NA	NA	No	NA	NA	NA	NA
Gdist:S41629_4955	BT01	0.003	0.0365	NA	No	No	No	No	No	No	No	0.530792308	0.1434375	Yes	NA	NA	NA	NA
Gdist:S209528_9132	BT01	0.003	0.031	NA	Yes	No	No	No	No	No	No	0.682433921	0.91619469	Yes	NA	NA	NA	NA
cDNA:S177852_1371	BT01	0.003	0.011	NA	No	No	No	No	No	No	No	NA	NA	No	NA	NA	NA	NA
cDNA:S315619_2265	BT01	0.003	-0.0163	NA	No	No	No	No	No	No	No	NA	NA	No	NA	NA	NA	NA
SalHit:S341987_3098	BT01	0.012	0.0581	NA	No	No	No	No	No	Yes (0.015)	No	0.407177419	0.66132237	Yes	NA	NA	NA	NA
Gdist:S632715_2055	BT01	0.012	0.03	NA	No	No	No	No	No	No	No	0.532767857	0.89499248	Yes	NA	NA	NA	NA
Gdist:S189002_716	BT01	0.012	0.028	NA	No	No	No	No	No	No	No	NA	NA	No	NA	NA	NA	NA
Gdist:S9435_3306	BT01	0.012	0.0088	NA	No	No	No	No	No	Yes (0.023)	No	NA	NA	No	NA	NA	NA	NA
Gdist:S246913_5166	BT01	0.012	0.0079	NA	No	No	No	No	No	No	No	NA	NA	No	NA	NA	NA	NA
Gdist:S1367_5121	BT01	0.012	0.0074	NA	No	No	No	No	No	No	No	NA	NA	No	NA	NA	NA	NA
LD:S88985_1626	BT01	0.39	0.0808	NA	No	No	No	No	No	No	No	0.982583449	0.28977273	Yes	NA	NA	NA	NA
Gdist:S441366_7652	BT01	0.39	0.0446	NA	No	No	No	No	No	Yes (0.043)	No	0.94260917	0.3762973	Yes	NA	NA	NA	NA
Gdist:S122610_1283	BT01	0.39	0.0389	NA	No	No	No	No	No	Yes (0.015)	No	0.200482759	0.32182759	Yes	NA	NA	NA	NA
LD:S88985_14448	BT01	0.39	0.0067	NA	No	No	No	No	No	No	No	NA	NA	No	NA	NA	NA	NA
SalHit:S98813_6560	BT01	0.39	0.0053	NA	Yes	No	No	No	No	No	No	NA	NA	No	NA	NA	NA	NA
Gdist:S266606_11520	BT01	0.476	0.0445	NA	No	No	No	No	No	Yes (0.012)	No	0.254299451	0.79436364	Yes	NA	NA	NA	NA
Gdist:S62415_1049	BT01	0.559	0.0488	NA	No	No	No	No	No	No	No	0.817611524	0.27708661	Yes	NA	NA	NA	NA
Gdist:S368648_4705	BT01	0.625	0.0001	NA	No	No	No	No	No	No	No	NA	NA	No	NA	NA	NA	NA
Gdist:S607923_3461	BT01	0.691	0.0168	NA	No	No	No	No	No	No	No	NA	NA	No	NA	NA	NA	NA
Gdist:S551108_5155	BT01	0.767	0.0078	NA	No	No	No	No	No	No	No	NA	NA	No	NA	NA	NA	NA
cDNA:S89695_5963	BT01	0.958	0.0125	NA	No	No	No	No	No	No	No	NA	NA	No	NA	NA	NA	NA
SalHit:S762083_2146	BT01	1.053	0.0062	NA	No	No	No	No	No	No	No	NA	NA	No	NA	NA	NA	NA
Gdist:S121444_1180	BT01	1.145	0.046	NA	No	No	No	No	No	No	No	0.375961538	0.16207627	Yes	NA	NA	NA	NA
SalHit:S234924_3667	BT01	1.145	0.0347	NA	No	No	No	No	No	No	No	0.565759053	0.37619505	Yes	NA	NA	NA	NA
SalHit:S263117_5869	BT01	1.146	0.0219	NA	No	No	No	No	No	No	No	NA	NA	No	NA	NA	NA	NA
cDNA:S164228_5818	BT01	2.175	0.0067	NA	No	No	No	No	No	Yes (0.023)	No	NA	NA	No	NA	NA	NA	NA
Gdist:S239787_6673	BT01	3.204	-0.003	NA	No	No	No	No	No	No	No	NA	NA	No	NA	NA	NA	NA
LD:S229656_12348	BT01	4.187	0.0161	NA	No	No	No	No	No	No	No	NA	NA	No	NA	NA	NA	NA
cDNA:S40801_3776	BT01	4.204	0.0325	NA	No	No	No	No	No	No	No	0.453596654	0.21798387	Yes	NA	NA	NA	NA
Gdist:S189901_5111	BT01	4.204	0.017	NA	Yes	No	No	No	No	No	No	NA	NA	No	NA	NA	NA	NA
LD:S229656_3723	BT01	4.204	0.002	NA	No	No	No	No	No	No	No	NA	NA	No	NA	NA	NA	NA
LD:S271068_3399	BT01	4.535	0.0482	NA	No	No	No	No	No	Yes (0.023)	No	0.9298125	0.1275	Yes	NA	NA	NA	NA
LD:S271068_14525	BT01	4.88	0.0358	NA	No	No	No	No	No	No	No	0.565714286	0.34518293	Yes	NA	NA	NA	NA
Gdist:S142531_1964	BT01	5.215	0.0735	NA	No	No	No	No	No	No	No	0.893131188	0.2136039	Yes	NA	NA	NA	NA
Gdist:S50605_5056	BT01	5.215	0.0253	NA	No	No	No	No	No	No	No	NA	NA	No	NA	NA	NA	NA
Gdist:S41088_4361	BT01	5.215	0.012	NA	No	No	No	No	No	No	No	NA	NA	No	NA	NA	NA	NA
SalHit:S282118_3930	BT01	5.215	-0.0139	NA	No	No	No	No	No	No	No	NA	NA	No	NA	NA	NA	NA
cDNA:S543515_1408	BT01	5.719	0.0503	NA	No	No	No	No	No	Yes (0.004)	Yes (0.035)	0.7171875	0.94814619	Yes	NA	NA	NA	NA
Gdist:S124475_1258	BT01	5.719	0.022	NA	No	No	No	No	No	Yes (0.012)	No	NA	NA	No	NA	NA	NA	NA
cDNA:S543515_1473	BT01	5.719	0.0076	NA	No	No	No	No	No	No	No	NA	NA	No	NA	NA	NA	NA
Gdist:S124475_421	BT01	5.719	0.0062	NA	No	No	No	No	No	No	No	NA	NA	No	NA	NA	NA	NA
SalHit:S258364_2087	BT01	5.719	0.0061	NA	No	No	No	No	No	No	No	NA	NA	No	NA	NA	NA	NA
Gdist:S196082_3762	BT01	5.976	0.0222	NA	No	No	No	No	No	No	No	NA	NA	No	NA	NA	NA	NA
Gdist:S360207_16392	BT01	6.096	0.0941	NA	No	No	No	No	No	No	No	0.0765	0.12170455	Yes	NA	NA	NA	NA
Gdist:S37327_1351	BT01	6.096	0.089	NA	No	No	No	No	No	No	No	0.735576923	0.89481818	Yes	NA	NA	NA	NA
Gdist:S547731_7184	BT01	6.096	0.067	NA	No	No	No	No	No	Yes (0.006)	Yes (0.042)	0.614833333	0.46026971	Yes	NA	NA	NA	NA
Gdist:S547731_3738	BT01	6.096	0.0458	NA	No	No	No	No	No	No	No	0.709894068	0.3183042	Yes	NA	NA	NA	NA
Gdist:S360207_16184	BT01	6.096	0.0056	NA	No	No	No	No	No	No	No	NA	NA	No	NA	NA	NA	NA
Gdist:S129778_1028	BT01	6.096	-0.0017	NA	No	No	No	No	No	Yes (0.035)	No	NA	NA	No	NA	NA	NA	NA
Gdist:S148298_4701	BT01	6.096	-0.0032	NA	No	No	No	No	No	No	No	NA	NA	No	NA	NA	NA	NA
Gdist:S201164_7496	BT01	6.842	0.0182	NA	No	No	No	No	No	No	No	NA	NA	No	NA	NA	NA	NA
Gdist:S225359_2787	BT01	6.847	0.0482	NA	No	No	No	No	No	Yes (0.009)	No	0.574279778	0.87956522	Yes	NA	NA	NA	NA
Gdist:S104594_982	BT01	6.853	0.0262	NA	No	No	No	No	No	Yes (0.044)	No	NA	NA	No	NA	NA	NA	NA
Gdist:S127569_9615	BT01	6.854	0.0203	NA	Yes	No	No	No	No	No	No	NA	NA	No	NA	NA	NA	NA
Gdist:S56520_5345	BT01	8.703	-0.0016	NA	No	No	No	No	No	No	No	NA	NA	No	NA	NA	NA	NA
cDNA:S357292_1178	BT01	8.751	0.0427	NA	No	No	No	No	No	Yes (0.007)	Yes (0.049)	0.953502155	0.26836694	Yes	NA	NA	NA	NA
Gdist:S27733_7194	BT01	8.751	0.0421	NA	No	No	No	No	No	No	No	0.508712121	0.88381141	Yes	NA	NA	NA	NA
cDNA:S92682_5328	BT01	8.751	0.0335	NA	No	No	No	No	No	No	No	0.709541578	0.9985	Yes	NA	NA	NA	NA
Gdist:S7145_5306	BT01	8.751	0.0086	NA	No	No	No	No	No	No	No	NA	NA	No	NA	NA	NA	NA
Gdist:S237556_9992	BT01	8.751	0.0063	NA	No	No	No	No	No	No	No	NA	NA	No	NA	NA	NA	NA
Gdist:S194915_5990	BT01	10.513	0.0461	NA	No	No	No	No	No	No	No	0.947958815	0.37626359	Yes	NA	NA	NA	NA
cDNA:S260469_1182	BT01	10.513	0.0249	NA	No	No	No	No	No	Yes (0.044)	No	NA	NA	No	NA	NA	NA	NA
Gdist:S398761_3978	BT01	13.387	-0.0053	NA	No	No	No	No	No	No	No	NA	NA	No	NA	NA	NA	NA
Gdist:S121446_4034	BT01	16.26	-0.0053	NA	No	No	No	No	No	No	No	NA	NA	No	NA	NA	NA	NA
cDNA:S104446_3884	BT01	16.385	-0.0107	NA	No	No	No	No	No	No	No	NA	NA	No	NA	NA	NA	NA
cDNA:S711523_1175	BT01	16.826	0.0378	NA	No	No	No	No	No	Yes (0)	Yes (0.003)	0.906372	0.374	Yes	NA	NA	NA	NA
SalHit:S389388_6314	BT01	20.781	-0.0054	NA	No	No	No	No	No	No	No	NA	NA	No	NA	NA	NA	NA
cDNA:S7674_5559	BT01	21.116	0.0011	NA	No	No	No	No	No	No	No	NA	NA	No	NA	NA	NA	NA
Gdist:S682849_7501	BT01	21.117	0.0134	NA	No	No	No	No	No	Yes (0.02)	No	NA	NA	No	NA	NA	NA	NA
Gdist:S30231_7788	BT01	21.117	-0.001	NA	No	No	No	No	No	No	No	NA	NA	No	NA	NA	NA	NA
Gdist:S64145_603	BT01	21.117	-0.007	NA	No	No	No	No	No	No	No	NA	NA	No	NA	NA	NA	NA
cDNA:S248003_6551	BT01	24.722	0.0281	NA	No	No	No	No	No	No	No	NA	NA	No	NA	NA	NA	NA
SalHit:S68852_10003	BT01	24.722	0.0037	NA	No	No	No	No	No	No	No	NA	NA	No	NA	NA	NA	NA
cDNA:S37782_4794	BT01	24.722	-0.003	NA	No	No	No	No	No	No	No	NA	NA	No	NA	NA	NA	NA
cDNA:S1382_3653	BT01	25.632	0.0262	NA	No	No	No	No	No	No	No	NA	NA	No	NA	NA	NA	NA
Gdist:S185352_7387	BT01	36.279	0.0131	NA	No	No	No	No	No	No	No	NA	NA	No	NA	NA	NA	NA
Gdist:S167399_7696	BT01	39.953	0.0057	NA	No	No	No	No	No	No	No	NA	NA	No	NA	NA	NA	NA
Gdist:S37853_4158	BT01	41.903	0.0135	NA	No	No	No	No	No	No	No	NA	NA	No	NA	NA	NA	NA
cDNA:S676635_1323	BT01	43.486	0.0112	NA	No	No	No	No	No	No	No	NA	NA	No	NA	NA	NA	NA
cDNA:S415600_773	BT01	45.115	0.026	NA	No	No	No	No	No	Yes (0.02)	No	NA	NA	No	NA	NA	NA	NA
cDNA:S415600_5145	BT01	45.77	0.0312	NA	No	No	No	No	No	No	No	0.238242857	0.7344	Yes	NA	NA	NA	NA
SalHit:S217001_1739	BT01	45.774	0.0386	NA	Yes	No	No	No	No	No	No	0.202959184	0.77613306	Yes	NA	NA	NA	NA
SalHit:S797477_1052	BT01	45.774	0.0177	NA	No	No	No	No	No	No	No	NA	NA	No	NA	NA	NA	NA
SalHit:S797477_1574	BT01	45.774	0.0174	NA	No	No	No	No	No	No	No	NA	NA	No	NA	NA	NA	NA
cDNA:S459129_1735	BT02	0	0.0354	NA	No	No	No	No	No	No	No	0.446736641	0.50813869	Yes	NA	NA	NA	NA
cDNA:S304515_4041	BT02	0.935	0.0236	NA	No	No	No	No	No	Yes (0.001)	Yes (0.016)	NA	NA	No	NA	NA	NA	NA
cDNA:S823443_822	BT02	1.221	-0.0131	NA	No	No	No	No	No	No	No	NA	NA	No	NA	NA	NA	NA
Gdist:S374679_7478	BT02	2.305	0.0099	NA	No	No	No	No	No	No	No	NA	NA	No	NA	NA	NA	NA
Gdist:S530345_735	BT02	2.309	0.0659	NA	No	No	No	No	No	Yes (0)	Yes (0.005)	0.93810219	0.71946429	Yes	NA	NA	NA	NA
Gdist:S519320_2282	BT02	2.309	0.0076	NA	No	No	No	No	No	No	No	NA	NA	No	NA	NA	NA	NA
SalHit:S117299_8462	BT02	2.465	-0.011	NA	No	No	No	No	No	No	No	NA	NA	No	NA	NA	NA	NA
SalarSNP:ESTNV_13128_133	BT02	2.467	-0.0041	NA	No	No	No	No	No	Yes (0.038)	No	NA	NA	No	NA	NA	NA	NA
SalHit:S736134_5529	BT02	3.006	0.0312	NA	Yes	No	No	No	No	No	No	0.633486683	0.84566784	Yes	NA	NA	NA	NA
SalHit:S736134_7346	BT02	3.242	0.0373	NA	Yes	No	No	No	No	No	No	0.614833333	0.98876506	Yes	NA	NA	NA	NA
cDNA:S611416_1700	BT02	3.242	0.0303	NA	No	No	No	No	No	No	No	0.522028302	0.81575481	Yes	NA	NA	NA	NA
cDNA:S611416_2436	BT02	3.253	0.0165	NA	Yes	No	No	No	No	No	No	NA	NA	No	NA	NA	NA	NA
Gdist:S8065_3229	BT02	4.716	0.0275	NA	No	No	No	No	No	Yes (0.037)	No	NA	NA	No	NA	NA	NA	NA
Gdist:S176224_5944	BT02	5.233	0.0201	NA	No	No	No	No	No	No	No	NA	NA	No	NA	NA	NA	NA
Gdist:S112613_8383	BT02	5.233	0.0054	NA	No	No	No	No	No	No	No	NA	NA	No	NA	NA	NA	NA
Gdist:S357104_9953	BT02	5.603	0.0104	NA	No	No	No	No	No	No	No	NA	NA	No	NA	NA	NA	NA
cDNA:S557906_1632	BT02	5.962	0.0327	NA	No	No	No	No	No	No	No	0.202959184	0.22871134	Yes	NA	NA	NA	NA
cDNA:S282882_3216	BT02	6.105	0.0465	NA	No	No	No	No	No	Yes (0.007)	No	0.916490895	0.33707813	Yes	NA	NA	NA	NA
Gdist:S620076_398	BT02	6.25	0.0013	NA	No	No	No	No	No	No	No	NA	NA	No	NA	NA	NA	NA
Gdist:S343_3623	BT02	6.409	0.0501	NA	No	No	No	No	No	Yes (0.023)	No	0.911143868	0.21798387	Yes	NA	NA	NA	NA
Gdist:S343768_3941	BT02	6.567	-0.0083	NA	No	No	No	No	No	No	No	NA	NA	No	NA	NA	NA	NA
Gdist:S9002_10672	BT02	7.729	0.0112	NA	No	No	No	No	No	No	No	NA	NA	No	NA	NA	NA	NA
Gdist:S233650_5483	BT02	7.731	-0.0118	NA	No	No	No	No	No	No	No	NA	NA	No	NA	NA	NA	NA
Gdist:S282568_420	BT02	8.245	0.0241	NA	No	No	No	No	No	Yes (0.014)	No	NA	NA	No	NA	NA	NA	NA
Gdist:S39020_3067	BT02	8.772	0.0538	NA	No	No	No	No	No	No	No	0.380912863	0.54853698	Yes	NA	NA	NA	NA
Gdist:S72498_4996	BT02	8.88	-0.0039	NA	No	No	No	No	No	No	No	NA	NA	No	NA	NA	NA	NA
Gdist:S827396_5437	BT02	9.13	0.043	NA	No	No	No	No	No	Yes (0.029)	No	0.705336538	0.50907273	Yes	NA	NA	NA	NA
Gdist:S827396_1282	BT02	9.268	0.0076	NA	No	No	No	No	No	No	No	NA	NA	No	NA	NA	NA	NA
Gdist:S808919_7870	BT02	9.408	-0.0043	NA	No	No	No	No	No	No	No	NA	NA	No	NA	NA	NA	NA
Gdist:S43041_9676	BT02	9.422	-0.0016	NA	No	No	No	No	No	No	No	NA	NA	No	NA	NA	NA	NA
SalHit:S838387_395	BT02	9.489	0.058	NA	No	Marginally sig	No	No	No	No	No	0.599485488	0.25943478	Yes	NA	NA	NA	NA
cDNA:S328188_1390	BT02	9.489	0.0416	NA	No	No	No	No	No	No	No	0.380912863	0.23816038	Yes	NA	NA	NA	NA

SalHit:S838387_712	BT02	9.715	0.0075	NA	No	No	No	No	No	No	No	NA	NA	No	NA	NA	NA	NA
Gene:S115043_649	BT02	10.702	0.0731	NA	No	No	No	No	No	Yes (0.02)	No	0.6875	0.3825	Yes	NA	NA	NA	NA
cDNA:S236680_5590	BT02	11.324	0.0032	NA	No	No	No	No	No	Yes (0.007)	Yes (0.049)	NA	NA	No	NA	NA	NA	NA
Gdist:S137268_1456	BT02	11.913	0.0101	NA	No	No	No	No	No	Yes (0.001)	Yes (0.012)	NA	NA	No	NA	NA	NA	NA
Gdist:S428107_6908	BT02	17.918	0.0325	NA	No	No	No	No	No	Yes (0)	Yes (0.004)	0.0095625	0.86111538	Yes	VAR INC	increased	NA	NA
Gdist:S50172_4002	BT02	17.93	0.0178	NA	No	No	No	No	No	No	No	NA	NA	No	NA	NA	NA	NA
Gdist:S316844_1627	BT02	20.304	0.0393	NA	No	No	No	No	No	No	No	0.329216418	0.3565678	Yes	NA	NA	NA	NA
cDNA:S53142_3762	BT02	20.439	0.0128	NA	No	No	No	No	No	No	No	NA	NA	No	NA	NA	NA	NA
Gdist:S85941_9281	BT02	21.29	-0.0059	NA	No	No	No	No	No	No	No	NA	NA	No	NA	NA	NA	NA
Gdist:S154926_15184	BT02	22.141	0.0226	NA	No	No	No	No	No	No	No	NA	NA	No	NA	NA	NA	NA
Gdist:S154926_11931	BT02	22.96	0.0514	NA	No	No	No	No	No	No	No	0.419853516	0.76824153	Yes	NA	NA	NA	NA
Gdist:S96636_9765	BT02	22.96	0.0315	NA	No	No	No	No	No	No	No	0.7171875	0.96861417	Yes	NA	NA	NA	NA
Gdist:S154926_5381	BT02	22.96	0.0262	NA	No	No	No	No	No	No	No	NA	NA	No	NA	NA	NA	NA
Gdist:S96636_14046	BT02	22.96	0.0225	NA	No	No	No	No	No	No	No	NA	NA	No	NA	NA	NA	NA
cDNA:S128101_345	BT02	23.213	0.0225	NA	No	No	No	No	No	No	No	NA	NA	No	NA	NA	NA	NA
Gdist:S407139_1408	BT02	23.466	0.0083	NA	No	No	No	No	No	No	No	NA	NA	No	NA	NA	NA	NA
Gdist:S30852_5724	BT02	23.775	0.0305	NA	Yes	No	No	No	No	No	No	0.893131188	0.93621951	Yes	NA	NA	NA	NA
SalHit:S584677_840	BT02	28.666	-0.0074	NA	No	No	No	No	No	No	No	NA	NA	No	NA	NA	NA	NA
cDNA:S176944_17346	BT02	28.678	0.0031	NA	No	No	No	No	No	No	No	NA	NA	No	NA	NA	NA	NA
Gdist:S66714_2881	BT02	28.681	0.0182	NA	No	No	No	No	No	Yes (0.035)	No	NA	NA	No	NA	NA	NA	NA
LD:S166532_9926	BT02	28.681	0.009	NA	No	No	No	No	No	No	No	NA	NA	No	NA	NA	NA	NA
Gdist:S471403_6714	BT02	28.681	-0.0001	NA	No	No	No	No	No	No	No	NA	NA	No	NA	NA	NA	NA
LD:S166532_1813	BT02	28.681	-0.0003	NA	No	No	No	No	No	No	No	NA	NA	No	NA	NA	NA	NA
Gdist:S471403_1321	BT02	28.682	0.0274	NA	No	No	No	No	No	No	No	NA	NA	No	NA	NA	NA	NA
Gdist:S660617_5385	BT02	28.841	0.0146	NA	No	No	No	No	No	No	No	NA	NA	No	NA	NA	NA	NA
Gdist:S87651_2888	BT02	31.337	0.0049	NA	No	No	No	No	No	No	No	NA	NA	No	NA	NA	NA	NA
LD:S96507_858	BT02	31.337	0.0039	NA	No	No	No	No	No	No	No	NA	NA	No	NA	NA	NA	NA
LD:S96507_10894	BT02	31.337	-0.0046	NA	No	No	No	No	No	No	No	NA	NA	No	NA	NA	NA	NA
SalHit:S91955_5816	BT02	31.928	0.0067	NA	Yes	No	No	No	No	No	No	NA	NA	No	NA	NA	NA	NA
Gdist:S444476_6666	BT02	32.52	0.0184	NA	No	No	No	No	No	No	No	NA	NA	No	NA	NA	NA	NA
LD:S144765_9154	BT02	33.181	0.0045	NA	No	No	No	No	No	No	No	NA	NA	No	NA	NA	NA	NA
Gdist:S565001_5794	BT02	37.777	0.0266	NA	No	No	No	No	No	No	No	NA	NA	No	NA	NA	NA	NA
SalHit:S140659_7566	BT02	39.162	-0.0079	NA	No	No	No	No	No	No	No	NA	NA	No	NA	NA	NA	NA
SalHit:S317662_1985	BT02	40.528	0.0195	NA	No	No	No	No	No	No	No	NA	NA	No	NA	NA	NA	NA
Gdist:S127792_6520	BT02	40.539	0.0257	NA	No	No	No	No	No	No	No	NA	NA	No	NA	NA	NA	NA
cDNA:S607119_856	BT02	40.539	-0.0037	NA	No	No	No	No	No	No	No	NA	NA	No	NA	NA	NA	NA
Gdist:S575992_4710	BT02	41.362	0.0398	NA	No	No	No	No	No	No	No	0.14025	0.71368902	Yes	NA	NA	NA	NA
Gdist:S225079_5366	BT02	41.714	0.03	NA	No	No	No	No	No	No	No	NA	NA	No	NA	NA	NA	NA
Gdist:S150041_834	BT02	42.027	0.0511	NA	No	No	No	No	No	No	No	0.115471698	0.23395631	Yes	NA	NA	NA	NA
Gdist:S150041_7238	BT02	42.027	-0.0033	NA	No	No	No	No	No	No	No	NA	NA	No	NA	NA	NA	NA
Gdist:S112883_6809	BT02	44.247	0.0232	NA	No	No	No	No	No	No	No	NA	NA	No	NA	NA	NA	NA
Gdist:S395870_6048	BT02	44.247	-0.0019	NA	No	No	No	No	No	No	No	NA	NA	No	NA	NA	NA	NA
cDNA:S642705_1575	BT02	44.251	0.0156	NA	No	No	No	No	No	No	No	NA	NA	No	NA	NA	NA	NA
cDNA:C124754657_212	BT02	44.255	0.0084	NA	No	No	No	No	No	No	No	NA	NA	No	NA	NA	NA	NA
cDNA:S159289_3695	BT02	44.846	0.0957	NA	No	No	No	No	No	Yes (0.014)	No	0.17983209	0.42987385	Yes	NA	NA	NA	NA
cDNA:S767508_719	BT02	45.43	0.0114	NA	No	No	No	No	No	No	No	NA	NA	No	NA	NA	NA	NA
SalHit:S37375_7111	BT02	45.431	0.0443	NA	No	No	No	No	No	No	No	0.982691275	0.6958849	Yes	NA	NA	NA	NA
cDNA:S258718_3516	BT02	45.45	-0.0042	NA	No	No	No	No	No	No	No	NA	NA	No	NA	NA	NA	NA
cDNA:S258718_7888	BT02	45.47	0.0125	NA	No	No	No	No	No	No	No	NA	NA	No	NA	NA	NA	NA
cDNA:S322930_3152	BT02	46.812	0.0182	NA	No	No	No	No	No	No	No	NA	NA	No	NA	NA	NA	NA
Gdist:S87840_7742	BT02	46.826	-0.0053	NA	No	No	No	No	No	Yes (0.035)	No	NA	NA	No	NA	NA	NA	NA
cDNA:S471822_9611	BT02	46.854	0.0446	NA	No	No	No	No	No	No	No	0.660278422	0.69352273	Yes	NA	NA	NA	NA
Gdist:S223968_2011	BT02	46.854	0.0228	NA	No	No	No	No	No	No	No	NA	NA	No	NA	NA	NA	NA
Gdist:S210417_5705	BT02	46.854	0.0198	NA	No	No	No	No	No	Yes (0.008)	Yes (0.049)	NA	NA	No	NA	NA	NA	NA
Gdist:S99221_1923	BT02	46.854	0.0097	NA	No	No	No	No	No	No	No	NA	NA	No	NA	NA	NA	NA
Gdist:S119128_9527	BT02	46.855	0.0691	NA	No	No	No	No	No	No	No	0.530792308	0.05976563	Yes	NA	NA	NA	NA
Gdist:S471295_2952	BT02	46.864	0.0351	none	No	No	No	No	No	No	No	0.0095625	0.50813869	Yes	VAR RED	Reduced	NA	NA
SalarSNP:ESTV_21132_1397	BT02	47.405	0.0381	NA	No	No	No	No	No	No	No	0.928928571	0.46026971	Yes	NA	NA	NA	NA
Gdist:S116612_6115	BT02	47.947	0.0623	none	No	No	No	No	No	No	No	0	0.56791921	Yes	VAR RED	Reduced	NA	yes
Gdist:S105720_2405	BT02	49.7	-0.0078	NA	No	No	No	No	No	No	No	NA	NA	No	NA	NA	NA	NA
Gdist:S208103_7211	BT02	51.355	0.0276	NA	No	No	No	No	No	No	No	NA	NA	No	NA	NA	NA	NA
Gdist:S208103_3697	BT02	51.355	0.0118	NA	No	No	No	No	No	No	No	NA	NA	No	NA	NA	NA	NA
Gdist:S208103_1413	BT02	51.355	0.0045	NA	No	No	No	No	No	No	No	NA	NA	No	NA	NA	NA	NA
Gdist:S156829_2282	BT02	51.355	-0.0008	NA	Yes	No	No	No	No	No	No	NA	NA	No	NA	NA	NA	NA
Gdist:S208103_9047	BT02	51.355	-0.0015	NA	No	No	No	No	No	No	No	NA	NA	No	NA	NA	NA	NA
cDNA:S54802_2392	BT02	51.355	-0.0052	NA	No	No	No	No	No	No	No	NA	NA	No	NA	NA	NA	NA
Gdist:S245672_3136	BT02	51.41	0.0003	NA	No	No	No	No	No	No	No	NA	NA	No	NA	NA	NA	NA
Gdist:S541107_1153	BT02	51.473	-0.0047	NA	No	No	No	No	No	No	No	NA	NA	No	NA	NA	NA	NA
cDNA:C124640749_257	BT02	52.862	0.0346	NA	No	No	No	No	No	No	No	0.228567073	0.83441924	Yes	NA	NA	NA	NA
Gdist:S422860_7473	BT02	52.886	0.0047	NA	No	No	No	No	No	No	No	NA	NA	No	NA	NA	NA	NA
Gdist:S96958_980	BT02	52.889	0.0156	NA	No	No	No	No	No	No	No	NA	NA	No	NA	NA	NA	NA
Gdist:S96958_3517	BT02	53.084	0.0607	NA	No	No	No	No	No	No	No	0.66341224	0.67788613	Yes	NA	NA	NA	NA
Gdist:S283804_6359	BT02	53.088	0.0152	NA	No	No	No	No	No	No	No	NA	NA	No	NA	NA	NA	NA
SalarSNP:ESTV_15513_116	BT02	56.202	0.0044	NA	No	No	No	No	No	No	No	NA	NA	No	NA	NA	NA	NA
Gdist:S417363_2045	BT02	56.242	0.0822	NA	Yes	No	No	No	No	No	No	0.0669375	0.87768412	Yes	NA	NA	NA	NA
Gdist:S26647_1613	BT02	56.242	0.0229	NA	No	No	No	No	No	No	No	NA	NA	No	NA	NA	NA	NA
Gdist:S417363_4651	BT02	56.242	0.0207	NA	No	No	No	No	No	No	No	NA	NA	No	NA	NA	NA	NA
Gdist:S137633_1828	BT02	56.242	0.0112	NA	Yes	No	No	No	No	No	No	NA	NA	No	NA	NA	NA	NA
cDNA:S374302_1853	BT02	56.692	0.0535	PREDIC	No	No	No	No	No	Yes (0.003)	Yes (0.027)	0.760560928	0.04098214	Yes	SKJ RED	NA	reduced	NA
Gdist:S340130_4102	BT02	57.141	-0.0114	NA	No	No	No	No	No	Yes (0.023)	No	NA	NA	No	NA	NA	NA	NA
Gdist:S339656_1631	BT02	57.55	0.0251	NA	No	No	No	No	No	No	No	NA	NA	No	NA	NA	NA	NA
Gdist:S203400_2557	BT02	59.061	0.0515	NA	No	No	No	No	No	No	No	0.508712121	0.55438291	Yes	NA	NA	NA	NA
cDNA:S128086_5877	BT02	59.404	0.0024	NA	No	No	No	No	No	No	No	NA	NA	No	NA	NA	NA	NA
Gdist:S618718_1504	BT02	60.126	0.0374	NA	No	No	No	No	No	No	No	0.593534483	0.95357891	Yes	NA	NA	NA	NA
Gdist:S766843_3656	BT02	60.126	0.0367	NA	No	No	No	No	No	Yes (0.006)	Yes (0.042)	0.916490895	0.2664959	Yes	NA	NA	NA	NA
Gdist:S66089_6737	BT02	60.994	0.0212	NA	No	No	No	No	No	No	No	NA	NA	No	NA	NA	NA	NA
Gdist:S83070_5578	BT02	60.995	0.0034	NA	No	No	No	No	No	No	No	NA	NA	No	NA	NA	NA	NA
Gdist:S58363_4952	BT02	61.951	0.0064	NA	No	No	No	No	No	No	No	NA	NA	No	NA	NA	NA	NA
Gdist:S58363_10189	BT02	62.296	0.0124	NA	No	No	No	No	No	No	No	NA	NA	No	NA	NA	NA	NA
Gdist:S81377_10143	BT02	65.674	0.0068	NA	Yes	No	No	No	No	No	No	NA	NA	No	NA	NA	NA	NA
cDNA:S532222_1862	BT02	65.674	0.0019	NA	No	No	No	No	No	No	No	NA	NA	No	NA	NA	NA	NA
Gdist:S81377_9429	BT02	65.674	0.0018	NA	Yes	No	No	No	No	No	No	NA	NA	No	NA	NA	NA	NA
Gdist:S153878_3763	BT02	65.675	0.0215	NA	Yes	No	No	No	No	No	No	NA	NA	No	NA	NA	NA	NA
Gdist:S153878_9142	BT02	65.675	0.0179	NA	No	No	No	No	No	No	No	NA	NA	No	NA	NA	NA	NA
Gdist:S47346_3449	BT02	66.354	-0.003	NA	No	No	No	No	No	No	No	NA	NA	No	NA	NA	NA	NA
Gdist:S178882_7836	BT02	66.984	0.0097	NA	No	No	No	No	No	No	No	NA	NA	No	NA	NA	NA	NA
Gdist:S287246_2980	BT02	67.867	0.0371	NA	No	No	No	No	No	No	No	0.982691275	0.74923536	Yes	NA	NA	NA	NA
cDNA:S139461_1842	BT02	67.867	0.0327	NA	No	No	No	No	No	Yes (0.007)	Yes (0.048)	0.963553748	0.99074602	Yes	NA	NA	NA	NA
Gdist:S32826_7674	BT02	67.867	-0.005	NA	No	No	No	No	No	No	No	NA	NA	No	NA	NA	NA	NA
LD:S656103_13895	BT02	70.382	-0.0059	NA	No	No	No	No	No	No	No	NA	NA	No	NA	NA	NA	NA
LD:S656103_3922	BT02	70.383	0.1061	NA	Yes	no	no	No	No	No	No	NA	NA	no	NA	NA	NA	NA
Gdist:S61343_3133	BT02	70.384	-0.0009	NA	No	No	No	No	No	Yes (0.035)	No	NA	NA	No	NA	NA	NA	NA
Gdist:S132618_1961	BT02	72.036	0.0065	NA	No	No	No	No	No	No	No	NA	NA	No	NA	NA	NA	NA
Gdist:S29950_3487	BT02	74.279	0.0128	none	No	No	No	No	No	No	No	0.038898305	0.81521236	Yes	VAR RED	Reduced	NA	yes
cDNA:S127517_4651	BT02	76.756	0.0015	NA	No	No	No	No	No	No	No	NA	NA	No	NA	NA	NA	NA
SalHit:S108074_3154	BT02	78.862	-0.0097	NA	Yes	No	No	No	No	No	No	NA	NA	No	NA	NA	NA	NA
cDNA:S671649_1009	BT02	82.571	-0.001	NA	No	No	No	No	No	No	No	NA	NA	No	NA	NA	NA	NA
SalHit:S51494_16759	BT02	82.581	0.0074	NA	No	No	No	No	No	No	No	NA	NA	No	NA	NA	NA	NA
SalHit:S324937_1374	BT02	82.582	0.016	NA	No	No	No	No	No	No	No	NA	NA	No	NA	NA	NA	NA
SalHit:S51494_11690	BT02	82.582	0.0105	NA	No	No	No	No	No	Yes (0.007)	Yes (0.049)	NA	NA	No	NA	NA	NA	NA
SalHit:S51494_16577	BT02	82.582	-0.0047	NA	No	No	No	No	No	No	No	NA	NA	No	NA	NA	NA	NA
SalHit:S260262_255	BT02	83.101	0.0179	NA	No	No	No	No	No	No	No	NA	NA	No	NA	NA	NA	NA
SalHit:S295324_1768	BT02	86.469	0.001	NA	Yes	No	No	No	No	No	No	NA	NA	No	NA	NA	NA	NA
SalHit:S328044_4638	BT02	86.47	-0.0078	NA	No	No	No	No	No	No	No	NA	NA	No	NA	NA	NA	NA
cDNA:S479703_3376	BT02	86.475	0.0151	NA	No	No	No	No	No	No	No	NA	NA	No	NA	NA	NA	NA
SalHit:S834419_990	BT02	86.476	0.0048	NA	No	No	No	No	No	No	No	NA	NA	No	NA	NA	NA	NA
SalHit:S694964_1144	BT02	86.476	0.0012	NA	No	No	No	No	No	No	No	NA	NA	No	NA	NA	NA	NA
Gdist:S116897_7662	BT03	0	0.0175	NA	Yes	No	No	No	No	No	No	NA	NA	No	NA	NA	NA	NA
cDNA:S371791_1792	BT03	1.006	0.02	NA	No	No	No	No	No	No	No	NA	NA	No	NA	NA	NA	NA
Gdist:S497918_6157	BT03	1.643	0.0137	NA	No	No	No	No	No	No	No	NA	NA	No	NA	NA	NA	NA
SalHit:S146126_7561	BT03	1.643	-0.0036	NA	No	No	No	No	No	No	No	NA	NA	No	NA	NA	NA	NA
Gdist:S235573_978	BT03	1.653	0.007	NA	No	No	No	No	No	No	No	NA	NA	No	NA	NA	NA	NA
SalHit:S737061_1987	BT03	1.654	0.0263	NA	No	No	No	No	No	No	No	NA	NA	No	NA	NA	NA	NA
cDNA:S60342_1952	BT03	1.699	0.0032	NA	No	No	No	No	No	Yes (0.048)	No	NA	NA	No	NA	NA	NA	NA
Gdist:S51485_4707	BT03	1.742	0.0324	NA	No	No	No	No	No	No	No	0.760560928	0.30990876	Yes	NA	NA	NA	NA
Gdist:S432899_722	BT03	1.742	0.0117	NA	No	No	No	No	No	No	No	NA	NA	No	NA	NA	NA	NA
Gdist:S51485_7388	BT03	1.933	-0.003	NA	No	No	No	No	No	No	No	NA	NA	No	NA	NA	NA	NA

Gdist:S336760_6723	BT03	2.005	0.0226	NA	No	No	No	No	No	No	No	NA	NA	No	NA	NA	NA	NA
Gdist:S241314_7855	BT03	2.088	0.0541	NA	No	No	No	No	No	No	No	0.19921875	0.53217391	Yes	NA	NA	NA	NA
Gdist:S91493_123	BT03	2.088	0.031	NA	Yes	No	No	No	No	Yes (0.004)	Yes (0.035)	0.136607143	0.28322519	Yes	NA	NA	NA	NA
Gdist:S142540_7602	BT03	2.088	0.0054	NA	No	No	No	No	No	No	No	NA	NA	No	NA	NA	NA	NA
Gdist:S51105_3284	BT03	2.088	0.0002	NA	No	No	No	No	No	No	No	NA	NA	No	NA	NA	NA	NA
Gdist:S91493_4912	BT03	2.088	0.0346	PREDIC	No	No	No	No	No	Yes (0.021)	No	0.0095625	0.81712572	Yes	VAR RED	Reduced	NA	yes
Gdist:S198448_9718	BT03	2.582	0.0124	NA	Yes	No	No	No	No	No	No	NA	NA	No	NA	NA	NA	NA
Gdist:S654478_7872	BT03	3.582	0.0126	NA	No	No	No	No	No	No	No	NA	NA	No	NA	NA	NA	NA
Gdist:S404371_13551	BT03	4.568	0.0389	NA	No	No	No	No	No	No	No	0.145413223	0.0675	Yes	NA	NA	NA	NA
Gdist:S447787_774	BT03	4.608	0.0338	NA	No	No	No	No	No	No	No	0.514568404	0.87029851	Yes	NA	NA	NA	NA
SalHit:S1706_14812	BT03	4.688	0.002	NA	No	No	No	No	No	No	No	NA	NA	No	NA	NA	NA	NA
Gdist:S359414_5450	BT03	5.649	0.0163	NA	No	No	No	No	No	No	No	NA	NA	No	NA	NA	NA	NA
Gdist:S256948_4900	BT03	5.653	-0.0125	NA	No	No	No	No	No	No	No	NA	NA	No	NA	NA	NA	NA
Gdist:S256948_7714	BT03	5.727	0.0059	NA	No	No	No	No	No	No	No	NA	NA	No	NA	NA	NA	NA
Gdist:S97144_4172	BT03	5.801	0.0133	NA	No	No	No	No	No	No	No	NA	NA	No	NA	NA	NA	NA
Gdist:S188325_1153	BT03	6.842	-0.0117	NA	No	No	No	No	No	No	No	NA	NA	No	NA	NA	NA	NA
Gdist:S163875_5661	BT03	7.765	0.0247	NA	No	No	No	No	No	No	No	NA	NA	No	NA	NA	NA	NA
Gdist:S60540_5084	BT03	8.993	0.001	NA	No	No	No	No	No	No	No	NA	NA	No	NA	NA	NA	NA
Gdist:S251464_5874	BT03	8.994	0.0081	NA	No	No	No	No	No	No	No	NA	NA	No	NA	NA	NA	NA
Gdist:S124973_3491	BT03	9.837	0.0212	NA	No	No	No	No	No	No	No	NA	NA	No	NA	NA	NA	NA
cDNA:S255641_2125	BT03	11.601	-0.0046	NA	No	No	No	No	No	No	No	NA	NA	No	NA	NA	NA	NA
SalHit:S558206_4087	BT03	11.845	0.0129	NA	No	No	No	No	No	No	No	NA	NA	No	NA	NA	NA	NA
Gdist:S255289_12673	BT03	12.827	0.0109	NA	No	No	No	No	No	No	No	NA	NA	No	NA	NA	NA	NA
Gdist:S2588_9839	BT03	13.008	0.0087	NA	No	No	No	No	No	No	No	NA	NA	No	NA	NA	NA	NA
LD:S224709_1027	BT03	13.01	0.0133	NA	No	No	No	No	No	No	No	NA	NA	No	NA	NA	NA	NA
Gdist:S2588_1936	BT03	13.01	-0.0102	NA	No	No	No	No	No	No	No	NA	NA	No	NA	NA	NA	NA
LD:S224709_10589	BT03	13.04	0.0106	NA	No	No	No	No	No	No	No	NA	NA	No	NA	NA	NA	NA
SalHit:S375996_4447	BT03	13.069	0.0284	NA	No	No	No	No	No	No	No	NA	NA	No	NA	NA	NA	NA
Gdist:S309347_3109	BT03	15.407	-0.0053	NA	No	No	No	No	No	No	No	NA	NA	No	NA	NA	NA	NA
Gdist:S4658_6998	BT03	15.648	0.0159	NA	Yes	No	No	No	No	No	No	0.419853516	0.54853698	Yes	NA	NA	NA	NA
Gdist:S270252_7058	BT03	17.969	-0.0055	NA	No	No	No	No	No	No	No	NA	NA	No	NA	NA	NA	NA
Gdist:S225060_4090	BT03	18.112	0.0244	NA	No	No	No	No	No	No	No	NA	NA	No	NA	NA	NA	NA
cDNA:S519266_2405	BT03	18.112	0.0151	NA	No	No	No	No	No	No	No	NA	NA	No	NA	NA	NA	NA
Gdist:S300815_3696	BT03	18.112	0.0136	NA	No	No	No	No	No	No	No	NA	NA	No	NA	NA	NA	NA
cDNA:S322654_3410	BT03	18.112	-0.0008	NA	No	No	No	No	No	No	No	NA	NA	No	NA	NA	NA	NA
cDNA:S744265_3173	BT03	18.579	0.0146	NA	No	No	No	No	No	No	No	NA	NA	No	NA	NA	NA	NA
cDNA:S325743_4384	BT03	20.091	-0.0012	NA	No	No	No	No	No	No	No	NA	NA	No	NA	NA	NA	NA
cDNA:S325743_5924	BT03	20.149	0.0088	NA	No	No	No	No	No	No	No	NA	NA	No	NA	NA	NA	NA
cDNA:S423457_1288	BT03	20.224	0.0297	NA	No	No	No	No	No	No	No	NA	NA	No	NA	NA	NA	NA
cDNA:S423457_2457	BT03	20.224	-0.0037	NA	No	No	No	No	No	No	No	NA	NA	No	NA	NA	NA	NA
SalHit:S509135_6353	BT03	20.226	-0.0067	NA	No	No	No	No	No	No	No	NA	NA	No	NA	NA	NA	NA
SalHit:S285971_2992	BT03	20.228	-0.0075	NA	No	No	No	No	No	No	No	NA	NA	No	NA	NA	NA	NA
cDNA:S19762_6844	BT03	21.348	-0.0111	NA	No	No	No	No	No	No	No	NA	NA	No	NA	NA	NA	NA
Gdist:S135388_5797	BT03	21.487	0.0105	NA	No	No	No	No	No	No	No	NA	NA	No	NA	NA	NA	NA
Gdist:S150556_3024	BT03	21.487	0.0103	NA	No	No	No	No	No	No	No	NA	NA	No	NA	NA	NA	NA
Gdist:S150556_4538	BT03	21.487	0.005	NA	No	No	No	No	No	No	No	NA	NA	No	NA	NA	NA	NA
Gdist:S150556_9200	BT03	21.491	-0.0089	NA	No	No	No	No	No	No	No	NA	NA	No	NA	NA	NA	NA
cDNA:C123610732_215	BT03	27.614	-0.0032	NA	No	No	No	No	No	No	No	NA	NA	No	NA	NA	NA	NA
SalHit:S85177_10121	BT03	28.068	0.0168	NA	No	No	No	No	No	No	No	NA	NA	No	NA	NA	NA	NA
SalHit:S175270_692	BT03	29.93	0.0345	NA	No	No	No	No	No	No	No	0.834737569	0.2645625	Yes	NA	NA	NA	NA
cDNA:C124692505_310	BT03	29.999	0.0034	NA	Yes	No	No	No	No	No	No	0.05961039	0.71946429	Yes	NA	NA	NA	NA
cDNA:S55677_6075	BT03	31.414	0.0132	NA	No	No	No	No	No	No	No	NA	NA	No	NA	NA	NA	NA
cDNA:S111663_3454	BT03	32.65	0.0009	NA	No	No	No	No	No	No	No	NA	NA	No	NA	NA	NA	NA
cDNA:S399531_1848	BT03	33.411	0.0288	NA	No	No	No	No	No	No	No	NA	NA	No	NA	NA	NA	NA
cDNA:S519981_717	BT03	35.998	0.0193	NA	No	No	No	No	No	No	No	NA	NA	No	NA	NA	NA	NA
cDNA:S191954_2250	BT03	38.584	0.0265	NA	No	No	No	No	No	No	No	NA	NA	No	NA	NA	NA	NA
Gdist:S417810_2571	BT03	38.617	0.0787	NA	No	No	No	No	No	No	No	0.565105932	0.69186047	Yes	NA	NA	NA	NA
cDNA:S124728_2350	BT03	38.617	0.0162	NA	No	No	No	No	No	No	No	NA	NA	No	NA	NA	NA	NA
Gdist:S67022_483	BT03	39.835	-0.0113	NA	No	No	No	No	No	No	No	NA	NA	No	NA	NA	NA	NA
SalHit:S90996_1668	BT03	41.066	0.0242	NA	No	No	No	No	No	No	No	NA	NA	No	NA	NA	NA	NA
Gdist:S264671_8990	BT03	42.377	0.0169	NA	No	No	No	No	No	No	No	NA	NA	No	NA	NA	NA	NA
Gdist:S73077_288	BT03	42.379	0.0204	NA	No	No	No	No	No	No	No	NA	NA	No	NA	NA	NA	NA
SalHit:S752338_5854	BT03	42.717	-0.0025	NA	No	No	No	No	No	No	No	NA	NA	No	NA	NA	NA	NA
Gene:S787535_1439	BT03	43.054	0.0121	NA	No	No	No	No	No	Yes (0.037)	No	NA	NA	No	NA	NA	NA	NA
Gdist:S119058_6641	BT03	44.908	-0.0041	NA	No	No	No	No	No	No	No	NA	NA	No	NA	NA	NA	NA
Gdist:S19011_5711	BT03	44.91	-0.0094	NA	No	No	No	No	No	No	No	NA	NA	No	NA	NA	NA	NA
cDNA:S518687_590	BT03	44.93	0.0017	NA	No	No	No	No	No	No	No	NA	NA	No	NA	NA	NA	NA
SalHit:S10486_5485	BT03	46.333	0.0077	NA	No	No	No	No	No	No	No	NA	NA	No	NA	NA	NA	NA
Gdist:S83796_6059	BT03	47.777	0.0117	NA	No	No	No	No	No	No	No	NA	NA	No	NA	NA	NA	NA
cDNA:S282124_239	BT03	47.933	0.0239	NA	No	No	No	No	No	No	No	NA	NA	No	NA	NA	NA	NA
Gdist:S110756_790	BT03	47.934	0.0113	NA	No	No	No	No	No	No	No	NA	NA	No	NA	NA	NA	NA
cDNA:S292910_1208	BT03	47.935	0.0218	NA	Yes	No	No	No	No	No	No	NA	NA	No	NA	NA	NA	NA
Gdist:S12320_3350	BT03	47.935	0.0094	NA	No	No	No	No	No	No	No	NA	NA	No	NA	NA	NA	NA
cDNA:S280691_409	BT03	48.244	0.0001	NA	No	No	No	No	No	No	No	NA	NA	No	NA	NA	NA	NA
Gdist:S164995_6490	BT03	48.496	0.0211	NA	No	No	No	No	No	Yes (0.004)	Yes (0.035)	NA	NA	No	NA	NA	NA	NA
Gdist:S358300_3992	BT03	48.496	0.0147	NA	No	No	No	No	No	No	No	NA	NA	No	NA	NA	NA	NA
Gdist:S146429_6829	BT03	48.496	0.0067	NA	No	No	No	No	No	Yes (0.002)	Yes (0.022)	NA	NA	No	NA	NA	NA	NA
Gdist:S48546_6758	BT03	48.496	-0.0031	NA	No	No	No	No	No	No	No	NA	NA	No	NA	NA	NA	NA
Gdist:S369028_9865	BT03	48.496	-0.0099	NA	No	No	No	No	No	No	No	NA	NA	No	NA	NA	NA	NA
Gdist:S2911_2429	BT03	48.498	0.0652	NA	No	No	No	No	No	Yes (0.002)	Yes (0.024)	0.513825	0.4815	Yes	NA	NA	NA	NA
Gdist:S1595_5752	BT03	50.694	0.0425	NA	No	No	No	No	No	No	No	0.71335443	0.88194268	Yes	NA	NA	NA	NA
Gdist:S202358_6188	BT03	50.823	0.02	NA	No	No	No	No	No	No	No	NA	NA	No	NA	NA	NA	NA
Gdist:S56030_4839	BT03	50.95	0.0334	NA	No	No	No	No	No	No	No	0.838743243	0.84780928	Yes	NA	NA	NA	NA
Gdist:S220297_5487	BT03	50.95	0.0302	NA	No	No	No	No	No	Yes (0.007)	Yes (0.047)	0.947958815	0.56791921	Yes	NA	NA	NA	NA
Gdist:S31368_4306	BT03	50.95	0.0265	NA	No	No	No	No	No	No	No	NA	NA	No	NA	NA	NA	NA
Gdist:S60704_5495	BT03	50.95	0.0132	NA	No	No	No	No	No	No	No	NA	NA	No	NA	NA	NA	NA
Gdist:S279953_863	BT03	50.95	0.005	NA	No	No	No	No	No	No	No	NA	NA	No	NA	NA	NA	NA
Gdist:S13965_9753	BT03	50.95	-0.0093	NA	No	No	No	No	No	No	No	NA	NA	No	NA	NA	NA	NA
SalHit:S78704_4164	BT03	50.95	-0.0103	NA	No	No	No	No	No	No	No	NA	NA	No	NA	NA	NA	NA
SalHit:S353786_532	BT03	52.789	0.0027	NA	No	No	No	No	No	No	No	NA	NA	No	NA	NA	NA	NA
Gdist:S489500_6605	BT03	52.821	0.0079	NA	No	No	No	No	No	No	No	NA	NA	No	NA	NA	NA	NA
SalHit:S164388_623	BT03	52.853	0.0132	NA	No	No	No	No	No	No	No	NA	NA	No	NA	NA	NA	NA
Gdist:S135079_5018	BT03	52.854	0.0503	NA	No	No	No	No	No	No	No	0.262581081	0.8128125	Yes	NA	NA	NA	NA
cDNA:S86519_8078	BT03	52.854	0.0116	NA	No	No	No	No	No	No	No	NA	NA	No	NA	NA	NA	NA
cDNA:S506450_638	BT03	52.854	-0.0033	NA	No	No	No	No	No	No	No	NA	NA	No	NA	NA	NA	NA
Gdist:S265308_3482	BT03	53.614	0.0226	NA	No	No	No	No	No	No	No	NA	NA	No	NA	NA	NA	NA
Gdist:S29534_6024	BT03	53.614	0.0175	NA	No	No	No	No	No	No	No	NA	NA	No	NA	NA	NA	NA
Gdist:S21757_2202	BT03	53.614	0.0165	NA	No	No	No	No	No	No	No	NA	NA	No	NA	NA	NA	NA
Gdist:S9126_6370	BT03	53.615	0.0055	NA	No	No	No	No	No	No	No	NA	NA	No	NA	NA	NA	NA
SalHit:S808420_898	BT03	53.615	0.0045	NA	No	No	No	No	No	No	No	NA	NA	No	NA	NA	NA	NA
cDNA:S324619_4826	BT03	53.84	0.027	NA	No	No	No	No	No	No	No	NA	NA	No	NA	NA	NA	NA
cDNA:S100723_472	BT03	54.045	0.0311	NA	No	No	No	No	No	No	No	0.340204327	0.89481818	Yes	NA	NA	NA	NA
SalHit:S254544_1255	BT03	54.045	0.0181	NA	No	No	No	No	No	No	No	0.870811856	0.96734461	Yes	NA	NA	NA	NA
cDNA:S195837_3240	BT03	54.193	0.0902	NA	No	No	No	No	No	No	No	0.17136	0.88547244	Yes	NA	NA	NA	NA
Gdist:S423022_8982	BT03	54.193	0.0912	NA	No	No	No	No	No	No	No	0.028867925	0.89727171	Yes	VAR INC	increased	NA	NA
Gene:S237713_2650	BT03	54.341	0.0538	NA	No	No	No	No	No	No	No	0.14025	0.93137645	Yes	NA	NA	NA	NA
Gene:S237713_1631	BT03	54.443	0.0371	NA	No	No	No	No	No	No	No	0.614833333	0.4067561	Yes	NA	NA	NA	NA
Gdist:S517991_1055	BT03	54.553	0.0209	NA	Yes	No	No	No	No	No	No	NA	NA	No	NA	NA	NA	NA
Gdist:S16260_2275	BT03	57.007	0.0583	NA	No	No	No	No	No	No	No	0.928928571	0.99439894	Yes	NA	NA	NA	NA
cDNA:S232646_1656	BT03	57.007	0.0265	NA	No	No	No	No	No	Yes (0.037)	No	NA	NA	No	NA	NA	NA	NA
Gdist:S10968_860	BT03	57.007	0.0125	NA	No	No	No	No	No	No	No	NA	NA	No	NA	NA	NA	NA
Gdist:S16260_2531	BT03	57.172	0.0201	NA	No	No	No	No	No	No	No	NA	NA	No	NA	NA	NA	NA
cDNA:S115958_1426	BT03	57.317	0.023	NA	No	No	No	No	No	No	No	NA	NA	No	NA	NA	NA	NA
Gdist:S332628_939	BT03	57.446	0.0378	NA	No	No	No	No	No	No	No	0.988505976	0.99011984	Yes	NA	NA	NA	NA
Gdist:S422914_5157	BT03	57.446	0.0162	NA	No	No	No	No	No	No	No	NA	NA	No	NA	NA	NA	NA
cDNA:S227505_6398	BT03	57.446	0.0113	NA	No	No	No	No	No	No	No	NA	NA	No	NA	NA	NA	NA
Gdist:S610843_1269	BT03	57.446	0.0077	NA	No	No	No	No	No	No	No	NA	NA	No	NA	NA	NA	NA
Gdist:S332628_7004	BT03	58.024	0.008	NA	No	No	No	No	No	No	No	NA	NA	No	NA	NA	NA	NA
Gdist:S132332_6141	BT03	58.595	0.047	NA	No	No	No	No	No	No	No	0.906372	0.41482394	Yes	NA	NA	NA	NA
Gdist:S201640_7954	BT03	58.595	0.0401	NA	Yes	No	No	No	No	No	No	0.906372	0.60473464	Yes	NA	NA	NA	NA
Gdist:S201640_7514	BT03	58.595	0.0392	NA	Yes	No	No	No	No	No	No	0.70977707	0.71368902	Yes	NA	NA	NA	NA
cDNA:S333249_277	BT03	58.595	0.0215	NA	No	No	No	No	No	No	No	NA	NA	No	NA	NA	NA	NA
Gdist:S45516_2538	BT03	58.595	0.0098	NA	No	No	No	No	No	No	No	NA	NA	No	NA	NA	NA	NA
Gdist:S106496_4572	BT03	58.595	0.0023	NA	No	No	No	No	No	Yes (0.004)	Yes (0.035)	NA	NA	No	NA	NA	NA	NA
Gdist:S350240_281	BT03	58.595	0.0011	NA	No	No	No	No	No	No	No	NA	NA	No	NA	NA	NA	NA
cDNA:S275534_2354	BT03	58.595	0.0003	NA	No	No	No	No	No	No	No	NA	NA	No	NA	NA	NA	NA
Gdist:S15291_5095	BT03	58.595	-0.0138	NA	No	No	No	No	No	No	No	NA	NA	No	NA	NA	NA	NA

Gdist:S216938_2222	BT03	59.039	0.0314	NA	No	No	No	No	No	No	No	0.817611524	0.55438291	Yes	NA	NA	NA	NA
Gdist:S292709_6375	BT03	59.039	0.0168	NA	No	No	No	No	No	Yes (0.044)	No	NA	NA	No	NA	NA	NA	NA
Gdist:S89159_686	BT03	59.039	-0.0036	NA	No	No	No	No	No	No	No	NA	NA	No	NA	NA	NA	NA
Gdist:S529069_6981	BT03	59.039	-0.0085	NA	No	No	No	No	No	No	No	NA	NA	No	NA	NA	NA	NA
Gdist:S292656_2541	BT03	61.734	-0.0013	NA	No	No	No	No	No	No	No	NA	NA	No	NA	NA	NA	NA
Gdist:S153277_4973	BT03	61.734	-0.0079	NA	No	No	No	No	No	No	No	NA	NA	No	NA	NA	NA	NA
Gdist:S68084_1916	BT03	61.734	-0.0107	NA	No	No	No	No	No	No	No	NA	NA	No	NA	NA	NA	NA
cDNA:S674382_1528	BT03	61.778	0.012	NA	No	No	No	No	No	No	No	NA	NA	No	NA	NA	NA	NA
cDNA:S312955_1490	BT03	64.729	0.0026	NA	No	No	No	No	No	No	No	NA	NA	No	NA	NA	NA	NA
Gdist:S129848_5047	BT04	0	0.0113	NA	No	No	No	No	No	No	No	NA	NA	No	NA	NA	NA	NA
Gdist:S366479_1931	BT04	1.259	0.003	NA	No	No	No	No	No	Yes (0.019)	No	NA	NA	No	NA	NA	NA	NA
cDNA:S590793_284	BT04	5.08	0.0343	NA	No	No	No	No	No	No	No	0.845991993	0.83654676	Yes	NA	NA	NA	NA
SalHit:S481938_1027	BT04	8.805	0.0106	NA	No	No	No	No	No	No	No	NA	NA	No	NA	NA	NA	NA
Gdist:S49275_688	BT04	9.811	0.0233	NA	No	No	No	No	No	No	No	NA	NA	No	NA	NA	NA	NA
SalHit:S531204_1936	BT04	9.82	0.0119	NA	No	No	No	No	No	No	No	NA	NA	No	NA	NA	NA	NA
SalHit:S453677_1818	BT04	9.829	-0.0018	NA	No	No	No	No	No	No	No	NA	NA	No	NA	NA	NA	NA
Gdist:S240868_1740	BT04	10.318	-0.0008	NA	No	No	No	No	No	Yes (0.014)	No	NA	NA	No	NA	NA	NA	NA
cDNA:S689999_655	BT04	17.614	0.0078	NA	No	No	No	No	No	No	No	NA	NA	No	NA	NA	NA	NA
cDNA:S685260_460	BT04	17.627	0.0699	none	No	No	No	No	No	No	No	0	0.82025797	Yes	VAR RED	Reduced	NA	NA
SalarSNP:GCR_cBin19351_Ctg	BT04	20.488	-0.0059	NA	No	No	No	No	No	No	No	NA	NA	No	NA	NA	NA	NA
Gdist:S215162_7375	BT04	23.575	0.0214	NA	Yes	No	No	No	No	No	No	NA	NA	No	NA	NA	NA	NA
Gdist:S61644_9984	BT04	23.576	0.0299	NA	No	No	No	No	No	No	No	NA	NA	No	NA	NA	NA	NA
SalHit:S300505_4118	BT04	23.576	0.0152	NA	No	No	No	No	No	No	No	NA	NA	No	NA	NA	NA	NA
Gdist:S445228_1192	BT04	23.576	0.0107	NA	No	No	No	No	No	No	No	NA	NA	No	NA	NA	NA	NA
cDNA:C124745537_293	BT04	23.576	0.0093	NA	No	No	No	No	No	No	No	NA	NA	No	NA	NA	NA	NA
Gdist:S399414_6513	BT04	23.576	0.0078	NA	No	No	No	No	No	No	No	NA	NA	No	NA	NA	NA	NA
cDNA:C124745537_734	BT04	23.576	0.0033	NA	No	No	No	No	No	No	No	NA	NA	No	NA	NA	NA	NA
Gdist:S400310_5517	BT04	23.576	-0.0017	NA	Yes	No	No	No	No	No	No	NA	NA	No	NA	NA	NA	NA
Gdist:S36312_7871	BT04	23.576	-0.0021	NA	No	No	No	No	No	No	No	NA	NA	No	NA	NA	NA	NA
cDNA:S760869_330	BT04	23.576	-0.0063	NA	No	No	No	No	No	No	No	NA	NA	No	NA	NA	NA	NA
Gdist:S122598_1256	BT04	23.576	0.0758	NA	Yes	No	No	No	No	No	No	0.038898305	0.68310705	Yes	VAR INC	increased	NA	NA
cDNA:S146372_1828	BT04	23.576	0.0691	none	No	No	No	No	No	No	No	0	0.87794046	Yes	VAR RED	Reduced	NA	NA
Gdist:S430420_2648	BT04	24.697	-0.0028	NA	No	No	No	No	No	No	No	NA	NA	No	NA	NA	NA	NA
Gdist:S105008_3937	BT04	25.37	-0.0042	NA	No	No	No	No	No	No	No	NA	NA	No	NA	NA	NA	NA
cDNA:S123137_914	BT04	26.362	-0.0043	NA	No	No	No	No	No	No	No	NA	NA	No	NA	NA	NA	NA
LD:S189168_759	BT04	26.646	0.1182	NA	No	No	No	No	No	No	No	0.078238636	0.82025797	Yes	NA	NA	NA	NA
LD:S189168_9385	BT04	26.689	0.0247	NA	No	No	No	No	No	No	No	NA	NA	No	NA	NA	NA	NA
SalHit:S434130_330	BT04	27.677	0.0021	NA	No	No	No	No	No	No	No	NA	NA	No	NA	NA	NA	NA
Gdist:S178796_525	BT04	30.512	0.0146	NA	No	No	No	No	No	Yes (0)	Yes (0.006)	NA	NA	No	NA	NA	NA	NA
cDNA:S120620_363	BT04	30.912	0.0038	NA	No	No	No	No	No	No	No	NA	NA	No	NA	NA	NA	NA
Gdist:S87694_5787	BT04	33.815	0.032	NA	No	No	No	No	No	No	No	0.992172619	0.73333893	Yes	NA	NA	NA	NA
Gdist:S206753_14733	BT04	37.683	0.0404	NA	No	No	No	No	No	No	No	0.213317308	0.51871403	Yes	NA	NA	NA	NA
Gdist:S71066_4535	BT04	44.389	0.0063	NA	No	No	No	No	No	No	No	NA	NA	No	NA	NA	NA	NA
Gdist:S363298_10405	BT04	48.087	0.0026	NA	No	No	No	No	No	No	No	NA	NA	No	NA	NA	NA	NA
cDNA:S160324_5645	BT04	49.529	-0.0022	NA	No	No	No	No	No	No	No	NA	NA	No	NA	NA	NA	NA
SalHit:S781410_2479	BT04	50.729	0.0115	NA	No	No	No	No	No	Yes (0.014)	No	NA	NA	No	NA	NA	NA	NA
Gdist:S33583_3025	BT04	51.377	0.0337	NA	No	No	No	No	No	No	No	0.603632813	0.46026971	Yes	NA	NA	NA	NA
cDNA:S252719_505	BT04	52.68	0.0253	NA	No	No	No	No	No	No	No	NA	NA	No	NA	NA	NA	NA
cDNA:S53964_3872	BT04	57.029	0.0146	NA	No	No	No	No	No	No	No	NA	NA	No	NA	NA	NA	NA
Gdist:S108412_1150	BT04	60.224	0.0526	NA	No	No	No	No	No	Yes (0.019)	No	0.453596654	0.20131579	Yes	NA	NA	NA	NA
cDNA:S109135_5389	BT04	60.257	0.018	NA	Yes	No	No	No	No	No	No	NA	NA	No	NA	NA	NA	NA
cDNA:S109135_8210	BT04	60.257	0.0073	NA	No	No	No	No	No	No	No	NA	NA	No	NA	NA	NA	NA
Gdist:S50994_9770	BT04	61.439	0.0143	NA	No	No	No	No	No	No	No	NA	NA	No	NA	NA	NA	NA
Gdist:S433022_5017	BT04	61.483	0.0231	NA	Yes	No	No	No	No	No	No	NA	NA	No	NA	NA	NA	NA
Gdist:S90994_2281	BT04	61.526	0.0428	NA	Yes	No	No	No	No	No	No	0.181478102	0.99074602	Yes	NA	NA	NA	NA
LD:S559071_729	BT04	62.601	0.037	NA	No	No	No	No	No	No	No	0.968035714	0.26256356	Yes	NA	NA	NA	NA
Gdist:S217107_2755	BT04	63.066	0.0883	none	No	Yes	No	No	No	No	No	0.468256228	0.24589286	Yes	Outlier Varde	Outlier Var	NA	yes
SalHit:S20943_3475	BT04	64.081	0.007	NA	No	No	No	No	No	No	No	NA	NA	No	NA	NA	NA	NA
Gene:S642560_577	BT04	65.115	0.0015	NA	No	No	No	No	No	No	No	NA	NA	No	NA	NA	NA	NA
Gene:S642560_3839	BT04	65.129	-0.0013	NA	No	No	No	No	No	No	No	NA	NA	No	NA	NA	NA	NA
Gdist:S430300_4960	BT04	66.04	0.0077	NA	No	No	No	No	No	Yes (0.004)	Yes (0.035)	NA	NA	No	NA	NA	NA	NA
SalHit:S65493_4081	BT04	66.088	0.0064	NA	No	No	No	No	No	No	No	NA	NA	No	NA	NA	NA	NA
Gdist:S517371_4139	BT04	67.824	0.0069	NA	No	No	No	No	No	No	No	NA	NA	No	NA	NA	NA	NA
SalHit:S62399_1028	BT04	68.087	-0.0036	NA	No	No	No	No	No	No	No	0.565105932	0.89499248	Yes	NA	NA	NA	NA
SalHit:S240613_4416	BT04	69.085	0.0299	NA	No	No	No	No	No	No	No	NA	NA	No	NA	NA	NA	NA
Gdist:S192776_7473	BT04	69.086	0.0077	NA	No	No	No	No	No	No	No	NA	NA	No	NA	NA	NA	NA
Gdist:S250809_7698	BT04	69.086	-0.0029	NA	No	No	No	No	No	No	No	NA	NA	No	NA	NA	NA	NA
Gdist:S231756_1430	BT04	69.551	0.0063	NA	No	No	No	No	No	No	No	NA	NA	No	NA	NA	NA	NA
Gdist:S231756_9169	BT04	69.562	-0.0026	NA	No	No	No	No	No	No	No	NA	NA	No	NA	NA	NA	NA
cDNA:S78201_3151	BT04	69.574	0.0256	NA	No	No	No	No	No	No	No	NA	NA	No	NA	NA	NA	NA
Gdist:S100408_780	BT04	69.574	0.0245	NA	No	No	No	No	No	No	No	NA	NA	No	NA	NA	NA	NA
Gdist:S150027_10819	BT04	69.574	0.0138	NA	No	No	No	No	No	No	No	NA	NA	No	NA	NA	NA	NA
Gdist:S150027_8437	BT04	69.574	-0.003	NA	No	No	No	No	No	No	No	NA	NA	No	NA	NA	NA	NA
Gdist:S273201_5307	BT04	69.575	0.0222	NA	No	No	No	No	No	No	No	NA	NA	No	NA	NA	NA	NA
LD:S38447_13299	BT04	69.576	0.0272	NA	No	No	No	No	No	No	No	NA	NA	No	NA	NA	NA	NA
cDNA:S47755_4734	BT04	69.577	-0.0066	NA	No	No	No	No	No	Yes (0.002)	Yes (0.024)	NA	NA	No	NA	NA	NA	NA
Gdist:S6494_4711	BT04	71.435	0.0138	NA	No	No	No	No	No	No	No	NA	NA	No	NA	NA	NA	NA
Gdist:S96008_3689	BT04	73.459	0.0002	NA	No	No	No	No	No	No	No	NA	NA	No	NA	NA	NA	NA
Gene:S523738_1899	BT04	73.459	-0.0031	NA	No	No	No	No	No	No	No	NA	NA	No	NA	NA	NA	NA
Gdist:S6494_964	BT04	73.459	-0.0101	NA	No	No	No	No	No	No	No	NA	NA	No	NA	NA	NA	NA
Gdist:S229737_2647	BT04	73.685	-0.0021	NA	No	No	No	No	No	No	No	NA	NA	No	NA	NA	NA	NA
Gdist:S414051_9168	BT04	73.807	0.0012	NA	No	No	No	No	No	Yes (0.002)	Yes (0.024)	NA	NA	No	NA	NA	NA	NA
Gdist:S22527_8212	BT04	77.955	0.0095	NA	No	No	No	No	No	No	No	NA	NA	No	NA	NA	NA	NA
Gene:S789955_2465	BT04	78.387	0.0551	NA	No	No	No	No	No	No	No	0.14025	0.87029851	Yes	NA	NA	NA	NA
Gdist:S830668_2442	BT04	78.517	-0.014	NA	No	No	No	No	No	No	No	NA	NA	No	NA	NA	NA	NA
cDNA:S710664_1215	BT04	78.531	-0.0023	NA	No	No	No	No	No	No	No	NA	NA	No	NA	NA	NA	NA
Gdist:S204483_4329	BT04	78.639	-0.0012	NA	No	No	No	No	No	No	No	NA	NA	No	NA	NA	NA	NA
Gdist:S346427_10827	BT04	82.379	0.02	NA	No	No	No	No	No	No	No	NA	NA	No	NA	NA	NA	NA
Gdist:S37877_3561	BT04	82.379	0	NA	No	No	No	No	No	No	No	NA	NA	No	NA	NA	NA	NA
Gdist:S64444_2754	BT04	82.379	-0.0108	NA	No	No	No	No	No	No	No	NA	NA	No	NA	NA	NA	NA
Gdist:S64444_2675	BT04	82.38	0.0074	NA	No	No	No	No	No	No	No	NA	NA	No	NA	NA	NA	NA
Gdist:S491469_9222	BT04	84.125	0.0229	NA	Yes	No	No	No	No	Yes (0.022) (*als	Yes (0.024)	NA	NA	No	NA	NA	NA	NA
Gdist:S513060_2174	BT04	85.889	-0.0129	NA	No	No	No	No	No	No	No	NA	NA	No	NA	NA	NA	NA
SalarSNP:ESTV_13137_137	BT04	86.147	0.0099	NA	No	No	No	No	No	No	No	NA	NA	No	NA	NA	NA	NA
Gdist:S101169_6923	BT04	86.151	0.0213	NA	No	No	No	No	No	No	No	NA	NA	No	NA	NA	NA	NA
Gdist:S132944_1725	BT04	86.155	0.0445	NA	No	No	No	No	No	No	No	0.963553748	0.33346154	Yes	NA	NA	NA	NA
SalHit:S58313_4910	BT04	86.155	-0.0091	NA	No	No	No	No	No	No	No	NA	NA	No	NA	NA	NA	NA
Gdist:S51520_3916	BT04	86.392	0.002	NA	No	No	No	No	No	No	No	NA	NA	No	NA	NA	NA	NA
Gdist:S408665_3579	BT04	86.396	0.0362	NA	No	No	No	No	No	Yes (0.043)	No	0.893131188	0.22871134	Yes	NA	NA	NA	NA
SalHit:S43861_1436	BT04	86.396	0.0283	NA	No	No	No	No	No	No	No	0.667191781	0.56791921	Yes	NA	NA	NA	NA
Gdist:S408665_5512	BT04	86.396	0.0136	NA	No	No	No	No	No	No	No	NA	NA	No	NA	NA	NA	NA
SalHit:S43861_2337	BT04	86.397	0.0079	NA	No	No	No	No	No	No	No	NA	NA	No	NA	NA	NA	NA
cDNA:C122548872_148	BT04	87.522	0.0009	NA	No	No	No	No	No	Yes (0.004)	Yes (0.035)	NA	NA	No	NA	NA	NA	NA
cDNA:S79964_174	BT05	0	0.0047	NA	No	No	No	No	No	No	No	NA	NA	No	NA	NA	NA	NA
cDNA:S255040_2501	BT05	0.066	0.0169	NA	No	No	No	No	No	No	No	NA	NA	No	NA	NA	NA	NA
cDNA:S293277_438	BT05	0.068	0.0399	NA	No	No	No	No	No	No	No	0.096631579	0.83808482	Yes	NA	NA	NA	NA
cDNA:S229093_237	BT05	0.068	0.0273	NA	No	No	No	No	No	No	No	0.606521739	0.89499248	Yes	NA	NA	NA	NA
cDNA:C124241706_353	BT05	0.068	0.0297	NA	No	No	No	No	No	No	No	NA	NA	No	NA	NA	NA	NA
cDNA:S655513_1414	BT05	0.068	-0.0075	NA	No	No	No	No	No	No	No	NA	NA	No	NA	NA	NA	NA
cDNA:S309721_1176	BT05	0.068	0.046	none	Yes	No	No	No	No	Yes (0.02)	No	0	0.98991	Yes	VAR RED	Reduced	NA	yes
SalHit:S530137_1949	BT05	1.277	0.0107	NA	No	No	No	No	No	No	No	NA	NA	No	NA	NA	NA	NA
Gdist:S574163_10014	BT05	1.357	0.0104	NA	No	No	No	No	No	No	No	NA	NA	No	NA	NA	NA	NA
Gdist:S42556_9071	BT05	3.946	0.0299	NA	No	No	No	No	No	No	No	NA	NA	No	NA	NA	NA	NA
Gdist:S35585_2670	BT05	4.117	-0.0019	NA	No	No	No	No	No	No	No	NA	NA	No	NA	NA	NA	NA
cDNA:S730927_529	BT05	5.342	0.0402	NA	No	No	No	No	No	No	No	0.870811856	0.70391278	Yes	NA	NA	NA	NA
Gdist:S95603_3558	BT05	5.375	-0.0051	NA	No	No	No	No	No	No	No	NA	NA	No	NA	NA	NA	NA
Gdist:S359627_16667	BT05	5.376	0.0409	NA	No	No	No	No	No	No	No	0.738009073	0.87029851	Yes	NA	NA	NA	NA
Gdist:S612707_3758	BT05	5.376	0.036	NA	No	No	No	No	No	No	No	0.916490895	0.77613306	Yes	NA	NA	NA	NA
Gdist:S34545_1552	BT05	5.376	0.0108	NA	No	No	No	No	No	No	No	NA	NA	No	NA	NA	NA	NA
Gdist:S68367_5660	BT05	6.934	0.0245	NA	No	No	No	No	No	No	No	NA	NA	No	NA	NA	NA	NA
SalHit:S29463_5965	BT05	6.934	0.0213	NA	No	No	No	No	No	No	No	NA	NA	No	NA	NA	NA	NA
SalHit:S826387_869	BT05	6.934	0.0205	NA	No	No	No	No	No	No	No	NA	NA	No	NA	NA	NA	NA
Gdist:S276066_8390	BT05	6.934	0.0109	NA	Yes	No	No	No	No	No	No	NA	NA	No	NA	NA	NA	NA
Gdist:S68367_2220	BT05	6.934	0.0103	NA	No	No	No	No	No	Yes (0.001)	Yes (0.014)	NA	NA	No	NA	NA	NA	NA
Gdist:S92499_6804	BT05	6.934	0.0013	NA	No	No	No	No	No	No	No	NA	NA	No	NA	NA	NA	NA
Gdist:S68367_6156	BT05	6.934	-0.0098	NA	No	No	No	No	No	No	No	NA	NA	No	NA	NA	NA	NA
cDNA:S830139_334	BT05	9.016	0.0237	NA	No	No	No	No	No	No	No	NA	NA	No	NA	NA	NA	NA

cDNA:S525580_1370	BT05	9.101	0.008	NA	No	No	No	No	No	No	No	NA	NA	No	NA	NA	NA	NA
Gdist:S230251_3848	BT05	11.263	0.0545	NA	No	No	No	No	No	No	No	0.508712121	0.51277174	Yes	NA	NA	NA	NA
Gdist:S63731_2375	BT05	11.263	0.0142	NA	No	No	No	No	No	No	No	NA	NA	No	NA	NA	NA	NA
Gdist:S529500_1136	BT05	11.263	0.0101	NA	No	No	No	No	No	No	No	NA	NA	No	NA	NA	NA	NA
SalHit:S233811_1875	BT05	11.263	0.0062	NA	No	No	No	No	No	No	No	NA	NA	No	NA	NA	NA	NA
SalHit:S233811_2086	BT05	11.263	0.0051	NA	No	No	No	No	No	Yes (0.002)	Yes (0.024)	NA	NA	No	NA	NA	NA	NA
Gdist:S230251_6244	BT05	11.263	0.0034	NA	No	No	No	No	No	No	No	NA	NA	No	NA	NA	NA	NA
Gdist:S136603_6180	BT05	11.263	0.0014	NA	No	No	No	No	No	No	No	NA	NA	No	NA	NA	NA	NA
cDNA:S68403_1523	BT05	11.263	0.0332	PREDIC	No	No	No	No	No	No	No	0.023418367	0.87956522	Yes	VAR RED	Reduced	NA	NA
SalHit:S279268_1478	BT05	13.058	-0.0019	NA	No	No	No	No	No	No	No	NA	NA	No	NA	NA	NA	NA
Gdist:S253720_4338	BT05	13.667	0.0259	NA	No	No	No	No	No	No	No	NA	NA	No	NA	NA	NA	NA
LD:S183034_13487	BT05	13.667	0.0235	NA	No	No	No	No	No	No	No	NA	NA	No	NA	NA	NA	NA
Gdist:S253720_7389	BT05	13.667	0.0225	NA	No	No	No	No	No	No	No	NA	NA	No	NA	NA	NA	NA
Gdist:S681888_6399	BT05	13.667	0.0193	NA	No	No	No	No	No	No	No	NA	NA	No	NA	NA	NA	NA
Gdist:S253720_4988	BT05	13.667	0.0083	NA	No	No	No	No	No	No	No	NA	NA	No	NA	NA	NA	NA
Gdist:S16554_8200	BT05	13.667	0.0068	NA	No	No	No	No	No	Yes (0.013)	No	NA	NA	No	NA	NA	NA	NA
LD:S183034_3756	BT05	13.667	-0.004	NA	No	No	No	No	No	No	No	NA	NA	No	NA	NA	NA	NA
cDNA:S405104_7335	BT05	15.595	0.0362	NA	No	No	No	No	No	Yes (0.022)	No	0.625060976	0.21798387	Yes	NA	NA	NA	NA
cDNA:S405104_1587	BT05	15.595	-0.0119	NA	No	No	No	No	No	Yes (0.048)	No	NA	NA	No	NA	NA	NA	NA
Gdist:S475070_6546	BT05	16.498	0.016	NA	No	No	No	No	No	No	No	NA	NA	No	NA	NA	NA	NA
Gdist:S85381_7267	BT05	16.529	0.0036	NA	No	No	No	No	No	No	No	NA	NA	No	NA	NA	NA	NA
Gdist:S85381_4939	BT05	16.559	0.0213	NA	No	No	No	No	No	No	No	NA	NA	No	NA	NA	NA	NA
cDNA:S293527_2798	BT05	16.595	0.0111	NA	No	No	No	No	No	No	No	NA	NA	No	NA	NA	NA	NA
Gdist:S95747_5341	BT05	17.672	0.0155	NA	No	No	No	No	No	No	No	NA	NA	No	NA	NA	NA	NA
Gdist:S204043_6591	BT05	17.672	0.0155	NA	No	No	No	No	No	No	No	NA	NA	No	NA	NA	NA	NA
SalHit:S119227_2435	BT05	17.673	0.0195	NA	No	No	No	No	No	No	No	NA	NA	No	NA	NA	NA	NA
SalHit:S119227_1244	BT05	17.714	0.0242	NA	No	No	No	No	No	No	No	NA	NA	No	NA	NA	NA	NA
Gdist:S96659_2257	BT05	17.769	0.0043	NA	No	No	No	No	No	No	No	NA	NA	No	NA	NA	NA	NA
Gdist:S44660_237	BT05	17.77	0.0092	NA	No	No	No	No	No	No	No	NA	NA	No	NA	NA	NA	NA
Gdist:S285036_8726	BT05	17.913	-0.007	NA	No	No	No	No	No	Yes (0.035)	No	NA	NA	No	NA	NA	NA	NA
Gdist:S497554_1269	BT05	19.704	0.0244	NA	No	No	No	No	No	No	No	NA	NA	No	NA	NA	NA	NA
Gdist:S497554_3191	BT05	19.705	0.0069	NA	No	No	No	No	No	No	No	NA	NA	No	NA	NA	NA	NA
Gdist:S239826_12936	BT05	19.71	0.0214	NA	No	No	No	No	No	No	No	NA	NA	No	NA	NA	NA	NA
cDNA:S21788_1182	BT05	19.872	0.0179	NA	No	No	No	No	No	No	No	NA	NA	No	NA	NA	NA	NA
Gdist:S540008_1839	BT05	19.913	0.0106	NA	No	No	No	No	No	No	No	NA	NA	No	NA	NA	NA	NA
SalHit:S167714_1334	BT05	23.359	0.0199	NA	No	No	No	No	No	No	No	NA	NA	No	NA	NA	NA	NA
cDNA:C124193644_487	BT05	25.489	0.01	NA	No	No	No	No	No	No	No	NA	NA	No	NA	NA	NA	NA
SalarSNP:ESTNV_24241_102	BT05	27.619	0.0104	NA	Yes	No	No	No	No	Yes (0) (*288 SNI	Yes (0.001) (*28	NA	NA	No	NA	NA	NA	NA
SalHit:S1027_8940	BT05	27.644	0.0595	NA	No	No	No	No	No	No	No	0.982691275	0.60417614	Yes	NA	NA	NA	NA
SalHit:S1027_7181	BT05	27.646	0.0057	NA	No	No	No	No	No	No	No	NA	NA	No	NA	NA	NA	NA
Gdist:S13164_5885	BT05	32.177	0.0127	NA	No	No	No	No	No	No	No	NA	NA	No	NA	NA	NA	NA
Gdist:S497709_1211	BT05	33.89	0.0024	NA	No	No	No	No	No	No	No	NA	NA	No	NA	NA	NA	NA
cDNA:S437669_209	BT05	33.974	0.0371	NA	No	No	No	No	No	No	No	0.916490895	0.79436364	Yes	NA	NA	NA	NA
SalHit:S159124_1208	BT05	37.126	0.002	NA	No	No	No	No	No	No	No	NA	NA	No	NA	NA	NA	NA
SalarSNP:BASS15_B7_G03_752	BT05	41.133	0.0066	NA	No	No	No	No	No	Yes (0)	Yes (0.009)	NA	NA	No	NA	NA	NA	NA
cDNA:S133625_906	BT05	41.972	0.0449	NA	No	No	No	No	No	No	No	0.910253165	0.40511084	Yes	NA	NA	NA	NA
cDNA:S569210_3034	BT05	44.81	0.0137	NA	No	No	No	No	No	No	No	NA	NA	No	NA	NA	NA	NA
cDNA:S569210_2498	BT05	44.81	0.0042	NA	No	No	No	No	No	No	No	NA	NA	No	NA	NA	NA	NA
Gdist:S64198_4811	BT05	44.81	-0.0002	NA	No	No	No	No	No	No	No	NA	NA	No	NA	NA	NA	NA
LD:S182363_10553	BT05	45.846	0.0517	NA	Yes	No	No	No	No	No	No	0.709659574	0.53701987	Yes	NA	NA	NA	NA
LD:S182363_1448	BT05	46.054	0.0259	NA	No	No	No	No	No	No	No	NA	NA	No	NA	NA	NA	NA
Gdist:S252406_9755	BT05	47.063	0.0197	NA	No	No	No	No	No	No	No	0.74387574	0.917662	Yes	NA	NA	NA	NA
cDNA:S357219_769	BT05	47.147	0.0386	NA	No	No	No	No	No	No	No	0.982691275	0.87844814	Yes	NA	NA	NA	NA
Gdist:S93404_10483	BT05	48.462	0.031	NA	No	No	No	No	No	Yes (0.001)	Yes (0.014)	0.365723684	0.33346154	Yes	NA	NA	NA	NA
Gdist:S167909_2010	BT05	48.482	0.0077	NA	No	No	No	No	No	No	No	NA	NA	No	NA	NA	NA	NA
Gdist:S90845_14730	BT05	48.516	0.0108	NA	No	No	No	No	No	No	No	NA	NA	No	NA	NA	NA	NA
Gdist:S17879_4184	BT05	49.097	0.0673	NA	No	No	No	No	No	Yes (0.002)	Yes (0.024)	0.965664557	0.6128453	Yes	NA	NA	NA	NA
Gdist:S156336_679	BT05	49.097	0.0519	NA	No	No	No	No	No	Yes (0.023)	No	0.978091922	0.98948864	Yes	NA	NA	NA	NA
Gdist:S218170_9435	BT05	49.097	0.0307	NA	No	No	No	No	No	No	No	0.916490895	0.92525912	Yes	NA	NA	NA	NA
Gdist:S51432_4537	BT05	49.097	0.0189	NA	No	No	No	No	No	Yes (0)	Yes (0.005)	0.9945	0.58291168	Yes	NA	NA	NA	NA
cDNA:S379086_6064	BT05	49.844	0.0201	NA	No	No	No	No	No	No	No	NA	NA	No	NA	NA	NA	NA
Gdist:S171660_5313	BT05	49.845	0.0101	NA	No	No	No	No	No	Yes (0.032)	No	NA	NA	No	NA	NA	NA	NA
Gdist:S171660_5453	BT05	50.419	0.0248	NA	No	No	No	No	No	Yes (0)	Yes (0.006)	NA	NA	No	NA	NA	NA	NA
Gdist:S54084_9356	BT05	50.557	0.0129	NA	No	No	No	No	No	Yes (0.012)	No	NA	NA	No	NA	NA	NA	NA
Gdist:S53108_2660	BT05	50.714	-0.0059	NA	No	No	No	No	No	No	No	NA	NA	No	NA	NA	NA	NA
Gdist:S564428_20142	BT05	50.841	0.0299	NA	Yes	No	No	No	No	No	No	NA	NA	No	NA	NA	NA	NA
Gdist:S153467_6701	BT05	50.967	0.0091	NA	No	No	No	No	No	No	No	NA	NA	No	NA	NA	NA	NA
Gdist:S268511_6812	BT05	51.564	0.0063	NA	No	No	No	No	No	No	No	NA	NA	No	NA	NA	NA	NA
Gdist:S100907_122	BT05	52	0.0129	NA	No	No	No	No	No	No	No	NA	NA	No	NA	NA	NA	NA
Gdist:S356774_6492	BT05	52.291	0.0198	NA	No	No	No	No	No	Yes (0.014)	No	NA	NA	No	NA	NA	NA	NA
cDNA:S764899_4758	BT05	52.325	0.0139	NA	No	No	No	No	No	No	No	NA	NA	No	NA	NA	NA	NA
cDNA:S13770_1805	BT05	52.477	0.0023	NA	No	No	No	No	No	No	No	NA	NA	No	NA	NA	NA	NA
Gdist:S126436_9807	BT05	53.428	-0.0083	NA	No	No	No	No	No	No	No	NA	NA	No	NA	NA	NA	NA
Gdist:S382649_11164	BT05	54.379	-0.0017	NA	No	No	No	No	No	No	No	NA	NA	No	NA	NA	NA	NA
Gdist:S96697_4610	BT05	57.272	0.0243	NA	No	No	No	No	No	No	No	NA	NA	No	NA	NA	NA	NA
Gdist:S3342_3400	BT05	59.143	-0.001	NA	No	No	No	No	No	No	No	NA	NA	No	NA	NA	NA	NA
SalHit:S54868_1385	BT05	61.015	0.029	NA	No	No	No	No	No	No	No	NA	NA	No	NA	NA	NA	NA
cDNA:S390903_1871	BT05	63.012	-0.0098	NA	No	No	No	No	No	No	No	NA	NA	No	NA	NA	NA	NA
cDNA:S14394_194	BT05	63.307	0.0247	NA	No	No	No	No	No	No	No	NA	NA	No	NA	NA	NA	NA
cDNA:S169620_2932	BT05	63.443	0.023	NA	No	No	No	No	No	No	No	NA	NA	No	NA	NA	NA	NA
SalHit:S34693_1686	BT05	67.123	-0.012	NA	No	No	No	No	No	No	No	NA	NA	No	NA	NA	NA	NA
Gdist:S543123_4907	BT05	67.123	0.064	PREDIC	No	No	No	No	No	No	No	0.016276596	0.94321023	Yes	VAR RED	Reduced	NA	yes
Gdist:S41465_3741	BT05	69.535	0.0292	NA	No	No	No	No	No	No	No	NA	NA	No	NA	NA	NA	NA
cDNA:S571530_2439	BT05	71.947	0.0089	NA	No	No	No	No	No	No	No	NA	NA	No	NA	NA	NA	NA
Gdist:S187440_8357	BT05	71.982	0.0203	NA	No	No	No	No	No	No	No	NA	NA	No	NA	NA	NA	NA
Gdist:S275374_7392	BT05	72.019	0.0283	NA	No	No	No	No	No	No	No	NA	NA	No	NA	NA	NA	NA
cDNA:S409498_652	BT05	72.028	0.0242	NA	Yes	No	No	No	No	No	No	NA	NA	No	NA	NA	NA	NA
SalHit:S597042_1961	BT05	72.028	0.0042	NA	No	No	No	No	No	Yes (0.009)	No	NA	NA	No	NA	NA	NA	NA
Gdist:S285956_7415	BT05	72.029	-0.0035	NA	No	No	No	No	No	No	No	NA	NA	No	NA	NA	NA	NA
Gdist:S225224_4710	BT05	77.952	-0.0061	NA	No	No	No	No	No	No	No	NA	NA	No	NA	NA	NA	NA
Gene:S93233_2108	BT05	79.8	-0.0017	NA	No	No	No	No	No	No	No	NA	NA	No	NA	NA	NA	NA
Gene:S830637_471	BT05	80.102	0.0248	NA	No	No	No	No	No	No	No	NA	NA	No	NA	NA	NA	NA
Gdist:S334317_2381	BT05	82.637	0.0394	NA	No	No	No	No	No	No	No	0.910253165	0.95466066	Yes	NA	NA	NA	NA
LD:S47306_2532	BT05	84.832	0.0284	NA	Yes	No	No	No	No	No	No	NA	NA	No	NA	NA	NA	NA
SalHit:S81886_3734	BT05	84.832	0.0175	NA	No	No	No	No	No	No	No	NA	NA	No	NA	NA	NA	NA
SalHit:S88367_11998	BT05	84.832	0.0127	NA	No	No	No	No	No	No	No	NA	NA	No	NA	NA	NA	NA
SalHit:S88367_9559	BT05	84.833	0.0369	NA	No	No	No	No	No	No	No	0.614833333	0.22871134	Yes	NA	NA	NA	NA
SalHit:S88367_2537	BT05	84.834	0.0249	NA	No	No	No	No	No	No	No	NA	NA	No	NA	NA	NA	NA
Gdist:S101675_4227	BT05	86.019	0.0302	NA	No	No	No	Yes (0.043)	No	No	No	0.916490895	0.17126866	Yes	NA	NA	NA	NA
cDNA:S529917_2353	BT05	88.773	0.0093	NA	No	No	No	No	No	No	No	NA	NA	No	NA	NA	NA	NA
SalHit:S42917_9951	BT05	88.773	0.0041	NA	No	No	No	No	No	No	No	NA	NA	No	NA	NA	NA	NA
cDNA:S20998_14238	BT05	88.773	0.0024	NA	No	No	No	No	No	No	No	NA	NA	No	NA	NA	NA	NA
cDNA:S20998_4758	BT05	88.773	-0.0106	NA	No	No	No	No	No	No	No	NA	NA	No	NA	NA	NA	NA
SalHit:S500022_1016	BT05	89.281	0.0096	NA	No	No	No	No	No	No	No	NA	NA	No	NA	NA	NA	NA
cDNA:S164512_3249	BT05	89.734	0.0515	NA	No	No	No	No	No	No	No	0.234364162	0.93365274	Yes	NA	NA	NA	NA
Gdist:S416816_5005	BT05	89.757	0.0437	NA	No	No	No	No	No	No	No	0.996605505	0.80916341	Yes	NA	NA	NA	NA
Gdist:S382672_9810	BT05	89.781	0.0278	NA	No	No	No	No	No	Yes (0.013)	No	NA	NA	No	NA	NA	NA	NA
Gdist:S382672_3438	BT05	89.781	0.0076	NA	No	No	No	No	No	No	No	NA	NA	No	NA	NA	NA	NA
cDNA:S105063_643	BT05	89.786	0.0073	NA	No	No	No	No	No	No	No	NA	NA	No	NA	NA	NA	NA
SalarSNP:ESTNV_16766_463	BT05	89.813	0.0444	NA	No	No	No	No	No	No	No	0.24225	0.53217391	Yes	NA	NA	NA	NA
Gdist:S34134_2418	BT05	94.312	0.0038	NA	No	No	No	No	No	No	No	NA	NA	No	NA	NA	NA	NA
cDNA:S283239_5129	BT05	94.317	0.0146	NA	No	No	No	No	No	No	No	NA	NA	No	NA	NA	NA	NA
Gdist:S71188_8148	BT05	96.871	0.0057	NA	Yes	No	No	No	No	No	No	NA	NA	No	NA	NA	NA	NA
cDNA:S524433_4324	BT05	97.428	0.0177	NA	No	No	No	No	No	No	No	NA	NA	No	NA	NA	NA	NA
SalHit:C123427156_80	BT05	97.454	0.0446	NA	No	No	No	No	No	No	No	0.654440559	0.7500974	Yes	NA	NA	NA	NA
SalHit:S318567_1701	BT05	97.736	0.02	NA	No	No	No	No	No	No	No	NA	NA	No	NA	NA	NA	NA
SalarSNP:ESTNV_35961_1479	BT06	0	0.0192	NA	No	No	No	No	No	No	No	NA	NA	No	NA	NA	NA	NA
SalHit:S171093_1659	BT06	0.133	-0.0035	NA	No	No	No	No	No	No	No	NA	NA	No	NA	NA	NA	NA
SalarSNP:GCR_cBin8345_Ctg1	BT06	0.145	0.0244	NA	No	No	No	No	No	No	No	NA	NA	No	NA	NA	NA	NA
SalarSNP:ESTV_20513_207	BT06	1.984	0.0328	NA	No	No	No	No	No	No	No	0.760560928	0.95466066	Yes	NA	NA	NA	NA
cDNA:S390597_2423	BT06	3.67	0.0402	NA	No	No	No	No	No	Yes (0)	Yes (0.008)	0.3193875	0.73792035	Yes	NA	NA	NA	NA
cDNA:S222300_2367	BT06	3.67	0.0109	NA	No	No	No	No	No	No	No	NA	NA	No	NA	NA	NA	NA
cDNA:S162820_857	BT06	5.678	0.0241	NA	No	No	No	No	No	No	No	NA	NA	No	NA	NA	NA	NA
SalHit:S600261_600	BT06	6.207	0.0036	NA	No	No	No	No	No	No	No	NA	NA	No	NA	NA	NA	NA
cDNA:S12068_2890	BT06	10.963	0.0065	NA	No	No	No	No	No	No	No	NA	NA	No	NA	NA	NA	NA
cDNA:S12068_320	BT06	10.989	0.0131	NA	No	No	No	No	No	No	No	NA	NA	No	NA	NA	NA	NA
cDNA:S347572_3504	BT06	11.005	0.0331	none	No	No	No	No	No	No	No	0	0.48116071	Yes	VAR RED	Reduced	NA	yes

cDNA:S206043_3642	BT06	13.117	0.0256	NA	No	No	No	No	No	No	No	NA	NA	No	NA	NA	NA	NA
Gdist:S233895_7485	BT06	13.12	0.0161	NA	No	No	No	No	No	No	No	NA	NA	No	NA	NA	NA	NA
cDNA:S456400_1829	BT06	13.12	0.0152	NA	No	No	No	No	No	No	No	NA	NA	No	NA	NA	NA	NA
cDNA:S467960_5741	BT06	13.153	0.0257	NA	No	No	No	No	No	No	No	NA	NA	No	NA	NA	NA	NA
SalarSNP:GCR_cBin44576_Ctg	BT06	22.429	0.009	NA	No	No	No	No	No	Yes (0)	Yes (0.001)	NA	NA	No	NA	NA	NA	NA
Gdist:S347238_9635	BT06	22.465	0.0294	NA	No	No	No	Yes (0.002)	Yes (0.007)	No	No	NA	NA	No	NA	NA	NA	NA
SalHit:S64808_2593	BT06	25.719	0.0215	NA	No	No	No	No	No	No	No	NA	NA	No	NA	NA	NA	NA
SalHit:S64808_1923	BT06	31.555	0.0182	NA	No	No	No	No	No	Yes (0.001)	Yes (0.014)	NA	NA	No	NA	NA	NA	NA
SalHit:S185934_458	BT06	37.391	0.0002	NA	No	No	No	No	No	No	No	NA	NA	No	NA	NA	NA	NA
SalHit:S176728_4365	BT06	41.687	-0.0083	NA	No	No	No	No	No	No	No	NA	NA	No	NA	NA	NA	NA
Gdist:S389681_7893	BT06	42.825	-0.0014	NA	No	No	No	No	No	No	No	NA	NA	No	NA	NA	NA	NA
Gdist:S421297_4945	BT06	43.416	0.0035	NA	No	No	No	No	No	No	No	NA	NA	No	NA	NA	NA	NA
Gdist:S30627_14083	BT06	45.858	0.0214	NA	No	No	No	No	No	No	No	NA	NA	No	NA	NA	NA	NA
Gdist:S2506_6823	BT06	46.945	-0.0008	NA	No	No	No	No	No	No	No	NA	NA	No	NA	NA	NA	NA
Gdist:S355641_2300	BT06	46.959	0.0174	NA	No	No	No	No	No	No	No	NA	NA	No	NA	NA	NA	NA
Gdist:S2506_996	BT06	46.976	0.0479	NA	No	No	No	No	No	No	No	0.279285714	0.39962687	Yes	NA	NA	NA	NA
cDNA:S436446_3416	BT06	47.074	0.0285	NA	No	No	No	No	No	No	No	NA	NA	No	NA	NA	NA	NA
cDNA:S102161_6750	BT06	47.095	0.0051	NA	No	No	No	No	No	No	No	NA	NA	No	NA	NA	NA	NA
cDNA:S102161_2082	BT06	47.445	0.0283	NA	No	No	No	No	No	Yes (0.012)	No	NA	NA	No	NA	NA	NA	NA
Gdist:S49171_6318	BT06	47.807	0.003	NA	No	No	No	No	No	No	No	NA	NA	No	NA	NA	NA	NA
Gdist:S164265_4478	BT06	49.957	0.0165	NA	No	No	No	No	No	Yes (0.02)	No	NA	NA	No	NA	NA	NA	NA
Gdist:S392774_8533	BT06	51.515	-0.0041	NA	No	No	No	No	No	Yes (0.007)	Yes (0.049)	NA	NA	No	NA	NA	NA	NA
SalarSNP:ESTV_15243_314	BT06	51.516	0.0705	NA	No	No	No	No	No	No	No	0.331625616	0.72269585	Yes	NA	NA	NA	NA
cDNA:S291008_2693	BT06	51.516	0.004	NA	No	No	No	No	No	No	No	NA	NA	No	NA	NA	NA	NA
cDNA:S291008_3424	BT06	51.516	-0.0015	NA	No	No	No	No	No	No	No	NA	NA	No	NA	NA	NA	NA
Gdist:S392774_2824	BT06	51.516	-0.0121	NA	Yes	No	No	No	No	No	No	NA	NA	No	NA	NA	NA	NA
Gdist:S262780_5400	BT06	55.326	0.0466	NA	No	No	No	No	No	Yes (0.048)	No	0.365723684	0.95466066	Yes	NA	NA	NA	NA
Gdist:S77763_6563	BT06	55.326	-0.0029	NA	No	No	No	No	No	No	No	NA	NA	No	NA	NA	NA	NA
Gdist:S234649_8466	BT06	55.576	-0.0088	NA	No	No	No	No	No	No	No	NA	NA	No	NA	NA	NA	NA
cDNA:S238020_10690	BT06	55.826	0.0201	NA	No	No	No	No	No	Yes (0.015)	No	NA	NA	No	NA	NA	NA	NA
Gdist:S150342_1884	BT06	55.875	0.0176	NA	No	No	No	No	No	No	No	NA	NA	No	NA	NA	NA	NA
cDNA:S540909_4091	BT06	55.962	-0.0081	NA	No	No	No	No	No	No	No	NA	NA	No	NA	NA	NA	NA
Gdist:S26639_1930	BT06	56.543	0.0041	NA	No	No	No	No	No	No	No	NA	NA	No	NA	NA	NA	NA
Gdist:S172474_5732	BT06	57.097	-0.006	NA	No	No	No	No	No	No	No	NA	NA	No	NA	NA	NA	NA
SalHit:S243803_4071	BT06	60.63	0.0436	NA	No	No	No	No	No	No	No	0.874749576	0.91619469	Yes	NA	NA	NA	NA
Gdist:S270724_5926	BT06	60.63	-0.0034	NA	No	No	No	No	No	No	No	NA	NA	No	NA	NA	NA	NA
Gdist:S154543_7222	BT06	60.631	0.0166	NA	Yes	No	No	No	No	No	No	NA	NA	No	NA	NA	NA	NA
Gdist:S330683_6886	BT06	62.109	-0.007	NA	No	No	No	No	No	No	No	NA	NA	No	NA	NA	NA	NA
Gdist:S330683_206	BT06	63.1	0.038	NA	No	No	No	No	No	No	No	0.817611524	0.33763889	Yes	NA	NA	NA	NA
Gdist:S38333_12201	BT06	64.395	0.008	NA	No	No	No	No	No	No	No	NA	NA	No	NA	NA	NA	NA
cDNA:S398101_2128	BT06	64.395	0.0064	NA	No	No	No	No	No	Yes (0.003)	Yes (0.027)	NA	NA	No	NA	NA	NA	NA
Gdist:S237988_1589	BT06	64.452	-0.0006	NA	No	No	No	No	No	No	No	NA	NA	No	NA	NA	NA	NA
Gdist:S366051_6089	BT06	67.701	-0.0044	NA	No	No	No	No	No	No	No	NA	NA	No	NA	NA	NA	NA
LD:S126259_12032	BT06	68.64	0.029	NA	No	No	No	No	No	No	No	NA	NA	No	NA	NA	NA	NA
LD:S126259_573	BT06	68.646	0.001	NA	Yes	No	No	No	No	No	No	NA	NA	No	NA	NA	NA	NA
cDNA:S130162_702	BT06	68.652	0.0751	NA	No	No	No	No	No	Yes (0.037)	No	0.297279793	0.8586601	Yes	NA	NA	NA	NA
cDNA:S43085_1432	BT06	68.652	0.0058	NA	No	No	No	No	No	Yes (0.007)	Yes (0.049)	NA	NA	No	NA	NA	NA	NA
cDNA:S234748_925	BT06	68.652	-0.0124	NA	Yes	No	No	No	No	No	No	NA	NA	No	NA	NA	NA	NA
Gdist:S206123_9204	BT06	68.658	0.0076	NA	No	No	No	No	No	No	No	NA	NA	No	NA	NA	NA	NA
Gdist:S598999_4368	BT06	71.135	-0.0001	NA	Yes	No	No	No	No	No	No	NA	NA	No	NA	NA	NA	NA
Gdist:S58534_5795	BT06	72.374	0.028	NA	No	No	No	No	No	No	No	0.275564516	0.84672504	Yes	NA	NA	NA	NA
Gdist:S13235_7808	BT06	72.374	0.0184	NA	Yes	No	No	No	No	No	No	NA	NA	No	NA	NA	NA	NA
Gdist:S151336_6037	BT06	73.027	0.0265	NA	No	No	No	No	No	No	No	NA	NA	No	NA	NA	NA	NA
Gdist:S151336_5896	BT06	73.478	-0.0063	NA	No	No	No	No	No	No	No	NA	NA	No	NA	NA	NA	NA
cDNA:S33235_315	BT06	73.479	0.027	NA	No	No	No	No	No	No	No	NA	NA	No	NA	NA	NA	NA
SalHit:S212497_8172	BT06	73.627	0.0038	NA	Yes	No	No	No	No	No	No	NA	NA	No	NA	NA	NA	NA
Gdist:S14505_7999	BT06	73.628	0.0058	NA	Yes	No	No	No	No	No	No	NA	NA	No	NA	NA	NA	NA
cDNA:S252705_4671	BT06	74.702	0.0199	NA	No	No	No	No	No	No	No	NA	NA	No	NA	NA	NA	NA
Gdist:S477153_7382	BT06	74.702	-0.0033	NA	No	No	No	No	No	No	No	NA	NA	No	NA	NA	NA	NA
cDNA:S252705_5472	BT06	74.702	-0.0051	NA	No	No	No	No	No	No	No	NA	NA	No	NA	NA	NA	NA
Gdist:S432837_1058	BT06	75.376	0.0548	NA	No	No	No	No	No	No	No	0.732797741	0.73333893	Yes	NA	NA	NA	NA
Gdist:S432837_1428	BT06	76.05	0.0241	NA	No	No	No	No	No	No	No	NA	NA	No	NA	NA	NA	NA
SalHit:S242729_7709	BT06	76.724	0.0084	NA	No	No	No	No	No	Yes (0.023)	No	NA	NA	No	NA	NA	NA	NA
Gdist:S290130_2222	BT06	76.764	0.0155	NA	No	No	No	No	No	No	No	NA	NA	No	NA	NA	NA	NA
Gdist:S78363_7410	BT06	76.764	0.0013	NA	No	No	No	No	No	No	No	NA	NA	No	NA	NA	NA	NA
Gdist:S78363_962	BT06	76.764	-0.0036	NA	No	No	No	No	No	No	No	NA	NA	No	NA	NA	NA	NA
Gdist:S344200_7042	BT06	76.764	-0.0123	NA	No	No	No	No	No	No	No	NA	NA	No	NA	NA	NA	NA
Gdist:S594889_7588	BT06	76.794	-0.0017	NA	No	No	No	No	No	No	No	NA	NA	No	NA	NA	NA	NA
Gdist:S594889_7885	BT06	77.432	0.0622	NA	No	No	No	No	No	No	No	0.505633562	0.71946429	Yes	NA	NA	NA	NA
SalHit:S49076_7001	BT06	77.432	0.0519	NA	No	No	No	No	No	No	No	0.361347926	0.69352273	Yes	NA	NA	NA	NA
SalHit:S49076_2139	BT06	77.432	0.0345	NA	No	No	No	No	No	No	No	0.97625523	0.77613306	Yes	NA	NA	NA	NA
SalHit:S49076_2694	BT06	77.432	0.0335	NA	No	No	No	No	No	No	No	0.754541016	0.78459529	Yes	NA	NA	NA	NA
SalHit:S49076_9602	BT06	77.432	0.0085	NA	No	No	No	No	No	No	No	NA	NA	No	NA	NA	NA	NA
Gdist:S113957_4095	BT06	77.432	-0.0069	NA	No	No	No	No	No	Yes (0)	Yes (0.006)	NA	NA	No	NA	NA	NA	NA
Gdist:S638094_1350	BT06	79.016	0.0074	NA	No	No	No	No	No	No	No	NA	NA	No	NA	NA	NA	NA
cDNA:S53928_6647	BT06	80.475	0.0489	NA	No	No	No	No	No	No	No	0.522028302	0.71946429	Yes	NA	NA	NA	NA
Gdist:S401841_17519	BT06	80.486	0.0391	NA	No	No	No	No	No	No	No	0.893131188	0.95357891	Yes	NA	NA	NA	NA
cDNA:S435837_8365	BT06	80.497	0.0213	NA	No	No	No	No	No	No	No	NA	NA	No	NA	NA	NA	NA
cDNA:S435837_6673	BT06	82.972	0.0232	NA	No	No	No	No	No	No	No	NA	NA	No	NA	NA	NA	NA
cDNA:S370001_9436	BT06	85.386	0.0301	NA	No	No	No	No	No	No	No	0.053873239	0.28089844	Yes	NA	NA	NA	NA
cDNA:S370001_11500	BT06	85.387	0.0155	NA	No	No	No	No	No	No	No	NA	NA	No	NA	NA	NA	NA
Gdist:S516108_8692	BT06	85.391	0.0121	NA	No	No	No	No	No	No	No	NA	NA	No	NA	NA	NA	NA
cDNA:S207226_5551	BT06	85.394	0.007	NA	No	No	No	No	No	No	No	NA	NA	No	NA	NA	NA	NA
Gdist:S341523_5552	BT06	88.677	0.0113	NA	No	No	No	No	No	No	No	NA	NA	No	NA	NA	NA	NA
Gdist:S350937_1703	BT06	90.318	0.0585	NA	No	No	No	No	No	No	No	0.911143868	0.84367021	Yes	NA	NA	NA	NA
Gdist:S141176_6740	BT06	90.458	0.0103	NA	No	No	No	No	No	No	No	NA	NA	No	NA	NA	NA	NA
Gdist:S475151_5761	BT06	91.2	-0.0033	NA	No	No	No	No	No	Yes (0.009)	No	NA	NA	No	NA	NA	NA	NA
Gdist:S89966_12304	BT06	91.224	0.0122	NA	No	No	No	No	No	No	No	NA	NA	No	NA	NA	NA	NA
LD:S30383_8588	BT06	91.224	-0.0003	NA	No	No	No	No	No	No	No	NA	NA	No	NA	NA	NA	NA
Gdist:S203343_7428	BT06	91.224	-0.0026	NA	No	No	No	No	No	Yes (0.002)	Yes (0.024)	NA	NA	No	NA	NA	NA	NA
cDNA:S336489_1653	BT06	92.837	0.0626	NA	No	No	No	No	No	Yes (0.002)	Yes (0.024)	0.375989362	0.93621951	Yes	NA	NA	NA	NA
Gdist:S260802_9469	BT06	92.837	0.0519	NA	Yes	No	No	No	No	Yes (0.023)	No	0.671982955	0.52391725	Yes	NA	NA	NA	NA
cDNA:S819025_669	BT06	92.837	0.0293	NA	No	No	No	No	No	Yes (0.037)	No	NA	NA	No	NA	NA	NA	NA
Gdist:S255467_6653	BT06	92.837	0.0869	NA	Yes	no	no	No	No	No	No	NA	NA	no	NA	NA	NA	NA
cDNA:S819025_2420	BT06	92.845	0.0703	NA	No	No	No	No	No	No	No	0.965664557	0.84677586	Yes	NA	NA	NA	NA
cDNA:S49912_673	BT06	94.125	0.0332	NA	No	No	No	No	No	Yes (0.004)	Yes (0.035)	0.933502203	0.80716535	Yes	NA	NA	NA	NA
cDNA:S49912_2049	BT06	94.154	0.0072	NA	No	No	No	No	No	No	No	NA	NA	No	NA	NA	NA	NA
cDNA:S49912_4653	BT06	94.181	0.0018	NA	No	No	No	No	No	Yes (0.013)	No	NA	NA	No	NA	NA	NA	NA
cDNA:S46008_1521	BT06	95.225	0.0048	NA	No	No	No	No	No	No	No	NA	NA	No	NA	NA	NA	NA
cDNA:S184823_3889	BT06	95.229	0.0286	NA	No	No	No	No	No	Yes (0.007)	No	NA	NA	No	NA	NA	NA	NA
cDNA:S184823_7256	BT06	95.234	0.0011	NA	No	No	No	No	No	No	No	NA	NA	No	NA	NA	NA	NA
Gdist:S31620_6458	BT06	95.416	0.0148	NA	No	No	No	No	No	Yes (0.009)	Yes (0.049)	NA	NA	No	NA	NA	NA	NA
cDNA:S249908_4794	BT06	95.598	0.0436	NA	No	No	No	No	No	Yes (0.006)	Yes (0.042)	0.603632813	0.72455172	Yes	NA	NA	NA	NA
cDNA:S249908_9625	BT06	95.598	0.0428	NA	No	No	No	No	No	Yes (0)	Yes (0.005)	0.558362069	0.3565678	Yes	NA	NA	NA	NA
Gdist:S101602_568	BT06	95.598	0.014	NA	No	No	No	No	No	No	No	NA	NA	No	NA	NA	NA	NA
Gdist:S9311_6504	BT06	95.598	0.0117	NA	No	No	No	No	No	No	No	NA	NA	No	NA	NA	NA	NA
cDNA:S36442_6427	BT06	95.598	0.0058	NA	No	No	No	No	No	No	No	NA	NA	No	NA	NA	NA	NA
Gdist:S453769_2029	BT06	95.599	0.0285	NA	No	No	No	No	No	Yes (0.002)	Yes (0.024)	NA	NA	No	NA	NA	NA	NA
Gdist:S453769_3795	BT06	95.6	0.0243	NA	No	No	No	No	No	Yes (0.003)	Yes (0.031)	NA	NA	No	NA	NA	NA	NA
Gdist:S37253_4936	BT06	96.525	-0.0013	NA	No	No	No	No	No	Yes (0)	Yes (0.001)	NA	NA	No	NA	NA	NA	NA
Gdist:S630971_2254	BT06	98.644	0.0182	NA	No	No	No	No	No	Yes (0.035)	No	NA	NA	No	NA	NA	NA	NA
SalarSNP:ESTNV_35796_168	BT06	98.822	0.0035	NA	No	No	No	No	No	No	No	NA	NA	No	NA	NA	NA	NA
Gene:S47271_2967	BT06	98.829	0.01	NA	No	No	No	No	No	No	No	NA	NA	No	NA	NA	NA	NA
Gene:S47271_1434	BT06	98.833	0.0141	NA	No	No	No	No	No	No	No	NA	NA	No	NA	NA	NA	NA
Gdist:S6441_5182	BT06	99.033	-0.0154	NA	No	No	No	No	No	No	No	NA	NA	No	NA	NA	NA	NA
SalarSNP:ESTNV_33260_818	BT06	99.266	0.0479	NA	No	No	No	No	No	No	No	0.365723684	0.84677586	Yes	NA	NA	NA	NA
Gdist:S167755_9411	BT06	99.508	0.0278	NA	No	No	No	No	No	No	No	NA	NA	No	NA	NA	NA	NA
cDNA:S467736_2080	BT06	99.508	0.0078	NA	No	No	No	No	No	No	No	NA	NA	No	NA	NA	NA	NA
cDNA:S467736_5425	BT06	99.509	0.0093	NA	No	No	No	No	No	No	No	NA	NA	No	NA	NA	NA	NA
cDNA:S467736_2414	BT06	99.51	0.0299	NA	No	No	No	No	No	No	No	NA	NA	No	NA	NA	NA	NA
cDNA:S35007_1727	BT06	99.511	0.0031	NA	No	No	No	No	No	No	No	NA	NA	No	NA	NA	NA	NA
cDNA:S18670_5330	BT06	99.511	0.0883	Salmo tr	No	Yes	No	No	No	No	No	0.614833333	0.54853698	Yes	Outlier Varde	Outlier Var	NA	yes
Gdist:S106111_3946	BT06	99.526	0.0085	NA	No	No	No	No	No	No	No	NA	NA	No	NA	NA	NA	NA
cDNA:S80843_7912	BT06	100.063	-0.0037	NA	No	No	No	No	No	No	No	NA	NA	No	NA	NA	NA	NA
Gdist:S22292_9134	BT06	100.332	0.0505	NA	No	No	No	No	No	No	No	0.463172727	0.61532609	Yes	NA	NA	NA	NA
Gdist:S365944_2332	BT06	101.726	0.0093	NA	No	No	No	No	No	No	No	NA	NA	No	NA	NA	NA	NA
Gdist:S297769_5036	BT06	101.82	0.0243	NA	No	No	No	No	No	No	No	NA	NA	No	NA	NA	NA	NA

Gdist:S120321_5469	BT06	101.897	0.0523	NA	No	No	No	No	No	No	No	0.578414634	0.46152893	Yes	NA	NA	NA	NA
Gdist:S241514_3572	BT06	101.897	0.0249	NA	No	No	No	No	No	No	No	NA	NA	No	NA	NA	NA	NA
Gdist:S120321_14257	BT06	101.897	0.0152	NA	No	No	No	No	No	No	No	NA	NA	No	NA	NA	NA	NA
Gdist:S397128_4077	BT06	101.906	0.0233	NA	No	No	No	No	No	No	No	NA	NA	No	NA	NA	NA	NA
Gdist:S11737_8352	BT06	101.926	0.008	NA	Yes	No	No	No	No	No	No	NA	NA	No	NA	NA	NA	NA
SalHit:S199057_2374	BT06	101.936	0.0059	NA	Yes	No	No	No	No	Yes (0.003)	Yes (0.03)	0.729436983	0.87029851	Yes	NA	NA	NA	NA
SalHit:S839660_1318	BT06	103.38	0.0003	NA	No	No	No	No	No	No	No	NA	NA	No	NA	NA	NA	NA
Gene:S667291_1079	BT06	103.38	0	NA	No	No	No	No	No	No	No	NA	NA	No	NA	NA	NA	NA
Gdist:S38014_11146	BT06	103.875	0.0262	NA	No	No	No	No	No	No	No	NA	NA	No	NA	NA	NA	NA
Gdist:S509178_1219	BT06	103.875	0.002	NA	No	No	No	No	No	No	No	NA	NA	No	NA	NA	NA	NA
cDNA:S837797_2394	BT06	103.875	0.0017	NA	No	No	No	No	No	No	No	NA	NA	No	NA	NA	NA	NA
Gdist:S58888_4016	BT06	103.875	0.0015	NA	No	No	No	No	No	No	No	NA	NA	No	NA	NA	NA	NA
Gdist:S835817_646	BT06	103.875	-0.0076	NA	No	No	No	No	No	No	No	NA	NA	No	NA	NA	NA	NA
cDNA:S837797_363	BT06	103.875	-0.0099	NA	No	No	No	No	No	No	No	NA	NA	No	NA	NA	NA	NA
Gdist:S329816_5156	BT06	103.978	0.0066	NA	No	No	No	No	No	No	No	NA	NA	No	NA	NA	NA	NA
Gdist:S411846_4216	BT06	104.114	-0.0005	NA	No	No	No	No	No	No	No	NA	NA	No	NA	NA	NA	NA
cDNA:S404555_12194	BT06	104.251	0.051	NA	No	No	No	No	No	Yes (0.004)	Yes (0.035)	0.614833333	0.76335837	Yes	NA	NA	NA	NA
cDNA:S404555_7877	BT06	104.251	0.0223	NA	No	No	No	No	No	No	No	NA	NA	No	NA	NA	NA	NA
SalHit:S151563_1728	BT06	104.251	0.0208	NA	No	No	No	No	No	Yes (0)	Yes (0.006)	NA	NA	No	NA	NA	NA	NA
SalHit:S706284_4420	BT06	104.251	0.0001	NA	No	No	No	No	No	Yes (0.006)	Yes (0.042)	NA	NA	No	NA	NA	NA	NA
cDNA:S304833_1946	BT06	104.251	-0.0009	NA	Yes	No	No	No	No	Yes (0.013)	No	NA	NA	No	NA	NA	NA	NA
cDNA:S678913_2022	BT06	104.251	-0.0033	NA	No	No	No	No	No	No	No	NA	NA	No	NA	NA	NA	NA
Gdist:S202467_7454	BT06	104.252	0.0351	NA	Yes	No	No	No	No	No	No	0.692970779	0.50291667	Yes	NA	NA	NA	NA
Gdist:S202467_5791	BT06	104.252	0.0343	NA	No	No	No	No	No	No	No	0.101479592	0.82025797	Yes	NA	NA	NA	NA
Gdist:S92508_518	BT06	104.256	0.042	NA	No	No	No	No	No	No	No	0.893131188	0.84677586	Yes	NA	NA	NA	NA
Gdist:S431843_874	BT07	0	0.0312	NA	No	No	No	No	No	No	No	0.593534483	0.53217391	Yes	NA	NA	NA	NA
cDNA:S835857_600	BT07	2.521	0.0003	NA	No	No	No	No	No	Yes (0.023)	No	NA	NA	No	NA	NA	NA	NA
cDNA:S608598_1333	BT07	2.721	0.0017	NA	No	No	No	No	No	No	No	NA	NA	No	NA	NA	NA	NA
Gdist:S146747_4637	BT07	2.724	0.0216	NA	No	No	No	No	No	No	No	NA	NA	No	NA	NA	NA	NA
Gdist:S210047_7592	BT07	3.491	0.0013	NA	No	No	No	No	No	No	No	NA	NA	No	NA	NA	NA	NA
Gdist:S163033_16042	BT07	7.8	-0.0132	NA	No	No	No	No	No	No	No	NA	NA	No	NA	NA	NA	NA
Gdist:S163033_15169	BT07	8.835	-0.0071	NA	Yes	No	No	No	No	No	No	NA	NA	No	NA	NA	NA	NA
SalHit:S744526_2175	BT07	8.845	0.0008	NA	No	No	No	No	No	No	No	NA	NA	No	NA	NA	NA	NA
cDNA:S26296_3384	BT07	9.525	0.0159	NA	No	No	No	No	No	No	No	0.874285714	0.80916341	Yes	NA	NA	NA	NA
Gdist:S407720_904	BT07	9.525	-0.0078	NA	No	No	No	No	No	No	No	NA	NA	No	NA	NA	NA	NA
cDNA:C124205688_219	BT07	9.528	-0.0108	NA	No	No	No	No	No	No	No	NA	NA	No	NA	NA	NA	NA
Gdist:S227855_2687	BT07	10.506	0.0156	NA	No	No	No	No	No	No	No	NA	NA	No	NA	NA	NA	NA
Gdist:S106843_3060	BT07	11.485	0.0314	NA	No	No	No	No	No	No	No	0.492734375	0.6958849	Yes	NA	NA	NA	NA
SalHit:S618840_2758	BT07	11.485	-0.0086	NA	No	No	No	No	No	No	No	NA	NA	No	NA	NA	NA	NA
cDNA:S273631_2250	BT07	11.985	0.0307	NA	No	No	No	No	No	No	No	0.233502907	0.46026971	Yes	NA	NA	NA	NA
cDNA:S273631_3441	BT07	11.988	0.0052	NA	Yes	No	No	No	No	No	No	NA	NA	No	NA	NA	NA	NA
cDNA:S132167_1829	BT07	11.991	-0.0012	NA	No	No	No	No	No	No	No	NA	NA	No	NA	NA	NA	NA
Gdist:S164621_6398	BT07	12.085	0.006	NA	No	No	No	No	No	No	No	NA	NA	No	NA	NA	NA	NA
Gdist:S149227_4245	BT07	12.171	0.0014	NA	No	No	No	No	No	No	No	NA	NA	No	NA	NA	NA	NA
Gdist:S10222_9302	BT07	14.534	0.0014	NA	No	No	No	No	No	No	No	NA	NA	No	NA	NA	NA	NA
Gdist:S377267_10717	BT07	15.388	0.0047	NA	No	No	No	No	No	No	No	NA	NA	No	NA	NA	NA	NA
Gdist:S123418_7093	BT07	17.94	-0.0029	NA	No	No	No	No	No	No	No	NA	NA	No	NA	NA	NA	NA
SalHit:S228814_2074	BT07	18.011	-0.0005	NA	No	No	No	No	No	No	No	NA	NA	No	NA	NA	NA	NA
Gdist:S3809_6423	BT07	20.648	0.0129	NA	No	No	No	No	No	No	No	NA	NA	No	NA	NA	NA	NA
Gdist:S634819_2294	BT07	20.65	0.0039	NA	No	No	No	No	No	No	No	NA	NA	No	NA	NA	NA	NA
Gdist:S218156_15069	BT07	20.783	-0.0011	NA	No	No	No	No	No	No	No	NA	NA	No	NA	NA	NA	NA
Gdist:S105127_8748	BT07	21.195	0.0184	NA	No	No	No	No	No	No	No	NA	NA	No	NA	NA	NA	NA
Gdist:S363218_972	BT07	21.591	0.0126	NA	No	No	No	No	No	No	No	NA	NA	No	NA	NA	NA	NA
Gdist:S391822_1236	BT07	21.591	0.0369	NA	No	No	No	No	No	No	No	0.0478125	0.26743902	Yes	VAR INC	increased	NA	yes
Gdist:S391822_5338	BT07	21.616	-0.007	NA	No	No	No	No	No	No	No	NA	NA	No	NA	NA	NA	NA
Gdist:S64322_9728	BT07	21.819	-0.0033	NA	No	No	No	No	No	No	No	NA	NA	No	NA	NA	NA	NA
cDNA:S84686_2112	BT07	23.322	0.028	NA	No	No	No	No	No	No	No	0.992172619	0.88385252	Yes	NA	NA	NA	NA
SalHit:S11514_2858	BT07	26.255	0.0086	NA	No	No	No	No	No	No	No	NA	NA	No	NA	NA	NA	NA
Gdist:S449182_5359	BT07	45.741	0.0059	NA	No	No	No	No	No	No	No	NA	NA	No	NA	NA	NA	NA
cDNA:C124250102_412	BT07	47.849	0.0401	NA	No	No	No	No	No	No	No	0.735636364	0.56773585	Yes	NA	NA	NA	NA
SalHit:S53219_2845	BT07	47.849	-0.0034	NA	No	No	No	No	No	No	No	NA	NA	No	NA	NA	NA	NA
cDNA:S621834_5067	BT07	47.849	0.0302	NA	No	No	No	No	No	Yes (0.001)	Yes (0.016)	0.845991993	0.04098214	Yes	SKJ INC	NA	increased	NA
Gdist:S298727_3047	BT07	52.656	0.0104	NA	No	No	No	No	No	No	No	NA	NA	No	NA	NA	NA	NA
Gdist:S158675_6988	BT07	52.656	0.0063	NA	Yes	No	No	No	No	No	No	NA	NA	No	NA	NA	NA	NA
SalHit:S10880_1341	BT07	53.097	0.0011	NA	No	No	No	No	No	No	No	NA	NA	No	NA	NA	NA	NA
Gdist:S546605_603	BT07	53.596	0.0034	NA	No	No	No	No	No	Yes (0.039)	No	NA	NA	No	NA	NA	NA	NA
Gdist:S241851_1802	BT07	53.597	-0.0079	NA	No	No	No	No	No	No	No	NA	NA	No	NA	NA	NA	NA
Gdist:S51135_6606	BT07	54.695	0.0202	NA	No	No	No	No	No	No	No	NA	NA	No	NA	NA	NA	NA
cDNA:S479522_1912	BT07	54.695	0.0067	NA	No	No	No	No	No	No	No	NA	NA	No	NA	NA	NA	NA
Gdist:S51135_6671	BT07	54.698	0.0173	NA	No	No	No	No	No	No	No	NA	NA	No	NA	NA	NA	NA
SalHit:S609634_1036	BT07	60.2	0.0615	NA	No	No	No	No	No	No	No	0.698909287	0.72269585	Yes	NA	NA	NA	NA
Gene:S282703_1456	BT07	60.2	0.0405	NA	No	No	No	No	No	No	No	0.614833333	0.88194268	Yes	NA	NA	NA	NA
Gdist:S75770_7431	BT07	60.2	0.0335	NA	No	No	No	No	No	No	No	0.916490895	0.40511084	Yes	NA	NA	NA	NA
LD:S139051_1298	BT07	60.2	0.0288	NA	No	No	No	No	No	No	No	NA	NA	No	NA	NA	NA	NA
cDNA:S35126_3369	BT07	60.2	0.0267	NA	No	No	No	No	No	No	No	NA	NA	No	NA	NA	NA	NA
Gdist:S119548_3098	BT07	60.2	0.0134	NA	No	No	No	No	No	No	No	NA	NA	No	NA	NA	NA	NA
cDNA:S268348_2100	BT07	60.2	0.007	NA	No	No	No	No	No	No	No	NA	NA	No	NA	NA	NA	NA
Gdist:S253708_7891	BT07	60.2	0.0012	NA	No	No	No	No	No	No	No	NA	NA	No	NA	NA	NA	NA
SalHit:S183101_2595	BT07	60.2	-0.0002	NA	No	No	No	No	No	No	No	NA	NA	No	NA	NA	NA	NA
Gdist:S221182_6229	BT07	60.2	-0.0081	NA	No	No	No	No	No	No	No	NA	NA	No	NA	NA	NA	NA
SalarSNP:GCR_cBin8189_Ctg1	BT07	60.2	-0.0105	NA	No	No	No	No	No	No	No	NA	NA	No	NA	NA	NA	NA
Gdist:S98235_3484	BT07	60.67	0.0129	NA	No	No	No	No	No	No	No	NA	NA	No	NA	NA	NA	NA
Gdist:S146211_5690	BT07	61.14	0.0222	NA	No	No	No	No	No	Yes (0.022)	No	NA	NA	No	NA	NA	NA	NA
Gdist:S273624_1168	BT07	63.032	0.0134	NA	No	No	No	No	No	No	No	NA	NA	No	NA	NA	NA	NA
Gdist:S273624_8437	BT07	63.035	0.0122	NA	Yes	No	No	No	No	No	No	NA	NA	No	NA	NA	NA	NA
Gdist:S40530_5594	BT07	65.371	0.0107	NA	No	No	No	No	No	No	No	NA	NA	No	NA	NA	NA	NA
Gdist:S19398_5530	BT07	67.884	0.0025	NA	No	No	No	No	No	No	No	NA	NA	No	NA	NA	NA	NA
SalHit:S447810_3696	BT07	70.397	0.0103	NA	No	No	No	No	No	Yes (0.004)	Yes (0.035)	NA	NA	No	NA	NA	NA	NA
cDNA:S572204_1064	BT07	73.591	0.0101	NA	No	No	No	No	No	No	No	NA	NA	No	NA	NA	NA	NA
SalHit:S253707_4432	BT07	73.656	-0.0058	NA	No	No	No	No	No	Yes (0.037)	No	NA	NA	No	NA	NA	NA	NA
SalHit:S191379_6348	BT07	73.717	0.019	NA	No	No	No	No	No	No	No	NA	NA	No	NA	NA	NA	NA
cDNA:S427654_5846	BT07	73.779	-0.0083	NA	No	No	No	No	No	No	No	NA	NA	No	NA	NA	NA	NA
cDNA:S21404_1309	BT07	77.495	0.0193	NA	No	No	No	No	No	No	No	NA	NA	No	NA	NA	NA	NA
SalHit:S720489_1453	BT07	86.473	0.0154	NA	No	No	No	No	No	No	No	NA	NA	No	NA	NA	NA	NA
Gdist:S178183_1939	BT07	88.92	0.0091	NA	No	No	No	No	No	No	No	NA	NA	No	NA	NA	NA	NA
Gdist:S521908_3793	BT07	89.143	0.0321	NA	No	No	No	No	No	No	No	0.902675081	0.67788613	Yes	NA	NA	NA	NA
Gdist:S91163_7567	BT07	89.143	0.0239	NA	No	No	No	No	No	No	No	NA	NA	No	NA	NA	NA	NA
cDNA:S92500_6012	BT07	89.214	0.0055	NA	No	No	No	No	No	No	No	NA	NA	No	NA	NA	NA	NA
cDNA:S59200_2354	BT07	92.136	0.0027	NA	No	No	No	No	No	No	No	NA	NA	No	NA	NA	NA	NA
Gene:S778345_741	BT07	93.609	0.0072	NA	No	No	No	No	No	No	No	NA	NA	No	NA	NA	NA	NA
SalarSNP:ESTNV_27243_587	BT07	95.082	-0.0111	NA	No	No	No	No	No	No	No	NA	NA	No	NA	NA	NA	NA
Gdist:S130688_5646	BT07	95.425	0.0065	NA	No	No	No	No	No	No	No	NA	NA	No	NA	NA	NA	NA
SalHit:S142292_1584	BT07	98.712	0.0643	NA	No	No	No	No	No	No	No	0.743034653	0.4815	Yes	NA	NA	NA	NA
SalHit:S594047_1310	BT07	104.243	-0.0025	NA	No	No	No	No	No	No	No	NA	NA	No	NA	NA	NA	NA
cDNA:S140351_5419	BT07	126.605	0.029	NA	No	No	No	No	No	Yes (0.023)	No	NA	NA	No	NA	NA	NA	NA
Gdist:S245527_5778	BT08	0	0.004	NA	No	No	No	No	No	No	No	NA	NA	No	NA	NA	NA	NA
cDNA:C124068305_390	BT08	1.149	0.0002	NA	No	No	No	No	No	No	No	NA	NA	No	NA	NA	NA	NA
Gdist:S9805_10765	BT08	2.96	0.0129	NA	No	No	No	No	No	No	No	NA	NA	No	NA	NA	NA	NA
Gdist:S50184_2171	BT08	3.522	0.04	NA	No	No	No	No	No	No	No	0.365723684	0.52134342	Yes	NA	NA	NA	NA
SalHit:S109150_1254	BT08	3.541	-0.0006	NA	No	No	No	No	No	No	No	NA	NA	No	NA	NA	NA	NA
Gene:S308708_3208	BT08	3.677	-0.01	NA	Yes	No	No	No	No	No	No	NA	NA	No	NA	NA	NA	NA
cDNA:S361757_4124	BT08	3.794	-0.0042	NA	No	No	No	No	No	No	No	NA	NA	No	NA	NA	NA	NA
cDNA:S565196_1646	BT08	12.675	0.0062	NA	No	No	No	No	No	No	No	NA	NA	No	NA	NA	NA	NA
cDNA:S343808_2594	BT08	17.879	-0.0102	NA	No	No	No	No	No	No	No	NA	NA	No	NA	NA	NA	NA
SalHit:S653212_179	BT08	18.051	-0.0064	NA	No	No	No	No	No	No	No	NA	NA	No	NA	NA	NA	NA
SalHit:S653212_1140	BT08	18.224	0.0103	NA	No	No	No	No	No	No	No	NA	NA	No	NA	NA	NA	NA
cDNA:S196928_1342	BT08	23.564	0.0097	NA	No	No	No	No	No	No	No	NA	NA	No	NA	NA	NA	NA
SalarSNP:ESTNV_30477_550	BT08	24.904	0.0237	NA	No	No	No	No	No	No	No	NA	NA	No	NA	NA	NA	NA
cDNA:S541784_2217	BT09	0	-0.0018	NA	No	No	No	No	No	No	No	NA	NA	No	NA	NA	NA	NA
Gene:S712373_1350	BT09	0.07	0.0264	NA	No	No	No	No	No	Yes (0.048)	No	NA	NA	No	NA	NA	NA	NA
Gdist:S180470_7370	BT09	0.07	0.0085	NA	No	No	No	No	No	Yes (0.044)	No	NA	NA	No	NA	NA	NA	NA
Gene:S712373_1710	BT09	0.07	0.0019	NA	No	No	No	No	No	Yes (0.002)	Yes (0.024)	NA	NA	No	NA	NA	NA	NA
Gdist:S46671_7362	BT09	0.07	-0.0054	NA	No	No	No	No	No	No	No	NA	NA	No	NA	NA	NA	NA
Gdist:S232726_6227	BT09	0.07	-0.0093	NA	No	No	No	No	No	No	No	NA	NA	No	NA	NA	NA	NA
Gdist:S9769_694	BT09	0.071	-0.0023	NA	No	No	No	No	No	No	No	NA	NA	No	NA	NA	NA	NA
SalHit:S396585_2965	BT09	0.08	-0.0031	NA	Yes	No	No	No	No	No	No	NA	NA	No	NA	NA	NA	NA
SalHit:S74979_874	BT09	2.615	0.009	NA	No	No	No	No	No	No	No	NA	NA	No	NA	NA	NA	NA

Gdist:S49720_507	BT09	2.616	0.0234	NA	No	No	No	No	No	No	No	NA	NA	No	NA	NA	NA	NA
SalHit:S626715_3624	BT09	2.616	0.0053	NA	No	No	No	No	No	No	No	NA	NA	No	NA	NA	NA	NA
Gdist:S669911_6878	BT09	2.616	0.0026	NA	No	No	No	No	No	No	No	NA	NA	No	NA	NA	NA	NA
Gdist:S669911_6641	BT09	2.616	0.0022	NA	No	No	No	No	No	No	No	NA	NA	No	NA	NA	NA	NA
Gdist:S114561_7994	BT09	3.464	0.028	NA	No	No	No	No	No	Yes (0.019)	No	NA	NA	No	NA	NA	NA	NA
SalHit:S217153_2544	BT09	3.464	0.0152	NA	No	No	No	No	No	No	No	NA	NA	No	NA	NA	NA	NA
Gdist:S201016_6814	BT09	3.464	0.0121	NA	No	No	No	No	No	Yes (0.001)	Yes (0.009)	NA	NA	No	NA	NA	NA	NA
Gdist:S467860_477	BT09	3.464	0.0096	NA	No	No	No	No	No	No	No	NA	NA	No	NA	NA	NA	NA
SalHit:S28329_3550	BT09	3.464	0.0078	NA	No	No	No	No	No	Yes (0.007)	No	NA	NA	No	NA	NA	NA	NA
Gene:S129840_2011	BT09	3.464	0.0064	NA	No	No	No	No	No	No	No	NA	NA	No	NA	NA	NA	NA
Gdist:S283249_208	BT09	3.464	0.0028	NA	No	No	No	No	No	No	No	NA	NA	No	NA	NA	NA	NA
SalHit:S245575_1093	BT09	3.464	-0.0023	NA	No	No	No	No	No	No	No	NA	NA	No	NA	NA	NA	NA
Gdist:S318976_2335	BT09	3.464	-0.014	NA	No	No	No	No	No	No	No	NA	NA	No	NA	NA	NA	NA
Gdist:S36381_5548	BT09	3.466	0.0112	NA	No	No	No	No	No	Yes (0)	Yes (0.003)	NA	NA	No	NA	NA	NA	NA
Gdist:S36381_5657	BT09	3.468	0.0102	NA	No	No	No	No	No	Yes (0)	Yes (0.004)	NA	NA	No	NA	NA	NA	NA
cDNA:S249523_196	BT09	4.775	-0.0038	NA	No	No	No	No	No	No	No	NA	NA	No	NA	NA	NA	NA
SalHit:S205437_11417	BT09	7.389	0.0371	NA	Yes	No	No	No	No	No	No	0.776679389	0.77227801	Yes	NA	NA	NA	NA
Gdist:S87765_2645	BT09	14.969	0.0591	none	No	No	No	No	No	No	No	0.038898305	0.49909851	Yes	VAR RED	Reduced	NA	yes
Gdist:S113093_3207	BT09	16.203	0.0203	NA	No	No	No	No	No	No	No	NA	NA	No	NA	NA	NA	NA
Gdist:S20056_5467	BT09	16.258	0.0244	NA	No	No	No	No	No	No	No	NA	NA	No	NA	NA	NA	NA
Gdist:S190364_6817	BT09	17.603	0.0099	NA	No	No	No	No	No	No	No	0.926563433	0.92372067	Yes	NA	NA	NA	NA
cDNA:S638202_1141	BT09	20.703	0.0341	NA	No	No	No	No	No	No	No	0.836015625	0.53217391	Yes	NA	NA	NA	NA
SalHit:S699749_1912	BT09	20.912	0.01	NA	No	No	No	No	No	No	No	NA	NA	No	NA	NA	NA	NA
SalHit:S440663_362	BT09	21.532	0.0011	NA	No	No	No	No	No	Yes (0.032)	No	NA	NA	No	NA	NA	NA	NA
SalHit:S98604_475	BT09	21.713	0.0134	NA	No	No	No	No	No	No	No	NA	NA	No	NA	NA	NA	NA
cDNA:S211903_5325	BT09	25.463	-0.0039	NA	No	No	No	No	No	No	No	NA	NA	No	NA	NA	NA	NA
Gdist:S11806_2740	BT09	25.488	0.0297	NA	No	No	No	No	No	No	No	NA	NA	No	NA	NA	NA	NA
Gdist:S11806_3970	BT09	25.488	0.018	NA	No	No	No	No	No	No	No	NA	NA	No	NA	NA	NA	NA
Gdist:S73922_1114	BT09	25.489	0.0111	NA	No	No	No	No	No	No	No	NA	NA	No	NA	NA	NA	NA
Gdist:S383195_199	BT09	26.119	0.0062	NA	No	No	No	No	No	No	No	NA	NA	No	NA	NA	NA	NA
Gdist:S14395_8283	BT09	27.488	0.0207	NA	No	No	No	No	No	No	No	NA	NA	No	NA	NA	NA	NA
Gdist:S341583_8089	BT09	28.959	0.0074	NA	No	No	No	No	No	No	No	NA	NA	No	NA	NA	NA	NA
SalHit:S452627_2679	BT09	31.38	0.0267	NA	No	No	No	No	No	No	No	0.574795082	0.21798387	Yes	NA	NA	NA	NA
SalarSNP:GCR_cBin43704_Ctg	BT09	36.96	0.0073	NA	No	No	No	No	No	No	No	NA	NA	No	NA	NA	NA	NA
Gdist:S171839_7107	BT09	36.96	-0.0046	NA	No	No	No	No	No	No	No	NA	NA	No	NA	NA	NA	NA
SalarSNP:GCR_cBin18774_Ctg	BT09	37.68	-0.0013	NA	No	No	No	No	No	No	No	NA	NA	No	NA	NA	NA	NA
Gdist:S61132_7845	BT09	43.999	-0.0072	NA	No	No	No	No	No	No	No	NA	NA	No	NA	NA	NA	NA
cDNA:S333649_178	BT09	47.928	0.0218	NA	No	No	No	No	No	No	No	NA	NA	No	NA	NA	NA	NA
SalarSNP:GCR_cBin4749_Ctg1	BT09	48.668	0.0178	NA	No	No	No	No	No	No	No	NA	NA	No	NA	NA	NA	NA
Gdist:S315826_2961	BT09	51.924	0.0272	NA	No	No	No	No	No	No	No	NA	NA	No	NA	NA	NA	NA
Gdist:S655608_6910	BT09	51.947	0.0314	NA	No	No	No	No	No	No	No	0.6875	0.74151316	Yes	NA	NA	NA	NA
Gdist:S332185_8167	BT09	51.947	0.0224	NA	No	No	No	No	No	No	No	NA	NA	No	NA	NA	NA	NA
SalarSNP:ESTNV_30052_103	BT09	51.948	0.0116	NA	No	No	No	No	No	No	No	NA	NA	No	NA	NA	NA	NA
Gdist:S26010_4642	BT09	51.948	0.0021	NA	No	No	No	No	No	Yes (0.013)	No	NA	NA	No	NA	NA	NA	NA
cDNA:S472595_1354	BT09	51.948	0.0005	NA	No	No	No	No	No	No	No	NA	NA	No	NA	NA	NA	NA
SalHit:S492378_7434	BT09	51.949	0.062	NA	No	No	No	No	No	No	No	0.975776224	0.9985	Yes	NA	NA	NA	NA
Gene:S171637_2011	BT09	51.949	0.0186	NA	No	No	No	No	No	Yes (0.013)	No	NA	NA	No	NA	NA	NA	NA
Gene:S171637_7022	BT09	51.949	0.0162	NA	No	No	No	No	No	No	No	NA	NA	No	NA	NA	NA	NA
Gdist:S31367_2663	BT09	51.949	0.0133	NA	No	No	No	No	No	No	No	NA	NA	No	NA	NA	NA	NA
Gdist:S116080_1571	BT09	51.949	0.0074	NA	No	No	No	No	No	No	No	NA	NA	No	NA	NA	NA	NA
Gene:S171637_4433	BT09	51.949	-0.0032	NA	No	No	No	No	No	No	No	NA	NA	No	NA	NA	NA	NA
cDNA:S212066_3949	BT09	51.949	-0.0055	NA	No	No	No	No	No	No	No	NA	NA	No	NA	NA	NA	NA
SalHit:S492378_5464	BT09	51.949	0.0502	PREDIC	No	No	No	No	No	No	No	0.519280063	0	Yes	SKJ RED	NA	reduced	NA
Gdist:S111722_10937	BT09	52.797	-0.0101	NA	No	No	No	No	No	No	No	NA	NA	No	NA	NA	NA	NA
cDNA:S337711_8313	BT09	53.284	0.0066	NA	Yes	No	No	No	No	No	No	NA	NA	No	NA	NA	NA	NA
Gene:S268762_268	BT09	53.769	0.0287	NA	No	No	No	No	No	No	No	NA	NA	No	NA	NA	NA	NA
Gdist:S253806_1210	BT09	53.799	0.0427	NA	No	No	No	No	No	No	No	0.299849246	0.89481818	Yes	NA	NA	NA	NA
Gdist:S82400_4842	BT09	54.104	0.0634	NA	No	No	No	No	No	Yes (0.012)	No	0.769430502	0.53851974	Yes	NA	NA	NA	NA
Gdist:S60782_1920	BT09	54.425	0.0822	NA	No	No	No	No	No	Yes (0.007)	Yes (0.049)	0.907678571	0.79584677	Yes	NA	NA	NA	NA
Gdist:S122028_6849	BT09	54.747	0.0415	none	No	No	No	No	No	Yes (0)	Yes (0.005)	0.016276596	0.97060027	Yes	VAR RED	Reduced	NA	NA
cDNA:S219947_3820	BT09	56.079	0.036	NA	No	No	No	No	No	Yes (0)	Yes (0.009)	0.982691275	0.80916341	Yes	NA	NA	NA	NA
cDNA:S349065_3669	BT09	56.079	0.0076	NA	No	No	No	No	No	Yes (0.02)	No	NA	NA	No	NA	NA	NA	NA
cDNA:S20157_2917	BT09	56.877	0.0415	NA	No	No	No	No	No	No	No	0.508712121	0.46026971	Yes	NA	NA	NA	NA
Gdist:S84344_3644	BT09	57.627	0.0385	NA	No	No	No	No	No	No	No	0.115121359	0.79436364	Yes	NA	NA	NA	NA
Gdist:S40778_1344	BT09	57.627	0.0095	NA	No	No	No	No	No	No	No	NA	NA	No	NA	NA	NA	NA
Gdist:S218717_4226	BT09	57.63	0.0513	NA	No	No	No	No	No	No	No	0.213317308	0.99439894	Yes	NA	NA	NA	NA
Gdist:S286419_10896	BT09	57.633	0.0053	NA	No	No	No	No	No	No	No	NA	NA	No	NA	NA	NA	NA
Gdist:S286419_16240	BT09	57.635	-0.0012	NA	No	No	No	No	No	Yes (0.037)	No	NA	NA	No	NA	NA	NA	NA
Gdist:S193081_187	BT09	57.808	0.0216	NA	No	No	No	No	No	No	No	NA	NA	No	NA	NA	NA	NA
Gdist:S193081_638	BT09	57.811	0.0333	NA	No	No	No	No	No	No	No	0.916490895	0.68551948	Yes	NA	NA	NA	NA
Gdist:S134481_11196	BT09	57.815	0.0083	NA	No	No	No	No	No	No	No	NA	NA	No	NA	NA	NA	NA
cDNA:S65210_2420	BT09	57.818	-0.0058	NA	Yes	No	No	No	No	No	No	NA	NA	No	NA	NA	NA	NA
SalHit:S32936_4225	BT09	58.4	0.0177	NA	No	No	No	No	No	No	No	NA	NA	No	NA	NA	NA	NA
Gdist:S125710_1245	BT09	58.401	0.0141	NA	No	No	No	No	No	No	No	NA	NA	No	NA	NA	NA	NA
Gdist:S265018_3760	BT09	59.181	0.0246	NA	No	No	No	No	No	Yes (0.007)	Yes (0.048)	NA	NA	No	NA	NA	NA	NA
Gdist:S200793_5657	BT09	59.191	0.0021	NA	No	No	No	No	No	No	No	NA	NA	No	NA	NA	NA	NA
Gdist:S65927_7341	BT09	59.192	0.062	NA	No	No	No	No	No	No	No	0.565714286	0.59462389	Yes	NA	NA	NA	NA
LD:S139091_1644	BT09	59.192	0.0307	NA	No	No	No	No	No	No	No	0.05961039	0.180625	Yes	NA	NA	NA	NA
Gdist:S200793_7519	BT09	59.192	0.0211	NA	No	No	No	No	No	No	No	NA	NA	No	NA	NA	NA	NA
Gdist:S72581_7641	BT09	59.192	0.0115	NA	No	No	No	No	No	No	No	NA	NA	No	NA	NA	NA	NA
LD:S139091_9797	BT09	59.192	0.0065	NA	No	No	No	No	No	No	No	NA	NA	No	NA	NA	NA	NA
Gdist:S408768_2269	BT09	59.192	0.0044	NA	No	No	No	No	No	No	No	NA	NA	No	NA	NA	NA	NA
Gdist:S677759_774	BT09	59.192	-0.0012	NA	No	No	No	No	No	No	No	NA	NA	No	NA	NA	NA	NA
cDNA:S162418_5551	BT09	59.202	0.0027	NA	No	No	No	No	No	No	No	NA	NA	No	NA	NA	NA	NA
Gdist:S380413_6454	BT09	60.23	0.0224	NA	No	No	No	No	No	No	No	NA	NA	No	NA	NA	NA	NA
Gdist:S17383_7730	BT09	61.301	0.0104	NA	No	No	No	No	No	No	No	NA	NA	No	NA	NA	NA	NA
Gdist:S249409_9272	BT09	61.317	0.0691	NA	No	No	No	No	No	No	No	0.85587766	0.83170686	Yes	NA	NA	NA	NA
Gdist:S53951_3539	BT09	61.366	0.0394	NA	No	No	No	No	No	No	No	0.982691275	0	Yes	SKJ INC	NA	increased	NA
cDNA:S410039_1043	BT09	63.766	0.0077	NA	Yes	No	No	No	No	No	No	NA	NA	No	NA	NA	NA	NA
cDNA:S486536_1792	BT09	64.267	0.0148	NA	No	No	No	No	No	No	No	NA	NA	No	NA	NA	NA	NA
Gdist:S55012_5914	BT09	65.165	0.0174	NA	No	No	No	No	No	No	No	NA	NA	No	NA	NA	NA	NA
Gdist:S505085_7507	BT09	65.165	0.0116	NA	No	No	No	No	No	No	No	NA	NA	No	NA	NA	NA	NA
Gdist:S264340_5568	BT09	65.195	0.0366	NA	No	No	No	No	No	No	No	0.016276596	0.89481818	Yes	VAR INC	increased	NA	yes
Gdist:S136510_7699	BT09	65.235	0.0254	NA	No	No	No	No	No	No	No	NA	NA	No	NA	NA	NA	NA
Gdist:S148_7189	BT09	65.275	0.0297	NA	Yes	No	No	No	No	No	No	NA	NA	No	NA	NA	NA	NA
cDNA:S345393_606	BT09	66.643	0.0382	none	No	No	No	No	No	No	No	0.928928571	0	Yes	SKJ RED	NA	reduced	yes
cDNA:S18529_1354	BT09	66.958	0.0196	NA	Yes	No	No	No	No	No	No	NA	NA	No	NA	NA	NA	NA
Gdist:S326794_3302	BT09	66.984	0.0524	NA	No	No	No	No	No	No	No	0	0	Yes	SKJ+VAR INC	increased	NA	NA
cDNA:S166130_1937	BT09	67.009	0.0083	NA	No	No	No	No	No	No	No	NA	NA	No	NA	NA	NA	NA
cDNA:S47498_732	BT09	67.529	0.0301	NA	No	No	No	No	No	No	No	0.841225314	0.8671301	Yes	NA	NA	NA	NA
SalHit:S697750_3426	BT09	68.054	0.0666	NA	No	No	No	No	No	No	No	0.870811856	0.83441924	Yes	NA	NA	NA	NA
Gdist:S238908_4593	BT09	68.58	0.0198	NA	No	No	No	No	No	Yes (0.007)	Yes (0.049)	NA	NA	No	NA	NA	NA	NA
cDNA:S377538_2760	BT09	68.619	0.0037	NA	No	No	No	No	No	Yes (0)	Yes (0.004)	NA	NA	No	NA	NA	NA	NA
cDNA:S208336_5062	BT09	68.664	0.0485	NA	No	No	No	No	No	No	No	0.705336538	0.56791921	Yes	NA	NA	NA	NA
cDNA:S546013_2702	BT09	68.734	0.0422	NA	No	No	No	No	No	Yes (0)	Yes (0.004)	0.239872881	0.28322519	Yes	NA	NA	NA	NA
cDNA:S495556_576	BT09	68.765	0.0266	NA	No	No	No	No	No	Yes (0.021)	No	NA	NA	No	NA	NA	NA	NA
SalHit:S735493_1940	BT09	68.796	0.027	NA	No	No	No	No	No	No	No	NA	NA	No	NA	NA	NA	NA
SalHit:S341887_2153	BT09	68.828	0.0235	NA	No	No	No	No	No	Yes (0)	Yes (0.007)	NA	NA	No	NA	NA	NA	NA
Gdist:S591863_1145	BT09	69.077	0.0284	NA	No	No	No	No	No	No	No	NA	NA	No	NA	NA	NA	NA
SalHit:S114016_1877	BT09	69.323	0.0083	NA	No	No	No	No	No	No	No	NA	NA	No	NA	NA	NA	NA
Gdist:S14656_1414	BT09	69.935	0.0681	NA	No	No	No	No	No	No	No	0.916490895	0.51782491	Yes	NA	NA	NA	NA
cDNA:S358873_5176	BT09	70.523	0.008	NA	No	No	No	No	No	No	No	NA	NA	No	NA	NA	NA	NA
Gdist:S112887_2627	BT09	71.111	0.034	NA	No	No	No	No	No	No	No	0.982691275	0.0306	Yes	SKJ INC	NA	increased	NA
Gdist:S112887_7924	BT09	71.666	-0.0029	NA	No	No	No	No	No	No	No	NA	NA	No	NA	NA	NA	NA
Gdist:S433254_6533	BT09	71.712	0.0132	NA	No	No	No	No	No	Yes (0.004)	Yes (0.035)	NA	NA	No	NA	NA	NA	NA
cDNA:S579834_1157	BT09	71.75	0.0634	NA	No	No	No	No	No	No	No	0.739602616	0.89481818	Yes	NA	NA	NA	NA
cDNA:S113096_14352	BT09	71.754	0.0941	PREDIC	Yes	Yes	No	No	No	Yes (0.035)	No	0.896875	0.16105263	Yes	Outlier Varde	Outlier Var	NA	yes
Gdist:S777928_6280	BT09	71.755	0.021	NA	No	No	No	No	No	No	No	NA	NA	No	NA	NA	NA	NA
Gdist:S777928_1666	BT09	71.764	0.039	PREDIC	No	No	No	No	No	No	No	0.0478125	0.4815	Yes	VAR RED	Reduced	NA	NA
cDNA:S46895_1964	BT09	71.774	0.0233	NA	No	No	No	No	No	No	No	NA	NA	No	NA	NA	NA	NA
Gdist:S137722_2258	BT09	73.106	0.0403	NA	No	No	No	No	No	No	No	0.533010355	0.1434375	Yes	NA	NA	NA	NA
cDNA:S238798_3838	BT09	74.438	0.0203	NA	No	No	No	No	No	No	No	NA	NA	No	NA	NA	NA	NA
cDNA:S509786_2280	BT09	74.439	0.03	NA	No	No	No	No	No	Yes (0.013)	No	NA	NA	No	NA	NA	NA	NA
cDNA:S242248_1277	BT09	74.495	0.0135	NA	No	No	No	No	No	No	No	NA	NA	No	NA	NA	NA	NA
cDNA:S242248_1019	BT09	74.495	0.0099	NA	No	No	No	No	No	No	No	NA	NA	No	NA	NA	NA	NA
SalHit:S267277_747	BT10	0	-0.0007	NA	No	No	No	No	No	No	No	NA	NA	No	NA	NA	NA	NA
SalarSNP:GCR_cBin11134_Ctg	BT10	2.391	0.027	NA	No	No	No	No	No	No	No	NA	NA	No	NA	NA	NA	NA

cDNA:S639012_1637	BT10	2.72	0.0149	NA	Yes	No	No	No	No	No	No	NA	NA	No	NA	NA	NA	NA
cDNA:S443384_1089	BT10	6.647	0.0126	NA	No	No	No	No	No	No	No	NA	NA	No	NA	NA	NA	NA
SalHit:S304891_1949	BT10	9.605	0.0258	NA	No	No	No	No	No	No	No	NA	NA	No	NA	NA	NA	NA
SalHit:S304891_767	BT10	9.991	-0.0077	NA	No	No	No	No	No	No	No	NA	NA	No	NA	NA	NA	NA
SalHit:S159013_6091	BT10	10.034	0.0126	NA	No	No	No	No	No	No	No	NA	NA	No	NA	NA	NA	NA
Gdist:S352552_6424	BT10	13.043	0.0121	NA	No	No	No	No	No	No	No	NA	NA	No	NA	NA	NA	NA
Gdist:S76491_5549	BT10	16.803	-0.0011	NA	No	No	No	No	No	No	No	NA	NA	No	NA	NA	NA	NA
Gdist:S117671_3409	BT10	20.562	-0.0076	NA	No	No	No	No	No	No	No	NA	NA	No	NA	NA	NA	NA
cDNA:S672840_1084	BT10	20.575	0.0202	NA	No	No	No	No	No	No	No	NA	NA	No	NA	NA	NA	NA
SalHit:S649731_1232	BT10	22.071	0.0014	NA	No	No	No	No	No	No	No	NA	NA	No	NA	NA	NA	NA
Gdist:S233810_3810	BT10	22.894	0.0145	NA	No	No	No	No	No	No	No	NA	NA	No	NA	NA	NA	NA
SalHit:S675517_922	BT10	26.39	0.0072	NA	No	No	No	No	No	No	No	NA	NA	No	NA	NA	NA	NA
cDNA:S365959_394	BT10	27.726	0.0103	NA	No	No	No	No	No	No	No	NA	NA	No	NA	NA	NA	NA
SalHit:S683685_828	BT10	27.73	0.0453	NA	No	No	No	No	No	No	No	0.710010571	0.86825552	Yes	NA	NA	NA	NA
SalHit:S460384_3598	BT10	28.454	0.0048	NA	No	No	No	No	No	No	No	NA	NA	No	NA	NA	NA	NA
SalHit:S144233_6895	BT10	33.307	0.0289	NA	Yes	No	No	No	No	No	No	NA	NA	No	NA	NA	NA	NA
Gdist:S66664_5967	BT10	33.308	0.0204	NA	No	No	No	No	No	No	No	0.427149805	0.46026971	Yes	NA	NA	NA	NA
Gdist:S104822_7598	BT10	33.308	0.0239	NA	No	No	No	No	No	No	No	NA	NA	No	NA	NA	NA	NA
cDNA:S325177_848	BT10	33.373	-0.0048	NA	No	No	No	No	No	No	No	NA	NA	No	NA	NA	NA	NA
cDNA:S52430_1340	BT10	33.596	-0.0047	NA	No	No	No	No	No	No	No	NA	NA	No	NA	NA	NA	NA
cDNA:S227479_670	BT10	34.021	0.0152	NA	No	No	No	No	No	No	No	NA	NA	No	NA	NA	NA	NA
Gdist:S280542_4627	BT10	34.222	0.0583	NA	No	No	No	No	No	No	No	0.919848943	0.82025797	Yes	NA	NA	NA	NA
Gdist:S149801_7514	BT10	38.863	0.0182	NA	No	No	No	No	No	No	No	NA	NA	No	NA	NA	NA	NA
cDNA:S479374_5109	BT10	40.295	-0.003	NA	No	No	No	No	No	No	No	NA	NA	No	NA	NA	NA	NA
Gdist:S269100_1959	BT10	43.958	0.0263	NA	No	No	No	No	No	No	No	NA	NA	No	NA	NA	NA	NA
Gdist:S313409_6746	BT10	44.5	0.0494	NA	No	No	No	No	No	No	No	0.55323913	0.21798387	Yes	NA	NA	NA	NA
SalHit:S341443_5355	BT10	44.585	-0.0154	NA	No	No	No	No	No	No	No	NA	NA	No	NA	NA	NA	NA
SalHit:S341443_3903	BT10	44.676	-0.0096	NA	No	No	No	No	No	No	No	NA	NA	No	NA	NA	NA	NA
Gdist:S25189_7758	BT10	44.728	0.0053	NA	No	No	No	No	No	Yes (0.002)	Yes (0.024)	NA	NA	No	NA	NA	NA	NA
Gdist:S135969_7447	BT10	45.001	-0.001	NA	No	No	No	No	No	No	No	NA	NA	No	NA	NA	NA	NA
Gdist:S95972_5107	BT10	46.545	0.0266	NA	No	No	No	No	No	No	No	NA	NA	No	NA	NA	NA	NA
Gdist:S58814_4848	BT10	47.001	-0.0027	NA	No	No	No	No	No	No	No	NA	NA	No	NA	NA	NA	NA
Gdist:S2691_8914	BT10	47.458	-0.0105	NA	No	No	No	No	No	No	No	NA	NA	No	NA	NA	NA	NA
Gdist:S292430_2076	BT10	47.915	0.0469	NA	No	No	No	No	No	No	No	0.729510309	0.89481818	Yes	NA	NA	NA	NA
cDNA:S409465_1832	BT10	49.068	0.0267	NA	No	No	No	No	No	No	No	NA	NA	No	NA	NA	NA	NA
Gdist:S221403_1362	BT10	49.348	0.0541	NA	No	No	No	No	No	No	No	0.574795082	0.99439894	Yes	NA	NA	NA	NA
Gdist:S200959_6679	BT10	49.629	0.0403	NA	No	No	No	No	No	No	No	0.93810219	0.49909851	Yes	NA	NA	NA	NA
Gdist:S182612_1853	BT10	53.446	-0.0004	NA	No	No	No	No	No	No	No	NA	NA	No	NA	NA	NA	NA
Gdist:S518013_3513	BT10	54.641	-0.0075	NA	No	No	No	No	No	No	No	NA	NA	No	NA	NA	NA	NA
Gdist:S518013_6627	BT10	56.199	-0.0112	NA	No	No	No	No	No	No	No	NA	NA	No	NA	NA	NA	NA
Gdist:S215773_1111	BT10	57.757	0.0106	NA	No	No	No	No	No	No	No	NA	NA	No	NA	NA	NA	NA
cDNA:S233433_1455	BT10	59.231	0.0147	NA	No	No	No	No	No	No	No	0.893131188	0.48116071	Yes	NA	NA	NA	NA
Gdist:S127471_4594	BT10	59.362	0.0079	NA	Yes	No	No	No	No	No	No	NA	NA	No	NA	NA	NA	NA
SalarSNP:ESTV_17580_1116	BT10	60.871	0.0142	NA	No	No	No	No	No	No	No	NA	NA	No	NA	NA	NA	NA
cDNA:S225247_1244	BT10	65.258	0.0316	NA	No	No	No	No	No	No	No	0.906372	0.31370504	Yes	NA	NA	NA	NA
Gdist:S321568_747	BT10	65.509	0.0243	NA	No	No	No	No	No	No	No	NA	NA	No	NA	NA	NA	NA
Gdist:S60603_1995	BT10	66.325	0.0239	NA	No	No	No	No	No	No	No	NA	NA	No	NA	NA	NA	NA
cDNA:S149017_8051	BT10	71.47	0.0063	NA	No	No	No	No	No	No	No	NA	NA	No	NA	NA	NA	NA
cDNA:S149017_7696	BT10	71.47	0.0043	NA	No	No	No	No	No	No	No	NA	NA	No	NA	NA	NA	NA
Gdist:S213566_7710	BT10	71.481	0.0197	NA	No	No	No	No	No	No	No	NA	NA	No	NA	NA	NA	NA
cDNA:C122898371_146	BT10	72.439	0.0145	NA	No	No	No	No	No	Yes (0.032)	No	NA	NA	No	NA	NA	NA	NA
cDNA:S117233_945	BT10	73.76	0.0052	NA	No	No	No	No	No	No	No	NA	NA	No	NA	NA	NA	NA
Gdist:S102366_4913	BT10	73.771	0.0129	NA	No	No	No	No	No	No	No	NA	NA	No	NA	NA	NA	NA
Gdist:S704659_217	BT10	74.917	0.0359	NA	No	No	No	No	No	No	No	0.062943038	0.78392784	Yes	NA	NA	NA	NA
Gdist:S20440_2687	BT10	75.044	0.0635	NA	No	No	No	No	No	No	No	0.176761364	0.88385252	Yes	NA	NA	NA	NA
cDNA:S586278_689	BT10	75.055	0.0236	NA	No	No	No	No	No	No	No	NA	NA	No	NA	NA	NA	NA
Gdist:S8211_2287	BT10	79.496	0.0451	NA	No	No	No	No	No	No	No	0.286374346	0.55438291	Yes	NA	NA	NA	NA
cDNA:S54056_1950	BT10	79.519	0.0041	NA	No	No	No	No	No	No	No	NA	NA	No	NA	NA	NA	NA
SalHit:S111428_3669	BT10	79.522	0.0058	NA	No	No	No	No	No	No	No	NA	NA	No	NA	NA	NA	NA
SalHit:S111428_12207	BT10	79.526	-0.0073	NA	No	No	No	No	No	No	No	NA	NA	No	NA	NA	NA	NA
cDNA:S374382_1018	BT10	79.775	0.0016	NA	No	No	No	No	No	No	No	NA	NA	No	NA	NA	NA	NA
Gdist:S264367_9420	BT10	80.029	0.0281	NA	No	No	No	No	No	No	No	NA	NA	No	NA	NA	NA	NA
Gdist:S181347_7161	BT10	80.029	0.0172	NA	No	No	No	No	No	No	No	NA	NA	No	NA	NA	NA	NA
cDNA:S97807_8343	BT10	83.346	0.0119	NA	No	No	No	No	No	No	No	NA	NA	No	NA	NA	NA	NA
Gdist:S10083_8654	BT10	83.346	0.0011	NA	No	No	No	No	No	No	No	NA	NA	No	NA	NA	NA	NA
Gdist:S314813_1830	BT10	83.696	0.0181	NA	No	No	No	No	No	No	No	NA	NA	No	NA	NA	NA	NA
Gdist:S332312_4302	BT10	83.764	0.0089	NA	No	No	No	No	No	No	No	NA	NA	No	NA	NA	NA	NA
Gdist:S226200_8143	BT10	83.833	0.0191	NA	No	No	No	No	No	Yes (0.03)	No	NA	NA	No	NA	NA	NA	NA
SalHit:S315074_1366	BT10	83.833	0.0046	NA	No	No	No	No	No	No	No	NA	NA	No	NA	NA	NA	NA
cDNA:S782156_6421	BT10	83.833	-0.0018	NA	No	No	No	No	No	No	No	NA	NA	No	NA	NA	NA	NA
cDNA:S594881_2145	BT10	83.833	-0.0086	NA	No	No	No	No	No	No	No	NA	NA	No	NA	NA	NA	NA
cDNA:S279651_4775	BT10	83.833	0.0699	NA	No	No	No	No	No	Yes (0.021)	No	0.0095625	0.83441924	Yes	VAR INC	increased	NA	NA
SalHit:S147716_301	BT10	83.903	0.0104	NA	No	No	No	No	No	No	No	NA	NA	No	NA	NA	NA	NA
SalHit:S261633_28093	BT10	85.878	0.0357	NA	No	No	No	No	No	No	No	0.614833333	0.56791921	Yes	NA	NA	NA	NA
cDNA:S551527_3124	BT10	85.892	-0.0014	NA	No	No	No	No	No	No	No	NA	NA	No	NA	NA	NA	NA
Gdist:S49554_6131	BT10	87.298	-0.0072	NA	No	No	No	No	No	No	No	NA	NA	No	NA	NA	NA	NA
Gdist:S29939_2779	BT10	87.791	0.0343	NA	No	No	No	No	No	No	No	0.926563433	0.33346154	Yes	NA	NA	NA	NA
Gdist:S119611_3628	BT10	87.835	-0.0102	NA	No	No	No	No	No	No	No	NA	NA	No	NA	NA	NA	NA
Gdist:S258349_7019	BT10	87.858	0.0173	NA	No	No	No	No	No	No	No	NA	NA	No	NA	NA	NA	NA
cDNA:S368796_2495	BT10	88.305	0.0164	NA	No	No	No	No	No	No	No	NA	NA	No	NA	NA	NA	NA
cDNA:S218110_2808	BT10	88.305	0.0033	NA	No	No	No	No	No	No	No	NA	NA	No	NA	NA	NA	NA
cDNA:S218110_1857	BT10	88.305	-0.0089	NA	No	No	No	No	No	No	No	NA	NA	No	NA	NA	NA	NA
SalHit:S356513_2505	BT10	88.306	0.0077	NA	No	No	No	No	No	No	No	NA	NA	No	NA	NA	NA	NA
Gdist:S417689_1190	BT10	88.983	0.0168	NA	No	No	No	No	No	No	No	NA	NA	No	NA	NA	NA	NA
SalHit:S406713_5055	BT10	89.255	0.0456	NA	No	No	No	No	No	No	No	0.96671108	0.84677586	Yes	NA	NA	NA	NA
Gdist:S331452_3731	BT10	89.256	0.0267	NA	No	No	No	No	No	No	No	NA	NA	No	NA	NA	NA	NA
Gdist:S331452_603	BT10	90.197	0.0008	NA	No	No	No	No	No	No	No	NA	NA	No	NA	NA	NA	NA
Gdist:S46787_12272	BT10	90.198	0.0401	NA	No	No	No	No	No	No	No	0.134391892	0.94814619	Yes	NA	NA	NA	NA
cDNA:S358699_7137	BT10	90.198	0.0084	NA	No	No	No	No	No	No	No	NA	NA	No	NA	NA	NA	NA
Gdist:S162224_9547	BT10	90.198	0.006	NA	No	No	No	No	No	No	No	NA	NA	No	NA	NA	NA	NA
Gdist:S33243_4672	BT10	90.198	0.0033	NA	No	No	No	No	No	No	No	NA	NA	No	NA	NA	NA	NA
Gdist:S138090_1240	BT10	90.198	0.002	NA	No	No	No	No	No	No	No	NA	NA	No	NA	NA	NA	NA
cDNA:S154301_764	BT10	90.198	-0.0056	NA	No	No	No	No	No	No	No	NA	NA	No	NA	NA	NA	NA
Gdist:S253331_655	BT10	91.323	-0.0045	NA	No	No	No	No	No	No	No	NA	NA	No	NA	NA	NA	NA
Gdist:S190771_3201	BT10	91.328	0.0276	NA	No	No	No	No	No	No	No	NA	NA	No	NA	NA	NA	NA
cDNA:S72507_1383	BT10	91.328	0.0165	NA	No	No	No	No	No	No	No	NA	NA	No	NA	NA	NA	NA
Gdist:S93091_14353	BT10	91.328	0.016	NA	No	No	No	No	No	Yes (0.043)	No	NA	NA	No	NA	NA	NA	NA
SalHit:S156798_3033	BT10	91.328	-0.0054	NA	No	No	No	No	No	No	No	NA	NA	No	NA	NA	NA	NA
SalHit:S269412_918	BT10	91.328	-0.0086	NA	No	No	No	No	No	No	No	NA	NA	No	NA	NA	NA	NA
Gdist:S98260_9233	BT10	91.497	0.0063	NA	Yes	No	No	No	No	No	No	NA	NA	No	NA	NA	NA	NA
Gdist:S250380_5981	BT10	91.705	0.0423	NA	No	No	No	No	No	No	No	0.614833333	0.98709677	Yes	NA	NA	NA	NA
Gdist:S178241_7465	BT10	91.705	0.0409	NA	Yes	No	No	No	No	No	No	0.870811856	0.71946429	Yes	NA	NA	NA	NA
Gdist:S589859_2320	BT10	91.705	0.0124	NA	No	No	No	No	No	No	No	0.182934783	0.89309302	Yes	NA	NA	NA	NA
Gdist:S230797_6745	BT10	91.705	0.0236	NA	Yes	No	No	No	No	No	No	NA	NA	No	NA	NA	NA	NA
Gdist:S190952_6693	BT10	91.705	0.0197	NA	No	No	No	No	No	No	No	NA	NA	No	NA	NA	NA	NA
Gdist:S176673_5059	BT10	91.705	0.0148	NA	No	No	No	No	No	No	No	NA	NA	No	NA	NA	NA	NA
Gdist:S126246_5762	BT10	91.705	0.0113	NA	No	No	No	No	No	No	No	NA	NA	No	NA	NA	NA	NA
Gdist:S98539_10619	BT10	91.705	0.0111	NA	No	No	No	No	No	Yes (0.021)	No	NA	NA	No	NA	NA	NA	NA
Gdist:S292815_3953	BT10	91.705	0.0102	NA	No	No	No	No	No	No	No	NA	NA	No	NA	NA	NA	NA
Gdist:S250380_7179	BT10	91.705	0.0038	NA	No	No	No	No	No	No	No	NA	NA	No	NA	NA	NA	NA
Gdist:S100032_8838	BT10	91.705	0.0004	NA	No	No	No	No	No	No	No	NA	NA	No	NA	NA	NA	NA
SalHit:S249108_1342	BT10	91.705	-0.003	NA	No	No	No	No	No	No	No	NA	NA	No	NA	NA	NA	NA
Gdist:S230797_6341	BT10	91.705	-0.0042	NA	No	No	No	No	No	No	No	NA	NA	No	NA	NA	NA	NA
Gdist:S589859_9693	BT10	91.705	-0.0058	NA	No	No	No	No	No	No	No	NA	NA	No	NA	NA	NA	NA
Gdist:S115795_8147	BT10	91.705	-0.0077	NA	No	No	No	No	No	No	No	NA	NA	No	NA	NA	NA	NA
Gdist:S261360_4638	BT10	91.705	-0.0084	NA	No	No	No	No	No	No	No	NA	NA	No	NA	NA	NA	NA
Gdist:S13847_7026	BT10	91.705	0.0752	NA	Yes	no	no	No	No	No	No	NA	NA	no	NA	NA	NA	NA
Gdist:S622109_7026	BT10	91.705	0.0388	PREDIC	No	No	No	No	No	No	No	0.0478125	0.32265306	Yes	VAR RED	Reduced	NA	yes
SalHit:S820702_1332	BT10	91.71	-0.0064	NA	No	No	No	No	No	Yes (0)	Yes (0.004)	NA	NA	No	NA	NA	NA	NA
cDNA:S97921_4263	BT10	91.714	0.0166	NA	No	No	No	No	No	No	No	NA	NA	No	NA	NA	NA	NA
Gdist:S215628_1616	BT10	91.719	0.0121	NA	No	No	No	No	No	No	No	NA	NA	No	NA	NA	NA	NA
Gdist:S239871_4455	BT10	91.723	0.0115	NA	No	No	No	No	No	No	No	NA	NA	No	NA	NA	NA	NA
SalHit:S468935_401	BT10	91.727	-0.0082	NA	No	No	No	No	No	No	No	NA	NA	No	NA	NA	NA	NA
SalHit:S543389_5919	BT10	92.996	0.0159	NA	No	No	No	No	No	No	No	NA	NA	No	NA	NA	NA	NA
Gdist:S710217_1748	BT10	93.558	-0.009	NA	No	No	No	No	No	No	No	NA	NA	No	NA	NA	NA	NA
Gdist:S61145_1101	BT10	94.174	0.0026	NA	No	No	No	No	No	Yes (0.019)	No	NA	NA	No	NA	NA	NA	NA
Gdist:S162264_883	BT10	94.201	0.0194	NA	No	No	No	No	No	No	No	NA	NA	No	NA	NA	NA	NA

Gdist:S162264_6776	BT10	94.253	0.0114	NA	No	No	No	No	No	No	No	NA	NA	No	NA	NA	NA	NA
SalHit:S833101_575	BT10	94.307	-0.0062	NA	No	No	No	No	No	No	No	NA	NA	No	NA	NA	NA	NA
cDNA:S762880_153	BT10	94.308	0.0373	NA	Yes	No	No	No	No	No	No	0.798073308	0.54853698	Yes	NA	NA	NA	NA
SalHit:S208211_6081	BT10	94.309	0.0357	NA	No	No	No	No	No	No	No	0.519280063	0.49637405	Yes	NA	NA	NA	NA
Gdist:S255921_1881	BT10	94.309	-0.0065	NA	No	No	No	No	No	No	No	NA	NA	No	NA	NA	NA	NA
Gdist:S41493_624	BT10	94.566	0.0182	NA	No	No	No	No	No	No	No	NA	NA	No	NA	NA	NA	NA
cDNA:S471028_893	BT10	94.739	0.0272	NA	No	No	No	No	No	No	No	NA	NA	No	NA	NA	NA	NA
Gdist:S343453_2566	BT10	94.739	0.0098	NA	No	No	No	No	No	No	No	NA	NA	No	NA	NA	NA	NA
Gdist:S583162_7049	BT10	94.739	0.0097	NA	No	No	No	No	No	No	No	NA	NA	No	NA	NA	NA	NA
Gdist:S139261_7130	BT10	94.739	0.0079	NA	No	No	No	No	No	No	No	NA	NA	No	NA	NA	NA	NA
cDNA:S12713_1968	BT10	94.739	0.0061	NA	No	No	No	No	No	Yes (0.007)	Yes (0.048)	NA	NA	No	NA	NA	NA	NA
cDNA:S12713_7023	BT10	94.743	0.0145	NA	No	No	No	No	No	No	No	NA	NA	No	NA	NA	NA	NA
cDNA:S367995_3467	BT10	94.762	0.0099	NA	No	No	No	No	No	No	No	NA	NA	No	NA	NA	NA	NA
cDNA:S580273_477	BT10	94.786	-0.004	NA	No	No	No	No	No	No	No	NA	NA	No	NA	NA	NA	NA
SalHit:S115876_228	BT11	0	0.0007	NA	No	No	No	No	No	No	No	NA	NA	No	NA	NA	NA	NA
Gdist:S144962_3154	BT11	0.004	0.0319	NA	No	No	No	No	No	No	No	0.333987805	0.50484375	Yes	NA	NA	NA	NA
Gdist:S2710_10392	BT11	0.004	0.0171	NA	No	No	No	No	No	No	No	NA	NA	No	NA	NA	NA	NA
cDNA:S366188_990	BT11	0.004	0.0149	NA	No	No	No	No	No	No	No	NA	NA	No	NA	NA	NA	NA
Gene:S118286_2598	BT11	0.004	0.0015	NA	No	No	No	No	No	No	No	NA	NA	No	NA	NA	NA	NA
cDNA:S213304_2056	BT11	0.006	0.0141	NA	No	No	No	No	No	No	No	NA	NA	No	NA	NA	NA	NA
cDNA:S307643_3137	BT11	0.008	0.0027	NA	No	No	No	No	No	No	No	NA	NA	No	NA	NA	NA	NA
cDNA:S14978_1947	BT11	0.98	-0.0051	NA	No	No	No	No	No	No	No	NA	NA	No	NA	NA	NA	NA
cDNA:S239832_569	BT11	1.987	0.0375	NA	No	No	No	No	No	No	No	0.896875	0.21798387	Yes	NA	NA	NA	NA
cDNA:S779032_1920	BT11	3.499	0.0102	NA	No	No	No	No	No	No	No	NA	NA	No	NA	NA	NA	NA
cDNA:S612731_1264	BT11	3.499	0.0065	NA	No	No	No	No	No	No	No	NA	NA	No	NA	NA	NA	NA
SalHit:S33900_1460	BT11	8.019	0.0187	NA	No	No	No	No	No	No	No	0.958982143	0.88194268	Yes	NA	NA	NA	NA
cDNA:S577556_2090	BT11	8.019	0.0008	NA	No	No	No	No	No	No	No	NA	NA	No	NA	NA	NA	NA
Gdist:S88467_9031	BT11	13.718	-0.0074	NA	No	No	No	No	No	No	No	NA	NA	No	NA	NA	NA	NA
Gdist:S243036_4817	BT11	14.994	-0.001	NA	No	No	No	No	No	No	No	NA	NA	No	NA	NA	NA	NA
Gdist:S243036_3109	BT11	15.035	0.0315	NA	No	No	No	No	No	No	No	0.893131188	0.04098214	Yes	SKJ INC	NA	increased	yes
Gdist:S214559_4353	BT11	17.195	0.01	NA	No	No	No	No	No	No	No	NA	NA	No	NA	NA	NA	NA
Gdist:S45919_9311	BT11	19.444	0.0168	NA	No	No	No	No	No	No	No	NA	NA	No	NA	NA	NA	NA
SalHit:S45460_2763	BT11	19.749	-0.0111	NA	No	No	No	No	No	No	No	NA	NA	No	NA	NA	NA	NA
SalHit:S45460_891	BT11	21.126	-0.0068	NA	No	No	No	No	No	Yes (0.013)	No	NA	NA	No	NA	NA	NA	NA
cDNA:S123351_2073	BT11	21.622	0.0868	NA	No	No	No	No	No	No	No	0.534375	0.49897556	Yes	NA	NA	NA	NA
SalHit:S519253_4343	BT11	21.622	0.0287	NA	No	No	No	No	No	No	No	NA	NA	No	NA	NA	NA	NA
SalHit:S258084_579	BT11	21.622	0.0219	NA	No	No	No	No	No	No	No	NA	NA	No	NA	NA	NA	NA
cDNA:S290555_1498	BT11	21.622	0.0017	NA	No	No	No	No	No	No	No	NA	NA	No	NA	NA	NA	NA
SalHit:S519253_1139	BT11	21.622	-0.0049	NA	No	No	No	No	No	No	No	NA	NA	No	NA	NA	NA	NA
cDNA:S123351_5681	BT11	21.622	-0.0089	NA	No	No	No	No	No	No	No	NA	NA	No	NA	NA	NA	NA
SalHit:S414332_763	BT11	21.723	-0.0002	NA	No	No	No	No	No	No	No	NA	NA	No	NA	NA	NA	NA
cDNA:S118058_4136	BT11	26.287	-0.009	NA	No	No	No	No	No	No	No	NA	NA	No	NA	NA	NA	NA
Gdist:S113823_3877	BT11	26.547	0.0047	NA	No	No	No	No	No	No	No	NA	NA	No	NA	NA	NA	NA
cDNA:S36111_15008	BT11	26.602	0.0217	NA	No	No	No	No	No	No	No	NA	NA	No	NA	NA	NA	NA
cDNA:S36111_2504	BT11	26.602	0.0105	NA	No	No	No	No	No	No	No	NA	NA	No	NA	NA	NA	NA
Gdist:S198580_2128	BT11	26.607	0.0336	NA	No	No	No	No	No	No	No	0.057636986	0.89481818	Yes	NA	NA	NA	NA
cDNA:S524260_1085	BT11	28.241	0.0069	NA	No	No	No	No	No	No	No	NA	NA	No	NA	NA	NA	NA
cDNA:S95432_762	BT11	28.349	0.0004	NA	No	No	No	No	No	No	No	NA	NA	No	NA	NA	NA	NA
cDNA:S72460_1666	BT11	30.369	0.0069	NA	No	No	No	No	No	No	No	NA	NA	No	NA	NA	NA	NA
Gdist:S120922_10442	BT11	32.482	-0.0019	NA	No	No	No	No	No	No	No	NA	NA	No	NA	NA	NA	NA
cDNA:S488628_11250	BT11	32.533	0.0885	NA	No	No	No	No	No	No	No	0.453596654	0.4818314	Yes	NA	NA	NA	NA
cDNA:S80201_8381	BT11	32.67	0.0036	NA	No	No	No	No	No	No	No	NA	NA	No	NA	NA	NA	NA
LD:S158022_2718	BT11	34.225	0.0256	NA	No	No	No	No	No	No	No	NA	NA	No	NA	NA	NA	NA
LD:S158022_10708	BT11	34.225	0.0001	NA	No	No	No	No	No	No	No	NA	NA	No	NA	NA	NA	NA
SalHit:S49115_8466	BT11	34.226	0.0406	NA	No	No	No	No	No	No	No	0.781630435	0.80649408	Yes	NA	NA	NA	NA
Gdist:S9963_7098	BT11	34.226	-0.0028	NA	No	No	No	No	No	No	No	NA	NA	No	NA	NA	NA	NA
Gdist:S271622_5595	BT11	34.226	-0.0083	NA	No	No	No	No	No	No	No	NA	NA	No	NA	NA	NA	NA
Gdist:S121022_623	BT11	34.226	0.081	none	No	No	No	No	No	No	No	0.0095625	0.32224315	Yes	VAR RED	Reduced	NA	yes
Gdist:S320083_398	BT11	35.11	0.0314	NA	No	No	No	No	No	No	No	0.204	0.71684019	Yes	NA	NA	NA	NA
Gdist:S320083_7200	BT11	35.176	0.0033	NA	No	No	No	No	No	No	No	NA	NA	No	NA	NA	NA	NA
Gdist:S22348_623	BT11	36.597	-0.0056	NA	No	No	No	No	No	No	No	NA	NA	No	NA	NA	NA	NA
Gdist:S57312_1819	BT11	39.579	0.0025	NA	No	No	No	No	No	No	No	NA	NA	No	NA	NA	NA	NA
Gdist:S424710_1044	BT11	39.583	0.0197	NA	No	No	No	No	No	Yes (0.022)	No	NA	NA	No	NA	NA	NA	NA
Gdist:S88922_2932	BT11	39.583	0.0196	NA	No	No	No	No	No	No	No	NA	NA	No	NA	NA	NA	NA
Gdist:S57312_4963	BT11	39.583	0.0078	NA	No	No	No	No	No	No	No	NA	NA	No	NA	NA	NA	NA
Gdist:S8865_1286	BT11	41.098	0.0048	NA	No	No	No	No	No	No	No	NA	NA	No	NA	NA	NA	NA
Gdist:S60774_9466	BT11	41.098	0.0014	NA	No	No	No	No	No	No	No	NA	NA	No	NA	NA	NA	NA
SalHit:S371829_3660	BT11	41.098	0.0007	NA	No	No	No	No	No	No	No	NA	NA	No	NA	NA	NA	NA
Gdist:S31046_2532	BT11	41.858	0.0197	NA	No	No	No	No	No	No	No	NA	NA	No	NA	NA	NA	NA
cDNA:S2985_4670	BT11	41.858	-0.0033	NA	No	No	No	No	No	No	No	NA	NA	No	NA	NA	NA	NA
cDNA:S167751_4478	BT11	42.669	0.0281	NA	No	No	No	No	No	No	No	NA	NA	No	NA	NA	NA	NA
Gdist:S319153_3473	BT11	46.695	0.0373	NA	No	No	No	No	No	No	No	0.514568404	0.46026971	Yes	NA	NA	NA	NA
Gdist:S99664_3018	BT11	47.411	0.0958	NA	No	No	No	No	No	Yes (0)	Yes (0.009)	0.530792308	0.9985	Yes	NA	NA	NA	NA
SalHit:S776102_2112	BT11	47.573	0.0367	PREDIC'	No	No	No	No	No	No	No	0	0.53217391	Yes	VAR RED	Reduced	NA	yes
cDNA:S255526_428	BT11	47.734	0.101	NA	No	No	No	No	No	No	No	0.050625	0.9985	Yes	NA	NA	NA	NA
SalHit:S279771_2947	BT11	47.806	0.1097	NA	No	No	No	No	No	Yes (0.037)	No	0.365723684	0.33791411	Yes	NA	NA	NA	NA
SalHit:S279771_1404	BT11	47.806	0.0572	NA	No	No	No	No	No	No	No	0.357593023	0.93279221	Yes	NA	NA	NA	NA
Gdist:S245652_6615	BT11	48.185	0.0403	NA	No	No	No	No	No	No	No	0.331875	0.87869952	Yes	NA	NA	NA	NA
Gene:S307364_1802	BT11	48.185	0.0257	NA	No	No	No	No	No	No	No	NA	NA	No	NA	NA	NA	NA
SalHit:S242306_1973	BT11	48.185	0.0034	NA	No	No	No	No	No	No	No	NA	NA	No	NA	NA	NA	NA
cDNA:S243280_5038	BT11	48.185	-0.003	NA	No	No	No	No	No	No	No	NA	NA	No	NA	NA	NA	NA
cDNA:S239551_1419	BT11	48.209	0.028	NA	No	No	No	No	No	No	No	NA	NA	No	NA	NA	NA	NA
cDNA:S65320_3890	BT11	48.234	0.0558	NA	No	No	No	No	No	No	No	0.340406699	0.74923536	Yes	NA	NA	NA	NA
cDNA:S134565_1451	BT11	49.825	0.0364	NA	No	No	No	No	No	No	No	0.870811856	0.76663812	Yes	NA	NA	NA	NA
Gdist:S19151_10137	BT11	49.873	0.102	NA	No	No	No	No	No	Yes (0.002)	Yes (0.024)	0.633830935	0.60712604	Yes	NA	NA	NA	NA
Gdist:S6488_4748	BT11	49.873	0.0397	NA	No	No	No	No	No	No	No	0.367597403	0.73333893	Yes	NA	NA	NA	NA
Gdist:S45895_6931	BT11	49.873	0.0236	NA	Yes	No	No	No	No	No	No	NA	NA	No	NA	NA	NA	NA
Gdist:S79935_1053	BT11	49.874	-0.0008	NA	No	No	No	No	No	No	No	NA	NA	No	NA	NA	NA	NA
Gdist:S85175_9255	BT11	49.875	0.111	NA	Yes	No	No	No	No	No	No	0.462071168	0.3565678	Yes	NA	NA	NA	NA
Gdist:S85175_3996	BT11	49.876	0.0834	NA	No	No	No	No	No	No	No	0.74387574	0.39220812	Yes	NA	NA	NA	NA
Gdist:S225852_5032	BT11	49.877	0.082	NA	No	No	No	No	No	No	No	0.46154059	0.79436364	Yes	NA	NA	NA	NA
Gdist:S78393_2797	BT11	49.877	0.054	NA	No	No	No	No	No	No	No	0.18	0.42304147	Yes	NA	NA	NA	NA
Gdist:S193883_6069	BT11	49.877	0.0323	NA	No	No	No	No	No	No	No	0.0765	0.72269585	Yes	NA	NA	NA	NA
Gdist:S480407_8341	BT11	49.877	0.0166	NA	No	No	No	No	No	No	No	NA	NA	No	NA	NA	NA	NA
Gdist:S403763_2905	BT11	49.877	-0.0113	NA	No	No	No	No	No	No	No	NA	NA	No	NA	NA	NA	NA
Gdist:S233150_6194	BT11	50.199	0.0024	NA	No	No	No	No	No	Yes (0.009)	Yes (0.049)	NA	NA	No	NA	NA	NA	NA
Gdist:S30786_2297	BT11	50.525	0.0061	NA	No	No	No	No	No	No	No	NA	NA	No	NA	NA	NA	NA
Gdist:S326335_5598	BT11	51.799	0.0176	NA	No	No	No	No	No	No	No	NA	NA	No	NA	NA	NA	NA
Gdist:S538198_2907	BT11	52.083	0.0326	NA	No	No	No	No	No	No	No	0.053873239	0.83274908	Yes	NA	NA	NA	NA
Gdist:S314969_4922	BT11	52.35	0	NA	No	No	No	No	No	No	No	NA	NA	No	NA	NA	NA	NA
Gdist:S47214_7025	BT11	52.448	0.0267	NA	No	No	No	No	No	No	No	NA	NA	No	NA	NA	NA	NA
Gdist:S185339_7770	BT11	52.538	0.0518	NA	Yes	No	No	No	No	No	No	0.6196875	0.76824153	Yes	NA	NA	NA	NA
Gdist:S111632_10971	BT11	52.64	0.0014	NA	No	No	No	No	No	No	No	NA	NA	No	NA	NA	NA	NA
Gdist:S49826_3445	BT11	52.728	0.0617	NA	No	No	No	No	No	No	No	0.471390845	0.6958849	Yes	NA	NA	NA	NA
cDNA:S105534_2802	BT11	52.728	0.041	NA	No	No	No	No	No	No	No	0.240674157	0.83332873	Yes	NA	NA	NA	NA
Gdist:S62212_1950	BT11	52.728	0.0245	NA	No	No	No	No	No	No	No	NA	NA	No	NA	NA	NA	NA
Gdist:S428213_1072	BT11	52.728	0.0219	NA	No	No	No	No	No	Yes (0)	Yes (0.004)	NA	NA	No	NA	NA	NA	NA
Gdist:S226687_10648	BT11	52.728	0.0163	NA	No	No	No	No	No	No	No	NA	NA	No	NA	NA	NA	NA
Gdist:S342944_6951	BT11	52.728	0.0093	NA	No	No	No	No	No	No	No	NA	NA	No	NA	NA	NA	NA
cDNA:S412123_7384	BT11	52.728	0.0036	NA	No	No	No	No	No	No	No	NA	NA	No	NA	NA	NA	NA
Gdist:S99675_8950	BT11	52.728	0.0033	NA	No	No	No	No	No	No	No	NA	NA	No	NA	NA	NA	NA
SalHit:S35917_3484	BT11	52.728	-0.0029	NA	No	No	No	No	No	No	No	NA	NA	No	NA	NA	NA	NA
Gdist:S77639_2052	BT11	52.728	-0.0059	NA	No	No	No	No	No	No	No	NA	NA	No	NA	NA	NA	NA
LD:S146297_6295	BT11	52.786	0.0153	NA	No	No	No	No	No	No	No	0.907678571	0.81337062	Yes	NA	NA	NA	NA
Gdist:S83646_2969	BT11	52.842	0.0263	NA	No	No	No	No	No	No	No	NA	NA	No	NA	NA	NA	NA
Gdist:S113290_2526	BT11	52.919	0.0304	NA	Yes	No	No	No	No	Yes (0)	Yes (0.008)	0.36125	0.72648741	Yes	NA	NA	NA	NA
cDNA:S687718_121	BT11	52.989	-0.0061	NA	No	No	No	No	No	No	No	NA	NA	No	NA	NA	NA	NA
cDNA:S36670_3719	BT11	53.05	0.0044	NA	No	No	No	No	No	No	No	NA	NA	No	NA	NA	NA	NA
Gdist:S225670_5253	BT11	53.105	0.0582	NA	No	No	No	No	No	No	No	0.937741935	0.87616995	Yes	NA	NA	NA	NA
Gdist:S391408_5061	BT11	53.105	0.057	NA	No	No	No	No	No	No	No	0.079862637	0.72269585	Yes	NA	NA	NA	NA
Gdist:S64149_15533	BT11	53.105	0.0284	NA	No	No	No	No	No	No	No	NA	NA	No	NA	NA	NA	NA
cDNA:S512863_3037	BT11	53.105	0.0272	NA	No	No	No	No	No	No	No	NA	NA	No	NA	NA	NA	NA
Gdist:S225670_11443	BT11	53.105	0.0159	NA	No	No	No	No	No	No	No	NA	NA	No	NA	NA	NA	NA
SalHit:S85907_571	BT11	53.105	0.0074	NA	No	No	No	No	No	No	No	NA	NA	No	NA	NA	NA	NA
Gdist:S208396_4887	BT11	53.105	0.0047	NA	No	No	No	No	No	No	No	NA	NA	No	NA	NA	NA	NA
cDNA:S831140_2740	BT11	53.105	0.0022	NA	No	No	No	No	No	No	No	NA	NA	No	NA	NA	NA	NA
cDNA:S185419_3086	BT11	53.105	-0.0025	NA	No	No	No	No	No	No	No	NA	NA	No	NA	NA	NA	NA

Gdist:S467063_2379	BT11	53.105	-0.0093	NA	No	No	No	No	No	No	No	NA	NA	No	NA	NA	NA	NA
Gdist:S135310_8259	BT11	53.273	0.014	NA	No	No	No	No	No	No	No	NA	NA	No	NA	NA	NA	NA
cDNA:S621565_1910	BT11	53.392	0.0093	NA	No	No	No	No	No	No	No	NA	NA	No	NA	NA	NA	NA
Gdist:S681213_7149	BT11	53.512	0.0165	NA	No	No	No	No	No	No	No	NA	NA	No	NA	NA	NA	NA
cDNA:S506131_1661	BT11	53.512	-0.0029	NA	No	No	No	No	No	No	No	NA	NA	No	NA	NA	NA	NA
SalarSNP:ESTV_16545_824	BT11	53.519	0.005	NA	No	No	No	No	No	No	No	NA	NA	No	NA	NA	NA	NA
Gdist:S369267_1186	BT12	0	0.0327	NA	No	No	No	No	No	No	No	0.870811856	0.82148131	Yes	NA	NA	NA	NA
cDNA:S199676_7836	BT12	0	-0.008	NA	No	No	No	No	No	No	No	NA	NA	No	NA	NA	NA	NA
Gdist:S511835_4059	BT12	0.001	-0.0052	NA	No	No	No	No	No	No	No	NA	NA	No	NA	NA	NA	NA
Gdist:S237175_830	BT12	0.001	-0.0072	NA	No	No	No	No	No	No	No	NA	NA	No	NA	NA	NA	NA
cDNA:S21271_1377	BT12	4.088	0.0124	NA	No	No	No	No	No	Yes (0)	Yes (0.004)	NA	NA	No	NA	NA	NA	NA
cDNA:S632206_1402	BT12	8.425	0.0309	NA	No	No	No	No	No	No	No	0.299431818	0.29401119	Yes	NA	NA	NA	NA
Gdist:S99944_3542	BT12	9.28	0.0123	NA	No	No	No	No	No	No	No	NA	NA	No	NA	NA	NA	NA
Gdist:S37571_17888	BT12	10.057	-0.0073	NA	No	No	No	No	No	No	No	NA	NA	No	NA	NA	NA	NA
SalHit:S22575_3588	BT12	10.476	0.0218	NA	No	No	No	No	No	No	No	0.083152174	0.87029851	Yes	NA	NA	NA	NA
SalHit:S22575_5272	BT12	10.477	0.0078	NA	No	No	No	No	No	No	No	NA	NA	No	NA	NA	NA	NA
cDNA:S684263_1131	BT12	10.479	0.0634	NA	No	No	No	No	No	No	No	0.633830935	0.77613306	Yes	NA	NA	NA	NA
Gdist:S170810_3416	BT12	10.48	0.0431	NA	No	No	No	No	No	No	No	0.531768293	0.97060027	Yes	NA	NA	NA	NA
SalHit:S440995_2172	BT12	11.111	0.0123	NA	No	No	No	No	No	No	No	NA	NA	No	NA	NA	NA	NA
Gdist:S377637_7617	BT12	13.302	0.0125	NA	No	No	No	No	No	No	No	NA	NA	No	NA	NA	NA	NA
Gdist:S620174_5049	BT12	19.336	0.0294	NA	No	No	No	No	No	Yes (0)	Yes (0.006)	NA	NA	No	NA	NA	NA	NA
cDNA:S277533_383	BT12	19.336	0.0117	NA	No	No	No	No	No	No	No	NA	NA	No	NA	NA	NA	NA
Gdist:S620174_7926	BT12	19.339	0.0045	NA	No	No	No	No	No	No	No	NA	NA	No	NA	NA	NA	NA
Gdist:S64750_11311	BT12	19.392	-0.0016	NA	No	No	No	No	No	No	No	NA	NA	No	NA	NA	NA	NA
Gdist:S80500_2065	BT12	20.529	0.0062	NA	No	No	No	No	No	Yes (0)	Yes (0.005)	NA	NA	No	NA	NA	NA	NA
Gdist:S82255_10685	BT12	25.574	0.0619	NA	Yes	No	No	No	No	Yes (0)	Yes (0.004)	0.682433921	0.23395631	Yes	NA	NA	NA	NA
Gdist:S181174_9540	BT12	30.077	0.0029	NA	No	No	No	No	No	Yes (0.032)	No	NA	NA	No	NA	NA	NA	NA
SalHit:S176657_4893	BT12	33.055	0.0091	NA	No	No	No	No	No	No	No	NA	NA	No	NA	NA	NA	NA
cDNA:S151742_11162	BT12	34.465	-0.0023	NA	No	No	No	No	No	Yes (0.021)	No	NA	NA	No	NA	NA	NA	NA
cDNA:S245573_4771	BT12	34.507	-0.0103	NA	No	No	No	No	No	No	No	NA	NA	No	NA	NA	NA	NA
Gdist:S609767_4989	BT12	34.754	0.0244	NA	No	No	No	No	No	No	No	0.050625	0.57143182	Yes	NA	NA	NA	NA
Gdist:S609767_3601	BT12	35.252	0.0014	NA	No	No	No	No	No	Yes (0.006)	Yes (0.042)	NA	NA	No	NA	NA	NA	NA
Gdist:S188311_7585	BT12	38.789	0.0041	NA	No	No	No	No	No	No	No	NA	NA	No	NA	NA	NA	NA
Gdist:S7143_5827	BT12	39.897	-0.0031	NA	No	No	No	No	No	No	No	NA	NA	No	NA	NA	NA	NA
LD:S143561_5364	BT12	40.681	0.0046	NA	No	No	No	No	No	Yes (0)	Yes (0.008)	NA	NA	No	NA	NA	NA	NA
Gdist:S424804_4900	BT12	40.937	0.036	NA	No	No	No	No	No	No	No	0.838743243	0.82025797	Yes	NA	NA	NA	NA
Gdist:S47229_2227	BT12	40.956	0.0099	NA	No	No	No	No	No	No	No	NA	NA	No	NA	NA	NA	NA
Gdist:S64593_7063	BT12	40.977	0.0057	NA	No	No	No	No	No	No	No	NA	NA	No	NA	NA	NA	NA
Gdist:S376158_15965	BT12	40.977	-0.0004	NA	No	No	No	No	No	No	No	NA	NA	No	NA	NA	NA	NA
SalHit:S102870_18665	BT12	41.096	0.0035	NA	No	No	No	No	No	No	No	NA	NA	No	NA	NA	NA	NA
Gdist:S648683_6096	BT12	43.067	0.0167	NA	No	No	No	No	No	Yes (0.003)	Yes (0.024)	NA	NA	No	NA	NA	NA	NA
Gdist:S333284_8105	BT12	43.227	0.0497	NA	No	No	No	No	No	No	No	0.773826923	0.71381387	Yes	NA	NA	NA	NA
SalHit:S585005_1586	BT12	43.227	-0.0111	NA	No	No	No	No	No	No	No	NA	NA	No	NA	NA	NA	NA
Gdist:S295466_3259	BT12	43.232	-0.0005	NA	No	No	No	No	No	Yes (0.021)	No	NA	NA	No	NA	NA	NA	NA
Gdist:S697184_8164	BT12	43.237	0.0665	NA	No	No	No	No	No	No	No	0.965664557	0.48116071	Yes	NA	NA	NA	NA
Gdist:S193534_15221	BT12	43.242	0.0372	NA	No	No	No	No	No	No	No	0.14025	0.69352273	Yes	NA	NA	NA	NA
Gdist:S193534_15834	BT12	43.249	0.0022	NA	No	No	No	No	No	No	No	NA	NA	No	NA	NA	NA	NA
Gdist:S151026_8038	BT12	43.255	0.0147	NA	No	No	No	No	No	No	No	NA	NA	No	NA	NA	NA	NA
Gdist:S517903_9642	BT12	43.261	0.0146	NA	No	No	No	No	No	Yes (0.037)	No	NA	NA	No	NA	NA	NA	NA
Gdist:S32801_7671	BT12	44.085	-0.0137	NA	No	No	No	No	No	No	No	NA	NA	No	NA	NA	NA	NA
Gdist:S372824_1686	BT12	44.925	0.0196	NA	No	No	No	No	No	No	No	NA	NA	No	NA	NA	NA	NA
Gdist:S234007_8606	BT12	45.831	0.0056	NA	Yes	No	No	No	No	No	No	NA	NA	No	NA	NA	NA	NA
cDNA:S10378_3626	BT12	45.831	0.0016	NA	Yes	No	No	No	No	No	No	NA	NA	No	NA	NA	NA	NA
Gdist:S375841_2581	BT12	46.456	0.0256	NA	No	No	No	No	No	No	No	NA	NA	No	NA	NA	NA	NA
Gdist:S53374_5251	BT12	46.469	0.0486	none	No	No	No	No	No	No	No	0	0.26256356	Yes	VAR RED	Reduced	NA	yes
Gdist:S119816_3896	BT12	47.255	0.0641	NA	No	No	No	No	No	No	No	0.593534483	0.69352273	Yes	NA	NA	NA	NA
LD:S185091_1642	BT12	48.887	0.0289	NA	No	No	No	No	No	No	No	NA	NA	No	NA	NA	NA	NA
cDNA:S306803_1347	BT12	48.887	0.007	NA	No	No	No	No	No	No	No	NA	NA	No	NA	NA	NA	NA
LD:S185091_6658	BT12	48.887	0.0006	NA	No	No	No	No	No	No	No	NA	NA	No	NA	NA	NA	NA
Gdist:S78966_7269	BT12	48.893	-0.005	NA	Yes	No	No	No	No	No	No	NA	NA	No	NA	NA	NA	NA
cDNA:S529070_2405	BT12	50.096	0.019	NA	No	No	No	No	No	No	No	NA	NA	No	NA	NA	NA	NA
Gdist:S69319_1850	BT12	50.096	0.013	NA	No	No	No	No	No	No	No	NA	NA	No	NA	NA	NA	NA
SalHit:S47024_3427	BT12	50.096	0.0083	NA	No	No	No	No	No	No	No	NA	NA	No	NA	NA	NA	NA
cDNA:S37230_3170	BT12	50.096	0.0048	NA	No	No	No	No	No	No	No	NA	NA	No	NA	NA	NA	NA
SalHit:S47024_3725	BT12	50.096	0.001	NA	No	No	No	No	No	No	No	NA	NA	No	NA	NA	NA	NA
cDNA:S225132_7775	BT12	50.096	-0.0107	NA	No	No	No	No	No	No	No	NA	NA	No	NA	NA	NA	NA
Gdist:S516664_7926	BT12	51.808	0.0169	NA	No	No	No	No	No	Yes (0)	Yes (0.007)	NA	NA	No	NA	NA	NA	NA
Gdist:S114220_1645	BT12	51.808	-0.0048	NA	No	No	No	No	No	No	No	NA	NA	No	NA	NA	NA	NA
Gdist:S8853_8906	BT12	52.067	0.0104	NA	No	No	No	No	No	No	No	NA	NA	No	NA	NA	NA	NA
Gdist:S8853_7577	BT12	52.311	0.057	NA	No	No	No	No	No	No	No	0.171673228	0.53217391	Yes	NA	NA	NA	NA
Gdist:S158346_7065	BT12	52.338	0.009	NA	No	No	No	No	No	No	No	NA	NA	No	NA	NA	NA	NA
cDNA:S373049_4395	BT12	52.362	0.0368	NA	No	No	No	No	No	No	No	0.074634146	0.33346154	Yes	NA	NA	NA	NA
cDNA:S136795_3145	BT12	52.362	0.0737	none	No	No	No	No	No	No	No	0.0095625	0.87029851	Yes	VAR RED	Reduced	NA	NA
Gdist:S11927_5169	BT12	53.325	0.0602	NA	Yes	No	No	No	No	No	No	0.453596654	0.89481818	Yes	NA	NA	NA	NA
cDNA:S106918_2860	BT12	53.326	-0.0067	NA	No	No	No	No	No	No	No	NA	NA	No	NA	NA	NA	NA
Gdist:S408123_8147	BT12	53.327	-0.0084	NA	No	No	No	No	No	No	No	NA	NA	No	NA	NA	NA	NA
cDNA:S700526_485	BT12	53.331	0.0298	NA	No	No	No	No	No	Yes (0.023)	No	NA	NA	No	NA	NA	NA	NA
SalHit:S57706_1989	BT12	53.709	0.0496	NA	No	No	No	No	No	Yes (0.048)	No	0.607901786	0.73616408	Yes	NA	NA	NA	NA
cDNA:S336443_13972	BT12	54.094	-0.0032	NA	No	No	No	No	No	No	No	NA	NA	No	NA	NA	NA	NA
Gdist:S508336_3453	BT12	54.626	0.0208	NA	No	No	No	No	No	No	No	NA	NA	No	NA	NA	NA	NA
Gdist:S3060_4779	BT12	54.626	0.0079	NA	No	No	No	No	No	No	No	NA	NA	No	NA	NA	NA	NA
Gdist:S317586_2473	BT12	54.626	0.0047	NA	No	No	No	No	No	No	No	NA	NA	No	NA	NA	NA	NA
Gdist:S508336_3668	BT12	54.626	-0.0014	NA	No	No	No	No	No	No	No	NA	NA	No	NA	NA	NA	NA
Gdist:S306571_3728	BT12	54.627	0.0316	NA	No	No	No	No	No	No	No	0.669157175	0.21798387	Yes	NA	NA	NA	NA
Gdist:S306571_4010	BT12	54.628	0.0307	NA	No	No	No	No	No	No	No	0.633486683	0.3285906	Yes	NA	NA	NA	NA
Gdist:S306571_6855	BT12	54.742	0.005	NA	No	No	No	No	No	No	No	NA	NA	No	NA	NA	NA	NA
SalarSNP:ESTNV_32045_280	BT12	54.857	0.0261	NA	No	No	No	No	No	No	No	NA	NA	No	NA	NA	NA	NA
cDNA:S670900_2441	BT12	54.964	0.0033	NA	No	No	No	No	No	No	No	NA	NA	No	NA	NA	NA	NA
Gene:S474031_795	BT12	55.636	0.0168	NA	No	No	No	No	No	No	No	NA	NA	No	NA	NA	NA	NA
Gdist:S369351_5216	BT12	57.007	-0.0123	NA	No	No	No	No	No	No	No	NA	NA	No	NA	NA	NA	NA
Gdist:S196369_3759	BT12	57.134	0.009	NA	No	No	No	No	No	Yes (0.002)	Yes (0.024)	NA	NA	No	NA	NA	NA	NA
Gdist:S618113_2649	BT12	57.271	0.0103	NA	No	No	No	No	No	No	No	NA	NA	No	NA	NA	NA	NA
Gdist:S618113_6185	BT12	57.272	0.0048	NA	No	No	No	No	No	No	No	NA	NA	No	NA	NA	NA	NA
cDNA:S242245_7699	BT12	57.908	0.0144	NA	No	No	No	No	No	No	No	NA	NA	No	NA	NA	NA	NA
Gdist:S211349_7251	BT12	58.438	0.0387	NA	No	No	No	No	No	No	No	0.396666667	0.37649215	Yes	NA	NA	NA	NA
Gdist:S20722_9954	BT12	58.438	0.0101	NA	No	No	No	No	No	No	No	NA	NA	No	NA	NA	NA	NA
Gdist:S211349_7837	BT12	58.438	0.01	NA	No	No	No	No	No	No	No	NA	NA	No	NA	NA	NA	NA
SalHit:S273164_1801	BT12	58.438	0.0046	NA	No	No	No	No	No	No	No	NA	NA	No	NA	NA	NA	NA
Gdist:S469005_1441	BT12	58.438	0.0031	NA	No	No	No	No	No	No	No	NA	NA	No	NA	NA	NA	NA
SalHit:S621605_4762	BT12	58.438	-0.0008	NA	No	No	No	No	No	No	No	NA	NA	No	NA	NA	NA	NA
Gdist:S115441_7423	BT12	58.438	-0.0065	NA	No	No	No	No	No	No	No	NA	NA	No	NA	NA	NA	NA
Gdist:S100973_6375	BT12	58.439	-0.0116	NA	No	No	No	No	No	No	No	NA	NA	No	NA	NA	NA	NA
Gdist:S171753_8933	BT12	58.44	0.0267	NA	No	No	No	No	No	Yes (0.002)	Yes (0.024)	NA	NA	No	NA	NA	NA	NA
cDNA:S78668_2682	BT12	58.452	0.0458	NA	No	No	No	No	No	No	No	0.220947205	0.2295	Yes	NA	NA	NA	NA
SalHit:S54277_3619	BT12	59.401	0.0024	NA	No	No	No	No	No	No	No	NA	NA	No	NA	NA	NA	NA
cDNA:S170395_4015	BT13	0	0.0117	NA	No	No	No	No	No	No	No	NA	NA	No	NA	NA	NA	NA
cDNA:S183371_1071	BT13	2.974	0.037	NA	No	No	No	No	No	No	No	0.874285714	0.84677586	Yes	NA	NA	NA	NA
cDNA:S550666_852	BT13	4.262	0.0023	NA	No	No	No	No	No	No	No	NA	NA	No	NA	NA	NA	NA
SalHit:S91307_4577	BT13	4.264	0.0381	NA	No	No	No	No	No	No	No	0.676930023	0.17126866	Yes	NA	NA	NA	NA
SalHit:S91307_10080	BT13	4.264	-0.0002	NA	No	No	No	No	No	No	No	NA	NA	No	NA	NA	NA	NA
cDNA:S741878_741	BT13	8.603	0.0144	NA	No	No	No	No	No	No	No	NA	NA	No	NA	NA	NA	NA
Gdist:S272921_2755	BT13	9.11	0.0106	NA	No	No	No	No	No	No	No	NA	NA	No	NA	NA	NA	NA
Gdist:S86152_756	BT13	9.11	-0.0017	NA	Yes	No	No	No	No	No	No	NA	NA	No	NA	NA	NA	NA
Gdist:S272921_6816	BT13	9.11	-0.0073	NA	No	No	No	No	No	No	No	NA	NA	No	NA	NA	NA	NA
cDNA:S549885_1391	BT13	9.11	-0.0097	NA	No	No	No	No	No	No	No	NA	NA	No	NA	NA	NA	NA
cDNA:S255992_1631	BT13	10.498	0.0146	NA	No	No	No	No	No	No	No	NA	NA	No	NA	NA	NA	NA
Gdist:S10257_3175	BT13	13.335	0.0223	NA	No	No	No	No	No	No	No	NA	NA	No	NA	NA	NA	NA
Gdist:S192779_5715	BT13	13.36	0.0071	NA	No	No	No	No	No	No	No	NA	NA	No	NA	NA	NA	NA
Gdist:S132906_5083	BT13	13.45	0.0116	NA	No	No	No	No	No	No	No	NA	NA	No	NA	NA	NA	NA
Gdist:S495400_7369	BT13	13.459	0.024	NA	No	No	No	No	No	No	No	NA	NA	No	NA	NA	NA	NA
Gdist:S160069_12300	BT13	13.469	0.0228	NA	No	No	No	No	No	No	No	NA	NA	No	NA	NA	NA	NA
cDNA:S287314_2130	BT13	15.043	0.0185	NA	No	No	No	No	No	No	No	NA	NA	No	NA	NA	NA	NA
cDNA:S326435_12628	BT13	15.043	0.0123	NA	No	No	No	No	No	No	No	NA	NA	No	NA	NA	NA	NA
cDNA:S370810_3013	BT13	15.043	-0.0028	NA	No	No	No	No	No	No	No	NA	NA	No	NA	NA	NA	NA
Gdist:S349813_6865	BT13	15.46	0.014	NA	No	No	No	No	No	No	No	NA	NA	No	NA	NA	NA	NA
Gdist:S360039_2326	BT13	15.873	0.0333	NA	No	No	No	No	No	No	No	0.519019293	0.90616071	Yes	NA	NA	NA	NA
SalarSNP:ESTV_15080_392	BT13	15.874	0.0186	NA	No	No	No	No	No	No	No	NA	NA	No	NA	NA	NA	NA

cDNA:S153293_2845	BT13	15.876	0.0033	NA	No	No	No	No	No	No	No	NA	NA	No	NA	NA	NA	NA
Gdist:S79020_5844	BT13	17.863	0.0529	NA	No	No	No	No	No	No	No	0.565759053	0.56791921	Yes	NA	NA	NA	NA
cDNA:S82741_15301	BT13	17.863	0.007	NA	No	No	No	No	No	No	No	NA	NA	No	NA	NA	NA	NA
cDNA:S82741_16645	BT13	17.863	-0.0023	NA	No	No	No	No	No	No	No	NA	NA	No	NA	NA	NA	NA
SalHit:S357188_2413	BT13	20.143	-0.0048	NA	Yes	No	No	No	No	No	No	NA	NA	No	NA	NA	NA	NA
cDNA:S279973_7852	BT13	21.724	0.0087	NA	No	No	No	No	No	No	No	NA	NA	No	NA	NA	NA	NA
Gene:S92009_2784	BT13	21.725	0.0312	NA	No	No	No	No	No	No	No	0.862473638	0.72269585	Yes	NA	NA	NA	NA
cDNA:S507443_10507	BT13	22.823	0.0058	NA	No	No	No	No	No	No	No	NA	NA	No	NA	NA	NA	NA
cDNA:S507443_10963	BT13	22.826	0.0188	NA	No	No	No	No	No	No	No	NA	NA	No	NA	NA	NA	NA
Gdist:S57705_8304	BT13	22.829	0.0456	NA	No	No	No	No	No	No	No	0.743034653	0.21798387	Yes	NA	NA	NA	NA
Gdist:S485238_4860	BT13	22.832	0.0167	NA	No	No	No	No	No	No	No	NA	NA	No	NA	NA	NA	NA
SalHit:S46568_10274	BT13	24.489	0.0045	NA	No	No	No	No	No	No	No	NA	NA	No	NA	NA	NA	NA
cDNA:S651830_6088	BT13	26.724	0.0115	NA	No	No	No	No	No	No	No	NA	NA	No	NA	NA	NA	NA
Gdist:S339767_9556	BT13	28.96	0.002	NA	Yes	No	No	No	No	No	No	NA	NA	No	NA	NA	NA	NA
Gdist:S143250_7038	BT13	30.574	0.0215	NA	No	No	No	No	No	Yes (0.013)	No	NA	NA	No	NA	NA	NA	NA
SalHit:S76349_7595	BT13	30.574	0.0167	NA	No	No	No	No	No	No	No	NA	NA	No	NA	NA	NA	NA
cDNA:S726233_1591	BT13	30.574	0.011	NA	No	No	No	No	No	Yes (0.037)	No	NA	NA	No	NA	NA	NA	NA
Gdist:S362726_6078	BT13	30.574	0.0104	NA	No	No	No	No	No	No	No	NA	NA	No	NA	NA	NA	NA
Gdist:S373163_2756	BT13	30.574	0.0074	NA	No	No	No	No	No	No	No	NA	NA	No	NA	NA	NA	NA
Gdist:S362726_3535	BT13	30.574	0.0069	NA	No	No	No	No	No	No	No	NA	NA	No	NA	NA	NA	NA
Gdist:S286836_5084	BT13	30.574	0.0023	NA	No	No	No	No	No	No	No	NA	NA	No	NA	NA	NA	NA
Gdist:S242933_769	BT13	30.574	0.0002	NA	No	No	No	No	No	No	No	NA	NA	No	NA	NA	NA	NA
Gdist:S24211_4997	BT13	30.574	-0.0041	NA	No	No	No	No	No	No	No	NA	NA	No	NA	NA	NA	NA
Gdist:S170073_6407	BT13	30.574	0.0397	NA	No	No	No	No	No	No	No	0.650339578	0.0306	Yes	SKJ INC	NA	increased	yes
cDNA:S41119_1719	BT13	30.841	0.0562	PREDIC'	No	No	No	No	No	No	No	0.735576923	0.01821429	Yes	SKJ RED	NA	reduced	yes
cDNA:S122885_4254	BT13	31.048	0.0161	NA	No	No	No	No	No	No	No	NA	NA	No	NA	NA	NA	NA
cDNA:S807823_507	BT13	31.304	0.0203	NA	No	No	No	No	No	No	No	NA	NA	No	NA	NA	NA	NA
cDNA:S150821_5852	BT13	33.078	0.0075	NA	No	No	No	No	No	Yes (0.007)	Yes (0.049)	NA	NA	No	NA	NA	NA	NA
cDNA:S150821_9294	BT13	33.095	0.0137	NA	No	No	No	No	No	No	No	NA	NA	No	NA	NA	NA	NA
SalHit:S126310_347	BT13	33.218	0.009	NA	No	No	No	No	No	No	No	NA	NA	No	NA	NA	NA	NA
Gdist:S420000_6807	BT13	33.529	-0.0045	NA	No	No	No	No	No	No	No	NA	NA	No	NA	NA	NA	NA
Gdist:S221163_7658	BT13	34.352	0.0256	NA	No	No	No	No	No	No	No	NA	NA	No	NA	NA	NA	NA
Gdist:S71571_6516	BT13	34.468	0.0095	NA	No	No	No	No	No	Yes (0.043)	No	NA	NA	No	NA	NA	NA	NA
Gdist:S30075_4166	BT13	34.468	0.003	NA	No	No	No	No	No	No	No	NA	NA	No	NA	NA	NA	NA
cDNA:S76720_2288	BT13	34.468	-0.0041	NA	No	No	No	No	No	No	No	NA	NA	No	NA	NA	NA	NA
Gdist:S30075_6826	BT13	34.468	-0.0043	NA	No	No	No	No	No	No	No	NA	NA	No	NA	NA	NA	NA
cDNA:S345968_9777	BT13	35.43	0.0106	NA	No	No	No	No	No	No	No	NA	NA	No	NA	NA	NA	NA
SalHit:S244701_3876	BT13	37.029	0.0277	NA	No	No	No	No	No	No	No	NA	NA	No	NA	NA	NA	NA
Gene:S695148_1062	BT13	37.029	-0.0132	NA	Yes	No	No	No	No	No	No	NA	NA	No	NA	NA	NA	NA
Gdist:S25131_8621	BT13	37.489	0.0212	NA	No	No	No	No	No	No	No	NA	NA	No	NA	NA	NA	NA
Gdist:S547283_2834	BT13	37.897	-0.0044	NA	No	No	No	No	No	No	No	NA	NA	No	NA	NA	NA	NA
Gdist:S26610_2904	BT13	37.939	0.0889	NA	Yes	No	No	No	No	No	No	0.370959052	0.28322519	Yes	NA	NA	NA	NA
Gdist:S258756_5187	BT13	37.939	0.0461	NA	Yes	No	No	No	No	No	No	0.735576923	0.80233566	Yes	NA	NA	NA	NA
Gdist:S141935_5679	BT13	37.939	0.046	NA	Yes	No	No	No	No	Yes (0.004)	Yes (0.035)	0.870811856	0.88358108	Yes	NA	NA	NA	NA
Gdist:S61089_5650	BT13	37.939	0.0403	NA	No	No	No	No	No	No	No	0.949902597	0.65440981	Yes	NA	NA	NA	NA
Gdist:S765417_6956	BT13	37.939	0.0287	NA	No	No	No	Yes (0.02)	No	No	No	NA	NA	No	NA	NA	NA	NA
LD:S258756_18380	BT13	37.939	0.0148	NA	No	No	No	No	No	No	No	NA	NA	No	NA	NA	NA	NA
cDNA:S838704_1071	BT13	37.939	0.0113	NA	No	No	No	No	No	No	No	NA	NA	No	NA	NA	NA	NA
LD:S258756_9572	BT13	37.939	-0.007	NA	No	No	No	No	No	No	No	NA	NA	No	NA	NA	NA	NA
cDNA:S702119_7663	BT13	37.951	0.0161	NA	No	No	No	No	No	No	No	NA	NA	No	NA	NA	NA	NA
cDNA:S73079_6850	BT13	38.837	0.0091	NA	Yes	No	No	No	No	Yes (0.006)	Yes (0.042)	NA	NA	No	NA	NA	NA	NA
cDNA:S619969_5417	BT13	39.481	0.0338	NA	No	No	No	No	No	Yes (0.038)	No	0.982691275	0.48116071	Yes	NA	NA	NA	NA
SalHit:S394519_1767	BT13	39.481	0.0111	NA	No	No	No	No	No	No	No	NA	NA	No	NA	NA	NA	NA
cDNA:S829676_1368	BT13	39.481	0.0096	NA	No	No	No	No	No	No	No	NA	NA	No	NA	NA	NA	NA
Gdist:S16798_7124	BT13	39.481	0.0085	NA	No	No	No	No	No	No	No	NA	NA	No	NA	NA	NA	NA
Gdist:S16798_5659	BT13	39.481	-0.0013	NA	No	No	No	No	No	No	No	NA	NA	No	NA	NA	NA	NA
Gdist:S210273_6426	BT13	39.666	0.015	NA	Yes	No	No	No	No	No	No	0.75075	0.1051875	Yes	NA	NA	NA	NA
Gdist:S264488_6320	BT13	39.858	0.0106	NA	No	No	No	No	No	No	No	NA	NA	No	NA	NA	NA	NA
cDNA:S591136_2909	BT13	39.858	-0.0052	NA	No	No	No	No	No	No	No	NA	NA	No	NA	NA	NA	NA
Gdist:S264488_9807	BT13	39.858	-0.0099	NA	Yes	No	No	No	No	No	No	NA	NA	No	NA	NA	NA	NA
SalarSNP:ESTNV_30342_1325	BT13	39.858	0.0799	NA	No	No	No	No	No	Yes (0.006)	Yes (0.042)	0.028867925	0.60417614	Yes	VAR INC	increased	NA	NA
Gdist:S104914_2923	BT13	39.88	0.0294	NA	No	No	No	No	No	No	No	NA	NA	No	NA	NA	NA	NA
Gdist:S666683_1774	BT13	40.486	0.0737	NA	No	No	No	No	No	No	No	0.139695652	0.9833502	Yes	NA	NA	NA	NA
Gdist:S666683_5670	BT13	40.486	0.0378	NA	Yes	No	No	No	No	No	No	0.682433921	0.53043554	Yes	NA	NA	NA	NA
Gdist:S250874_7670	BT13	40.487	0.0347	NA	No	No	No	No	No	Yes (0)	Yes (0.003)	0.385661157	0.41482394	Yes	NA	NA	NA	NA
Gdist:S250874_4561	BT13	40.489	-0.0043	NA	No	No	No	No	No	No	No	NA	NA	No	NA	NA	NA	NA
Gdist:S248163_1782	BT13	40.513	-0.0003	NA	No	No	No	No	No	No	No	NA	NA	No	NA	NA	NA	NA
Gdist:S68597_4677	BT13	43.58	-0.0127	NA	No	No	No	No	No	No	No	NA	NA	No	NA	NA	NA	NA
Gdist:S91895_4247	BT13	43.687	0.0198	NA	No	No	No	No	No	Yes (0.021)	No	0.901027732	0.41482394	Yes	NA	NA	NA	NA
Gdist:S67501_7028	BT13	44.426	-0.0073	NA	No	No	No	No	No	No	No	NA	NA	No	NA	NA	NA	NA
cDNA:S126541_3351	BT13	44.62	0.0011	NA	No	No	No	No	No	No	No	NA	NA	No	NA	NA	NA	NA
Gdist:S58119_5057	BT13	44.805	0.0139	NA	No	No	No	No	No	No	No	NA	NA	No	NA	NA	NA	NA
cDNA:S807177_306	BT13	44.805	0.0114	NA	No	No	No	No	No	Yes (0.001)	Yes (0.01)	NA	NA	No	NA	NA	NA	NA
cDNA:S256656_7787	BT13	44.805	0.0031	NA	No	No	No	No	No	No	No	NA	NA	No	NA	NA	NA	NA
cDNA:S318858_10221	BT13	44.805	0.0019	NA	No	No	No	No	No	No	No	NA	NA	No	NA	NA	NA	NA
Gdist:S458609_9395	BT13	44.811	0.0321	NA	No	No	No	No	No	No	No	0.97625523	0.69352273	Yes	NA	NA	NA	NA
SalHit:S51948_4671	BT13	46.173	-0.0007	NA	No	No	No	No	No	No	No	NA	NA	No	NA	NA	NA	NA
SalHit:S51948_2505	BT13	46.179	0.0007	NA	No	No	No	No	No	No	No	NA	NA	No	NA	NA	NA	NA
cDNA:S59871_2796	BT13	46.186	0.0176	NA	No	No	No	No	No	No	No	NA	NA	No	NA	NA	NA	NA
Gdist:S815659_6763	BT13	46.186	0.0154	NA	No	No	No	No	No	No	No	NA	NA	No	NA	NA	NA	NA
cDNA:S569434_1910	BT13	46.533	-0.0029	NA	No	No	No	No	No	No	No	NA	NA	No	NA	NA	NA	NA
cDNA:S487087_629	BT13	46.931	0.0208	NA	No	No	No	No	No	No	No	0.218915094	0.69186047	Yes	NA	NA	NA	NA
Gdist:S94591_1508	BT13	46.959	-0.0022	NA	No	No	No	No	No	No	No	NA	NA	No	NA	NA	NA	NA
cDNA:S755749_1729	BT13	46.987	0.0125	NA	No	No	No	No	No	No	No	0.774525862	0.3565678	Yes	NA	NA	NA	NA
Gdist:S46231_7252	BT13	47.014	-0.003	NA	No	No	No	No	No	No	No	NA	NA	No	NA	NA	NA	NA
Gdist:S26026_10676	BT13	47.043	0.009	NA	No	No	No	No	No	No	No	NA	NA	No	NA	NA	NA	NA
Gdist:S30112_3479	BT13	47.069	0.0464	NA	No	No	No	No	No	Yes (0.008)	No	0.380912863	0.074375	Yes	NA	NA	NA	NA
cDNA:S239119_2637	BT13	47.069	0.0312	NA	No	No	No	No	No	No	No	0.682433921	0.79436364	Yes	NA	NA	NA	NA
cDNA:S310740_5108	BT13	47.069	0.0204	NA	No	No	No	No	No	No	No	NA	NA	No	NA	NA	NA	NA
cDNA:S415746_2653	BT13	47.069	0.0113	NA	No	No	No	No	No	No	No	NA	NA	No	NA	NA	NA	NA
SalHit:S166885_4145	BT13	47.069	0.0084	NA	No	No	No	No	No	No	No	NA	NA	No	NA	NA	NA	NA
cDNA:S310740_4879	BT13	47.069	0.0062	NA	No	No	No	No	No	No	No	NA	NA	No	NA	NA	NA	NA
Gdist:S744590_3769	BT13	47.069	0.0015	NA	No	No	No	No	No	No	No	NA	NA	No	NA	NA	NA	NA
Gdist:S503196_3759	BT13	47.069	0.0007	NA	No	No	No	No	No	No	No	NA	NA	No	NA	NA	NA	NA
Gdist:S503196_7657	BT13	47.069	-0.0003	NA	No	No	No	No	No	No	No	NA	NA	No	NA	NA	NA	NA
cDNA:S310740_8498	BT13	47.069	-0.009	NA	Yes	No	No	No	No	No	No	NA	NA	No	NA	NA	NA	NA
SalHit:S401259_10429	BT13	47.069	-0.0133	NA	No	No	No	No	No	No	No	NA	NA	No	NA	NA	NA	NA
Gdist:S216680_5599	BT13	47.072	0.0105	NA	No	No	No	No	No	Yes (0.012)	No	NA	NA	No	NA	NA	NA	NA
cDNA:S443930_1034	BT13	47.077	0.0031	NA	No	No	No	No	No	No	No	NA	NA	No	NA	NA	NA	NA
Gdist:S193124_6201	BT13	47.081	0.0092	NA	No	No	No	No	No	No	No	NA	NA	No	NA	NA	NA	NA
cDNA:S606420_2644	BT13	47.313	0.0021	NA	No	No	No	No	No	Yes (0.037)	No	NA	NA	No	NA	NA	NA	NA
LD:S107235_2148	BT13	47.54	0.0291	NA	No	No	No	No	No	No	No	NA	NA	No	NA	NA	NA	NA
cDNA:S448226_3940	BT13	47.54	0.011	NA	No	No	No	No	No	No	No	NA	NA	No	NA	NA	NA	NA
Gdist:S107235_2071	BT13	47.54	0.01	NA	Yes	No	No	No	No	No	No	NA	NA	No	NA	NA	NA	NA
cDNA:S220067_3688	BT13	48.095	0.0202	NA	No	No	No	No	No	Yes (0.007)	Yes (0.049)	NA	NA	No	NA	NA	NA	NA
SalHit:S52170_270	BT14	0	0.1018	NA	No	No	No	No	No	No	No	0.093590426	0.374	Yes	NA	NA	NA	NA
Gdist:S95102_3370	BT14	0	0.0189	NA	No	No	No	No	No	No	No	NA	NA	No	NA	NA	NA	NA
cDNA:C123299019_115	BT14	0	-0.0045	NA	No	No	No	No	No	No	No	NA	NA	No	NA	NA	NA	NA
Gdist:S156572_1700	BT14	0	-0.0084	NA	No	No	No	No	No	No	No	NA	NA	No	NA	NA	NA	NA
cDNA:S118837_6577	BT14	0.014	-0.0085	NA	No	No	No	No	No	No	No	NA	NA	No	NA	NA	NA	NA
Gdist:S49472_3963	BT14	1.922	-0.0058	NA	No	No	No	No	No	No	No	NA	NA	No	NA	NA	NA	NA
Gdist:S197557_3245	BT14	1.956	0.0319	NA	No	No	No	No	No	No	No	0.911143868	0	Yes	SKJ INC	NA	increased -	NA
cDNA:S93807_4051	BT14	1.985	0.0096	NA	No	No	No	No	No	No	No	NA	NA	No	NA	NA	NA	NA
Gdist:S41698_10566	BT14	2.026	0.0097	NA	No	No	No	No	No	No	No	NA	NA	No	NA	NA	NA	NA
Gdist:S41698_6798	BT14	2.255	0.0058	NA	No	No	No	No	No	No	No	NA	NA	No	NA	NA	NA	NA
Gdist:S7620_3244	BT14	8.619	0.0536	NA	No	No	No	No	No	No	No	0.639010664	0.56791921	Yes	NA	NA	NA	NA
Gdist:S7620_5752	BT14	8.655	0.0044	NA	No	No	No	No	No	No	No	NA	NA	No	NA	NA	NA	NA
Gdist:S323064_1442	BT14	14.145	0.0053	NA	No	No	No	No	No	No	No	NA	NA	No	NA	NA	NA	NA
Gdist:S323064_2014	BT14	14.185	0.0234	NA	Yes	No	No	No	No	No	No	NA	NA	No	NA	NA	NA	NA
Gdist:S107142_7279	BT14	17.512	0.034	NA	No	No	No	No	No	No	No	0.262581081	0.40849515	Yes	NA	NA	NA	NA
Gdist:S107142_234	BT14	17.512	0.0751	none	No	No	No	No	No	No	No	0.96671108	0.01821429	Yes	SKJ RED	NA	reduced	yes
cDNA:S353186_3074	BT14	18.56	-0.0006	NA	No	No	No	No	No	No	No	NA	NA	No	NA	NA	NA	NA
SalarSNP:ESTNV_35477_857	BT14	18.576	0.0051	NA	No	No	No	No	No	No	No	NA	NA	No	NA	NA	NA	NA
LD:S304413_9219	BT14	23.216	0.0579	NA	No	No	No	No	No	No	No	0.197937063	0.17485714	Yes	NA	NA	NA	NA
cDNA:S130742_2245	BT14	23.216	0.0381	NA	Yes	No	No	No	No	No	No	0.41769	0.42304147	Yes	NA	NA	NA	NA
Gdist:S660344_1826	BT14	23.216	0.0183	NA	No	No	No	No	No	No	No	NA	NA	No	NA	NA	NA	NA
LD:S304413_928	BT14	23.216	0.0037	NA	No	No	No	No	No	No	No	NA	NA	No	NA	NA	NA	NA

Gdist:S660344_6695	BT14	23.216	0.0028	NA	No	No	No	No	No	No	No	NA	NA	No	NA	NA	NA	NA
Gdist:S482911_4853	BT14	29.46	0.0279	NA	No	No	No	No	No	No	No	NA	NA	No	NA	NA	NA	NA
Gdist:S379720_1425	BT14	30.861	0.0003	NA	No	No	No	No	No	No	No	NA	NA	No	NA	NA	NA	NA
Gdist:S67978_6355	BT14	30.914	0.0421	NA	Yes	No	No	No	No	No	No	0.419853516	0.60712604	Yes	NA	NA	NA	NA
LD:S109921_874	BT14	30.931	0.0216	NA	No	No	No	No	No	No	No	NA	NA	No	NA	NA	NA	NA
LD:S109921_13369	BT14	30.937	0.013	NA	No	No	No	No	No	No	No	NA	NA	No	NA	NA	NA	NA
Gdist:S109921_14334	BT14	30.951	0.0245	NA	No	No	No	No	No	No	No	NA	NA	No	NA	NA	NA	NA
Gdist:S511299_5345	BT14	30.964	0.0089	NA	No	No	No	No	No	No	No	NA	NA	No	NA	NA	NA	NA
Gdist:S31082_2578	BT14	32.613	0.0035	NA	No	No	No	No	No	Yes (0.035)	No	NA	NA	No	NA	NA	NA	NA
Gdist:S238631_3607	BT14	32.656	-0.0021	NA	No	No	No	No	No	No	No	NA	NA	No	NA	NA	NA	NA
cDNA:S513732_2662	BT14	34.417	0.0025	NA	No	No	No	No	No	Yes (0.007)	Yes (0.049)	NA	NA	No	NA	NA	NA	NA
cDNA:S2274_967	BT14	34.47	0.0096	NA	No	No	No	No	No	No	No	NA	NA	No	NA	NA	NA	NA
Gdist:S240092_6180	BT14	34.471	0.0494	NA	No	No	No	No	No	No	No	0.233502907	0.60417614	Yes	NA	NA	NA	NA
cDNA:S2274_506	BT14	34.471	0.0147	NA	No	No	No	No	No	Yes (0.007)	Yes (0.048)	NA	NA	No	NA	NA	NA	NA
Gdist:S198389_5261	BT14	34.471	0.0042	NA	No	No	No	No	No	No	No	NA	NA	No	NA	NA	NA	NA
Gdist:S44486_8674	BT14	34.959	0.0034	NA	No	No	No	No	No	No	No	NA	NA	No	NA	NA	NA	NA
Gdist:S44486_4530	BT14	35.478	-0.0111	NA	No	No	No	No	No	No	No	NA	NA	No	NA	NA	NA	NA
Gdist:S54834_6997	BT14	36.105	-0.0051	NA	No	No	No	No	No	No	No	NA	NA	No	NA	NA	NA	NA
SalHit:S661277_5664	BT14	38.671	0.0045	NA	No	No	No	No	No	Yes (0.007)	Yes (0.049)	NA	NA	No	NA	NA	NA	NA
Gdist:S195477_14645	BT14	38.671	0.0012	NA	No	No	No	No	No	No	No	NA	NA	No	NA	NA	NA	NA
Gdist:S386457_9386	BT14	38.671	0.0005	NA	No	No	No	No	No	No	No	NA	NA	No	NA	NA	NA	NA
cDNA:S160993_9051	BT14	39.23	0.0756	NA	No	No	No	No	No	No	No	0.079862637	0.21798387	Yes	NA	NA	NA	NA
cDNA:S160993_8558	BT14	39.23	0.0114	NA	No	No	No	No	No	No	No	NA	NA	No	NA	NA	NA	NA
Gdist:S53541_2096	BT14	39.231	0.0086	NA	No	No	No	No	No	Yes (0.007)	Yes (0.049)	NA	NA	No	NA	NA	NA	NA
Gdist:S116498_6891	BT14	41.276	-0.0085	NA	No	No	No	No	No	No	No	NA	NA	No	NA	NA	NA	NA
cDNA:S495254_1113	BT14	42.755	0.0002	NA	No	No	No	No	No	No	No	NA	NA	No	NA	NA	NA	NA
cDNA:S169037_3948	BT14	43.54	0.0033	NA	No	No	No	No	No	No	No	NA	NA	No	NA	NA	NA	NA
SalHit:S49260_1709	BT14	43.591	0.0281	NA	No	No	No	No	No	No	No	NA	NA	No	NA	NA	NA	NA
Gdist:S457503_10471	BT14	43.6	-0.0026	NA	No	No	No	No	No	No	No	NA	NA	No	NA	NA	NA	NA
Gdist:S250383_6156	BT14	43.646	-0.0047	NA	No	No	No	No	No	No	No	NA	NA	No	NA	NA	NA	NA
Gdist:S84103_5394	BT14	45.747	0.0077	NA	No	No	No	No	No	Yes (0.046)	No	NA	NA	No	NA	NA	NA	NA
SalHit:S643886_6448	BT14	47.894	0.0075	NA	No	No	No	No	No	No	No	NA	NA	No	NA	NA	NA	NA
Gdist:S93760_6902	BT14	47.97	-0.0052	NA	No	No	No	No	No	No	No	NA	NA	No	NA	NA	NA	NA
Gdist:S129244_13486	BT14	48.379	0.0073	NA	No	No	No	No	No	No	No	NA	NA	No	NA	NA	NA	NA
cDNA:S666270_3095	BT14	49.242	0.0212	NA	No	No	No	No	No	No	No	NA	NA	No	NA	NA	NA	NA
SalarSNP:ESTNV_32552_113	BT14	56.368	-0.0008	NA	No	No	No	No	No	No	No	NA	NA	No	NA	NA	NA	NA
cDNA:S739620_1210	BT15	0	0.0048	NA	No	No	No	No	No	No	No	NA	NA	No	NA	NA	NA	NA
cDNA:S76557_5098	BT15	0.88	0.0271	NA	No	No	No	No	No	No	No	0.214394904	0.20131579	Yes	NA	NA	NA	NA
SalHit:S605138_705	BT15	7.107	0.0257	NA	No	No	No	No	No	No	No	NA	NA	No	NA	NA	NA	NA
LD:S19938_1411	BT15	12.331	0.0045	NA	No	No	No	No	No	No	No	NA	NA	No	NA	NA	NA	NA
cDNA:S187071_1047	BT15	14.061	0.0116	NA	No	No	No	No	No	No	No	NA	NA	No	NA	NA	NA	NA
Gdist:S26670_2535	BT15	22.979	0.018	NA	No	No	No	No	No	No	No	NA	NA	No	NA	NA	NA	NA
Gdist:S8036_3287	BT15	25.573	0.0255	NA	No	No	No	No	No	No	No	NA	NA	No	NA	NA	NA	NA
SalHit:S214652_2227	BT15	25.573	0.005	NA	No	No	No	No	No	No	No	NA	NA	No	NA	NA	NA	NA
cDNA:S150356_541	BT15	26.136	0.0004	NA	No	No	No	No	No	No	No	NA	NA	No	NA	NA	NA	NA
SalHit:S119971_1271	BT15	27.343	0.0232	NA	No	No	No	No	No	No	No	NA	NA	No	NA	NA	NA	NA
cDNA:S64643_2871	BT15	28.511	0.0251	NA	Yes	No	No	No	No	No	No	NA	NA	No	NA	NA	NA	NA
Gdist:S6243_5446	BT15	28.592	-0.006	NA	No	No	No	No	No	No	No	NA	NA	No	NA	NA	NA	NA
Gdist:S358285_1092	BT15	31.727	0.0026	NA	No	No	No	No	No	No	No	NA	NA	No	NA	NA	NA	NA
SalHit:S49089_986	BT15	31.734	0.0092	NA	No	No	No	No	No	Yes (0)	Yes (0.007)	NA	NA	No	NA	NA	NA	NA
Gdist:S247256_2512	BT15	31.74	0.0271	NA	Yes	No	No	No	No	No	No	NA	NA	No	NA	NA	NA	NA
SalHit:S280657_1162	BT15	52.079	-0.0063	NA	No	No	No	No	No	No	No	NA	NA	No	NA	NA	NA	NA
cDNA:S301357_9739	BT16	0	-0.0111	NA	No	No	No	No	No	Yes (0.015)	No	NA	NA	No	NA	NA	NA	NA
cDNA:S625101_1079	BT16	0.235	0.0011	NA	No	No	No	No	No	No	No	NA	NA	No	NA	NA	NA	NA
Gdist:S113620_6102	BT16	3.896	0.0047	NA	No	No	No	No	No	No	No	NA	NA	No	NA	NA	NA	NA
cDNA:S360008_1889	BT16	4.125	0.0091	NA	No	No	No	No	No	No	No	NA	NA	No	NA	NA	NA	NA
Gdist:S39401_4373	BT16	5.203	-0.0099	NA	No	No	No	No	No	No	No	NA	NA	No	NA	NA	NA	NA
Gdist:S237828_5646	BT16	8.878	0.0108	NA	No	No	No	No	No	No	No	NA	NA	No	NA	NA	NA	NA
Gdist:S56173_1211	BT16	9.357	-0.0077	NA	No	No	No	No	No	No	No	NA	NA	No	NA	NA	NA	NA
Gdist:S8497_3287	BT16	9.513	0.0059	NA	No	No	No	No	No	No	No	NA	NA	No	NA	NA	NA	NA
Gdist:S106782_8017	BT16	13.738	0.0084	NA	No	No	No	No	No	No	No	NA	NA	No	NA	NA	NA	NA
Gdist:S79088_5202	BT16	14.179	-0.0041	NA	No	No	No	No	No	Yes (0.048)	No	NA	NA	No	NA	NA	NA	NA
cDNA:S624913_741	BT16	15.182	0.0488	NA	No	No	No	No	No	No	No	0.299431818	0.73333893	Yes	NA	NA	NA	NA
Gdist:S453561_5410	BT16	18.075	0.0633	NA	No	No	No	No	No	No	No	0.178308271	0.59462389	Yes	NA	NA	NA	NA
cDNA:S118434_5718	BT16	18.08	-0.0047	NA	No	No	No	No	No	No	No	NA	NA	No	NA	NA	NA	NA
Gdist:S611098_8555	BT16	18.08	-0.0065	NA	No	No	No	No	No	No	No	NA	NA	No	NA	NA	NA	NA
Gdist:S120141_2338	BT16	18.082	0.0124	NA	Yes	No	No	No	No	No	No	NA	NA	No	NA	NA	NA	NA
cDNA:S96224_8004	BT16	18.757	-0.0145	NA	No	No	No	No	No	No	No	NA	NA	No	NA	NA	NA	NA
cDNA:S212652_2299	BT16	19.484	-0.0096	NA	No	No	No	No	No	No	No	NA	NA	No	NA	NA	NA	NA
Gdist:S273721_9684	BT16	23.554	0.0131	NA	No	No	No	No	No	No	No	NA	NA	No	NA	NA	NA	NA
Gdist:S273721_4337	BT16	23.554	0.0022	NA	No	No	No	No	No	No	No	NA	NA	No	NA	NA	NA	NA
Gdist:S18807_5080	BT16	25.082	0.0046	NA	No	No	No	No	No	No	No	NA	NA	No	NA	NA	NA	NA
SalHit:S797343_7313	BT16	25.082	-0.0028	NA	No	No	No	No	No	No	No	NA	NA	No	NA	NA	NA	NA
LD:S410327_13451	BT16	25.17	0.0388	NA	No	No	No	No	No	Yes (0.043)	No	0.205182119	0.11839286	Yes	NA	NA	NA	NA
Gdist:S2610_9039	BT16	26.685	0.0293	NA	No	No	No	No	No	No	No	NA	NA	No	NA	NA	NA	NA
cDNA:S418182_2800	BT16	26.695	-0.002	NA	No	No	No	No	No	No	No	NA	NA	No	NA	NA	NA	NA
LD:S158422_2767	BT16	31.162	0.0058	NA	Yes	No	No	No	No	No	No	NA	NA	No	NA	NA	NA	NA
Gdist:S188677_4401	BT16	31.162	-0.0021	NA	No	No	No	No	No	No	No	NA	NA	No	NA	NA	NA	NA
Gdist:S188677_7042	BT16	31.162	0.0378	NA	No	No	No	No	No	No	No	0.0095625	0.80233566	Yes	VAR INC	increased	NA	yes
Gene:S149631_906	BT16	31.686	0.0458	NA	No	No	No	No	No	No	No	0.988505976	0.82025797	Yes	NA	NA	NA	NA
Gdist:S46088_3082	BT16	31.686	0.0115	NA	No	No	No	No	No	No	No	NA	NA	No	NA	NA	NA	NA
Gdist:S431672_3697	BT16	34.607	0.0256	NA	No	No	No	No	No	No	No	NA	NA	No	NA	NA	NA	NA
SalHit:S505394_4239	BT16	34.839	0.022	NA	No	No	No	No	No	No	No	NA	NA	No	NA	NA	NA	NA
Gdist:S289737_6832	BT16	35.073	0.0312	NA	No	No	No	No	No	No	No	0.377637712	0.75343413	Yes	NA	NA	NA	NA
Gdist:S14288_3111	BT16	35.114	0.0186	NA	No	No	No	No	No	No	No	NA	NA	No	NA	NA	NA	NA
Gdist:S14288_8090	BT16	36.817	0.0069	NA	No	No	No	No	No	No	No	NA	NA	No	NA	NA	NA	NA
Gdist:S375094_5419	BT16	38.52	0.0489	NA	No	No	No	No	No	No	No	0.6196875	0.8903222	Yes	NA	NA	NA	NA
SalHit:S648440_1926	BT16	38.52	0.0159	NA	No	No	No	No	No	No	No	NA	NA	No	NA	NA	NA	NA
Gdist:S146363_4892	BT16	38.524	-0.0008	NA	No	No	No	No	No	No	No	NA	NA	No	NA	NA	NA	NA
Gdist:S576229_9129	BT16	38.524	0.0819	none	Yes	No	No	No	No	No	No	0.016276596	0.91619469	Yes	VAR RED	Reduced	NA	NA
Gdist:S195734_1812	BT16	39.38	-0.0051	NA	No	No	No	No	No	No	No	NA	NA	No	NA	NA	NA	NA
cDNA:S597808_1618	BT16	39.401	0.0085	NA	No	No	No	No	No	No	No	NA	NA	No	NA	NA	NA	NA
Gdist:S406012_5746	BT16	41.002	0.0095	NA	No	No	No	No	No	No	No	NA	NA	No	NA	NA	NA	NA
Gdist:S9521_14044	BT16	41.034	0.0343	NA	No	No	No	No	No	No	No	0.565714286	0.24710177	Yes	NA	NA	NA	NA
Gdist:S57654_6357	BT16	41.034	0.0204	NA	No	No	No	No	No	No	No	NA	NA	No	NA	NA	NA	NA
SalHit:S23849_2475	BT16	41.034	0.0135	NA	No	No	No	No	No	No	No	NA	NA	No	NA	NA	NA	NA
Gdist:S171555_4291	BT16	42.562	0.0026	NA	No	No	No	No	No	No	No	NA	NA	No	NA	NA	NA	NA
SalHit:S469071_971	BT16	42.607	0.013	NA	No	No	No	No	No	No	No	NA	NA	No	NA	NA	NA	NA
SalHit:S469071_2535	BT16	42.607	-0.0042	NA	No	No	No	No	No	Yes (0.048)	No	NA	NA	No	NA	NA	NA	NA
Gdist:S134747_2885	BT16	43.211	0.0036	NA	No	No	No	No	No	No	No	NA	NA	No	NA	NA	NA	NA
Gdist:S482727_4666	BT16	43.828	0.0262	NA	No	No	No	No	No	No	No	NA	NA	No	NA	NA	NA	NA
cDNA:S14549_908	BT16	44.389	0.0092	NA	No	No	No	No	No	No	No	NA	NA	No	NA	NA	NA	NA
SalarSNP:GCR_cBin8585_Ctg1	BT16	45.244	-0.0032	NA	No	No	No	No	No	No	No	NA	NA	No	NA	NA	NA	NA
Gdist:S9514_7775	BT16	46.128	0.0105	NA	No	No	No	No	No	No	No	NA	NA	No	NA	NA	NA	NA
Gdist:S323491_6225	BT16	46.128	-0.0072	NA	No	No	No	No	No	No	No	NA	NA	No	NA	NA	NA	NA
Gene:S322365_1730	BT16	46.761	-0.0087	NA	No	No	No	No	No	No	No	NA	NA	No	NA	NA	NA	NA
cDNA:S43503_3699	BT16	47.721	0.0165	NA	No	No	No	No	No	No	No	NA	NA	No	NA	NA	NA	NA
cDNA:S420548_5277	BT16	47.721	0.0162	NA	No	No	No	No	No	No	No	NA	NA	No	NA	NA	NA	NA
cDNA:S614082_811	BT16	47.721	0.0123	NA	No	No	No	No	No	No	No	NA	NA	No	NA	NA	NA	NA
SalHit:S687744_7975	BT16	47.721	0.0068	NA	No	No	No	No	No	No	No	NA	NA	No	NA	NA	NA	NA
Gdist:S137439_430	BT16	47.721	0.0018	NA	No	No	No	No	No	No	No	NA	NA	No	NA	NA	NA	NA
Gdist:S684460_5899	BT16	47.721	-0.0012	NA	No	No	No	No	No	No	No	NA	NA	No	NA	NA	NA	NA
Gdist:S165667_8094	BT16	47.721	-0.0023	NA	No	No	No	No	No	No	No	NA	NA	No	NA	NA	NA	NA
SalarSNP:ESTNV_33882_1084	BT16	47.721	-0.0041	NA	No	No	No	No	No	No	No	NA	NA	No	NA	NA	NA	NA
SalHit:S678411_577	BT16	47.721	-0.01	NA	No	No	No	No	No	No	No	NA	NA	No	NA	NA	NA	NA
SalHit:S83919_2393	BT16	48.525	0.0126	NA	No	No	No	No	No	No	No	NA	NA	No	NA	NA	NA	NA
SalHit:S214020_2561	BT16	48.526	0.0351	NA	Yes	No	No	No	No	No	No	0.776679389	0.24828947	Yes	NA	NA	NA	NA
Gdist:S492181_3329	BT16	48.526	0.0309	NA	No	No	No	No	No	No	No	0.279285714	0.84677586	Yes	NA	NA	NA	NA
Gdist:S492181_7981	BT16	48.526	0.0211	NA	Yes	No	No	No	No	No	No	NA	NA	No	NA	NA	NA	NA
cDNA:S724824_2713	BT16	48.526	0.0164	NA	No	No	No	No	No	No	No	NA	NA	No	NA	NA	NA	NA
cDNA:S286429_654	BT16	48.526	0.005	NA	No	No	No	No	No	No	No	NA	NA	No	NA	NA	NA	NA
SalHit:S641000_5592	BT16	48.526	0.0019	NA	No	No	No	No	No	No	No	NA	NA	No	NA	NA	NA	NA
Gdist:S404817_3473	BT16	48.526	0.0016	NA	No	No	No	No	No	No	No	NA	NA	No	NA	NA	NA	NA
Gdist:S55808_438	BT16	48.527	0.0194	NA	No	No	No	No	No	Yes (0)	Yes (0.008)	NA	NA	No	NA	NA	NA	NA
Gdist:S75720_1907	BT16	48.527	0.0162	NA	No	No	No	No	No	No	No	NA	NA	No	NA	NA	NA	NA
SalHit:S768213_1908	BT16	48.527	0.0066	NA	No	No	No	No	No	No	No	NA	NA	No	NA	NA	NA	NA
cDNA:S161866_1891	BT16	48.527	-0.0046	NA	No	No	No	No	No	No	No	NA	NA	No	NA	NA	NA	NA

SalHit:S267372_2757	BT17	0	0.0063	NA	No	No	No	No	No	No	No	NA	NA	No	NA	NA	NA	NA
cDNA:C124072625_365	BT17	0.383	0.0153	NA	No	No	No	No	No	No	No	NA	NA	No	NA	NA	NA	NA
Gdist:S84543_6593	BT17	2.052	0.0344	NA	No	No	No	No	No	Yes (0.035)	No	0.419853516	0.87616995	Yes	NA	NA	NA	NA
SalHit:S55224_232	BT17	2.309	0.0145	NA	No	No	No	No	No	No	No	NA	NA	No	NA	NA	NA	NA
Gdist:S111475_3504	BT17	11.589	0.032	NA	No	No	No	No	No	No	No	0.916490895	0.87608911	Yes	NA	NA	NA	NA
Gdist:S31502_6472	BT17	16.211	0.0328	none	No	No	No	No	No	No	No	0.928928571	0	Yes	SKJ RED	NA	reduced	NA
SalarSNP:ESTNV_25560_563	BT17	16.269	0.0096	NA	No	No	No	No	No	No	No	NA	NA	No	NA	NA	NA	NA
SalHit:S576605_3402	BT17	16.328	0.0228	NA	No	No	No	No	No	No	No	NA	NA	No	NA	NA	NA	NA
cDNA:S331069_333	BT17	16.386	0.0031	NA	No	No	No	No	No	No	No	NA	NA	No	NA	NA	NA	NA
Gdist:S112240_3631	BT17	16.428	0.0218	NA	No	No	No	No	No	No	No	0.664111239	0.83986655	Yes	NA	NA	NA	NA
SalarSNP:ESTNV_37153_794	BT17	16.428	0.0152	NA	No	No	No	No	No	No	No	NA	NA	No	NA	NA	NA	NA
Gdist:S13432_7148	BT17	16.432	0.0307	NA	No	No	No	No	No	No	No	0.944574855	0.24213303	Yes	NA	NA	NA	NA
Gdist:S116218_6824	BT17	20.021	-0.003	NA	No	No	No	No	No	No	No	NA	NA	No	NA	NA	NA	NA
Gdist:S74204_6794	BT17	20.331	0.0113	NA	No	No	No	No	No	Yes (0.043)	No	NA	NA	No	NA	NA	NA	NA
Gdist:S275209_6204	BT17	23.011	0.0916	NA	No	No	No	No	No	Yes (0.004)	Yes (0.035)	0.468256228	0.53217391	Yes	NA	NA	NA	NA
cDNA:S89683_2659	BT17	23.361	0.026	NA	No	No	No	No	No	No	No	NA	NA	No	NA	NA	NA	NA
cDNA:S89683_2533	BT17	23.711	0.021	NA	No	No	No	No	No	No	No	NA	NA	No	NA	NA	NA	NA
Gdist:S48219_2891	BT17	29.996	0.0058	NA	Yes	No	No	No	No	No	No	NA	NA	No	NA	NA	NA	NA
cDNA:S110517_4456	BT17	29.999	0.0071	NA	No	No	No	No	No	No	No	NA	NA	No	NA	NA	NA	NA
LD:S214769_4157	BT17	30	0.0264	NA	No	No	No	No	No	No	No	NA	NA	No	NA	NA	NA	NA
Gdist:S128480_1702	BT17	30	0.004	NA	No	No	No	No	No	No	No	NA	NA	No	NA	NA	NA	NA
cDNA:S414350_1592	BT17	30.499	0.0417	NA	No	No	No	No	No	No	No	0.705336538	0.55438291	Yes	NA	NA	NA	NA
LD:S214769_14934	BT17	30.514	0.0139	NA	No	No	No	No	No	No	No	NA	NA	No	NA	NA	NA	NA
Gdist:S214769_17521	BT17	30.514	-0.0057	NA	No	No	No	No	No	No	No	NA	NA	No	NA	NA	NA	NA
Gdist:S39740_1700	BT17	31.248	0.0055	NA	No	No	No	No	No	No	No	NA	NA	No	NA	NA	NA	NA
Gdist:S45583_3699	BT17	31.25	0.0097	NA	No	No	No	No	No	No	No	0.519280063	0.1434375	Yes	NA	NA	NA	NA
LD:S45583_5902	BT17	31.251	-0.0073	NA	No	No	No	No	No	No	No	NA	NA	No	NA	NA	NA	NA
LD:S45583_14264	BT17	31.252	0.0141	NA	No	No	No	No	No	No	No	NA	NA	No	NA	NA	NA	NA
cDNA:S542005_1955	BT17	32.902	0.0217	NA	No	No	No	No	No	No	No	NA	NA	No	NA	NA	NA	NA
cDNA:S363566_1471	BT17	34.197	0.0358	NA	No	No	No	No	No	No	No	0.850603908	0.3825	Yes	NA	NA	NA	NA
Gdist:S142071_5198	BT17	34.209	-0.0025	NA	No	No	No	No	No	No	No	NA	NA	No	NA	NA	NA	NA
Gdist:S142071_2129	BT17	34.593	0.0279	NA	No	No	No	No	No	No	No	NA	NA	No	NA	NA	NA	NA
Gdist:S225680_3820	BT17	34.594	0.0116	NA	No	No	No	No	No	No	No	NA	NA	No	NA	NA	NA	NA
Gdist:S279403_9793	BT17	35.395	0.0216	NA	No	No	No	No	No	No	No	NA	NA	No	NA	NA	NA	NA
Gdist:S279403_9877	BT17	35.4	-0.0048	NA	Yes	No	No	No	No	No	No	NA	NA	No	NA	NA	NA	NA
SalHit:S221112_266	BT17	35.548	0.0104	NA	No	No	No	No	No	Yes (0.006)	Yes (0.043)	0.660520833	0.59144776	Yes	NA	NA	NA	NA
cDNA:S262570_3483	BT17	35.706	0.0048	NA	No	No	No	No	No	No	No	NA	NA	No	NA	NA	NA	NA
cDNA:S204896_3399	BT17	42.362	0.003	NA	No	No	No	No	No	No	No	NA	NA	No	NA	NA	NA	NA
Gdist:S125452_7634	BT17	42.367	0.0053	NA	No	No	No	No	No	Yes (0.044)	No	NA	NA	No	NA	NA	NA	NA
SalHit:S575105_1693	BT17	42.554	-0.001	NA	No	No	No	No	No	Yes (0.021)	No	NA	NA	No	NA	NA	NA	NA
SalHit:S578279_333	BT17	47.576	0.0607	NA	No	No	No	No	No	No	No	0.1275	0.80649408	Yes	NA	NA	NA	NA
SalarSNP:ESTNV_36887_452	BT17	47.576	0.0161	NA	No	No	No	No	No	No	No	NA	NA	No	NA	NA	NA	NA
SalarSNP:ESTNV_30479_416	BT17	47.576	0.0117	NA	No	No	No	No	No	No	No	NA	NA	No	NA	NA	NA	NA
Gdist:S302039_5076	BT17	47.576	-0.0027	NA	No	No	No	No	No	Yes (0.023)	No	NA	NA	No	NA	NA	NA	NA
SalHit:S402232_1651	BT17	48.31	0.0487	NA	No	No	No	No	No	No	No	0.016276596	0.0306	Yes	SKJ+VAR INC	increased	increased	NA
Gene:S13023_338	BT17	48.579	0.0218	NA	No	No	No	No	No	No	No	NA	NA	No	NA	NA	NA	NA
Gdist:S151671_9639	BT17	49.234	0.0663	NA	No	No	No	No	No	No	No	0.928928571	0.96861417	Yes	NA	NA	NA	NA
cDNA:S198906_868	BT17	49.234	0.0336	NA	No	No	No	No	No	No	No	0.893131188	0.83274908	Yes	NA	NA	NA	NA
SalHit:S262497_3516	BT17	49.234	0.0088	NA	No	No	No	No	No	No	No	NA	NA	No	NA	NA	NA	NA
cDNA:S133384_1630	BT17	49.234	0.0071	NA	No	No	No	No	No	No	No	NA	NA	No	NA	NA	NA	NA
cDNA:S189184_774	BT17	49.564	-0.0022	NA	No	No	No	No	No	No	No	NA	NA	No	NA	NA	NA	NA
cDNA:S345860_1024	BT17	50.116	0.0018	NA	No	No	No	No	No	No	No	NA	NA	No	NA	NA	NA	NA
SalHit:S552727_1630	BT17	50.119	0.0095	NA	No	No	No	No	No	No	No	NA	NA	No	NA	NA	NA	NA
SalHit:S417941_755	BT17	51.529	0.0404	NA	No	No	No	No	No	No	No	0.574795082	0.6958849	Yes	NA	NA	NA	NA
cDNA:S10364_858	BT17	54.43	0.0496	NA	No	No	No	No	No	No	No	0.743034653	0.48842308	Yes	NA	NA	NA	NA
SalHit:S597256_2140	BT17	54.458	0.0725	Salmo tr	Yes	No	No	No	No	Yes (0.037)	No	0.0095625	0.51910714	Yes	VAR INC	increased	NA	yes
SalHit:C123376875_123	BT17	56.212	0.0015	NA	Yes	No	No	No	No	No	No	NA	NA	No	NA	NA	NA	NA
Gdist:S87311_4815	BT18	0	0.0253	NA	No	No	No	No	No	No	No	NA	NA	No	NA	NA	NA	NA
SalHit:S43071_3808	BT18	0.021	0.0222	NA	No	No	No	No	No	No	No	NA	NA	No	NA	NA	NA	NA
Gdist:S94599_4328	BT18	0.026	-0.0048	NA	No	No	No	No	No	No	No	NA	NA	No	NA	NA	NA	NA
Gdist:S321579_2600	BT18	0.038	-0.0002	NA	No	No	No	No	No	No	No	NA	NA	No	NA	NA	NA	NA
Gene:S640863_2054	BT18	0.04	0.0346	NA	No	No	No	No	No	No	No	0.279285714	0.39962687	Yes	NA	NA	NA	NA
Gene:S640863_2290	BT18	0.044	0.0042	NA	No	No	No	No	No	No	No	NA	NA	No	NA	NA	NA	NA
SalHit:S233419_8892	BT18	5.808	0.022	NA	No	No	No	No	No	No	No	NA	NA	No	NA	NA	NA	NA
Gdist:S121987_4199	BT18	5.846	0.0181	NA	Yes	No	No	No	No	No	No	NA	NA	No	NA	NA	NA	NA
Gdist:S75685_3966	BT18	5.846	0.0171	NA	No	No	No	No	No	No	No	NA	NA	No	NA	NA	NA	NA
SalHit:S413688_3464	BT18	5.846	0.0022	NA	No	No	No	No	No	No	No	NA	NA	No	NA	NA	NA	NA
cDNA:S8550_272	BT18	5.846	-0.0131	NA	No	No	No	No	No	No	No	NA	NA	No	NA	NA	NA	NA
Gdist:S121987_6689	BT18	5.853	-0.0097	NA	Yes	No	No	No	No	Yes (0.012)	No	NA	NA	No	NA	NA	NA	NA
Gdist:S219078_5165	BT18	6.243	-0.0123	NA	No	No	No	No	No	No	No	NA	NA	No	NA	NA	NA	NA
Gdist:S11640_7049	BT18	9.163	-0.0036	NA	No	No	No	No	No	No	No	NA	NA	No	NA	NA	NA	NA
Gene:S175365_5658	BT18	11.754	0.0566	none	No	No	No	No	No	No	No	0.963553748	0	Yes	SKJ RED	NA	reduced	NA
Gene:S839123_87	BT18	13.049	0.0108	NA	No	No	No	No	No	No	No	NA	NA	No	NA	NA	NA	NA
Gdist:S174870_5795	BT18	15.926	0.0004	NA	No	No	No	No	No	No	No	NA	NA	No	NA	NA	NA	NA
Gdist:S119534_10343	BT18	17.597	0.0202	NA	No	No	No	No	No	No	No	NA	NA	No	NA	NA	NA	NA
Gdist:S119534_5653	BT18	18.435	0.0295	NA	No	No	No	No	No	No	No	NA	NA	No	NA	NA	NA	NA
Gdist:S509470_6040	BT18	22.5	0.0037	NA	No	No	No	No	No	No	No	NA	NA	No	NA	NA	NA	NA
Gdist:S509470_3792	BT18	22.5	-0.0029	NA	No	No	No	No	No	No	No	NA	NA	No	NA	NA	NA	NA
Gdist:S110976_581	BT18	23.181	0.0033	NA	No	No	No	No	No	No	No	NA	NA	No	NA	NA	NA	NA
cDNA:S453499_827	BT18	24.762	0.017	NA	No	No	No	No	No	No	No	NA	NA	No	NA	NA	NA	NA
Gdist:S113989_5664	BT18	26.141	0.0022	NA	No	No	No	No	No	No	No	NA	NA	No	NA	NA	NA	NA
cDNA:S86370_1080	BT18	27.916	0.0049	NA	No	No	No	No	No	No	No	NA	NA	No	NA	NA	NA	NA
cDNA:S727972_1217	BT18	29.627	0.0217	NA	No	No	No	No	No	No	No	NA	NA	No	NA	NA	NA	NA
cDNA:S154283_1102	BT18	30.223	0.0099	NA	No	No	No	No	No	Yes (0.023)	No	NA	NA	No	NA	NA	NA	NA
cDNA:S114312_248	BT18	30.87	-0.0056	NA	No	No	No	No	No	No	No	NA	NA	No	NA	NA	NA	NA
cDNA:S263179_1819	BT18	30.982	0.0204	NA	No	No	No	No	No	No	No	NA	NA	No	NA	NA	NA	NA
cDNA:S346048_5673	BT18	31.017	0.0163	NA	No	No	No	No	No	No	No	NA	NA	No	NA	NA	NA	NA
Gdist:S88105_3337	BT18	31.095	0.0228	NA	No	No	No	No	No	No	No	NA	NA	No	NA	NA	NA	NA
cDNA:S64260_3920	BT18	31.099	0.0328	NA	No	No	No	No	No	No	No	0.871286449	0.94321023	Yes	NA	NA	NA	NA
cDNA:S376368_5043	BT18	31.104	0.0478	NA	No	No	No	No	No	No	No	0.673928571	0.174375	Yes	NA	NA	NA	NA
Gdist:S355068_573	BT18	32.38	0.0333	NA	No	No	No	No	No	No	No	0.365723684	0.89481818	Yes	NA	NA	NA	NA
cDNA:C124528172_494	BT18	32.38	0.0212	NA	No	No	No	No	No	No	No	NA	NA	No	NA	NA	NA	NA
Gdist:S62713_5259	BT18	32.38	0.0019	NA	No	No	No	No	No	No	No	NA	NA	No	NA	NA	NA	NA
Gdist:S35706_2318	BT18	33.078	0.0002	NA	No	No	No	No	No	No	No	NA	NA	No	NA	NA	NA	NA
Gdist:S35706_2935	BT18	33.078	-0.0077	NA	No	No	No	No	No	No	No	NA	NA	No	NA	NA	NA	NA
cDNA:S124403_4920	BT18	33.078	-0.009	NA	No	No	No	No	No	No	No	NA	NA	No	NA	NA	NA	NA
Gdist:S35706_5421	BT18	33.093	0.0065	NA	No	No	No	No	No	No	No	NA	NA	No	NA	NA	NA	NA
Gdist:S655076_2933	BT18	36.912	0.0045	NA	No	No	No	No	No	No	No	NA	NA	No	NA	NA	NA	NA
Gdist:S120639_8272	BT18	36.997	0.0468	NA	No	No	No	No	No	No	No	0.514568404	0.32564189	Yes	NA	NA	NA	NA
Gdist:S150464_4617	BT18	37.828	-0.0014	NA	No	No	No	No	No	No	No	NA	NA	No	NA	NA	NA	NA
Gdist:S150464_1018	BT18	38.566	0	NA	No	No	No	No	No	No	No	NA	NA	No	NA	NA	NA	NA
cDNA:S87097_4670	BT18	39.29	0.0156	NA	No	No	No	No	No	No	No	NA	NA	No	NA	NA	NA	NA
SalHit:S161886_4354	BT18	39.29	-0.0002	NA	No	No	No	No	No	No	No	NA	NA	No	NA	NA	NA	NA
cDNA:S87097_5007	BT18	39.29	-0.0011	NA	No	No	No	No	No	Yes (0.035)	No	NA	NA	No	NA	NA	NA	NA
Gdist:S9249_2949	BT18	39.541	0.0412	NA	No	Yes	No	No	No	No	No	0	0.69352273	Yes	VAR INC	increased	NA	yes
Gdist:S9249_14269	BT18	39.791	0.0176	NA	No	No	No	No	No	Yes (0.002)	Yes (0.02)	NA	NA	No	NA	NA	NA	NA
Gdist:S9249_8004	BT18	41.559	0.0488	NA	No	No	No	No	No	No	No	0.468256228	0.88732839	Yes	NA	NA	NA	NA
Gdist:S245191_7381	BT18	42.063	0.0174	NA	Yes	No	No	No	No	No	No	NA	NA	No	NA	NA	NA	NA
SalHit:S38141_6780	BT18	42.072	0.005	NA	No	No	No	No	No	Yes (0.02)	No	NA	NA	No	NA	NA	NA	NA
Gdist:S187993_3785	BT18	42.079	0.0313	NA	No	No	No	No	No	No	No	0.176538462	0.72269585	Yes	NA	NA	NA	NA
SalHit:S31821_9847	BT18	42.116	0.0107	NA	No	No	No	No	No	No	No	NA	NA	No	NA	NA	NA	NA
SalarSNP:ESTNV_36918_790	BT18	44.009	0.0206	NA	No	No	No	No	No	Yes (0.014)	No	NA	NA	No	NA	NA	NA	NA
Gdist:S30909_14491	BT18	44.141	-0.0044	NA	No	No	No	No	No	No	No	NA	NA	No	NA	NA	NA	NA
Gdist:S42647_9352	BT18	45.468	0.0045	NA	No	No	No	No	No	No	No	NA	NA	No	NA	NA	NA	NA
Gdist:S259770_6213	BT18	45.476	-0.0055	NA	No	No	No	No	No	No	No	NA	NA	No	NA	NA	NA	NA
Gdist:S558326_1436	BT18	47.287	0.0274	NA	No	No	No	No	No	No	No	NA	NA	No	NA	NA	NA	NA
SalHit:S406986_4178	BT18	49.042	0.0068	NA	No	No	No	No	No	No	No	NA	NA	No	NA	NA	NA	NA
cDNA:S363461_6741	BT18	49.042	0.0009	NA	Yes	No	No	No	No	No	No	NA	NA	No	NA	NA	NA	NA
cDNA:S165871_3361	BT18	49.043	0.01	NA	Yes	No	No	No	No	No	No	NA	NA	No	NA	NA	NA	NA
cDNA:S171492_2300	BT18	49.326	0.0124	NA	No	No	No	No	No	Yes (0.003)	Yes (0.031)	NA	NA	No	NA	NA	NA	NA
cDNA:S582565_327	BT18	49.917	0.0131	NA	No	No	No	No	No	Yes (0.004)	Yes (0.035)	NA	NA	No	NA	NA	NA	NA
Gdist:S134762_1869	BT18	49.918	0.0033	NA	No	No	No	No	No	Yes (0.046)	No	NA	NA	No	NA	NA	NA	NA
cDNA:S201721_1936	BT18	49.918	0.0011	NA	No	No	No	No	No	No	No	NA	NA	No	NA	NA	NA	NA
cDNA:S52627_2009	BT18	49.918	-0.0042	NA	No	No	No	No	No	Yes (0)	Yes (0.005)	NA	NA	No	NA	NA	NA	NA
Gene:S678020_4224	BT18	49.919	0.0195	NA	No	No	No	No	No	No	No	NA	NA	No	NA	NA	NA	NA
Gdist:S306468_5178	BT18	49.919	0.0079	NA	No	No	No	No	No	No	No	NA	NA	No	NA	NA	NA	NA
cDNA:S267203_6159	BT18	49.926	0.0009	NA	No	No	No	No	No	No	No	NA	NA	No	NA	NA	NA	NA

cDNA:S47753_3153	BT18	49.926	-0.0016	NA	No	No	No	No	No	No	No	NA	NA	No	NA	NA	NA	NA
Gdist:S82024_6472	BT18	50.744	0.0077	NA	No	No	No	No	No	No	No	NA	NA	No	NA	NA	NA	NA
cDNA:S68579_5662	BT18	51.435	0.0152	NA	Yes	No	No	No	No	No	No	NA	NA	No	NA	NA	NA	NA
Gdist:S536265_4046	BT18	51.818	0.0405	NA	Yes	No	No	No	No	No	No	0.845991993	0.595125	Yes	NA	NA	NA	NA
Gdist:S342274_7869	BT18	51.832	0.0038	NA	No	No	No	No	No	No	No	NA	NA	No	NA	NA	NA	NA
Gdist:S60727_14228	BT18	51.846	0.0103	NA	No	No	No	No	No	No	No	NA	NA	No	NA	NA	NA	NA
Gdist:S80623_3543	BT18	51.846	0.0037	NA	Yes	No	No	No	No	No	No	NA	NA	No	NA	NA	NA	NA
LD:S162605_5921	BT18	52.224	0.0534	NA	No	No	No	No	No	No	No	0.956797994	0.24466216	Yes	NA	NA	NA	NA
cDNA:S961_6169	BT18	52.224	0.0341	NA	No	No	No	No	No	Yes (0.015)	No	0.050625	0.76824153	Yes	NA	NA	NA	NA
Gdist:S218866_10545	BT18	52.224	0.0286	NA	Yes	No	No	No	No	No	No	NA	NA	No	NA	NA	NA	NA
SalHit:S288563_5644	BT18	52.224	0.0184	NA	No	No	No	No	No	No	No	NA	NA	No	NA	NA	NA	NA
SalHit:S288563_7916	BT18	52.224	0.0184	NA	No	No	No	No	No	No	No	NA	NA	No	NA	NA	NA	NA
Gdist:S18533_2699	BT18	52.224	0.0087	NA	No	No	No	No	No	No	No	NA	NA	No	NA	NA	NA	NA
SalHit:S288563_5173	BT18	52.224	0.0072	NA	No	No	No	No	No	No	No	NA	NA	No	NA	NA	NA	NA
Gdist:S262697_3192	BT18	52.224	0.0063	NA	No	No	No	No	No	Yes (0.021)	No	NA	NA	No	NA	NA	NA	NA
cDNA:S277744_4747	BT18	52.224	0.0046	NA	No	No	No	No	No	No	No	NA	NA	No	NA	NA	NA	NA
cDNA:S1296_2391	BT18	52.224	0.0035	NA	No	No	No	No	No	No	No	NA	NA	No	NA	NA	NA	NA
LD:S162605_15211	BT18	52.224	0.0012	NA	No	No	No	No	No	No	No	NA	NA	No	NA	NA	NA	NA
Gdist:S30094_10154	BT18	52.224	0.0003	NA	No	No	No	No	No	Yes (0)	Yes (0.004)	NA	NA	No	NA	NA	NA	NA
cDNA:S623498_4238	BT18	52.224	-0.0035	NA	No	No	No	No	No	No	No	NA	NA	No	NA	NA	NA	NA
Gdist:S113953_6012	BT18	52.598	0.0432	NA	No	No	No	No	No	No	No	0.862473638	0.18608108	Yes	NA	NA	NA	NA
cDNA:S513890_2408	BT18	53.018	0.0033	NA	No	No	No	No	No	Yes (0)	Yes (0.006)	0.0095625	0.46907407	Yes	VAR INC	increased	NA	NA
cDNA:S347134_7224	BT18	53.126	0.006	NA	No	No	No	No	No	No	No	NA	NA	No	NA	NA	NA	NA
Gdist:S674434_415	BT18	53.903	0.0165	NA	No	No	No	No	No	Yes (0.023)	No	NA	NA	No	NA	NA	NA	NA
Gdist:S72178_6170	BT18	53.908	0.0172	NA	No	No	No	No	No	Yes (0)	Yes (0.005)	NA	NA	No	NA	NA	NA	NA
Gdist:S211150_4377	BT18	53.912	-0.008	NA	No	No	No	No	No	No	No	NA	NA	No	NA	NA	NA	NA
Gdist:S179492_4653	BT18	54.26	0.0081	NA	No	No	No	No	No	Yes (0.002)	Yes (0.024)	NA	NA	No	NA	NA	NA	NA
Gdist:S173197_2357	BT18	54.267	0.0319	NA	No	No	No	No	No	No	No	0.514568404	0.37646053	Yes	NA	NA	NA	NA
LD:S57836_13135	BT18	54.274	0.0332	NA	No	No	No	No	No	No	No	0.546428571	0.76335837	Yes	NA	NA	NA	NA
Gdist:S580032_7318	BT18	54.349	0.0181	NA	No	No	No	No	No	No	No	NA	NA	No	NA	NA	NA	NA
Gdist:S82706_8842	BT18	55.939	0.0298	NA	Yes	No	No	No	No	No	No	NA	NA	No	NA	NA	NA	NA
Gdist:S187130_2080	BT18	56.54	0.0371	NA	No	No	No	No	No	No	No	0.953502155	0.55866719	Yes	NA	NA	NA	NA
Gdist:S5107_7083	BT18	56.555	0.0616	NA	No	No	No	Yes (0.033)	No	No	No	0.838743243	0.35534024	Yes	NA	NA	NA	NA
Gdist:S35381_6655	BT18	56.555	0.0058	NA	No	No	No	No	No	No	No	NA	NA	No	NA	NA	NA	NA
cDNA:S397066_4260	BT18	56.56	0.0377	NA	No	No	No	No	No	No	No	0.741189759	0.97212245	Yes	NA	NA	NA	NA
Gdist:S9252_10058	BT18	57.711	-0.0003	NA	No	No	No	No	No	Yes (0.002)	Yes (0.024)	NA	NA	No	NA	NA	NA	NA
cDNA:S75019_4677	BT18	57.763	0.0422	NA	No	No	No	No	No	No	No	0.213317308	0.60473464	Yes	NA	NA	NA	NA
Gdist:S202449_8644	BT18	57.763	0.0308	NA	No	No	No	No	No	No	No	0.742903386	0.6958849	Yes	NA	NA	NA	NA
Gdist:S5573_6262	BT18	57.763	0.0221	NA	No	No	No	No	No	No	No	NA	NA	No	NA	NA	NA	NA
cDNA:S74748_3603	BT18	57.763	0.0219	NA	No	No	No	No	No	No	No	NA	NA	No	NA	NA	NA	NA
Gdist:S29822_11735	BT18	57.763	0.0215	NA	No	No	No	No	No	No	No	NA	NA	No	NA	NA	NA	NA
Gdist:S80178_6161	BT18	57.763	0.0193	NA	No	No	No	No	No	No	No	NA	NA	No	NA	NA	NA	NA
Gdist:S426986_2583	BT18	57.763	0.0157	NA	No	No	No	No	No	No	No	NA	NA	No	NA	NA	NA	NA
Gdist:S270675_913	BT18	57.763	0.013	NA	No	No	No	No	No	No	No	NA	NA	No	NA	NA	NA	NA
cDNA:S474699_744	BT18	57.763	0.013	NA	No	No	No	No	No	No	No	NA	NA	No	NA	NA	NA	NA
Gdist:S181338_3734	BT18	57.763	0.0063	NA	No	No	No	No	No	No	No	NA	NA	No	NA	NA	NA	NA
cDNA:S603437_983	BT18	57.763	0.0018	NA	No	No	No	No	No	No	No	NA	NA	No	NA	NA	NA	NA
cDNA:S654208_2204	BT18	57.763	0	NA	No	No	No	No	No	No	No	NA	NA	No	NA	NA	NA	NA
Gdist:S29822_10594	BT18	57.763	-0.0022	NA	No	No	No	No	No	No	No	NA	NA	No	NA	NA	NA	NA
cDNA:S285965_1544	BT18	57.763	-0.0075	NA	No	No	No	No	No	No	No	NA	NA	No	NA	NA	NA	NA
Gdist:S218335_2397	BT18	57.763	-0.0113	NA	Yes	No	No	No	No	Yes (0.014)	No	NA	NA	No	NA	NA	NA	NA
Gdist:S75610_10060	BT18	57.763	-0.0116	NA	No	No	No	No	No	No	No	NA	NA	No	NA	NA	NA	NA
SalHit:S25721_5836	BT18	57.764	0.03	NA	No	No	No	No	No	No	No	NA	NA	No	NA	NA	NA	NA
SalHit:S25721_7918	BT18	57.778	0.0221	NA	No	No	No	No	No	No	No	NA	NA	No	NA	NA	NA	NA
SalHit:S50453_1549	BT19	0	0.0218	NA	Yes	No	No	No	No	No	No	NA	NA	No	NA	NA	NA	NA
SalHit:S306722_1825	BT19	1.487	0.0019	NA	No	No	No	No	No	No	No	NA	NA	No	NA	NA	NA	NA
Gdist:S158947_7421	BT19	1.499	0.0606	NA	No	No	No	No	No	No	No	0.678851351	0.72753417	Yes	NA	NA	NA	NA
cDNA:S266225_1746	BT19	1.561	0.0151	NA	Yes	No	No	No	No	Yes (0.014)	No	NA	NA	No	NA	NA	NA	NA
Gdist:S65186_1884	BT19	1.563	0.0477	NA	No	No	No	No	No	No	No	0.820352505	0.60417614	Yes	NA	NA	NA	NA
Gdist:S313587_1220	BT19	1.73	-0.0021	NA	No	No	No	No	No	No	No	NA	NA	No	NA	NA	NA	NA
Gdist:S477174_2979	BT19	4.32	0.0084	NA	No	No	No	No	No	No	No	NA	NA	No	NA	NA	NA	NA
Gdist:S629174_10246	BT19	5.351	-0.002	NA	Yes	No	No	No	No	No	No	NA	NA	No	NA	NA	NA	NA
cDNA:S78935_3658	BT19	5.352	0.002	NA	No	No	No	No	No	No	No	NA	NA	No	NA	NA	NA	NA
SalHit:S612302_7117	BT19	6.279	0.0075	NA	No	No	No	No	No	No	No	NA	NA	No	NA	NA	NA	NA
SalHit:S118711_4851	BT19	6.303	0.0456	NA	No	No	No	No	No	No	No	0.299431818	0.17485714	Yes	NA	NA	NA	NA
SalHit:S612302_2168	BT19	6.303	-0.0067	NA	No	No	No	No	No	Yes (0.023)	No	NA	NA	No	NA	NA	NA	NA
SalHit:S118711_8963	BT19	6.304	0.0374	NA	Yes	No	No	No	No	No	No	0.641989437	0.83779174	Yes	NA	NA	NA	NA
Gdist:S8928_3592	BT19	6.305	0.0195	NA	No	No	No	No	No	No	No	NA	NA	No	NA	NA	NA	NA
Gene:S35120_486	BT19	6.305	0.0108	NA	Yes	No	No	No	No	No	No	NA	NA	No	NA	NA	NA	NA
cDNA:S521910_1865	BT19	6.305	0.0025	NA	No	No	No	No	No	No	No	NA	NA	No	NA	NA	NA	NA
Gdist:S8928_5360	BT19	6.307	-0.0021	NA	No	No	No	No	No	No	No	NA	NA	No	NA	NA	NA	NA
Gdist:S408054_4248	BT19	6.346	0.0181	NA	No	No	No	No	No	No	No	NA	NA	No	NA	NA	NA	NA
SalHit:S126014_6497	BT19	7.728	-0.0029	NA	No	No	No	No	No	Yes (0.032)	No	NA	NA	No	NA	NA	NA	NA
SalHit:S799945_710	BT19	10.03	0.0128	NA	No	No	No	No	No	No	No	NA	NA	No	NA	NA	NA	NA
Gdist:S509402_8608	BT19	10.03	-0.003	NA	No	No	No	No	No	No	No	NA	NA	No	NA	NA	NA	NA
Gdist:S274931_2188	BT19	10.03	-0.009	NA	No	No	No	No	No	Yes (0)	Yes (0.004)	NA	NA	No	NA	NA	NA	NA
Gdist:S364012_6936	BT19	10.186	0.0437	NA	Yes	No	No	No	No	No	No	0.988505976	0.1434375	Yes	NA	NA	NA	NA
Gdist:S373275_5074	BT19	13.179	0.0103	NA	No	No	No	No	No	No	No	NA	NA	No	NA	NA	NA	NA
Gdist:S220267_13534	BT19	13.355	-0.0012	NA	No	No	No	No	No	Yes (0.002)	Yes (0.024)	NA	NA	No	NA	NA	NA	NA
Gdist:S220267_14577	BT19	13.36	0.0012	NA	No	No	No	No	No	Yes (0.014)	No	NA	NA	No	NA	NA	NA	NA
SalHit:S81217_8404	BT19	13.365	0.0293	NA	No	No	No	No	No	No	No	NA	NA	No	NA	NA	NA	NA
cDNA:S524638_117	BT19	13.365	0.0261	NA	No	No	No	No	No	Yes (0.012)	No	NA	NA	No	NA	NA	NA	NA
SalHit:S81217_10509	BT19	13.365	0.0113	NA	No	No	No	No	No	No	No	NA	NA	No	NA	NA	NA	NA
Gdist:S85572_6735	BT19	13.365	0.0068	NA	No	No	No	No	No	No	No	NA	NA	No	NA	NA	NA	NA
cDNA:S475438_2430	BT19	13.365	-0.0016	NA	No	No	No	No	No	No	No	NA	NA	No	NA	NA	NA	NA
cDNA:S683469_299	BT19	15.318	0.0079	NA	No	No	No	No	No	Yes (0)	Yes (0.009)	NA	NA	No	NA	NA	NA	NA
cDNA:S265325_1830	BT19	23.814	-0.002	NA	No	No	No	No	No	No	No	NA	NA	No	NA	NA	NA	NA
LD:S436279_10720	BT19	24.39	0.0136	NA	No	No	No	No	No	No	No	NA	NA	No	NA	NA	NA	NA
Gdist:S44370_2167	BT19	27.108	0.0249	NA	No	No	No	No	No	No	No	NA	NA	No	NA	NA	NA	NA
cDNA:S295997_808	BT19	27.109	0.0062	NA	No	No	No	No	No	No	No	NA	NA	No	NA	NA	NA	NA
Gdist:S116660_5693	BT19	27.111	-0.0011	NA	No	No	No	No	No	No	No	NA	NA	No	NA	NA	NA	NA
Gdist:S116660_10167	BT19	27.111	-0.0039	NA	No	No	No	No	No	Yes (0.007)	No	NA	NA	No	NA	NA	NA	NA
Gdist:S321426_6372	BT19	27.965	-0.0022	NA	No	No	No	No	No	Yes (0.032)	No	NA	NA	No	NA	NA	NA	NA
Gdist:S155219_1764	BT19	30.128	0.0131	NA	No	No	No	No	No	No	No	NA	NA	No	NA	NA	NA	NA
SalarSNP:ESTV_13981_575	BT19	30.133	-0.001	NA	No	No	No	No	No	No	No	NA	NA	No	NA	NA	NA	NA
Gdist:S18797_6393	BT19	30.442	0.0021	NA	No	No	No	No	No	No	No	NA	NA	No	NA	NA	NA	NA
SalHit:S619952_1799	BT19	32.282	0.0619	NA	No	No	No	No	No	No	No	0.351682464	0.3565678	Yes	NA	NA	NA	NA
cDNA:S295692_1789	BT19	32.316	0.0052	NA	No	No	No	No	No	No	No	NA	NA	No	NA	NA	NA	NA
Gdist:S307061_419	BT19	37.121	0.0204	NA	Yes	No	No	No	No	No	No	NA	NA	No	NA	NA	NA	NA
SalHit:S581751_1502	BT19	37.901	0.0031	NA	No	No	No	No	No	No	No	NA	NA	No	NA	NA	NA	NA
Gdist:S40741_4112	BT19	39.427	0.019	NA	Yes	No	No	No	No	No	No	NA	NA	No	NA	NA	NA	NA
Gdist:S785195_7288	BT19	39.683	0.0154	NA	No	No	No	No	No	No	No	NA	NA	No	NA	NA	NA	NA
Gdist:S479839_14426	BT19	40.166	0.0237	NA	No	No	No	No	No	No	No	NA	NA	No	NA	NA	NA	NA
SalHit:S550185_614	BT19	40.424	-0.0015	NA	No	No	No	No	No	No	No	NA	NA	No	NA	NA	NA	NA
Gdist:S249254_136	BT19	41.417	0.013	NA	No	No	No	No	No	No	No	NA	NA	No	NA	NA	NA	NA
Gdist:S239630_2828	BT19	41.417	0.0121	NA	No	No	No	No	No	No	No	NA	NA	No	NA	NA	NA	NA
Gdist:S695422_4610	BT19	41.42	0.0409	NA	No	No	No	No	No	No	No	0.453596654	0.95357891	Yes	NA	NA	NA	NA
cDNA:S73902_5315	BT19	41.42	0.0361	NA	No	No	No	No	No	No	No	0.078238636	0.82025797	Yes	NA	NA	NA	NA
cDNA:S304352_1007	BT19	41.42	0.0412	PREDIC'	No	No	No	No	No	No	No	0.0095625	0.83654676	Yes	VAR RED	Reduced	NA	yes
cDNA:S283503_224	BT19	41.626	0.026	NA	No	No	No	No	No	No	No	0.531510736	0.8616041	Yes	NA	NA	NA	NA
Gdist:S475860_5599	BT19	42.218	0.018	NA	No	No	No	No	No	Yes (0)	Yes (0.008)	NA	NA	No	NA	NA	NA	NA
Gdist:S475860_1835	BT19	42.218	0.0035	NA	No	No	No	No	No	Yes (0.035)	No	NA	NA	No	NA	NA	NA	NA
LD:S168444_13275	BT19	42.686	0.0178	NA	No	No	No	No	No	No	No	NA	NA	No	NA	NA	NA	NA
LD:S168444_21932	BT19	43.176	0.0285	NA	No	No	No	No	No	No	No	NA	NA	No	NA	NA	NA	NA
Gdist:S376809_6494	BT19	44.367	0.0199	NA	Yes	No	No	No	No	No	No	0.798073308	0.13270408	Yes	NA	NA	NA	NA
Gdist:S146498_5322	BT19	44.372	-0.0045	NA	Yes	No	No	No	No	No	No	NA	NA	No	NA	NA	NA	NA
cDNA:S433351_2306	BT19	44.377	0.0461	NA	No	No	No	No	No	Yes (0.035)	No	0.532767857	0.98585128	Yes	NA	NA	NA	NA
LD:S139093_3902	BT19	44.377	0.0313	NA	Yes	No	No	No	No	No	No	0.895226151	0.41482394	Yes	NA	NA	NA	NA
Gdist:S20175_4137	BT19	44.377	0.0231	NA	No	No	No	No	No	Yes (0.043)	No	NA	NA	No	NA	NA	NA	NA
Gdist:S146498_10504	BT19	44.377	0.1958	NA	Yes	no	no	No	No	No	No	NA	NA	no	NA	NA	NA	NA
Gdist:S499800_4765	BT19	44.377	0.1325	SIX6.....l	Yes	Yes	No	No	No	No	No	0.565105932	0.82148131	Yes	Outlier Varde	Outlier Var	NA	yes
SalHit:S525071_6983	BT19	46.167	0.0034	NA	Yes	No	No	No	No	No	No	NA	NA	No	NA	NA	NA	NA
SalHit:S525071_6038	BT19	46.167	-0.0067	NA	No	No	No	No	No	No	No	NA	NA	No	NA	NA	NA	NA
Gdist:S799448_9673	BT19	46.173	0.071	NA	No	No	No	No	No	No	No	0.134391892	0.88813764	Yes	NA	NA	NA	NA
Gdist:S476718_7908	BT19	46.18	-0.013	NA	No	No	No	No	No	No	No	NA	NA	No	NA	NA	NA	NA
Gdist:S396961_2735	BT19	46.382	0.0039	NA	Yes	No	No	No	No	No	No	NA	NA	No	NA	NA	NA	NA
Gdist:S338481_5696	BT19	46.588	0.0116	NA	No	No	No	No	No	Yes (0.007)	Yes (0.049)	NA	NA	No	NA	NA	NA	NA

Gdist:S513265_4803	BT19	46.774	0.0152	NA	Yes	No	No	No	No	No	No	NA	NA	No	NA	NA	NA	NA
Gdist:S104125_7099	BT19	47.317	0.0522	NA	No	No	No	No	No	No	No	0.735576923	0.95357891	Yes	NA	NA	NA	NA
Gdist:S88291_1138	BT19	47.872	0.0022	NA	No	No	No	No	No	No	No	NA	NA	No	NA	NA	NA	NA
Gdist:S208424_7748	BT19	47.872	-0.002	NA	No	No	No	No	No	No	No	NA	NA	No	NA	NA	NA	NA
cDNA:S302429_3381	BT19	48.625	0.0198	NA	No	No	No	No	No	No	No	NA	NA	No	NA	NA	NA	NA
cDNA:S84920_5746	BT19	48.626	0.085	NA	Yes	No	No	No	No	No	No	0.329480198	0.83808482	Yes	NA	NA	NA	NA
cDNA:S20680_1709	BT19	48.626	0.0236	NA	No	No	No	No	No	No	No	NA	NA	No	NA	NA	NA	NA
cDNA:S166813_3437	BT19	48.725	0.019	NA	No	No	No	No	No	No	No	NA	NA	No	NA	NA	NA	NA
cDNA:S104710_1557	BT19	48.842	0.036	NA	No	No	No	No	No	No	No	0.510426421	0.61532609	Yes	NA	NA	NA	NA
cDNA:S104710_1633	BT19	48.95	-0.0025	NA	No	No	No	No	No	No	No	NA	NA	No	NA	NA	NA	NA
Gdist:S40792_1868	BT19	49.043	0.0471	NA	No	No	No	No	No	No	No	0.093590426	0.84488496	Yes	NA	NA	NA	NA
cDNA:S104710_3393	BT19	49.043	0.0311	NA	No	No	No	No	No	Yes (0.009)	No	0.532767857	0.37616022	Yes	NA	NA	NA	NA
cDNA:S3786_11163	BT19	49.043	0.013	NA	No	No	No	No	No	No	No	NA	NA	No	NA	NA	NA	NA
Gdist:S532621_6393	BT19	49.043	0.0097	NA	No	No	No	No	No	Yes (0.004)	Yes (0.035)	NA	NA	No	NA	NA	NA	NA
Gdist:S40792_1298	BT19	49.043	-0.0007	NA	No	No	No	No	No	No	No	NA	NA	No	NA	NA	NA	NA
Gdist:S40792_2940	BT19	49.043	-0.0077	NA	No	No	No	No	No	No	No	NA	NA	No	NA	NA	NA	NA
cDNA:S748975_1097	BT19	49.854	0.0107	NA	No	No	No	No	No	No	No	NA	NA	No	NA	NA	NA	NA
SalHit:S167224_1311	BT19	50.594	0.0284	NA	No	No	No	No	No	No	No	NA	NA	No	NA	NA	NA	NA
cDNA:C123994563_51	BT19	51.301	0.0054	NA	No	No	No	No	No	No	No	NA	NA	No	NA	NA	NA	NA
Gdist:S260824_9407	BT19	52.925	0.0309	NA	No	No	No	No	No	No	No	0.380912863	0.2664959	Yes	NA	NA	NA	NA
Gdist:S260824_7668	BT19	52.94	0.0314	NA	No	No	No	No	No	No	No	0.12983945	0.17126866	Yes	NA	NA	NA	NA
cDNA:S342230_358	BT19	52.955	0.0064	NA	No	No	No	No	No	No	No	NA	NA	No	NA	NA	NA	NA
cDNA:S363339_747	BT19	52.956	0.0214	NA	No	No	No	No	No	No	No	0.574279778	0.46026971	Yes	NA	NA	NA	NA
cDNA:S342230_989	BT19	52.956	-0.0031	NA	No	No	No	No	No	No	No	NA	NA	No	NA	NA	NA	NA
Gdist:S139450_15410	BT19	52.956	-0.0117	NA	No	No	No	No	No	No	No	NA	NA	No	NA	NA	NA	NA
Gdist:S157867_5401	BT19	52.956	0.0443	none	No	No	No	No	No	No	No	0.928928571	0.01821429	Yes	SKJ RED	NA	reduced	yes
cDNA:S571359_1687	BT20	0	0.0072	NA	No	No	No	No	No	Yes (0.002)	Yes (0.024)	NA	NA	No	NA	NA	NA	NA
Gdist:S123238_331	BT20	0.283	0.07	NA	No	No	No	No	No	No	No	0.115471698	0.59723684	Yes	NA	NA	NA	NA
Gdist:S16868_3818	BT20	0.313	0.016	NA	No	No	No	No	No	No	No	NA	NA	No	NA	NA	NA	NA
Gdist:S16868_7515	BT20	0.505	-0.0135	NA	No	No	No	No	No	No	No	NA	NA	No	NA	NA	NA	NA
cDNA:S502052_2637	BT20	3.228	0.0153	NA	No	No	No	No	No	No	No	NA	NA	No	NA	NA	NA	NA
Gdist:S142662_6335	BT20	4.243	0.0123	NA	No	No	No	No	No	No	No	NA	NA	No	NA	NA	NA	NA
Gdist:S438844_6753	BT20	10.053	-0.0059	NA	No	No	No	No	No	No	No	NA	NA	No	NA	NA	NA	NA
Gdist:S169407_3124	BT20	10.128	0.0735	none	No	No	No	No	No	No	No	0.044625	0.92678416	Yes	VAR RED	Reduced	NA	NA
Gdist:S169407_6947	BT20	10.142	0.0141	NA	No	No	No	No	No	No	No	NA	NA	No	NA	NA	NA	NA
Gdist:S30466_5523	BT20	10.157	0.0779	NA	No	No	No	No	No	Yes (0.047)	No	0.104318182	0.72269585	Yes	NA	NA	NA	NA
Gdist:S16320_4528	BT20	12.482	0.0245	NA	No	No	No	No	No	No	No	NA	NA	No	NA	NA	NA	NA
Gdist:S16642_4685	BT20	14.203	0.0143	NA	No	No	No	No	No	No	No	NA	NA	No	NA	NA	NA	NA
cDNA:S25899_3079	BT20	14.206	0.0024	NA	No	No	No	No	No	No	No	NA	NA	No	NA	NA	NA	NA
Gdist:S156350_814	BT20	19.256	0.0049	NA	No	No	No	No	No	No	No	NA	NA	No	NA	NA	NA	NA
SalHit:S78760_103	BT20	19.256	-0.0025	NA	No	No	No	No	No	No	No	NA	NA	No	NA	NA	NA	NA
Gdist:S6676_2498	BT20	19.765	0.0011	NA	No	No	No	No	No	No	No	NA	NA	No	NA	NA	NA	NA
Gdist:S257438_1496	BT20	19.853	-0.0074	NA	No	No	No	No	No	No	No	NA	NA	No	NA	NA	NA	NA
cDNA:S235071_667	BT20	21.693	0.0207	NA	No	No	No	No	No	Yes (0.002)	Yes (0.024)	NA	NA	No	NA	NA	NA	NA
SalHit:S642671_1637	BT20	21.804	-0.0082	NA	No	No	No	No	No	No	No	NA	NA	No	NA	NA	NA	NA
SalHit:S645775_422	BT20	22.666	0.0401	NA	No	No	No	No	No	No	No	0.632846715	0.65440981	Yes	NA	NA	NA	NA
Gdist:S465795_6468	BT20	27.599	0.0199	NA	Yes	No	No	No	No	No	No	NA	NA	No	NA	NA	NA	NA
SalHit:S148576_2204	BT20	28.235	0.0342	NA	No	No	No	No	No	Yes (0.022)	No	0.346071429	0.78166494	Yes	NA	NA	NA	NA
Gdist:S529519_5242	BT20	28.86	-0.0069	NA	No	No	No	No	No	No	No	NA	NA	No	NA	NA	NA	NA
Gdist:S390641_3261	BT20	29.485	-0.0079	NA	No	No	No	No	No	No	No	NA	NA	No	NA	NA	NA	NA
SalHit:S215479_2458	BT20	30.706	0.0049	NA	No	No	No	No	No	No	No	NA	NA	No	NA	NA	NA	NA
Gdist:S206726_2476	BT20	32.109	0.0031	NA	No	No	No	No	No	No	No	NA	NA	No	NA	NA	NA	NA
cDNA:S384781_3281	BT20	32.17	-0.0014	NA	No	No	No	No	No	No	No	NA	NA	No	NA	NA	NA	NA
Gdist:S93061_4537	BT20	32.183	0.0007	NA	No	No	No	No	No	No	No	NA	NA	No	NA	NA	NA	NA
SalHit:S168157_991	BT20	33.45	-0.0126	NA	No	No	No	No	No	No	No	NA	NA	No	NA	NA	NA	NA
cDNA:S309375_5264	BT20	35.345	0.0029	NA	No	No	No	No	No	No	No	NA	NA	No	NA	NA	NA	NA
Gdist:S100414_3290	BT20	35.645	0.0835	NA	No	No	No	No	No	No	No	0.916490895	0.17	Yes	NA	NA	NA	NA
Gdist:S268328_4748	BT20	35.984	0.0065	NA	No	No	No	No	No	No	No	NA	NA	No	NA	NA	NA	NA
SalHit:C123911743_415	BT20	38.846	-0.0108	NA	No	No	No	No	No	No	No	NA	NA	No	NA	NA	NA	NA
Gdist:S44854_5620	BT20	39.036	-0.0059	NA	No	No	No	No	No	No	No	NA	NA	No	NA	NA	NA	NA
cDNA:S142821_2593	BT20	39.039	0.0185	NA	No	No	No	No	No	Yes (0.014)	No	NA	NA	No	NA	NA	NA	NA
cDNA:S409144_10758	BT20	39.039	0.0165	NA	No	No	No	No	No	No	No	NA	NA	No	NA	NA	NA	NA
Gdist:S456287_4611	BT20	39.703	0.028	NA	Yes	No	No	No	No	No	No	NA	NA	No	NA	NA	NA	NA
Gdist:S315006_6423	BT20	39.703	0.0097	NA	No	No	No	No	No	No	No	NA	NA	No	NA	NA	NA	NA
cDNA:S741234_1247	BT20	39.704	0.0485	NA	No	No	No	No	No	No	No	0.893131188	0.27708661	Yes	NA	NA	NA	NA
cDNA:S741234_882	BT20	39.704	0.0316	NA	No	No	No	No	No	No	No	0.625060976	0.99074602	Yes	NA	NA	NA	NA
SalHit:S508212_1407	BT20	39.704	0.0284	NA	No	No	No	No	No	No	No	NA	NA	No	NA	NA	NA	NA
cDNA:S561676_2226	BT20	39.704	0.0099	NA	No	No	No	No	No	No	No	NA	NA	No	NA	NA	NA	NA
cDNA:C124635139_172	BT20	39.704	0.0053	NA	No	No	No	No	No	Yes (0.007)	Yes (0.049)	NA	NA	No	NA	NA	NA	NA
SalHit:S372646_3054	BT20	41.023	0.0316	NA	No	No	No	No	No	No	No	0.805828652	0.87029851	Yes	NA	NA	NA	NA
SalHit:S372646_403	BT20	41.023	0.0178	NA	No	No	No	No	No	No	No	NA	NA	No	NA	NA	NA	NA
cDNA:S833938_256	BT20	41.026	0.0391	NA	No	No	No	No	No	No	No	0.558273121	0.46026971	Yes	NA	NA	NA	NA
SalHit:S175742_1533	BT20	41.028	0.029	NA	No	No	No	No	No	No	No	NA	NA	No	NA	NA	NA	NA
Gdist:S186377_2059	BT20	41.03	0.0463	NA	No	No	No	No	No	No	No	0.988505976	0.30033333	Yes	NA	NA	NA	NA
cDNA:S200492_3963	BT20	41.797	0.022	NA	No	No	No	No	No	No	No	NA	NA	No	NA	NA	NA	NA
cDNA:S177784_5558	BT20	42.576	0.0433	NA	No	No	No	No	No	No	No	0.982691275	0.87029851	Yes	NA	NA	NA	NA
cDNA:S156230_1049	BT20	42.59	0.0266	NA	Yes	No	No	No	No	No	No	NA	NA	No	NA	NA	NA	NA
cDNA:S156230_8577	BT20	43.667	0.0092	NA	No	No	No	No	No	No	No	NA	NA	No	NA	NA	NA	NA
Gdist:S134417_1070	BT20	45.603	0.0425	NA	No	No	No	No	No	Yes (0.021)	No	0.826397601	0.37646053	Yes	NA	NA	NA	NA
cDNA:S835090_2041	BT20	45.603	0.0281	NA	No	No	No	No	No	No	No	NA	NA	No	NA	NA	NA	NA
Gdist:S204465_14058	BT20	45.603	-0.0012	NA	No	No	No	No	No	No	No	NA	NA	No	NA	NA	NA	NA
SalarSNP:ESTNV_36818_1287	BT20	46.773	0.007	NA	No	No	No	No	No	No	No	NA	NA	No	NA	NA	NA	NA
cDNA:S427565_10290	BT20	46.778	0.0343	NA	Yes	No	No	No	No	Yes (0.001)	Yes (0.017)	0.870811856	0.44161364	Yes	NA	NA	NA	NA
Gdist:S78385_7201	BT20	46.778	0.032	NA	No	No	No	No	No	No	No	0.635059809	0.60712604	Yes	NA	NA	NA	NA
Gdist:S354264_3505	BT20	46.778	0.0024	NA	No	No	No	No	No	No	No	NA	NA	No	NA	NA	NA	NA
Gdist:S90534_5337	BT20	46.782	0.0055	NA	No	No	No	No	No	No	No	0.982691275	0.8671301	Yes	NA	NA	NA	NA
Gdist:S459930_9324	BT20	46.786	0.016	NA	No	No	No	No	No	No	No	NA	NA	No	NA	NA	NA	NA
Gdist:S423638_6249	BT20	47.42	0.0221	NA	No	No	No	No	No	No	No	NA	NA	No	NA	NA	NA	NA
SalHit:S166075_4193	BT20	48.013	0.0634	NA	No	No	No	No	No	No	No	0.997	0.49897556	Yes	NA	NA	NA	NA
SalHit:S432900_3804	BT20	48.013	0.0138	NA	No	No	No	No	No	No	No	NA	NA	No	NA	NA	NA	NA
Gdist:S23355_11612	BT20	48.013	0.0104	NA	No	No	No	No	No	No	No	NA	NA	No	NA	NA	NA	NA
SalHit:S102823_1006	BT20	48.013	0.0034	NA	No	No	No	No	No	No	No	NA	NA	No	NA	NA	NA	NA
SalHit:S166075_5092	BT20	48.013	-0.0049	NA	No	No	No	No	No	No	No	NA	NA	No	NA	NA	NA	NA
Gdist:S269064_2292	BT20	48.013	-0.0066	NA	No	No	No	No	No	No	No	NA	NA	No	NA	NA	NA	NA
SalarSNP:ESTNV_30276_856	BT20	48.285	0.0155	NA	No	No	No	No	No	No	No	NA	NA	No	NA	NA	NA	NA
Gdist:S210723_7642	BT20	48.554	-0.0057	NA	No	No	No	No	No	No	No	NA	NA	No	NA	NA	NA	NA
LD:S429316_11581	BT20	48.811	0.0202	NA	No	No	No	No	No	No	No	NA	NA	No	NA	NA	NA	NA
SalarSNP:ESTNV_32130_141	BT20	48.811	0.0152	NA	No	No	No	No	No	No	No	NA	NA	No	NA	NA	NA	NA
Gdist:S382659_6762	BT20	48.811	0.0141	NA	No	No	No	No	No	No	No	NA	NA	No	NA	NA	NA	NA
Gdist:S781394_2998	BT20	48.811	0.0061	NA	No	No	No	No	No	No	No	NA	NA	No	NA	NA	NA	NA
Gdist:S388831_8558	BT20	48.811	0.0034	NA	No	No	No	No	No	No	No	NA	NA	No	NA	NA	NA	NA
cDNA:S216045_7099	BT20	48.811	-0.0017	NA	No	No	No	No	No	No	No	NA	NA	No	NA	NA	NA	NA
Gdist:S470803_5758	BT20	48.811	-0.0017	NA	No	No	No	No	No	No	No	NA	NA	No	NA	NA	NA	NA
cDNA:S216045_7664	BT20	48.811	-0.0041	NA	No	No	No	No	No	No	No	NA	NA	No	NA	NA	NA	NA
LD:S429316_2484	BT20	48.816	0.0019	NA	No	No	No	No	No	No	No	NA	NA	No	NA	NA	NA	NA
Gene:C124534064_203	BT20	51.027	-0.0139	NA	No	No	No	No	No	No	No	NA	NA	No	NA	NA	NA	NA
Gdist:S55915_12399	BT20	51.029	0.0353	NA	No	No	No	No	No	No	No	0.963553748	0.05976563	Yes	NA	NA	NA	NA
cDNA:S217532_351	BT20	51.031	0.0248	NA	No	No	No	No	No	No	No	NA	NA	No	NA	NA	NA	NA
Gdist:S592287_5141	BT20	51.033	0.0227	NA	No	No	No	No	No	Yes (0.004)	Yes (0.035)	NA	NA	No	NA	NA	NA	NA
LD:S336308_8856	BT20	51.033	0.0185	NA	No	No	No	No	No	No	No	NA	NA	No	NA	NA	NA	NA
cDNA:S823547_277	BT20	51.033	0.0141	NA	Yes	No	No	No	No	No	No	NA	NA	No	NA	NA	NA	NA
Gdist:S302235_680	BT20	51.033	0.0024	NA	Yes	No	No	No	No	No	No	NA	NA	No	NA	NA	NA	NA
LD:S336308_518	BT20	51.033	0.0901	NA	Yes	no	no	No	No	No	No	NA	NA	no	NA	NA	NA	NA
Gdist:S14496_6161	BT20	51.15	-0.0023	NA	No	No	No	No	No	No	No	NA	NA	No	NA	NA	NA	NA
Gdist:S29320_4825	BT20	51.264	-0.0042	NA	No	No	No	No	No	No	No	NA	NA	No	NA	NA	NA	NA
Gdist:S94010_4134	BT20	51.577	0.035	NA	No	No	No	No	No	No	No	0.682433921	0.26357143	Yes	NA	NA	NA	NA
Gdist:S170431_7731	BT20	51.89	0.0275	NA	No	No	No	No	No	No	No	NA	NA	No	NA	NA	NA	NA
Gdist:S170431_3226	BT20	52.193	0.0083	NA	No	No	No	No	No	No	No	NA	NA	No	NA	NA	NA	NA
cDNA:S347938_221	BT20	52.457	0.0239	NA	No	No	No	No	No	No	No	0.468256228	0.79388265	Yes	NA	NA	NA	NA
SalHit:S123549_2712	BT20	52.457	-0.0141	NA	No	No	No	No	No	No	No	NA	NA	No	NA	NA	NA	NA
Gdist:S792208_8877	BT20	53.065	0.0154	NA	No	No	No	No	No	No	No	NA	NA	No	NA	NA	NA	NA
cDNA:S141016_2579	BT20	53.065	0.011	NA	No	No	No	No	No	No	No	NA	NA	No	NA	NA	NA	NA
SalHit:S758078_2162	BT20	53.065	0.0049	NA	No	No	No	No	No	No	No	NA	NA	No	NA	NA	NA	NA
Gdist:S193142_8558	BT20	53.066	0.008	NA	No	No	No	No	No	Yes (0.047)	No	NA	NA	No	NA	NA	NA	NA
Gdist:S65245_4147	BT20	53.066	0.0067	NA	No	No	No	No	No	No	No	NA	NA	No	NA	NA	NA	NA
cDNA:S321236_5259	BT20	53.067	0.0098	NA	No	No	No	No	No	No	No	NA	NA	No	NA	NA	NA	NA
Gdist:S299064_4718	BT20	53.947	0.0213	NA	Yes	No	No	No	No	No	No	NA	NA	No	NA	NA	NA	NA

Gdist:S114372_2369	BT20	53.947	0.0188	NA	No	No	No	No	No	No	No	NA	NA	No	NA	NA	NA	NA
Gdist:S191174_5281	BT20	54.17	-0.0086	NA	No	No	No	No	No	No	No	NA	NA	No	NA	NA	NA	NA
Gdist:S42832_4896	BT20	54.392	0.0093	NA	No	No	No	No	No	No	No	NA	NA	No	NA	NA	NA	NA
SalHit:S604501_4136	BT20	54.836	0.0226	NA	No	No	No	No	No	No	No	NA	NA	No	NA	NA	NA	NA
cDNA:S398768_3382	BT20	54.836	0.0085	NA	No	No	No	No	No	No	No	NA	NA	No	NA	NA	NA	NA
SalHit:S768178_1923	BT20	54.836	0.0055	NA	No	No	No	No	No	No	No	NA	NA	No	NA	NA	NA	NA
Gdist:S462221_6287	BT20	54.931	0.0218	NA	No	No	No	No	No	No	No	NA	NA	No	NA	NA	NA	NA
Gdist:S81661_2699	BT20	54.939	-0.0078	NA	No	No	No	No	No	No	No	NA	NA	No	NA	NA	NA	NA
Gdist:S451658_3478	BT20	55.08	0.0476	NA	No	No	No	No	No	No	No	0.893131188	0.83111989	Yes	NA	NA	NA	NA
SalHit:S261794_1054	BT20	55.278	0.0222	NA	No	No	No	No	No	No	No	NA	NA	No	NA	NA	NA	NA
SalHit:S413354_2932	BT20	55.278	0.0172	NA	No	No	No	No	No	No	No	NA	NA	No	NA	NA	NA	NA
Gdist:S39356_11764	BT20	55.278	0.0135	NA	No	No	No	No	No	No	No	NA	NA	No	NA	NA	NA	NA
Gdist:S71172_14636	BT20	55.278	-0.0092	NA	No	No	No	No	No	No	No	NA	NA	No	NA	NA	NA	NA
SalHit:S187171_19839	BT21	0	0.0062	NA	No	No	No	No	No	No	No	NA	NA	No	NA	NA	NA	NA
SalHit:S187171_8005	BT21	0.01	0.0161	NA	No	No	No	No	No	No	No	NA	NA	No	NA	NA	NA	NA
Gdist:S68807_7174	BT21	0.015	0.0019	NA	No	No	No	No	No	No	No	NA	NA	No	NA	NA	NA	NA
Gdist:S228567_2066	BT21	0.015	-0.0023	NA	No	No	No	No	No	No	No	NA	NA	No	NA	NA	NA	NA
Gdist:S81214_2733	BT21	0.972	0.0297	NA	No	No	No	No	No	No	No	NA	NA	No	NA	NA	NA	NA
LD:S81214_3030	BT21	1.929	0.0147	NA	No	No	No	No	No	No	No	NA	NA	No	NA	NA	NA	NA
Gdist:S420256_1491	BT21	1.96	0.0098	NA	No	No	No	No	No	No	No	NA	NA	No	NA	NA	NA	NA
Gdist:S420256_7661	BT21	1.96	-0.0006	NA	No	No	No	No	No	Yes (0.048)	No	NA	NA	No	NA	NA	NA	NA
LD:S81214_21664	BT21	1.96	-0.0093	NA	No	No	No	No	No	No	No	NA	NA	No	NA	NA	NA	NA
Gdist:S93637_7164	BT21	6.011	0.0039	NA	No	No	No	No	No	No	No	NA	NA	No	NA	NA	NA	NA
Gdist:S82536_5953	BT21	6.66	0.0077	NA	No	No	No	No	No	Yes (0.023)	No	NA	NA	No	NA	NA	NA	NA
Gene:S346962_803	BT21	6.667	0.0118	NA	No	No	No	No	No	No	No	NA	NA	No	NA	NA	NA	NA
Gene:S346962_934	BT21	6.668	0.0015	NA	No	No	No	No	No	No	No	NA	NA	No	NA	NA	NA	NA
cDNA:S277616_2134	BT21	7.204	0.0279	NA	No	No	No	No	No	No	No	NA	NA	No	NA	NA	NA	NA
SalHit:S341408_5153	BT21	8.603	0.0545	NA	No	No	No	No	No	No	No	0.982691275	0.82025797	Yes	NA	NA	NA	NA
Gdist:S216916_439	BT21	10.291	-0.0131	NA	No	No	No	No	No	No	No	NA	NA	No	NA	NA	NA	NA
Gdist:S216916_10552	BT21	11.978	0.049	NA	No	No	No	No	No	No	No	0.419853516	0.17778169	Yes	NA	NA	NA	NA
SalHit:S249521_11216	BT21	18.256	0.0668	NA	No	No	No	No	No	No	No	0.188517857	0.37853627	Yes	NA	NA	NA	NA
Gdist:S465983_7394	BT21	18.287	0.0243	NA	No	No	No	No	No	No	No	NA	NA	No	NA	NA	NA	NA
Gdist:S465983_5990	BT21	18.287	0.0119	NA	No	No	No	No	No	No	No	NA	NA	No	NA	NA	NA	NA
Gdist:S386025_2013	BT21	19.168	0.0031	NA	No	No	No	No	No	No	No	NA	NA	No	NA	NA	NA	NA
Gdist:S386025_9401	BT21	19.86	-0.012	NA	No	No	No	No	No	Yes (0.014)	No	NA	NA	No	NA	NA	NA	NA
Gdist:S163921_1612	BT21	19.877	0.0327	NA	No	No	No	No	No	No	No	0	0.89481818	Yes	VAR INC	increased	NA	yes
Gdist:S163921_4754	BT21	19.901	0.0133	NA	No	No	No	No	No	No	No	NA	NA	No	NA	NA	NA	NA
Gdist:S23063_484	BT21	19.927	0.0022	NA	No	No	No	No	No	No	No	NA	NA	No	NA	NA	NA	NA
Gdist:S80512_5184	BT21	21.032	0.0085	NA	No	No	No	No	No	No	No	NA	NA	No	NA	NA	NA	NA
Gdist:S70723_8202	BT21	21.745	0.0395	none	No	No	No	No	No	Yes (0.038)	No	0.574795082	0.01821429	Yes	SKJ RED	NA	reduced	NA
Gdist:S70723_7943	BT21	22.42	0.0063	NA	No	No	No	No	No	Yes (0.014)	No	NA	NA	No	NA	NA	NA	NA
cDNA:S232968_4649	BT21	23.104	0.0154	NA	No	No	No	No	No	No	No	NA	NA	No	NA	NA	NA	NA
SalHit:S70344_7682	BT21	23.188	0.023	NA	No	No	No	No	No	No	No	0.946271739	0.97391984	Yes	NA	NA	NA	NA
cDNA:S802713_646	BT21	23.189	-0.0044	NA	No	No	No	No	No	No	No	NA	NA	No	NA	NA	NA	NA
Gdist:S222976_2044	BT21	25.283	0.0335	NA	No	No	No	No	No	No	No	0.793867925	0.21798387	Yes	NA	NA	NA	NA
Gdist:S21304_7473	BT21	26.093	0.0504	NA	No	No	No	No	No	No	No	0.171673228	0.69352273	Yes	NA	NA	NA	NA
Gdist:S104994_4018	BT21	26.903	0.0068	NA	No	No	No	No	No	No	No	NA	NA	No	NA	NA	NA	NA
Gdist:S37462_9253	BT21	29.168	0.0632	NA	No	No	No	No	No	No	No	0.7171875	0.21798387	Yes	NA	NA	NA	NA
cDNA:S220841_14465	BT21	29.168	0.0054	NA	No	No	No	No	No	No	No	NA	NA	No	NA	NA	NA	NA
Gdist:S257608_7713	BT21	29.197	-0.0099	NA	No	No	No	No	No	No	No	NA	NA	No	NA	NA	NA	NA
Gdist:S257608_4354	BT21	29.227	0.0066	NA	No	No	No	No	No	No	No	NA	NA	No	NA	NA	NA	NA
cDNA:S96652_4022	BT21	29.603	0.0399	NA	No	No	No	No	No	Yes (0.013)	No	0.926563433	0.77613306	Yes	NA	NA	NA	NA
cDNA:S366279_1007	BT21	29.603	0.0158	NA	No	No	No	No	No	No	No	NA	NA	No	NA	NA	NA	NA
cDNA:S396796_272	BT21	29.603	0.0123	NA	No	No	No	No	No	No	No	NA	NA	No	NA	NA	NA	NA
Gdist:S562528_6503	BT21	29.603	-0.0018	NA	No	No	No	No	No	No	No	NA	NA	No	NA	NA	NA	NA
Gdist:S562528_1768	BT21	29.603	-0.0025	NA	No	No	No	No	No	No	No	NA	NA	No	NA	NA	NA	NA
SalHit:S3164_6418	BT21	31.642	0.0068	NA	No	No	No	No	No	No	No	NA	NA	No	NA	NA	NA	NA
SalHit:S3164_1583	BT21	31.738	0.0173	NA	No	No	No	No	No	No	No	NA	NA	No	NA	NA	NA	NA
Gdist:S213246_4428	BT21	31.825	0.0384	NA	No	No	No	No	No	No	No	0.113613861	0.61532609	Yes	NA	NA	NA	NA
cDNA:S496464_1251	BT21	31.869	0.036	NA	No	No	No	No	No	No	No	0	0.84780928	Yes	VAR INC	increased	NA	yes
Gdist:S273006_5988	BT21	33.916	0.0319	NA	No	No	No	No	No	No	No	0.674134615	0.82025797	Yes	NA	NA	NA	NA
Gdist:S289517_3784	BT21	40.168	0.0114	NA	No	No	No	No	No	No	No	NA	NA	No	NA	NA	NA	NA
Gene:S82686_865	BT21	40.175	-0.0015	NA	No	No	No	No	No	No	No	NA	NA	No	NA	NA	NA	NA
Gdist:S56626_7438	BT21	40.182	0.0338	NA	No	No	No	No	No	No	No	0.233502907	0.2295	Yes	NA	NA	NA	NA
SalHit:C124761545_557	BT21	40.182	0.0289	NA	Yes	No	No	No	No	No	No	NA	NA	No	NA	NA	NA	NA
Gdist:S130202_7380	BT21	40.182	0.0181	NA	No	No	No	No	No	No	No	NA	NA	No	NA	NA	NA	NA
Gdist:S504912_4595	BT21	40.182	0.0078	NA	No	No	No	No	No	No	No	NA	NA	No	NA	NA	NA	NA
Gdist:S52374_4157	BT21	40.182	-0.0076	NA	No	No	No	No	No	No	No	NA	NA	No	NA	NA	NA	NA
SalHit:S90355_10517	BT21	40.182	-0.0111	NA	No	No	No	No	No	No	No	NA	NA	No	NA	NA	NA	NA
Gdist:S199547_1135	BT21	40.182	0.0356	NA	Yes	no	no	No	No	No	No	NA	NA	no	NA	NA	NA	NA
SalHit:S90355_11358	BT21	40.182	0.052	NA	No	No	No	No	No	Yes (0)	Yes (0.001)	0.079862637	0	Yes	SKJ INC (VAR m	NA	increased	NA
Gdist:S104771_5605	BT21	41.07	0.0326	NA	No	No	No	No	No	No	No	0.916490895	0.23538462	Yes	NA	NA	NA	NA
cDNA:S732543_1370	BT21	41.079	-0.0048	NA	Yes	No	No	No	No	No	No	NA	NA	No	NA	NA	NA	NA
Gdist:S121123_1695	BT21	41.111	0	NA	No	No	No	No	No	No	No	NA	NA	No	NA	NA	NA	NA
Gdist:S121123_6870	BT21	44.056	0.0276	NA	Yes	No	No	No	No	No	No	0.514568404	0.50388376	Yes	NA	NA	NA	NA
Gdist:S549050_7895	BT21	44.673	-0.0066	NA	No	No	No	No	No	Yes (0.021)	No	NA	NA	No	NA	NA	NA	NA
SalHit:S154900_1539	BT21	45.271	0.0337	NA	No	No	No	No	No	No	No	0.781630435	0.60473464	Yes	NA	NA	NA	NA
Gdist:S157374_15551	BT21	45.271	0.0267	NA	No	No	No	No	No	No	No	NA	NA	No	NA	NA	NA	NA
cDNA:S202817_2591	BT21	45.98	-0.0007	NA	No	No	No	No	No	No	No	NA	NA	No	NA	NA	NA	NA
Gdist:S166211_6461	BT21	46.693	0.0282	NA	No	No	No	No	No	No	No	NA	NA	No	NA	NA	NA	NA
cDNA:S268496_4507	BT21	46.693	0.0228	NA	No	No	No	No	No	No	No	NA	NA	No	NA	NA	NA	NA
Gdist:S21291_4318	BT21	46.693	0.0153	NA	No	No	No	No	No	No	No	NA	NA	No	NA	NA	NA	NA
Gdist:S52932_2025	BT21	46.693	0.0041	NA	No	No	No	No	No	No	No	NA	NA	No	NA	NA	NA	NA
Gdist:S52932_8715	BT21	46.693	-0.0033	NA	No	No	No	No	No	No	No	NA	NA	No	NA	NA	NA	NA
Gdist:S16205_6576	BT21	48.132	0.0173	NA	Yes	No	No	No	No	No	No	NA	NA	No	NA	NA	NA	NA
Gdist:S65166_7728	BT21	50.376	0.0099	NA	No	No	No	No	No	No	No	NA	NA	No	NA	NA	NA	NA
cDNA:S561043_583	BT21	50.376	-0.0013	NA	No	No	No	No	No	No	No	NA	NA	No	NA	NA	NA	NA
cDNA:S307975_3938	BT21	50.377	0.0221	NA	No	No	No	No	No	No	No	NA	NA	No	NA	NA	NA	NA
Gdist:S218503_6952	BT21	50.377	-0.0003	NA	No	No	No	No	No	No	No	NA	NA	No	NA	NA	NA	NA
Gdist:S18707_6553	BT21	51.186	0.0296	NA	No	No	No	No	No	No	No	NA	NA	No	NA	NA	NA	NA
Gdist:S394951_3073	BT21	51.199	0.0099	NA	No	No	No	No	No	No	No	NA	NA	No	NA	NA	NA	NA
Gdist:S311492_2101	BT21	51.239	-0.0036	NA	No	No	No	No	No	No	No	NA	NA	No	NA	NA	NA	NA
cDNA:S719929_1070	BT21	52.962	0.0248	NA	No	No	No	No	No	Yes (0.037)	No	NA	NA	No	NA	NA	NA	NA
SalHit:S798853_175	BT21	52.963	0.0544	NA	No	No	No	No	No	No	No	0.633830935	0.71957245	Yes	NA	NA	NA	NA
Gdist:S44003_8707	BT21	52.963	0.0272	NA	No	No	No	No	No	No	No	NA	NA	No	NA	NA	NA	NA
Gdist:S420788_3419	BT21	53.147	0.0327	NA	No	No	No	No	No	Yes (0.038)	No	0.893131188	0.88708726	Yes	NA	NA	NA	NA
Gdist:S367447_10254	BT21	53.336	0.0169	NA	No	No	No	No	No	No	No	NA	NA	No	NA	NA	NA	NA
Gdist:S423133_2595	BT21	53.511	0.0299	NA	No	No	No	No	No	No	No	NA	NA	No	NA	NA	NA	NA
SalHit:S600699_4332	BT21	53.631	0.0457	none	No	No	No	No	No	Yes (0.003)	Yes (0.024)	0.893131188	0.01821429	Yes	SKJ RED	NA	reduced	NA
cDNA:S773321_1853	BT21	53.632	0.0257	NA	No	No	No	No	No	No	No	NA	NA	No	NA	NA	NA	NA
SalHit:S146911_10466	BT21	53.633	0.0566	NA	No	No	No	No	No	No	No	0.651501168	0.27234	Yes	NA	NA	NA	NA
Gdist:S188492_4634	BT21	53.925	0.0241	NA	No	No	No	No	No	No	No	NA	NA	No	NA	NA	NA	NA
cDNA:S478587_2350	BT21	55.979	0.002	NA	No	No	No	No	No	No	No	NA	NA	No	NA	NA	NA	NA
Gdist:S290667_7179	BT22	0	0.0548	NA	No	No	No	No	No	No	No	0.453596654	0.3565678	Yes	NA	NA	NA	NA
Gdist:S290667_8789	BT22	1.946	-0.0097	NA	No	No	No	No	No	No	No	NA	NA	No	NA	NA	NA	NA
Gdist:S29318_6998	BT22	2.148	0.0106	NA	No	No	No	No	No	No	No	NA	NA	No	NA	NA	NA	NA
Gdist:S273904_7270	BT22	3.176	0.0417	NA	No	No	No	No	No	No	No	0.916490895	0.37646053	Yes	NA	NA	NA	NA
cDNA:S253487_2550	BT22	3.184	0.0264	NA	No	No	No	No	No	No	No	NA	NA	No	NA	NA	NA	NA
cDNA:S253487_1033	BT22	3.185	0.0325	NA	No	No	No	No	No	No	No	0.666092677	0.71968009	Yes	NA	NA	NA	NA
SalHit:S63194_3846	BT22	3.185	0.0254	NA	No	No	No	No	No	No	No	NA	NA	No	NA	NA	NA	NA
cDNA:S589758_2221	BT22	3.185	0.0198	NA	No	No	No	No	No	No	No	NA	NA	No	NA	NA	NA	NA
cDNA:S78222_1368	BT22	3.255	0.0077	NA	No	No	No	No	No	No	No	NA	NA	No	NA	NA	NA	NA
SalHit:S349460_2626	BT22	3.373	0.0626	NA	No	No	No	No	No	No	No	0.206348684	0.95357891	Yes	NA	NA	NA	NA
Gdist:S67352_2624	BT22	3.487	-0.0175	NA	No	No	No	No	No	No	No	NA	NA	No	NA	NA	NA	NA
Gdist:S387653_3590	BT22	3.87	0.0451	NA	No	No	No	No	No	No	No	0.682433921	0.74416667	Yes	NA	NA	NA	NA
cDNA:S100450_763	BT22	3.896	0.0067	NA	No	No	No	No	No	No	No	NA	NA	No	NA	NA	NA	NA
SalarSNP:GCR_cBin23273_Ctg	BT22	7.332	-0.0062	NA	No	No	No	No	No	No	No	NA	NA	No	NA	NA	NA	NA
Gdist:S46426_6692	BT22	10.768	-0.0067	NA	No	No	No	No	No	No	No	NA	NA	No	NA	NA	NA	NA
LD:S434590_10049	BT22	15.698	0.0018	NA	No	No	No	No	No	No	No	NA	NA	No	NA	NA	NA	NA
LD:S434590_1813	BT22	16.084	0.0083	NA	No	No	No	No	No	No	No	NA	NA	No	NA	NA	NA	NA
Gdist:S232218_8803	BT22	19.347	-0.0087	NA	No	No	No	No	No	No	No	NA	NA	No	NA	NA	NA	NA
Gdist:S232218_1291	BT22	19.348	0.0079	NA	No	No	No	No	No	No	No	NA	NA	No	NA	NA	NA	NA
cDNA:S337092_5433	BT22	23.774	-0.0124	NA	No	No	No	No	No	No	No	NA	NA	No	NA	NA	NA	NA
Gdist:S90795_4465	BT22	25.209	0.0091	NA	No	No	No	No	No	No	No	NA	NA	No	NA	NA	NA	NA
Gdist:S353988_9826	BT22	26.215	0.0224	NA	No	No	No	No	No	No	No	NA	NA	No	NA	NA	NA	NA
SalHit:S534814_2714	BT22	26.215	0.0182	NA	No	No	No	No	No	Yes (0.02)	No	NA	NA	No	NA	NA	NA	NA
Gdist:S665335_338	BT22	26.215	0.017	NA	No	No	No	No	No	No	No	NA	NA	No	NA	NA	NA	NA

cDNA:S405223_686	BT22	26.251	0.0255	NA	No	No	No	No	No	No	No	NA	NA	No	NA	NA	NA	NA
cDNA:S68433_2631	BT22	28.976	-0.0023	NA	No	No	No	No	No	No	No	NA	NA	No	NA	NA	NA	NA
cDNA:S137691_2198	BT22	31.408	0.0029	NA	No	No	No	No	No	No	No	NA	NA	No	NA	NA	NA	NA
Gdist:S663796_2369	BT22	31.816	-0.0031	NA	No	No	No	No	No	No	No	NA	NA	No	NA	NA	NA	NA
Gdist:S131843_326	BT22	32.281	0.0233	NA	No	No	No	No	No	Yes (0.025)	No	NA	NA	No	NA	NA	NA	NA
Gdist:S23960_7977	BT22	32.746	0.013	NA	No	No	No	No	No	Yes (0.025)	No	NA	NA	No	NA	NA	NA	NA
Gdist:S104238_4733	BT22	33.21	0.0739	NA	No	No	No	No	No	No	No	0.035416667	0.46026971	Yes	VAR INC	increased	NA	NA
Gdist:S36907_1864	BT22	33.688	-0.0029	NA	No	No	No	No	No	No	No	NA	NA	No	NA	NA	NA	NA
Gdist:S693953_2004	BT22	35.879	0.0307	NA	No	No	No	No	No	Yes (0.023)	No	0	0.57317221	Yes	VAR INC	increased	NA	yes
Gdist:S546493_916	BT22	36.492	0.008	NA	No	No	No	No	No	Yes (0.001)	Yes (0.016)	NA	NA	No	NA	NA	NA	NA
cDNA:S187829_864	BT22	37.111	0.0961	NA	No	No	No	No	No	No	No	0.774525862	0.92940304	Yes	NA	NA	NA	NA
cDNA:S187829_3326	BT22	37.112	0.057	NA	No	No	No	No	No	No	No	0.692970779	0.83441924	Yes	NA	NA	NA	NA
LD:S190550_1832	BT22	37.119	0.0558	NA	No	No	No	No	No	No	No	0.906372	0.89499248	Yes	NA	NA	NA	NA
Gdist:S345983_4350	BT22	39.108	0.0222	NA	No	No	No	No	No	No	No	NA	NA	No	NA	NA	NA	NA
cDNA:S467031_884	BT22	40.423	0.006	NA	No	No	No	No	No	No	No	NA	NA	No	NA	NA	NA	NA
SalHit:S241808_9086	BT22	40.431	0.0302	NA	No	No	No	No	No	No	No	0.906372	0.60417614	Yes	NA	NA	NA	NA
Gdist:S125305_3642	BT22	40.431	0.0285	NA	No	No	No	No	No	Yes (0.037)	No	NA	NA	No	NA	NA	NA	NA
cDNA:S27567_6436	BT22	40.431	0.0238	NA	No	No	No	No	No	No	No	NA	NA	No	NA	NA	NA	NA
cDNA:S27567_4317	BT22	40.431	-0.0063	NA	No	No	No	No	No	No	No	NA	NA	No	NA	NA	NA	NA
Gdist:S217006_2126	BT22	40.575	0.0095	NA	No	No	No	No	No	No	No	NA	NA	No	NA	NA	NA	NA
Gdist:S8836_997	BT22	40.604	0.0068	NA	No	No	No	No	No	No	No	NA	NA	No	NA	NA	NA	NA
Gdist:S42081_3723	BT22	41.42	0.001	NA	No	No	No	No	No	No	No	NA	NA	No	NA	NA	NA	NA
cDNA:S315457_3367	BT22	41.946	0.0309	NA	No	No	No	No	No	No	No	0.530792308	0.80649408	Yes	NA	NA	NA	NA
cDNA:S315457_6106	BT22	42.496	0.0231	NA	No	No	No	No	No	No	No	NA	NA	No	NA	NA	NA	NA
SalHit:S93239_12662	BT22	43.879	-0.0053	NA	No	No	No	No	No	No	No	NA	NA	No	NA	NA	NA	NA
LD:S571533_18083	BT22	43.955	0.03	NA	No	No	No	No	No	No	No	NA	NA	No	NA	NA	NA	NA
Gdist:S76453_2529	BT22	45.988	-0.011	NA	No	No	No	No	No	No	No	NA	NA	No	NA	NA	NA	NA
cDNA:S293894_14384	BT22	45.991	0.0384	NA	No	No	No	No	No	No	No	0.724528008	0.87029851	Yes	NA	NA	NA	NA
SalHit:S38409_6737	BT22	45.991	0.0319	NA	No	No	No	No	No	No	No	0.845991993	0.89481818	Yes	NA	NA	NA	NA
cDNA:S293894_13016	BT22	45.991	0.0163	NA	No	No	No	No	No	No	No	NA	NA	No	NA	NA	NA	NA
cDNA:S436246_4872	BT22	46.188	0.0136	NA	No	No	No	No	No	No	No	NA	NA	No	NA	NA	NA	NA
Gdist:S70340_919	BT22	46.296	0.0495	NA	No	No	No	Yes (0.022)	No	No	No	0.603632813	0.87616995	Yes	NA	NA	NA	NA
SalHit:S114598_3165	BT22	46.413	0.03	NA	No	No	No	No	No	No	No	NA	NA	No	NA	NA	NA	NA
SalHit:S114598_6898	BT22	46.413	0.0204	NA	No	No	No	No	No	No	No	NA	NA	No	NA	NA	NA	NA
Gdist:S137108_15317	BT22	46.413	0.0148	NA	No	No	No	No	No	No	No	NA	NA	No	NA	NA	NA	NA
Gdist:S57690_8477	BT22	46.413	0.0124	NA	No	No	No	No	No	No	No	NA	NA	No	NA	NA	NA	NA
cDNA:S30059_2725	BT22	46.413	0.0037	NA	No	No	No	No	No	No	No	NA	NA	No	NA	NA	NA	NA
Gdist:S658098_8466	BT22	46.413	-0.0035	NA	No	No	No	No	No	No	No	NA	NA	No	NA	NA	NA	NA
Gdist:S134518_5486	BT22	46.414	0.0067	NA	No	No	No	No	No	No	No	NA	NA	No	NA	NA	NA	NA
Gdist:S294633_6473	BT22	46.43	-0.0131	NA	No	No	No	No	No	No	No	NA	NA	No	NA	NA	NA	NA
Gdist:S203070_5914	BT22	46.45	-0.0056	NA	No	No	No	No	No	No	No	NA	NA	No	NA	NA	NA	NA
SalHit:S361210_2144	BT22	48.078	0.0741	NA	No	No	No	No	No	No	No	0.760560928	0.88732839	Yes	NA	NA	NA	NA
Gdist:S58449_6751	BT22	48.078	0.0482	NA	No	No	No	No	No	No	No	0.334223301	0.16303279	Yes	NA	NA	NA	NA
Gdist:S73431_1912	BT22	48.078	0.0376	NA	Yes	No	No	No	No	No	No	0.532767857	0.16303279	Yes	NA	NA	NA	NA
cDNA:S74998_8681	BT22	48.078	0.0334	NA	Yes	No	No	No	No	No	No	0.916490895	0.94595609	Yes	NA	NA	NA	NA
cDNA:S706337_4219	BT22	48.078	0.0172	NA	No	No	No	No	No	No	No	NA	NA	No	NA	NA	NA	NA
cDNA:S316629_667	BT22	48.078	0.0141	NA	No	No	No	No	No	Yes (0)	Yes (0.005)	NA	NA	No	NA	NA	NA	NA
SalHit:S820211_536	BT22	48.078	0.0105	NA	No	No	No	No	No	No	No	NA	NA	No	NA	NA	NA	NA
cDNA:S74998_6460	BT22	48.078	0.0073	NA	No	No	No	No	No	No	No	NA	NA	No	NA	NA	NA	NA
SalHit:S395278_4852	BT22	48.078	0.0071	NA	No	No	No	No	No	No	No	NA	NA	No	NA	NA	NA	NA
Gdist:S52634_4922	BT22	48.078	-0.0027	NA	No	No	No	No	No	No	No	NA	NA	No	NA	NA	NA	NA
cDNA:S314116_482	BT22	48.078	-0.0058	NA	No	No	No	No	No	No	No	NA	NA	No	NA	NA	NA	NA
cDNA:S74998_5369	BT22	48.078	0.1546	NA	Yes	no	no	No	No	No	No	NA	NA	no	NA	NA	NA	NA
cDNA:S414296_4127	BT22	48.079	0.0228	NA	Yes	No	No	No	No	No	No	NA	NA	No	NA	NA	NA	NA
Gdist:S181927_4963	BT22	48.082	0.0191	NA	No	No	No	No	No	Yes (0.023)	No	NA	NA	No	NA	NA	NA	NA
cDNA:S722226_5785	BT22	48.611	0.0136	NA	No	No	No	No	No	No	No	NA	NA	No	NA	NA	NA	NA
cDNA:S208909_3903	BT22	49.212	0.0273	NA	No	No	No	No	No	No	No	NA	NA	No	NA	NA	NA	NA
cDNA:S361636_1084	BT22	49.212	0.014	NA	No	No	No	No	No	No	No	NA	NA	No	NA	NA	NA	NA
SalHit:S707709_1535	BT22	49.212	0.0117	NA	No	No	No	No	No	No	No	NA	NA	No	NA	NA	NA	NA
Gdist:S539076_9358	BT22	49.212	0.004	NA	No	No	No	No	No	No	No	NA	NA	No	NA	NA	NA	NA
cDNA:S350970_3409	BT22	49.212	0.0035	NA	No	No	No	No	No	No	No	NA	NA	No	NA	NA	NA	NA
cDNA:S361636_6984	BT22	49.212	0	NA	No	No	No	No	No	Yes (0.014)	No	NA	NA	No	NA	NA	NA	NA
cDNA:S50805_3979	BT22	49.212	-0.0022	NA	No	No	No	No	No	No	No	NA	NA	No	NA	NA	NA	NA
Gdist:S8933_3315	BT22	49.589	0.0013	NA	No	No	No	No	No	No	No	NA	NA	No	NA	NA	NA	NA
cDNA:S251134_1830	BT22	49.589	0.0007	NA	No	No	No	No	No	No	No	NA	NA	No	NA	NA	NA	NA
cDNA:S304943_12655	BT22	49.589	0.035	PREDIC	No	No	No	No	No	No	No	0	0.48116071	Yes	VAR RED	Reduced	NA	NA
cDNA:S134413_3941	BT22	49.59	-0.0035	NA	Yes	No	No	No	No	No	No	NA	NA	No	NA	NA	NA	NA
cDNA:S696948_633	BT22	52.198	-0.0051	NA	No	No	No	No	No	No	No	NA	NA	No	NA	NA	NA	NA
cDNA:S250827_279	BT22	52.491	-0.0036	NA	No	No	No	No	No	No	No	NA	NA	No	NA	NA	NA	NA
cDNA:S41920_882	BT22	52.834	0.0614	NA	No	No	No	No	No	No	No	0.906372	0.95466066	Yes	NA	NA	NA	NA
cDNA:S434970_6835	BT22	52.834	0.0414	NA	No	No	No	No	No	No	No	0.285868421	0.95466066	Yes	NA	NA	NA	NA
cDNA:S638468_283	BT22	53.756	0.0564	NA	No	No	No	No	No	No	No	0.05961039	0.46026971	Yes	NA	NA	NA	NA
cDNA:S170983_2252	BT22	53.756	0.0199	NA	No	No	No	No	No	Yes (0.023)	No	NA	NA	No	NA	NA	NA	NA
Gdist:S319505_3792	BT22	53.756	0.0123	NA	No	No	No	No	No	No	No	NA	NA	No	NA	NA	NA	NA
cDNA:S577097_1137	BT22	53.757	0.0217	NA	No	No	No	No	No	No	No	NA	NA	No	NA	NA	NA	NA
cDNA:S251955_427	BT22	53.757	0.0139	NA	No	No	No	No	No	No	No	NA	NA	No	NA	NA	NA	NA
Gdist:S214784_4546	BT22	53.757	0.006	NA	No	No	No	No	No	No	No	NA	NA	No	NA	NA	NA	NA
SalHit:S161781_2673	BT22	53.776	0.0092	NA	No	No	No	No	No	No	No	NA	NA	No	NA	NA	NA	NA
SalHit:S161781_3028	BT22	53.792	0.0135	NA	No	No	No	No	No	No	No	NA	NA	No	NA	NA	NA	NA
SalHit:S161781_4577	BT22	53.813	0.0208	NA	No	No	No	No	No	No	No	NA	NA	No	NA	NA	NA	NA
cDNA:S116047_1442	BT22	53.907	0.0133	NA	No	No	No	No	No	No	No	NA	NA	No	NA	NA	NA	NA
Gdist:S229470_1027	BT22	54.198	0.0486	NA	No	No	No	No	No	Yes (0.001)	Yes (0.014)	0.468256228	0.77613306	Yes	NA	NA	NA	NA
cDNA:S535600_1124	BT22	54.198	0.0353	NA	Yes	No	No	No	No	No	No	0.374291845	0.71946429	Yes	NA	NA	NA	NA
cDNA:S538614_2376	BT22	54.198	0.014	NA	No	No	No	No	No	No	No	NA	NA	No	NA	NA	NA	NA
cDNA:S364146_769	BT22	54.198	0.0026	NA	No	No	No	No	No	No	No	NA	NA	No	NA	NA	NA	NA
Gene:S321905_1056	BT22	55.066	0.007	NA	No	No	No	No	No	No	No	NA	NA	No	NA	NA	NA	NA
Gdist:S58560_1190	BT23	0	0.0307	NA	Yes	No	No	No	No	No	No	0.166572581	0.73022727	Yes	NA	NA	NA	NA
cDNA:S698052_515	BT23	0.531	0.0174	NA	No	No	No	No	No	No	No	NA	NA	No	NA	NA	NA	NA
cDNA:S270982_6549	BT23	0.534	0.0232	NA	No	No	No	No	No	No	No	NA	NA	No	NA	NA	NA	NA
Gdist:S165611_4703	BT23	1.691	0.0051	NA	No	No	No	No	No	No	No	NA	NA	No	NA	NA	NA	NA
Gdist:S1657_6099	BT23	1.695	-0.0012	NA	No	No	No	No	No	No	No	NA	NA	No	NA	NA	NA	NA
Gdist:S425526_3665	BT23	2.156	-0.0125	NA	No	No	No	No	No	No	No	NA	NA	No	NA	NA	NA	NA
Gdist:S154339_585	BT23	2.637	-0.0053	NA	No	No	No	No	No	No	No	NA	NA	No	NA	NA	NA	NA
Gdist:S526823_10455	BT23	2.64	0.036	NA	No	No	No	No	No	No	No	0.906372	0.94321023	Yes	NA	NA	NA	NA
SalHit:S679571_2134	BT23	2.64	0.0184	NA	No	No	No	No	No	No	No	NA	NA	No	NA	NA	NA	NA
Gdist:S62832_3532	BT23	2.64	0.0109	NA	No	No	No	No	No	Yes (0.019)	No	NA	NA	No	NA	NA	NA	NA
Gdist:S3770_2725	BT23	2.64	0.0097	NA	No	No	No	No	No	No	No	NA	NA	No	NA	NA	NA	NA
Gdist:S273558_1153	BT23	2.64	0.0051	NA	No	No	No	No	No	No	No	NA	NA	No	NA	NA	NA	NA
Gdist:S549721_6289	BT23	2.64	0.0004	NA	No	No	No	No	No	No	No	NA	NA	No	NA	NA	NA	NA
Gdist:S11886_5301	BT23	2.64	-0.0018	NA	Yes	No	No	No	No	No	No	NA	NA	No	NA	NA	NA	NA
Gdist:S154339_6006	BT23	2.64	-0.0026	NA	No	No	No	No	No	No	No	NA	NA	No	NA	NA	NA	NA
Gdist:S62832_9453	BT23	2.64	-0.008	NA	No	No	No	No	No	No	No	NA	NA	No	NA	NA	NA	NA
Gdist:S605693_1243	BT23	2.745	-0.0018	NA	No	No	No	No	No	No	No	NA	NA	No	NA	NA	NA	NA
cDNA:S228964_6518	BT23	2.854	-0.0053	NA	No	No	No	No	No	No	No	NA	NA	No	NA	NA	NA	NA
Gdist:S734588_2445	BT23	2.953	-0.0075	NA	No	No	No	No	No	No	No	NA	NA	No	NA	NA	NA	NA
Gdist:S704139_293	BT23	3.015	0.0017	NA	No	No	No	No	No	No	No	NA	NA	No	NA	NA	NA	NA
Gdist:S491791_12029	BT23	3.09	0.0185	NA	No	No	No	No	No	No	No	NA	NA	No	NA	NA	NA	NA
Gdist:S178087_3099	BT23	3.466	0.0041	NA	No	No	No	No	No	No	No	NA	NA	No	NA	NA	NA	NA
cDNA:S173199_1673	BT23	3.602	0.0032	NA	No	No	No	No	No	Yes (0)	Yes (0.004)	NA	NA	No	NA	NA	NA	NA
Gdist:S234050_6365	BT23	3.728	0.0088	NA	No	No	No	No	No	No	No	NA	NA	No	NA	NA	NA	NA
Gdist:S20202_6726	BT23	3.843	0.144	NA	No	No	No	No	No	Yes (0)	Yes (0.009)	0.838743243	0.09807692	Yes	NA	NA	NA	NA
cDNA:S727135_932	BT23	3.843	0.0188	NA	No	No	No	No	No	No	No	NA	NA	No	NA	NA	NA	NA
Gdist:S365002_1741	BT23	3.843	0.0095	NA	No	No	No	No	No	No	No	NA	NA	No	NA	NA	NA	NA
Gdist:S365002_7169	BT23	3.843	0.0072	NA	No	No	No	No	No	No	No	NA	NA	No	NA	NA	NA	NA
Gdist:S365002_7483	BT23	3.843	0.0042	NA	No	No	No	No	No	No	No	NA	NA	No	NA	NA	NA	NA
Gdist:S199474_7751	BT23	3.843	0	NA	No	No	No	No	No	No	No	NA	NA	No	NA	NA	NA	NA
Gdist:S214897_7448	BT23	3.843	-0.0003	NA	No	No	No	No	No	No	No	NA	NA	No	NA	NA	NA	NA
Gdist:S235024_3562	BT23	3.843	-0.0022	NA	No	No	No	No	No	No	No	NA	NA	No	NA	NA	NA	NA
Gdist:S235024_4842	BT23	3.843	-0.0035	NA	No	No	No	No	No	No	No	NA	NA	No	NA	NA	NA	NA
cDNA:S124423_2529	BT23	3.843	-0.008	NA	No	No	No	No	No	Yes (0.014)	No	NA	NA	No	NA	NA	NA	NA
cDNA:S757328_1267	BT23	3.843	-0.0116	NA	No	No	No	No	No	No	No	NA	NA	No	NA	NA	NA	NA
cDNA:S125992_655	BT23	3.861	-0.0003	NA	No	No	No	No	No	No	No	0.57739782	0.54853698	Yes	NA	NA	NA	NA
cDNA:S273477_6161	BT23	5.875	-0.0023	NA	No	No	No	No	No	No	No	NA	NA	No	NA	NA	NA	NA
cDNA:S273477_6465	BT23	7.889	0.0018	NA	No	No	No	No	No	No	No	NA	NA	No	NA	NA	NA	NA
SalHit:S44456_5540	BT23	7.889	0.0303	NA	No	No	No	No	No	No	No	0.0095625	0.11839286	Yes	VAR INC	increased	NA	NA
SalHit:S44456_2602	BT23	7.89	0.0524	NA	No	No	No	No	No	No	No	0.589173387	0.55045673	Yes	NA	NA	NA	NA
Gdist:S546980_1705	BT23	7.89	0.0103	NA	No	No	No	No	No	Yes (0.043)	No	NA	NA	No	NA	NA	NA	NA

Gdist:S3660_5193	BT23	7.892	0.0122	NA	No	No	No	No	No	No	No	NA	NA	No	NA	NA	NA	NA
Gdist:S3660_7291	BT23	7.893	-0.0018	NA	No	No	No	No	No	No	No	NA	NA	No	NA	NA	NA	NA
Gdist:S734057_4564	BT23	9.755	0.0056	NA	No	No	No	No	No	No	No	NA	NA	No	NA	NA	NA	NA
Gdist:S272268_2840	BT23	11.617	0.0439	NA	No	No	No	No	No	No	No	0.60374036	0.26256356	Yes	NA	NA	NA	NA
Gdist:S491611_7462	BT23	11.622	0.0399	NA	No	No	No	No	No	No	No	0.176761364	0.85357204	Yes	NA	NA	NA	NA
Gdist:S473281_6421	BT23	11.622	0.0136	NA	No	No	No	No	No	No	No	NA	NA	No	NA	NA	NA	NA
SalHit:S142700_4079	BT23	12.298	-0.0008	NA	No	No	No	No	No	No	No	NA	NA	No	NA	NA	NA	NA
cDNA:S60720_2183	BT23	12.338	0.0104	NA	No	No	No	No	No	No	No	NA	NA	No	NA	NA	NA	NA
SalHit:S518988_677	BT23	12.857	0.0011	NA	No	No	No	No	No	No	No	NA	NA	No	NA	NA	NA	NA
SalHit:S331166_5798	BT23	12.874	0.027	NA	No	No	No	No	No	No	No	NA	NA	No	NA	NA	NA	NA
Gdist:S22388_1722	BT23	12.88	0.0489	NA	Yes	No	No	No	No	Yes(0.006) (in 28	Yes(0.024) (in 2	0	0.21798387	Yes	VAR INC	increased	NA	yes
cDNA:S121981_5646	BT23	14.474	0.0096	NA	No	No	No	No	No	No	No	NA	NA	No	NA	NA	NA	NA
Gdist:S229239_745	BT23	15.427	-0.0011	NA	No	No	No	No	No	No	No	NA	NA	No	NA	NA	NA	NA
Gdist:S316257_7482	BT23	18.784	0.0038	NA	No	No	No	No	No	No	No	NA	NA	No	NA	NA	NA	NA
cDNA:S43178_5577	BT23	20.004	0.0143	NA	No	No	No	No	No	No	No	NA	NA	No	NA	NA	NA	NA
Gdist:S263705_2977	BT23	20.612	0.009	NA	No	No	No	No	No	No	No	NA	NA	No	NA	NA	NA	NA
cDNA:S11384_4796	BT23	25.958	0.0028	NA	No	No	No	No	No	No	No	NA	NA	No	NA	NA	NA	NA
cDNA:S331455_6435	BT23	27.418	0.0276	NA	No	No	No	No	No	No	No	NA	NA	No	NA	NA	NA	NA
Gdist:S232302_6633	BT23	28.335	0.0024	NA	No	No	No	No	No	No	No	NA	NA	No	NA	NA	NA	NA
cDNA:S715975_734	BT23	28.354	-0.0088	NA	No	No	No	No	No	No	No	NA	NA	No	NA	NA	NA	NA
cDNA:S715975_2165	BT23	28.543	0.0095	NA	No	No	No	No	No	Yes (0.037)	No	NA	NA	No	NA	NA	NA	NA
Gdist:S187098_14972	BT23	29.966	0.0134	NA	No	No	No	No	No	No	No	NA	NA	No	NA	NA	NA	NA
Gdist:S26919_6612	BT23	30.018	0.0042	NA	No	No	No	No	No	No	No	NA	NA	No	NA	NA	NA	NA
Gdist:S210574_9971	BT23	30.019	-0.0051	NA	No	No	No	No	No	No	No	NA	NA	No	NA	NA	NA	NA
cDNA:S412895_1278	BT23	34.366	0.0009	NA	No	No	No	No	No	No	No	NA	NA	No	NA	NA	NA	NA
SalarSNP:ESTV_14058_333	BT23	34.411	0.0021	NA	No	No	No	No	No	No	No	NA	NA	No	NA	NA	NA	NA
cDNA:S797162_1001	BT23	34.418	0.0457	NA	No	No	No	No	No	No	No	0.982691275	0.59424107	Yes	NA	NA	NA	NA
Gdist:S286362_813	BT23	35.427	0.0223	NA	No	No	No	No	No	No	No	NA	NA	No	NA	NA	NA	NA
SalHit:S542331_3847	BT23	35.46	-0.0046	NA	No	No	No	No	No	No	No	NA	NA	No	NA	NA	NA	NA
Gdist:S226919_5606	BT23	35.492	-0.0081	NA	No	No	No	No	No	No	No	NA	NA	No	NA	NA	NA	NA
Gdist:S226919_5757	BT23	35.492	-0.0147	NA	No	No	No	No	No	No	No	NA	NA	No	NA	NA	NA	NA
cDNA:S774473_657	BT23	35.494	0.0001	NA	No	No	No	No	No	Yes (0.032)	No	NA	NA	No	NA	NA	NA	NA
cDNA:S774473_3443	BT23	35.497	0.0161	NA	No	No	No	No	No	No	No	NA	NA	No	NA	NA	NA	NA
Gdist:S46831_3287	BT23	36.673	0.0287	NA	No	No	No	No	No	Yes (0.023)	No	NA	NA	No	NA	NA	NA	NA
Gdist:S448320_4210	BT23	39.119	0.0283	NA	Yes	No	No	No	No	No	No	NA	NA	No	NA	NA	NA	NA
SalHit:S73487_2332	BT23	43.04	0.0119	NA	No	No	No	No	No	No	No	NA	NA	No	NA	NA	NA	NA
cDNA:S100721_1974	BT23	46.962	0.003	NA	No	No	No	No	No	No	No	NA	NA	No	NA	NA	NA	NA
Gdist:S40349_4848	BT23	47.797	-0.0027	NA	No	No	No	No	No	No	No	NA	NA	No	NA	NA	NA	NA
cDNA:S444883_764	BT23	51.106	0.0168	NA	No	No	No	No	No	Yes (0.006)	Yes (0.043)	NA	NA	No	NA	NA	NA	NA
Gdist:S417245_9728	BT23	51.333	0.0282	NA	No	No	No	No	No	No	No	NA	NA	No	NA	NA	NA	NA
Gdist:S417245_7993	BT23	51.334	0.0738	NA	No	No	No	No	No	No	No	0.078238636	0.53217391	Yes	NA	NA	NA	NA
Gdist:S417245_8819	BT23	51.334	0.0224	NA	No	No	No	No	No	No	No	NA	NA	No	NA	NA	NA	NA
Gdist:S49874_900	BT23	51.334	0.0008	NA	No	No	No	No	No	No	No	NA	NA	No	NA	NA	NA	NA
Gdist:S49874_6547	BT23	51.334	-0.0006	NA	No	No	No	No	No	No	No	NA	NA	No	NA	NA	NA	NA
LD:S71841_754	BT23	51.334	-0.0014	NA	No	No	No	No	No	No	No	NA	NA	No	NA	NA	NA	NA
Gdist:S414518_2848	BT23	52.043	-0.0016	NA	No	No	No	No	No	No	No	NA	NA	No	NA	NA	NA	NA
Gdist:S217753_4756	BT23	53.276	-0.0082	NA	No	No	No	No	No	Yes (0.035)	No	NA	NA	No	NA	NA	NA	NA
Gdist:S373249_6018	BT23	55.104	0.0875	NA	No	No	No	No	No	No	No	0.735576923	0.52391725	Yes	NA	NA	NA	NA
Gdist:S121954_1538	BT23	55.104	0.0056	NA	No	No	No	No	No	Yes (0.004)	Yes (0.035)	NA	NA	No	NA	NA	NA	NA
Gdist:S115075_8430	BT23	55.678	0.0532	NA	No	No	No	No	No	No	No	0.742859281	0.34814371	Yes	NA	NA	NA	NA
Gdist:S228061_9109	BT23	55.678	-0.0113	NA	No	No	No	No	No	No	No	NA	NA	No	NA	NA	NA	NA
Gdist:S71390_8982	BT23	56.556	0.0206	NA	No	No	No	No	No	No	No	NA	NA	No	NA	NA	NA	NA
Gdist:S89810_17658	BT23	57.602	0.0255	NA	Yes	no	no	No	No	No	No	NA	NA	no	NA	NA	NA	NA
Gdist:S89810_12533	BT23	57.603	0.0227	NA	No	No	No	No	No	No	No	NA	NA	No	NA	NA	NA	NA
Gdist:S89810_3116	BT23	57.604	-0.0038	NA	No	No	No	No	No	No	No	NA	NA	No	NA	NA	NA	NA
cDNA:S168405_2082	BT23	57.607	0.0435	NA	Yes	No	No	No	No	No	No	0.862473638	0.98752681	Yes	NA	NA	NA	NA
cDNA:S56850_3419	BT23	57.607	0.014	NA	No	No	No	No	No	No	No	NA	NA	No	NA	NA	NA	NA
cDNA:S56850_553	BT23	57.607	0.0085	NA	No	No	No	No	No	No	No	NA	NA	No	NA	NA	NA	NA
Gdist:S465440_554	BT23	57.607	-0.0016	NA	No	No	No	No	No	No	No	NA	NA	No	NA	NA	NA	NA
cDNA:S56850_1488	BT23	57.607	-0.0025	NA	No	No	No	No	No	No	No	NA	NA	No	NA	NA	NA	NA
Gdist:S552992_1902	BT23	57.607	-0.0037	NA	No	No	No	No	No	No	No	NA	NA	No	NA	NA	NA	NA
cDNA:S168405_3804	BT23	57.613	0.0275	NA	No	No	No	No	No	Yes (0.047)	No	NA	NA	No	NA	NA	NA	NA
SalHit:S704716_2081	BT23	57.637	0.0153	NA	No	No	No	No	No	Yes (0.002)	Yes (0.023)	NA	NA	No	NA	NA	NA	NA
Gene:S737940_1607	BT23	57.738	0.0145	NA	No	No	No	No	No	No	No	NA	NA	No	NA	NA	NA	NA
Gdist:S25947_4810	BT23	57.738	0.0134	NA	No	No	No	No	No	No	No	NA	NA	No	NA	NA	NA	NA
cDNA:S242162_9689	BT23	0	-0.0084	NA	No	No	No	No	No	No	No	NA	NA	No	NA	NA	NA	NA
cDNA:S543174_1716	BT24	0.881	0.049	NA	No	No	No	No	No	No	No	0.982691275	0.95357891	Yes	NA	NA	NA	NA
Gdist:S8683_4348	BT24	1.795	0.01	NA	No	No	No	No	No	No	No	NA	NA	No	NA	NA	NA	NA
Gdist:S41255_20254	BT24	8.235	0.0066	NA	No	No	No	No	No	Yes (0.007)	Yes (0.049)	NA	NA	No	NA	NA	NA	NA
Gdist:S99030_236	BT24	9.409	0.014	NA	No	No	No	No	No	No	No	NA	NA	No	NA	NA	NA	NA
cDNA:S247343_843	BT24	9.689	-0.0008	NA	No	No	No	No	No	No	No	NA	NA	No	NA	NA	NA	NA
Gdist:S11313_2046	BT24	9.987	0.0219	NA	No	No	No	No	No	No	No	NA	NA	No	NA	NA	NA	NA
Gdist:S29013_2139	BT24	10.285	0.023	NA	No	No	No	No	No	No	No	NA	NA	No	NA	NA	NA	NA
Gdist:S29013_4784	BT24	10.583	0.0275	NA	No	No	No	No	No	No	No	NA	NA	No	NA	NA	NA	NA
cDNA:S50428_3932	BT24	11.341	0.0067	NA	No	No	No	No	No	No	No	NA	NA	No	NA	NA	NA	NA
SalHit:S5395_4782	BT24	15.589	0.0294	NA	No	No	No	No	No	No	No	NA	NA	No	NA	NA	NA	NA
SalHit:S5395_7944	BT24	15.854	0.0151	NA	No	No	No	No	No	No	No	NA	NA	No	NA	NA	NA	NA
Gdist:S12805_1108	BT24	16.133	0.0642	NA	No	No	No	No	No	No	No	0.871576923	0.29401119	Yes	NA	NA	NA	NA
cDNA:S672808_3857	BT24	19.998	0.0175	NA	No	No	No	No	No	No	No	0.919848943	0.83441924	Yes	NA	NA	NA	NA
Gene:S310019_2288	BT24	20.186	0.0307	PREDIC'	No	No	No	No	No	No	No	0.60374036	0.01821429	Yes	SKJ RED	NA	reduced	NA
cDNA:S421584_3494	BT24	23.215	0.0333	NA	No	No	No	No	No	No	No	0.941752915	0.83441924	Yes	NA	NA	NA	NA
Gdist:S103101_1480	BT24	26.09	-0.0001	NA	No	No	No	No	No	No	No	NA	NA	No	NA	NA	NA	NA
Gdist:S33982_15022	BT24	28.442	-0.0027	NA	Yes	No	No	No	No	No	No	NA	NA	No	NA	NA	NA	NA
LD:S27039_17128	BT24	30.878	0.0012	NA	No	No	No	No	No	No	No	NA	NA	No	NA	NA	NA	NA
Gdist:S89304_4873	BT24	32.793	-0.0001	NA	No	No	No	No	No	Yes (0.007)	Yes (0.049)	NA	NA	No	NA	NA	NA	NA
Gene:S512153_3945	BT24	33.07	0.0033	NA	No	No	No	No	No	No	No	NA	NA	No	NA	NA	NA	NA
SalHit:S612770_3168	BT24	33.083	0.0237	NA	No	No	No	No	No	No	No	NA	NA	No	NA	NA	NA	NA
SalHit:S640273_2950	BT24	33.499	0.0011	NA	No	No	No	No	No	No	No	NA	NA	No	NA	NA	NA	NA
Gdist:S338641_200	BT24	34.326	-0.0017	NA	No	No	No	No	No	No	No	NA	NA	No	NA	NA	NA	NA
Gdist:S19359_475	BT24	34.327	-0.0038	NA	No	No	No	No	No	No	No	NA	NA	No	NA	NA	NA	NA
Gdist:S35568_4383	BT24	34.38	0.0337	NA	No	No	No	No	No	Yes (0.002)	Yes (0.024)	0	0.31739362	Yes	VAR INC	increased	NA	yes
Gdist:S73958_5187	BT24	37.954	0.0214	NA	No	No	No	No	No	No	No	NA	NA	No	NA	NA	NA	NA
Gdist:S137894_1019	BT24	38.022	0.0301	NA	No	No	No	No	No	No	No	0.158597561	0.74151316	Yes	NA	NA	NA	NA
Gdist:S44804_4498	BT24	39.564	0.0067	NA	No	No	No	No	No	No	No	NA	NA	No	NA	NA	NA	NA
cDNA:S387575_1543	BT24	40.345	-0.0048	NA	No	No	No	No	No	No	No	NA	NA	No	NA	NA	NA	NA
Gdist:S54123_414	BT24	40.346	-0.0014	NA	No	No	No	No	No	No	No	NA	NA	No	NA	NA	NA	NA
cDNA:S293652_756	BT24	40.347	0.0535	NA	No	No	No	No	No	Yes (0.039)	No	0.756052632	0.97060027	Yes	NA	NA	NA	NA
SalHit:S502826_4965	BT24	40.347	0.0036	NA	No	No	No	No	No	No	No	NA	NA	No	NA	NA	NA	NA
Gdist:S75100_906	BT24	40.347	-0.0053	NA	No	No	No	No	No	No	No	NA	NA	No	NA	NA	NA	NA
Gdist:S258470_3162	BT24	40.5	0.0252	NA	No	No	No	No	No	Yes (0.047)	No	NA	NA	No	NA	NA	NA	NA
Gdist:S188165_4687	BT24	41.165	-0.0053	NA	No	No	No	No	No	No	No	NA	NA	No	NA	NA	NA	NA
Gdist:S4888_7404	BT24	41.791	0.0035	NA	No	No	No	No	No	No	No	NA	NA	No	NA	NA	NA	NA
Gdist:S4888_7757	BT24	43.329	-0.0029	NA	No	No	No	No	No	No	No	NA	NA	No	NA	NA	NA	NA
Gdist:S43515_4911	BT24	43.966	0.0628	NA	No	No	No	No	No	Yes (0)	Yes (0.006)	0.895226151	0.23678571	Yes	NA	NA	NA	NA
cDNA:S177192_4107	BT24	43.966	0.061	NA	No	No	No	No	No	No	No	0.357476636	0.60417614	Yes	NA	NA	NA	NA
cDNA:S171993_2472	BT24	43.966	0.0073	NA	No	No	No	No	No	No	No	NA	NA	No	NA	NA	NA	NA
cDNA:S177192_3587	BT24	43.966	0.0051	NA	No	No	No	No	No	No	No	NA	NA	No	NA	NA	NA	NA
SalHit:S559536_6183	BT24	43.966	-0.0011	NA	No	No	No	No	No	No	No	NA	NA	No	NA	NA	NA	NA
Gdist:S354917_679	BT24	43.966	-0.0018	NA	No	No	No	No	No	No	No	NA	NA	No	NA	NA	NA	NA
cDNA:S639484_1049	BT24	44.603	0.0656	NA	No	No	No	No	No	No	No	0.862473638	0.41646028	Yes	NA	NA	NA	NA
cDNA:S526017_13088	BT24	44.712	0.0457	NA	No	No	No	No	No	No	No	0.916490895	0.41008413	Yes	NA	NA	NA	NA
Gdist:S748895_789	BT24	44.712	0.0331	NA	No	No	No	No	No	No	No	0.514568404	0.71946429	Yes	NA	NA	NA	NA
cDNA:S255038_318	BT24	45.372	0.0097	NA	No	No	No	No	No	No	No	NA	NA	No	NA	NA	NA	NA
Gdist:S246604_3421	BT24	45.378	-0.0007	NA	No	No	No	No	No	Yes (0.014)	No	NA	NA	No	NA	NA	NA	NA
Gdist:S18241_9917	BT24	46.086	0.0321	NA	No	No	No	No	No	Yes (0)	Yes (0.005)	0.838743243	0.82993937	Yes	NA	NA	NA	NA
Gdist:S86903_9017	BT24	46.141	0.0123	NA	No	No	No	No	No	Yes (0.019)	No	NA	NA	No	NA	NA	NA	NA
Gdist:S318863_3453	BT24	46.141	-0.0002	NA	No	No	No	No	No	No	No	NA	NA	No	NA	NA	NA	NA
cDNA:S33700_8300	BT24	46.703	0.018	NA	No	No	No	No	No	No	No	NA	NA	No	NA	NA	NA	NA
Gdist:S689160_923	BT24	46.708	0.0005	NA	No	No	No	No	No	No	No	NA	NA	No	NA	NA	NA	NA
cDNA:S194904_542	BT24	46.713	0.0288	NA	No	No	No	No	No	No	No	NA	NA	No	NA	NA	NA	NA
Gdist:S604697_6378	BT24	46.713	0.01	NA	No	No	No	No	No	No	No	NA	NA	No	NA	NA	NA	NA
cDNA:S553258_6312	BT24	46.713	0.0022	NA	No	No	No	No	No	No	No	NA	NA	No	NA	NA	NA	NA
Gdist:S157336_8348	BT24	46.713	-0.0124	NA	No	No	No	No	No	No	No	NA	NA	No	NA	NA	NA	NA
Gdist:S578094_2124	BT24	46.714	0.0082	NA	No	No	No	No	No	No	No	NA	NA	No	NA	NA	NA	NA
Gdist:S59402_5653	BT24	46.889	-0.0079	NA	No	No	No	No	No	No	No	NA	NA	No	NA	NA	NA	NA
Gdist:S109710_14627	BT24	47.603	0.0084	NA	No	No	No	No	No	No	No	NA	NA	No	NA	NA	NA	NA
cDNA:S443195_3302	BT24	47.728	0.0178	NA	Yes	No	No	No	No	No	No	NA	NA	No	NA	NA	NA	NA

cDNA:S822390_3958	BT24	47.848	0.1261	none	No	Yes	No	No	No	No	No	0.070833333	0.78238636	Yes	Outlier Varde	Outlier Var	NA	yes
Gdist:S456937_7358	BT24	47.849	0.0297	NA	No	No	No	No	No	No	No	NA	NA	No	NA	NA	NA	NA
cDNA:S98811_398	BT24	47.849	0.0028	NA	No	No	No	No	No	No	No	NA	NA	No	NA	NA	NA	NA
Gdist:S227567_6835	BT24	48.329	-0.0044	NA	No	No	No	No	No	No	No	NA	NA	No	NA	NA	NA	NA
cDNA:S541376_1738	BT24	48.996	0.0243	NA	No	No	No	No	No	No	No	NA	NA	No	NA	NA	NA	NA
cDNA:S127270_2739	BT24	49.687	-0.0036	NA	No	No	No	No	No	No	No	NA	NA	No	NA	NA	NA	NA
Gdist:S130372_767	BT24	50.461	0.0229	NA	No	No	No	No	No	Yes (0.001)	Yes (0.012)	NA	NA	No	NA	NA	NA	NA
Gdist:S25811_2468	BT24	50.461	0.0157	NA	Yes	No	No	No	No	No	No	NA	NA	No	NA	NA	NA	NA
Gdist:S497351_5246	BT24	50.557	0.0401	NA	No	No	No	No	No	Yes (0)	Yes (0.008)	0.6408	0.83111989	Yes	NA	NA	NA	NA
Gdist:S547003_6840	BT24	50.557	0.0149	NA	No	No	No	No	No	No	No	0.532767857	0.97060027	Yes	NA	NA	NA	NA
Gdist:S337806_15034	BT24	51.291	0.0531	NA	No	No	No	No	No	Yes (0)	Yes (0.009)	0.963070613	0.49909851	Yes	NA	NA	NA	NA
Gdist:S16214_3578	BT24	51.291	0.0195	NA	No	No	No	No	No	No	No	NA	NA	No	NA	NA	NA	NA
Gdist:S455166_11450	BT24	51.291	0.0112	NA	No	No	No	No	No	No	No	NA	NA	No	NA	NA	NA	NA
Gdist:S108805_7766	BT24	51.291	0.0102	NA	No	No	No	No	No	No	No	NA	NA	No	NA	NA	NA	NA
Gdist:S164686_8361	BT24	51.291	0.0069	NA	No	No	No	No	No	No	No	NA	NA	No	NA	NA	NA	NA
Gdist:S171891_6913	BT24	51.291	-0.0017	NA	No	No	No	No	No	No	No	NA	NA	No	NA	NA	NA	NA
cDNA:S739704_2409	BT24	51.291	-0.0046	NA	No	No	No	No	No	No	No	NA	NA	No	NA	NA	NA	NA
Gdist:S53895_6775	BT24	51.292	-0.0084	NA	No	No	No	No	No	No	No	NA	NA	No	NA	NA	NA	NA
Gene:S281001_1853	BT24	51.896	0.0054	NA	No	No	No	No	No	No	No	NA	NA	No	NA	NA	NA	NA
Gdist:S120406_890	BT24	51.896	0.0036	NA	No	No	No	No	No	No	No	NA	NA	No	NA	NA	NA	NA
SalarSNP:GCR_rBin8898_Ctg1_	BT24	51.897	0.0149	NA	No	No	No	No	No	No	No	NA	NA	No	NA	NA	NA	NA
Gdist:S46974_8627	BT24	51.99	0.0062	NA	No	No	No	No	No	No	No	NA	NA	No	NA	NA	NA	NA
Gdist:S46974_4883	BT24	52.212	0.0091	NA	No	No	No	No	No	No	No	NA	NA	No	NA	NA	NA	NA
SalHit:S355279_1207	BT24	54.287	0.0069	NA	No	No	No	No	No	No	No	NA	NA	No	NA	NA	NA	NA
Gdist:S459471_8936	BT25	0	0.0427	NA	Yes	No	No	No	No	No	No	0.601934211	0.1434375	Yes	NA	NA	NA	NA
cDNA:S495609_518	BT25	0.053	-0.0134	NA	No	No	No	No	No	No	No	NA	NA	No	NA	NA	NA	NA
Gdist:S237603_6472	BT25	0.059	0.002	NA	No	No	No	No	No	No	No	NA	NA	No	NA	NA	NA	NA
Gdist:S146436_7185	BT25	0.769	0.082	NA	Yes	no	no	No	No	No	No	NA	NA	no	NA	NA	NA	NA
Gdist:S120580_14694	BT25	6.729	-0.0037	NA	No	No	No	No	No	No	No	NA	NA	No	NA	NA	NA	NA
cDNA:S85734_1781	BT25	6.83	0.0025	NA	No	No	No	No	No	No	No	NA	NA	No	NA	NA	NA	NA
SalHit:S386453_5112	BT25	7.582	0.0055	NA	No	No	No	No	No	No	No	NA	NA	No	NA	NA	NA	NA
SalHit:S386453_3287	BT25	8.336	-0.0031	NA	No	No	No	No	No	No	No	NA	NA	No	NA	NA	NA	NA
Gdist:S211063_4803	BT25	9.365	0.0094	NA	No	No	No	No	No	Yes (0.002)	Yes (0.024)	NA	NA	No	NA	NA	NA	NA
Gdist:S78690_4943	BT25	9.514	0.0797	none	No	Yes	No	No	No	No	No	0.0095625	0.66132237	Yes	VAR RED + Outl	Reduced	NA	yes
Gdist:S78690_7641	BT25	9.651	0.0053	NA	No	No	No	No	No	Yes (0.007)	Yes (0.047)	NA	NA	No	NA	NA	NA	NA
Gdist:S166346_1746	BT25	9.845	0.0092	NA	No	No	No	No	No	Yes (0.035)	No	NA	NA	No	NA	NA	NA	NA
Gdist:S124786_413	BT25	9.885	0.0066	NA	No	No	No	No	No	No	No	NA	NA	No	NA	NA	NA	NA
Gdist:S196777_6053	BT25	17.366	0.0427	NA	No	No	No	No	No	No	No	0.357476636	0.37626359	Yes	NA	NA	NA	NA
Gdist:S196777_4226	BT25	17.508	0.02	NA	No	No	No	No	No	No	No	NA	NA	No	NA	NA	NA	NA
Gdist:S361831_4422	BT25	18.136	0.027	NA	No	No	No	No	No	No	No	NA	NA	No	NA	NA	NA	NA
Gdist:S361831_2035	BT25	18.299	0.0019	NA	No	No	No	No	No	No	No	NA	NA	No	NA	NA	NA	NA
SalHit:S365449_7529	BT25	18.309	-0.005	NA	No	No	No	No	No	No	No	NA	NA	No	NA	NA	NA	NA
Gdist:S218837_1880	BT25	21.914	0.0256	NA	No	No	No	No	No	No	No	NA	NA	No	NA	NA	NA	NA
SalHit:S268906_11932	BT25	21.916	0.0083	NA	No	No	No	No	No	No	No	NA	NA	No	NA	NA	NA	NA
Gdist:S335726_9602	BT25	22.022	0.0028	NA	No	No	No	No	No	No	No	NA	NA	No	NA	NA	NA	NA
LD:S546671_9492	BT25	24.94	-0.0004	NA	No	No	No	No	No	No	No	NA	NA	No	NA	NA	NA	NA
Gdist:S92266_3866	BT25	25.157	0.0176	NA	No	No	No	No	No	No	No	NA	NA	No	NA	NA	NA	NA
LD:S546671_1046	BT25	25.157	0.0108	NA	No	No	No	No	No	Yes (0.014)	No	NA	NA	No	NA	NA	NA	NA
Gdist:S248557_1363	BT25	25.157	0.0089	NA	No	No	No	No	No	No	No	NA	NA	No	NA	NA	NA	NA
Gdist:S270087_4390	BT25	25.157	0.0012	NA	No	No	No	No	No	No	No	NA	NA	No	NA	NA	NA	NA
Gdist:S571608_843	BT25	26.697	0.0192	NA	No	No	No	No	No	No	No	NA	NA	No	NA	NA	NA	NA
Gdist:S224720_948	BT25	26.697	0.0053	NA	No	No	No	No	No	No	No	NA	NA	No	NA	NA	NA	NA
Gdist:S235970_6941	BT25	26.697	0.0005	NA	No	No	No	No	No	No	No	NA	NA	No	NA	NA	NA	NA
Gdist:S571608_4799	BT25	26.697	-0.0114	NA	No	No	No	No	No	No	No	NA	NA	No	NA	NA	NA	NA
SalHit:S261274_7888	BT25	26.698	0.0413	NA	No	No	No	No	No	No	No	0.176308594	0.16207627	Yes	NA	NA	NA	NA
SalHit:S261274_20497	BT25	27.002	0.0001	NA	No	No	No	No	No	No	No	NA	NA	No	NA	NA	NA	NA
cDNA:S194179_14559	BT25	27.297	0.0191	NA	No	No	No	No	No	No	No	NA	NA	No	NA	NA	NA	NA
Gdist:S484971_4342	BT25	27.593	0.0117	NA	No	No	No	No	No	No	No	NA	NA	No	NA	NA	NA	NA
cDNA:S490342_360	BT25	27.679	0.0114	NA	No	No	No	No	No	No	No	NA	NA	No	NA	NA	NA	NA
cDNA:S490342_1168	BT25	28.451	0.0005	NA	No	No	No	No	No	No	No	NA	NA	No	NA	NA	NA	NA
Gdist:S520769_6596	BT25	31.675	0.0077	NA	No	No	No	No	No	No	No	NA	NA	No	NA	NA	NA	NA
cDNA:S27464_5751	BT25	32.75	0.0194	NA	No	No	No	No	No	Yes (0.014)	No	NA	NA	No	NA	NA	NA	NA
cDNA:S27464_3079	BT25	32.752	0.0067	NA	No	No	No	No	No	No	No	NA	NA	No	NA	NA	NA	NA
cDNA:S178550_6364	BT25	32.754	0.0635	NA	No	No	No	No	No	No	No	0.682433921	0.33763889	Yes	NA	NA	NA	NA
Gdist:S74727_1161	BT25	32.754	0.0411	NA	Yes	No	No	No	No	No	No	0.365723684	0.87844814	Yes	NA	NA	NA	NA
Gdist:S74727_1735	BT25	32.755	0.0118	NA	No	No	No	No	No	No	No	NA	NA	No	NA	NA	NA	NA
Gdist:S5781_4187	BT25	32.755	-0.0018	NA	No	No	No	No	No	No	No	NA	NA	No	NA	NA	NA	NA
SalHit:S146115_2865	BT25	33.239	0.0152	NA	No	No	No	No	No	Yes (0.023)	No	NA	NA	No	NA	NA	NA	NA
Gdist:S111849_1134	BT25	33.681	0.043	NA	No	No	No	No	No	No	No	0.189893617	0.42304147	Yes	NA	NA	NA	NA
Gdist:S68113_3175	BT25	33.681	0.0195	NA	No	No	No	No	No	Yes (0.007)	Yes (0.049)	NA	NA	No	NA	NA	NA	NA
Gdist:S68113_4309	BT25	33.681	0.0119	NA	No	No	No	No	No	No	No	NA	NA	No	NA	NA	NA	NA
Gdist:S212541_13018	BT25	33.729	-0.0042	NA	No	No	No	No	No	No	No	NA	NA	No	NA	NA	NA	NA
Gdist:S427034_13504	BT25	33.804	-0.0046	NA	No	No	No	No	No	No	No	NA	NA	No	NA	NA	NA	NA
Gdist:S427034_10898	BT25	33.883	-0.005	NA	No	No	No	No	No	No	No	NA	NA	No	NA	NA	NA	NA
Gdist:S95767_499	BT25	33.973	0.0289	NA	No	No	No	No	No	No	No	NA	NA	No	NA	NA	NA	NA
LD:S192579_16897	BT25	34.063	0.0502	NA	No	No	No	No	No	No	No	0.810042056	0.35534024	Yes	NA	NA	NA	NA
Gdist:S95767_7622	BT25	34.063	0.0286	NA	No	No	No	No	No	No	No	NA	NA	No	NA	NA	NA	NA
cDNA:S623208_2215	BT25	34.063	0.0137	NA	No	No	No	No	No	No	No	NA	NA	No	NA	NA	NA	NA
Gdist:S95767_8921	BT25	34.063	0.011	NA	No	No	No	No	No	No	No	NA	NA	No	NA	NA	NA	NA
Gdist:S28109_1273	BT25	34.474	0.0195	NA	No	No	No	No	No	No	No	NA	NA	No	NA	NA	NA	NA
cDNA:S458790_919	BT25	34.93	0.0659	NA	No	No	No	No	No	Yes (0.013)	No	0.896875	0.54853698	Yes	NA	NA	NA	NA
Gdist:S177676_2877	BT25	34.931	0.0118	NA	No	No	No	No	No	No	No	NA	NA	No	NA	NA	NA	NA
Gdist:S53259_10982	BT25	35.462	0.0167	NA	No	No	No	No	No	No	No	NA	NA	No	NA	NA	NA	NA
cDNA:S301968_735	BT25	36.738	-0.0022	NA	No	No	No	No	No	No	No	NA	NA	No	NA	NA	NA	NA
Gdist:S407303_5611	BT25	36.777	-0.0067	NA	No	No	No	No	No	No	No	NA	NA	No	NA	NA	NA	NA
Gdist:S132532_3332	BT25	36.969	0.0049	NA	No	No	No	No	No	No	No	NA	NA	No	NA	NA	NA	NA
Gdist:S132532_5276	BT25	36.978	0.0191	NA	No	No	No	No	No	Yes (0.048)	No	NA	NA	No	NA	NA	NA	NA
Gdist:S54358_3770	BT25	36.978	0.0146	NA	No	No	No	No	No	No	No	NA	NA	No	NA	NA	NA	NA
Gdist:S102093_4513	BT25	36.978	0.012	NA	No	No	No	No	No	No	No	NA	NA	No	NA	NA	NA	NA
Gdist:S557219_1096	BT25	36.978	0.0099	NA	No	No	No	No	No	No	No	NA	NA	No	NA	NA	NA	NA
SalHit:S312971_3029	BT25	36.979	0.0316	NA	Yes	No	No	No	No	No	No	0.93810219	0.87819512	Yes	NA	NA	NA	NA
Gdist:S58950_9765	BT25	40.744	0.0102	NA	Yes	No	No	No	No	No	No	NA	NA	No	NA	NA	NA	NA
Gdist:S48132_4545	BT25	40.844	0.0275	NA	No	No	No	No	No	No	No	NA	NA	No	NA	NA	NA	NA
Gdist:S40452_2363	BT25	40.945	0.0191	NA	No	No	No	No	No	No	No	0.890122881	0.31320652	Yes	NA	NA	NA	NA
Gdist:S193033_8543	BT25	40.945	0.0203	NA	No	No	No	No	No	No	No	NA	NA	No	NA	NA	NA	NA
SalarSNP:GCR_hBin20519_Ctg	BT25	40.945	0.0095	NA	No	No	No	No	No	No	No	NA	NA	No	NA	NA	NA	NA
Gdist:S444429_4794	BT25	40.945	0.0033	NA	No	No	No	No	No	No	No	NA	NA	No	NA	NA	NA	NA
Gdist:S27139_8838	BT25	40.945	0.0001	NA	Yes	No	No	No	No	No	No	NA	NA	No	NA	NA	NA	NA
Gdist:S89055_8031	BT25	40.945	-0.0035	NA	No	No	No	No	No	No	No	NA	NA	No	NA	NA	NA	NA
Gdist:S40452_7717	BT25	40.945	-0.0065	NA	Yes	No	No	No	No	No	No	NA	NA	No	NA	NA	NA	NA
SalHit:S577812_3658	BT25	40.945	0.0695	PREDIC	No	Yes	No	No	No	No	No	0.870811856	0.84677586	Yes	Outlier Varde	Outlier Var	NA	yes
SalHit:S718588_3898	BT25	40.947	0.0499	NA	Yes	No	No	No	No	No	No	0.664111239	0.83441924	Yes	NA	NA	NA	NA
Gdist:S90455_1615	BT25	40.949	0.0419	NA	No	No	No	No	No	No	No	0.023418367	0.34793675	Yes	VAR INC	increased	NA	yes
SalarSNP:GCR_hBin7522_Ctg1_	BT25	40.952	-0.0107	NA	No	No	No	No	No	No	No	NA	NA	No	NA	NA	NA	NA
cDNA:S172462_1007	BT25	41.515	-0.0042	NA	No	No	No	No	No	No	No	NA	NA	No	NA	NA	NA	NA
SalHit:S795010_298	BT25	42.079	0.0454	NA	No	No	No	No	No	No	No	0.442817308	0.84677586	Yes	NA	NA	NA	NA
cDNA:S456769_2371	BT25	42.082	-0.0041	NA	No	No	No	No	No	No	No	NA	NA	No	NA	NA	NA	NA
SalHit:S62224_1665	BT25	42.083	-0.0031	NA	No	No	No	No	No	No	No	NA	NA	No	NA	NA	NA	NA
SalHit:S286928_2598	BT25	42.165	0.0432	NA	No	No	No	No	No	No	No	0.838743243	0.21798387	Yes	NA	NA	NA	NA
cDNA:S479169_1824	BT25	42.287	0.0117	NA	No	No	No	No	No	No	No	NA	NA	No	NA	NA	NA	NA
Gdist:S50999_2944	BT25	43.354	-0.0018	NA	No	No	No	No	No	No	No	NA	NA	No	NA	NA	NA	NA
Gene:C124177984_226	BT25	43.363	0.0052	NA	No	No	No	No	No	No	No	NA	NA	No	NA	NA	NA	NA
Gene:S290628_5233	BT25	43.364	-0.0074	NA	No	No	No	No	No	No	No	NA	NA	No	NA	NA	NA	NA
Gdist:S108308_5065	BT25	43.401	-0.0085	NA	No	No	No	No	No	No	No	NA	NA	No	NA	NA	NA	NA
Gdist:S46704_6509	BT26	0	0.0036	NA	No	No	No	No	No	No	No	NA	NA	No	NA	NA	NA	NA
Gdist:S46704_5064	BT26	0.013	0.0149	NA	No	No	No	No	No	No	No	NA	NA	No	NA	NA	NA	NA
Gdist:S525671_8405	BT26	1.025	0.0352	NA	No	No	No	No	No	No	No	0.862473638	0.46026971	Yes	NA	NA	NA	NA
cDNA:S151021_283	BT26	3.221	0.0151	NA	No	No	No	No	No	No	No	NA	NA	No	NA	NA	NA	NA
Gdist:S154739_6213	BT26	4.707	0.0347	NA	No	No	No	No	No	No	No	0.150491803	0.92404832	Yes	NA	NA	NA	NA
Gdist:S110827_3868	BT26	6.193	0.0036	NA	No	No	No	No	No	No	No	NA	NA	No	NA	NA	NA	NA
Gdist:S147033_6365	BT26	6.23	0.0023	NA	No	No	No	No	No	No	No	NA	NA	No	NA	NA	NA	NA
Gene:S402069_249	BT26	6.252	0.0053	NA	No	No	No	No	No	No	No	NA	NA	No	NA	NA	NA	NA
SalHit:S114474_2548	BT26	6.4	0.03	NA	No	No	No	No	No	No	No	NA	NA	No	NA	NA	NA	NA
Gdist:S34865_6360	BT26	9.495	-0.0042	NA	No	No	No	No	No	No	No	NA	NA	No	NA	NA	NA	NA
cDNA:S16389_833	BT26	9.816	0.0183	NA	No	No	No	No	No	No	No	NA	NA	No	NA	NA	NA	NA
Gdist:S131798_5083	BT26	10.136	0.0184	NA	No	No	No	No	No	No	No	NA	NA	No	NA	NA	NA	NA
Gdist:S240257_3828	BT26	13.509	0.0212	NA	No	No	No	No	No	No	No	NA	NA	No	NA	NA	NA	NA

Gdist:S455821_3746	BT26	32.038	-0.0092	NA	No	No	No	No	No	No	No	NA	NA	No	NA	NA	NA	NA
Gdist:S125070_10203	BT26	33.115	0.0364	NA	No	No	No	No	No	No	No	0.928928571	0.56791921	Yes	NA	NA	NA	NA
Gdist:S100441_8833	BT26	33.128	0.0202	NA	No	No	No	No	No	No	No	NA	NA	No	NA	NA	NA	NA
Gdist:S352773_7123	BT26	33.128	-0.0019	NA	No	No	No	No	No	No	No	NA	NA	No	NA	NA	NA	NA
Gdist:S24649_5125	BT26	33.128	-0.0106	NA	No	No	No	No	No	No	No	NA	NA	No	NA	NA	NA	NA
Gdist:S289480_5417	BT26	33.131	0.0717	NA	No	No	No	No	No	Yes (0.043)	No	0.233502907	0.73347996	Yes	NA	NA	NA	NA
Gdist:S289480_4758	BT26	33.134	0.0341	NA	Yes	No	No	No	No	No	No	0.988505976	0.82025797	Yes	NA	NA	NA	NA
Gdist:S37890_4546	BT26	33.21	0.0389	NA	No	No	No	No	No	No	No	0.565105932	0.46026971	Yes	NA	NA	NA	NA
Gdist:S107890_2224	BT26	33.288	-0.0119	NA	No	No	No	No	No	No	No	NA	NA	No	NA	NA	NA	NA
Gdist:S653836_6436	BT26	35.31	0.0072	NA	No	No	No	No	No	Yes (0.007)	Yes (0.049)	0.916490895	0.9833502	Yes	NA	NA	NA	NA
Gdist:S8305_15065	BT26	35.31	-0.0064	NA	No	No	No	No	No	No	No	NA	NA	No	NA	NA	NA	NA
Gdist:S8305_8664	BT26	35.311	-0.0072	NA	No	No	No	No	No	No	No	NA	NA	No	NA	NA	NA	NA
SalHit:S140459_3613	BT26	36.82	0.0013	NA	No	No	No	No	No	No	No	NA	NA	No	NA	NA	NA	NA
Gdist:S58098_2927	BT26	36.843	0.0288	NA	No	No	No	No	No	No	No	NA	NA	No	NA	NA	NA	NA
SalHit:S787651_531	BT26	38.566	0.0101	NA	No	No	No	No	No	No	No	NA	NA	No	NA	NA	NA	NA
Gdist:S133717_6434	BT26	38.924	-0.0021	NA	No	No	No	No	No	No	No	NA	NA	No	NA	NA	NA	NA
SalHit:S83418_6389	BT26	40.464	0.0238	NA	No	No	No	No	No	No	No	NA	NA	No	NA	NA	NA	NA
SalHit:S503742_5592	BT26	40.464	-0.0115	NA	No	No	No	No	No	No	No	NA	NA	No	NA	NA	NA	NA
Gdist:S12575_1547	BT26	40.615	0.0195	NA	Yes	No	No	No	No	No	No	NA	NA	No	NA	NA	NA	NA
SalHit:S482017_6928	BT26	42.088	0.0036	NA	No	No	No	No	No	No	No	NA	NA	No	NA	NA	NA	NA
cDNA:C123300059_91	BT26	44.26	0.0059	NA	No	No	No	No	No	No	No	NA	NA	No	NA	NA	NA	NA
cDNA:S581445_450	BT26	45.615	0.0125	NA	No	No	No	No	No	No	No	NA	NA	No	NA	NA	NA	NA
SalarSNP:GCR_cBin5170_Ctg1	BT26	47.771	0.0011	NA	No	No	No	No	No	No	No	NA	NA	No	NA	NA	NA	NA
SalHit:C124197598_304	BT26	48.411	0.0394	NA	Yes	No	No	No	No	No	No	0.874285714	0.84367021	Yes	NA	NA	NA	NA
SalHit:S527948_252	BT27	0	0.0203	NA	No	No	No	No	No	No	No	NA	NA	No	NA	NA	NA	NA
Gdist:S232219_5301	BT27	0	-0.0053	NA	No	No	No	No	No	No	No	NA	NA	No	NA	NA	NA	NA
Gdist:S148133_1069	BT27	0.003	0.0426	NA	No	No	No	No	No	No	No	0.982691275	0.90315672	Yes	NA	NA	NA	NA
cDNA:S280750_4106	BT27	0.099	-0.0011	NA	No	No	No	No	No	No	No	NA	NA	No	NA	NA	NA	NA
SalHit:S37979_3170	BT27	1.346	0.034	NA	No	No	No	No	No	Yes (0.025)	No	0.997	0.46026971	Yes	NA	NA	NA	NA
SalHit:S146101_2536	BT27	5.43	0.0338	NA	No	No	No	No	No	Yes (0.021)	No	0.60374036	0.37652344	Yes	NA	NA	NA	NA
cDNA:S257231_2778	BT27	5.438	0.0178	NA	No	No	No	No	No	No	No	NA	NA	No	NA	NA	NA	NA
cDNA:S198876_8759	BT27	5.441	0.0065	NA	Yes	No	No	No	No	No	No	NA	NA	No	NA	NA	NA	NA
cDNA:S3392_1022	BT27	5.442	0.0178	NA	No	No	No	No	No	No	No	NA	NA	No	NA	NA	NA	NA
Gdist:S521676_4341	BT27	6.323	0.0081	NA	No	No	No	No	No	No	No	NA	NA	No	NA	NA	NA	NA
SalHit:S162316_3358	BT27	8.916	-0.0001	NA	Yes	No	No	No	No	No	No	NA	NA	No	NA	NA	NA	NA
Gdist:S325586_5759	BT27	16.072	0.0179	NA	No	No	No	No	No	Yes (0.047)	No	NA	NA	No	NA	NA	NA	NA
SalHit:S126835_16266	BT27	16.95	-0.0073	NA	No	No	No	No	No	No	No	NA	NA	No	NA	NA	NA	NA
Gdist:S130620_9198	BT27	16.95	-0.0075	NA	No	No	No	No	No	Yes (0.037)	No	NA	NA	No	NA	NA	NA	NA
Gdist:S55507_8337	BT27	18.066	0.0017	NA	No	No	No	No	No	No	No	NA	NA	No	NA	NA	NA	NA
SalHit:S726481_118	BT27	18.074	-0.0014	NA	No	No	No	No	No	No	No	NA	NA	No	NA	NA	NA	NA
cDNA:S516836_5414	BT27	18.081	0.0174	NA	No	No	No	No	No	Yes (0)	Yes (0.001)	NA	NA	No	NA	NA	NA	NA
Gdist:S7454_7921	BT27	20.57	0.0129	NA	No	No	No	No	No	No	No	NA	NA	No	NA	NA	NA	NA
Gdist:S410915_8446	BT27	21.563	-0.0062	NA	No	No	No	No	No	No	No	NA	NA	No	NA	NA	NA	NA
Gdist:S108339_5983	BT27	25.706	0.0202	NA	No	No	No	No	No	No	No	NA	NA	No	NA	NA	NA	NA
Gdist:S108339_7213	BT27	26.496	0.015	NA	No	No	No	No	No	No	No	NA	NA	No	NA	NA	NA	NA
SalarSNP:MHC_IA_41640:4182	BT27	27.229	0.0016	NA	No	No	No	No	No	No	No	NA	NA	No	NA	NA	NA	NA
SalarSNP:MHC_IA_42900:4301	BT27	27.26	0.0049	NA	No	No	No	No	No	No	No	NA	NA	No	NA	NA	NA	NA
Gdist:S430932_7683	BT27	27.291	0.0147	NA	No	No	No	No	No	No	No	0.982691275	0.95357891	Yes	NA	NA	NA	NA
Gdist:S430932_892	BT27	27.291	0.0286	NA	No	No	No	No	No	No	No	NA	NA	No	NA	NA	NA	NA
Gdist:S430932_7616	BT27	27.291	0.0104	NA	No	No	No	No	No	No	No	NA	NA	No	NA	NA	NA	NA
SalHit:S339911_3339	BT27	27.291	0.0046	NA	No	No	No	No	No	No	No	NA	NA	No	NA	NA	NA	NA
SalHit:S339911_6409	BT27	27.291	-0.0073	NA	No	No	No	No	No	No	No	NA	NA	No	NA	NA	NA	NA
Gdist:S98242_7253	BT27	28.002	0.016	NA	No	No	No	No	No	No	No	NA	NA	No	NA	NA	NA	NA
SalHit:S297381_2880	BT27	28.002	-0.0063	NA	No	No	No	No	No	No	No	NA	NA	No	NA	NA	NA	NA
cDNA:S297454_6571	BT27	30.133	-0.0069	NA	No	No	No	No	No	Yes (0.022)	No	NA	NA	No	NA	NA	NA	NA
cDNA:S297454_5449	BT27	30.134	0.0187	NA	No	No	No	No	No	Yes (0.007)	No	NA	NA	No	NA	NA	NA	NA
SalHit:S257951_2189	BT27	30.193	0.0182	NA	No	No	No	No	No	No	No	NA	NA	No	NA	NA	NA	NA
Gdist:S281987_4404	BT27	33.775	-0.0069	NA	No	No	No	No	No	No	No	NA	NA	No	NA	NA	NA	NA
Gdist:S452616_6055	BT27	35.139	0.0848	none	Yes	No	No	No	No	No	No	0	0.91198442	Yes	VAR RED	Reduced	NA	yes
Gdist:S684253_1039	BT27	35.166	0.0072	NA	No	No	No	No	No	No	No	NA	NA	No	NA	NA	NA	NA
Gdist:S215560_4477	BT27	35.166	0.001	NA	No	No	No	No	No	No	No	NA	NA	No	NA	NA	NA	NA
SalHit:S131591_3093	BT27	35.166	0.0393	NA	No	No	No	No	No	No	No	0.735576923	0.01821429	Yes	SKJ INC	NA	increased	yes
SalHit:S393517_1929	BT27	35.168	0.0045	NA	No	No	No	No	No	No	No	NA	NA	No	NA	NA	NA	NA
Gdist:S223544_3397	BT27	35.176	0.0301	NA	No	No	No	No	No	No	No	0.982691275	0.97957317	Yes	NA	NA	NA	NA
Gdist:S626031_5038	BT27	42.361	0.0037	NA	No	No	No	No	No	Yes (0.044)	No	NA	NA	No	NA	NA	NA	NA
cDNA:S196323_2857	BT27	46.465	0.0046	NA	Yes	No	No	No	No	No	No	NA	NA	No	NA	NA	NA	NA
Gdist:S149456_654	BT27	46.728	0.0219	NA	No	No	No	No	No	Yes (0.03)	No	NA	NA	No	NA	NA	NA	NA
Gdist:S235201_7057	BT27	46.99	0.0254	NA	No	No	No	No	No	No	No	NA	NA	No	NA	NA	NA	NA
cDNA:S660672_1738	BT27	47.724	0.0389	PREDIC	No	No	No	No	No	No	No	0.731157407	0	Yes	SKJ RED	NA	reduced	yes
cDNA:S164085_5376	BT27	47.729	0.009	NA	No	No	No	No	No	No	No	NA	NA	No	NA	NA	NA	NA
Gdist:S94179_6082	BT27	47.755	0.0278	NA	No	No	No	No	No	No	No	NA	NA	No	NA	NA	NA	NA
SalHit:S222106_10549	BT27	48.318	0.0241	NA	No	No	No	No	No	No	No	NA	NA	No	NA	NA	NA	NA
Gdist:S37688_6595	BT27	48.318	0.0075	NA	No	No	No	No	No	No	No	NA	NA	No	NA	NA	NA	NA
Gdist:S202603_7315	BT27	48.321	0.0361	NA	No	No	No	No	No	No	No	0.1071	0.09807692	Yes	NA	NA	NA	NA
SalHit:S234782_2105	BT27	52.515	-0.006	NA	No	No	No	No	No	No	No	NA	NA	No	NA	NA	NA	NA
SalHit:S747242_398	BT27	52.535	0.0138	NA	No	No	No	No	No	No	No	NA	NA	No	NA	NA	NA	NA
Gdist:S31249_4111	BT27	52.539	0.0005	NA	No	No	No	No	No	Yes (0.037)	No	NA	NA	No	NA	NA	NA	NA
SalarSNP:GCR_cBin44942_Ctg	BT27	52.839	0.018	NA	No	No	No	No	No	No	No	NA	NA	No	NA	NA	NA	NA
SalHit:S801501_1444	BT27	53.139	0.0507	NA	No	No	No	No	No	No	No	0.519280063	0.31739362	Yes	NA	NA	NA	NA
Gdist:S345100_1033	BT27	53.139	0.0249	NA	No	No	No	No	No	No	No	NA	NA	No	NA	NA	NA	NA
Gdist:S345100_7775	BT27	53.139	0.0183	NA	No	No	No	No	No	No	No	NA	NA	No	NA	NA	NA	NA
cDNA:S552588_975	BT27	53.139	0.0026	NA	No	No	No	No	No	No	No	NA	NA	No	NA	NA	NA	NA
Gdist:S319965_5624	BT27	53.139	-0.0084	NA	No	No	No	No	No	No	No	NA	NA	No	NA	NA	NA	NA
Gdist:S204343_605	BT27	53.139	0.0369	PREDIC	No	No	No	No	No	No	No	0.038898305	0.83624322	Yes	VAR RED	Reduced	NA	yes
cDNA:S155659_1271	BT27	53.854	-0.0048	NA	No	No	No	No	No	No	No	NA	NA	No	NA	NA	NA	NA
cDNA:S628260_5673	BT27	53.87	0.0126	NA	No	No	No	No	No	No	No	NA	NA	No	NA	NA	NA	NA
Gdist:S476075_6822	BT27	53.876	0.0174	NA	No	No	No	No	No	No	No	NA	NA	No	NA	NA	NA	NA
cDNA:S438534_1353	BT27	54.516	0.0304	NA	Yes	No	No	No	No	No	No	0.639308511	0.59462389	Yes	NA	NA	NA	NA
cDNA:S347511_3755	BT27	55.078	0.0441	NA	No	No	No	No	No	Yes (0.037)	No	0.176538462	0.1434375	Yes	NA	NA	NA	NA
SalHit:S634821_498	BT27	55.133	0.0259	NA	No	No	No	No	No	No	No	NA	NA	No	NA	NA	NA	NA
cDNA:S649928_1697	BT27	55.133	0.0156	NA	No	No	No	No	No	No	No	NA	NA	No	NA	NA	NA	NA
cDNA:S649928_1079	BT27	55.133	-0.0033	NA	No	No	No	No	No	No	No	NA	NA	No	NA	NA	NA	NA
SalarSNP:ESTNV_32423_381	BT27	55.133	-0.0048	NA	No	No	No	No	No	No	No	NA	NA	No	NA	NA	NA	NA
SalHit:S74545_5079	BT27	55.398	0.0045	NA	No	No	No	No	No	No	No	NA	NA	No	NA	NA	NA	NA
cDNA:S327027_2206	BT28	0	-0.0063	NA	No	No	No	No	No	No	No	NA	NA	No	NA	NA	NA	NA
cDNA:C124413300_569	BT28	0.143	-0.0033	NA	No	No	No	No	No	No	No	NA	NA	No	NA	NA	NA	NA
cDNA:S300070_3495	BT28	0.572	0.0103	NA	No	No	No	No	No	No	No	NA	NA	No	NA	NA	NA	NA
Gdist:S323962_5726	BT28	3.167	-0.0107	NA	No	No	No	No	No	No	No	NA	NA	No	NA	NA	NA	NA
cDNA:S584975_3561	BT28	3.95	0.042	NA	No	No	No	No	No	No	No	0.299431818	0.89481818	Yes	NA	NA	NA	NA
cDNA:S231999_1945	BT28	5.658	0.0087	NA	No	No	No	No	No	No	No	NA	NA	No	NA	NA	NA	NA
SalHit:S540769_959	BT28	6.429	-0.0038	NA	No	No	No	No	No	No	No	NA	NA	No	NA	NA	NA	NA
Gdist:S34604_7094	BT28	6.691	0.0068	NA	No	No	No	No	No	No	No	NA	NA	No	NA	NA	NA	NA
Gdist:S126650_8027	BT28	6.824	0.0885	NA	Yes	no	no	No	No	No	No	NA	NA	no	NA	NA	NA	NA
Gdist:S13053_6942	BT28	8.889	0.014	NA	No	No	No	No	No	No	No	NA	NA	No	NA	NA	NA	NA
Gene:S790412_561	BT28	12.276	0.0333	NA	No	No	No	No	No	No	No	0.519280063	0.48116071	Yes	NA	NA	NA	NA
Gdist:S88978_7878	BT28	17.089	0.0182	NA	No	No	No	No	No	No	No	NA	NA	No	NA	NA	NA	NA
Gdist:S88978_5825	BT28	17.131	-0.0023	none	No	No	No	No	No	No	No	0.0095625	0.48440154	Yes	VAR RED	Reduced	NA	yes
Gdist:S255381_1038	BT28	20.247	0.0474	none	Yes	No	No	No	No	No	No	0	0.81575481	Yes	VAR RED	Reduced	NA	yes
Gdist:S476304_2983	BT28	20.772	0.014	NA	No	No	No	No	No	No	No	NA	NA	No	NA	NA	NA	NA
Gdist:S195249_14487	BT28	27.262	0.0159	NA	No	No	No	No	No	No	No	NA	NA	No	NA	NA	NA	NA
Gdist:S273682_6131	BT28	29.182	0.0292	NA	No	No	No	No	No	No	No	NA	NA	No	NA	NA	NA	NA
cDNA:S224362_842	BT28	30.282	0.01	NA	No	No	No	No	No	No	No	NA	NA	No	NA	NA	NA	NA
Gdist:S136955_6950	BT28	30.282	0.0078	NA	No	No	No	No	No	Yes (0.004)	Yes (0.033)	NA	NA	No	NA	NA	NA	NA
Gdist:S222096_4008	BT28	30.282	0.0032	NA	No	No	No	No	No	No	No	NA	NA	No	NA	NA	NA	NA
Gdist:S222096_5341	BT28	30.282	0.0015	NA	No	No	No	No	No	Yes (0.032)	No	NA	NA	No	NA	NA	NA	NA
Gdist:S354215_5179	BT28	30.282	-0.0056	NA	No	No	No	No	No	Yes (0.022)	No	NA	NA	No	NA	NA	NA	NA
Gdist:S222096_3642	BT28	30.282	0.0235	NA	No	No	No	No	No	No	No	0	0.70376232	Yes	VAR INC	increased	NA	NA
Gdist:S371335_11038	BT28	31.099	0.0118	NA	Yes	No	No	No	No	No	No	NA	NA	No	NA	NA	NA	NA
SalHit:S227738_2874	BT28	31.21	0.0518	NA	No	No	No	No	No	No	No	0.690994565	0.95357891	Yes	NA	NA	NA	NA
cDNA:S10072_4210	BT28	33.168	0.0271	NA	No	No	No	No	No	No	No	NA	NA	No	NA	NA	NA	NA
Gdist:S146373_3747	BT28	33.404	0.0093	NA	No	No	No	No	No	Yes (0.023)	No	NA	NA	No	NA	NA	NA	NA
Gdist:S115799_3157	BT28	33.845	0.0167	NA	No	No	No	No	No	No	No	NA	NA	No	NA	NA	NA	NA
cDNA:S290599_2915	BT28	34.816	0.012	NA	No	No	No	No	No	Yes (0.014)	No	NA	NA	No	NA	NA	NA	NA
Gdist:S409313_5424	BT28	36.288	0.0208	NA	No	No	No	No	No	Yes (0.014)	No	NA	NA	No	NA	NA	NA	NA
Gdist:S54928_3450	BT28	38.753	0.0302	none	No	No	No	No	No	No	No	0	0.96812069	Yes	VAR RED	Reduced	NA	NA
Gdist:S125541_4530	BT28	39.787	0.0352	NA	No	No	No	No	No	No	No	0.532477204	0.706875	Yes	NA	NA	NA	NA
Gdist:S25145_8223	BT28	42.448	0.0121	NA	No	No	No	No	No	No	No	NA	NA	No	NA	NA	NA	NA

Gdist:S60715_1199	BT28	42.455	0.0388	NA	No	No	No	No	No	No	No	0.6196875	0.3785567	Yes	NA	NA	NA	NA
Gdist:S455666_3114	BT28	46.403	0.1149	NA	No	No	No	No	No	No	No	0.60374036	0.3065625	Yes	NA	NA	NA	NA
Gdist:S455666_7064	BT28	46.405	0.101	NA	No	No	No	No	No	No	No	0.60374036	0.3183042	Yes	NA	NA	NA	NA
Gdist:S626596_2531	BT28	46.407	-0.0056	NA	No	No	No	No	No	No	No	NA	NA	No	NA	NA	NA	NA
SalHit:S409665_1998	BT28	46.455	0.0692	NA	No	No	No	No	No	No	No	0.508712121	0.60417614	Yes	NA	NA	NA	NA
Gdist:S85530_3792	BT28	47.296	0.0574	NA	Yes	No	No	No	No	No	No	0.988505976	0.76824153	Yes	NA	NA	NA	NA
Gdist:S85530_308	BT28	47.541	0.0237	NA	No	No	No	No	No	Yes (0.007)	No	NA	NA	No	NA	NA	NA	NA
Gdist:S293156_5610	BT28	48.062	0.0079	NA	No	No	No	No	No	No	No	NA	NA	No	NA	NA	NA	NA
cDNA:S589335_1602	BT28	48.235	0.0245	NA	No	No	No	No	No	No	No	NA	NA	No	NA	NA	NA	NA
Gdist:S168408_3259	BT28	50.603	0.0175	NA	No	No	No	No	No	No	No	NA	NA	No	NA	NA	NA	NA
Gdist:S264178_335	BT28	50.606	0.03	NA	No	No	No	No	No	No	No	NA	NA	No	NA	NA	NA	NA
Gdist:S284585_2743	BT28	50.606	0.0194	NA	Yes	No	No	No	No	No	No	NA	NA	No	NA	NA	NA	NA
Gdist:S454459_7441	BT28	50.614	0.0318	NA	No	No	No	No	No	No	No	0.636282816	0.88813764	Yes	NA	NA	NA	NA
Gdist:S43880_1857	BT28	51.932	0.0281	NA	No	No	No	No	No	No	No	NA	NA	No	NA	NA	NA	NA
Gdist:S37227_7785	BT28	51.932	0.0018	NA	No	No	No	No	No	No	No	NA	NA	No	NA	NA	NA	NA
Gdist:S294330_6974	BT28	51.932	-0.0013	NA	No	No	No	No	No	No	No	NA	NA	No	NA	NA	NA	NA
Gdist:S740430_4643	BT28	52.116	0.0641	NA	No	No	No	No	No	No	No	0.534375	0.92525912	Yes	NA	NA	NA	NA
cDNA:S596508_8961	BT28	52.221	0.0274	NA	No	No	No	No	No	No	No	NA	NA	No	NA	NA	NA	NA
Gdist:S282567_3385	BT28	52.325	0.0265	NA	No	No	No	No	No	No	No	NA	NA	No	NA	NA	NA	NA
cDNA:S825915_1100	BT28	52.325	0.024	NA	No	No	No	No	No	No	No	NA	NA	No	NA	NA	NA	NA
Gdist:S436692_1348	BT28	52.325	0.0039	NA	No	No	No	No	No	No	No	NA	NA	No	NA	NA	NA	NA
Gdist:S284818_5332	BT28	52.729	0.0009	NA	No	No	No	No	No	No	No	NA	NA	No	NA	NA	NA	NA
Gdist:S294476_8206	BT28	53.26	0.0312	NA	No	No	No	No	No	No	No	0.821666667	0.44161364	Yes	NA	NA	NA	NA
Gene:S525873_108	BT28	53.26	0.0176	NA	No	No	No	No	No	No	No	NA	NA	No	NA	NA	NA	NA
Gdist:S7892_6532	BT28	53.26	0.0034	NA	No	No	No	No	No	No	No	NA	NA	No	NA	NA	NA	NA
Gdist:S6283_3963	BT28	53.322	0.0209	NA	No	No	No	No	No	No	No	NA	NA	No	NA	NA	NA	NA
Gdist:S79059_1879	BT28	53.384	0.0889	NA	No	No	No	No	No	No	No	0.0478125	0.52827797	Yes	VAR INC	increased	NA	NA
Gdist:S30004_9738	BT28	54.304	0.039	NA	No	No	No	No	No	No	No	0.982691275	0.54853698	Yes	NA	NA	NA	NA
Gdist:S126650_9899	BT28	54.307	-0.0115	NA	Yes	No	No	No	No	No	No	NA	NA	No	NA	NA	NA	NA
cDNA:S180637_703	BT28	54.988	0.0396	NA	No	No	No	No	No	No	No	0.606521739	0.81456963	Yes	NA	NA	NA	NA
Gdist:S124088_7851	BT28	55.681	-0.001	NA	No	No	No	No	No	No	No	NA	NA	No	NA	NA	NA	NA
Gdist:S383427_1527	BT28	55.729	0.0493	NA	No	No	No	No	No	No	No	0.11796729	0.3315	Yes	NA	NA	NA	NA
Gdist:S753220_6339	BT28	55.729	0.0472	NA	No	No	No	No	No	No	No	0.916490895	0.53217391	Yes	NA	NA	NA	NA
SalHit:S407342_880	BT28	55.729	0.0434	NA	No	No	No	No	No	No	No	0.237413793	0.60417614	Yes	NA	NA	NA	NA
cDNA:S194666_1674	BT28	55.729	0.029	NA	No	No	No	No	No	No	No	NA	NA	No	NA	NA	NA	NA
Gdist:S383427_2509	BT28	55.729	0.0206	NA	No	No	No	No	No	No	No	NA	NA	No	NA	NA	NA	NA
cDNA:S111702_1407	BT28	55.729	0.0164	NA	No	No	No	No	No	Yes (0.035)	No	NA	NA	No	NA	NA	NA	NA
Gdist:S116118_10589	BT28	55.729	0.0128	NA	No	No	No	No	No	Yes (0.004)	Yes (0.035)	NA	NA	No	NA	NA	NA	NA
cDNA:S407073_2825	BT28	55.729	0.0078	NA	No	No	No	No	No	No	No	NA	NA	No	NA	NA	NA	NA
cDNA:S297048_3936	BT28	55.729	-0.0008	NA	No	No	No	No	No	No	No	NA	NA	No	NA	NA	NA	NA
cDNA:S828038_587	BT28	55.734	-0.0108	NA	No	No	No	No	No	No	No	0.838743243	0.73333893	Yes	NA	NA	NA	NA
cDNA:S720470_272	BT28	55.735	-0.0078	NA	No	No	No	No	No	No	No	NA	NA	No	NA	NA	NA	NA
cDNA:S566778_923	BT29	0	-0.0023	NA	No	No	No	No	No	No	No	NA	NA	No	NA	NA	NA	NA
cDNA:S48128_2938	BT29	0.922	0.015	NA	No	No	No	No	No	No	No	NA	NA	No	NA	NA	NA	NA
Gdist:S352270_3238	BT29	1.888	0.0266	NA	Yes	No	No	No	No	No	No	NA	NA	No	NA	NA	NA	NA
cDNA:S243791_4937	BT29	2.853	-0.0036	NA	No	No	No	No	No	No	No	NA	NA	No	NA	NA	NA	NA
Gdist:S303669_4492	BT29	2.937	-0.0073	NA	No	No	No	No	No	No	No	NA	NA	No	NA	NA	NA	NA
Gdist:S47297_805	BT29	3.415	0.0287	NA	No	No	No	No	No	Yes (0.048)	No	NA	NA	No	NA	NA	NA	NA
cDNA:S20419_2654	BT29	3.652	0.0043	NA	No	No	No	No	No	No	No	NA	NA	No	NA	NA	NA	NA
Gdist:S196825_5115	BT29	3.956	0.0401	NA	No	No	No	No	No	No	No	0.63780285	0.72657534	Yes	NA	NA	NA	NA
Gdist:S175989_2428	BT29	5.993	0.0098	NA	No	No	No	No	No	No	No	NA	NA	No	NA	NA	NA	NA
Gene:S413536_297	BT29	5.999	0.0486	NA	No	No	No	No	No	No	No	0.476118881	0.97882632	Yes	NA	NA	NA	NA
SalarSNP:GCR_cBin17378_Ctg	BT29	7.124	0.0006	NA	No	No	No	No	No	No	No	NA	NA	No	NA	NA	NA	NA
cDNA:S105897_2400	BT29	18.63	0.0415	NA	No	No	No	No	No	Yes (0.007)	Yes (0.049)	0.565105932	0.62684636	Yes	NA	NA	NA	NA
Gdist:S229342_7969	BT29	21.672	0.0084	NA	No	No	No	No	No	No	No	NA	NA	No	NA	NA	NA	NA
LD:S269684_1562	BT29	23.725	0.0321	NA	No	No	No	No	No	No	No	0.098582474	0.33707813	Yes	NA	NA	NA	NA
SalHit:S344034_1491	BT29	24.202	0.0029	NA	No	No	No	No	No	No	No	NA	NA	No	NA	NA	NA	NA
Gdist:S70799_3913	BT29	25.331	0.0157	NA	No	No	No	No	No	No	No	NA	NA	No	NA	NA	NA	NA
Gdist:S203333_2297	BT29	27.588	0.0292	NA	No	No	No	No	No	No	No	0.614833333	0.800955	Yes	NA	NA	NA	NA
cDNA:S315299_565	BT29	28.717	0.0178	NA	No	No	No	No	No	No	No	NA	NA	No	NA	NA	NA	NA
Gdist:S237536_4418	BT29	31.781	0.0123	NA	No	No	No	No	No	Yes (0.044)	No	NA	NA	No	NA	NA	NA	NA
Gdist:S625346_1946	BT29	31.845	0.0336	NA	No	No	No	No	No	No	No	0.519019293	0.88730408	Yes	NA	NA	NA	NA
Gdist:S257634_2635	BT29	31.845	0.028	NA	No	No	No	No	No	No	No	NA	NA	No	NA	NA	NA	NA
Gdist:S612164_4378	BT29	31.845	0.0117	NA	No	No	No	No	No	Yes (0.02)	No	NA	NA	No	NA	NA	NA	NA
Gdist:S490411_1398	BT29	31.845	-0.0036	NA	No	No	No	No	No	No	No	NA	NA	No	NA	NA	NA	NA
Gdist:S123212_5018	BT29	31.845	-0.0106	NA	No	No	No	No	No	No	No	NA	NA	No	NA	NA	NA	NA
Gdist:S94536_773	BT29	31.847	0.0001	NA	No	No	No	No	No	No	No	NA	NA	No	NA	NA	NA	NA
SalHit:S525370_259	BT29	34.025	-0.006	NA	No	No	No	No	No	No	No	NA	NA	No	NA	NA	NA	NA
cDNA:S113427_4484	BT29	34.04	0.0535	NA	Yes	No	No	No	No	No	No	0.603366142	0.4818314	Yes	NA	NA	NA	NA
Gdist:S103230_2073	BT29	34.04	0.0209	NA	No	No	No	No	No	No	No	NA	NA	No	NA	NA	NA	NA
cDNA:S113427_14338	BT29	34.04	0.0126	NA	No	No	No	No	No	No	No	NA	NA	No	NA	NA	NA	NA
cDNA:S147850_1094	BT29	35.215	0.0316	NA	No	No	No	No	No	No	No	0.593534483	0.60473464	Yes	NA	NA	NA	NA
Gdist:S335579_9253	BT29	38.202	0.0277	NA	No	No	No	No	No	No	No	NA	NA	No	NA	NA	NA	NA
Gdist:S364170_3356	BT29	38.427	-0.0027	NA	No	No	No	No	No	No	No	NA	NA	No	NA	NA	NA	NA
Gdist:S216255_8244	BT29	38.634	0.012	NA	No	No	No	No	No	No	No	NA	NA	No	NA	NA	NA	NA
Gdist:S35446_706	BT29	43.439	0.0253	NA	No	No	No	No	No	No	No	NA	NA	No	NA	NA	NA	NA
cDNA:S554410_3524	BT29	45.767	0.0112	NA	No	No	No	No	No	No	No	NA	NA	No	NA	NA	NA	NA
SalHit:S217108_4004	BT29	46.162	0.0077	NA	No	No	No	No	No	No	No	NA	NA	No	NA	NA	NA	NA
SalHit:S75583_3748	BT29	46.163	0.0185	NA	No	No	No	No	No	Yes (0.046)	No	NA	NA	No	NA	NA	NA	NA
Gdist:S383366_10329	BT29	46.163	-0.0091	NA	No	No	No	No	No	No	No	NA	NA	No	NA	NA	NA	NA
Gdist:S6117_4696	BT29	46.164	0.0479	none	No	No	No	No	No	No	No	0.0095625	0.88194268	Yes	VAR RED	Reduced	NA	NA
Gdist:S409381_1574	BT29	46.166	0.0315	NA	No	No	No	No	No	No	No	0.893131188	0.53217391	Yes	NA	NA	NA	NA
Gdist:S10940_2042	BT29	51.183	0.0125	NA	No	No	No	No	No	Yes (0.035)	No	NA	NA	No	NA	NA	NA	NA
Gdist:S635245_7636	BT29	54.082	0.0265	NA	No	No	No	No	No	No	No	NA	NA	No	NA	NA	NA	NA
Gdist:S530174_867	BT29	54.543	0.0119	NA	No	No	No	No	No	No	No	NA	NA	No	NA	NA	NA	NA
Gdist:S190898_3366	BT29	55.009	-0.0012	NA	No	No	No	No	No	No	No	NA	NA	No	NA	NA	NA	NA
cDNA:S366861_1180	BT29	55.045	0.0145	NA	No	No	No	No	No	No	No	NA	NA	No	NA	NA	NA	NA
Gdist:S175766_1990	BT29	55.08	0.0544	NA	No	No	No	No	No	No	No	0.612582487	0.1434375	Yes	NA	NA	NA	NA
Gdist:S75237_2255	BT29	56.18	0.0427	NA	No	No	No	No	No	No	No	0.907678571	0.34793675	Yes	NA	NA	NA	NA
Gdist:S75237_6644	BT29	57.219	0.0173	NA	No	No	No	No	No	No	No	NA	NA	No	NA	NA	NA	NA
Gdist:S273578_4932	BT29	59.402	0.0155	NA	No	No	No	No	No	No	No	NA	NA	No	NA	NA	NA	NA
Gdist:S94519_2437	BT29	59.498	0.0236	NA	No	No	No	No	No	No	No	NA	NA	No	NA	NA	NA	NA
Gdist:S52255_5395	BT29	65.078	0.0109	NA	No	No	No	No	No	No	No	NA	NA	No	NA	NA	NA	NA
Gdist:S19804_820	BT29	65.11	0.0276	NA	No	No	No	No	No	No	No	NA	NA	No	NA	NA	NA	NA
Gdist:S4312_3227	BT29	65.118	-0.0004	NA	No	No	No	No	No	No	No	NA	NA	No	NA	NA	NA	NA
Gdist:S4312_7371	BT29	65.126	0.0137	NA	No	No	No	No	No	No	No	NA	NA	No	NA	NA	NA	NA
Gdist:S115968_7253	BT29	67.141	0.0153	NA	No	No	No	No	No	No	No	NA	NA	No	NA	NA	NA	NA
Gdist:S77986_1331	BT29	68.478	0.0327	NA	No	No	No	No	No	No	No	0.405728745	0.7246445	Yes	NA	NA	NA	NA
SalarSNP:ESTNV_35308_389	BT29	68.478	0.0058	NA	No	No	No	No	No	No	No	NA	NA	No	NA	NA	NA	NA
Gene:S276486_3960	BT29	69.839	0.034	NA	No	No	No	No	No	No	No	0.896875	0.69562972	Yes	NA	NA	NA	NA
cDNA:C124725291_143	BT29	69.839	0.0231	NA	No	No	No	No	No	No	No	NA	NA	No	NA	NA	NA	NA
Gene:S171022_6436	BT29	69.877	0.0245	NA	No	No	No	No	No	No	No	NA	NA	No	NA	NA	NA	NA
SalHit:S494492_5892	BT29	69.877	0.0117	NA	No	No	No	No	No	Yes (0.001)	Yes (0.017)	NA	NA	No	NA	NA	NA	NA
Gdist:S263519_1867	BT29	69.877	0.0084	NA	Yes	No	No	No	No	No	No	NA	NA	No	NA	NA	NA	NA
SalHit:S126925_1562	BT29	69.877	0.0059	NA	No	No	No	No	No	No	No	NA	NA	No	NA	NA	NA	NA
LD:S240758_5242	BT29	69.878	0.0418	NA	Yes	No	No	No	No	No	No	0.365723684	0.82025797	Yes	NA	NA	NA	NA
Gdist:S87415_11622	BT29	72.015	0.0129	NA	No	No	No	No	No	No	No	NA	NA	No	NA	NA	NA	NA
cDNA:S422982_9383	BT29	72.097	0.0105	NA	No	No	No	No	No	No	No	0.446736641	0.37646053	Yes	NA	NA	NA	NA
cDNA:S184986_7445	BT29	72.097	0.0036	NA	No	No	No	No	No	No	No	NA	NA	No	NA	NA	NA	NA
Gdist:S6438_4812	BT29	73.403	0.0338	NA	No	No	No	No	No	Yes (0.039)	No	0.59272118	0.49897556	Yes	NA	NA	NA	NA
Gdist:S248244_4193	BT29	73.882	0.0043	NA	No	No	No	No	No	No	No	NA	NA	No	NA	NA	NA	NA
Gdist:S214687_707	BT29	74.85	0.0377	NA	No	No	No	No	No	No	No	0.558362069	0.81392442	Yes	NA	NA	NA	NA
cDNA:S201621_1382	BT29	74.85	0.034	NA	No	No	No	No	No	Yes (0.021)	No	0.664111239	0.80606362	Yes	NA	NA	NA	NA
Gdist:S307616_5920	BT29	75.235	0.0163	NA	No	No	No	No	No	No	No	NA	NA	No	NA	NA	NA	NA
SalHit:S768218_2293	BT29	75.235	0.0083	NA	No	No	No	No	No	Yes (0.013)	No	NA	NA	No	NA	NA	NA	NA
cDNA:S388728_2949	BT29	76.132	0.0468	NA	No	No	No	No	No	No	No	0.75075	0.72169811	Yes	NA	NA	NA	NA
Gdist:S254257_11272	BT29	76.132	0.0294	NA	No	No	No	No	No	No	No	NA	NA	No	NA	NA	NA	NA
cDNA:S525559_3367	BT29	76.132	0.0067	NA	No	No	No	No	No	No	No	NA	NA	No	NA	NA	NA	NA
cDNA:S525559_3568	BT29	76.132	0.0046	NA	No	No	No	No	No	No	No	NA	NA	No	NA	NA	NA	NA
Gdist:S124875_7732	BT29	76.133	0.0173	NA	No	No	No	No	No	No	No	NA	NA	No	NA	NA	NA	NA
cDNA:S73751_1345	BT29	76.185	0.0247	NA	No	No	No	No	No	No	No	NA	NA	No	NA	NA	NA	NA
Gdist:S124875_7407	BT29	76.185	0.0071	NA	No	No	No	No	No	No	No	NA	NA	No	NA	NA	NA	NA
cDNA:S559995_1169	BT29	77.803	0.024	NA	No	No	No	No	No	No	No	NA	NA	No	NA	NA	NA	NA
cDNA:S548371_2277	BT29	77.803	0.0098	NA	No	No	No	No	No	No	No	NA	NA	No	NA	NA	NA	NA
cDNA:S261458_2796	BT29	77.864	-0.001	NA	No	No	No	No	No	No	No	NA	NA	No	NA	NA	NA	NA
cDNA:S734982_525	BT29	77.919	0.0339	NA	No	No	No	No	No	No	No	0.927462742	0.74151316	Yes	NA	NA	NA	NA
cDNA:S806252_551	BT29	79.879	0.0267	NA	Yes	No	No	No	No	Yes (0.037)	No	NA	NA	No	NA	NA	NA	NA

cDNA:S806252_634	BT29	80.239	0.0449	NA	No	No	No	No	No	No	No	0.460416667	0.62684636	Yes	NA	NA	NA	NA
cDNA:S416873_399	BT30	0	-0.0055	NA	No	No	No	No	No	Yes (0.037)	No	NA	NA	No	NA	NA	NA	NA
SalHit:S240648_3170	BT30	0.005	-0.0065	NA	No	No	No	No	No	Yes (0.003)	Yes (0.027)	NA	NA	No	NA	NA	NA	NA
Gdist:S172697_6405	BT30	3.11	0.0141	NA	No	No	No	No	No	No	No	NA	NA	No	NA	NA	NA	NA
Gdist:S172697_6486	BT30	3.177	0.045	NA	No	No	No	No	No	No	No	0.63780285	0.32140625	Yes	NA	NA	NA	NA
SalHit:S235382_924	BT30	9.122	0.0092	NA	No	No	No	No	No	No	No	NA	NA	No	NA	NA	NA	NA
cDNA:S332936_3224	BT30	12.417	0.0208	NA	No	No	No	No	No	No	No	NA	NA	No	NA	NA	NA	NA
cDNA:S706325_1228	BT30	13.095	0.0351	NA	No	No	No	No	No	No	No	0.680764045	0.61532609	Yes	NA	NA	NA	NA
Gdist:S166005_2655	BT30	13.802	-0.0056	NA	No	No	No	No	No	No	No	NA	NA	No	NA	NA	NA	NA
Gdist:S119433_5515	BT30	16.025	-0.013	NA	No	No	No	No	No	No	No	NA	NA	No	NA	NA	NA	NA
Gdist:S119433_5963	BT30	16.347	-0.0029	NA	No	No	No	No	No	No	No	NA	NA	No	NA	NA	NA	NA
cDNA:S27306_4435	BT30	18.296	0.0602	NA	No	No	No	No	No	No	No	0.233502907	0.1275	Yes	NA	NA	NA	NA
Gdist:S35797_2655	BT30	18.296	0.0436	NA	Yes	No	No	No	No	No	No	0.514568404	0.1275	Yes	NA	NA	NA	NA
Gdist:S82769_8758	BT30	19.156	0.0466	NA	No	No	No	No	No	No	No	0.683456044	0.46026971	Yes	NA	NA	NA	NA
Gdist:S97611_18247	BT30	19.156	0.0242	NA	No	No	No	No	No	No	No	NA	NA	No	NA	NA	NA	NA
cDNA:S249113_11946	BT30	19.825	0.0146	NA	No	No	No	No	No	No	No	NA	NA	No	NA	NA	NA	NA
SalHit:S92841_633	BT30	22.291	0.0078	NA	No	No	No	No	No	No	No	NA	NA	No	NA	NA	NA	NA
Gdist:S403990_5235	BT30	24.627	0.0361	NA	No	No	No	No	No	No	No	0.532767857	0.89481818	Yes	NA	NA	NA	NA
Gdist:S158589_6904	BT30	24.627	0.0231	NA	No	No	No	No	No	No	No	NA	NA	No	NA	NA	NA	NA
cDNA:S186934_1562	BT30	24.627	-0.0043	NA	No	No	No	No	No	No	No	NA	NA	No	NA	NA	NA	NA
Gdist:S403990_7756	BT30	24.627	-0.0072	NA	No	No	No	No	No	No	No	NA	NA	No	NA	NA	NA	NA
Gdist:S105837_10378	BT30	25.174	0.0263	NA	No	No	No	No	No	No	No	NA	NA	No	NA	NA	NA	NA
Gdist:S387822_1001	BT30	25.761	0.001	NA	No	No	No	No	No	No	No	NA	NA	No	NA	NA	NA	NA
cDNA:S150416_2053	BT30	26.885	0.043	NA	No	No	No	No	No	No	No	0.982691275	0.87768412	Yes	NA	NA	NA	NA
Gdist:S245913_12990	BT30	27.855	0.0149	NA	No	No	No	No	No	No	No	NA	NA	No	NA	NA	NA	NA
Gdist:S245913_12179	BT30	28.825	0.012	NA	No	No	No	No	No	No	No	NA	NA	No	NA	NA	NA	NA
SalHit:S37673_4072	BT30	29.322	0.0729	NA	No	No	No	No	No	No	No	0.55323913	0.0675	Yes	NA	NA	NA	NA
Gdist:S238600_8194	BT30	29.322	0.0425	NA	Yes	No	No	No	No	No	No	0.18	0.12170455	Yes	NA	NA	NA	NA
Gdist:S361328_4903	BT30	29.322	0.0002	NA	No	No	No	No	No	No	No	NA	NA	No	NA	NA	NA	NA
Gdist:S238600_11607	BT30	29.517	-0.0017	NA	Yes	No	No	No	No	No	No	NA	NA	No	NA	NA	NA	NA
Gdist:S511634_5986	BT30	29.727	0.1184	NA	No	No	No	No	No	No	No	0.138783186	0.36960674	Yes	NA	NA	NA	NA
Gdist:S123104_1259	BT30	29.727	0.0339	NA	No	No	No	No	No	No	No	0.870811856	0.99611478	Yes	NA	NA	NA	NA
Gdist:S395349_5906	BT30	29.727	0.0079	NA	No	No	No	No	No	No	No	NA	NA	No	NA	NA	NA	NA
Gdist:S19455_7330	BT30	30.414	0.0077	NA	Yes	No	No	No	No	No	No	NA	NA	No	NA	NA	NA	NA
cDNA:S839443_3282	BT30	31.089	0.0056	NA	No	No	No	No	No	No	No	NA	NA	No	NA	NA	NA	NA
cDNA:S443429_844	BT30	32.102	-0.0126	NA	No	No	No	No	No	No	No	NA	NA	No	NA	NA	NA	NA
SalHit:S76032_10732	BT30	32.158	0.0173	NA	No	No	No	No	No	No	No	NA	NA	No	NA	NA	NA	NA
Gdist:S186751_2975	BT30	32.218	0.013	NA	No	No	No	No	No	No	No	NA	NA	No	NA	NA	NA	NA
Gdist:S298799_5433	BT30	32.282	0.0641	NA	Yes	No	No	No	No	Yes (0.022) (*als	No	0.893131188	0.53851974	Yes	NA	NA	NA	NA
Gdist:S304497_6832	BT30	32.282	0.044	NA	No	No	No	No	No	No	No	0.405728745	0.46026971	Yes	NA	NA	NA	NA
Gdist:S304497_9267	BT30	32.282	0.0124	NA	No	No	No	No	No	No	No	NA	NA	No	NA	NA	NA	NA
SalHit:S653524_6455	BT30	33.545	0.0182	NA	No	No	No	No	No	No	No	NA	NA	No	NA	NA	NA	NA
Gdist:S56983_2235	BT30	33.549	0.0083	NA	No	No	No	No	No	No	No	NA	NA	No	NA	NA	NA	NA
Gdist:S91607_7236	BT30	33.999	0.0081	NA	Yes	No	No	No	No	No	No	NA	NA	No	NA	NA	NA	NA
cDNA:S665865_592	BT30	34.157	0.0157	NA	No	No	No	No	No	No	No	NA	NA	No	NA	NA	NA	NA
Gdist:S637640_3321	BT30	34.157	0.0111	NA	No	No	No	No	No	No	No	NA	NA	No	NA	NA	NA	NA
Gdist:S86936_7429	BT30	34.168	0.0221	NA	No	No	No	No	No	No	No	NA	NA	No	NA	NA	NA	NA
Gdist:S37758_7578	BT30	35.14	0.02	NA	No	No	No	No	No	No	No	NA	NA	No	NA	NA	NA	NA
Gdist:S476440_1325	BT30	35.335	0.0695	NA	No	No	No	No	No	No	No	0	0.92940304	Yes	VAR INC	increased	NA	NA
cDNA:S41242_10981	BT30	38.089	0.026	NA	No	No	No	No	No	No	No	NA	NA	No	NA	NA	NA	NA
SalHit:S490385_4491	BT30	38.104	0.0424	NA	No	No	No	No	No	No	No	0.541781525	0.92940304	Yes	NA	NA	NA	NA
Gdist:S470738_2345	BT30	38.104	0.0068	NA	No	No	No	No	No	No	No	NA	NA	No	NA	NA	NA	NA
cDNA:S323771_3746	BT30	38.242	0.0118	NA	Yes	No	No	No	No	No	No	NA	NA	No	NA	NA	NA	NA
cDNA:S323771_6228	BT30	38.302	0.0082	NA	No	No	No	No	No	No	No	NA	NA	No	NA	NA	NA	NA
cDNA:S150131_2338	BT30	38.342	0.0113	NA	Yes	No	No	No	No	No	No	NA	NA	No	NA	NA	NA	NA
SalHit:S341846_8220	BT30	38.365	-0.0002	NA	No	no	No	No	No	No	No	NA	NA	No	NA	NA	NA	NA
SalHit:S17423_6878	BT30	38.387	0.1224	NA	Yes	no	no	No	No	No	No	NA	NA	no	NA	NA	NA	NA
Gdist:S311040_2596	BT30	38.469	0.0011	NA	No	No	No	No	No	No	No	NA	NA	No	NA	NA	NA	NA
cDNA:S402468_6204	BT30	38.505	-0.0001	NA	No	No	No	No	No	No	No	NA	NA	No	NA	NA	NA	NA
LD:S187433_714	BT30	38.89	0.0076	NA	No	No	No	No	No	No	No	NA	NA	No	NA	NA	NA	NA
Gdist:S176592_1387	BT30	38.89	0.005	NA	No	No	No	No	No	No	No	NA	NA	No	NA	NA	NA	NA
Gdist:S162384_609	BT30	38.89	0.0316	NA	No	No	No	No	No	Yes (0.014)	No	0.870811856	0	Yes	SKJ INC	NA	increased -	NA
cDNA:S38574_1326	BT30	40.042	0.0111	NA	No	No	No	No	No	No	No	NA	NA	No	NA	NA	NA	NA
Gene:S799671_712	BT30	40.611	0.034	NA	No	No	No	No	No	No	No	0.530792308	0.88165329	Yes	NA	NA	NA	NA
cDNA:S208_4966	BT30	41.184	0.042	NA	No	No	No	No	No	No	No	0.906182927	0.4067561	Yes	NA	NA	NA	NA
Gdist:S642317_3477	BT30	41.184	-0.0061	NA	No	No	No	No	No	No	No	NA	NA	No	NA	NA	NA	NA
Gdist:S20172_3792	BT30	41.184	-0.0093	NA	No	No	No	No	No	No	No	NA	NA	No	NA	NA	NA	NA
Gdist:S642317_1800	BT30	41.368	-0.0018	NA	No	No	No	No	No	No	No	NA	NA	No	NA	NA	NA	NA
SalHit:S510473_1387	BT30	41.561	0.0595	NA	No	No	No	No	No	No	No	0.838743243	0.21798387	Yes	NA	NA	NA	NA
cDNA:S87911_5985	BT30	41.561	0.0055	NA	No	No	No	No	No	No	No	NA	NA	No	NA	NA	NA	NA
Gdist:S199319_5001	BT30	41.561	0.0032	NA	Yes	No	No	No	No	No	No	NA	NA	No	NA	NA	NA	NA
cDNA:S87911_1237	BT30	41.885	0.0247	NA	No	No	No	No	No	Yes (0.021)	No	NA	NA	No	NA	NA	NA	NA
Gdist:S87797_6901	BT30	42.319	0.0046	NA	No	No	No	No	No	No	No	NA	NA	No	NA	NA	NA	NA
SalHit:S35637_2122	BT30	42.32	0.0331	NA	No	No	No	No	No	No	No	0.05961039	0.33707813	Yes	NA	NA	NA	NA
Gdist:S67618_2426	BT30	42.32	0.0323	NA	No	No	No	No	No	No	No	0.2295	0.93956009	Yes	NA	NA	NA	NA
SalHit:S111443_3771	BT30	42.32	0.0108	NA	No	No	No	No	No	No	No	NA	NA	No	NA	NA	NA	NA
SalHit:S1132_954	BT30	42.32	0.0064	NA	No	No	No	No	No	No	No	NA	NA	No	NA	NA	NA	NA
LD:S41726_11296	BT30	42.32	-0.001	NA	No	No	No	No	No	No	No	NA	NA	No	NA	NA	NA	NA
Gdist:S440775_7145	BT30	42.32	-0.0135	NA	No	No	No	No	No	No	No	NA	NA	No	NA	NA	NA	NA
cDNA:S114445_7992	BT30	42.32	0.0947	NA	Yes	no	no	No	No	No	No	NA	NA	no	NA	NA	NA	NA
Gdist:S346563_7497	BT30	42.622	0.0769	PREDIC'	No	No	No	No	No	No	No	0	0.51910714	Yes	VAR RED	Reduced	NA	NA
Gdist:S227261_6624	BT30	42.868	0.0049	NA	Yes	No	No	No	No	No	No	NA	NA	No	NA	NA	NA	NA
cDNA:S675938_3404	BT30	42.868	0.0504	PREDIC'	No	No	No	No	No	No	No	0.204	0	Yes	SKJ RED	NA	reduced	yes
Gdist:S346563_3503	BT30	42.868	0.0475	PREDIC'	No	No	No	No	No	No	No	0.0095625	0.17	Yes	VAR RED	Reduced	NA	yes
Gdist:S125_4975	BT30	43.223	0.0088	NA	No	No	No	No	No	No	No	NA	NA	No	NA	NA	NA	NA
cDNA:S638107_1152	BT30	43.563	0.0185	NA	No	No	No	No	No	No	No	NA	NA	No	NA	NA	NA	NA
cDNA:S130317_7021	BT30	43.563	0.0181	NA	No	No	No	No	No	No	No	NA	NA	No	NA	NA	NA	NA
cDNA:S785610_1712	BT30	43.563	0.014	NA	No	No	No	No	No	No	No	NA	NA	No	NA	NA	NA	NA
cDNA:S638107_1835	BT30	43.563	-0.0006	NA	No	No	No	No	No	No	No	NA	NA	No	NA	NA	NA	NA
cDNA:S411373_345	BT30	44.099	0.0028	NA	No	No	No	No	No	No	No	NA	NA	No	NA	NA	NA	NA
SalHit:S171769_2652	BT30	44.635	-0.0007	NA	No	No	No	No	No	No	No	NA	NA	No	NA	NA	NA	NA
cDNA:S744004_654	BT30	45.157	0.0163	NA	No	No	No	No	No	No	No	NA	NA	No	NA	NA	NA	NA
cDNA:S509562_6631	BT31	0	0.0321	NA	No	No	No	No	No	No	No	0.340204327	0.89499248	Yes	NA	NA	NA	NA
Gdist:S27593_2338	BT31	0.018	0.0192	NA	No	No	No	No	No	No	No	NA	NA	No	NA	NA	NA	NA
cDNA:S311598_624	BT31	0.067	-0.0007	NA	No	No	No	No	No	No	No	NA	NA	No	NA	NA	NA	NA
Gdist:S349354_1666	BT31	3.334	0.0078	NA	No	No	No	No	No	No	No	NA	NA	No	NA	NA	NA	NA
Gdist:S38546_8225	BT31	6.601	-0.0063	NA	No	No	No	No	No	No	No	NA	NA	No	NA	NA	NA	NA
Gdist:S38546_7603	BT31	6.605	0.0146	NA	No	No	No	No	No	No	No	NA	NA	No	NA	NA	NA	NA
Gdist:S215791_11521	BT31	6.61	0.0148	NA	No	No	No	No	No	No	No	NA	NA	No	NA	NA	NA	NA
cDNA:S491520_1295	BT31	6.614	-0.0003	NA	No	No	No	No	No	No	No	NA	NA	No	NA	NA	NA	NA
cDNA:S491520_853	BT31	6.618	-0.0002	NA	No	No	No	No	No	No	No	NA	NA	No	NA	NA	NA	NA
cDNA:S424361_552	BT31	6.623	0.0065	NA	No	No	No	No	No	No	No	NA	NA	No	NA	NA	NA	NA
Gdist:S368930_6372	BT31	6.652	0.0088	NA	Yes	No	No	No	No	No	No	NA	NA	No	NA	NA	NA	NA
cDNA:S118429_1535	BT31	6.682	0.018	NA	No	No	No	No	No	No	No	NA	NA	No	NA	NA	NA	NA
cDNA:S118429_3382	BT31	6.711	0.0255	NA	No	No	No	No	No	No	No	0.781630435	0.800955	Yes	NA	NA	NA	NA
cDNA:S687148_2057	BT31	8.164	0.0088	NA	No	No	No	No	No	No	No	NA	NA	No	NA	NA	NA	NA
cDNA:S90292_4948	BT31	9.405	0.0108	NA	No	No	No	No	No	No	No	NA	NA	No	NA	NA	NA	NA
Gdist:S681517_7040	BT31	14.841	0.066	NA	No	No	No	No	No	Yes (0.046)	No	0.580451482	0.9985	Yes	NA	NA	NA	NA
Gdist:S51962_10556	BT31	16.897	-0.015	NA	No	No	No	No	No	No	No	NA	NA	No	NA	NA	NA	NA
Gdist:S53668_3653	BT31	19.812	-0.0047	NA	Yes	No	No	No	No	No	No	NA	NA	No	NA	NA	NA	NA
Gdist:S128191_9860	BT31	21.837	0.0416	NA	No	No	No	No	No	No	No	0.705336538	0.56791921	Yes	NA	NA	NA	NA
Gdist:S284708_3856	BT31	21.837	0.031	NA	No	No	No	No	No	No	No	0.928928571	0.46026971	Yes	NA	NA	NA	NA
Gdist:S3295_6437	BT31	21.837	0.0269	NA	No	No	No	No	No	No	No	NA	NA	No	NA	NA	NA	NA
Gdist:S98358_10302	BT31	21.837	0.0231	NA	No	No	No	No	No	No	No	NA	NA	No	NA	NA	NA	NA
Gdist:S64325_2625	BT31	21.837	0.016	NA	No	No	No	No	No	No	No	NA	NA	No	NA	NA	NA	NA
cDNA:C124495874_565	BT31	21.837	0.0114	NA	No	No	No	No	No	No	No	NA	NA	No	NA	NA	NA	NA
Gdist:S64325_5158	BT31	21.837	0.009	NA	No	No	No	No	No	No	No	NA	NA	No	NA	NA	NA	NA
Gdist:S128191_5714	BT31	21.837	0.007	NA	No	No	No	No	No	No	No	NA	NA	No	NA	NA	NA	NA
Gdist:S13963_8153	BT31	21.837	0.0041	NA	No	No	No	No	No	No	No	NA	NA	No	NA	NA	NA	NA
LD:S489312_14508	BT31	21.837	0.0036	NA	No	No	No	No	No	No	No	NA	NA	No	NA	NA	NA	NA
Gdist:S401824_4858	BT31	21.837	0.0007	NA	No	No	No	No	No	No	No	NA	NA	No	NA	NA	NA	NA
Gdist:S401824_4448	BT31	21.837	-0.0036	NA	No	No	No	No	No	No	No	NA	NA	No	NA	NA	NA	NA
LD:S489312_6068	BT31	21.837	-0.0089	NA	No	No	No	No	No	No	No	NA	NA	No	NA	NA	NA	NA
cDNA:S480496_654	BT31	21.837	-0.0117	NA	No	No	No	No	No	No	No	NA	NA	No	NA	NA	NA	NA
Gdist:S78525_622	BT31	30.182	-0.0036	NA	Yes	No	No	No	No	No	No	NA	NA	No	NA	NA	NA	NA
Gdist:S115607_10942	BT31	30.253	0.006	NA	No	No	No	No	No	Yes (0.007)	No	NA	NA	No	NA	NA	NA	NA
cDNA:S445299_1229	BT31	31.932	0.0252	NA	No	No	No	No	No	No	No	NA	NA	No	NA	NA	NA	NA

Gdist:S247723_4246	BT31	32.067	-0.0077	NA	No	No	No	No	No	No	No	NA	NA	No	NA	NA	NA	NA
Gdist:S374589_6614	BT31	36.165	-0.0086	NA	No	No	No	No	No	No	No	NA	NA	No	NA	NA	NA	NA
Gdist:S38554_6627	BT31	36.206	0.0088	NA	No	No	No	No	No	No	No	NA	NA	No	NA	NA	NA	NA
Gdist:S562814_5473	BT31	36.244	0.0672	NA	Yes	No	No	No	No	No	No	0.946271739	0.61532609	Yes	NA	NA	NA	NA
Gdist:S4510_3280	BT31	36.244	-0.0084	NA	Yes	No	No	No	No	No	No	NA	NA	No	NA	NA	NA	NA
Gdist:S8452_3116	BT31	37.039	0.0205	NA	No	No	No	No	No	No	No	NA	NA	No	NA	NA	NA	NA
Gdist:S9906_2101	BT31	37.039	0.0084	NA	No	No	No	No	No	No	No	NA	NA	No	NA	NA	NA	NA
LD:S110750_883	BT31	37.144	0.0134	NA	No	No	No	No	No	No	No	NA	NA	No	NA	NA	NA	NA
cDNA:S792758_2028	BT31	37.913	0.0677	NA	No	No	No	No	No	No	No	0.870355263	0.59995627	Yes	NA	NA	NA	NA
Gdist:S603069_6325	BT31	37.916	0.0196	NA	No	No	No	Yes (0.038)	No	No	No	NA	NA	No	NA	NA	NA	NA
cDNA:S148838_2039	BT31	37.916	-0.0034	NA	No	No	No	No	No	No	No	NA	NA	No	NA	NA	NA	NA
Gdist:S60199_2227	BT31	39.25	0.0473	NA	No	No	No	No	No	No	No	0.510426421	0.89481818	Yes	NA	NA	NA	NA
Gdist:S223897_9134	BT31	40.493	-0.0019	NA	No	No	No	No	No	No	No	NA	NA	No	NA	NA	NA	NA
Gdist:S223897_1103	BT31	41.737	0.0033	NA	No	No	No	No	No	No	No	NA	NA	No	NA	NA	NA	NA
Gdist:S321265_10516	BT31	43.231	0.0055	NA	No	No	No	No	No	No	No	NA	NA	No	NA	NA	NA	NA
Gdist:S321265_16863	BT31	43.439	0.0409	NA	No	No	No	No	No	Yes (0.023)	No	0.365723684	0.6958849	Yes	NA	NA	NA	NA
cDNA:S219140_556	BT31	43.664	0.0311	NA	No	No	No	No	No	No	No	0.468256228	0.88381141	Yes	NA	NA	NA	NA
Gdist:S174880_7332	BT31	43.665	-0.0157	NA	No	No	No	No	No	No	No	NA	NA	No	NA	NA	NA	NA
Gdist:S284572_9752	BT31	43.666	0.0366	NA	No	No	No	No	No	No	No	0.519019293	0.83441924	Yes	NA	NA	NA	NA
Gdist:S246222_5790	BT31	44.584	-0.0021	NA	No	No	No	No	No	Yes (0.037)	No	NA	NA	No	NA	NA	NA	NA
Gdist:S57492_3241	BT31	45.559	-0.0036	NA	No	No	No	No	No	No	No	NA	NA	No	NA	NA	NA	NA
Gdist:S156988_213	BT31	45.562	0.0139	NA	No	No	No	No	No	No	No	NA	NA	No	NA	NA	NA	NA
Gdist:S107902_230	BT31	45.562	0.0098	NA	No	No	No	No	No	No	No	NA	NA	No	NA	NA	NA	NA
Gdist:S220636_231	BT31	45.564	0.0084	NA	No	No	No	No	No	No	No	0.380912863	0.94321023	Yes	NA	NA	NA	NA
Gdist:S274636_4816	BT31	45.564	-0.0136	NA	No	No	No	No	No	No	No	NA	NA	No	NA	NA	NA	NA
Gdist:S344029_3210	BT31	45.567	0.0073	NA	No	No	No	No	No	No	No	NA	NA	No	NA	NA	NA	NA
Gdist:S344029_2288	BT31	45.585	-0.0065	NA	No	No	No	No	No	No	No	NA	NA	No	NA	NA	NA	NA
cDNA:S453306_1429	BT31	48.318	0.0221	NA	No	No	No	No	No	No	No	NA	NA	No	NA	NA	NA	NA
cDNA:S185779_2734	BT31	48.341	0.0011	NA	No	No	No	No	No	No	No	NA	NA	No	NA	NA	NA	NA
Gdist:S70625_3771	BT31	48.738	0.032	NA	No	No	No	No	No	No	No	0.365723684	0.96233402	Yes	NA	NA	NA	NA
Gdist:S331269_7144	BT31	49.134	0.0333	NA	No	No	No	No	No	No	No	0.598035714	0.33707813	Yes	NA	NA	NA	NA
SalHit:S567449_927	BT31	49.136	0.0238	NA	Yes	No	No	No	No	No	No	NA	NA	No	NA	NA	NA	NA
Gdist:S144932_5186	BT31	49.138	0.0075	NA	No	No	No	No	No	No	No	NA	NA	No	NA	NA	NA	NA
Gdist:S413985_4517	BT31	49.998	0.0333	NA	No	No	No	No	No	No	No	0.24146648	0.17126866	Yes	NA	NA	NA	NA
Gdist:S83600_6529	BT31	49.998	0.0195	NA	No	No	No	No	No	No	No	NA	NA	No	NA	NA	NA	NA
Gdist:S129785_3163	BT31	49.998	0.0183	NA	No	No	No	No	No	No	No	NA	NA	No	NA	NA	NA	NA
Gene:S236632_813	BT31	49.998	0.0057	NA	No	No	No	No	No	Yes (0.023)	No	NA	NA	No	NA	NA	NA	NA
Gdist:S1329_4466	BT31	49.998	0.002	NA	Yes	No	No	No	No	No	No	NA	NA	No	NA	NA	NA	NA
cDNA:S4461_8291	BT31	50.992	0.0451	none	No	No	No	No	No	No	No	0.0095625	0.94404255	Yes	VAR RED	Reduced	NA	yes
Gdist:S116213_5101	BT31	51.95	-0.0076	NA	No	No	No	No	No	No	No	NA	NA	No	NA	NA	NA	NA
SalHit:S163322_1067	BT31	51.952	-0.0076	NA	No	No	No	No	No	No	No	NA	NA	No	NA	NA	NA	NA
Gdist:S236939_9881	BT31	51.955	0.0611	NA	No	No	No	No	No	No	No	0.9945	0.87029851	Yes	NA	NA	NA	NA
Gdist:S72514_7400	BT31	51.958	0.0232	NA	No	No	No	No	No	No	No	NA	NA	No	NA	NA	NA	NA
Gdist:S179805_2544	BT31	51.958	0.01	NA	No	No	No	No	No	No	No	NA	NA	No	NA	NA	NA	NA
cDNA:S441699_8582	BT31	51.958	0.0061	NA	No	No	No	No	No	No	No	NA	NA	No	NA	NA	NA	NA
Gdist:S450052_2191	BT31	51.958	0.0015	NA	No	No	No	No	No	No	No	NA	NA	No	NA	NA	NA	NA
cDNA:S74898_5777	BT31	51.958	0.0004	NA	No	No	No	No	No	No	No	NA	NA	No	NA	NA	NA	NA
cDNA:S74898_3892	BT31	51.958	-0.0011	NA	No	No	No	No	No	No	No	NA	NA	No	NA	NA	NA	NA
SalHit:S4303_9108	BT31	51.958	-0.0076	NA	No	No	No	No	No	No	No	NA	NA	No	NA	NA	NA	NA
cDNA:S74898_7495	BT31	51.958	-0.0078	NA	No	No	No	No	No	No	No	NA	NA	No	NA	NA	NA	NA
Gdist:S442718_4976	BT31	52.74	-0.0088	NA	No	No	No	No	No	No	No	NA	NA	No	NA	NA	NA	NA
SalHit:S260584_2458	BT31	52.96	0.0095	NA	No	No	No	No	No	No	No	NA	NA	No	NA	NA	NA	NA
SalHit:S260584_10644	BT31	52.961	-0.013	NA	No	No	No	No	No	No	No	NA	NA	No	NA	NA	NA	NA
cDNA:S201721_4183	BT31	52.962	0.0116	NA	No	No	No	No	No	Yes (0.043)	No	NA	NA	No	NA	NA	NA	NA
cDNA:S183231_554	BT31	52.962	-0.008	NA	No	No	No	No	No	No	No	NA	NA	No	NA	NA	NA	NA
cDNA:C123789935_234	BT31	52.963	0.0212	NA	No	No	No	No	No	Yes (0.005)	Yes (0.04)	NA	NA	No	NA	NA	NA	NA
SalHit:S343921_1954	BT31	52.964	0.0049	NA	No	No	No	No	No	No	No	NA	NA	No	NA	NA	NA	NA
cDNA:S262853_2386	BT31	52.965	0.0075	NA	No	No	No	No	No	No	No	NA	NA	No	NA	NA	NA	NA
cDNA:S569396_1043	BT31	52.965	0.003	NA	No	No	No	No	No	No	No	NA	NA	No	NA	NA	NA	NA
Gdist:S212380_1809	BT31	53.614	0.008	NA	No	No	No	No	No	No	No	NA	NA	No	NA	NA	NA	NA
Gdist:S212110_1152	BT31	53.795	0.0006	NA	No	No	No	No	No	No	No	NA	NA	No	NA	NA	NA	NA
SalHit:S392460_1601	BT31	53.795	0	NA	No	No	No	No	No	No	No	NA	NA	No	NA	NA	NA	NA
Gdist:S100942_9401	BT31	54.742	0.0333	NA	No	No	No	No	No	No	No	0.963553748	0.87029851	Yes	NA	NA	NA	NA
Gdist:S165925_1807	BT31	54.747	0.0211	NA	No	No	No	No	No	No	No	NA	NA	No	NA	NA	NA	NA
Gdist:S41747_6292	BT31	54.753	0.0222	NA	No	No	No	No	No	No	No	NA	NA	No	NA	NA	NA	NA
cDNA:S143080_508	BT31	55.006	0.0109	NA	No	No	No	No	No	No	No	NA	NA	No	NA	NA	NA	NA
Gdist:S234653_6232	BT31	55.269	0.0026	NA	No	No	No	No	No	No	No	0.475105263	0.92372067	Yes	NA	NA	NA	NA
Gdist:S169819_444	BT31	55.535	0.0036	NA	No	No	No	No	No	No	No	NA	NA	No	NA	NA	NA	NA
Gdist:S33382_9751	BT31	55.813	-0.007	NA	No	No	No	No	No	No	No	NA	NA	No	NA	NA	NA	NA
Gdist:S33382_6692	BT31	55.814	0.0166	NA	No	No	No	No	No	Yes (0.03)	No	NA	NA	No	NA	NA	NA	NA
Gdist:S254141_4306	BT31	55.814	0.0151	NA	No	No	No	No	No	No	No	NA	NA	No	NA	NA	NA	NA
Gdist:S96590_11234	BT31	55.814	0.0094	NA	No	No	No	No	No	No	No	NA	NA	No	NA	NA	NA	NA
cDNA:S63343_1741	BT31	55.814	0.0031	NA	Yes	No	No	No	No	No	No	NA	NA	No	NA	NA	NA	NA
Gdist:S90898_423	BT31	55.814	0.0031	NA	Yes	No	No	No	No	No	No	NA	NA	No	NA	NA	NA	NA
Gdist:S133403_1620	BT31	55.814	0.003	NA	No	No	No	No	No	No	No	NA	NA	No	NA	NA	NA	NA
Gdist:S244785_7396	BT31	55.814	-0.0022	NA	No	No	No	No	No	No	No	NA	NA	No	NA	NA	NA	NA
Gdist:S263264_6501	BT31	55.814	-0.0073	NA	No	No	No	No	No	No	No	NA	NA	No	NA	NA	NA	NA
cDNA:S404751_3670	BT31	56.163	0.0122	NA	No	No	No	No	No	No	No	NA	NA	No	NA	NA	NA	NA
Gdist:S218263_5586	BT31	56.481	0.0068	NA	No	No	No	No	No	No	No	NA	NA	No	NA	NA	NA	NA
Gdist:S146766_6312	BT31	56.821	0.0165	NA	No	No	No	No	No	No	No	NA	NA	No	NA	NA	NA	NA
Gene:S66998_3416	BT31	56.821	-0.0011	NA	No	No	No	No	No	No	No	NA	NA	No	NA	NA	NA	NA
SalHit:S192156_16661	BT31	56.821	-0.0044	NA	No	No	No	No	No	No	No	NA	NA	No	NA	NA	NA	NA
cDNA:S257543_2471	BT31	57.063	0.0117	NA	Yes	No	No	No	No	No	No	NA	NA	No	NA	NA	NA	NA
cDNA:S280660_1114	BT31	57.324	0.0254	NA	No	No	No	No	No	No	No	NA	NA	No	NA	NA	NA	NA
Gdist:S190352_2988	BT31	57.324	0.0208	NA	No	No	No	No	No	No	No	NA	NA	No	NA	NA	NA	NA
Gdist:S190352_7424	BT31	57.324	-0.0153	NA	No	No	No	No	No	No	No	NA	NA	No	NA	NA	NA	NA
SalHit:S274116_5651	BT31	57.369	-0.0026	NA	No	No	No	No	No	No	No	NA	NA	No	NA	NA	NA	NA
cDNA:S192149_1046	BT31	57.428	0.0442	NA	No	No	No	No	No	No	No	0.926563433	0.87029851	Yes	NA	NA	NA	NA
SalHit:S525583_945	BT31	57.735	0.0158	NA	No	No	No	No	No	No	No	NA	NA	No	NA	NA	NA	NA
Gdist:S105084_3459	BT31	57.735	0.0107	NA	No	No	No	No	No	No	No	NA	NA	No	NA	NA	NA	NA
SalarSNP:ESTNV_31852_56	BT31	57.735	0.0085	NA	No	No	No	No	No	No	No	NA	NA	No	NA	NA	NA	NA
cDNA:S58910_6486	BT31	57.735	0.0019	NA	No	No	No	No	No	No	No	NA	NA	No	NA	NA	NA	NA
Gdist:S758275_6493	BT31	57.735	-0.0074	NA	No	No	No	No	No	No	No	NA	NA	No	NA	NA	NA	NA
cDNA:S58910_7178	BT31	57.736	0.0284	NA	No	No	No	No	No	No	No	NA	NA	No	NA	NA	NA	NA
cDNA:S536151_3951	BT31	57.738	0.0317	NA	No	No	No	No	No	No	No	0.982691275	0.60417614	Yes	NA	NA	NA	NA
SalHit:S409379_746	BT31	57.744	-0.0141	NA	No	No	No	No	No	No	No	NA	NA	No	NA	NA	NA	NA
Gdist:S50383_2209	BT32	0	0.0087	NA	No	No	No	No	No	No	No	NA	NA	No	NA	NA	NA	NA
cDNA:S21692_2011	BT32	0.007	0.0228	Salmo tr	Yes	No	No	No	No	No	No	0.6875	0.0306	Yes	SKJ RED	NA	reduced	yes
cDNA:S198718_8135	BT32	5.19	-0.0054	NA	No	No	No	No	No	No	No	NA	NA	No	NA	NA	NA	NA
Gdist:S128049_4987	BT32	8.087	0.0147	NA	Yes	No	No	No	No	No	No	NA	NA	No	NA	NA	NA	NA
Gdist:S195276_11612	BT32	11.872	0.0316	NA	No	No	No	No	No	No	No	0.781630435	0.9985	Yes	NA	NA	NA	NA
Gdist:S152151_10254	BT32	14.691	0.0231	NA	No	No	No	No	No	No	No	NA	NA	No	NA	NA	NA	NA
Gdist:S152151_3686	BT32	14.691	0.0668	none	No	No	No	No	No	No	No	0.546428571	0	Yes	SKJ RED	NA	reduced	NA
Gdist:S231537_3739	BT32	16.884	0.0078	NA	No	No	No	No	No	No	No	NA	NA	No	NA	NA	NA	NA
cDNA:S228436_3377	BT32	17.365	0.042	NA	No	No	No	No	No	No	No	0.286875	0.48948276	Yes	NA	NA	NA	NA
Gdist:S234396_11260	BT32	20.59	0.0873	NA	No	No	No	No	No	No	No	0.982691275	0.18339041	Yes	NA	NA	NA	NA
Gdist:S199462_1772	BT32	25.121	0.0242	NA	No	No	No	No	No	No	No	NA	NA	No	NA	NA	NA	NA
SalHit:S139984_3276	BT32	25.346	0.0304	NA	No	No	No	No	No	No	No	0.7803	0.21973404	Yes	NA	NA	NA	NA
Gdist:S176939_7530	BT32	26.813	0.0584	NA	No	No	No	No	No	No	No	0.532767857	0.93956009	Yes	NA	NA	NA	NA
cDNA:S246052_6786	BT32	28.229	0.0239	NA	No	No	No	No	No	No	No	NA	NA	No	NA	NA	NA	NA
Gdist:S502119_7179	BT32	28.398	0.0638	NA	No	No	No	No	No	No	No	0.906372	0.98991	Yes	NA	NA	NA	NA
Gdist:S502119_5464	BT32	28.398	0.0308	NA	No	No	No	No	No	No	No	0.982691275	0.90315672	Yes	NA	NA	NA	NA
Gdist:S178165_3664	BT32	30.777	0.0709	NA	No	No	No	No	No	No	No	0.196637324	0.84541961	Yes	NA	NA	NA	NA
Gdist:S527567_2278	BT32	30.807	0.0199	NA	No	No	No	No	No	No	No	NA	NA	No	NA	NA	NA	NA
Gdist:S18053_3928	BT32	30.883	0.0568	NA	No	No	No	No	No	No	No	0.633830935	0.97060027	Yes	NA	NA	NA	NA
cDNA:S188721_1253	BT32	30.902	0.1159	NA	No	No	No	No	No	No	No	0.614833333	0.78459529	Yes	NA	NA	NA	NA
Gdist:S331500_6704	BT32	33.123	0.0005	NA	No	No	No	No	No	No	No	NA	NA	No	NA	NA	NA	NA
Gdist:S308860_8611	BT32	33.394	0.0507	NA	No	No	No	No	No	No	No	0.453596654	0.78459529	Yes	NA	NA	NA	NA
cDNA:S151018_2140	BT32	34.156	0.0186	NA	No	No	No	No	No	No	No	NA	NA	No	NA	NA	NA	NA
cDNA:S151018_3636	BT32	34.214	0.027	NA	No	No	No	No	No	No	No	NA	NA	No	NA	NA	NA	NA
Gdist:S252612_1361	BT32	34.274	0.0365	NA	No	No	No	No	No	No	No	0.0765	0.72269585	Yes	NA	NA	NA	NA
Gdist:S314660_8149	BT32	36.548	0.0231	NA	No	No	No	No	No	No	No	NA	NA	No	NA	NA	NA	NA
Gdist:S487573_3291	BT32	36.548	0.0026	NA	No	No	No	No	No	No	No	NA	NA	No	NA	NA	NA	NA
Gdist:S487573_6219	BT32	36.548	0.0009	NA	No	No	No	No	No	No	No	NA	NA	No	NA	NA	NA	NA
SalHit:S661159_2554	BT32	36.598	0.0826	NA	Yes	no	no	No	No	No	No	NA	NA	no	NA	NA	NA	NA
SalHit:S661159_285	BT32	36.632	0.0529	NA	Yes	No	No	No	No	No	No	0.057636986	0.53701987	Yes	NA	NA	NA	NA
cDNA:S249178_1898	BT32	38.407	0.026	NA	No	No	No	No	No	No	No	0.098582474	0.88732839	Yes	NA	NA	NA	NA

SalHit:S188619_2201	BT32	38.407	-0.0077	NA	No	No	No	No	No	No	No	NA	NA	No	NA	NA	NA	NA
SalHit:S188619_7117	BT32	38.407	-0.0091	NA	No	No	No	No	No	No	No	NA	NA	No	NA	NA	NA	NA
SalHit:S402209_5711	BT32	38.41	0.0119	NA	No	No	No	No	No	No	No	NA	NA	No	NA	NA	NA	NA
LD:S102196_408	BT32	38.412	0.01	NA	No	No	No	No	No	No	No	NA	NA	No	NA	NA	NA	NA
Gdist:S114376_8423	BT32	38.747	0.0011	NA	No	No	No	No	No	No	No	NA	NA	No	NA	NA	NA	NA
cDNA:S663695_501	BT32	40.57	0.0846	NA	Yes	No	No	No	No	Yes (0.007)	Yes (0.049)	0.7171875	0.69352273	Yes	NA	NA	NA	NA
Gdist:S100275_4086	BT32	41.411	0.0147	NA	No	No	No	No	No	No	No	NA	NA	No	NA	NA	NA	NA
cDNA:S423813_7520	BT32	42.252	0.0369	NA	No	No	No	No	No	Yes (0.007)	Yes (0.049)	0.906372	0.72269585	Yes	NA	NA	NA	NA
Gdist:S6246_5781	BT32	42.252	0.0343	NA	No	No	No	No	No	No	No	0.419853516	0.68332031	Yes	NA	NA	NA	NA
SalHit:S517254_2461	BT32	42.252	0.0289	NA	No	No	No	No	No	No	No	NA	NA	No	NA	NA	NA	NA
cDNA:S423813_4844	BT32	42.252	0.0278	NA	No	No	No	No	No	No	No	NA	NA	No	NA	NA	NA	NA
Gdist:S63697_2099	BT32	42.252	0.0227	NA	No	No	No	No	No	No	No	NA	NA	No	NA	NA	NA	NA
Gdist:S112167_5363	BT32	42.252	0.0163	NA	No	No	No	No	No	Yes (0.008)	Yes (0.049)	NA	NA	No	NA	NA	NA	NA
Gdist:S293856_3107	BT32	42.252	0.0142	NA	No	No	No	No	No	No	No	NA	NA	No	NA	NA	NA	NA
Gdist:S261547_6344	BT32	42.252	0.0141	NA	Yes	No	No	No	No	No	No	NA	NA	No	NA	NA	NA	NA
Gdist:S252008_4876	BT32	42.252	0.0123	NA	No	No	No	No	No	No	No	NA	NA	No	NA	NA	NA	NA
Gdist:S5462_9909	BT32	42.252	0.0118	NA	No	No	No	No	No	No	No	NA	NA	No	NA	NA	NA	NA
Gdist:S216078_1493	BT32	42.252	0.0088	NA	No	No	No	No	No	No	No	NA	NA	No	NA	NA	NA	NA
Gdist:S261547_3634	BT32	42.252	0.0072	NA	No	No	No	No	No	No	No	NA	NA	No	NA	NA	NA	NA
Gdist:S112167_4394	BT32	42.252	0.0044	NA	No	No	No	No	No	No	No	NA	NA	No	NA	NA	NA	NA
Gdist:S135483_11558	BT32	42.252	-0.0062	NA	No	No	No	No	No	No	No	NA	NA	No	NA	NA	NA	NA
Gdist:S333385_6513	BT32	42.252	0.0493	NA	No	No	No	No	No	No	No	0.916318681	0.01821429	Yes	SKJ INC	NA	increased	yes
Gdist:S351074_4648	BT32	43.187	0.0054	NA	No	No	No	No	No	No	No	NA	NA	No	NA	NA	NA	NA
cDNA:S434039_577	BT32	44.066	0.0268	NA	No	No	No	No	No	No	No	NA	NA	No	NA	NA	NA	NA
cDNA:S434039_906	BT32	44.066	0.0023	NA	No	No	No	No	No	No	No	NA	NA	No	NA	NA	NA	NA
SalHit:S323319_843	BT32	44.152	0.0356	NA	No	No	No	No	No	No	No	0.6875	0.88730408	Yes	NA	NA	NA	NA
Gdist:S595440_3015	BT32	44.354	0.0201	NA	No	No	No	No	No	No	No	NA	NA	No	NA	NA	NA	NA
Gdist:S571194_5965	BT32	44.542	0.0213	NA	No	No	No	No	No	No	No	NA	NA	No	NA	NA	NA	NA
Gdist:S182431_6153	BT32	44.79	0.0401	NA	No	No	No	No	No	No	No	0.996605505	0.98357143	Yes	NA	NA	NA	NA
Gdist:S230360_12338	BT32	45.13	0.0224	NA	No	No	No	No	No	No	No	NA	NA	No	NA	NA	NA	NA
Gdist:S356641_1485	BT32	45.867	0.0016	NA	No	No	No	No	No	No	No	NA	NA	No	NA	NA	NA	NA
Gdist:S97861_2200	BT32	45.898	0.0113	NA	No	No	No	No	No	No	No	NA	NA	No	NA	NA	NA	NA
Gdist:S97861_5521	BT32	45.929	0.0072	NA	No	No	No	No	No	No	No	NA	NA	No	NA	NA	NA	NA
cDNA:S652596_4392	BT32	45.961	0.0016	NA	No	No	No	No	No	Yes (0.021)	No	NA	NA	No	NA	NA	NA	NA
Gdist:S267826_5318	BT32	46.099	0.0208	NA	No	No	No	No	No	No	No	NA	NA	No	NA	NA	NA	NA
Gdist:S345718_1721	BT32	46.236	0.0109	NA	No	No	No	No	No	No	No	NA	NA	No	NA	NA	NA	NA
Gdist:S592675_2825	BT32	46.463	0.0284	NA	No	No	No	No	No	No	No	NA	NA	No	NA	NA	NA	NA
Gdist:S67288_4174	BT32	46.576	0.021	NA	No	No	No	No	No	No	No	NA	NA	No	NA	NA	NA	NA
Gdist:S67288_5220	BT32	46.579	0.0102	NA	No	No	No	No	No	No	No	NA	NA	No	NA	NA	NA	NA
Gdist:S493401_2830	BT32	46.61	0.0053	NA	No	No	No	No	No	No	No	NA	NA	No	NA	NA	NA	NA
Gdist:S493401_8841	BT32	46.799	0.0393	NA	No	No	No	No	No	No	No	0.462071168	0.95466066	Yes	NA	NA	NA	NA
Gdist:S548276_7541	BT32	47.029	0.0061	NA	No	No	No	No	No	No	No	NA	NA	No	NA	NA	NA	NA
Gene:S413875_2948	BT32	47.268	0.0096	NA	No	No	No	No	No	No	No	NA	NA	No	NA	NA	NA	NA
Gene:S13230_1136	BT32	47.478	0.0135	NA	No	No	No	No	No	No	No	NA	NA	No	NA	NA	NA	NA
Gene:S13230_584	BT32	49.557	-0.0158	NA	No	No	No	No	No	No	No	NA	NA	No	NA	NA	NA	NA
SalHit:S10848_3453	BT32	49.604	0.0393	NA	No	No	No	No	No	No	No	0.843830645	0.74151316	Yes	NA	NA	NA	NA
Gdist:S191480_3388	BT32	49.604	-0.0069	NA	No	No	No	No	No	No	No	NA	NA	No	NA	NA	NA	NA
cDNA:S77867_5110	BT32	49.604	-0.0111	NA	No	No	No	No	No	No	No	NA	NA	No	NA	NA	NA	NA
Gdist:S732557_6491	BT32	49.614	-0.0021	NA	Yes	No	No	No	No	No	No	NA	NA	No	NA	NA	NA	NA
SalHit:S155290_2396	BT32	49.626	0.0223	NA	No	No	No	No	No	No	No	NA	NA	No	NA	NA	NA	NA
Gdist:S432297_2110	BT32	50.039	0.0005	NA	No	No	No	No	No	No	No	NA	NA	No	NA	NA	NA	NA
Gdist:S463707_2000	BT32	50.799	0.0153	NA	No	No	No	No	No	No	No	NA	NA	No	NA	NA	NA	NA
cDNA:S163213_185	BT32	51.214	0.0005	NA	No	No	No	No	No	No	No	NA	NA	No	NA	NA	NA	NA
Gdist:S193538_1239	BT32	51.217	-0.0024	NA	No	No	No	No	No	No	No	NA	NA	No	NA	NA	NA	NA
SalHit:S483775_3667	BT32	52.012	0.0316	NA	No	No	No	No	No	No	No	0.489120209	0.9985	Yes	NA	NA	NA	NA
SalHit:S483775_2814	BT32	52.072	-0.0135	NA	No	No	No	No	No	No	No	NA	NA	No	NA	NA	NA	NA
SalHit:S155440_3655	BT32	52.131	0.0216	NA	No	No	No	No	No	Yes (0.013)	No	NA	NA	No	NA	NA	NA	NA
cDNA:S694773_326	BT32	52.646	0.026	NA	No	No	No	No	No	No	No	NA	NA	No	NA	NA	NA	NA
cDNA:S373842_3964	BT32	53.157	0.0287	NA	No	No	No	No	No	No	No	NA	NA	No	NA	NA	NA	NA
Gdist:S26048_8595	BT32	53.157	0.057	NA	No	No	No	No	No	No	No	0.028867925	0.41361244	Yes	VAR INC	increased	NA	NA
cDNA:S115738_2816	BT32	53.162	0.0321	NA	No	No	No	No	No	No	No	0.495931034	0.57490211	Yes	NA	NA	NA	NA
SalarSNP:ESTNV_33050_438	BT32	53.197	0.0267	NA	No	No	No	No	No	No	No	NA	NA	No	NA	NA	NA	NA
cDNA:S816485_4193	BT32	54.683	0.0358	NA	No	No	No	No	No	No	No	0.996605505	0.09807692	Yes	NA	NA	NA	NA
cDNA:S547212_811	BT32	54.983	0.0008	NA	No	No	No	No	No	No	No	NA	NA	No	NA	NA	NA	NA
cDNA:S506501_872	BT33	0	0.0144	NA	No	No	No	No	No	No	No	NA	NA	No	NA	NA	NA	NA
cDNA:S111749_3373	BT33	0.014	-0.0105	NA	No	No	No	No	No	No	No	NA	NA	No	NA	NA	NA	NA
Gdist:S147619_10126	BT33	3.686	0.0155	NA	No	No	No	No	No	No	No	NA	NA	No	NA	NA	NA	NA
Gdist:S70554_1338	BT33	4.437	-0.0035	NA	No	No	No	No	No	No	No	NA	NA	No	NA	NA	NA	NA
Gdist:S70554_8796	BT33	4.438	0.0219	NA	No	No	No	No	No	No	No	NA	NA	No	NA	NA	NA	NA
Gdist:S221165_2286	BT33	5.553	0.0044	NA	No	No	No	No	No	No	No	NA	NA	No	NA	NA	NA	NA
Gdist:S221165_3732	BT33	5.945	-0.0043	NA	No	No	No	No	No	No	No	NA	NA	No	NA	NA	NA	NA
Gdist:S276525_2435	BT33	5.946	0.0129	NA	No	No	No	No	No	No	No	NA	NA	No	NA	NA	NA	NA
cDNA:C124302365_425	BT33	6.192	0.0062	NA	No	No	No	No	No	No	No	NA	NA	No	NA	NA	NA	NA
cDNA:S793090_1806	BT33	12.947	0.01	NA	No	No	No	No	No	No	No	NA	NA	No	NA	NA	NA	NA
SalHit:S243747_5691	BT33	13.687	0.0202	NA	No	No	No	No	No	No	No	NA	NA	No	NA	NA	NA	NA
cDNA:S378656_8483	BT33	13.69	0.021	NA	No	No	No	No	No	No	No	NA	NA	No	NA	NA	NA	NA
cDNA:S378656_7609	BT33	13.69	0.0201	NA	No	No	No	No	No	No	No	NA	NA	No	NA	NA	NA	NA
cDNA:S184852_493	BT33	13.69	-0.0005	NA	No	No	No	No	No	No	No	NA	NA	No	NA	NA	NA	NA
cDNA:S110598_4394	BT33	13.69	-0.0044	NA	No	No	No	No	No	No	No	NA	NA	No	NA	NA	NA	NA
cDNA:S355742_2202	BT33	17.359	0.0194	NA	No	No	No	No	No	No	No	NA	NA	No	NA	NA	NA	NA
Gdist:S16994_6368	BT33	20.387	0.0097	NA	No	No	No	No	No	No	No	NA	NA	No	NA	NA	NA	NA
Gdist:S106795_4580	BT33	23.657	0.0141	NA	No	No	No	No	No	No	No	NA	NA	No	NA	NA	NA	NA
Gdist:S76057_7808	BT33	23.674	0.0085	NA	No	No	No	No	No	Yes (0.015)	No	NA	NA	No	NA	NA	NA	NA
Gdist:S89996_5449	BT33	23.729	0.0004	NA	No	No	No	No	No	No	No	NA	NA	No	NA	NA	NA	NA
Gdist:S203283_4061	BT33	26.34	0.0245	NA	No	No	No	No	No	No	No	NA	NA	No	NA	NA	NA	NA
cDNA:S672896_1109	BT33	26.38	0.0334	NA	No	No	No	No	No	No	No	0.365723684	0.23950935	Yes	NA	NA	NA	NA
Gdist:S18609_8176	BT33	30.215	0.0207	NA	No	No	No	No	No	Yes (0.003)	Yes (0.028)	NA	NA	No	NA	NA	NA	NA
cDNA:S223180_922	BT33	33.646	0.0353	NA	No	No	No	No	No	No	No	0.239872881	0.60473464	Yes	NA	NA	NA	NA
cDNA:S133525_2346	BT33	33.846	0.0533	NA	No	No	No	No	No	No	No	0.916490895	0.83808482	Yes	NA	NA	NA	NA
cDNA:S77337_2763	BT33	33.853	0.0292	NA	No	No	No	No	No	No	No	NA	NA	No	NA	NA	NA	NA
Gdist:S143339_5595	BT33	33.853	0.0225	NA	No	No	No	No	No	No	No	NA	NA	No	NA	NA	NA	NA
SalHit:S54501_5201	BT33	33.853	0.0173	NA	No	No	No	No	No	No	No	NA	NA	No	NA	NA	NA	NA
Gdist:S38858_5360	BT33	33.853	-0.0029	NA	No	No	No	No	No	No	No	NA	NA	No	NA	NA	NA	NA
cDNA:S77337_1096	BT33	33.853	-0.0139	NA	No	No	No	No	No	No	No	NA	NA	No	NA	NA	NA	NA
Gdist:S282772_7232	BT33	33.974	0.0508	NA	No	No	No	No	No	No	No	0.639604953	0.84677586	Yes	NA	NA	NA	NA
cDNA:S829392_887	BT33	35.436	0.02	NA	No	No	No	No	No	No	No	NA	NA	No	NA	NA	NA	NA
Gdist:S82464_8432	BT33	35.542	-0.0012	NA	No	No	No	No	No	No	No	NA	NA	No	NA	NA	NA	NA
cDNA:S128955_9570	BT33	35.653	0.0245	NA	No	No	No	No	No	No	No	NA	NA	No	NA	NA	NA	NA
Gdist:S341587_1300	BT33	35.765	0.0456	NA	Yes	No	No	No	No	No	No	0.41769	0.21798387	Yes	NA	NA	NA	NA
SalHit:S76374_963	BT33	35.765	-0.0085	NA	Yes	No	No	No	No	No	No	NA	NA	No	NA	NA	NA	NA
Gdist:S50778_1623	BT33	40.8	0.0627	NA	No	No	No	No	No	No	No	0.919848943	0.89727171	Yes	NA	NA	NA	NA
Gdist:S67374_8016	BT33	40.8	0.0398	NA	No	No	No	No	No	No	No	0.442817308	0.65440981	Yes	NA	NA	NA	NA
Gdist:S227373_2228	BT33	40.8	-0.0052	NA	No	No	No	No	No	No	No	NA	NA	No	NA	NA	NA	NA
cDNA:S175617_6065	BT33	40.8	-0.0076	NA	No	No	No	No	No	No	No	NA	NA	No	NA	NA	NA	NA
Gdist:S68863_5526	BT33	41.285	0.0073	NA	No	No	No	No	No	No	No	NA	NA	No	NA	NA	NA	NA
Gdist:S61025_6654	BT33	41.285	-0.0119	NA	No	No	No	No	No	No	No	NA	NA	No	NA	NA	NA	NA
Gdist:S45156_1049	BT33	41.51	0.0187	NA	No	No	No	No	No	No	No	NA	NA	No	NA	NA	NA	NA
Gdist:S143538_4173	BT33	41.731	0.0241	NA	No	No	No	No	No	No	No	NA	NA	No	NA	NA	NA	NA
Gdist:S143538_2537	BT33	41.731	0.022	NA	Yes	No	No	No	No	No	No	NA	NA	No	NA	NA	NA	NA
Gdist:S56481_7456	BT33	41.731	0.2033	NA	Yes	no	no	No	No	No	No	NA	NA	no	NA	NA	NA	NA
Gdist:S1127_7992	BT33	42.124	-0.01	NA	No	No	No	No	No	No	No	NA	NA	No	NA	NA	NA	NA
SalHit:S311726_3277	BT33	42.892	0.0281	NA	No	No	No	No	No	No	No	NA	NA	No	NA	NA	NA	NA
SalHit:S145953_5771	BT33	42.892	0.0132	NA	No	No	No	No	No	No	No	NA	NA	No	NA	NA	NA	NA
SalHit:S145953_9490	BT33	42.892	0.0113	NA	No	No	No	No	No	No	No	NA	NA	No	NA	NA	NA	NA
Gdist:S106382_7119	BT33	42.892	0.0112	NA	Yes	No	No	No	No	No	No	NA	NA	No	NA	NA	NA	NA
cDNA:S298750_5277	BT33	42.892	-0.0008	NA	No	No	No	No	No	No	No	NA	NA	No	NA	NA	NA	NA
cDNA:S52440_3397	BT33	43.332	0.0684	NA	No	No	No	No	No	No	No	0.921923077	0.60463173	Yes	NA	NA	NA	NA
cDNA:S499496_4647	BT33	43.332	0.0228	NA	No	No	No	No	No	No	No	NA	NA	No	NA	NA	NA	NA
Gdist:S308906_15933	BT33	43.364	0.0373	NA	No	No	No	No	No	No	No	0.926563433	0.61573171	Yes	NA	NA	NA	NA
Gdist:S584933_4695	BT33	43.847	-0.0019	NA	No	No	No	No	No	No	No	NA	NA	No	NA	NA	NA	NA
cDNA:S28762_5847	BT33	43.991	-0.0066	NA	No	No	No	No	No	No	No	NA	NA	No	NA	NA	NA	NA
SalHit:S688722_1069	BT33	45.524	0.0157	NA	No	No	No	No	No	No	No	NA	NA	No	NA	NA	NA	NA
cDNA:S129171_513	BT33	46.41	-0.0049	NA	No	No	No	No	No	No	No	NA	NA	No	NA	NA	NA	NA
SalHit:S125768_4275	BT34	0	0.0224	NA	No	No	No	No	No	No	No	NA	NA	No	NA	NA	NA	NA
Gdist:S17128_3728	BT34	0.936	0.0171	NA	Yes	No	No	No	No	No	No	NA	NA	No	NA	NA	NA	NA
Gdist:S73914_2266	BT34	0.936	0.0076	NA	No	No	No	No	No	No	No	NA	NA	No	NA	NA	NA	NA
cDNA:S286516_1913	BT34	0.939	-0.0059	NA	No	No	No	No	No	No	No	NA	NA	No	NA	NA	NA	NA

Gdist:S366614_5874	BT34	5.079	0.05	NA	No	No	No	No	No	No	No	0.953502155	0.52744737	Yes	NA	NA	NA	NA
Gdist:S366614_5034	BT34	5.263	-0.0047	NA	No	No	No	No	No	No	No	NA	NA	No	NA	NA	NA	NA
Gdist:S10297_8837	BT34	9.206	0.0128	NA	No	No	No	No	No	No	No	NA	NA	No	NA	NA	NA	NA
cDNA:S363729_1681	BT34	11.204	0.0188	NA	No	No	No	No	No	No	No	NA	NA	No	NA	NA	NA	NA
cDNA:S363729_4252	BT34	11.228	0.018	NA	Yes	No	No	No	No	No	No	NA	NA	No	NA	NA	NA	NA
cDNA:S704032_1342	BT34	12.045	0.0442	NA	No	No	No	No	No	No	No	0.982691275	0.77613306	Yes	NA	NA	NA	NA
Gdist:S72550_7757	BT34	12.045	0.033	NA	No	No	No	No	No	No	No	0.965664557	0.53217391	Yes	NA	NA	NA	NA
SalHit:S106841_6477	BT34	12.045	0.0075	NA	No	No	No	No	No	No	No	NA	NA	No	NA	NA	NA	NA
Gdist:S43844_2420	BT34	13.679	-0.0027	NA	No	No	No	No	No	No	No	NA	NA	No	NA	NA	NA	NA
Gdist:S43844_5503	BT34	15.378	0.0122	NA	No	No	No	No	No	No	No	NA	NA	No	NA	NA	NA	NA
Gdist:S247269_5485	BT34	15.406	-0.0012	NA	No	No	No	No	No	No	No	NA	NA	No	NA	NA	NA	NA
SalarSNP:ESTNV_36159_477	BT34	23.445	-0.0023	NA	No	No	No	No	No	No	No	NA	NA	No	NA	NA	NA	NA
Gdist:S80706_1302	BT34	23.836	0.0883	NA	No	No	No	No	No	No	No	0.225276074	0.63441532	Yes	NA	NA	NA	NA
Gdist:S28933_5728	BT34	27.391	0.0081	NA	No	No	No	No	No	No	No	NA	NA	No	NA	NA	NA	NA
cDNA:S252802_1574	BT34	28.202	0.0089	NA	No	No	No	No	No	No	No	NA	NA	No	NA	NA	NA	NA
Gdist:S66944_1366	BT34	28.734	-0.0069	NA	No	No	No	No	No	No	No	NA	NA	No	NA	NA	NA	NA
cDNA:S433169_1653	BT34	28.998	-0.0021	NA	No	No	No	No	No	No	No	NA	NA	No	NA	NA	NA	NA
cDNA:C123791721_258	BT34	29.063	0.0476	NA	No	No	No	No	No	No	No	0.957618026	0.24466216	Yes	NA	NA	NA	NA
cDNA:S48744_15351	BT34	33.945	0.0011	NA	No	No	No	No	No	No	No	NA	NA	No	NA	NA	NA	NA
cDNA:S48744_11537	BT34	34.187	0.0224	NA	No	No	No	No	No	No	No	NA	NA	No	NA	NA	NA	NA
cDNA:S48744_3876	BT34	34.187	0.0089	NA	No	No	No	No	No	No	No	NA	NA	No	NA	NA	NA	NA
SalHit:S226473_1887	BT34	34.193	0.0218	NA	No	No	No	No	No	No	No	NA	NA	No	NA	NA	NA	NA
Gdist:S52659_1703	BT34	36.201	0.0218	NA	No	No	No	No	No	No	No	NA	NA	No	NA	NA	NA	NA
cDNA:S263051_694	BT34	36.201	0.0179	NA	No	No	No	No	No	Yes (0)	Yes (0.004)	NA	NA	No	NA	NA	NA	NA
Gdist:S239427_14418	BT34	37.14	0.0135	NA	No	No	No	No	No	No	No	NA	NA	No	NA	NA	NA	NA
Gdist:S239427_13768	BT34	37.14	0.0124	NA	No	No	No	No	No	No	No	NA	NA	No	NA	NA	NA	NA
Gdist:S71493_4035	BT34	37.142	0.0207	NA	No	No	No	No	No	Yes (0)	Yes (0.004)	NA	NA	No	NA	NA	NA	NA
Gdist:S223597_6005	BT34	39.189	0.0087	NA	No	No	No	No	No	No	No	NA	NA	No	NA	NA	NA	NA
cDNA:S167866_4323	BT34	41.304	0.0108	NA	No	No	No	No	No	Yes (0.004)	Yes (0.035)	NA	NA	No	NA	NA	NA	NA
Gdist:S139852_9194	BT34	44.437	0.0101	NA	No	No	No	No	No	No	No	NA	NA	No	NA	NA	NA	NA
Gdist:S17607_5698	BT34	44.437	-0.0115	NA	No	No	No	No	No	Yes (0.023)	No	NA	NA	No	NA	NA	NA	NA
Gdist:S139852_8868	BT34	44.712	0.0253	NA	No	No	No	No	No	Yes (0)	Yes (0.006)	NA	NA	No	NA	NA	NA	NA
Gdist:S215004_8796	BT34	44.969	0.0207	NA	No	No	No	No	No	No	No	NA	NA	No	NA	NA	NA	NA
Gdist:S137971_2840	BT34	44.987	0.0038	NA	Yes	No	No	No	No	No	No	NA	NA	No	NA	NA	NA	NA
Gdist:S418082_4014	BT34	44.99	0.013	NA	No	No	No	No	No	No	No	NA	NA	No	NA	NA	NA	NA
cDNA:S553568_2443	BT34	44.993	0.0129	NA	No	No	No	No	No	Yes (0.014)	No	NA	NA	No	NA	NA	NA	NA
Gdist:S96446_10551	BT34	44.993	0.0026	NA	No	No	No	No	No	No	No	NA	NA	No	NA	NA	NA	NA
Gdist:S27650_3735	BT34	45.352	0.004	NA	No	No	No	No	No	No	No	NA	NA	No	NA	NA	NA	NA
Gdist:S52880_6577	BT34	45.693	0.0102	NA	Yes	No	No	No	No	No	No	NA	NA	No	NA	NA	NA	NA
Gdist:S454628_7586	BT34	45.693	0.0056	NA	No	No	No	No	No	No	No	NA	NA	No	NA	NA	NA	NA
SalHit:S373646_810	BT34	45.693	-0.0089	NA	No	No	No	No	No	Yes (0.044)	No	NA	NA	No	NA	NA	NA	NA
Gdist:S19007_438	BT34	45.697	0.0032	NA	No	No	No	No	No	No	No	NA	NA	No	NA	NA	NA	NA
Gdist:S172613_436	BT34	48.048	0.0717	NA	No	No	No	No	No	Yes (0.014)	No	0.705336538	0.84566784	Yes	NA	NA	NA	NA
Gdist:S289677_7267	BT34	48.048	0.0106	NA	No	No	No	No	No	No	No	NA	NA	No	NA	NA	NA	NA
Gdist:S452881_2913	BT34	48.211	0.0265	NA	No	No	No	No	No	Yes (0.014)	No	NA	NA	No	NA	NA	NA	NA
Gdist:S48218_2914	BT34	48.578	0.0378	NA	No	No	No	No	No	No	No	0.745423228	0.800955	Yes	NA	NA	NA	NA
Gdist:S26094_4843	BT34	50.18	-0.0055	NA	No	No	No	No	No	No	No	NA	NA	No	NA	NA	NA	NA
Gdist:S505268_3066	BT34	50.203	0.014	NA	No	No	No	No	No	No	No	NA	NA	No	NA	NA	NA	NA
Gdist:S283576_5326	BT34	50.224	0.0091	NA	Yes	No	No	No	No	No	No	NA	NA	No	NA	NA	NA	NA
Gdist:S102207_543	BT34	50.224	-0.0082	NA	No	No	No	No	No	No	No	NA	NA	No	NA	NA	NA	NA
Gdist:S270855_4493	BT34	50.675	0.0001	NA	No	No	No	No	No	No	No	NA	NA	No	NA	NA	NA	NA
cDNA:S736834_3880	BT34	51.182	-0.0004	NA	No	No	No	No	No	No	No	NA	NA	No	NA	NA	NA	NA
cDNA:S36850_3607	BT34	51.661	0.028	NA	No	No	No	No	No	Yes (0.013)	No	NA	NA	No	NA	NA	NA	NA
cDNA:S36850_6296	BT34	52.042	0.0094	NA	No	No	No	No	No	Yes (0.004)	Yes (0.035)	NA	NA	No	NA	NA	NA	NA
Gdist:S708093_5089	BT34	52.465	0.0317	NA	No	No	No	No	No	No	No	0.531768293	0.97212245	Yes	NA	NA	NA	NA
cDNA:S203107_4811	BT34	52.918	-0.0008	NA	No	No	No	No	No	Yes (0.013)	No	NA	NA	No	NA	NA	NA	NA
Gdist:S67077_5510	BT34	52.919	0.0346	NA	No	No	No	No	No	No	No	0.77015896	0.61532609	Yes	NA	NA	NA	NA
Gdist:S511570_3346	BT34	52.919	0.0157	NA	No	No	No	No	No	No	No	NA	NA	No	NA	NA	NA	NA
cDNA:S76268_7320	BT34	52.919	0.0152	NA	No	No	No	No	No	No	No	NA	NA	No	NA	NA	NA	NA
LD:S439680_11105	BT34	53.091	0.01	NA	No	No	No	No	No	No	No	NA	NA	No	NA	NA	NA	NA
Gdist:S97591_1821	BT34	53.333	0.0213	NA	No	No	No	No	No	No	No	NA	NA	No	NA	NA	NA	NA
Gdist:S97591_2753	BT34	53.58	0.0214	NA	No	No	No	No	No	No	No	NA	NA	No	NA	NA	NA	NA
cDNA:S282715_14917	BT34	53.837	0.0043	NA	No	No	No	No	No	No	No	NA	NA	No	NA	NA	NA	NA
cDNA:S503987_1310	BT34	54.089	0.0291	NA	Yes	No	No	No	No	No	No	NA	NA	No	NA	NA	NA	NA
SalHit:S131311_2546	BT34	54.089	0.0235	NA	No	No	No	No	No	No	No	NA	NA	No	NA	NA	NA	NA
cDNA:S42272_2039	BT34	54.089	0.0171	NA	No	No	No	No	No	No	No	NA	NA	No	NA	NA	NA	NA
SalHit:S594636_3547	BT34	54.089	0.0079	NA	No	No	No	No	No	No	No	NA	NA	No	NA	NA	NA	NA
SalHit:S355030_2257	BT34	54.089	0.0004	NA	No	No	No	No	No	No	No	NA	NA	No	NA	NA	NA	NA
cDNA:S549631_6781	BT34	54.089	-0.0069	NA	No	No	No	No	No	No	No	NA	NA	No	NA	NA	NA	NA
SalHit:S39061_10111	BT34	54.51	0.0332	NA	No	No	No	No	No	No	No	0.578414634	0.97060027	Yes	NA	NA	NA	NA
cDNA:S299605_718	BT34	55.201	0.0061	NA	No	No	No	No	No	No	No	NA	NA	No	NA	NA	NA	NA
Gdist:S105141_3863	BT34	55.204	0.0148	NA	No	No	No	No	No	No	No	NA	NA	No	NA	NA	NA	NA
Gdist:S105141_4123	BT34	55.204	0.0032	NA	No	No	No	No	No	No	No	NA	NA	No	NA	NA	NA	NA
SalarSNP:ESTV_17160_114	BT34	55.204	-0.0033	NA	No	No	No	No	No	No	No	NA	NA	No	NA	NA	NA	NA
SalHit:S468867_988	BT34	55.205	0.0012	NA	No	No	No	No	No	No	No	NA	NA	No	NA	NA	NA	NA
SalHit:S673707_838	BT34	55.205	-0.0008	NA	No	No	No	No	No	No	No	NA	NA	No	NA	NA	NA	NA
SalHit:S468867_1710	BT34	55.212	0.0158	NA	No	No	No	No	No	No	No	NA	NA	No	NA	NA	NA	NA
Gdist:S475841_1584	BT34	55.212	0.0085	NA	No	No	No	No	No	No	No	NA	NA	No	NA	NA	NA	NA
Gdist:S475841_9570	BT34	55.213	0.0159	NA	No	No	No	No	No	No	No	NA	NA	No	NA	NA	NA	NA
Gdist:S339816_4291	BT35	0	0.0213	NA	No	No	No	No	No	No	No	NA	NA	No	NA	NA	NA	NA
cDNA:S38989_1707	BT35	2.682	0.0446	NA	No	No	No	No	No	No	No	0.870811856	0.41008413	Yes	NA	NA	NA	NA
cDNA:S530503_2428	BT35	2.682	0.0375	NA	No	No	No	No	No	No	No	0.7171875	0.73333893	Yes	NA	NA	NA	NA
SalHit:S335584_5764	BT35	2.682	0.03	NA	No	No	No	No	No	No	No	NA	NA	No	NA	NA	NA	NA
Gdist:S69367_3982	BT35	2.682	0.0163	NA	Yes	No	No	No	No	No	No	NA	NA	No	NA	NA	NA	NA
Gdist:S238568_3355	BT35	2.682	0.0027	NA	No	No	No	No	No	No	No	NA	NA	No	NA	NA	NA	NA
Gdist:S70913_7507	BT35	10.047	0.0298	NA	No	No	No	No	No	No	No	0.462071168	0.82025797	Yes	NA	NA	NA	NA
cDNA:S49962_7061	BT35	13.089	-0.0079	NA	No	No	No	No	No	No	No	NA	NA	No	NA	NA	NA	NA
cDNA:S49962_2185	BT35	13.089	-0.0118	NA	No	No	No	No	No	No	No	NA	NA	No	NA	NA	NA	NA
Gdist:S35267_5176	BT35	13.231	0.0409	NA	No	No	No	No	No	No	No	0.580451482	0.4818314	Yes	NA	NA	NA	NA
cDNA:S160349_4078	BT35	13.673	0.0388	NA	No	No	No	No	No	No	No	0.220947205	0.69580402	Yes	NA	NA	NA	NA
cDNA:S384021_4373	BT35	13.673	0.031	NA	No	No	No	No	No	No	No	0.982691275	0.66132237	Yes	NA	NA	NA	NA
Gdist:S92280_8106	BT35	13.673	0.0267	NA	No	No	No	No	No	No	No	NA	NA	No	NA	NA	NA	NA
Gdist:S127042_3442	BT35	13.673	0.0143	NA	No	No	No	No	No	No	No	NA	NA	No	NA	NA	NA	NA
Gdist:S47976_3194	BT35	13.675	0.0422	NA	No	No	No	No	No	No	No	0.742859281	0.74156455	Yes	NA	NA	NA	NA
cDNA:S77853_1811	BT35	13.69	0.0294	NA	No	No	No	No	No	No	No	0.753772016	0.75593211	Yes	NA	NA	NA	NA
Gdist:S339816_3071	BT35	13.736	0.0139	NA	Yes	No	No	No	No	No	No	NA	NA	No	NA	NA	NA	NA
cDNA:S660504_2091	BT35	14.004	-0.0019	NA	No	No	No	No	No	No	No	NA	NA	No	NA	NA	NA	NA
Gdist:S156620_726	BT35	14.265	0.0116	NA	No	No	No	No	No	No	No	NA	NA	No	NA	NA	NA	NA
cDNA:S699696_588	BT35	14.648	0.0015	NA	Yes	No	No	No	No	No	No	NA	NA	No	NA	NA	NA	NA
Gdist:S1887_3917	BT35	22.678	0.0141	NA	Yes	No	No	No	No	No	No	NA	NA	No	NA	NA	NA	NA
Gdist:S455907_4238	BT35	22.995	0.0292	NA	No	No	No	No	No	No	No	0.726987578	0.46026971	Yes	NA	NA	NA	NA
Gdist:S270436_3843	BT35	25.739	0.0141	NA	No	No	No	No	No	No	No	NA	NA	No	NA	NA	NA	NA
cDNA:S508312_687	BT35	30.254	-0.0041	NA	No	No	No	No	No	No	No	NA	NA	No	NA	NA	NA	NA
Gdist:S97704_2827	BT35	39.903	0.0037	NA	No	No	No	No	No	No	No	NA	NA	No	NA	NA	NA	NA
Gdist:S583411_3893	BT35	40.92	0.0108	NA	No	No	No	No	No	No	No	NA	NA	No	NA	NA	NA	NA
SalHit:S516356_4851	BT35	41.555	-0.0062	NA	No	No	No	No	No	No	No	NA	NA	No	NA	NA	NA	NA
Gdist:S150832_4425	BT35	43.496	0.0003	NA	No	No	No	No	No	No	No	NA	NA	No	NA	NA	NA	NA
cDNA:S196052_8001	BT35	43.643	0.0112	NA	No	No	No	No	No	No	No	NA	NA	No	NA	NA	NA	NA
Gene:S177672_925	BT35	43.718	0.0116	NA	No	No	No	No	No	No	No	NA	NA	No	NA	NA	NA	NA
cDNA:S59534_1178	BT35	45.538	0.0339	NA	No	No	No	No	No	No	No	0.916490895	0.87582368	Yes	NA	NA	NA	NA
Gdist:S22228_6945	BT35	46.881	0.0231	NA	No	No	No	No	No	Yes (0.014)	No	NA	NA	No	NA	NA	NA	NA
Gdist:S96583_8444	BT35	51.462	0.0256	NA	No	No	No	No	No	No	No	NA	NA	No	NA	NA	NA	NA
Gdist:S273471_3154	BT35	52.242	0.0026	NA	No	No	No	No	No	No	No	NA	NA	No	NA	NA	NA	NA
LD:S594729_17207	BT35	53.021	0.0332	NA	Yes	No	No	No	No	No	No	0.682433921	0.3565678	Yes	NA	NA	NA	NA
Gdist:S321737_7439	BT35	53.023	0.0468	NA	No	No	No	No	No	No	No	0.115121359	0.57143182	Yes	NA	NA	NA	NA
cDNA:S678589_1098	BT35	53.023	0.0097	NA	No	No	No	No	No	No	No	NA	NA	No	NA	NA	NA	NA
Gdist:S265937_4642	BT35	53.825	0.0671	NA	No	No	No	No	No	No	No	0.050625	0.65440981	Yes	NA	NA	NA	NA
Gdist:S265937_3238	BT35	53.825	0.0284	NA	No	No	No	No	No	No	No	NA	NA	No	NA	NA	NA	NA
cDNA:S168148_15564	BT35	53.828	-0.0023	NA	No	No	No	No	No	No	No	NA	NA	No	NA	NA	NA	NA
Gdist:S261737_3653	BT35	55.235	0.0208	NA	No	No	No	No	No	No	No	NA	NA	No	NA	NA	NA	NA
Gdist:S261737_6399	BT35	55.235	-0.0056	NA	No	No	No	No	No	Yes (0.035)	No	NA	NA	No	NA	NA	NA	NA
Gdist:S261737_9811	BT35	55.24	0.0042	NA	No	No	No	No	No	No	No	NA	NA	No	NA	NA	NA	NA
Gdist:S238521_6414	BT35	57.291	-0.0006	NA	No	No	No	No	No	No	No	NA	NA	No	NA	NA	NA	NA
Gdist:S62543_5457	BT35	57.301	0.0012	NA	No	No	No	No	No	No	No	NA	NA	No	NA	NA	NA	NA
Gdist:S364600_3193	BT35	57.311	0.0377	NA	No	No	No	No	No	No	No	0.907678571	0.88194268	Yes	NA	NA	NA	NA
SalHit:S614561_3987	BT35	57.311	0.0167	NA	No	No	No	No	No	No	No	NA	NA	No	NA	NA	NA	NA
cDNA:S10356_987	BT35	57.311	0.0082	NA	Yes	No	No	No	No	No	No	NA	NA	No	NA	NA	NA	NA

Gdist:S52502_2377	BT35	57.311	0.0054	NA	Yes	No	No	No	No	No	No	NA	NA	No	NA	NA	NA	NA
SalarSNP:GCR_cBin18664_Ctg	BT35	57.311	0.0371	NA	No	No	No	No	No	No	No	0.016276596	0.87819512	Yes	VAR INC	increased	NA	NA
cDNA:S291123_361	BT35	58.4	0.0077	NA	No	No	No	No	No	Yes (0)	Yes (0.004)	NA	NA	No	NA	NA	NA	NA
cDNA:S470748_6041	BT35	59.676	0.0265	NA	Yes	No	No	No	No	No	No	NA	NA	No	NA	NA	NA	NA
cDNA:S470748_4209	BT35	59.677	0.0538	none	Yes	No	No	No	No	No	No	0.016276596	0.53217391	Yes	VAR RED	Reduced	NA	yes
Gdist:S210000_6349	BT35	59.803	0.0085	NA	Yes	No	No	No	No	No	No	NA	NA	No	NA	NA	NA	NA
Gdist:S300381_5583	BT35	60.451	0.0157	NA	No	No	No	No	No	No	No	NA	NA	No	NA	NA	NA	NA
Gdist:S165349_3716	BT35	60.452	0.0747	PREDIC	No	No	Yes	No	No	Yes (0.023)	No	0.533010355	0.93621951	Yes	Outlier Skjern	Outlier Skj	NA	yes
Gdist:S20071_2696	BT35	61.653	-0.0035	NA	No	No	No	No	No	No	No	NA	NA	No	NA	NA	NA	NA
Gdist:S259622_1374	BT35	63.868	0.007	NA	No	No	No	No	No	No	No	NA	NA	No	NA	NA	NA	NA
Gdist:S164572_6264	BT35	64.363	0.0296	NA	Yes	No	No	No	No	No	No	NA	NA	No	NA	NA	NA	NA
Gdist:S291969_4425	BT35	64.379	0.0455	NA	No	No	No	No	No	No	No	0.213317308	0.05976563	Yes	NA	NA	NA	NA
Gdist:S377665_4790	BT35	64.379	0.0426	NA	No	No	No	No	No	No	No	0.916490895	0.48116071	Yes	NA	NA	NA	NA
cDNA:S311358_11177	BT35	64.379	0.0201	NA	No	No	No	No	No	No	No	NA	NA	No	NA	NA	NA	NA
cDNA:S639300_3047	BT35	64.379	0.0504	PREDIC	No	No	No	No	No	No	No	0.982691275	0	Yes	SKJ RED	NA	reduced	NA
Gdist:S490487_193	BT35	64.877	-0.0029	NA	No	No	No	No	No	No	No	NA	NA	No	NA	NA	NA	NA
SalHit:S259229_3718	BT35	64.942	0.0529	NA	No	No	No	No	No	No	No	0.735576923	0.73347996	Yes	NA	NA	NA	NA
Gdist:S208592_5727	BT35	64.942	0.009	NA	No	No	No	No	No	No	No	NA	NA	No	NA	NA	NA	NA
cDNA:S31330_3843	BT35	64.942	-0.0103	NA	No	No	No	No	No	No	No	NA	NA	No	NA	NA	NA	NA
Gdist:S322656_3407	BT35	65.712	0.0202	NA	No	No	No	No	No	No	No	NA	NA	No	NA	NA	NA	NA
cDNA:S42646_1319	BT35	66.066	0.0419	NA	No	No	No	No	No	No	No	0.357476636	0.05976563	Yes	NA	NA	NA	NA
Gdist:S51737_6399	BT35	67.135	-0.0047	NA	No	No	No	No	No	No	No	NA	NA	No	NA	NA	NA	NA
SalHit:S82254_6678	BT35	67.138	0.0413	NA	No	No	No	No	No	No	No	0.262581081	0.21798387	Yes	NA	NA	NA	NA
cDNA:S38713_4675	BT35	67.138	0.0162	NA	No	No	No	No	No	No	No	NA	NA	No	NA	NA	NA	NA
cDNA:S68977_1000	BT35	67.138	-0.003	NA	No	No	No	No	No	No	No	NA	NA	No	NA	NA	NA	NA
Gdist:S100352_5216	BT35	67.544	0.0042	NA	No	No	No	No	No	No	No	NA	NA	No	NA	NA	NA	NA
Gdist:S85275_7034	BT35	67.562	0.0083	NA	No	No	No	No	No	No	No	NA	NA	No	NA	NA	NA	NA
Gdist:S72843_2519	BT35	67.617	0.0483	NA	No	No	No	No	No	No	No	0.530792308	0.74416667	Yes	NA	NA	NA	NA
Gdist:S275336_5237	BT35	67.617	0.0335	NA	No	No	No	No	No	No	No	0.405728745	0.76824153	Yes	NA	NA	NA	NA
cDNA:S125095_4124	BT35	67.618	0.0267	NA	No	No	No	No	No	No	No	NA	NA	No	NA	NA	NA	NA
Gdist:S306037_1444	BT35	67.618	0.0198	NA	No	No	No	No	No	No	No	NA	NA	No	NA	NA	NA	NA
Gdist:S77596_8698	BT35	67.619	0.0145	NA	No	No	No	No	No	No	No	NA	NA	No	NA	NA	NA	NA
SalHit:S104654_669	BT35	67.671	0.0062	NA	No	No	No	No	No	No	No	NA	NA	No	NA	NA	NA	NA
cDNA:S600558_675	BT35	68.279	0.0024	NA	No	No	No	No	No	No	No	NA	NA	No	NA	NA	NA	NA
cDNA:S335445_3045	BT35	68.293	0.0053	NA	No	No	No	No	No	No	No	NA	NA	No	NA	NA	NA	NA
Gdist:S25408_5243	BT36	0	0.0414	NA	No	No	No	No	No	No	No	0.719672557	0.7167233	Yes	NA	NA	NA	NA
cDNA:S784689_1447	BT36	0.228	0.0055	NA	No	No	No	No	No	Yes (0.039)	No	NA	NA	No	NA	NA	NA	NA
Gdist:S42000_1205	BT36	3.172	0.0586	NA	No	No	No	No	No	No	No	0.367597403	0.49897556	Yes	NA	NA	NA	NA
Gdist:S53778_2726	BT36	5.18	-0.001	NA	No	No	No	No	No	Yes (0.013)	No	NA	NA	No	NA	NA	NA	NA
Gdist:S89559_2936	BT36	7.488	0.0041	NA	No	No	No	No	No	Yes (0.025)	No	NA	NA	No	NA	NA	NA	NA
cDNA:S52985_1976	BT36	10.105	0.0617	NA	No	No	No	No	No	No	No	0.682433921	0.24213303	Yes	NA	NA	NA	NA
cDNA:S15772_1853	BT36	10.311	0.0033	NA	No	No	No	No	No	No	No	NA	NA	No	NA	NA	NA	NA
SalHit:S453385_428	BT36	10.324	0.0074	NA	No	No	No	No	No	No	No	NA	NA	No	NA	NA	NA	NA
SalHit:S453385_786	BT36	10.36	0.0054	NA	No	No	No	No	No	No	No	NA	NA	No	NA	NA	NA	NA
SalHit:S453385_2176	BT36	10.361	0.035	NA	No	No	No	No	No	No	No	0.916490895	0.69352273	Yes	NA	NA	NA	NA
SalHit:S316727_1311	BT36	12.98	0.0183	NA	No	No	No	No	No	No	No	NA	NA	No	NA	NA	NA	NA
Gdist:S93662_4606	BT36	19.715	0.0374	NA	No	No	No	No	No	Yes (0.007)	Yes (0.049)	0.911143868	0.92678416	Yes	NA	NA	NA	NA
Gdist:S366036_2568	BT36	19.715	0.0139	NA	No	No	No	No	No	No	No	NA	NA	No	NA	NA	NA	NA
Gdist:S93662_250	BT36	19.715	0.0088	NA	No	No	No	No	No	No	No	NA	NA	No	NA	NA	NA	NA
SalHit:S205573_1241	BT36	19.806	0.029	NA	No	No	No	No	No	No	No	NA	NA	No	NA	NA	NA	NA
SalHit:S592297_684	BT36	20.063	0.1711	NA	Yes	no	no	No	No	No	No	NA	NA	no	NA	NA	NA	NA
SalHit:S592297_1046	BT36	20.325	0.0377	NA	No	No	No	No	No	No	No	0.837857143	0.905231	Yes	NA	NA	NA	NA
SalHit:S592297_2656	BT36	20.406	0.0397	NA	No	No	No	No	No	No	No	0.954878049	0.72269585	Yes	NA	NA	NA	NA
SalarSNP:ESTNV_32349_576	BT36	20.491	0.0529	NA	No	No	No	No	No	No	No	0.841225314	0.72169811	Yes	NA	NA	NA	NA
cDNA:S441715_114	BT36	20.491	0.0118	NA	No	No	No	No	No	No	No	NA	NA	No	NA	NA	NA	NA
cDNA:S441715_1042	BT36	20.492	0.0091	NA	Yes	No	No	No	No	No	No	NA	NA	No	NA	NA	NA	NA
SalHit:S105331_3668	BT36	25.597	0.0049	NA	Yes	No	No	No	No	No	No	NA	NA	No	NA	NA	NA	NA
Gdist:S36228_3046	BT36	25.668	0.0136	NA	No	No	No	No	No	No	No	NA	NA	No	NA	NA	NA	NA
Gdist:S385256_2310	BT36	25.688	0.0021	NA	No	No	No	No	No	No	No	NA	NA	No	NA	NA	NA	NA
SalHit:S97503_354	BT36	27.062	0.0247	NA	No	No	No	No	No	Yes (0.043)	No	NA	NA	No	NA	NA	NA	NA
SalHit:S463531_4086	BT36	27.122	0.0254	NA	No	No	No	No	No	Yes (0.012)	No	NA	NA	No	NA	NA	NA	NA
SalHit:C124022803_337	BT36	27.122	0.0154	NA	No	No	No	No	No	No	No	NA	NA	No	NA	NA	NA	NA
cDNA:S62874_2994	BT36	27.564	0.0122	NA	No	No	No	No	No	No	No	NA	NA	No	NA	NA	NA	NA
cDNA:S309020_2087	BT36	31.896	0.0043	NA	No	No	No	No	No	No	No	NA	NA	No	NA	NA	NA	NA
Gdist:S98509_1652	BT36	34.782	0.026	NA	No	No	No	No	No	No	No	NA	NA	No	NA	NA	NA	NA
Gdist:S409619_4086	BT36	38.073	0.0245	NA	No	No	No	No	No	Yes (0.023)	No	NA	NA	No	NA	NA	NA	NA
Gdist:S12991_3989	BT36	38.091	0.0185	NA	No	No	No	No	No	No	No	NA	NA	No	NA	NA	NA	NA
Gdist:S583414_6553	BT36	40.454	-0.0031	NA	No	No	No	No	No	No	No	NA	NA	No	NA	NA	NA	NA
cDNA:C123447604_140	BT36	40.491	-0.0102	NA	No	No	No	No	No	No	No	NA	NA	No	NA	NA	NA	NA
cDNA:S192515_5942	BT36	40.56	0.0085	NA	No	No	No	No	No	No	No	NA	NA	No	NA	NA	NA	NA
cDNA:S139611_3301	BT36	42.559	0.0836	Salmo s	No	Yes	No	No	No	No	No	0.028867925	0.83229167	Yes	VAR RED + Outl	Reduced	NA	yes
cDNA:S97964_2369	BT36	42.817	0.0074	NA	No	No	No	No	No	No	No	NA	NA	No	NA	NA	NA	NA
SalHit:S330374_228	BT36	44.848	-0.0089	NA	No	No	No	No	No	No	No	NA	NA	No	NA	NA	NA	NA
cDNA:C123587298_320	BT36	48.485	-0.003	NA	No	No	No	No	No	No	No	NA	NA	No	NA	NA	NA	NA
SalarSNP:ESTV_21544_726	BT37	0	0.0053	NA	No	No	No	No	No	No	No	NA	NA	No	NA	NA	NA	NA
SalHit:S568137_774	BT37	0	-0.0041	NA	No	No	No	No	No	No	No	NA	NA	No	NA	NA	NA	NA
cDNA:S340707_312	BT37	1.036	0.0062	NA	No	No	No	No	No	No	No	NA	NA	No	NA	NA	NA	NA
cDNA:S415421_1829	BT37	1.562	-0.0118	NA	No	No	No	No	No	No	No	NA	NA	No	NA	NA	NA	NA
cDNA:S404147_2649	BT37	1.835	-0.0117	NA	No	No	No	No	No	Yes (0.023)	No	NA	NA	No	NA	NA	NA	NA
Gdist:S29240_1489	BT37	3.946	0.0251	NA	No	No	No	No	No	No	No	NA	NA	No	NA	NA	NA	NA
cDNA:S328502_2742	BT37	9.241	0.0003	NA	No	No	No	No	No	No	No	NA	NA	No	NA	NA	NA	NA
SalHit:S1416_3847	BT37	9.325	0.0324	none	No	No	No	No	No	No	No	0.0095625	0.87029851	Yes	VAR RED	Reduced	NA	yes
SalHit:S420806_11496	BT37	10.035	0.0037	NA	No	No	No	No	No	No	No	NA	NA	No	NA	NA	NA	NA
SalHit:S420806_8290	BT37	10.24	0.0141	NA	No	No	No	No	No	No	No	NA	NA	No	NA	NA	NA	NA
cDNA:S629902_2397	BT37	11.415	0.1133	none	No	No	No	No	No	No	No	0.038898305	0.83624322	Yes	VAR RED	Reduced	NA	yes
Gdist:S117158_3382	BT37	21.674	0.0506	NA	Yes	No	No	No	No	No	No	0.115471698	0.2295	Yes	NA	NA	NA	NA
cDNA:S318333_3219	BT37	21.699	0.0124	NA	No	No	No	No	No	No	No	NA	NA	No	NA	NA	NA	NA
cDNA:S318333_2480	BT37	22.121	0.0011	NA	No	No	No	No	No	No	No	NA	NA	No	NA	NA	NA	NA
cDNA:S113375_2115	BT37	27.946	0.0083	NA	Yes	No	No	No	No	No	No	NA	NA	No	NA	NA	NA	NA
cDNA:S113375_1244	BT37	30.285	0.0114	NA	No	No	No	No	No	No	No	NA	NA	No	NA	NA	NA	NA
Gdist:S6684_7743	BT37	33.602	0.0432	NA	No	No	No	No	No	No	No	0.823682994	0.88385252	Yes	NA	NA	NA	NA
Gdist:S125086_6731	BT37	33.625	0.028	NA	No	No	No	No	No	No	No	NA	NA	No	NA	NA	NA	NA
cDNA:S420821_4762	BT37	34.799	0.0157	NA	No	No	No	No	No	No	No	NA	NA	No	NA	NA	NA	NA
Gdist:S18537_5068	BT37	34.8	0.0137	NA	No	No	No	No	No	No	No	NA	NA	No	NA	NA	NA	NA
cDNA:S483389_655	BT37	40.551	0.0493	NA	No	No	No	No	No	No	No	0.471390845	0.91619469	Yes	NA	NA	NA	NA
SalHit:S407026_7260	BT37	40.552	-0.0047	NA	No	No	No	No	No	No	No	NA	NA	No	NA	NA	NA	NA
cDNA:S64181_3413	BT37	41.246	0.0272	NA	Yes	No	No	No	No	No	No	NA	NA	No	NA	NA	NA	NA
SalHit:S828444_1201	BT37	43.493	0.0257	NA	No	No	No	No	No	No	No	NA	NA	No	NA	NA	NA	NA
SalHit:S636915_947	BT37	43.592	0.0062	NA	No	No	No	No	No	No	No	NA	NA	No	NA	NA	NA	NA
Gene:S1971_885	BT37	44.351	0.0214	NA	No	No	No	No	No	No	No	NA	NA	No	NA	NA	NA	NA
Gdist:S79028_3191	BT37	45.333	0.025	NA	Yes	No	No	No	No	No	No	NA	NA	No	NA	NA	NA	NA
cDNA:S365376_1033	BT38	0	0.012	NA	No	No	No	No	No	Yes (0.022)	No	NA	NA	No	NA	NA	NA	NA
Gdist:S131730_2802	BT38	4.637	-0.0096	NA	No	No	No	No	No	No	No	NA	NA	No	NA	NA	NA	NA
Gdist:S370999_8329	BT38	4.757	0.0067	NA	No	No	No	No	No	No	No	NA	NA	No	NA	NA	NA	NA
Gdist:S7518_5696	BT38	10.059	0.0091	NA	No	No	No	No	No	No	No	NA	NA	No	NA	NA	NA	NA
LD:S224847_12342	BT38	10.304	0.0232	NA	No	No	No	No	No	No	No	0.14025	0.94321023	Yes	NA	NA	NA	NA
Gene:S240223_3885	BT38	10.618	0.0027	NA	Yes	No	No	No	No	No	No	NA	NA	No	NA	NA	NA	NA
Gdist:S50345_4157	BT38	10.979	0.0098	NA	No	No	No	No	No	No	No	NA	NA	No	NA	NA	NA	NA
Gdist:S521067_2486	BT38	10.979	-0.001	NA	No	No	No	No	No	Yes (0.004)	Yes (0.035)	NA	NA	No	NA	NA	NA	NA
Gdist:S74890_9507	BT38	14.405	-0.0001	NA	Yes	No	No	No	No	No	No	NA	NA	No	NA	NA	NA	NA
Gdist:S62465_8715	BT38	14.408	0.0186	NA	No	No	No	No	No	No	No	NA	NA	No	NA	NA	NA	NA
LD:S259174_1840	BT38	17.173	0.0297	NA	No	No	No	No	No	No	No	0.399743852	0.52356383	Yes	NA	NA	NA	NA
LD:S259174_14147	BT38	17.205	-0.0024	NA	No	No	No	No	No	No	No	NA	NA	No	NA	NA	NA	NA
Gdist:S142660_3589	BT38	17.207	-0.0107	NA	No	No	No	No	No	No	No	NA	NA	No	NA	NA	NA	NA
Gdist:S424284_8844	BT38	17.208	0.0041	NA	No	No	No	No	No	No	No	NA	NA	No	NA	NA	NA	NA
Gdist:S90602_2446	BT38	17.22	0.0234	NA	No	No	No	No	No	No	No	NA	NA	No	NA	NA	NA	NA
Gdist:S90602_5989	BT38	17.225	0.019	NA	No	No	No	No	No	No	No	NA	NA	No	NA	NA	NA	NA
Gdist:S64730_6850	BT38	17.444	-0.0153	NA	No	No	No	No	No	No	No	NA	NA	No	NA	NA	NA	NA
Gdist:S5274_14182	BT38	17.659	-0.0056	NA	No	No	No	No	No	No	No	NA	NA	No	NA	NA	NA	NA
Gdist:S170062_4086	BT38	17.873	-0.0026	NA	No	No	No	No	No	No	No	NA	NA	No	NA	NA	NA	NA
Gdist:S294436_5767	BT38	23.187	0.023	NA	No	No	No	No	No	No	No	NA	NA	No	NA	NA	NA	NA
Gdist:S294436_7649	BT38	23.187	0.001	NA	No	No	No	No	No	No	No	NA	NA	No	NA	NA	NA	NA
Gdist:S294436_4194	BT38	23.745	0.0118	NA	No	No	No	No	No	No	No	NA	NA	No	NA	NA	NA	NA
Gdist:S749541_5049	BT38	24.174	0.0133	NA	No	No	No	No	No	No	No	NA	NA	No	NA	NA	NA	NA
Gdist:S147058_566	BT38	25.122	0.0036	NA	No	No	No	No	No	No	No	NA	NA	No	NA	NA	NA	NA
cDNA:S156589_1653	BT38	26.391	-0.0007	NA	No	No	No	No	No	No	No	NA	NA	No	NA	NA	NA	NA
cDNA:S263501_1046	BT38	26.709	0.0132	NA	No	No	No	No	No	No	No	NA	NA	No	NA	NA	NA	NA

SalHit:S372965_4460	BT38	26.765	-0.0055	NA	No	No	No	No	No	No	No	NA	NA	No	NA	NA	NA	NA
cDNA:S125374_3570	BT38	32.433	0.0347	NA	No	No	No	No	No	No	No	0.907678571	0.83654676	Yes	NA	NA	NA	NA
Gdist:S26802_3445	BT38	33.684	0.0332	NA	No	No	No	No	No	No	No	0.9945	0.73333893	Yes	NA	NA	NA	NA
Gdist:S170623_5256	BT38	36.935	-0.0029	NA	No	No	No	No	No	No	No	NA	NA	No	NA	NA	NA	NA
Gdist:S137942_5888	BT38	37.137	0.0027	NA	No	No	No	No	No	No	No	NA	NA	No	NA	NA	NA	NA
Gdist:S277830_3921	BT38	37.673	0.0081	NA	No	No	No	No	No	No	No	0.217879747	0.80649408	Yes	NA	NA	NA	NA
Gdist:S258043_7017	BT38	37.941	0.026	NA	No	No	No	No	No	No	No	NA	NA	No	NA	NA	NA	NA
Gdist:S258043_9155	BT38	37.941	0.0155	NA	No	No	No	No	No	No	No	NA	NA	No	NA	NA	NA	NA
Gdist:S561164_2111	BT38	37.941	0.0118	NA	No	No	No	No	No	No	No	NA	NA	No	NA	NA	NA	NA
Gdist:S18950_668	BT38	37.941	0.0109	NA	No	No	No	No	No	No	No	NA	NA	No	NA	NA	NA	NA
Gdist:S92323_3399	BT38	37.941	0.0042	NA	No	No	No	No	No	No	No	NA	NA	No	NA	NA	NA	NA
Gdist:S258043_6425	BT38	37.941	-0.0002	NA	No	No	No	No	No	No	No	NA	NA	No	NA	NA	NA	NA
Gdist:S242633_5556	BT38	37.941	-0.0007	NA	No	No	No	No	No	No	No	NA	NA	No	NA	NA	NA	NA
Gdist:S158610_4775	BT38	37.944	0.0183	NA	No	No	No	No	No	No	No	NA	NA	No	NA	NA	NA	NA
cDNA:S411783_742	BT38	37.953	0.0088	NA	No	No	No	No	No	No	No	NA	NA	No	NA	NA	NA	NA
cDNA:C124634845_491	BT38	40.477	0.0158	NA	No	No	No	No	No	No	No	NA	NA	No	NA	NA	NA	NA
cDNA:S119796_361	BT38	40.99	-0.0023	NA	No	No	No	No	No	Yes (0.021)	No	NA	NA	No	NA	NA	NA	NA
cDNA:S504876_1571	BT38	41.093	0.0039	NA	No	No	No	No	No	Yes (0.035)	No	NA	NA	No	NA	NA	NA	NA
cDNA:S260722_894	BT38	41.111	0.0007	NA	No	No	No	No	No	Yes (0.021)	No	NA	NA	No	NA	NA	NA	NA
cDNA:S353226_1964	BT38	41.924	0.0102	NA	No	No	No	No	No	No	No	NA	NA	No	NA	NA	NA	NA
Gdist:S346042_1237	BT38	42.71	0.0242	NA	No	No	No	No	No	No	No	NA	NA	No	NA	NA	NA	NA
Gdist:S61195_134	BT38	42.71	0.024	NA	No	No	No	No	No	Yes (0.021)	No	NA	NA	No	NA	NA	NA	NA
Gdist:S498517_12927	BT38	42.71	0.023	NA	No	No	No	No	No	Yes (0.002)	Yes (0.024)	NA	NA	No	NA	NA	NA	NA
Gdist:S319155_3685	BT38	42.71	0.0094	NA	No	No	No	No	No	No	No	NA	NA	No	NA	NA	NA	NA
Gdist:S58919_3868	BT38	42.71	0.0053	NA	No	No	No	No	No	No	No	NA	NA	No	NA	NA	NA	NA
Gdist:S82886_3406	BT38	42.715	0.02	NA	Yes	No	No	No	No	No	No	NA	NA	No	NA	NA	NA	NA
cDNA:S512740_2933	BT38	42.72	-0.0044	NA	Yes	No	No	No	No	No	No	NA	NA	No	NA	NA	NA	NA
cDNA:S512740_4303	BT38	42.724	-0.0102	NA	No	No	No	No	No	No	No	NA	NA	No	NA	NA	NA	NA
Gdist:S305584_12370	BT38	44.23	0.0149	NA	No	No	No	No	No	No	No	NA	NA	No	NA	NA	NA	NA
cDNA:S331108_1684	BT38	44.618	-0.0111	NA	No	No	No	No	No	Yes (0.001)	Yes (0.011)	NA	NA	No	NA	NA	NA	NA
SalHit:S227800_14465	BT38	44.999	0.0276	NA	No	No	No	No	No	No	No	NA	NA	No	NA	NA	NA	NA
cDNA:S8184_724	BT38	44.999	0.0135	NA	No	No	No	No	No	Yes (0.043)	No	NA	NA	No	NA	NA	NA	NA
SalHit:S129621_2871	BT38	44.999	0.0124	NA	No	No	No	No	No	No	No	NA	NA	No	NA	NA	NA	NA
Gdist:S738908_4158	BT38	44.999	0.0001	NA	No	No	No	No	No	No	No	NA	NA	No	NA	NA	NA	NA
SalHit:S227800_4162	BT38	44.999	-0.0039	NA	No	No	No	No	No	Yes (0.014)	No	NA	NA	No	NA	NA	NA	NA
SalHit:S227800_3402	BT38	44.999	-0.005	NA	No	No	No	No	No	No	No	NA	NA	No	NA	NA	NA	NA
cDNA:S331108_2743	BT38	44.999	-0.0055	NA	No	No	No	No	No	No	No	NA	NA	No	NA	NA	NA	NA
Gdist:S59908_10924	BT38	45.427	0.0035	NA	No	No	No	No	No	No	No	NA	NA	No	NA	NA	NA	NA
Gdist:S214130_1540	BT38	45.593	0.0238	NA	No	No	No	No	No	No	No	NA	NA	No	NA	NA	NA	NA
Gdist:S251409_6260	BT38	45.856	0.0434	NA	No	No	No	No	No	No	No	0.7171875	0.39962687	Yes	NA	NA	NA	NA
cDNA:S322137_1124	BT38	45.856	0.0337	NA	No	No	No	No	No	No	No	0.139695652	0.88165329	Yes	NA	NA	NA	NA
Gdist:S59410_6978	BT38	45.856	-0.0014	NA	No	No	No	No	No	No	No	NA	NA	No	NA	NA	NA	NA
Gdist:S130140_5833	BT38	45.88	0.0306	NA	No	No	No	No	No	No	No	0.963553748	0.59723684	Yes	NA	NA	NA	NA
Gdist:S79262_8781	BT38	45.905	0.0207	NA	No	No	No	No	No	No	No	NA	NA	No	NA	NA	NA	NA
SalHit:S202609_547	BT38	47.338	0.0489	NA	No	No	No	No	No	Yes (0)	Yes (0.009)	0.926563433	0.84566784	Yes	NA	NA	NA	NA
SalHit:S681882_3013	BT38	47.364	0.0295	NA	No	No	No	No	No	Yes (0.021)	No	NA	NA	No	NA	NA	NA	NA
cDNA:S566907_1654	BT38	47.365	0.043	NA	No	No	No	No	No	No	No	0.982691275	0.57891892	Yes	NA	NA	NA	NA
SalHit:S681882_2890	BT38	47.365	0.0337	NA	No	No	No	No	No	No	No	0.742859281	0.923625	Yes	NA	NA	NA	NA
SalHit:S34679_800	BT38	47.365	0.0304	NA	No	No	No	No	No	No	No	0.608301527	0.1275	Yes	NA	NA	NA	NA
SalHit:S34679_17551	BT38	47.365	0.0236	NA	No	No	No	No	No	No	No	NA	NA	No	NA	NA	NA	NA
Gdist:S135888_8430	BT38	47.365	0.0147	NA	No	No	No	No	No	No	No	NA	NA	No	NA	NA	NA	NA
SalarSNP:ESTNV_36533_796	BT38	47.365	0.0144	NA	No	No	No	No	No	No	No	NA	NA	No	NA	NA	NA	NA
cDNA:S566907_6021	BT38	47.365	0.0055	NA	Yes	No	No	No	No	No	No	NA	NA	No	NA	NA	NA	NA
Gdist:S424236_2957	BT38	47.365	0.0053	NA	No	No	No	No	No	No	No	NA	NA	No	NA	NA	NA	NA
Gdist:S12863_5738	BT38	47.365	0.0013	NA	No	No	No	No	No	No	No	NA	NA	No	NA	NA	NA	NA
Gdist:S365106_1019	BT38	47.365	-0.0064	NA	No	No	No	No	No	Yes (0.004)	Yes (0.035)	NA	NA	No	NA	NA	NA	NA
Gdist:S307090_446	BT38	47.365	-0.0096	NA	No	No	No	No	No	No	No	NA	NA	No	NA	NA	NA	NA
cDNA:S566907_1991	BT38	47.365	0.0712	NA	Yes	no	no	No	No	No	No	NA	NA	no	NA	NA	NA	NA
Gdist:S456240_7264	BT38	47.403	0.0123	NA	No	No	No	No	No	No	No	NA	NA	No	NA	NA	NA	NA
cDNA:S194405_4648	BT38	47.444	0.0095	NA	Yes	No	No	No	No	No	No	NA	NA	No	NA	NA	NA	NA
Gdist:S471566_203	BT38	50.612	0.0011	NA	No	No	No	No	No	No	No	NA	NA	No	NA	NA	NA	NA
Gene:S363330_3277	BT38	50.612	-0.0075	NA	Yes	No	No	No	No	No	No	NA	NA	No	NA	NA	NA	NA
Gdist:S160823_4331	BT38	51.005	0.0117	NA	No	No	No	No	No	No	No	NA	NA	No	NA	NA	NA	NA
Gdist:S126223_591	BT38	51.479	0.021	NA	No	No	No	No	No	No	No	NA	NA	No	NA	NA	NA	NA
Gdist:S594074_9347	BT38	51.504	0.0584	NA	No	No	No	No	No	No	No	0.893131188	0.3565678	Yes	NA	NA	NA	NA
Gdist:S626117_1457	BT38	51.53	0.0371	NA	No	No	No	No	No	No	No	0.838743243	0.65440981	Yes	NA	NA	NA	NA
Gdist:S342053_9320	BT38	51.53	0.0203	NA	Yes	No	No	No	No	No	No	NA	NA	No	NA	NA	NA	NA
SalHit:S262166_3433	BT38	51.53	0.0121	NA	No	No	No	No	No	No	No	NA	NA	No	NA	NA	NA	NA
cDNA:S44770_8034	BT38	51.53	0.0117	NA	No	No	No	No	No	No	No	NA	NA	No	NA	NA	NA	NA
Gdist:S342053_7249	BT38	51.53	0.0047	NA	No	No	No	No	No	No	No	NA	NA	No	NA	NA	NA	NA
Gdist:S47588_4768	BT38	51.53	-0.012	NA	No	No	No	No	No	Yes (0.048)	No	NA	NA	No	NA	NA	NA	NA
cDNA:S635018_516	BT38	51.927	0.0225	NA	No	No	No	No	No	No	No	NA	NA	No	NA	NA	NA	NA
SalHit:S56217_940	BT38	52.34	0.0123	NA	No	No	No	No	No	No	No	NA	NA	No	NA	NA	NA	NA
SalHit:S602336_6773	BT38	52.34	-0.0001	NA	No	No	No	No	No	No	No	NA	NA	No	NA	NA	NA	NA
Gene:S445579_467	BT38	52.34	-0.002	NA	No	No	No	No	No	No	No	NA	NA	No	NA	NA	NA	NA
cDNA:S755450_1408	BT38	52.36	-0.004	NA	Yes	No	No	No	No	No	No	NA	NA	No	NA	NA	NA	NA
Gdist:S51312_7330	BT38	52.61	0.0138	NA	No	No	No	No	No	No	No	NA	NA	No	NA	NA	NA	NA
Gdist:S51312_7711	BT38	52.725	0.0233	NA	No	No	No	No	No	No	No	NA	NA	No	NA	NA	NA	NA
Gdist:S146062_1439	BT38	55.002	-0.0123	NA	No	No	No	No	No	No	No	NA	NA	No	NA	NA	NA	NA
cDNA:C124697205_473	BT38	55.004	-0.0034	NA	No	No	No	No	No	No	No	NA	NA	No	NA	NA	NA	NA
Gdist:S181325_6555	BT38	55.005	0.0185	NA	No	No	No	No	No	No	No	NA	NA	No	NA	NA	NA	NA
cDNA:S559237_2085	BT38	55.005	0.0067	NA	Yes	No	No	No	No	No	No	NA	NA	No	NA	NA	NA	NA
cDNA:S427444_2796	BT38	55.007	0.0273	NA	No	No	No	No	No	No	No	NA	NA	No	NA	NA	NA	NA
cDNA:S220818_892	BT38	55.645	0.0109	NA	No	No	No	No	No	No	No	NA	NA	No	NA	NA	NA	NA
SalHit:S627974_2057	BT39	0	0.0233	NA	No	No	No	No	No	No	No	NA	NA	No	NA	NA	NA	NA
Gdist:S353767_9113	BT39	0	0.0175	NA	No	No	No	No	No	No	No	NA	NA	No	NA	NA	NA	NA
Gdist:S108825_7124	BT39	0.082	0.0625	NA	No	No	No	No	No	No	No	0	0.46026971	Yes	VAR INC	increased	NA	yes
SalHit:S18885_1674	BT39	0.234	0.0399	NA	No	No	No	No	No	No	No	0.8925	0.90708767	Yes	NA	NA	NA	NA
Gdist:S391711_1723	BT39	7.579	0.037	NA	No	No	No	No	No	Yes (0.003)	Yes (0.03)	0.988505976	0.23395631	Yes	NA	NA	NA	NA
Gdist:S391711_1307	BT39	7.584	0.0103	NA	No	No	No	No	No	No	No	NA	NA	No	NA	NA	NA	NA
Gdist:S6450_2371	BT39	23.083	0.0072	NA	No	No	No	No	No	No	No	NA	NA	No	NA	NA	NA	NA
Gdist:S150354_8511	BT39	23.226	0.0473	NA	No	No	No	No	No	No	No	0.655587209	0.81392442	Yes	NA	NA	NA	NA
Gdist:S150354_5018	BT39	23.226	0.0238	NA	No	No	No	No	No	No	No	NA	NA	No	NA	NA	NA	NA
Gene:S460136_553	BT39	23.226	0.0067	NA	Yes	No	No	No	No	Yes (0.013)	No	NA	NA	No	NA	NA	NA	NA
Gdist:S96522_1841	BT39	23.226	0.0051	NA	No	No	No	No	No	No	No	NA	NA	No	NA	NA	NA	NA
Gdist:S558937_4668	BT39	23.226	0.005	NA	No	No	No	No	No	No	No	NA	NA	No	NA	NA	NA	NA
Gdist:S193527_292	BT39	23.233	-0.0019	NA	No	No	No	No	No	Yes (0)	Yes (0.009)	NA	NA	No	NA	NA	NA	NA
Gdist:S58860_5003	BT39	23.24	-0.0112	NA	No	No	No	No	No	No	No	NA	NA	No	NA	NA	NA	NA
Gdist:S20598_970	BT39	23.444	0.0081	NA	No	No	No	No	No	No	No	NA	NA	No	NA	NA	NA	NA
Gdist:S151794_4079	BT39	26.017	0.0157	NA	No	No	No	No	No	No	No	0.299431818	0.87029851	Yes	NA	NA	NA	NA
cDNA:C124481658_275	BT39	26.194	-0.0112	NA	No	No	No	No	No	No	No	NA	NA	No	NA	NA	NA	NA
Gdist:S316856_7845	BT39	35.599	-0.002	NA	No	No	No	No	No	No	No	NA	NA	No	NA	NA	NA	NA
cDNA:S183974_5330	BT39	42.121	0.0271	NA	No	No	No	No	No	No	No	NA	NA	No	NA	NA	NA	NA
cDNA:S562620_2065	BT39	42.121	0.0249	NA	No	No	No	No	No	No	No	NA	NA	No	NA	NA	NA	NA
Gdist:S114106_7048	BT39	42.121	0.0173	NA	No	No	No	No	No	No	No	NA	NA	No	NA	NA	NA	NA
cDNA:S562620_1139	BT39	42.121	0.0054	NA	No	No	No	No	No	No	No	NA	NA	No	NA	NA	NA	NA
Gdist:S114106_2022	BT39	42.121	-0.0025	NA	No	No	No	No	No	No	No	NA	NA	No	NA	NA	NA	NA
Gdist:S307891_8631	BT39	42.121	0.1037	none	Yes	Yes	Yes	No	No	No	No	0.574795082	0.83863636	Yes	Outlier Varde &	Outlier Var	NA	yes
Gdist:S46966_11604	BT39	44.21	0.0378	NA	No	No	No	No	No	No	No	0.221944444	0.81337062	Yes	NA	NA	NA	NA
Gdist:S46966_9492	BT39	44.211	-0.0008	NA	No	No	No	No	No	No	No	NA	NA	No	NA	NA	NA	NA
Gdist:S246010_10785	BT39	44.217	0.0105	NA	No	No	No	No	No	No	No	0.053873239	0.48116071	Yes	NA	NA	NA	NA
Gdist:S203203_4789	BT39	44.291	-0.0099	NA	No	No	No	No	No	No	No	NA	NA	No	NA	NA	NA	NA
Gdist:S245865_2638	BT39	44.372	-0.0092	NA	No	No	No	No	No	No	No	NA	NA	No	NA	NA	NA	NA
Gdist:S245865_7630	BT39	44.45	0.0213	none	No	No	No	No	No	No	No	0	0.70376232	Yes	VAR INC + Outl	Outlier Var	NA	yes
Gdist:S92880_6893	BT39	44.533	0.0117	NA	No	No	No	No	No	No	No	NA	NA	No	NA	NA	NA	NA
cDNA:S80986_5698	BT39	44.546	-0.0121	NA	No	No	No	No	No	No	No	NA	NA	No	NA	NA	NA	NA
Gdist:S163404_8754	BT39	46.081	0.0014	NA	No	No	No	No	No	No	No	NA	NA	No	NA	NA	NA	NA
Gdist:S287518_5794	BT39	46.175	0.0223	NA	No	No	No	No	No	No	No	NA	NA	No	NA	NA	NA	NA
Gdist:S525898_1297	BT39	47.921	-0.0121	NA	No	No	No	No	No	No	No	NA	NA	No	NA	NA	NA	NA
SalarSNP:ESTNV_37086_867	BT39	47.923	0.0094	NA	No	No	No	No	No	No	No	NA	NA	No	NA	NA	NA	NA
Gdist:S138607_4627	BT39	48.877	-0.0064	NA	No	No	No	No	No	No	No	NA	NA	No	NA	NA	NA	NA
Gdist:S241572_3967	BT39	48.888	0.0031	NA	No	No	No	No	No	Yes (0.021)	No	NA	NA	No	NA	NA	NA	NA
Gdist:S54043_5022	BT39	52.657	-0.0034	NA	No	No	No	No	No	No	No	NA	NA	No	NA	NA	NA	NA
SalHit:S19196_949	BT39	54.783	-0.0013	NA	Yes	No	No	No	No	No	No	NA	NA	No	NA	NA	NA	NA
Gdist:S490061_6098	BT39	56.34	0.0207	NA	No	No	No	No	No	No	No	0.837857143	0.92678416	Yes	NA	NA	NA	NA
Gdist:S159056_4732	BT39	58.301	0.0146	NA	No	No	No	No	No	No	No	NA	NA	No	NA	NA	NA	NA
Gdist:S159056_11023	BT39	58.302	-0.0024	NA	No	No	No	No	No	No	No	NA	NA	No	NA	NA	NA	NA

Gdist:S42631_3308	BT39	59.35	0.0183	NA	No	No	No	No	No	No	No	0.982691275	0.98752681	Yes	NA	NA	NA	NA
Gdist:S123827_11252	BT39	59.541	0.0008	NA	No	No	No	No	No	No	No	NA	NA	No	NA	NA	NA	NA
Gdist:S540262_7580	BT39	59.731	0.0024	NA	No	No	No	No	No	No	No	NA	NA	No	NA	NA	NA	NA
Gdist:S8097_6080	BT39	59.735	0.0024	NA	No	No	No	No	No	No	No	NA	NA	No	NA	NA	NA	NA
Gdist:S5512_7078	BT39	59.739	0.0081	NA	No	No	No	No	No	No	No	NA	NA	No	NA	NA	NA	NA
cDNA:S14268_488	BT39	59.742	0.0016	NA	No	No	No	No	No	No	No	NA	NA	No	NA	NA	NA	NA
cDNA:S203527_1959	BT39	60.021	-0.0022	NA	No	No	No	No	No	No	No	NA	NA	No	NA	NA	NA	NA
Gdist:S198740_10984	BT39	61.759	0.0166	NA	No	No	No	No	No	No	No	NA	NA	No	NA	NA	NA	NA
cDNA:S417833_884	BT39	61.759	0.0132	NA	No	No	No	No	No	No	No	NA	NA	No	NA	NA	NA	NA
Gdist:S218976_658	BT39	61.759	0.0002	NA	No	No	No	No	No	No	No	NA	NA	No	NA	NA	NA	NA
Gdist:S357248_9754	BT39	61.759	-0.0006	NA	No	No	No	No	No	No	No	NA	NA	No	NA	NA	NA	NA
Gdist:S58971_14711	BT39	62.466	0.006	NA	No	No	No	No	No	No	No	NA	NA	No	NA	NA	NA	NA
Gdist:S389074_7997	BT39	62.466	0.0026	NA	No	No	No	No	No	No	No	NA	NA	No	NA	NA	NA	NA
cDNA:S655629_4062	BT39	62.534	-0.0056	NA	No	No	No	No	No	No	No	NA	NA	No	NA	NA	NA	NA
Gdist:S424263_9861	BT39	63.633	0.012	NA	No	No	No	No	No	No	No	NA	NA	No	NA	NA	NA	NA
Gdist:S441834_7882	BT39	64.666	0.0589	NA	No	No	No	No	No	No	No	0.233502907	0.33346154	Yes	NA	NA	NA	NA
Gdist:S441834_13052	BT39	64.669	0.0282	NA	No	No	No	No	No	No	No	NA	NA	No	NA	NA	NA	NA
Gdist:S151287_12936	BT39	64.699	0.019	NA	No	No	No	No	No	No	No	NA	NA	No	NA	NA	NA	NA
Gdist:S63101_5477	BT39	66.058	0.0036	NA	No	No	No	No	No	No	No	NA	NA	No	NA	NA	NA	NA
Gdist:S19339_4724	BT39	66.06	-0.0017	NA	No	No	No	No	No	No	No	NA	NA	No	NA	NA	NA	NA
Gdist:S388247_1797	BT39	66.062	0.0096	NA	No	No	No	No	No	No	No	NA	NA	No	NA	NA	NA	NA
LD:S56055_2429	BT39	66.062	0.0083	NA	No	No	No	No	No	No	No	NA	NA	No	NA	NA	NA	NA
Gdist:S189316_5901	BT39	66.062	-0.0103	NA	No	No	No	No	No	No	No	NA	NA	No	NA	NA	NA	NA
Gdist:S194107_4544	BT39	66.839	0.0328	NA	No	No	No	No	No	No	No	0.565105932	0.37633065	Yes	NA	NA	NA	NA
cDNA:S538039_7455	BT39	67.796	-0.0154	NA	No	No	No	No	No	No	No	NA	NA	No	NA	NA	NA	NA
cDNA:S248614_2597	BT39	67.896	0.0153	NA	No	No	No	No	No	No	No	NA	NA	No	NA	NA	NA	NA
Gdist:S95941_2611	BT39	68.577	0.0482	NA	Yes	No	No	No	No	No	No	0.184370504	0.48116071	Yes	NA	NA	NA	NA
SalHit:S253158_3206	BT39	68.578	0.0151	NA	No	No	No	No	No	No	No	NA	NA	No	NA	NA	NA	NA
Gdist:S315516_8915	BT39	68.578	0.0127	NA	No	No	No	No	No	No	No	NA	NA	No	NA	NA	NA	NA
Gdist:S319960_7431	BT39	70.936	0.0417	NA	No	No	No	No	No	No	No	0.254299451	0.074375	Yes	NA	NA	NA	NA
Gdist:S378362_8127	BT39	70.937	0.0778	NA	No	No	No	No	No	No	No	0.062943038	0.84672504	Yes	NA	NA	NA	NA
Gdist:S113214_1620	BT39	70.937	0.0153	NA	No	No	No	No	No	No	No	NA	NA	No	NA	NA	NA	NA
SalHit:S48341_1363	BT39	71.167	0.0191	NA	No	No	No	No	No	No	No	NA	NA	No	NA	NA	NA	NA
Gdist:S216407_3667	BT40	0	0.0015	NA	No	No	No	No	No	No	No	NA	NA	No	NA	NA	NA	NA
Gdist:S375902_4837	BT40	0.022	0.0313	NA	No	No	No	No	No	No	No	0.442817308	0.800955	Yes	NA	NA	NA	NA
cDNA:S2645_1067	BT40	0.022	0.0091	NA	No	No	No	No	No	No	No	NA	NA	No	NA	NA	NA	NA
Gdist:S193269_2840	BT40	0.022	-0.0059	NA	No	No	No	No	No	Yes (0.021)	No	NA	NA	No	NA	NA	NA	NA
SalHit:S65799_1161	BT40	0.49	0.0066	NA	No	No	No	No	No	No	No	NA	NA	No	NA	NA	NA	NA
cDNA:S517417_2310	BT40	0.492	0.0054	NA	No	No	No	No	No	Yes (0.037)	No	NA	NA	No	NA	NA	NA	NA
Gdist:S112993_2878	BT40	6.671	0.0032	NA	No	No	No	No	No	No	No	NA	NA	No	NA	NA	NA	NA
cDNA:S174408_2524	BT40	6.842	0.0138	NA	No	No	No	No	No	Yes (0.037)	No	NA	NA	No	NA	NA	NA	NA
Gdist:S120982_3932	BT40	6.894	0.0245	NA	No	No	No	Yes (0.038)	No	No	No	NA	NA	No	NA	NA	NA	NA
SalHit:C124762720_287	BT40	8.597	0.0345	NA	No	No	No	No	No	No	No	0.9945	0.89727171	Yes	NA	NA	NA	NA
SalHit:S127117_1306	BT40	9.831	0.0132	NA	No	No	No	No	No	No	No	NA	NA	No	NA	NA	NA	NA
Gdist:S303872_6279	BT40	12.564	0.0341	NA	No	No	No	No	No	No	No	0.871576923	0.56791921	Yes	NA	NA	NA	NA
Gdist:S71018_1428	BT40	14.031	0.013	NA	No	No	No	No	No	No	No	NA	NA	No	NA	NA	NA	NA
Gene:S172375_1029	BT40	17.325	0.0046	NA	No	No	No	No	No	No	No	NA	NA	No	NA	NA	NA	NA
Gdist:S67424_8050	BT40	17.325	-0.0072	NA	No	No	No	No	No	No	No	NA	NA	No	NA	NA	NA	NA
Gdist:S151488_906	BT40	17.34	0.0037	NA	No	No	No	No	No	No	No	NA	NA	No	NA	NA	NA	NA
cDNA:S163450_973	BT40	20.039	-0.0017	NA	No	No	No	No	No	No	No	NA	NA	No	NA	NA	NA	NA
SalHit:S113858_5503	BT40	22.442	-0.0039	NA	No	No	No	No	No	No	No	NA	NA	No	NA	NA	NA	NA
Gdist:S239095_3364	BT40	22.499	-0.0053	NA	No	No	No	No	No	No	No	NA	NA	No	NA	NA	NA	NA
cDNA:S36325_2195	BT40	22.499	-0.0063	NA	No	No	No	No	No	No	No	NA	NA	No	NA	NA	NA	NA
Gdist:S239095_8204	BT40	22.507	-0.0051	NA	No	No	No	No	No	No	No	NA	NA	No	NA	NA	NA	NA
Gdist:S99112_5418	BT40	33.205	-0.0028	NA	No	No	No	No	No	No	No	NA	NA	No	NA	NA	NA	NA
Gdist:S529969_6952	BT40	34.816	0.0117	NA	No	No	No	No	No	No	No	NA	NA	No	NA	NA	NA	NA
Gdist:S29350_7487	BT40	34.86	0.0049	NA	No	No	No	No	No	No	No	NA	NA	No	NA	NA	NA	NA
Gdist:S1807_5747	BT40	34.862	-0.0017	NA	No	No	No	No	No	No	No	NA	NA	No	NA	NA	NA	NA
Gdist:S1807_2321	BT40	34.862	-0.0126	NA	No	No	No	No	No	No	No	NA	NA	No	NA	NA	NA	NA
Gdist:S190289_2144	BT40	35.556	0.0052	NA	No	No	No	No	No	Yes (0.043)	No	NA	NA	No	NA	NA	NA	NA
Gdist:S36082_17231	BT40	36.042	0.0295	NA	No	No	No	No	No	No	No	0.51786526	0.78238636	Yes	NA	NA	NA	NA
cDNA:S91962_7567	BT40	36.473	0.0112	NA	No	No	No	No	No	No	No	NA	NA	No	NA	NA	NA	NA
Gdist:S361402_1207	BT40	37.299	-0.0053	NA	No	No	No	No	No	No	No	NA	NA	No	NA	NA	NA	NA
cDNA:S103981_1041	BT40	42.945	-0.0095	NA	No	No	No	No	No	No	No	NA	NA	No	NA	NA	NA	NA
Gdist:S521613_1456	BT40	45.702	-0.0035	NA	No	No	No	No	No	No	No	NA	NA	No	NA	NA	NA	NA
Gdist:S187369_5006	BT40	45.944	0.004	NA	No	No	No	No	No	No	No	NA	NA	No	NA	NA	NA	NA
cDNA:S576685_1623	BT40	46.186	0.0002	NA	Yes	No	No	No	No	No	No	NA	NA	No	NA	NA	NA	NA
cDNA:S576685_1429	BT40	46.388	0.0034	NA	Yes	No	No	No	No	No	No	NA	NA	No	NA	NA	NA	NA
Gdist:S3254_2158	BT40	46.393	0.0065	NA	No	No	No	No	No	No	No	NA	NA	No	NA	NA	NA	NA
SalarSNP:GCR_cBin34489_Ctg	BT40	46.395	0.0062	NA	No	No	No	No	No	No	No	NA	NA	No	NA	NA	NA	NA
Gdist:S26847_11703	BT40	46.395	-0.0026	NA	No	No	No	No	No	No	No	NA	NA	No	NA	NA	NA	NA
cDNA:S246877_5300	BT40	46.396	0.0764	NA	No	No	No	No	No	No	No	0.504742268	0.46026971	Yes	NA	NA	NA	NA
cDNA:S246877_5804	BT40	46.396	0.0764	NA	No	No	No	No	No	No	No	0.233502907	0.46026971	Yes	NA	NA	NA	NA
Gdist:S53105_9491	BT40	46.396	0.0115	NA	No	No	No	No	No	No	No	NA	NA	No	NA	NA	NA	NA
Gdist:S293746_313	BT40	51.728	0	NA	No	No	No	No	No	No	No	NA	NA	No	NA	NA	NA	NA
Gdist:S90746_1139	BT40	53.641	0.0313	NA	No	No	No	No	No	No	No	0.469308511	0.9833502	Yes	NA	NA	NA	NA
Gdist:S461442_9572	BT40	55.433	-0.0038	NA	No	No	No	No	No	No	No	NA	NA	No	NA	NA	NA	NA
Gdist:S461442_4554	BT40	55.442	-0.0016	NA	No	No	No	No	No	No	No	NA	NA	No	NA	NA	NA	NA
Gdist:S454896_9545	BT40	55.452	0.0321	NA	Yes	No	No	No	No	No	No	0.988505976	0.78533742	Yes	NA	NA	NA	NA
Gdist:S7933_3998	BT40	55.452	0.0149	NA	No	No	No	No	No	No	No	NA	NA	No	NA	NA	NA	NA
Gdist:S34277_5375	BT40	55.452	0.0144	NA	No	No	No	No	No	No	No	NA	NA	No	NA	NA	NA	NA
Gdist:S147812_7826	BT40	55.452	0.0124	NA	No	No	No	No	No	Yes (0.001)	Yes (0.014)	NA	NA	No	NA	NA	NA	NA
cDNA:S464216_6837	BT40	55.452	0.011	NA	No	No	No	No	No	Yes (0)	Yes (0.005)	NA	NA	No	NA	NA	NA	NA
Gdist:S475716_8639	BT40	55.452	0.0089	NA	No	No	No	No	No	Yes (0.013)	No	NA	NA	No	NA	NA	NA	NA
SalHit:S113308_970	BT40	56.92	0.0411	NA	No	No	No	No	No	Yes (0.013)	No	0.495931034	0.33346154	Yes	NA	NA	NA	NA
Gdist:S297801_1473	BT40	57.618	0.0166	NA	No	No	No	No	No	No	No	NA	NA	No	NA	NA	NA	NA
cDNA:S123529_5079	BT40	57.857	-0.0059	NA	No	No	No	No	No	No	No	NA	NA	No	NA	NA	NA	NA
cDNA:S598943_992	BT40	58.095	-0.0083	NA	No	No	No	No	No	Yes (0.023)	No	NA	NA	No	NA	NA	NA	NA
Gdist:S401177_3694	BT40	58.097	0.0029	NA	Yes	No	No	No	No	No	No	NA	NA	No	NA	NA	NA	NA
Gdist:S40469_3688	BT40	58.099	0.009	NA	No	No	No	No	No	No	No	NA	NA	No	NA	NA	NA	NA
SalHit:S563209_3485	BT40	58.102	0.0529	NA	No	No	No	No	No	No	No	0.367597403	0.53701987	Yes	NA	NA	NA	NA
cDNA:S5174_7473	BT40	58.104	0.0454	NA	No	No	No	No	No	No	No	0.805187617	0.39962687	Yes	NA	NA	NA	NA
cDNA:S69030_5885	BT40	58.105	0.0021	NA	No	No	No	No	No	No	No	NA	NA	No	NA	NA	NA	NA
cDNA:S398633_712	BT40	59.471	0.0069	NA	Yes	No	No	No	No	No	No	NA	NA	No	NA	NA	NA	NA
Gdist:S145778_3425	BT40	59.575	0.0273	NA	No	No	No	No	No	No	No	NA	NA	No	NA	NA	NA	NA
Gdist:S76060_6063	BT40	59.575	-0.0065	NA	No	No	No	No	No	No	No	NA	NA	No	NA	NA	NA	NA
LD:S83132_9406	BT40	59.576	0.0123	NA	Yes	No	No	No	No	No	No	NA	NA	No	NA	NA	NA	NA
LD:S83132_634	BT40	59.576	-0.0038	NA	No	No	No	No	No	No	No	NA	NA	No	NA	NA	NA	NA
Gdist:S267347_5574	BT40	60.218	-0.0021	NA	Yes	No	No	Yes (0.001) (*288	Yes (0) (*288 SNI	No	No	NA	NA	No	NA	NA	NA	NA
Gdist:S581396_3933	BT40	60.268	0.0238	NA	No	No	No	No	No	No	No	NA	NA	No	NA	NA	NA	NA
SalHit:S95866_2343	BT40	60.319	0.0318	NA	No	No	No	No	No	No	No	0.916490895	0.87794046	Yes	NA	NA	NA	NA
Gdist:S448304_5774	BT40	60.366	0.0622	NA	No	No	No	No	No	Yes (0.023)	No	0.530792308	0.77468354	Yes	NA	NA	NA	NA
Gdist:S113363_8211	BT40	60.366	0.0222	NA	No	No	No	No	No	No	No	NA	NA	No	NA	NA	NA	NA
SalHit:S337125_1011	BT40	60.366	0.019	NA	No	No	No	No	No	No	No	NA	NA	No	NA	NA	NA	NA
Gdist:S74445_2466	BT40	60.366	0.0164	NA	Yes	No	No	No	No	No	No	NA	NA	No	NA	NA	NA	NA
Gdist:S213275_6315	BT40	60.366	0.0061	NA	No	No	No	No	No	No	No	NA	NA	No	NA	NA	NA	NA
Gdist:S213275_5909	BT40	60.366	0.0023	NA	No	No	No	No	No	No	No	NA	NA	No	NA	NA	NA	NA
SalHit:S466797_6775	BT40	60.366	-0.0017	NA	No	No	No	No	No	No	No	NA	NA	No	NA	NA	NA	NA
cDNA:S552157_3420	BT40	60.447	0.007	NA	No	No	No	No	No	No	No	NA	NA	No	NA	NA	NA	NA
cDNA:S106622_3244	BT40	60.52	0.007	NA	No	No	No	No	No	No	No	NA	NA	No	NA	NA	NA	NA
cDNA:S106622_14447	BT40	60.592	0.0107	NA	No	No	No	No	No	Yes (0.002)	Yes (0.024)	NA	NA	No	NA	NA	NA	NA
cDNA:S225167_3381	BT40	60.668	0.048	NA	Yes	No	No	No	No	Yes (0.002)	Yes (0.024)	0.992172619	0.87608911	Yes	NA	NA	NA	NA
Gdist:S137969_2061	BT40	60.734	-0.0061	NA	No	No	No	No	No	No	No	NA	NA	No	NA	NA	NA	NA
cDNA:S55582_1063	BT40	60.865	0.0019	NA	Yes	No	No	No	No	Yes (0.015)	No	NA	NA	No	NA	NA	NA	NA
Gdist:S22470_352	BT40	60.923	-0.004	NA	No	No	No	No	No	No	No	NA	NA	No	NA	NA	NA	NA
Gdist:S387818_6438	BT40	60.977	0.0321	NA	No	No	No	No	No	No	No	0.926563433	0.87869952	Yes	NA	NA	NA	NA
cDNA:S580446_422	BT40	60.977	0.031	NA	No	No	No	No	No	No	No	0.204	0.89481818	Yes	NA	NA	NA	NA
SalHit:S116298_7643	BT40	60.977	0.0171	NA	No	No	No	No	No	No	No	NA	NA	No	NA	NA	NA	NA
Gdist:S85637_8012	BT40	60.977	0.0166	NA	No	No	No	No	No	No	No	NA	NA	No	NA	NA	NA	NA
Gdist:S122960_5739	BT40	60.977	0.0146	NA	Yes	No	No	No	No	No	No	NA	NA	No	NA	NA	NA	NA
Gdist:S7806_9395	BT40	60.977	0.0108	NA	No	No	No	No	No	No	No	NA	NA	No	NA	NA	NA	NA
Gdist:S666501_4977	BT40	60.977	0.0087	NA	No	No	No	No	No	No	No	NA	NA	No	NA	NA	NA	NA
SalHit:S318001_4665	BT40	60.977	0.0081	NA	No	No	No	No	No	No	No	NA	NA	No	NA	NA	NA	NA
Gdist:S243104_6653	BT40	60.977	0.0018	NA	No	No	No	No	No	No	No	NA	NA	No	NA	NA	NA	NA
Gdist:S302602_6741	BT40	60.978	-0.009	NA	No	No	No	No	No	No	No	NA	NA	No	NA	NA	NA	NA
Gene:S123339_1421	BT40	61.024	0.0134	NA	Yes	No	No	No	No	No	No	NA	NA	No	NA	NA	NA	NA

SNP ID	chi^2	df	Pr(chi^2 >)	FDR adjusted P value
cDNA:C122548872_148	0.700923	1	0.402473675	0.996192022
cDNA:C122898371_146	1.557749	1	0.211995095	0.968970438
cDNA:C123299019_115	0.720895	1	0.39585048	1
cDNA:C123300059_91	0.322232	1	0.57026917	1
cDNA:C123447604_140	2.038815	1	0.15332903	0.961439446
cDNA:C123587298_320	1.501769	1	0.220399307	0.966711643
cDNA:C123610732_215	0.685991	1	0.407531199	1
cDNA:C123789935_234	2.84959	1	0.091397237	0.890762712
cDNA:C123791721_258	3.108853	1	0.077867764	0.837628032
cDNA:C123994563_51	0.275047	1	0.599966362	1
cDNA:C124068305_390	0.285416	1	0.593173237	1
cDNA:C124072625_365	0.787682	1	0.374802034	1
cDNA:C124193644_487	0.428664	1	0.512645146	1
cDNA:C124205688_219	0.512054	1	0.474251062	1
cDNA:C124241706_353	1.542354	1	0.214267706	0.959095066
cDNA:C124250102_412	0.002741	1	0.958247068	1
cDNA:C124302365_425	0.002341	1	0.961411507	1
cDNA:C124413300_569	1.568014	1	0.210495719	0.968970438
cDNA:C124481658_275	0.289584	1	0.590487433	1
cDNA:C124495874_565	0.093345	1	0.759966618	1
cDNA:C124528172_494	0.111775	1	0.738132577	1
cDNA:C124634845_491	1.803314	1	0.179312307	0.948045845
cDNA:C124635139_172	2.287689	1	0.130403958	0.957294737
cDNA:C124640749_257	0.019975	1	0.887608047	1
cDNA:C124692505_310	1.212114	1	0.270913842	0.97046332
cDNA:C124697205_473	1.171128	1	0.279169634	1
cDNA:C124725291_143	2.143433	1	0.143181151	0.961678135
cDNA:C124745537_293	0.039593	1	0.842279151	1
cDNA:C124745537_734	0.387432	1	0.533652362	1
cDNA:C124754657_212	1.796646	1	0.180118561	0.959095066
cDNA:S100450_763	2.906761	1	0.088208868	0.826214689
cDNA:S10072_4210	2.58E-06	1	0.998717533	1
cDNA:S100721_1974	2.906128	1	0.088243514	0.881646018
cDNA:S100723_472	1.553352	1	0.212641309	0.967531453
cDNA:S102161_2082	8.287017	1	0.003992954	0.574514286
cDNA:S102161_6750	2.674481	1	0.10196855	0.890957983
cDNA:S10356_987	0.585063	1	0.444334666	1
cDNA:S10364_858	0.003992	1	0.949623813	1
cDNA:S10378_3626	0.207001	1	0.649128223	1
cDNA:S103981_1041	0.000837	1	0.976913946	1
cDNA:S104446_3884	1.462894	1	0.226470246	0.98872956
cDNA:S104710_1557	0.604863	1	0.436728665	1
cDNA:S104710_1633	0.010412	1	0.918726328	1
cDNA:S104710_3393	0.279856	1	0.596795436	1
cDNA:S105063_643	0.876829	1	0.349071683	1
cDNA:S105534_2802	0.296812	1	0.585888104	1
cDNA:S105897_2400	7.240714	1	0.007126862	0.373061224

cDNA:S106622_14447	3.432667	1	0.063918794	0.873196429
cDNA:S106622_3244	0.191469	1	0.661696483	1
cDNA:S106918_2860	0.328116	1	0.566770293	1
cDNA:S109135_5389	1.260482	1	0.261559888	0.996192022
cDNA:S109135_8210	0.857028	1	0.354571708	1
cDNA:S110517_4456	0.217817	1	0.640708297	1
cDNA:S110598_4394	0.308669	1	0.578497817	1
cDNA:S111663_3454	0.205909	1	0.649992779	1
cDNA:S111702_1407	0.072837	1	0.787249575	1
cDNA:S111749_3373	0.009081	1	0.924080386	1
cDNA:S113096_14352	2.762775	1	0.096480746	0.890957983
cDNA:S113375_1244	0.013308	1	0.908159213	1
cDNA:S113375_2115	0.069463	1	0.792120502	1
cDNA:S113427_14338	2.097863	1	0.147505162	0.930719512
cDNA:S113427_4484	9.994972	1	0.001569683	0.152333333
cDNA:S11384_4796	0.040603	1	0.840306059	1
cDNA:S114312_248	0.002469	1	0.96036726	1
cDNA:S115738_2816	0.755481	1	0.384746456	1
cDNA:S115958_1426	0.134165	1	0.714152045	1
cDNA:S116047_1442	2.497293	1	0.114042205	0.896898129
cDNA:S117233_945	0.322232	1	0.57026917	1
cDNA:S118058_4136	4.198208	1	0.040466718	0.738065728
cDNA:S118429_1535	1.312549	1	0.251933651	0.959095066
cDNA:S118429_3382	0.198616	1	0.655840341	1
cDNA:S118434_5718	0.46302	1	0.496215914	1
cDNA:S118837_6577	0.019822	1	0.888035531	1
cDNA:S119796_361	2.049426	1	0.152263602	0.930719512
cDNA:S120620_363	3.001813	1	0.083171392	0.886540773
cDNA:S12068_2890	2.520723	1	0.112359054	0.923049505
cDNA:S12068_320	0.731754	1	0.392315383	1
cDNA:S121981_5646	0.00751	1	0.930942049	1
cDNA:S122885_4254	0.496461	1	0.481059221	1
cDNA:S123137_914	1.601123	1	0.205744124	0.959095066
cDNA:S123351_2073	0.034407	1	0.852843527	1
cDNA:S123351_5681	0.157706	1	0.691277637	1
cDNA:S123529_5079	4.704013	1	0.03009228	0.915683241
cDNA:S124403_4920	0.484421	1	0.486426601	1
cDNA:S124423_2529	0.111775	1	0.738132577	1
cDNA:S124728_2350	0.299403	1	0.584256653	1
cDNA:S125095_4124	1.527141	1	0.216541964	0.968970438
cDNA:S125374_3570	2.587204	1	0.107730329	0.923049505
cDNA:S125992_655	4.193071	1	0.040589498	0.787240876
cDNA:S126541_3351	4.196204	1	0.040514586	0.727469388
cDNA:S12713_1968	3.519975	1	0.060633355	0.787240876
cDNA:S12713_7023	0.067066	1	0.795657143	1
cDNA:S127270_2739	0.604863	1	0.436728665	1
cDNA:S127517_4651	0.869453	1	0.351106676	0.996192022
cDNA:S128086_5877	1.695252	1	0.192910082	0.972155745

cDNA:S128101_345	0.069463	1	0.792120502	1
cDNA:S128955_9570	1.799832	1	0.179732785	0.960075029
cDNA:S129171_513	0.083696	1	0.77234977	1
cDNA:S1296_2391	0.023154	1	0.879057895	1
cDNA:S130162_702	0.640695	1	0.423459059	1
cDNA:S130317_7021	0.059263	1	0.807663992	1
cDNA:S130742_2245	1.704775	1	0.191664653	0.961678135
cDNA:S132167_1829	2.184097	1	0.139442792	0.930719512
cDNA:S133384_1630	3.035273	1	0.081472837	0.873196429
cDNA:S133525_2346	0.365006	1	0.545739273	1
cDNA:S133625_906	0.124822	1	0.723862117	1
cDNA:S134413_3941	0.162512	1	0.686853827	1
cDNA:S134565_1451	0.006491	1	0.935785299	1
cDNA:S136795_3145	0.552293	1	0.457382229	1
cDNA:S137691_2198	0.05752	1	0.810458789	1
cDNA:S13770_1805	0.106631	1	0.744013313	1
cDNA:S1382_3653	0.101318	1	0.750253328	1
cDNA:S139461_1842	2.186017	1	0.139269062	0.923049505
cDNA:S139611_3301	1.34337	1	0.246440601	0.959095066
cDNA:S140351_5419	5.880161	1	0.015312425	0.669071895
cDNA:S141016_2579	0.041504	1	0.838568924	1
cDNA:S14268_488	8.78086	1	0.003044077	0.243733333
cDNA:S142821_2593	0.534271	1	0.464816621	1
cDNA:S143080_508	0.524603	1	0.468884852	1
cDNA:S14394_194	0.065933	1	0.797353309	1
cDNA:S14549_908	1.125237	1	0.288793539	0.992751958
cDNA:S146372_1828	3.406717	1	0.064931492	0.819448276
cDNA:S147850_1094	0.012173	1	0.912146246	1
cDNA:S148838_2039	6.74E-05	1	0.99344982	1
cDNA:S149017_7696	0.994823	1	0.318566465	0.992434326
cDNA:S149017_8051	5.392601	1	0.020222308	0.632769231
cDNA:S14978_1947	0.163582	1	0.685879835	1
cDNA:S150131_2338	0.706112	1	0.400737493	1
cDNA:S150356_541	0.697739	1	0.403544339	1
cDNA:S150416_2053	0.006068	1	0.937907968	1
cDNA:S150821_5852	0.030044	1	0.862390852	1
cDNA:S150821_9294	0.013473	1	0.907593396	1
cDNA:S151018_2140	0.40947	1	0.522238848	1
cDNA:S151018_3636	0.733789	1	0.391657798	1
cDNA:S151021_283	3.163544	1	0.075299554	0.826214689
cDNA:S151742_11162	0.024876	1	0.874676086	1
cDNA:S153293_2845	0.008442	1	0.926792028	1
cDNA:S154283_1102	0.409914	1	0.522013326	1
cDNA:S154301_764	0.147488	1	0.70094804	1
cDNA:S155659_1271	0.228679	1	0.632504783	1
cDNA:S156230_1049	1.810471	1	0.178451738	0.936024096
cDNA:S156230_8577	0.00239	1	0.961007388	1
cDNA:S156589_1653	0.00283	1	0.957577477	1

cDNA:S15772_1853	1.557749	1	0.211995095	0.961439446
cDNA:S159289_3695	1.58941	1	0.207410421	0.97046332
cDNA:S160324_5645	0.35667	1	0.550361646	1
cDNA:S160349_4078	8.050478	1	0.004549164	0.58496
cDNA:S160993_8558	1.292438	1	0.255599094	0.968970438
cDNA:S160993_9051	0.047313	1	0.827806545	1
cDNA:S161866_1891	0.134499	1	0.713811947	1
cDNA:S162418_5551	0.001082	1	0.973764239	1
cDNA:S162820_857	0.445614	1	0.50442514	1
cDNA:S163213_185	0.257989	1	0.611505071	1
cDNA:S163450_973	1.40406	1	0.236044954	0.974933333
cDNA:S16389_833	0.253728	1	0.614462357	1
cDNA:S164085_5376	5.094007	1	0.024008659	0.669071895
cDNA:S164228_5818	3.132173	1	0.076761351	0.90096945
cDNA:S164512_3249	2.276868	1	0.131316811	0.910498084
cDNA:S165871_3361	1.18547	1	0.276245199	0.992751958
cDNA:S166130_1937	0.845186	1	0.357917702	1
cDNA:S166813_3437	0.552293	1	0.457382229	1
cDNA:S167751_4478	1.559733	1	0.211704347	0.957294737
cDNA:S167866_4323	0.363478	1	0.546581201	1
cDNA:S168148_15564	1.386949	1	0.238920999	0.992751958
cDNA:S168405_2082	1.159819	1	0.281503168	0.98872956
cDNA:S168405_3804	1.880248	1	0.170305921	0.923049505
cDNA:S169037_3948	0.28754	1	0.591801434	1
cDNA:S169620_2932	0.275646	1	0.599569217	1
cDNA:S170395_4015	0.020404	1	0.88641571	1
cDNA:S170983_2252	0.139401	1	0.708876868	1
cDNA:S171492_2300	0.005503	1	0.940864504	1
cDNA:S171993_2472	0.052826	1	0.818217156	1
cDNA:S172462_1007	0.0149	1	0.90284676	1
cDNA:S173199_1673	1.292438	1	0.255599094	0.968970438
cDNA:S174408_2524	0.039237	1	0.842979669	1
cDNA:S175617_6065	1.650256	1	0.198924138	0.996192022
cDNA:S176944_17346	4.520155	1	0.033497793	0.738065728
cDNA:S177192_3587	0.094363	1	0.758702666	1
cDNA:S177192_4107	1.980684	1	0.159318392	0.948045845
cDNA:S177784_5558	0.008688	1	0.925738358	1
cDNA:S177852_1371	1.573083	1	0.209759792	0.966711643
cDNA:S178550_6364	16.23573	1	5.59E-05	0.152333333
cDNA:S180637_703	1.43297	1	0.231280285	0.959095066
cDNA:S183231_554	0.360036	1	0.548486172	1
cDNA:S183371_1071	2.402664	1	0.121128821	0.910498084
cDNA:S183974_5330	0.489492	1	0.484153801	1
cDNA:S184823_3889	0.682209	1	0.408826991	1
cDNA:S184823_7256	0.117015	1	0.732294869	1
cDNA:S184852_493	5.906456	1	0.015085493	0.595162791
cDNA:S184986_7445	0.031291	1	0.859593153	1
cDNA:S18529_1354	1.302641	1	0.253731357	0.972155745

cDNA:S185419_3086	0.117653	1	0.731594504	1
cDNA:S185779_2734	1.390543	1	0.238313377	0.972155745
cDNA:S18670_5330	0.654756	1	0.418417435	1
cDNA:S186934_1562	0.003084	1	0.955713602	1
cDNA:S187071_1047	0.599443	1	0.438790814	1
cDNA:S187829_3326	0.540664	1	0.462157622	1
cDNA:S187829_864	0.015435	1	0.901128131	1
cDNA:S188721_1253	2.506065	1	0.113408794	0.873196429
cDNA:S189184_774	0.00029	1	0.986406698	1
cDNA:S191954_2250	0.11936	1	0.729729282	1
cDNA:S192149_1046	0.671888	1	0.412394183	1
cDNA:S192515_5942	0.147605	1	0.700834717	1
cDNA:S194179_14559	1.171128	1	0.279169634	1
cDNA:S194405_4648	1.512903	1	0.218696605	0.972666047
cDNA:S194666_1674	0.679239	1	0.40984901	1
cDNA:S194904_542	0.025432	1	0.873296404	1
cDNA:S195837_3240	4.515972	1	0.033579807	0.677037037
cDNA:S196052_8001	0.154544	1	0.694230013	1
cDNA:S196323_2857	1.565073	1	0.21092392	0.961678135
cDNA:S196928_1342	1.871158	1	0.17134256	0.959095066
cDNA:S19762_6844	0.007416	1	0.931372413	1
cDNA:S198718_8135	0.594814	1	0.440563428	0.996192022
cDNA:S198876_8759	0.445614	1	0.50442514	1
cDNA:S198906_868	0.038474	1	0.844495343	1
cDNA:S199676_7836	0.010495	1	0.918403494	1
cDNA:S200492_3963	1.671971	1	0.19599472	0.961439446
cDNA:S20157_2917	27.11251	1	1.92E-07	0.332363636
cDNA:S201621_1382	1.070658	1	0.300796911	0.996192022
cDNA:S201721_1936	0.222148	1	0.637408312	1
cDNA:S201721_4183	0.157577	1	0.691397319	1
cDNA:S202817_2591	0.406258	1	0.52387473	1
cDNA:S203107_4811	1.292438	1	0.255599094	0.972115607
cDNA:S203527_1959	3.57926	1	0.058505193	0.826214689
cDNA:S20419_2654	0.537313	1	0.463548265	1
cDNA:S204896_3399	0.060155	1	0.806251823	1
cDNA:S206043_3642	1.142575	1	0.285108878	0.996192022
cDNA:S20680_1709	0.629564	1	0.427515198	1
cDNA:S207226_5551	3.233714	1	0.072136914	0.826214689
cDNA:S208_4966	2.890624	1	0.089096429	0.829512605
cDNA:S208336_5062	0.0759	1	0.782932195	1
cDNA:S208909_3903	0.351442	1	0.553297877	1
cDNA:S20998_14238	2.170633	1	0.140668319	0.936024096
cDNA:S20998_4758	2.657358	1	0.103071787	0.888665245
cDNA:S211903_5325	1.679974	1	0.194927951	0.961439446
cDNA:S212066_3949	0.041504	1	0.838568924	1
cDNA:S212652_2299	0.004242	1	0.948071253	1
cDNA:S21271_1377	0.768315	1	0.380738801	0.996867696
cDNA:S213304_2056	0.061524	1	0.80410269	1

cDNA:S21404_1309	0.051898	1	0.819793441	1
cDNA:S216045_7099	0.060155	1	0.806251823	1
cDNA:S216045_7664	0.399311	1	0.527445409	1
cDNA:S21692_2011	0.908874	1	0.340413251	0.996192022
cDNA:S217532_351	4.633223	1	0.0313586	0.712451282
cDNA:S21788_1182	0.007637	1	0.930359681	1
cDNA:S218110_1857	0.538711	1	0.462967477	1
cDNA:S218110_2808	0.545751	1	0.460059194	1
cDNA:S219140_556	2.092669	1	0.148007255	0.930719512
cDNA:S219947_3820	2.205394	1	0.137528763	0.930719512
cDNA:S220067_3688	0.006633	1	0.935087032	1
cDNA:S220818_892	0.028005	1	0.867097143	1
cDNA:S220841_14465	0.089898	1	0.764307005	1
cDNA:S222300_2367	0.024211	1	0.876349478	1
cDNA:S223180_922	0.336095	1	0.562092272	1
cDNA:S224362_842	0.285879	1	0.592873744	1
cDNA:S225132_7775	0.112816	1	0.736960669	1
cDNA:S225167_3381	0.038474	1	0.844495343	1
cDNA:S225247_1244	0.102625	1	0.748701748	1
cDNA:S2274_506	0.508928	1	0.475603097	1
cDNA:S2274_967	1.080373	1	0.298614153	1
cDNA:S227479_670	1.95231	1	0.162338116	0.957294737
cDNA:S227505_6398	1.33E-05	1	0.997089594	1
cDNA:S228436_3377	5.397684	1	0.020163496	0.632769231
cDNA:S228964_6518	0.150737	1	0.697831937	1
cDNA:S229093_237	5.127778	1	0.02354585	0.669071895
cDNA:S231999_1945	0.726051	1	0.394166183	1
cDNA:S232646_1656	1.171337	1	0.27912663	0.972666047
cDNA:S232968_4649	2.887228	1	0.089284429	0.845861183
cDNA:S233433_1455	0.022871	1	0.879792108	1
cDNA:S234748_925	0.604863	1	0.436728665	1
cDNA:S235071_667	1.71092	1	0.190866045	0.959095066
cDNA:S236680_5590	11.57757	1	0.000667521	0
cDNA:S238020_10690	1.443064	1	0.229644154	0.961678135
cDNA:S238798_3838	0.001615	1	0.967940846	1
cDNA:S239119_2637	1.026634	1	0.310950672	1
cDNA:S239551_1419	0.325041	1	0.568593548	1
cDNA:S239832_569	5.609923	1	0.017859047	0.632769231
cDNA:S242162_9689	1.838621	1	0.175112713	0.961678135
cDNA:S242245_7699	1.153524	1	0.282812628	1
cDNA:S242248_1019	0.091114	1	0.762765125	1
cDNA:S242248_1277	0.132224	1	0.716137659	1
cDNA:S243280_5038	0.980208	1	0.32214749	0.996192022
cDNA:S243791_4937	0.071714	1	0.788857135	1
cDNA:S245573_4771	3.583958	1	0.058339994	0.787240876
cDNA:S246052_6786	0.81677	1	0.366126218	1
cDNA:S246877_5300	3.031886	1	0.081643069	0.870908189
cDNA:S246877_5804	4.376163	1	0.03644495	0.826214689

cDNA:S247343_843	6.100257	1	0.013516226	0.669071895
cDNA:S248003_6551	3.159434	1	0.075489333	0.831364384
cDNA:S248614_2597	3.240677	1	0.071830953	0.849373737
cDNA:S249113_11946	0.052385	1	0.818964768	1
cDNA:S249178_1898	0.74534	1	0.387955647	1
cDNA:S249523_196	1.045313	1	0.306588952	0.996192022
cDNA:S249908_4794	2.504393	1	0.113529205	0.910498084
cDNA:S249908_9625	0.36646	1	0.544940002	1
cDNA:S250827_279	0.926605	1	0.33574667	0.996192022
cDNA:S251134_1830	1.00583	1	0.315903958	0.996192022
cDNA:S251955_427	3.824887	1	0.050496764	0.829512605
cDNA:S252705_4671	1.281462	1	0.257627341	0.972666047
cDNA:S252705_5472	0.061524	1	0.80410269	1
cDNA:S252719_505	0.439144	1	0.507535886	1
cDNA:S252802_1574	0.150737	1	0.697831937	1
cDNA:S253487_1033	0.686915	1	0.407215528	1
cDNA:S253487_2550	6.72E-05	1	0.9934587	1
cDNA:S255038_318	0.538711	1	0.462967477	1
cDNA:S255040_2501	0.239762	1	0.624377824	1
cDNA:S255526_428	3.186595	1	0.074244526	0.873196429
cDNA:S255641_2125	0.388101	1	0.533299106	1
cDNA:S255992_1631	0.0067	1	0.93476319	1
cDNA:S256656_7787	0.27081	1	0.602788701	1
cDNA:S257231_2778	2.288758	1	0.130314134	0.923049505
cDNA:S257543_2471	1.693678	1	0.193116835	0.961439446
cDNA:S258718_3516	0.453035	1	0.500897409	1
cDNA:S258718_7888	0.435962	1	0.509077818	1
cDNA:S25899_3079	1.445095	1	0.22931656	0.968970438
cDNA:S260469_1182	0.819592	1	0.365299461	1
cDNA:S260722_894	0.77674	1	0.378140062	1
cDNA:S261458_2796	1.200344	1	0.273252836	0.968970438
cDNA:S262570_3483	1.917625	1	0.166118705	0.957294737
cDNA:S262853_2386	1.12688	1	0.288441808	1
cDNA:S26296_3384	1.617097	1	0.203497029	0.959095066
cDNA:S263051_694	0.00964	1	0.921786892	1
cDNA:S263179_1819	2.08782	1	0.148477822	0.949004255
cDNA:S263501_1046	0.057746	1	0.810093922	1
cDNA:S2645_1067	0.996733	1	0.3181024	0.991787234
cDNA:S265325_1830	0.351442	1	0.553297877	1
cDNA:S266225_1746	0.256891	1	0.61226397	1
cDNA:S267203_6159	2.340397	1	0.126057412	0.90850501
cDNA:S268348_2100	1.674933	1	0.195599091	0.957294737
cDNA:S268496_4507	0.011432	1	0.914850443	1
cDNA:S270982_6549	0.640695	1	0.423459059	0.996192022
cDNA:S27306_4435	2.370988	1	0.123608801	0.910498084
cDNA:S273477_6161	0.588867	1	0.442857505	1
cDNA:S273477_6465	0.419974	1	0.516949801	1
cDNA:S273631_2250	0.945236	1	0.330934786	0.983407136

cDNA:S273631_3441	0.000841	1	0.976858967	1
cDNA:S27464_3079	0.048616	1	0.825488538	1
cDNA:S27464_5751	0.696468	1	0.403972929	1
cDNA:S275534_2354	0.423669	1	0.515111937	1
cDNA:S27567_4317	1.802688	1	0.179387842	0.961678135
cDNA:S27567_6436	0.093937	1	0.759230622	1
cDNA:S277533_383	1.062359	1	0.30267775	0.996192022
cDNA:S277616_2134	0.739386	1	0.389857707	1
cDNA:S277744_4747	0.82374	1	0.364089027	0.996192022
cDNA:S279651_4775	0.406258	1	0.52387473	1
cDNA:S279973_7852	0.687247	1	0.407102475	0.996192022
cDNA:S280660_1114	0.086219	1	0.76904005	1
cDNA:S280691_409	1.694019	1	0.193072029	0.959095066
cDNA:S280750_4106	0.239725	1	0.624405116	1
cDNA:S282124_239	0.341897	1	0.558736431	1
cDNA:S282715_14917	0.106244	1	0.744461687	1
cDNA:S282882_3216	4.785003	1	0.028708598	0.695312883
cDNA:S283239_5129	0.761991	1	0.382706294	0.996192022
cDNA:S283503_224	1.326617	1	0.249407964	0.964
cDNA:S285965_1544	2.072041	1	0.150020665	0.948045845
cDNA:S286429_654	3.583958	1	0.058339994	0.826214689
cDNA:S286516_1913	7.364207	1	0.006653495	0.628989247
cDNA:S287314_2130	0.387432	1	0.533652362	1
cDNA:S28762_5847	0.093993	1	0.759161373	1
cDNA:S290555_1498	0.880287	1	0.348123191	0.996192022
cDNA:S290599_2915	2.04E-05	1	0.996396603	1
cDNA:S291008_2693	0.005056	1	0.943311493	1
cDNA:S291008_3424	0.453698	1	0.500584052	1
cDNA:S291123_361	4.500339	1	0.033888134	0.707612903
cDNA:S292910_1208	0.450724	1	0.501991427	1
cDNA:S293277_438	0.228679	1	0.632504783	1
cDNA:S293527_2798	1.152387	1	0.283049995	0.996192022
cDNA:S293652_756	0.114681	1	0.734876915	1
cDNA:S293894_13016	0.27545	1	0.599698983	1
cDNA:S293894_14384	0.637521	1	0.424610077	1
cDNA:S295692_1789	0.833254	1	0.361333361	0.996192022
cDNA:S295997_808	0.089898	1	0.764307005	1
cDNA:S297048_3936	0.136026	1	0.712264306	1
cDNA:S297454_5449	0.828041	1	0.362839698	0.996192022
cDNA:S297454_6571	0.121962	1	0.726915831	1
cDNA:S2985_4670	0.755481	1	0.384746456	1
cDNA:S298750_5277	2.291891	1	0.130051358	0.914
cDNA:S299605_718	1.820373	1	0.177268889	0.957294737
cDNA:S300070_3495	0.029445	1	0.863755537	1
cDNA:S30059_2725	0.000742	1	0.978267909	1
cDNA:S301357_9739	1.805408	1	0.179060083	0.930719512
cDNA:S301968_735	1.703848	1	0.191785477	0.996192022
cDNA:S302429_3381	0.245567	1	0.620213669	1

cDNA:S304352_1007	1.369144	1	0.241958963	0.968970438
cDNA:S304515_4041	0.012173	1	0.912146246	1
cDNA:S304833_1946	1.99025	1	0.158314731	0.937295352
cDNA:S304943_12655	1.172612	1	0.278865202	0.972666047
cDNA:S306803_1347	0.029567	1	0.86347532	1
cDNA:S307643_3137	0.913377	1	0.339220084	0.996192022
cDNA:S307975_3938	7.432193	1	0.006406723	0.595162791
cDNA:S309020_2087	0.329578	1	0.565907486	1
cDNA:S309375_5264	6.878279	1	0.008724954	0.669071895
cDNA:S309721_1176	5.547942	1	0.018502433	0.669071895
cDNA:S310740_4879	0.065933	1	0.797353309	1
cDNA:S310740_5108	1.337574	1	0.247462314	0.968970438
cDNA:S310740_8498	0.076983	1	0.781428971	1
cDNA:S311358_11177	0.319289	1	0.57203518	1
cDNA:S311598_624	1.389312	1	0.238521321	1
cDNA:S312955_1490	0.059701	1	0.806968367	1
cDNA:S31330_3843	0.315082	1	0.574578159	1
cDNA:S314116_482	1.196064	1	0.274109829	0.996192022
cDNA:S315299_565	0.628759	1	0.427810788	1
cDNA:S315457_3367	0.876829	1	0.349071683	1
cDNA:S315457_6106	0.242658	1	0.622293028	1
cDNA:S315619_2265	0.358834	1	0.549154697	1
cDNA:S316629_667	2.308654	1	0.128655419	0.923049505
cDNA:S318333_2480	5.657974	1	0.017376188	0.373061224
cDNA:S318333_3219	0.567602	1	0.451213324	0.996192022
cDNA:S318858_10221	4.639435	1	0.031245282	0.712451282
cDNA:S321236_5259	0.764986	1	0.381772723	1
cDNA:S322137_1124	0.337289	1	0.561398064	1
cDNA:S322654_3410	0.110302	1	0.739800409	1
cDNA:S322930_3152	1.650256	1	0.198924138	0.996192022
cDNA:S323771_3746	1.137276	1	0.28622869	0.973350649
cDNA:S323771_6228	0.80111	1	0.370761754	0.996192022
cDNA:S324619_4826	3.547543	1	0.059633698	0.823205298
cDNA:S325177_848	0.003759	1	0.951109536	1
cDNA:S325743_4384	1.043539	1	0.306999756	0.996192022
cDNA:S325743_5924	6.416082	1	0.011309142	0.628989247
cDNA:S326435_12628	0.335616	1	0.562370721	1
cDNA:S327027_2206	0.880287	1	0.348123191	0.996192022
cDNA:S328188_1390	0.059084	1	0.807949034	1
cDNA:S328502_2742	2.079391	1	0.149299781	0.94994382
cDNA:S331069_333	0.298122	1	0.585061954	1
cDNA:S331108_1684	1.251976	1	0.263175421	0.968970438
cDNA:S331108_2743	0.000392	1	0.984213615	1
cDNA:S331455_6435	3.114992	1	0.077574869	0.870908189
cDNA:S33235_315	0.121076	1	0.727870101	1
cDNA:S332936_3224	2.904306	1	0.088343246	0.849373737
cDNA:S333249_277	3.403438	1	0.065060671	0.873196429
cDNA:S333649_178	0.20129	1	0.653681577	1

cDNA:S335445_3045	0.360106	1	0.548447496	1
cDNA:S336443_13972	0.175914	1	0.674908846	1
cDNA:S336489_1653	0.437618	1	0.508274257	1
cDNA:S33700_8300	0.751595	1	0.385971578	1
cDNA:S337092_5433	0.0144	1	0.904483119	1
cDNA:S337711_8313	1.43705	1	0.230617313	0.996192022
cDNA:S3392_1022	0.033804	1	0.85412445	1
cDNA:S340707_312	0.111259	1	0.738715776	1
cDNA:S342230_358	1.689637	1	0.193648822	0.959095066
cDNA:S342230_989	0.647942	1	0.420849361	1
cDNA:S343808_2594	0.135583	1	0.712711825	1
cDNA:S345393_606	3.159434	1	0.075489333	0.873196429
cDNA:S345860_1024	0.135787	1	0.712505809	1
cDNA:S345968_9777	0.102319	1	0.749064403	1
cDNA:S346048_5673	0.00751	1	0.930942049	1
cDNA:S347134_7224	4.110592	1	0.042615444	0.823205298
cDNA:S347511_3755	5.278555	1	0.021589653	0.595162791
cDNA:S347572_3504	0.633721	1	0.425993721	1
cDNA:S347938_221	3.71E-06	1	0.99846338	1
cDNA:S349065_3669	3.163544	1	0.075299554	0.826214689
cDNA:S35007_1727	0.490614	1	0.48365372	1
cDNA:S350970_3409	5.55E-05	1	0.994054356	1
cDNA:S35126_3369	0.72152	1	0.395645736	0.996192022
cDNA:S353186_3074	0.007895	1	0.929199613	1
cDNA:S353226_1964	2.667412	1	0.10242248	0.912272212
cDNA:S355742_2202	0.039878	1	0.841718881	1
cDNA:S357219_769	1.925799	1	0.165218773	0.930719512
cDNA:S357292_1178	1.24902	1	0.26373976	0.961678135
cDNA:S358699_7137	1.119053	1	0.290122559	0.996192022
cDNA:S358873_5176	0.192775	1	0.660617288	1
cDNA:S360008_1889	1.011249	1	0.314603847	0.996192022
cDNA:S36111_15008	4.113805	1	0.042534573	0.787240876
cDNA:S36111_2504	0.025387	1	0.873406972	1
cDNA:S361636_1084	5.359804	1	0.020606095	0.741308756
cDNA:S361636_6984	0.216047	1	0.642068659	1
cDNA:S361757_4124	0.000929	1	0.975679869	1
cDNA:S36325_2195	2.000098	1	0.157289041	0.923049505
cDNA:S363339_747	0.360106	1	0.548447496	1
cDNA:S363461_6741	0.055641	1	0.813523118	1
cDNA:S363566_1471	1.942023	1	0.163449001	0.957294737
cDNA:S363729_1681	1.169038	1	0.279598941	0.983407136
cDNA:S363729_4252	0.350768	1	0.553678537	1
cDNA:S364146_769	4.68742	1	0.030384232	0.706961326
cDNA:S36442_6427	0.279286	1	0.597169888	1
cDNA:S365376_1033	0.968737	1	0.324995424	0.996192022
cDNA:S365959_394	0.886251	1	0.346495377	0.996192022
cDNA:S366188_990	0.37966	1	0.537785018	1
cDNA:S366279_1007	0.001059	1	0.974044128	1

cDNA:S36670_3719	2.124502	1	0.144959875	0.90096945
cDNA:S366861_1180	5.576161	1	0.018206592	0.628989247
cDNA:S367995_3467	0.784837	1	0.375666017	1
cDNA:S36850_3607	0.227732	1	0.633210806	1
cDNA:S36850_6296	1.497944	1	0.220988019	0.961678135
cDNA:S368796_2495	1.299127	1	0.254372712	0.968970438
cDNA:S370001_11500	0.12141	1	0.727510432	1
cDNA:S370001_9436	4.639435	1	0.031245282	0.669071895
cDNA:S370810_3013	5.051414	1	0.024605847	0.734784314
cDNA:S371791_1792	0.540664	1	0.462157622	1
cDNA:S37230_3170	0.094076	1	0.759058069	1
cDNA:S373049_4395	0.137638	1	0.71064095	1
cDNA:S373842_3964	0.164823	1	0.684754764	1
cDNA:S374302_1853	1.08262	1	0.298112284	0.968970438
cDNA:S374382_1018	0.930002	1	0.334862324	0.999094441
cDNA:S376368_5043	0.126439	1	0.722153293	1
cDNA:S377538_2760	0.064688	1	0.799234168	1
cDNA:S37782_4794	0.453698	1	0.500584052	1
cDNA:S3786_11163	0.055898	1	0.813100871	1
cDNA:S378656_7609	0.7787	1	0.377538949	0.996192022
cDNA:S378656_8483	0.010495	1	0.918403494	1
cDNA:S379086_6064	0.746218	1	0.387676437	1
cDNA:S384021_4373	0.181682	1	0.669931395	1
cDNA:S384781_3281	0.059736	1	0.806913245	1
cDNA:S38574_1326	1.298996	1	0.254396726	0.996192022
cDNA:S38713_4675	3.46991	1	0.062494717	0.826214689
cDNA:S387575_1543	5.314672	1	0.021146579	0.632769231
cDNA:S388728_2949	0.429507	1	0.51223103	1
cDNA:S38989_1707	0.078163	1	0.779803117	1
cDNA:S390597_2423	0.184104	1	0.667870035	1
cDNA:S390903_1871	0.607218	1	0.435837124	1
cDNA:S396796_272	0.263666	1	0.607612983	1
cDNA:S397066_4260	0.003085	1	0.955703596	1
cDNA:S398101_2128	1.025769	1	0.311154523	0.996192022
cDNA:S398633_712	0.327433	1	0.567174635	1
cDNA:S398768_3382	0.796425	1	0.372164248	0.996192022
cDNA:S399531_1848	0.645997	1	0.421547454	1
cDNA:S402468_6204	0.066153	1	0.797023262	1
cDNA:S404147_2649	0.639876	1	0.423755797	1
cDNA:S404555_12194	1.497864	1	0.221000388	0.960075029
cDNA:S404555_7877	1.348939	1	0.245463621	0.960075029
cDNA:S404751_3670	0.012918	1	0.909509227	1
cDNA:S405104_1587	0.032505	1	0.856923399	1
cDNA:S405104_7335	0.416593	1	0.518642087	1
cDNA:S405223_686	0.616112	1	0.432495691	1
cDNA:S407073_2825	0.00849	1	0.926584577	1
cDNA:S40801_3776	0.743594	1	0.388512044	1
cDNA:S409144_10758	1.359153	1	0.243684293	0.968970438

cDNA:S409465_1832	4.029495	1	0.04471131	0.78448583
cDNA:S409498_652	1.385456	1	0.23917393	0.996192022
cDNA:S410039_1043	0.017498	1	0.894763368	1
cDNA:S41119_1719	0.352228	1	0.552854589	1
cDNA:S411373_345	4.959936	1	0.025941161	0.706961326
cDNA:S411783_742	0.010495	1	0.918403494	1
cDNA:S412123_7384	1.394425	1	0.237659274	0.968970438
cDNA:S41242_10981	0.956693	1	0.328021579	0.996192022
cDNA:S412895_1278	0.275047	1	0.599966362	1
cDNA:S414296_4127	0.874662	1	0.34966782	0.996192022
cDNA:S414350_1592	0.220084	1	0.638975545	1
cDNA:S415421_1829	0.167862	1	0.682018681	1
cDNA:S415600_5145	1.005187	1	0.316058673	0.996192022
cDNA:S415600_773	1.004369	1	0.316255575	0.996192022
cDNA:S415746_2653	0.002007	1	0.964269798	1
cDNA:S416873_399	0.452616	1	0.501095207	1
cDNA:S417833_884	0.003179	1	0.955037387	1
cDNA:S418182_2800	0.054283	1	0.815771383	1
cDNA:S41920_882	1.287384	1	0.256530522	0.968970438
cDNA:S420548_5277	0.400792	1	0.526680677	1
cDNA:S420821_4762	6.343583	1	0.011780669	0.595162791
cDNA:S421584_3494	0.687247	1	0.407102475	0.999094441
cDNA:S42272_2039	0.36196	1	0.547419559	1
cDNA:S422982_9383	0.607218	1	0.435837124	1
cDNA:S423457_1288	0.492245	1	0.482927612	1
cDNA:S423457_2457	0.071413	1	0.789290499	1
cDNA:S423813_4844	0.147811	1	0.700636543	1
cDNA:S423813_7520	1.780881	1	0.182041168	0.961439446
cDNA:S424361_552	4.363797	1	0.036710402	0.873196429
cDNA:S42646_1319	0.169888	1	0.680211017	1
cDNA:S427444_2796	0.54045	1	0.462246108	1
cDNA:S427565_10290	0.83287	1	0.361444092	0.998652349
cDNA:S427654_5846	0.031291	1	0.859593153	1
cDNA:S43085_1432	1.443064	1	0.229644154	0.960075029
cDNA:S43178_5577	0.962649	1	0.326520435	0.996192022
cDNA:S433169_1653	0.247141	1	0.619095714	1
cDNA:S433351_2306	0.097688	1	0.754621452	1
cDNA:S434039_577	0.00098	1	0.975026917	1
cDNA:S434039_906	0.000225	1	0.988020216	1
cDNA:S434970_6835	1.344057	1	0.246319813	0.959095066
cDNA:S43503_3699	1.255267	1	0.262548816	0.996192022
cDNA:S435837_6673	0.001975	1	0.964552732	1
cDNA:S435837_8365	1.141781	1	0.285276403	0.97046332
cDNA:S436246_4872	2.304946	1	0.128962777	0.930719512
cDNA:S436446_3416	2.235334	1	0.134887474	0.888665245
cDNA:S437669_209	1.894333	1	0.168713991	0.930719512
cDNA:S438534_1353	1.505864	1	0.219771264	0.968970438
cDNA:S441699_8582	3.777704	1	0.051939879	0.782353909

cDNA:S441715_1042	0.603431	1	0.437271789	1
cDNA:S441715_114	3.289154	1	0.069738932	0.826214689
cDNA:S443195_3302	5.177891	1	0.022876072	0.669071895
cDNA:S443384_1089	1.300725	1	0.254080856	0.968970438
cDNA:S443429_844	0.0759	1	0.782932195	1
cDNA:S443930_1034	3.501317	1	0.061320041	0.812444444
cDNA:S444883_764	0.01487	1	0.902943991	1
cDNA:S445299_1229	0.323609	1	0.569446934	1
cDNA:S4461_8291	1.172612	1	0.278865202	0.972666047
cDNA:S44770_8034	1.473456	1	0.224801216	1
cDNA:S448226_3940	2.294743	1	0.129812656	0.923049505
cDNA:S453306_1429	0.020491	1	0.886174633	1
cDNA:S453499_827	10.33774	1	0.001303373	0.152333333
cDNA:S456400_1829	0.567602	1	0.451213324	1
cDNA:S456769_2371	2.19728	1	0.13825448	0.923049505
cDNA:S458790_919	0.018451	1	0.891951187	1
cDNA:S459129_1735	5.645241	1	0.017502809	0.669071895
cDNA:S46008_1521	0.669525	1	0.413217236	1
cDNA:S464216_6837	2.086474	1	0.148608686	0.923049505
cDNA:S467031_884	0.298122	1	0.585061954	1
cDNA:S467736_2080	0.000238	1	0.987678623	1
cDNA:S467736_2414	0.091484	1	0.762299439	1
cDNA:S467736_5425	0.155735	1	0.693113873	1
cDNA:S467960_5741	1.308144	1	0.252730997	0.961678135
cDNA:S46895_1964	0.010495	1	0.918403494	1
cDNA:S470748_4209	1.798947	1	0.179839877	0.959095066
cDNA:S470748_6041	0.001607	1	0.968022247	1
cDNA:S471028_893	0.069349	1	0.792286314	1
cDNA:S471822_9611	5.185043	1	0.022782111	0.671037975
cDNA:S472595_1354	0.000397	1	0.984109887	1
cDNA:S474699_744	0.007637	1	0.930359681	1
cDNA:S47498_732	0.126474	1	0.722116607	1
cDNA:S475438_2430	0.33713	1	0.561490315	1
cDNA:S47753_3153	4.272173	1	0.03874135	0.747448889
cDNA:S47755_4734	0.111259	1	0.738715776	1
cDNA:S478587_2350	1.307963	1	0.252763812	1
cDNA:S479169_1824	0.216047	1	0.642068659	1
cDNA:S479374_5109	2.816485	1	0.093300496	0.838442667
cDNA:S479522_1912	0.379847	1	0.537685266	1
cDNA:S479703_3376	0.066629	1	0.796310248	1
cDNA:S480496_654	0.972305	1	0.324106026	0.996192022
cDNA:S48128_2938	0.898015	1	0.343314619	0.972155745
cDNA:S483389_655	7.168237	1	0.007420567	0.448982456
cDNA:S486536_1792	2.110696	1	0.146272743	0.959095066
cDNA:S487087_629	1.208488	1	0.271631836	0.97046332
cDNA:S48744_11537	0.001975	1	0.964552732	1
cDNA:S48744_15351	0.66118	1	0.416143627	1
cDNA:S48744_3876	0.104896	1	0.746031937	1

cDNA:S488628_11250	0.433988	1	0.510038463	1
cDNA:S490342_1168	3.908822	1	0.048033245	0.787240876
cDNA:S490342_360	0.666188	1	0.414383888	1
cDNA:S491520_1295	0.144755	1	0.703599162	1
cDNA:S491520_853	0.101494	1	0.750044542	1
cDNA:S495254_1113	0.000281	1	0.986623142	1
cDNA:S495556_576	0.198701	1	0.655771362	1
cDNA:S495609_518	0.496461	1	0.481059221	1
cDNA:S496464_1251	2.376989	1	0.123134665	0.930719512
cDNA:S49912_2049	0.059701	1	0.806968367	1
cDNA:S49912_4653	0.228454	1	0.632672876	1
cDNA:S49912_673	1.830882	1	0.176023462	0.961439446
cDNA:S499496_4647	1.579165	1	0.208880983	0.996192022
cDNA:S49962_2185	1.074606	1	0.299907465	0.97046332
cDNA:S49962_7061	2.293677	1	0.129901865	0.873196429
cDNA:S502052_2637	0.24545	1	0.620297154	1
cDNA:S503987_1310	0.050035	1	0.823002925	1
cDNA:S50428_3932	0.328116	1	0.566770293	1
cDNA:S504876_1571	0.142895	1	0.705420252	1
cDNA:S506131_1661	4.671411	1	0.030668713	0.671037975
cDNA:S506450_638	2.473385	1	0.115788344	0.90850501
cDNA:S506501_872	1.026634	1	0.310950672	1
cDNA:S507443_10507	0.857028	1	0.354571708	1
cDNA:S507443_10963	0.597463	1	0.439547568	1
cDNA:S50805_3979	0.980208	1	0.32214749	0.996192022
cDNA:S508312_687	1.802688	1	0.179387842	0.959095066
cDNA:S509562_6631	1.156477	1	0.282197446	0.996192022
cDNA:S509786_2280	0.059701	1	0.806968367	1
cDNA:S512740_2933	0.154782	1	0.694006573	1
cDNA:S512740_4303	1.237697	1	0.265915369	0.968970438
cDNA:S512863_3037	1.810471	1	0.178451738	0.946642857
cDNA:S513732_2662	0.317357	1	0.573200072	1
cDNA:S513890_2408	2.12925	1	0.144511444	0.923049505
cDNA:S516836_5414	3.224236	1	0.072555661	0.831364384
cDNA:S5174_7473	1.456402	1	0.227503463	0.968970438
cDNA:S517417_2310	6.183624	1	0.01289379	0.669071895
cDNA:S518687_590	0.983666	1	0.321295315	0.996192022
cDNA:S519266_2405	1.527141	1	0.216541964	0.968970438
cDNA:S519981_717	0.008302	1	0.927399546	1
cDNA:S521910_1865	0.129802	1	0.718637367	1
cDNA:S524260_1085	1.679974	1	0.194927951	0.959095066
cDNA:S52430_1340	0.108084	1	0.742335543	1
cDNA:S52440_3397	0.051429	1	0.820594555	1
cDNA:S524433_4324	0.682209	1	0.408826991	1
cDNA:S524638_117	0.902387	1	0.342142408	0.996192022
cDNA:S525559_3367	0.019486	1	0.888982124	1
cDNA:S525559_3568	0.530368	1	0.466452373	1
cDNA:S525580_1370	0.887793	1	0.346076039	0.996192022

cDNA:S526017_13088	0.168644	1	0.681319369	1
cDNA:S52627_2009	0.983004	1	0.321458216	0.996192022
cDNA:S529070_2405	0.23676	1	0.626555726	1
cDNA:S52985_1976	9.301026	1	0.002290255	0.332363636
cDNA:S529917_2353	0.540382	1	0.462274623	1
cDNA:S530503_2428	1.20475	1	0.272374307	0.97046332
cDNA:S53142_3762	0.119545	1	0.729528	1
cDNA:S532222_1862	1.091218	1	0.296201593	0.996192022
cDNA:S535600_1124	1.68908	1	0.193722235	0.957294737
cDNA:S536151_3951	2.333153	1	0.126645147	0.910498084
cDNA:S538039_7455	0.45638	1	0.499320785	1
cDNA:S538614_2376	0.111966	1	0.737916839	1
cDNA:S53928_6647	0.302117	1	0.582558334	1
cDNA:S53964_3872	0.818913	1	0.365498081	1
cDNA:S54056_1950	0.939114	1	0.332505829	1
cDNA:S540909_4091	0.764986	1	0.381772723	1
cDNA:S541376_1738	0.024741	1	0.875012922	1
cDNA:S541784_2217	0.27545	1	0.599698983	1
cDNA:S542005_1955	4.751611	1	0.02927088	0.669071895
cDNA:S543174_1716	8.31E-07	1	0.999272518	1
cDNA:S543515_1408	0.339768	1	0.559963089	1
cDNA:S543515_1473	0.112346	1	0.73748894	1
cDNA:S546013_2702	2.915323	1	0.087741868	0.873196429
cDNA:S547212_811	0.388419	1	0.533131355	1
cDNA:S54802_2392	1.202685	1	0.272785641	0.996192022
cDNA:S548371_2277	0.001271	1	0.971560648	1
cDNA:S549631_6781	1.153524	1	0.282812628	1
cDNA:S549885_1391	0.63961	1	0.423851911	1
cDNA:S550666_852	0.014196	1	0.905160412	1
cDNA:S551527_3124	1.446224	1	0.229134833	0.980549309
cDNA:S552157_3420	0.157577	1	0.691397319	1
cDNA:S552588_975	1.694019	1	0.193072029	0.961678135
cDNA:S553258_6312	0.207418	1	0.648799019	1
cDNA:S553568_2443	0.540664	1	0.462157622	1
cDNA:S554410_3524	0.034347	1	0.852971196	1
cDNA:S55582_1063	0.131662	1	0.716714837	1
cDNA:S55677_6075	0.012104	1	0.912393704	1
cDNA:S557906_1632	4.018719	1	0.044997871	0.738065728
cDNA:S559237_2085	1.492537	1	0.221823198	0.97046332
cDNA:S559995_1169	1.587074	1	0.207744737	0.968970438
cDNA:S561043_583	0.229433	1	0.631944657	1
cDNA:S561676_2226	1.389134	1	0.23855143	0.959095066
cDNA:S562620_1139	3.457354	1	0.062971001	0.826214689
cDNA:S562620_2065	0.60921	1	0.435085488	1
cDNA:S565196_1646	3.217702	1	0.072845808	0.845861183
cDNA:S566778_923	0.036123	1	0.8492607	1
cDNA:S566907_1654	0.312608	1	0.576084274	1
cDNA:S566907_6021	0.405745	1	0.524137212	1

cDNA:S56850_1488	0.648529	1	0.420639053	1
cDNA:S56850_3419	0.505825	1	0.476951587	1
cDNA:S56850_553	0.003861	1	0.950455214	1
cDNA:S569210_2498	0.670094	1	0.413019008	1
cDNA:S569210_3034	3.775057	1	0.052022115	0.826214689
cDNA:S569396_1043	1.202685	1	0.272785641	0.992751958
cDNA:S569434_1910	1.190907	1	0.275146607	0.97046332
cDNA:S571359_1687	0.253728	1	0.614462357	1
cDNA:S571530_2439	0.791524	1	0.37363976	1
cDNA:S572204_1064	0.146002	1	0.702386079	1
cDNA:S576685_1429	0.241509	1	0.623118278	1
cDNA:S576685_1623	1.062359	1	0.30267775	0.996192022
cDNA:S577097_1137	0.062672	1	0.802321059	1
cDNA:S577556_2090	0.127493	1	0.721045237	1
cDNA:S579834_1157	4.557283	1	0.032779037	0.669071895
cDNA:S580273_477	0.229322	1	0.632027374	1
cDNA:S580446_422	0.181128	1	0.670405386	1
cDNA:S581445_450	0.438974	1	0.507618169	1
cDNA:S582565_327	0.005392	1	0.94146452	1
cDNA:S584975_3561	0.000206	1	0.988542558	1
cDNA:S586278_689	11.28006	1	0.000783439	0.322588235
cDNA:S58910_6486	1.344057	1	0.246319813	0.968970438
cDNA:S58910_7178	0.020404	1	0.88641571	1
cDNA:S589335_1602	0.074094	1	0.785466226	1
cDNA:S589758_2221	0.933058	1	0.334069465	0.996192022
cDNA:S590793_284	0.696352	1	0.404012072	0.996867696
cDNA:S591136_2909	0.057746	1	0.810093922	1
cDNA:S59200_2354	1.152387	1	0.283049995	0.996192022
cDNA:S594881_2145	0.35667	1	0.550361646	1
cDNA:S59534_1178	0.052826	1	0.818217156	1
cDNA:S596508_8961	0.076983	1	0.781428971	1
cDNA:S597808_1618	2.149254	1	0.142639206	0.923049505
cDNA:S59871_2796	0.869453	1	0.351106676	0.996192022
cDNA:S598943_992	2.551771	1	0.110170693	0.930719512
cDNA:S600558_675	0.341351	1	0.559050647	1
cDNA:S60342_1952	0.832999	1	0.361406636	0.996192022
cDNA:S603437_983	2.336256	1	0.12639297	0.923049505
cDNA:S606420_2644	0.130275	1	0.718147468	1
cDNA:S607119_856	1.047152	1	0.306163986	0.996192022
cDNA:S60720_2183	0.180619	1	0.670842247	1
cDNA:S608598_1333	1.706068	1	0.191496301	0.961678135
cDNA:S611416_1700	2.607309	1	0.106372139	0.873196429
cDNA:S611416_2436	0.089898	1	0.764307005	1
cDNA:S612731_1264	0.325041	1	0.568593548	1
cDNA:S614082_811	4.120735	1	0.042360681	0.762314894
cDNA:S619969_5417	3.108393	1	0.07788977	0.826214689
cDNA:S621565_1910	0.567602	1	0.451213324	1
cDNA:S621834_5067	0.285879	1	0.592873744	1

cDNA:S623208_2215	0.857028	1	0.354571708	1
cDNA:S623498_4238	0.069463	1	0.792120502	1
cDNA:S624913_741	0.018206	1	0.892666459	1
cDNA:S625101_1079	0.207001	1	0.649128223	1
cDNA:S628260_5673	1.602103	1	0.205605393	0.957294737
cDNA:S62874_2994	0.026511	1	0.870657674	1
cDNA:S629902_2397	5.875635	1	0.015351833	0.58496
cDNA:S632206_1402	2.661397	1	0.102810383	0.923049505
cDNA:S63343_1741	0.558735	1	0.454770415	1
cDNA:S635018_516	0.77674	1	0.378140062	1
cDNA:S638107_1152	2.104804	1	0.146837134	0.930719512
cDNA:S638107_1835	0.6108	1	0.434486832	1
cDNA:S638202_1141	1.428029	1	0.232086322	0.97046332
cDNA:S638468_283	0.112259	1	0.737587266	1
cDNA:S639012_1637	1.171128	1	0.279169634	1
cDNA:S639300_3047	0.225163	1	0.635133984	1
cDNA:S639484_1049	2.894713	1	0.08887059	0.829512605
cDNA:S64181_3413	0.033638	1	0.854478967	1
cDNA:S64260_3920	0.007774	1	0.92974164	1
cDNA:S642705_1575	1.389312	1	0.238521321	1
cDNA:S64643_2871	1.059786	1	0.303264059	0.996192022
cDNA:S649928_1079	0.338243	1	0.560845309	1
cDNA:S649928_1697	1.085123	1	0.297554291	0.996192022
cDNA:S651830_6088	1.547521	1	0.213501743	0.991787234
cDNA:S65210_2420	0.013473	1	0.907593396	1
cDNA:S652596_4392	1.831691	1	0.175927929	0.996192022
cDNA:S65320_3890	0.004474	1	0.946670294	1
cDNA:S654208_2204	0.159786	1	0.689353383	1
cDNA:S655513_1414	3.097067	1	0.078433487	0.838442667
cDNA:S655629_4062	0.18116	1	0.670378066	1
cDNA:S660504_2091	3.035273	1	0.081472837	0.873196429
cDNA:S660672_1738	0.894592	1	0.344235873	0.98872956
cDNA:S663695_501	4.196204	1	0.040514586	0.756969163
cDNA:S665865_592	0.040085	1	0.841315297	1
cDNA:S666270_3095	0.689081	1	0.406476984	0.996192022
cDNA:S670900_2441	0.227815	1	0.63314882	1
cDNA:S671649_1009	2.515136	1	0.112757895	0.873196429
cDNA:S672808_3857	3.661932	1	0.05566905	0.78448583
cDNA:S672840_1084	0.00098	1	0.975026917	1
cDNA:S672896_1109	1.188458	1	0.275640905	0.972666047
cDNA:S674382_1528	0.090494	1	0.763549579	1
cDNA:S675938_3404	0.014196	1	0.905160412	1
cDNA:S676635_1323	17.03164	1	3.68E-05	0
cDNA:S678589_1098	0.517062	1	0.472097785	1
cDNA:S678913_2022	5.007697	1	0.025234862	0.669071895
cDNA:S683469_299	0.119847	1	0.729200358	1
cDNA:S68403_1523	3.234933	1	0.072083243	0.846554707
cDNA:S684263_1131	0.035604	1	0.850336141	1

cDNA:S68433_2631	0.023443	1	0.878309564	1
cDNA:S685260_460	4.246659	1	0.039327623	0.712451282
cDNA:S68579_5662	0.134499	1	0.713811947	1
cDNA:S687148_2057	0.647102	1	0.421150573	1
cDNA:S687718_121	0.165837	1	0.683837982	1
cDNA:S68977_1000	5.37327	1	0.020447609	0.706272727
cDNA:S689999_655	1.725697	1	0.188961368	0.959095066
cDNA:S69030_5885	7.168237	1	0.007420567	0.448982456
cDNA:S694773_326	0.889128	1	0.345713609	0.996192022
cDNA:S696948_633	1.838621	1	0.175112713	0.957294737
cDNA:S698052_515	7.11989	1	0.007623337	0.595162791
cDNA:S699696_588	0.078079	1	0.779917438	1
cDNA:S700526_485	0.117015	1	0.732294869	1
cDNA:S702119_7663	0.27545	1	0.599698983	1
cDNA:S704032_1342	0.862686	1	0.352988119	1
cDNA:S706325_1228	0.002095	1	0.963493024	1
cDNA:S706337_4219	1.490476	1	0.222142545	0.968970438
cDNA:S710664_1215	0.035507	1	0.850537792	1
cDNA:S711523_1175	2.227605	1	0.135563823	0.923049505
cDNA:S715975_2165	0.528604	1	0.467194149	1
cDNA:S715975_734	0.178099	1	0.673011667	1
cDNA:S719929_1070	0.933058	1	0.334069465	0.996192022
cDNA:S720470_272	2.887961	1	0.089243815	0.873196429
cDNA:S722226_5785	1.95231	1	0.162338116	0.959095066
cDNA:S72460_1666	5.224121	1	0.022275704	0.707612903
cDNA:S724824_2713	0.643276	1	0.422527137	1
cDNA:S72507_1383	0.048449	1	0.825785233	1
cDNA:S726233_1591	0.131662	1	0.716714837	1
cDNA:S727135_932	0.53809	1	0.463225392	1
cDNA:S727972_1217	3.192852	1	0.073960853	0.831364384
cDNA:S73079_6850	1.822015	1	0.177073583	0.959095066
cDNA:S730927_529	1.062359	1	0.30267775	0.996192022
cDNA:S732543_1370	0.175455	1	0.675308734	1
cDNA:S734982_525	0.067722	1	0.794683461	1
cDNA:S736834_3880	3.529462	1	0.060287361	0.787240876
cDNA:S73751_1345	7.404794	1	0.00650503	0.669071895
cDNA:S73902_5315	0.849667	1	0.356646492	0.996192022
cDNA:S739620_1210	0.625321	1	0.429076744	1
cDNA:S739704_2409	0.056807	1	0.811616466	1
cDNA:S741234_1247	0.862173	1	0.353131214	0.996192022
cDNA:S741234_882	0.323609	1	0.569446934	1
cDNA:S741878_741	0.00283	1	0.957577477	1
cDNA:S744004_654	0.13587	1	0.712421516	1
cDNA:S744265_3173	0.238806	1	0.625069839	1
cDNA:S74748_3603	0.577757	1	0.447192893	1
cDNA:S748975_1097	1.24902	1	0.26373976	0.968970438
cDNA:S74898_3892	0.279856	1	0.596795436	1
cDNA:S74898_5777	0.395382	1	0.529483964	1

cDNA:S74898_7495	0.051429	1	0.820594555	1
cDNA:S74998_6460	0.567602	1	0.451213324	1
cDNA:S74998_8681	0.505825	1	0.476951587	1
cDNA:S75019_4677	0.325302	1	0.568438439	1
cDNA:S755450_1408	3.944015	1	0.047038383	0.741308756
cDNA:S755749_1729	0.067162	1	0.795513944	1
cDNA:S757328_1267	0.857523	1	0.354432773	0.996192022
cDNA:S760869_330	1.080373	1	0.298614153	1
cDNA:S76268_7320	3.132173	1	0.076761351	0.885814978
cDNA:S762880_153	3.71448	1	0.053942409	0.826214689
cDNA:S764899_4758	1.742976	1	0.186762181	0.959095066
cDNA:S76557_5098	4.306044	1	0.037977181	0.759688312
cDNA:S76720_2288	0.73613	1	0.390903536	0.996192022
cDNA:S7674_5559	5.147401	1	0.023281193	0.706272727
cDNA:S767508_719	0.032106	1	0.857794913	1
cDNA:S773321_1853	0.419472	1	0.517200733	1
cDNA:S77337_1096	0.240368	1	0.623940336	1
cDNA:S77337_2763	1.653294	1	0.198511177	0.961678135
cDNA:S774473_3443	1.860275	1	0.172593028	0.957294737
cDNA:S774473_657	0.496483	1	0.481049641	1
cDNA:S77853_1811	0.191748	1	0.66146592	1
cDNA:S77867_5110	8.31E-07	1	0.999272518	1
cDNA:S779032_1920	0.12093	1	0.728028461	1
cDNA:S78201_3151	0.085853	1	0.769516726	1
cDNA:S782156_6421	2.044963	1	0.152710695	0.930719512
cDNA:S78222_1368	0.370939	1	0.542492973	1
cDNA:S784689_1447	2.495803	1	0.114150149	0.90096945
cDNA:S785610_1712	0.081158	1	0.775733591	1
cDNA:S78668_2682	2.557901	1	0.109744209	0.915683241
cDNA:S78935_3658	0.443197	1	0.505583244	1
cDNA:S792758_2028	6.305402	1	0.012037063	0.632769231
cDNA:S793090_1806	0.052385	1	0.818964768	1
cDNA:S797162_1001	0.663194	1	0.415434591	1
cDNA:S79964_174	0.21454	1	0.643232334	1
cDNA:S80201_8381	1.701593	1	0.192079811	0.957294737
cDNA:S802713_646	0.002055	1	0.963842268	1
cDNA:S806252_551	0.367957	1	0.544119886	1
cDNA:S806252_634	2.227605	1	0.135563823	0.948045845
cDNA:S807177_306	0.001271	1	0.971560648	1
cDNA:S807823_507	11.80818	1	0.000589709	0.152333333
cDNA:S80843_7912	1.799832	1	0.179732785	0.959095066
cDNA:S80986_5698	0.055148	1	0.814335373	1
cDNA:S816485_4193	0.250389	1	0.616801394	1
cDNA:S8184_724	0.069463	1	0.792120502	1
cDNA:S819025_2420	0.074094	1	0.785466226	1
cDNA:S819025_669	0.000936	1	0.975588981	1
cDNA:S822390_3958	9.65326	1	0.001890158	0.243733333
cDNA:S823443_822	0.089898	1	0.764307005	1

cDNA:S823547_277	0.037313	1	0.846830009	1
cDNA:S825915_1100	0.136866	1	0.711417194	1
cDNA:S82741_15301	0.492245	1	0.482927612	1
cDNA:S82741_16645	0.154544	1	0.694230013	1
cDNA:S828038_587	1.174568	1	0.278464603	0.996192022
cDNA:S829392_887	0.896144	1	0.343817762	0.996192022
cDNA:S829676_1368	1.362767	1	0.243058596	0.996192022
cDNA:S830139_334	0.003085	1	0.955703596	1
cDNA:S831140_2740	0.020491	1	0.886174633	1
cDNA:S833938_256	0.524603	1	0.468884852	1
cDNA:S835090_2041	0.136026	1	0.712264306	1
cDNA:S835857_600	0.000837	1	0.976913946	1
cDNA:S837797_2394	2.875906	1	0.089914365	0.873196429
cDNA:S837797_363	0.003273	1	0.954376861	1
cDNA:S838704_1071	0.825554	1	0.363561398	0.996192022
cDNA:S839443_3282	0.455272	1	0.499841895	1
cDNA:S84686_2112	0.001975	1	0.964552732	1
cDNA:S84920_5746	5.379889	1	0.020370176	0.669071895
cDNA:S8550_272	9.517219	1	0.002035528	0.152333333
cDNA:S85734_1781	0.966289	1	0.325607401	0.996192022
cDNA:S86370_1080	2.039138	1	0.153296474	0.948045845
cDNA:S86519_8078	0.003085	1	0.955703596	1
cDNA:S87097_4670	2.545642	1	0.110598922	0.957294737
cDNA:S87097_5007	1.049842	1	0.305543499	1
cDNA:S87911_1237	0.011578	1	0.914312298	1
cDNA:S87911_5985	0.111775	1	0.738132577	1
cDNA:S89683_2533	1.208292	1	0.271670717	0.968970438
cDNA:S89683_2659	1.742976	1	0.186762181	0.996192022
cDNA:S89695_5963	0.06859	1	0.793400898	1
cDNA:S90292_4948	0.308669	1	0.578497817	1
cDNA:S91962_7567	7.211354	1	0.007244381	0.632769231
cDNA:S92500_6012	1.799832	1	0.179732785	0.948045845
cDNA:S92682_5328	11.69973	1	0.000625092	0.809542857
cDNA:S93807_4051	8.579465	1	0.003399753	0.152333333
cDNA:S95432_762	0.303532	1	0.581676721	1
cDNA:S961_6169	0.000557	1	0.981175494	1
cDNA:S96224_8004	0.059604	1	0.807122346	1
cDNA:S96652_4022	0.103952	1	0.747137893	1
cDNA:S97807_8343	1.15E-05	1	0.997292869	1
cDNA:S97921_4263	0.342124	1	0.55860554	1
cDNA:S97964_2369	0.017468	1	0.894852073	1
cDNA:S98811_398	1.424313	1	0.232694825	0.969060241
Gdist:S100032_8838	0.051898	1	0.819793441	1
Gdist:S100275_4086	0.862686	1	0.352988119	1
Gdist:S100352_5216	0.057958	1	0.80975224	1
Gdist:S100408_780	2.473385	1	0.115788344	0.894878661
Gdist:S100414_3290	0.829701	1	0.36235899	0.996192022
Gdist:S100441_8833	1.012838	1	0.314223838	0.996192022

Gdist:S10083_8654	0.228679	1	0.632504783	1
Gdist:S100907_122	0.00703	1	0.933178166	1
Gdist:S100942_9401	1.274613	1	0.258903033	0.97046332
Gdist:S100973_6375	1.63047	1	0.201638114	0.957294737
Gdist:S101169_6923	1.172612	1	0.278865202	0.976510166
Gdist:S101602_568	0.055815	1	0.813237214	1
Gdist:S101675_4227	0.382486	1	0.536275674	1
Gdist:S102093_4513	0.103952	1	0.747137893	1
Gdist:S102207_543	0.442553	1	0.505892884	1
Gdist:S10222_9302	0.17828	1	0.672855578	1
Gdist:S102366_4913	0.239725	1	0.624405116	1
Gdist:S10257_3175	0.033073	1	0.855693693	1
Gdist:S10297_8837	1.274655	1	0.258895175	0.992434326
Gdist:S103101_1480	0.113717	1	0.735951842	1
Gdist:S103230_2073	0.034968	1	0.851662118	1
Gdist:S104125_7099	0.069349	1	0.792286314	1
Gdist:S104238_4733	0.314091	1	0.575180331	1
Gdist:S104594_982	0.48232	1	0.487373545	1
Gdist:S104771_5605	0.183444	1	0.668430286	1
Gdist:S104822_7598	0.980208	1	0.32214749	0.996192022
Gdist:S104914_2923	1.443064	1	0.229644154	0.965886463
Gdist:S104994_4018	0.637571	1	0.424591751	1
Gdist:S105008_3937	5.666467	1	0.017292248	0.669071895
Gdist:S105084_3459	0.66053	1	0.416372841	1
Gdist:S105127_8748	0.220364	1	0.63876254	1
Gdist:S105141_3863	0.615524	1	0.432715592	1
Gdist:S105141_4123	0.013104	1	0.908864264	1
Gdist:S105720_2405	3.885057	1	0.048717603	0.823205298
Gdist:S105837_10378	0.009081	1	0.924080386	1
Gdist:S106111_3946	0.942055	1	0.331749902	1
Gdist:S106382_7119	0.6108	1	0.434486832	1
Gdist:S106496_4572	0.536355	1	0.463947316	1
Gdist:S106782_8017	0.339768	1	0.559963089	1
Gdist:S106795_4580	1.798947	1	0.179839877	0.960075029
Gdist:S106843_3060	0.583886	1	0.444793134	1
Gdist:S107142_234	0.889128	1	0.345713609	0.996192022
Gdist:S107142_7279	0.620937	1	0.430699352	1
Gdist:S107235_2071	0.907506	1	0.340776944	0.996192022
Gdist:S107890_2224	0.227434	1	0.633432727	1
Gdist:S107902_230	2.14682	1	0.142865481	0.923049505
Gdist:S108308_5065	0.010535	1	0.91824946	1
Gdist:S108339_5983	0.144755	1	0.703599162	1
Gdist:S108339_7213	0.323879	1	0.569285725	1
Gdist:S108412_1150	0.101494	1	0.750044542	1
Gdist:S108805_7766	0.15583	1	0.693025436	1
Gdist:S108825_7124	1.292438	1	0.255599094	0.962317181
Gdist:S10940_2042	0.18651	1	0.665837729	1
Gdist:S10968_860	3.319623	1	0.068457537	0.823205298

Gdist:S109710_14627	0.656945	1	0.41764043	1
Gdist:S109921_14334	3.334374	1	0.067846203	0.826214689
Gdist:S110756_790	0.594814	1	0.440563428	0.996192022
Gdist:S110827_3868	1.53311	1	0.215646222	0.957294737
Gdist:S110976_581	0.147774	1	0.700672123	1
Gdist:S111475_3504	1.202578	1	0.272807064	0.98872956
Gdist:S111632_10971	0.465487	1	0.495070592	1
Gdist:S111722_10937	0.015435	1	0.901128131	1
Gdist:S111849_1134	2.510139	1	0.113115942	0.923049505
Gdist:S112167_4394	0.544698	1	0.46049222	1
Gdist:S112167_5363	0.006972	1	0.933454814	1
Gdist:S112240_3631	1.969922	1	0.160456094	0.923049505
Gdist:S112613_8383	2.682033	1	0.101486121	0.897039175
Gdist:S1127_7992	0.898015	1	0.343314619	0.972666047
Gdist:S112883_6809	0.100854	1	0.750806963	1
Gdist:S112887_2627	0.205909	1	0.649992779	1
Gdist:S112887_7924	0.234688	1	0.628069424	1
Gdist:S112993_2878	0.779613	1	0.377259486	0.996192022
Gdist:S113093_3207	0.485784	1	0.48581396	1
Gdist:S11313_2046	0.361514	1	0.547666584	1
Gdist:S113214_1620	0.079868	1	0.777476049	1
Gdist:S113290_2526	1.246883	1	0.264148739	0.996192022
Gdist:S113363_8211	2.097863	1	0.147505162	0.930719512
Gdist:S113620_6102	0.959035	1	0.327430094	0.996192022
Gdist:S113823_3877	2.092669	1	0.148007255	0.914
Gdist:S113953_6012	0.024905	1	0.87460511	1
Gdist:S113957_4095	2.370988	1	0.123608801	0.914
Gdist:S113989_5664	0.001135	1	0.973126044	1
Gdist:S114106_2022	1.78717	1	0.181271389	0.968970438
Gdist:S114106_7048	0.071702	1	0.788874323	1
Gdist:S114220_1645	0.00849	1	0.926584577	1
Gdist:S114372_2369	0.146002	1	0.702386079	1
Gdist:S114376_8423	0.968737	1	0.324995424	0.996192022
Gdist:S114561_7994	0.147488	1	0.70094804	1
Gdist:S115075_8430	4.749311	1	0.029310028	0.712451282
Gdist:S115441_7423	0.896144	1	0.343817762	0.996192022
Gdist:S115607_10942	0.711379	1	0.398986368	1
Gdist:S115795_8147	0.019438	1	0.889116898	1
Gdist:S115799_3157	0.370725	1	0.542609176	1
Gdist:S115968_7253	0.142835	1	0.705479145	1
Gdist:S116080_1571	0.616184	1	0.432468741	1
Gdist:S116118_10589	1.268687	1	0.260013117	0.996192022
Gdist:S116213_5101	4.504542	1	0.03380494	0.669071895
Gdist:S116218_6824	0.006623	1	0.935138282	1
Gdist:S11640_7049	0.594814	1	0.440563428	0.996192022
Gdist:S116498_6891	4.363797	1	0.036710402	0.823205298
Gdist:S116612_6115	1.880248	1	0.170305921	0.930719512
Gdist:S116660_10167	0.026701	1	0.870199818	1

Gdist:S116660_5693	0.724633	1	0.394628536	0.996192022
Gdist:S116897_7662	0.654756	1	0.418417435	1
Gdist:S117158_3382	0.04132	1	0.838921501	1
Gdist:S11737_8352	0.898283	1	0.343242641	1
Gdist:S117671_3409	1.200344	1	0.273252836	0.97046332
Gdist:S11806_2740	1.520019	1	0.217616632	0.961678135
Gdist:S11806_3970	1.480562	1	0.22368661	0.961678135
Gdist:S11886_5301	0.02645	1	0.87080504	1
Gdist:S119058_6641	1.190154	1	0.275298493	0.982108556
Gdist:S119128_9527	0.557107	1	0.455428156	1
Gdist:S11927_5169	0.165837	1	0.683837982	1
Gdist:S119433_5515	0.010966	1	0.916600637	1
Gdist:S119433_5963	0.324819	1	0.568726177	1
Gdist:S119534_10343	0.644477	1	0.422094333	1
Gdist:S119534_5653	0.411411	1	0.521254359	1
Gdist:S119548_3098	0.587453	1	0.443405628	1
Gdist:S119611_3628	0.001059	1	0.974044128	1
Gdist:S119816_3896	8.310257	1	0.003942181	0.332363636
Gdist:S120141_2338	0.609469	1	0.434987624	1
Gdist:S120321_14257	0.157706	1	0.691277637	1
Gdist:S120321_5469	0.540664	1	0.462157622	1
Gdist:S120406_890	1.421221	1	0.233202499	0.969060241
Gdist:S120580_14694	0.118754	1	0.73038994	1
Gdist:S120639_8272	5.83934	1	0.015671701	0.669071895
Gdist:S120922_10442	3.072494	1	0.079627231	0.873196429
Gdist:S120982_3932	0.280959	1	0.596073482	1
Gdist:S121022_623	0.542915	1	0.461227163	1
Gdist:S121123_1695	0.857679	1	0.354389037	0.996192022
Gdist:S121123_6870	1.838621	1	0.175112713	0.957294737
Gdist:S121444_1180	3.314643	1	0.068665233	0.826214689
Gdist:S121446_4034	0.164823	1	0.684754764	1
Gdist:S121954_1538	1.117159	1	0.290531088	0.996192022
Gdist:S121987_4199	0.134165	1	0.714152045	1
Gdist:S121987_6689	0.000929	1	0.975679869	1
Gdist:S122028_6849	0.48165	1	0.487675909	1
Gdist:S122598_1256	5.768175	1	0.016318978	0.661561905
Gdist:S122610_1283	1.300725	1	0.254080856	0.97046332
Gdist:S122960_5739	2.234964	1	0.134919767	0.957294737
Gdist:S123104_1259	4.568363	1	0.032567676	0.823205298
Gdist:S12320_3350	2.577914	1	0.10836437	0.910498084
Gdist:S123212_5018	0.034347	1	0.852971196	1
Gdist:S123238_331	1.396315	1	0.237341534	0.968970438
Gdist:S123418_7093	0.071702	1	0.788874323	1
Gdist:S123827_11252	0.117653	1	0.731594504	1
Gdist:S124088_7851	0.878316	1	0.348663227	1
Gdist:S124475_1258	6.72E-05	1	0.9934587	1
Gdist:S124475_421	0.786281	1	0.375227099	0.996192022
Gdist:S124786_413	2.726165	1	0.098716099	0.873196429

Gdist:S124875_7407	2.070682	1	0.150154406	0.923049505
Gdist:S124875_7732	0.059701	1	0.806968367	1
Gdist:S124973_3491	0.12093	1	0.728028461	1
Gdist:S125_4975	0.078163	1	0.779803117	1
Gdist:S125070_10203	2.255602	1	0.133131702	0.930719512
Gdist:S125086_6731	0.751595	1	0.385971578	1
Gdist:S125305_3642	0.250389	1	0.616801394	1
Gdist:S125452_7634	0.000379	1	0.984474824	1
Gdist:S125541_4530	0.353239	1	0.552285228	1
Gdist:S125710_1245	0.298122	1	0.585061954	1
Gdist:S12575_1547	1.099253	1	0.294430035	0.972155745
Gdist:S126223_591	0.319694	1	0.571791316	1
Gdist:S126246_5762	0.088741	1	0.765784135	1
Gdist:S126436_9807	0.091484	1	0.762299439	1
Gdist:S126650_9899	0.243629	1	0.621597499	1
Gdist:S127042_3442	5.588934	1	0.018074296	0.669071895
Gdist:S127471_4594	0.034077	1	0.853543542	1
Gdist:S127569_9615	0.027254	1	0.868873775	1
Gdist:S127792_6520	0.015435	1	0.901128131	1
Gdist:S128049_4987	0.589242	1	0.442712292	1
Gdist:S12805_1108	0.3054	1	0.580516751	1
Gdist:S128191_5714	0.569598	1	0.450418641	1
Gdist:S128191_9860	0.440245	1	0.507004197	1
Gdist:S128480_1702	0.260189	1	0.609990233	1
Gdist:S12863_5738	0.339768	1	0.559963089	1
Gdist:S129244_13486	1.674933	1	0.195599091	0.961678135
Gdist:S129778_1028	0.940194	1	0.332227887	0.996192022
Gdist:S129785_3163	0.328116	1	0.566770293	1
Gdist:S129848_5047	5.166774	1	0.023022937	0.669071895
Gdist:S12991_3989	0.81677	1	0.366126218	0.996192022
Gdist:S130140_5833	0.171128	1	0.679111156	1
Gdist:S130202_7380	0.267634	1	0.60492344	1
Gdist:S130372_767	4.069337	1	0.043668399	0.747448889
Gdist:S13053_6942	1.719127	1	0.189805434	0.957294737
Gdist:S130620_9198	0.654105	1	0.418648702	1
Gdist:S130688_5646	3.025039	1	0.081988335	0.838442667
Gdist:S13164_5885	0.180856	1	0.670638936	1
Gdist:S131730_2802	1.965942	1	0.160879232	0.957294737
Gdist:S131798_5083	0.701689	1	0.40221684	1
Gdist:S131843_326	0.754096	1	0.385182437	1
Gdist:S132332_6141	0.012988	1	0.909264519	1
Gdist:S13235_7808	0.154657	1	0.694123695	1
Gdist:S132532_3332	0.253728	1	0.614462357	1
Gdist:S132532_5276	2.014158	1	0.155837666	0.960075029
Gdist:S132618_1961	0.042658	1	0.836370966	1
Gdist:S1329_4466	0.318019	1	0.572800737	1
Gdist:S132906_5083	1.386949	1	0.238920999	0.991787234
Gdist:S132944_1725	0.466699	1	0.494509687	1

Gdist:S133403_1620	0.285879	1	0.592873744	1
Gdist:S133717_6434	0.030598	1	0.861140384	1
Gdist:S13432_7148	0.329106	1	0.566186242	1
Gdist:S134417_1070	1.476286	1	0.224356574	0.969060241
Gdist:S134481_11196	0.895732	1	0.343928589	0.996192022
Gdist:S134518_5486	0.039878	1	0.841718881	1
Gdist:S134747_2885	5.348419	1	0.020741068	0.669071895
Gdist:S134762_1869	0.814186	1	0.366885655	1
Gdist:S135079_5018	1.701593	1	0.192079811	0.957294737
Gdist:S135310_8259	0.731754	1	0.392315383	1
Gdist:S135388_5797	2.169981	1	0.140727967	0.923049505
Gdist:S135483_11558	0.171128	1	0.679111156	1
Gdist:S135888_8430	0.101494	1	0.750044542	1
Gdist:S135969_7447	0.849011	1	0.356832368	0.996192022
Gdist:S136510_7699	1.796675	1	0.180115043	0.962317181
Gdist:S136603_6180	0.567602	1	0.451213324	0.996867696
Gdist:S1367_5121	0.001615	1	0.967940846	1
Gdist:S136955_6950	1.59E-05	1	0.99682218	1
Gdist:S137108_15317	1.067256	1	0.301566131	0.996192022
Gdist:S137268_1456	3.708548	1	0.05413447	0.787240876
Gdist:S137439_430	0.484544	1	0.486371374	1
Gdist:S137633_1828	1.415577	1	0.234132726	0.968970438
Gdist:S137722_2258	5.007697	1	0.025234862	0.677037037
Gdist:S137894_1019	0.539526	1	0.462629238	1
Gdist:S137942_5888	0.034407	1	0.852843527	1
Gdist:S137969_2061	0.793228	1	0.373125782	0.996867696
Gdist:S137971_2840	4.540339	1	0.033105037	0.741308756
Gdist:S138090_1240	0.132682	1	0.71566685	1
Gdist:S138607_4627	0.344152	1	0.55744245	1
Gdist:S139261_7130	0.266391	1	0.605763052	1
Gdist:S139450_15410	0.048449	1	0.825785233	1
Gdist:S13963_8153	0.360036	1	0.548486172	1
Gdist:S13965_9753	2.91421	1	0.087802416	0.83679564
Gdist:S139852_8868	0.237218	1	0.626222779	1
Gdist:S139852_9194	0.041504	1	0.838568924	1
Gdist:S141176_6740	3.019848	1	0.082251174	0.873196429
Gdist:S141935_5679	0.03619	1	0.849123286	1
Gdist:S142071_2129	0.28754	1	0.591801434	1
Gdist:S142071_5198	0.751753	1	0.385921654	0.996192022
Gdist:S142531_1964	0.776056	1	0.378350202	1
Gdist:S142540_7602	0.102319	1	0.749064403	1
Gdist:S142660_3589	0.015458	1	0.901055389	1
Gdist:S142662_6335	0.236605	1	0.626669084	1
Gdist:S14288_3111	0.428019	1	0.512962706	1
Gdist:S14288_8090	0.876829	1	0.349071683	1
Gdist:S143250_7038	0.029567	1	0.86347532	1
Gdist:S143339_5595	1.58941	1	0.207410421	0.962317181
Gdist:S143538_2537	1.917625	1	0.166118705	0.94994382

Gdist:S143538_4173	0.854426	1	0.355303222	0.996192022
Gdist:S14395_8283	5.156157	1	0.023164092	0.671037975
Gdist:S144932_5186	1.806633	1	0.178912648	0.961678135
Gdist:S14496_6161	1.08262	1	0.298112284	0.961678135
Gdist:S144962_3154	3.14944	1	0.075953007	0.826214689
Gdist:S14505_7999	2.039138	1	0.153296474	0.948045845
Gdist:S145778_3425	0.116826	1	0.73250315	1
Gdist:S146062_1439	0.530882	1	0.466236179	1
Gdist:S146211_5690	0.980208	1	0.32214749	0.996192022
Gdist:S146363_4892	2.110696	1	0.146272743	0.957294737
Gdist:S146373_3747	0.222617	1	0.637053248	1
Gdist:S146429_6829	0.524603	1	0.468884852	1
Gdist:S146498_5322	0.046927	1	0.828500139	1
Gdist:S14656_1414	0.713339	1	0.398337651	0.996192022
Gdist:S146747_4637	0.410148	1	0.521894343	1
Gdist:S146766_6312	0.214794	1	0.643035821	1
Gdist:S147033_6365	0.074094	1	0.785466226	1
Gdist:S147058_566	3.62196	1	0.057021648	0.787240876
Gdist:S147619_10126	0.490969	1	0.483495451	1
Gdist:S147812_7826	3.533886	1	0.060126709	0.826214689
Gdist:S148_7189	0.004566	1	0.946124344	1
Gdist:S148133_1069	0.001082	1	0.973764239	1
Gdist:S148298_4701	2.18136	1	0.139690964	0.948045845
Gdist:S149227_4245	1.74178	1	0.186913396	0.94994382
Gdist:S149456_654	0.007809	1	0.929581905	1
Gdist:S149801_7514	0.567602	1	0.451213324	0.996192022
Gdist:S150027_10819	0.247141	1	0.619095714	1
Gdist:S150027_8437	0.200027	1	0.654699262	1
Gdist:S150041_7238	0.25402	1	0.614258688	1
Gdist:S150041_834	3.267155	1	0.070680112	0.826214689
Gdist:S150342_1884	0.019975	1	0.887608047	1
Gdist:S150354_5018	0.057958	1	0.80975224	1
Gdist:S150354_8511	3.612714	1	0.057339461	0.738065728
Gdist:S150464_1018	4.156779	1	0.041468199	0.787240876
Gdist:S150464_4617	0.061524	1	0.80410269	1
Gdist:S150556_3024	6.931403	1	0.008469524	0.574514286
Gdist:S150556_4538	3.132173	1	0.076761351	0.896898129
Gdist:S150556_9200	1.037871	1	0.308317241	0.996192022
Gdist:S150832_4425	0.489492	1	0.484153801	1
Gdist:S151026_8038	1.145219	1	0.284552149	0.989962466
Gdist:S151287_12936	1.150141	1	0.28351969	1
Gdist:S151336_5896	0.055148	1	0.814335373	1
Gdist:S151336_6037	0.639876	1	0.423755797	1
Gdist:S151488_906	1.40068	1	0.236609749	0.959095066
Gdist:S151671_9639	1.29856	1	0.254476465	0.961678135
Gdist:S151794_4079	2.384512	1	0.122543175	0.910498084
Gdist:S152151_10254	0.223134	1	0.636662031	1
Gdist:S152151_3686	2.293677	1	0.129901865	0.914

Gdist:S15291_5095	0.035646	1	0.850249308	1
Gdist:S153277_4973	6.066989	1	0.013773156	0.539409836
Gdist:S153467_6701	0.11936	1	0.729729282	1
Gdist:S153878_3763	0.023443	1	0.878309564	1
Gdist:S153878_9142	0.062797	1	0.802128482	1
Gdist:S154339_585	0.023762	1	0.877491647	1
Gdist:S154339_6006	0.067722	1	0.794683461	1
Gdist:S154543_7222	1.344057	1	0.246319813	0.968970438
Gdist:S154739_6213	1.937222	1	0.163970442	0.996192022
Gdist:S154926_11931	0.129802	1	0.718637367	1
Gdist:S154926_15184	0.469229	1	0.493342003	1
Gdist:S154926_5381	1.428681	1	0.231979814	0.961678135
Gdist:S155219_1764	3.214887	1	0.072971219	0.845861183
Gdist:S156336_679	2.095979	1	0.147687129	0.948045845
Gdist:S156350_814	0.054362	1	0.815639457	1
Gdist:S156572_1700	0.000936	1	0.975588981	1
Gdist:S156620_726	0.438974	1	0.507618169	1
Gdist:S156829_2282	0.643203	1	0.422553415	1
Gdist:S156988_213	0.21454	1	0.643232334	1
Gdist:S157336_8348	0.052609	1	0.818584161	1
Gdist:S157374_15551	0.886251	1	0.346495377	0.996867696
Gdist:S157867_5401	2.228961	1	0.135444896	0.923049505
Gdist:S158346_7065	0.066153	1	0.797023262	1
Gdist:S158589_6904	0.455272	1	0.499841895	1
Gdist:S158610_4775	0.051898	1	0.819793441	1
Gdist:S158675_6988	1.491561	1	0.221974422	0.974933333
Gdist:S158947_7421	1.326617	1	0.249407964	0.968970438
Gdist:S159056_11023	3.22695	1	0.072435467	0.845153247
Gdist:S159056_4732	1.938411	1	0.163841154	0.959095066
Gdist:S1595_5752	0.171128	1	0.679111156	1
Gdist:S160069_12300	0.953549	1	0.328817627	0.996192022
Gdist:S160823_4331	1.174568	1	0.278464603	0.991787234
Gdist:S16205_6576	0.140551	1	0.707733609	1
Gdist:S16214_3578	1.256572	1	0.262300884	0.969060241
Gdist:S162224_9547	0.776056	1	0.378350202	1
Gdist:S162264_6776	0.000392	1	0.984213615	1
Gdist:S162264_883	1.559733	1	0.211704347	0.959095066
Gdist:S162384_609	0.005642	1	0.940126088	1
Gdist:S16260_2275	0.237393	1	0.626095542	1
Gdist:S16260_2531	3.941442	1	0.047110391	0.826214689
Gdist:S163033_15169	2.384512	1	0.122543175	0.923049505
Gdist:S163033_16042	0.616884	1	0.43220758	1
Gdist:S16320_4528	0.029875	1	0.862774801	1
Gdist:S163404_8754	1.061568	1	0.302857878	0.984691538
Gdist:S163875_5661	0.060667	1	0.805445105	1
Gdist:S163921_1612	0.01276	1	0.910062963	1
Gdist:S163921_4754	1.389312	1	0.238521321	1
Gdist:S164265_4478	2.026828	1	0.154542822	0.923049505

Gdist:S164572_6264	0.862686	1	0.352988119	1
Gdist:S164621_6398	0.164823	1	0.684754764	1
Gdist:S164686_8361	0.044538	1	0.832855744	1
Gdist:S164995_6490	0.095191	1	0.757678836	1
Gdist:S165349_3716	1.287384	1	0.256530522	0.961678135
Gdist:S16554_8200	1.080373	1	0.298614153	0.996192022
Gdist:S165611_4703	0.139482	1	0.708796359	1
Gdist:S165667_8094	0.037463	1	0.846525668	1
Gdist:S1657_6099	1.970189	1	0.160427761	0.948045845
Gdist:S165925_1807	0.04096	1	0.839615679	1
Gdist:S166005_2655	0.594814	1	0.440563428	0.996192022
Gdist:S166211_6461	1.246883	1	0.264148739	0.996192022
Gdist:S166346_1746	1.953562	1	0.162203488	0.923049505
Gdist:S16642_4685	2.12925	1	0.144511444	0.930719512
Gdist:S167399_7696	2.250332	1	0.133585725	0.923049505
Gdist:S167755_9411	13.7081	1	0.000213532	0
Gdist:S167909_2010	0.028032	1	0.867033555	1
Gdist:S16798_5659	2.082239	1	0.149021479	0.923049505
Gdist:S16798_7124	0.791524	1	0.37363976	1
Gdist:S168408_3259	0.6108	1	0.434486832	1
Gdist:S16868_3818	1.222369	1	0.268896211	0.996192022
Gdist:S16868_7515	0.351442	1	0.553297877	1
Gdist:S169407_3124	2.04382	1	0.152825489	0.936024096
Gdist:S169407_6947	0.367957	1	0.544119886	1
Gdist:S169819_444	0.323609	1	0.569446934	1
Gdist:S16994_6368	3.775057	1	0.052022115	0.845153247
Gdist:S170062_4086	2.667412	1	0.10242248	0.915683241
Gdist:S170073_6407	7.473815	1	0.006260277	0.562461538
Gdist:S170431_3226	0.004179	1	0.94845568	1
Gdist:S170431_7731	4.136384	1	0.041970739	0.787240876
Gdist:S170623_5256	2.836627	1	0.092137427	0.914
Gdist:S170810_3416	0.331413	1	0.5648283	1
Gdist:S17128_3728	0.060667	1	0.805445105	1
Gdist:S171555_4291	0.240783	1	0.623641214	1
Gdist:S171660_5313	2.563533	1	0.109353946	0.910498084
Gdist:S171660_5453	1.121061	1	0.28969003	0.996192022
Gdist:S171753_8933	2.248295	1	0.133761687	0.930719512
Gdist:S171839_7107	1.003088	1	0.316564458	0.996192022
Gdist:S171891_6913	0.73613	1	0.390903536	0.992434326
Gdist:S172474_5732	0.037463	1	0.846525668	1
Gdist:S172613_436	0.055815	1	0.813237214	1
Gdist:S172697_6405	0.165584	1	0.684066729	1
Gdist:S172697_6486	5.50E-05	1	0.994082501	1
Gdist:S173197_2357	4.120735	1	0.042360681	0.787240876
Gdist:S17383_7730	1.119053	1	0.290122559	0.996192022
Gdist:S174870_5795	4.496307	1	0.033968145	0.707612903
Gdist:S174880_7332	0.746218	1	0.387676437	1
Gdist:S175766_1990	2.293677	1	0.129901865	0.914

Gdist:S175989_2428	0.983004	1	0.321458216	0.996192022
Gdist:S17607_5698	0.25402	1	0.614258688	1
Gdist:S176224_5944	1.292438	1	0.255599094	0.968970438
Gdist:S176592_1387	0.178355	1	0.672790584	1
Gdist:S176673_5059	0.000819	1	0.977166193	1
Gdist:S176939_7530	0.093993	1	0.759161373	1
Gdist:S177676_2877	4.422204	1	0.035474194	0.712451282
Gdist:S178087_3099	2.823989	1	0.092865324	0.873196429
Gdist:S178165_3664	0.129802	1	0.718637367	1
Gdist:S178183_1939	0.423669	1	0.515111937	1
Gdist:S178241_7465	0.421025	1	0.516426161	1
Gdist:S17879_4184	0.713339	1	0.398337651	0.996192022
Gdist:S178796_525	0.006109	1	0.937698776	1
Gdist:S178882_7836	0.610838	1	0.434472507	1
Gdist:S179492_4653	0.237942	1	0.625696306	1
Gdist:S179805_2544	0.336095	1	0.562092272	1
Gdist:S180470_7370	0.82374	1	0.364089027	0.996192022
Gdist:S18053_3928	0.557107	1	0.455428156	1
Gdist:S1807_2321	1.297989	1	0.254580841	0.996192022
Gdist:S1807_5747	1.680682	1	0.194833891	0.957294737
Gdist:S181174_9540	1.446224	1	0.229134833	0.97046332
Gdist:S181325_6555	0.147811	1	0.700636543	1
Gdist:S181338_3734	0.096711	1	0.75581256	1
Gdist:S181347_7161	0.845186	1	0.357917702	0.996192022
Gdist:S181927_4963	0.007094	1	0.932876979	1
Gdist:S18241_9917	1.069475	1	0.301063973	0.996192022
Gdist:S182431_6153	0.843814	1	0.35830825	0.996192022
Gdist:S182612_1853	2.812375	1	0.093539815	0.873196429
Gdist:S18533_2699	0.62947	1	0.427549962	1
Gdist:S185339_7770	3.948312	1	0.046918398	0.747448889
Gdist:S185352_7387	3.669321	1	0.055422745	0.826214689
Gdist:S18537_5068	0.06859	1	0.793400898	1
Gdist:S18609_8176	1.408946	1	0.235231458	0.97046332
Gdist:S186377_2059	0.263612	1	0.60764975	1
Gdist:S186751_2975	0.498577	1	0.480125871	1
Gdist:S18707_6553	0.793873	1	0.372931436	1
Gdist:S187098_14972	1.038735	1	0.308115769	0.996192022
Gdist:S187130_2080	0.408965	1	0.522495195	1
Gdist:S187369_5006	0.046927	1	0.828500139	1
Gdist:S187440_8357	0.276866	1	0.598763055	1
Gdist:S18797_6393	0.102554	1	0.748786086	1
Gdist:S187993_3785	3.565064	1	0.059007459	0.819448276
Gdist:S18807_5080	0.045835	1	0.830475212	1
Gdist:S188165_4687	1.512903	1	0.218696605	0.98872956
Gdist:S188311_7585	0.237393	1	0.626095542	1
Gdist:S188325_1153	0.318819	1	0.572318349	1
Gdist:S188492_4634	0.419716	1	0.517078617	1
Gdist:S188677_4401	1.59E-05	1	0.99682218	1

Gdist:S188677_7042	0.445614	1	0.50442514	1
Gdist:S1887_3917	2.031334	1	0.1540853	0.959095066
Gdist:S189002_716	0.143344	1	0.704978554	1
Gdist:S189316_5901	0.537313	1	0.463548265	1
Gdist:S18950_668	0.155523	1	0.693312071	1
Gdist:S189901_5111	2.284217	1	0.130696079	0.873196429
Gdist:S19007_438	1.497864	1	0.221000388	0.959095066
Gdist:S19011_5711	0.155523	1	0.693312071	1
Gdist:S190289_2144	0.059263	1	0.807663992	1
Gdist:S190352_2988	0.001059	1	0.974044128	1
Gdist:S190352_7424	1.209712	1	0.271389265	0.968970438
Gdist:S190364_6817	0.551057	1	0.457886218	1
Gdist:S190771_3201	0.017468	1	0.894852073	1
Gdist:S190898_3366	0.524603	1	0.468884852	1
Gdist:S190952_6693	0.36196	1	0.547419559	1
Gdist:S191174_5281	0.000414	1	0.983775114	1
Gdist:S191480_3388	0.828041	1	0.362839698	0.996192022
Gdist:S19151_10137	0.048449	1	0.825785233	1
Gdist:S192776_7473	1.851284	1	0.173634136	0.959095066
Gdist:S192779_5715	0.28754	1	0.591801434	1
Gdist:S193033_8543	0.490614	1	0.48365372	1
Gdist:S193081_187	4.596013	1	0.032046392	0.768067227
Gdist:S193081_638	1.800932	1	0.179599916	0.959095066
Gdist:S193124_6201	0.001777	1	0.966377785	1
Gdist:S193142_8558	0.469229	1	0.493342003	1
Gdist:S193269_2840	0.28754	1	0.591801434	1
Gdist:S19339_4724	0.26989	1	0.60340581	1
Gdist:S193527_292	0.119847	1	0.729200358	1
Gdist:S193534_15221	1.128098	1	0.288181305	0.984691538
Gdist:S193534_15834	0.028589	1	0.865732318	1
Gdist:S193538_1239	0.086219	1	0.76904005	1
Gdist:S19359_475	0.876718	1	0.349102296	0.996192022
Gdist:S193883_6069	1.164194	1	0.280597392	0.996192022
Gdist:S19398_5530	0.16629	1	0.683429997	1
Gdist:S194107_4544	2.475784	1	0.115611766	0.900862423
Gdist:S19455_7330	1.229377	1	0.26752817	1
Gdist:S194915_5990	3.093119	1	0.07862395	0.826214689
Gdist:S195249_14487	3.073688	1	0.079568786	0.910498084
Gdist:S195276_11612	0.926605	1	0.33574667	0.996192022
Gdist:S195477_14645	0.63687	1	0.424846402	1
Gdist:S195734_1812	0.012173	1	0.912146246	1
Gdist:S196082_3762	0.331413	1	0.5648283	1
Gdist:S196369_3759	1.15E-05	1	0.997292869	1
Gdist:S196777_4226	1.141283	1	0.285381484	0.996192022
Gdist:S196777_6053	0.000278	1	0.986697003	1
Gdist:S196825_5115	0.16694	1	0.682845931	1
Gdist:S197557_3245	0.814186	1	0.366885655	1
Gdist:S19804_820	0.028032	1	0.867033555	1

Gdist:S198389_5261	0.966289	1	0.325607401	0.996192022
Gdist:S198448_9718	0.850625	1	0.356375623	1
Gdist:S198580_2128	0.006972	1	0.933454814	1
Gdist:S198740_10984	1.281462	1	0.257627341	0.996192022
Gdist:S199319_5001	2.726589	1	0.098689863	0.886061135
Gdist:S199462_1772	0.002374	1	0.961140574	1
Gdist:S199474_7751	0.184104	1	0.667870035	1
Gdist:S20056_5467	0.003352	1	0.953832145	1
Gdist:S20071_2696	0.134165	1	0.714152045	1
Gdist:S200793_5657	127.4018	1	0	0
Gdist:S200793_7519	0.436652	1	0.508742694	1
Gdist:S200959_6679	0.135583	1	0.712711825	1
Gdist:S201016_6814	0.121363	1	0.727560585	1
Gdist:S201164_7496	1.821528	1	0.177131482	0.961439446
Gdist:S201640_7514	0.690725	1	0.405918048	1
Gdist:S201640_7954	1.738458	1	0.18733437	0.937295352
Gdist:S20172_3792	0.325302	1	0.568438439	1
Gdist:S20175_4137	2.08782	1	0.148477822	0.930719512
Gdist:S20202_6726	7.716805	1	0.005470914	0.421846154
Gdist:S202358_6188	0.754096	1	0.385182437	1
Gdist:S202449_8644	3.07432	1	0.079537839	0.849373737
Gdist:S202467_5791	0.002374	1	0.961140574	1
Gdist:S202467_7454	0.024194	1	0.876392996	1
Gdist:S202603_7315	0.922131	1	0.336915963	0.996192022
Gdist:S203070_5914	0.170225	1	0.679912131	1
Gdist:S203203_4789	2.887961	1	0.089243815	0.852150376
Gdist:S203283_4061	0.779613	1	0.377259486	0.996192022
Gdist:S203333_2297	0.154657	1	0.694123695	1
Gdist:S203343_7428	2.761474	1	0.096559221	0.873196429
Gdist:S203400_2557	0.037463	1	0.846525668	1
Gdist:S204043_6591	0.007774	1	0.92974164	1
Gdist:S204343_605	1.727608	1	0.188716692	0.959095066
Gdist:S20440_2687	0.23373	1	0.628771561	1
Gdist:S204465_14058	0.038474	1	0.844495343	1
Gdist:S204483_4329	0.40262	1	0.525739254	1
Gdist:S20598_970	0.284595	1	0.593704883	1
Gdist:S206123_9204	0.547693	1	0.459261833	1
Gdist:S206726_2476	0.722132	1	0.395445356	0.996192022
Gdist:S206753_14733	1.153524	1	0.282812628	1
Gdist:S20722_9954	0.008302	1	0.927399546	1
Gdist:S208103_1413	0.12511	1	0.723557064	1
Gdist:S208103_3697	0.057798	1	0.810010481	1
Gdist:S208103_7211	0.000379	1	0.984474824	1
Gdist:S208103_9047	0.035507	1	0.850537792	1
Gdist:S208396_4887	1.271075	1	0.259565139	0.98872956
Gdist:S208424_7748	0.091114	1	0.762765125	1
Gdist:S208592_5727	0.101494	1	0.750044542	1
Gdist:S209528_9132	2.892568	1	0.088988961	0.915683241

Gdist:S210000_6349	6.41E-05	1	0.993611253	1
Gdist:S210047_7592	1.71197	1	0.190729968	0.960075029
Gdist:S210273_6426	0.027254	1	0.868873775	1
Gdist:S210417_5705	0.777406	1	0.377935649	0.996192022
Gdist:S210574_9971	0.216047	1	0.642068659	1
Gdist:S210723_7642	0.980208	1	0.32214749	0.996192022
Gdist:S211063_4803	0.3716	1	0.542133665	1
Gdist:S211150_4377	2.007318	1	0.156541868	0.996192022
Gdist:S211349_7251	0.983004	1	0.321458216	0.996192022
Gdist:S211349_7837	1.024562	1	0.311439295	0.996192022
Gdist:S212110_1152	0.295205	1	0.586904065	1
Gdist:S212380_1809	2.05908	1	0.151301447	0.957294737
Gdist:S212541_13018	0.339768	1	0.559963089	1
Gdist:S21291_4318	4.172424	1	0.041086989	0.762314894
Gdist:S21304_7473	2.400401	1	0.121304142	0.912272212
Gdist:S213246_4428	0.776845	1	0.378107643	0.996192022
Gdist:S213275_5909	0.539526	1	0.462629238	1
Gdist:S213275_6315	0.040617	1	0.840279299	1
Gdist:S213566_7710	0.297692	1	0.58533305	1
Gdist:S214130_1540	0.009081	1	0.924080386	1
Gdist:S214559_4353	0.043855	1	0.834122747	1
Gdist:S214687_707	0.429918	1	0.512029306	1
Gdist:S214769_17521	0.007796	1	0.929643111	1
Gdist:S214784_4546	0.665335	1	0.414682731	1
Gdist:S214897_7448	0.81646	1	0.366217207	1
Gdist:S215004_8796	0.093993	1	0.759161373	1
Gdist:S215162_7375	0.659414	1	0.41676691	1
Gdist:S215560_4477	4.749311	1	0.029310028	0.669071895
Gdist:S215628_1616	0.514494	1	0.473199889	1
Gdist:S215773_1111	2.448232	1	0.117657328	0.910498084
Gdist:S215791_11521	1.233924	1	0.266645258	1
Gdist:S216078_1493	0.113687	1	0.735985125	1
Gdist:S216255_8244	1.694019	1	0.193072029	0.959095066
Gdist:S216407_3667	1.001424	1	0.316966255	0.996192022
Gdist:S216680_5599	0.302766	1	0.582153302	1
Gdist:S216916_10552	5.798932	1	0.016035908	0.669071895
Gdist:S216916_439	0.436652	1	0.508742694	0.996192022
Gdist:S216938_2222	0.11936	1	0.729729282	1
Gdist:S217006_2126	0.165092	1	0.684511335	1
Gdist:S217107_2755	11.7481	1	0.000609054	0.152333333
Gdist:S21757_2202	0.074094	1	0.785466226	1
Gdist:S217753_4756	0.048616	1	0.825488538	1
Gdist:S218156_15069	1.599392	1	0.205989433	0.948045845
Gdist:S218170_9435	0.094334	1	0.758737629	1
Gdist:S218263_5586	0.071239	1	0.789541473	1
Gdist:S218335_2397	1.71197	1	0.190729968	0.959095066
Gdist:S218503_6952	2.170633	1	0.140668319	0.93486758
Gdist:S218717_4226	0.240368	1	0.623940336	1

Gdist:S218837_1880	0.682209	1	0.408826991	1
Gdist:S218866_10545	1.109802	1	0.292124932	0.972155745
Gdist:S218976_658	0.677372	1	0.410493107	1
Gdist:S219078_5165	1.764243	1	0.184096168	0.959095066
Gdist:S220267_13534	1.150141	1	0.28351969	0.999306667
Gdist:S220267_14577	0.227421	1	0.633442455	1
Gdist:S220297_5487	0.314091	1	0.575180331	1
Gdist:S220636_231	0.361291	1	0.547790068	1
Gdist:S221163_7658	2.258049	1	0.132921429	0.923049505
Gdist:S221165_2286	1.1144	1	0.291127499	0.961678135
Gdist:S221165_3732	1.074606	1	0.299907465	0.972666047
Gdist:S221182_6229	1.208488	1	0.271631836	0.972666047
Gdist:S221403_1362	3.903947	1	0.048172793	0.826214689
Gdist:S222096_3642	0.043855	1	0.834122747	1
Gdist:S222096_4008	0.325302	1	0.568438439	1
Gdist:S222096_5341	0.054362	1	0.815639457	1
Gdist:S22228_6945	0.257989	1	0.611505071	1
Gdist:S22292_9134	0.159588	1	0.689536188	1
Gdist:S222976_2044	1.704775	1	0.191664653	0.961439446
Gdist:S22348_623	1.875833	1	0.170808555	0.957294737
Gdist:S223544_3397	1.545858	1	0.213747943	0.957294737
Gdist:S223597_6005	0.067722	1	0.794683461	1
Gdist:S22388_1722	0.853578	1	0.355542038	0.996192022
Gdist:S223897_1103	0.113831	1	0.735823606	1
Gdist:S223897_9134	0.814186	1	0.366885655	1
Gdist:S223968_2011	0.081158	1	0.775733591	1
Gdist:S22470_352	1.424008	1	0.232744837	0.964
Gdist:S224720_948	1.547521	1	0.213501743	0.98872956
Gdist:S225060_4090	0.217654	1	0.640832942	1
Gdist:S225079_5366	0.16694	1	0.682845931	1
Gdist:S225224_4710	4.812431	1	0.028255152	0.706272727
Gdist:S22527_8212	5.179902	1	0.022849607	0.706272727
Gdist:S225359_2787	0.071413	1	0.789290499	1
Gdist:S225670_11443	0.191919	1	0.661324459	1
Gdist:S225670_5253	0.163504	1	0.685951103	1
Gdist:S225680_3820	4.40054	1	0.035927563	0.826214689
Gdist:S225852_5032	0.147811	1	0.700636543	1
Gdist:S226200_8143	0.165837	1	0.683837982	1
Gdist:S226687_10648	1.238256	1	0.265807438	0.996192022
Gdist:S226919_5606	0.318019	1	0.572800737	1
Gdist:S226919_5757	4.684109	1	0.030442851	0.706272727
Gdist:S227261_6624	1.492537	1	0.221823198	0.961678135
Gdist:S227373_2228	0.012504	1	0.910964734	1
Gdist:S227567_6835	0.083335	1	0.772828422	1
Gdist:S227855_2687	0.40262	1	0.525739254	1
Gdist:S228061_9109	1.609055	1	0.204624698	0.960075029
Gdist:S228567_2066	0.995876	1	0.318310522	0.996192022
Gdist:S229239_745	1.199139	1	0.273493873	0.996192022

Gdist:S229342_7969	0.012338	1	0.911555487	1
Gdist:S229470_1027	0.057958	1	0.80975224	1
Gdist:S229737_2647	0.706047	1	0.400759091	1
Gdist:S230251_3848	7.187043	1	0.00734319	0.448982456
Gdist:S230251_6244	1.672237	1	0.195959183	0.957294737
Gdist:S230360_12338	0.303532	1	0.581676721	1
Gdist:S23063_484	0.093585	1	0.759667671	1
Gdist:S230797_6341	0.112346	1	0.73748894	1
Gdist:S230797_6745	0.102364	1	0.749011155	1
Gdist:S231537_3739	0.258055	1	0.61145926	1
Gdist:S231756_1430	0.208641	1	0.647834992	1
Gdist:S231756_9169	0.189846	1	0.663045217	1
Gdist:S232218_1291	1.032489	1	0.309574719	1
Gdist:S232218_8803	1.497042	1	0.22112707	0.983407136
Gdist:S232219_5301	1.77801	1	0.182393891	0.936024096
Gdist:S232302_6633	0.437618	1	0.508274257	1
Gdist:S232726_6227	0.022412	1	0.880997194	1
Gdist:S233150_6194	1.061568	1	0.302857878	0.98872956
Gdist:S23355_11612	0.865012	1	0.352339919	1
Gdist:S233650_5483	0.21614	1	0.641997159	1
Gdist:S233810_3810	0.16629	1	0.683429997	1
Gdist:S233895_7485	1.33E-05	1	0.997089594	1
Gdist:S234007_8606	0.254231	1	0.614111513	1
Gdist:S234050_6365	0.017803	1	0.893854153	1
Gdist:S234396_11260	1.24902	1	0.26373976	0.968970438
Gdist:S234649_8466	0.096864	1	0.755625669	1
Gdist:S234653_6232	1.914924	1	0.166417331	0.930719512
Gdist:S235024_3562	0.493671	1	0.482294293	1
Gdist:S235024_4842	0.031085	1	0.860050874	1
Gdist:S235201_7057	0.569598	1	0.450418641	1
Gdist:S235573_978	0.46302	1	0.496215914	1
Gdist:S235970_6941	2.266517	1	0.13219672	0.949004255
Gdist:S236939_9881	4.928798	1	0.026412681	0.700550898
Gdist:S237175_830	0.022341	1	0.881183294	1
Gdist:S237536_4418	1.672378	1	0.195940378	0.960075029
Gdist:S237556_9992	6.511972	1	0.010715063	0.632769231
Gdist:S237603_6472	0.745762	1	0.387821437	1
Gdist:S237828_5646	0.23676	1	0.626555726	1
Gdist:S237988_1589	0.003429	1	0.953303923	1
Gdist:S238521_6414	0.165584	1	0.684066729	1
Gdist:S238568_3355	0.911037	1	0.339839472	0.996192022
Gdist:S238600_11607	1.627529	1	0.202045221	0.989962466
Gdist:S238600_8194	1.460577	1	0.226838366	0.968970438
Gdist:S238631_3607	0.143809	1	0.704523523	1
Gdist:S238908_4593	0.295205	1	0.586904065	1
Gdist:S239095_3364	0.027569	1	0.868125	1
Gdist:S239095_8204	0.012662	1	0.910408316	1
Gdist:S239427_13768	0.945236	1	0.330934786	0.996192022

Gdist:S239427_14418	6.391096	1	0.011469422	0.562461538
Gdist:S23960_7977	0.724633	1	0.394628536	0.996192022
Gdist:S239630_2828	0.99627	1	0.318214851	0.996192022
Gdist:S239787_6673	1.650256	1	0.198924138	0.961678135
Gdist:S239826_12936	0.241319	1	0.623255182	1
Gdist:S239871_4455	0.80111	1	0.370761754	0.996192022
Gdist:S240092_6180	0.300393	1	0.583635967	1
Gdist:S240257_3828	0.314091	1	0.575180331	1
Gdist:S240868_1740	0.165837	1	0.683837982	1
Gdist:S241314_7855	1.346137	1	0.245954645	0.968970438
Gdist:S241514_3572	0.022648	1	0.880375511	1
Gdist:S241572_3967	1.831143	1	0.175992639	0.957294737
Gdist:S241851_1802	2.504915	1	0.113491594	0.90850501
Gdist:S24211_4997	1.110603	1	0.29195088	0.996192022
Gdist:S242633_5556	0.301384	1	0.583016369	1
Gdist:S242933_769	0.141372	1	0.706920569	1
Gdist:S243036_3109	0.909881	1	0.340145886	0.996192022
Gdist:S243036_4817	0.069463	1	0.792120502	1
Gdist:S243104_6653	0.000521	1	0.981790197	1
Gdist:S244785_7396	0.132862	1	0.715482692	1
Gdist:S245191_7381	0.373423	1	0.541144198	1
Gdist:S245527_5778	0.012173	1	0.912146246	1
Gdist:S245652_6615	1.999827	1	0.157317191	0.930719512
Gdist:S245672_3136	0.014903	1	0.902837459	1
Gdist:S245865_2638	0.142181	1	0.706122679	1
Gdist:S245865_7630	7.286225	1	0.006948534	0.628989247
Gdist:S245913_12179	3.565064	1	0.059007459	0.787240876
Gdist:S245913_12990	4.444916	1	0.035005318	0.773676349
Gdist:S246010_10785	1.237697	1	0.265915369	0.968970438
Gdist:S246222_5790	0.258055	1	0.61145926	1
Gdist:S24649_5125	0.896428	1	0.343741318	0.996192022
Gdist:S246604_3421	0.064688	1	0.799234168	1
Gdist:S246913_5166	0.741638	1	0.389136674	1
Gdist:S247256_2512	0.001213	1	0.972214271	1
Gdist:S247269_5485	1.75695	1	0.185005347	0.960075029
Gdist:S247723_4246	0.720702	1	0.395913724	0.996867696
Gdist:S248163_1782	0.591762	1	0.441738389	1
Gdist:S248244_4193	0.165584	1	0.684066729	1
Gdist:S248557_1363	4.710224	1	0.029983743	0.706272727
Gdist:S249254_136	0.517871	1	0.471751077	1
Gdist:S249409_9272	4.622527	1	0.031554722	0.671037975
Gdist:S250380_5981	0.303532	1	0.581676721	1
Gdist:S250380_7179	0.069463	1	0.792120502	1
Gdist:S250383_6156	1.389134	1	0.23855143	0.959095066
Gdist:S2506_6823	0.886251	1	0.346495377	0.999306667
Gdist:S2506_996	4.032905	1	0.044621047	0.762314894
Gdist:S250809_7698	1.024673	1	0.311413224	0.969060241
Gdist:S250874_4561	0.411411	1	0.521254359	1

Gdist:S250874_7670	7.11989	1	0.007623337	0.574514286
Gdist:S25131_8621	3.135562	1	0.07660197	0.845153247
Gdist:S251409_6260	1.444033	1	0.229487746	0.959095066
Gdist:S25145_8223	0.262332	1	0.608522454	1
Gdist:S251464_5874	2.742396	1	0.097718147	0.923049505
Gdist:S25189_7758	0.106631	1	0.744013313	1
Gdist:S252008_4876	1.821528	1	0.177131482	0.965886463
Gdist:S252406_9755	0.0703	1	0.790900949	1
Gdist:S252612_1361	0.48232	1	0.487373545	1
Gdist:S253331_655	0.007637	1	0.930359681	1
Gdist:S253708_7891	0.708191	1	0.40004493	1
Gdist:S253720_4338	3.444985	1	0.063443995	0.787240876
Gdist:S253720_4988	0.023762	1	0.877491647	1
Gdist:S253720_7389	0.026146	1	0.871544058	1
Gdist:S253806_1210	6.522696	1	0.010650642	0.632769231
Gdist:S25408_5243	0.085924	1	0.769424988	1
Gdist:S254141_4306	2.367543	1	0.123881856	0.930719512
Gdist:S254257_11272	0.089898	1	0.764307005	1
Gdist:S255289_12673	0.004566	1	0.946124344	1
Gdist:S255381_1038	0.24545	1	0.620297154	1
Gdist:S255921_1881	3.192852	1	0.073960853	0.852150376
Gdist:S256948_4900	0.324461	1	0.568938753	1
Gdist:S256948_7714	0.818913	1	0.365498081	1
Gdist:S257438_1496	0.008058	1	0.9284722	1
Gdist:S257608_4354	0.9722	1	0.324132133	0.996192022
Gdist:S257608_7713	0.490969	1	0.483495451	1
Gdist:S257634_2635	0.594814	1	0.440563428	0.996192022
Gdist:S258043_6425	0.030275	1	0.861866769	1
Gdist:S258043_7017	0.003861	1	0.950455214	1
Gdist:S258043_9155	0.000364	1	0.984787862	1
Gdist:S25811_2468	0.039593	1	0.842279151	1
Gdist:S258349_7019	0.194233	1	0.659416299	1
Gdist:S258470_3162	0.230274	1	0.6313205	1
Gdist:S258756_5187	6.432031	1	0.011208034	0.595162791
Gdist:S2588_1936	0.001082	1	0.973764239	1
Gdist:S2588_9839	3.186595	1	0.074244526	0.826214689
Gdist:S25947_4810	0.625097	1	0.429159547	0.996192022
Gdist:S259622_1374	0.696532	1	0.403951417	1
Gdist:S259770_6213	1.738458	1	0.18733437	0.948045845
Gdist:S26010_4642	0.038474	1	0.844495343	1
Gdist:S26026_10676	0.004179	1	0.94845568	1
Gdist:S26048_8595	0.953549	1	0.328817627	0.996192022
Gdist:S260802_9469	0.071714	1	0.788857135	1
Gdist:S260824_7668	4.937037	1	0.026287059	0.669071895
Gdist:S260824_9407	1.000764	1	0.317125708	0.992389036
Gdist:S26094_4843	2.049426	1	0.152263602	0.923049505
Gdist:S2610_9039	0.028032	1	0.867033555	1
Gdist:S261360_4638	0.123774	1	0.724976631	1

Gdist:S261547_3634	0.817469	1	0.365921158	0.996192022
Gdist:S261547_6344	4.333641	1	0.037366233	0.787240876
Gdist:S261737_3653	0.007884	1	0.929246075	1
Gdist:S261737_6399	0.149406	1	0.699103783	1
Gdist:S261737_9811	0.406258	1	0.52387473	1
Gdist:S262697_3192	0.106244	1	0.744461687	1
Gdist:S262780_5400	0.816166	1	0.366303421	0.996192022
Gdist:S263264_6501	0.093937	1	0.759230622	1
Gdist:S263519_1867	0.041504	1	0.838568924	1
Gdist:S263705_2977	2.004617	1	0.156820948	0.930719512
Gdist:S264178_335	1.062359	1	0.30267775	0.996192022
Gdist:S264340_5568	1.33E-05	1	0.997089594	1
Gdist:S264367_9420	0.478486	1	0.489109103	1
Gdist:S264488_6320	0.985707	1	0.320793964	0.996192022
Gdist:S264488_9807	0.663194	1	0.415434591	1
Gdist:S264671_8990	1.650256	1	0.198924138	0.961678135
Gdist:S265018_3760	0.000237	1	0.987729905	1
Gdist:S265308_3482	2.511937	1	0.112987013	0.910498084
Gdist:S265937_3238	2.278512	1	0.131177663	0.930719512
Gdist:S265937_4642	0.253728	1	0.614462357	1
Gdist:S26610_2904	0.505038	1	0.477294615	1
Gdist:S26639_1930	0.117653	1	0.731594504	1
Gdist:S26647_1613	2.12925	1	0.144511444	0.912272212
Gdist:S266606_11520	0.272865	1	0.601416585	1
Gdist:S26670_2535	0.594814	1	0.440563428	0.996192022
Gdist:S267347_5574	0.003085	1	0.955703596	1
Gdist:S267826_5318	1.937222	1	0.163970442	0.996192022
Gdist:S26802_3445	0.111966	1	0.737916839	1
Gdist:S268328_4748	0.056065	1	0.812826559	1
Gdist:S26847_11703	0.046927	1	0.828500139	1
Gdist:S268511_6812	0.16629	1	0.683429997	1
Gdist:S269064_2292	3.75831	1	0.052545654	0.826214689
Gdist:S2691_8914	0.214869	1	0.642977627	1
Gdist:S269100_1959	3.430989	1	0.063983761	0.844430446
Gdist:S26919_6612	0.033073	1	0.855693693	1
Gdist:S270087_4390	1.209712	1	0.271389265	0.969060241
Gdist:S270252_7058	3.834102	1	0.050219874	0.826214689
Gdist:S270436_3843	5.104106	1	0.023869276	0.574514286
Gdist:S270675_913	0.240909	1	0.623550297	1
Gdist:S270724_5926	0.222617	1	0.637053248	1
Gdist:S270855_4493	0.484421	1	0.486426601	1
Gdist:S2710_10392	0.363478	1	0.546581201	1
Gdist:S27139_8838	3.770797	1	0.052154773	0.768067227
Gdist:S271622_5595	0.002087	1	0.96356077	1
Gdist:S272268_2840	0.029445	1	0.863755537	1
Gdist:S272921_2755	0.119602	1	0.72946629	1
Gdist:S272921_6816	0.933058	1	0.334069465	0.996192022
Gdist:S273006_5988	0.093093	1	0.760281589	1

Gdist:S273201_5307	0.147605	1	0.700834717	1
Gdist:S273471_3154	1.080373	1	0.298614153	1
Gdist:S273558_1153	2.031334	1	0.1540853	0.949004255
Gdist:S273578_4932	0.454495	1	0.500207881	1
Gdist:S273624_1168	0.139413	1	0.70886563	1
Gdist:S273624_8437	2.758164	1	0.096759225	0.888665245
Gdist:S273682_6131	0.001576	1	0.968331419	1
Gdist:S273721_4337	0.098667	1	0.753434611	1
Gdist:S273721_9684	1.009893	1	0.314928439	1
Gdist:S273904_7270	0.183766	1	0.66815645	1
Gdist:S274636_4816	0.24925	1	0.617603633	1
Gdist:S274931_2188	0.305291	1	0.58058457	1
Gdist:S275209_6204	1.763489	1	0.184189952	0.957294737
Gdist:S275336_5237	0.012173	1	0.912146246	1
Gdist:S275374_7392	0.9722	1	0.324132133	0.996192022
Gdist:S27593_2338	7.164716	1	0.007435144	0.504275862
Gdist:S276066_8390	0.423669	1	0.515111937	1
Gdist:S27650_3735	3.114992	1	0.077574869	0.826214689
Gdist:S276525_2435	1.107687	1	0.292585341	0.97046332
Gdist:S27733_7194	1.326617	1	0.249407964	0.969060241
Gdist:S277830_3921	0.31901	1	0.572203487	1
Gdist:S279403_9793	0.065988	1	0.79727083	1
Gdist:S279403_9877	0.713023	1	0.398442218	1
Gdist:S279953_863	0.033073	1	0.855693693	1
Gdist:S280542_4627	0.741368	1	0.389222872	1
Gdist:S28109_1273	0.314091	1	0.575180331	1
Gdist:S281987_4404	0.440245	1	0.507004197	1
Gdist:S282567_3385	0.589242	1	0.442712292	1
Gdist:S282568_420	0.007796	1	0.929643111	1
Gdist:S282772_7232	1.109802	1	0.292124932	0.98872956
Gdist:S283249_208	2.587978	1	0.107677689	0.890762712
Gdist:S283576_5326	0.536037	1	0.464079626	1
Gdist:S283804_6359	0.191919	1	0.661324459	1
Gdist:S284572_9752	1.810471	1	0.178451738	0.948045845
Gdist:S284585_2743	0.776845	1	0.378107643	0.996192022
Gdist:S284708_3856	0.048616	1	0.825488538	1
Gdist:S284818_5332	0.654141	1	0.418635978	1
Gdist:S285036_8726	0.061524	1	0.80410269	1
Gdist:S285956_7415	0.967746	1	0.325243023	0.996192022
Gdist:S286362_813	0.16629	1	0.683429997	1
Gdist:S286419_10896	3.101004	1	0.078244007	0.873196429
Gdist:S286419_16240	3.529462	1	0.060287361	0.794782609
Gdist:S286836_5084	0.059263	1	0.807663992	1
Gdist:S287246_2980	0.123261	1	0.725524506	1
Gdist:S287518_5794	0.591762	1	0.441738389	1
Gdist:S28933_5728	0.157706	1	0.691277637	1
Gdist:S289480_4758	1.15E-05	1	0.997292869	1
Gdist:S289480_5417	2.946676	1	0.086054373	0.914

Gdist:S289517_3784	0.037313	1	0.846830009	1
Gdist:S289677_7267	2.760074	1	0.096643794	0.873196429
Gdist:S289737_6832	1.080373	1	0.298614153	0.996192022
Gdist:S29013_2139	0.002144	1	0.96307173	1
Gdist:S29013_4784	0.00849	1	0.926584577	1
Gdist:S290130_2222	0.000282	1	0.986611264	1
Gdist:S290667_7179	3.532736	1	0.060168411	0.826214689
Gdist:S290667_8789	1.024673	1	0.311413224	0.969060241
Gdist:S2911_2429	4.136081	1	0.041978247	0.712451282
Gdist:S291969_4425	2.301625	1	0.129238719	0.910498084
Gdist:S29240_1489	0.002341	1	0.961411507	1
Gdist:S292430_2076	0.751595	1	0.385971578	1
Gdist:S292656_2541	0.275047	1	0.599966362	1
Gdist:S292709_6375	0.614843	1	0.432970095	1
Gdist:S292815_3953	1.559672	1	0.211713298	0.959095066
Gdist:S293156_5610	0.023443	1	0.878309564	1
Gdist:S29318_6998	0.913377	1	0.339220084	0.996192022
Gdist:S29320_4825	0.453035	1	0.500897409	1
Gdist:S29350_7487	0.524603	1	0.468884852	1
Gdist:S293746_313	0.009566	1	0.922088354	1
Gdist:S293856_3107	0.360036	1	0.548486172	1
Gdist:S294330_6974	0.759124	1	0.383602878	0.996192022
Gdist:S294436_4194	0.139413	1	0.70886563	1
Gdist:S294436_5767	0.243948	1	0.621369007	1
Gdist:S294436_7649	0.453035	1	0.500897409	1
Gdist:S294476_8206	2.563533	1	0.109353946	0.910498084
Gdist:S294633_6473	0.170225	1	0.679912131	1
Gdist:S29534_6024	0.588867	1	0.442857505	1
Gdist:S295466_3259	0.000414	1	0.983775114	1
Gdist:S297769_5036	0.069349	1	0.792286314	1
Gdist:S297801_1473	0.234883	1	0.627926856	1
Gdist:S29822_10594	2.492557	1	0.114385714	0.923049505
Gdist:S29822_11735	1.048489	1	0.30585526	0.996192022
Gdist:S298727_3047	0.634756	1	0.425616177	1
Gdist:S298799_5433	1.20475	1	0.272374307	0.972666047
Gdist:S299064_4718	0.83287	1	0.361444092	0.996192022
Gdist:S29939_2779	0.266391	1	0.605763052	1
Gdist:S29950_3487	4.12887	1	0.042157485	0.747448889
Gdist:S30004_9738	0.003563	1	0.952401286	1
Gdist:S300381_5583	0.009081	1	0.924080386	1
Gdist:S30075_4166	0.284595	1	0.593704883	1
Gdist:S30075_6826	0.296812	1	0.585888104	1
Gdist:S300815_3696	3.053557	1	0.080560504	0.923049505
Gdist:S30094_10154	0.305291	1	0.58058457	1
Gdist:S30112_3479	11.32314	1	0.000765473	0.152333333
Gdist:S302039_5076	0.682209	1	0.408826991	1
Gdist:S302235_680	1.986925	1	0.158662749	0.930719512
Gdist:S30231_7788	0.931938	1	0.334359773	0.996192022

Gdist:S302602_6741	0.002374	1	0.961140574	1
Gdist:S303669_4492	0.654769	1	0.418412822	0.996867696
Gdist:S303872_6279	0.113717	1	0.735951842	1
Gdist:S304497_6832	0.18651	1	0.665837729	1
Gdist:S304497_9267	0.00652	1	0.935642079	1
Gdist:S30466_5523	1.24902	1	0.26373976	0.968970438
Gdist:S305584_12370	1.679974	1	0.194927951	0.959095066
Gdist:S3060_4779	0.212923	1	0.644486199	1
Gdist:S306037_1444	0.588867	1	0.442857505	1
Gdist:S30627_14083	1.888846	1	0.169332123	0.948045845
Gdist:S306468_5178	0.135583	1	0.712711825	1
Gdist:S306571_3728	0.360791	1	0.548067329	1
Gdist:S306571_4010	0.332683	1	0.56408369	1
Gdist:S306571_6855	2.104804	1	0.146837134	0.930719512
Gdist:S307061_419	0.808578	1	0.368540946	0.996192022
Gdist:S307090_446	0.337201	1	0.561449322	1
Gdist:S307616_5920	0.294361	1	0.58743938	1
Gdist:S30786_2297	0.190206	1	0.662745195	1
Gdist:S307891_8631	0.121363	1	0.727560585	1
Gdist:S30852_5724	0.393034	1	0.530708968	1
Gdist:S308860_8611	0.513592	1	0.473587839	1
Gdist:S308906_15933	0.026014	1	0.871866779	1
Gdist:S30909_14491	1.497864	1	0.221000388	0.959095066
Gdist:S309347_3109	0.201823	1	0.653253171	1
Gdist:S31046_2532	1.798947	1	0.179839877	0.961678135
Gdist:S31082_2578	1.77766	1	0.182436962	0.960075029
Gdist:S311040_2596	1.136355	1	0.286423899	0.992434326
Gdist:S311492_2101	0.093585	1	0.759667671	1
Gdist:S31249_4111	0.129802	1	0.718637367	1
Gdist:S313409_6746	0.690062	1	0.406143385	1
Gdist:S313587_1220	0.946003	1	0.330738688	1
Gdist:S31367_2663	0.205909	1	0.649992779	1
Gdist:S31368_4306	2.580681	1	0.108175103	0.90096945
Gdist:S314660_8149	0.567602	1	0.451213324	1
Gdist:S314813_1830	0.054362	1	0.815639457	1
Gdist:S314969_4922	4.305995	1	0.037978266	0.707612903
Gdist:S315006_6423	1.778503	1	0.182333268	0.948045845
Gdist:S31502_6472	0.366695	1	0.544811308	1
Gdist:S315516_8915	4.816053	1	0.028195834	0.707612903
Gdist:S315826_2961	1.251976	1	0.263175421	0.968970438
Gdist:S31620_6458	1.450966	1	0.228372992	0.969060241
Gdist:S316257_7482	2.327172	1	0.127132675	0.910498084
Gdist:S316844_1627	1.894333	1	0.168713991	0.923049505
Gdist:S316856_7845	0.046927	1	0.828500139	1
Gdist:S317586_2473	0.365006	1	0.545739273	1
Gdist:S318863_3453	0.654105	1	0.418648702	1
Gdist:S318976_2335	0.283908	1	0.594150934	1
Gdist:S319153_3473	0.000364	1	0.984787862	1

Gdist:S319155_3685	1.120814	1	0.289743189	0.992434326
Gdist:S319505_3792	0.000281	1	0.986623142	1
Gdist:S319960_7431	1.607709	1	0.204814086	0.960075029
Gdist:S319965_5624	0.524603	1	0.468884852	1
Gdist:S320083_398	0.743688	1	0.388482039	1
Gdist:S320083_7200	0.438682	1	0.507759287	1
Gdist:S321265_10516	0.297692	1	0.58533305	1
Gdist:S321265_16863	0.9722	1	0.324132133	0.996192022
Gdist:S321426_6372	1.359409	1	0.243639968	0.97046332
Gdist:S321568_747	1.701593	1	0.192079811	0.959095066
Gdist:S321579_2600	0.029445	1	0.863755537	1
Gdist:S321737_7439	1.70697	1	0.191378953	0.959095066
Gdist:S322656_3407	0.000349	1	0.985094077	1
Gdist:S323064_1442	1.047433	1	0.306099124	1
Gdist:S323064_2014	0.372866	1	0.541446017	1
Gdist:S323491_6225	0.790126	1	0.374062122	0.996192022
Gdist:S323962_5726	0.148537	1	0.699937268	1
Gdist:S3254_2158	1.307963	1	0.252763812	1
Gdist:S325586_5759	1.799832	1	0.179732785	0.959095066
Gdist:S326335_5598	3.339682	1	0.067627697	0.826214689
Gdist:S326794_3302	0.720702	1	0.395913724	1
Gdist:S32801_7671	0.314091	1	0.575180331	1
Gdist:S32826_7674	3.12371	1	0.077160937	0.831364384
Gdist:S3295_6437	0.105865	1	0.744901529	1
Gdist:S329816_5156	0.140439	1	0.707844715	1
Gdist:S330683_206	4.16328	1	0.041309326	0.738065728
Gdist:S330683_6886	0.12511	1	0.723557064	1
Gdist:S331269_7144	2.726165	1	0.098716099	0.873196429
Gdist:S331452_3731	3.75831	1	0.052545654	0.787240876
Gdist:S331452_603	1.345163	1	0.246125447	1
Gdist:S331500_6704	1.024673	1	0.311413224	0.972155745
Gdist:S332185_8167	0.462777	1	0.4963288	1
Gdist:S332312_4302	3.552857	1	0.059443009	0.787240876
Gdist:S33243_4672	0.31931	1	0.572022383	1
Gdist:S332628_7004	3.775057	1	0.052022115	0.812444444
Gdist:S332628_939	0.000238	1	0.987678623	1
Gdist:S333284_8105	0.96117	1	0.326892393	0.996192022
Gdist:S333385_6513	0.856924	1	0.354600831	0.996192022
Gdist:S33382_6692	0.025387	1	0.873406972	1
Gdist:S33382_9751	0.305291	1	0.58058457	1
Gdist:S3342_3400	0.009932	1	0.920614792	1
Gdist:S334317_2381	0.904426	1	0.341597712	0.996192022
Gdist:S335579_9253	2.801007	1	0.094205129	0.886540773
Gdist:S335726_9602	1.43297	1	0.231280285	0.960075029
Gdist:S33583_3025	4.113805	1	0.042534573	0.812444444
Gdist:S336760_6723	0.786281	1	0.375227099	0.996192022
Gdist:S337806_15034	1.982932	1	0.159081912	0.94994382
Gdist:S338481_5696	0.237393	1	0.626095542	1

Gdist:S338641_200	0.23676	1	0.626555726	1
Gdist:S339656_1631	0.079012	1	0.778641073	1
Gdist:S339767_9556	0.025432	1	0.873296404	1
Gdist:S339816_3071	0.263666	1	0.607612983	1
Gdist:S339816_4291	5.560601	1	0.018369106	0.782353909
Gdist:S33982_15022	2.96341	1	0.085168176	0.885814978
Gdist:S340130_4102	9.940537	1	0.001616784	0.332363636
Gdist:S34134_2418	0.153717	1	0.695008686	1
Gdist:S341523_5552	2.20767	1	0.137325921	0.959095066
Gdist:S341583_8089	0.774627	1	0.378789371	1
Gdist:S341587_1300	3.882189	1	0.048800888	0.799340502
Gdist:S342053_7249	1.492537	1	0.221823198	0.996192022
Gdist:S342053_9320	1.441024	1	0.22997372	0.972155745
Gdist:S342274_7869	0.341897	1	0.558736431	1
Gdist:S34277_5375	2.266205	1	0.132223353	0.936024096
Gdist:S342944_6951	1.608799	1	0.204660686	0.957294737
Gdist:S343_3623	0.472208	1	0.491972976	0.996192022
Gdist:S343453_2566	1.045264	1	0.306600309	0.996192022
Gdist:S343768_3941	0.043155	1	0.835433269	1
Gdist:S344029_2288	2.735334	1	0.098150975	0.910498084
Gdist:S344029_3210	0.174406	1	0.676224943	1
Gdist:S344200_7042	0.541643	1	0.461752767	1
Gdist:S345100_1033	2.186017	1	0.139269062	0.923049505
Gdist:S345100_7775	0.165837	1	0.683837982	1
Gdist:S34545_1552	0.323609	1	0.569446934	1
Gdist:S345718_1721	3.956077	1	0.046702411	0.741308756
Gdist:S345983_4350	0.367972	1	0.544111874	1
Gdist:S34604_7094	0.744666	1	0.388170337	0.996192022
Gdist:S346042_1237	1.276813	1	0.258492495	0.989962466
Gdist:S346427_10827	3.235646	1	0.07205189	0.837628032
Gdist:S346563_3503	0.086219	1	0.76904005	1
Gdist:S346563_7497	0.092793	1	0.760655663	1
Gdist:S347238_9635	4.697634	1	0.030204167	0.706272727
Gdist:S34865_6360	2.051612	1	0.152045145	0.959095066
Gdist:S349354_1666	0.426901	1	0.51351373	1
Gdist:S349813_6865	3.109557	1	0.077834118	0.826214689
Gdist:S350240_281	0.695985	1	0.404136097	1
Gdist:S350937_1703	0.129802	1	0.718637367	1
Gdist:S351074_4648	0.429523	1	0.512223047	1
Gdist:S352270_3238	0.020404	1	0.88641571	1
Gdist:S352552_6424	2.098224	1	0.147470319	0.957294737
Gdist:S35267_5176	1.830882	1	0.176023462	0.959095066
Gdist:S352773_7123	0.059736	1	0.806913245	1
Gdist:S353767_9113	1.025769	1	0.311154523	0.996192022
Gdist:S35381_6655	0.026511	1	0.870657674	1
Gdist:S353988_9826	0.006803	1	0.934262634	1
Gdist:S354215_5179	0.923648	1	0.336518835	0.996192022
Gdist:S354264_3505	0.133282	1	0.715053324	1

Gdist:S35446_706	0.045835	1	0.830475212	1
Gdist:S354917_679	0.002374	1	0.961140574	1
Gdist:S355068_573	1.010388	1	0.314809924	0.992751958
Gdist:S355641_2300	1.849511	1	0.173840248	0.930719512
Gdist:S35568_4383	17.44287	1	2.96E-05	0
Gdist:S35585_2670	1.679465	1	0.194995567	0.948045845
Gdist:S356641_1485	2.855871	1	0.091040908	0.873196429
Gdist:S356774_6492	0.532217	1	0.465676097	1
Gdist:S35706_2318	0.091114	1	0.762765125	1
Gdist:S35706_2935	0.061385	1	0.804320734	1
Gdist:S35706_5421	0.776845	1	0.378107643	0.996192022
Gdist:S357104_9953	0.027569	1	0.868125	1
Gdist:S357248_9754	1.433248	1	0.231235112	0.97046332
Gdist:S35797_2655	3.114992	1	0.077574869	0.83679564
Gdist:S358285_1092	0.246945	1	0.619234202	1
Gdist:S358300_3992	1.101767	1	0.293878545	0.996192022
Gdist:S359414_5450	0.002055	1	0.963842268	1
Gdist:S359627_16667	0.74534	1	0.387955647	1
Gdist:S360039_2326	0.641465	1	0.423180772	1
Gdist:S360207_16184	25.78261	1	3.82E-07	0.243733333
Gdist:S360207_16392	2.124502	1	0.144959875	0.930719512
Gdist:S36082_17231	0.032106	1	0.857794913	1
Gdist:S361328_4903	0.814186	1	0.366885655	1
Gdist:S361402_1207	0.15537	1	0.693455596	1
Gdist:S361831_2035	0.012169	1	0.912159778	1
Gdist:S361831_4422	0.117648	1	0.731599545	1
Gdist:S36228_3046	0.05288	1	0.81812565	1
Gdist:S362726_3535	0.314091	1	0.575180331	1
Gdist:S362726_6078	3.033724	1	0.081550641	0.831364384
Gdist:S36312_7871	0.002374	1	0.961140574	1
Gdist:S363218_972	0.466699	1	0.494509687	1
Gdist:S363298_10405	0.025943	1	0.872039625	1
Gdist:S36381_5548	0.000206	1	0.988542558	1
Gdist:S36381_5657	0.117648	1	0.731599545	1
Gdist:S364012_6936	0.827609	1	0.362964824	0.996192022
Gdist:S364170_3356	0.076313	1	0.782357478	1
Gdist:S364600_3193	0.003179	1	0.955037387	1
Gdist:S365002_1741	0.368348	1	0.543906281	1
Gdist:S365002_7169	1.835273	1	0.175505943	0.961439446
Gdist:S365002_7483	0.000664	1	0.979448484	1
Gdist:S365106_1019	0.041685	1	0.838220813	1
Gdist:S365944_2332	0.002374	1	0.961140574	1
Gdist:S3660_5193	0.069349	1	0.792286314	1
Gdist:S3660_7291	0.551057	1	0.457886218	1
Gdist:S366036_2568	0.029445	1	0.863755537	1
Gdist:S366051_6089	0.188562	1	0.664116206	1
Gdist:S366479_1931	0.45638	1	0.499320785	1
Gdist:S366614_5034	0.000819	1	0.977166193	1

Gdist:S366614_5874	0.168644	1	0.681319369	1
Gdist:S367447_10254	3.708515	1	0.054135547	0.787240876
Gdist:S368648_4705	0.857523	1	0.354432773	0.996192022
Gdist:S368930_6372	0.000742	1	0.978267909	1
Gdist:S369028_9865	0.037313	1	0.846830009	1
Gdist:S36907_1864	0.390596	1	0.531986543	1
Gdist:S369267_1186	0.717448	1	0.396982315	1
Gdist:S369351_5216	2.716555	1	0.099312165	0.923049505
Gdist:S370999_8329	0.019438	1	0.889116898	1
Gdist:S371335_11038	3.770322	1	0.052169596	0.826214689
Gdist:S37227_7785	0.034968	1	0.851662118	1
Gdist:S37253_4936	0.393034	1	0.530708968	1
Gdist:S372553_4889	0.296468	1	0.586104943	1
Gdist:S372824_1686	1.285925	1	0.256800339	0.969060241
Gdist:S373163_2756	0.024741	1	0.875012922	1
Gdist:S373249_6018	8.707247	1	0.003169475	0.373061224
Gdist:S37327_1351	0.761991	1	0.382706294	0.996192022
Gdist:S373275_5074	3.284731	1	0.069927071	0.886540773
Gdist:S374589_6614	0.733789	1	0.391657798	1
Gdist:S37462_9253	0.388419	1	0.533131355	1
Gdist:S374679_7478	0.727454	1	0.393709782	0.996192022
Gdist:S375094_5419	0.000557	1	0.981175494	1
Gdist:S37571_17888	0.400946	1	0.526600925	1
Gdist:S375841_2581	1.492537	1	0.221823198	0.996192022
Gdist:S375902_4837	2.247808	1	0.133803781	0.902898785
Gdist:S376158_15965	0.001135	1	0.973126044	1
Gdist:S376809_6494	0.923648	1	0.336518835	0.996192022
Gdist:S37688_6595	3.45128	1	0.063202823	0.839240106
Gdist:S3770_2725	1.292438	1	0.255599094	0.97046332
Gdist:S377267_10717	0.029875	1	0.862774801	1
Gdist:S37758_7578	3.00285	1	0.083118192	0.912272212
Gdist:S377637_7617	0.746218	1	0.387676437	1
Gdist:S377665_4790	1.351565	1	0.245004733	0.97046332
Gdist:S378362_8127	5.185043	1	0.022782111	0.706272727
Gdist:S37853_4158	1.942023	1	0.163449001	0.946642857
Gdist:S37877_3561	0.536585	1	0.46385142	1
Gdist:S37890_4546	1.49413	1	0.221576816	0.968970438
Gdist:S379720_1425	1.456402	1	0.227503463	0.996192022
Gdist:S38014_11146	0.059263	1	0.807663992	1
Gdist:S380413_6454	2.37983	1	0.122910909	0.948045845
Gdist:S3809_6423	0.706047	1	0.400759091	1
Gdist:S382649_11164	0.234783	1	0.627999504	1
Gdist:S382659_6762	0.644104	1	0.422228726	1
Gdist:S382672_3438	0.032932	1	0.855996813	1
Gdist:S382672_9810	0.777406	1	0.377935649	0.996192022
Gdist:S383195_199	1.121042	1	0.289694141	0.996192022
Gdist:S38333_12201	0.234688	1	0.628069424	1
Gdist:S383366_10329	0.003179	1	0.955037387	1

Gdist:S383427_1527	1.955783	1	0.161964989	0.957294737
Gdist:S383427_2509	1.156399	1	0.282213535	0.973350649
Gdist:S385256_2310	1.247673	1	0.263997341	0.972666047
Gdist:S38546_7603	0.328116	1	0.566770293	1
Gdist:S38546_8225	0.207001	1	0.649128223	1
Gdist:S38554_6627	2.239072	1	0.134561719	0.930719512
Gdist:S386025_2013	8.707247	1	0.003169475	0.243733333
Gdist:S386025_9401	0.567602	1	0.451213324	1
Gdist:S386457_9386	0.004179	1	0.94845568	1
Gdist:S387653_3590	0.360791	1	0.548067329	1
Gdist:S387818_6438	0.665335	1	0.414682731	1
Gdist:S387822_1001	4.090682	1	0.043120228	0.712451282
Gdist:S388247_1797	0.516943	1	0.472148576	1
Gdist:S38858_5360	0.062672	1	0.802321059	1
Gdist:S388831_8558	2.760074	1	0.096643794	0.873196429
Gdist:S389074_7997	1.443064	1	0.229644154	0.967531453
Gdist:S389681_7893	0.051085	1	0.821185804	1
Gdist:S39020_3067	5.894499	1	0.015188252	0.759688312
Gdist:S390641_3261	0.008406	1	0.926950843	1
Gdist:S391408_5061	0.113431	1	0.736270889	1
Gdist:S391711_1307	7.322777	1	0.006808612	0.332363636
Gdist:S391711_1723	4.914974	1	0.026624868	0.700550898
Gdist:S391822_1236	0.332683	1	0.56408369	1
Gdist:S391822_5338	0.014964	1	0.902640469	1
Gdist:S392774_2824	0.323879	1	0.569285725	1
Gdist:S392774_8533	5.578773	1	0.018179457	0.669071895
Gdist:S39356_11764	0.139482	1	0.708796359	1
Gdist:S39401_4373	0.006491	1	0.935785299	1
Gdist:S394951_3073	0.685873	1	0.407571696	0.996192022
Gdist:S395349_5906	0.536193	1	0.464014804	0.996867696
Gdist:S395870_6048	0.210674	1	0.646239519	1
Gdist:S396961_2735	0.71317	1	0.398393416	0.996192022
Gdist:S397128_4077	0.026014	1	0.871866779	1
Gdist:S39740_1700	0.00142	1	0.969945124	1
Gdist:S398761_3978	0.192775	1	0.660617288	1
Gdist:S399414_6513	0.461793	1	0.496787406	1
Gdist:S400310_5517	1.611049	1	0.20434447	0.968970438
Gdist:S401177_3694	1.806633	1	0.178912648	0.959095066
Gdist:S401824_4448	0.285416	1	0.593173237	1
Gdist:S401824_4858	0.739386	1	0.389857707	1
Gdist:S401841_17519	0.00098	1	0.975026917	1
Gdist:S40349_4848	3.775856	1	0.051997301	0.787240876
Gdist:S403763_2905	0.184104	1	0.667870035	1
Gdist:S403990_5235	5.177891	1	0.022876072	0.669071895
Gdist:S403990_7756	0.139125	1	0.709152928	1
Gdist:S404371_13551	1.059786	1	0.303264059	0.992434326
Gdist:S40452_2363	0.004242	1	0.948071253	1
Gdist:S40452_7717	0.00239	1	0.961007388	1

Gdist:S40469_3688	0.814186	1	0.366885655	1
Gdist:S404817_3473	6.416082	1	0.011309142	0.448982456
Gdist:S40530_5594	1.268913	1	0.259970671	0.98712
Gdist:S406012_5746	0.006491	1	0.935785299	1
Gdist:S407139_1408	0.275646	1	0.599569217	1
Gdist:S407303_5611	0.359769	1	0.548634378	1
Gdist:S40741_4112	0.889855	1	0.34551671	0.996192022
Gdist:S407720_904	0.254663	1	0.613810816	1
Gdist:S40778_1344	1.048489	1	0.30585526	0.972666047
Gdist:S40792_1298	5.775364	1	0.016252353	0.823205298
Gdist:S40792_1868	0.000225	1	0.988020216	1
Gdist:S40792_2940	1.446224	1	0.229134833	0.968970438
Gdist:S408054_4248	0.001253	1	0.971768001	1
Gdist:S408123_8147	5.779151	1	0.016217373	0.669071895
Gdist:S408665_3579	0.24925	1	0.617603633	1
Gdist:S408665_5512	0.093093	1	0.760281589	1
Gdist:S408768_2269	0.139995	1	0.708286402	1
Gdist:S409313_5424	0.093993	1	0.759161373	1
Gdist:S409381_1574	0.739386	1	0.389857707	0.999306667
Gdist:S409619_4086	7.030653	1	0.008012614	0.574514286
Gdist:S41088_4361	0.28754	1	0.591801434	1
Gdist:S410915_8446	0.746218	1	0.387676437	1
Gdist:S411846_4216	0.000936	1	0.975588981	1
Gdist:S41255_20254	0.009081	1	0.924080386	1
Gdist:S413985_4517	0.23577	1	0.627278372	1
Gdist:S414051_9168	0.808578	1	0.368540946	0.996192022
Gdist:S414518_2848	2.267271	1	0.132132388	0.910498084
Gdist:S41465_3741	3.109557	1	0.077834118	0.826214689
Gdist:S41493_624	0.292838	1	0.588407534	1
Gdist:S41629_4955	2.143433	1	0.143181151	0.957294737
Gdist:S416816_5005	0.110302	1	0.739800409	1
Gdist:S41698_10566	0.395172	1	0.529593264	1
Gdist:S41698_6798	0.696352	1	0.404012072	0.999306667
Gdist:S417245_7993	1.40151	1	0.2364709	0.996192022
Gdist:S417245_8819	4.914974	1	0.026624868	0.669071895
Gdist:S417245_9728	0.759124	1	0.383602878	0.996192022
Gdist:S417363_2045	3.654763	1	0.05590911	0.773676349
Gdist:S417363_4651	0.069463	1	0.792120502	1
Gdist:S41747_6292	0.423439	1	0.515225794	1
Gdist:S417689_1190	3.903947	1	0.048172793	0.823205298
Gdist:S417810_2571	4.029495	1	0.04471131	0.747448889
Gdist:S418082_4014	2.241776	1	0.134326606	0.948045845
Gdist:S42000_1205	2.578013	1	0.108357584	0.886540773
Gdist:S420000_6807	0.216898	1	0.641414014	1
Gdist:S420256_1491	0.223134	1	0.636662031	1
Gdist:S420256_7661	0.106631	1	0.744013313	1
Gdist:S420788_3419	0.290251	1	0.590059822	1
Gdist:S42081_3723	0.902387	1	0.342142408	0.996192022

Gdist:S421297_4945	1.312549	1	0.251933651	0.959095066
Gdist:S422860_7473	1.85941	1	0.172692945	0.957294737
Gdist:S422914_5157	0.217817	1	0.640708297	1
Gdist:S423022_8982	2.036832	1	0.153529142	0.915683241
Gdist:S423133_2595	0.575131	1	0.448227249	1
Gdist:S423638_6249	2.336256	1	0.12639297	0.923049505
Gdist:S424236_2957	1.434231	1	0.231075105	0.97046332
Gdist:S424263_9861	0.490969	1	0.483495451	1
Gdist:S424284_8844	0.831256	1	0.361909661	1
Gdist:S424710_1044	0.342498	1	0.558391091	1
Gdist:S424804_4900	0.067594	1	0.794872775	1
Gdist:S425526_3665	0.898015	1	0.343314619	0.972155745
Gdist:S42556_9071	0.093993	1	0.759161373	1
Gdist:S42631_3308	0.713339	1	0.398337651	0.996192022
Gdist:S42647_9352	0.163504	1	0.685951103	1
Gdist:S426986_2583	0.340001	1	0.559828429	1
Gdist:S427034_10898	1.24664	1	0.264195151	0.980549309
Gdist:S427034_13504	3.604481	1	0.057624049	0.787240876
Gdist:S428107_6908	0.018125	1	0.892906388	1
Gdist:S428213_1072	1.125237	1	0.288793539	0.992389036
Gdist:S42832_4896	0.017516	1	0.894709086	1
Gdist:S430300_4960	0.103952	1	0.747137893	1
Gdist:S43041_9676	2.186017	1	0.139269062	0.923049505
Gdist:S430420_2648	0.426901	1	0.51351373	1
Gdist:S430932_7616	2.12925	1	0.144511444	0.923049505
Gdist:S430932_7683	0.089898	1	0.764307005	1
Gdist:S430932_892	0.030275	1	0.861866769	1
Gdist:S4312_3227	0.002144	1	0.96307173	1
Gdist:S4312_7371	0.013099	1	0.908879991	1
Gdist:S431672_3697	0.000742	1	0.978267909	1
Gdist:S431843_874	4.136081	1	0.041978247	0.706961326
Gdist:S432297_2110	3.135562	1	0.07660197	0.831364384
Gdist:S432837_1058	0.117653	1	0.731594504	1
Gdist:S432837_1428	3.363785	1	0.066644725	0.881646018
Gdist:S432899_722	1.660415	1	0.197547361	0.968970438
Gdist:S433022_5017	4.137338	1	0.041947096	0.738065728
Gdist:S433254_6533	0.163582	1	0.685879835	1
Gdist:S43515_4911	2.702537	1	0.100188724	0.866134663
Gdist:S436692_1348	0.438974	1	0.507618169	1
Gdist:S43844_2420	0.291104	1	0.589513746	1
Gdist:S43844_5503	7.647338	1	0.005685615	0.595162791
Gdist:S43880_1857	0.10349	1	0.747680926	1
Gdist:S438844_6753	0.217817	1	0.640708297	1
Gdist:S44003_8707	0.26989	1	0.60340581	1
Gdist:S440775_7145	1.309845	1	0.252422695	0.992751958
Gdist:S441366_7652	0.000753	1	0.978107162	1
Gdist:S441834_13052	0.110302	1	0.739800409	1
Gdist:S441834_7882	0.419974	1	0.516949801	1

Gdist:S442718_4976	2.344397	1	0.125734188	0.923049505
Gdist:S44370_2167	0.003283	1	0.954305582	1
Gdist:S444429_4794	0.147605	1	0.700834717	1
Gdist:S444476_6666	19.37101	1	1.08E-05	0
Gdist:S44486_4530	0.007094	1	0.932876979	1
Gdist:S44486_8674	0.183083	1	0.668736789	1
Gdist:S445228_1192	1.153524	1	0.282812628	1
Gdist:S44660_237	0.034407	1	0.852843527	1
Gdist:S447787_774	6.365766	1	0.011634284	0.58496
Gdist:S44804_4498	0.039593	1	0.842279151	1
Gdist:S448304_5774	1.922631	1	0.165566876	0.964
Gdist:S448320_4210	0.029445	1	0.863755537	1
Gdist:S44854_5620	1.917625	1	0.166118705	0.957294737
Gdist:S449182_5359	2.412859	1	0.120342431	0.902898785
Gdist:S450052_2191	1.061568	1	0.302857878	0.97046332
Gdist:S4510_3280	1.135881	1	0.286524356	0.996192022
Gdist:S45156_1049	0.472208	1	0.491972976	0.996192022
Gdist:S451658_3478	0.777406	1	0.377935649	0.996192022
Gdist:S452616_6055	6.416082	1	0.011309142	0.669071895
Gdist:S452881_2913	0.666188	1	0.414383888	1
Gdist:S453561_5410	0.114681	1	0.734876915	1
Gdist:S453769_2029	1.559672	1	0.211713298	0.968970438
Gdist:S453769_3795	0.652014	1	0.419393454	1
Gdist:S454459_7441	0.289661	1	0.590437681	1
Gdist:S454628_7586	0.12093	1	0.728028461	1
Gdist:S454896_9545	0.0759	1	0.782932195	1
Gdist:S45516_2538	0.484544	1	0.486371374	1
Gdist:S455166_11450	0.014315	1	0.904763184	1
Gdist:S455666_3114	3.768003	1	0.05224198	0.812444444
Gdist:S455666_7064	3.094172	1	0.078573117	0.886061135
Gdist:S455821_3746	0.014903	1	0.902837459	1
Gdist:S45583_3699	0.04255	1	0.836574228	1
Gdist:S455907_4238	0.986815	1	0.320522144	0.996192022
Gdist:S456240_7264	0.002018	1	0.964172371	1
Gdist:S456287_4611	0.639876	1	0.423755797	1
Gdist:S456937_7358	0.350467	1	0.553849112	1
Gdist:S457503_10471	1.217743	1	0.269804041	0.991787234
Gdist:S458609_9395	0.697671	1	0.403567413	1
Gdist:S45895_6931	1.251976	1	0.263175421	0.961439446
Gdist:S45919_9311	1.433248	1	0.231235112	0.97046332
Gdist:S459471_8936	1.038735	1	0.308115769	0.992434326
Gdist:S459930_9324	2.587978	1	0.107677689	0.873196429
Gdist:S46088_3082	0.696532	1	0.403951417	1
Gdist:S461442_4554	0.033625	1	0.854507414	1
Gdist:S461442_9572	0.243948	1	0.621369007	1
Gdist:S462221_6287	0.0225	1	0.880763476	1
Gdist:S46231_7252	0.227434	1	0.633432727	1
Gdist:S463707_2000	0.556879	1	0.455520644	1

Gdist:S46426_6692	6.508984	1	0.010733083	0.595162791
Gdist:S465440_554	0.15583	1	0.693025436	1
Gdist:S465795_6468	0.12141	1	0.727510432	1
Gdist:S4658_6998	1.996742	1	0.157637773	0.948045845
Gdist:S465983_5990	2.258049	1	0.132921429	0.930719512
Gdist:S465983_7394	5.479792	1	0.019237547	0.669071895
Gdist:S46671_7362	0.01436	1	0.904616686	1
Gdist:S46704_5064	0.038474	1	0.844495343	1
Gdist:S46704_6509	0.81677	1	0.366126218	1
Gdist:S467063_2379	0.855215	1	0.355081201	0.996192022
Gdist:S467860_477	0.583963	1	0.444762951	0.996192022
Gdist:S46787_12272	0.062672	1	0.802321059	1
Gdist:S46831_3287	0.076765	1	0.781730875	1
Gdist:S469005_1441	0.19822	1	0.65616162	1
Gdist:S46966_11604	0.000837	1	0.976913946	1
Gdist:S46966_9492	2.685681	1	0.101253932	0.923049505
Gdist:S46974_4883	0.323879	1	0.569285725	1
Gdist:S46974_8627	1.288659	1	0.256295175	0.97046332
Gdist:S470738_2345	0.634756	1	0.425616177	1
Gdist:S470803_5758	1.764243	1	0.184096168	0.958322412
Gdist:S471295_2952	2.515136	1	0.112757895	0.873196429
Gdist:S471403_1321	0.426901	1	0.51351373	1
Gdist:S471403_6714	0.82374	1	0.364089027	0.996192022
Gdist:S471566_203	3.488183	1	0.061808408	0.826214689
Gdist:S47214_7025	0.366695	1	0.544811308	1
Gdist:S47229_2227	1.767231	1	0.18372508	0.966711643
Gdist:S47297_805	4.885662	1	0.027080641	0.677037037
Gdist:S473281_6421	5.976814	1	0.014495163	0.669071895
Gdist:S47346_3449	0.03957	1	0.842324116	1
Gdist:S475070_6546	0.173646	1	0.676891367	1
Gdist:S475151_5761	0.016362	1	0.898218481	1
Gdist:S475716_8639	0.041685	1	0.838220813	1
Gdist:S475841_1584	1.202685	1	0.272785641	0.978984303
Gdist:S475841_9570	0.425325	1	0.514291949	1
Gdist:S475860_1835	0.102163	1	0.749249255	1
Gdist:S475860_5599	1.410814	1	0.234921275	0.973350649
Gdist:S47588_4768	0.004479	1	0.946639806	1
Gdist:S476075_6822	0	1	1	1
Gdist:S476304_2983	0.713339	1	0.398337651	0.996192022
Gdist:S476440_1325	0.943025	1	0.331501047	0.996192022
Gdist:S476718_7908	1.622309	1	0.202770064	0.969060241
Gdist:S477153_7382	0.685323	1	0.407759611	1
Gdist:S477174_2979	0.336095	1	0.562092272	1
Gdist:S47976_3194	14.58846	1	0.000133731	0
Gdist:S479839_14426	4.365904	1	0.036665029	0.773676349
Gdist:S480407_8341	4.363797	1	0.036710402	0.839240106
Gdist:S48132_4545	15.64316	1	7.65E-05	0.332363636
Gdist:S48218_2914	1.515704	1	0.218270788	0.958322412

Gdist:S48219_2891	0.001135	1	0.973126044	1
Gdist:S482727_4666	2.685681	1	0.101253932	0.912272212
Gdist:S482911_4853	0.97922	1	0.322391562	0.996192022
Gdist:S484971_4342	0.702739	1	0.401864893	1
Gdist:S485238_4860	1.492537	1	0.221823198	0.97046332
Gdist:S48546_6758	2.538734	1	0.111083854	0.923049505
Gdist:S487573_3291	0.078163	1	0.779803117	1
Gdist:S487573_6219	0.895732	1	0.343928589	0.996192022
Gdist:S4888_7404	0.456142	1	0.499432768	1
Gdist:S4888_7757	0.001521	1	0.968895185	1
Gdist:S489500_6605	2.760074	1	0.096643794	0.873196429
Gdist:S490061_6098	2.004617	1	0.156820948	0.948045845
Gdist:S490411_1398	0.395172	1	0.529593264	1
Gdist:S490487_193	1.331904	1	0.248466747	0.972666047
Gdist:S491469_9222	0.053295	1	0.817426631	1
Gdist:S491611_7462	0.479922	1	0.488457707	1
Gdist:S49171_6318	0.71317	1	0.398393416	0.996192022
Gdist:S491791_12029	0.647102	1	0.421150573	1
Gdist:S492181_3329	0.896794	1	0.343642881	1
Gdist:S492181_7981	1.208292	1	0.271670717	0.972666047
Gdist:S49275_688	1.937222	1	0.163970442	0.996192022
Gdist:S493401_2830	2.729677	1	0.098499182	0.881646018
Gdist:S493401_8841	8.343171	1	0.003871396	0.562461538
Gdist:S49472_3963	0.032963	1	0.855930356	1
Gdist:S495400_7369	1.080373	1	0.298614153	1
Gdist:S49554_6131	0.00041	1	0.983836956	1
Gdist:S49720_507	0.540664	1	0.462157622	1
Gdist:S497351_5246	0.232428	1	0.629729337	1
Gdist:S497554_1269	1.250051	1	0.263542795	0.991787234
Gdist:S497554_3191	0.591132	1	0.441981746	0.999094441
Gdist:S497709_1211	0.059263	1	0.807663992	1
Gdist:S497918_6157	0.030712	1	0.860883717	1
Gdist:S49826_3445	14.79489	1	0.00011986	0.152333333
Gdist:S498517_12927	0.147811	1	0.700636543	1
Gdist:S49874_6547	0.16629	1	0.683429997	1
Gdist:S49874_900	0.108084	1	0.742335543	1
Gdist:S499800_4765	1.172612	1	0.278865202	0.984691538
Gdist:S50172_4002	4.136384	1	0.041970739	0.787240876
Gdist:S50184_2171	2.12925	1	0.144511444	0.923049505
Gdist:S502119_5464	0.059701	1	0.806968367	1
Gdist:S502119_7179	1.067256	1	0.301566131	0.992434326
Gdist:S503196_3759	0.00166	1	0.967499865	1
Gdist:S503196_7657	2.552088	1	0.110148619	0.923049505
Gdist:S50345_4157	0.026438	1	0.870836033	1
Gdist:S50383_2209	0.071702	1	0.788874323	1
Gdist:S504912_4595	0.921056	1	0.337197791	0.996192022
Gdist:S505085_7507	1.256572	1	0.262300884	0.968970438
Gdist:S505268_3066	0.422589	1	0.515647852	1

Gdist:S50605_5056	0.36646	1	0.544940002	1
Gdist:S50778_1623	1.448486	1	0.228771072	0.97046332
Gdist:S508336_3453	0.159708	1	0.689425517	1
Gdist:S508336_3668	1.386949	1	0.238920999	0.996192022
Gdist:S509178_1219	1.274613	1	0.258903033	0.97046332
Gdist:S509402_8608	0.191748	1	0.66146592	1
Gdist:S509470_3792	0.395382	1	0.529483964	1
Gdist:S509470_6040	0.006633	1	0.935087032	1
Gdist:S50994_9770	0.171128	1	0.679111156	1
Gdist:S50999_2944	0.003085	1	0.955703596	1
Gdist:S5107_7083	0.541643	1	0.461752767	1
Gdist:S51105_3284	5.198257	1	0.022609544	0.700550898
Gdist:S511299_5345	2.650574	1	0.103512525	0.886061135
Gdist:S51135_6606	0.064471	1	0.799564446	1
Gdist:S51135_6671	0.122381	1	0.726466308	1
Gdist:S511570_3346	0.857523	1	0.354432773	0.996192022
Gdist:S511634_5986	7.024017	1	0.008042361	0.332363636
Gdist:S511835_4059	0.205655	1	0.650194121	1
Gdist:S513060_2174	0.81677	1	0.366126218	0.996192022
Gdist:S51312_7330	0.451729	1	0.501515031	1
Gdist:S51312_7711	0.02664	1	0.870346089	1
Gdist:S513265_4803	0.098667	1	0.753434611	1
Gdist:S51432_4537	2.127394	1	0.144686528	0.923049505
Gdist:S51485_4707	1.285925	1	0.256800339	0.972666047
Gdist:S51485_7388	0.003563	1	0.952401286	1
Gdist:S51520_3916	1.917081	1	0.166178778	0.957294737
Gdist:S516108_8692	1.312549	1	0.251933651	0.960075029
Gdist:S516664_7926	0.768315	1	0.380738801	0.996192022
Gdist:S51737_6399	0.409914	1	0.522013326	1
Gdist:S517371_4139	2.495803	1	0.114150149	0.873196429
Gdist:S517903_9642	1.402595	1	0.236289568	0.972155745
Gdist:S517991_1055	1.784874	1	0.181551962	0.949004255
Gdist:S518013_3513	0.037687	1	0.846072577	1
Gdist:S518013_6627	0.165837	1	0.683837982	1
Gdist:S519320_2282	0.537313	1	0.463548265	1
Gdist:S51962_10556	0.625321	1	0.429076744	0.996867696
Gdist:S520769_6596	0.898015	1	0.343314619	0.968970438
Gdist:S521067_2486	0.18436	1	0.667652609	1
Gdist:S521613_1456	0.034347	1	0.852971196	1
Gdist:S521676_4341	0.165584	1	0.684066729	1
Gdist:S521908_3793	0.066831	1	0.796008342	1
Gdist:S52255_5395	0.497642	1	0.480537898	1
Gdist:S52374_4157	0.505825	1	0.476951587	1
Gdist:S52502_2377	2.266318	1	0.132213662	0.930719512
Gdist:S525671_8405	5.560601	1	0.018369106	0.759688312
Gdist:S525898_1297	0.490969	1	0.483495451	1
Gdist:S52634_4922	0.067066	1	0.795657143	1
Gdist:S52659_1703	1.936069	1	0.164096015	0.930719512

Gdist:S526823_10455	1.141283	1	0.285381484	0.996192022
Gdist:S5274_14182	0.117653	1	0.731594504	1
Gdist:S527567_2278	0.007809	1	0.929581905	1
Gdist:S52880_6577	0.729055	1	0.393189797	1
Gdist:S529069_6981	0.118239	1	0.730951899	1
Gdist:S52932_2025	0.438482	1	0.507855837	1
Gdist:S52932_8715	0.014196	1	0.905160412	1
Gdist:S529500_1136	0.01397	1	0.905914468	1
Gdist:S529519_5242	6.508984	1	0.010733083	0.669071895
Gdist:S529969_6952	1.587074	1	0.207744737	0.961678135
Gdist:S530174_867	4.169872	1	0.041148905	0.799340502
Gdist:S530345_735	0.061524	1	0.80410269	1
Gdist:S53105_9491	0.849011	1	0.356832368	1
Gdist:S53108_2660	4.03808	1	0.044484409	0.812444444
Gdist:S53259_10982	1.473456	1	0.224801216	1
Gdist:S532621_6393	1.85941	1	0.172692945	0.948045845
Gdist:S53374_5251	0.370725	1	0.542609176	1
Gdist:S53541_2096	0.026901	1	0.869718911	1
Gdist:S536265_4046	0.041504	1	0.838568924	1
Gdist:S53668_3653	2.105241	1	0.14679519	0.949004255
Gdist:S53778_2726	0.00283	1	0.957577477	1
Gdist:S538198_2907	1.456402	1	0.227503463	0.996192022
Gdist:S53895_6775	0.147605	1	0.700834717	1
Gdist:S539076_9358	0.931938	1	0.334359773	0.996192022
Gdist:S53951_3539	0.318819	1	0.572318349	1
Gdist:S540008_1839	3.771466	1	0.052133907	0.826214689
Gdist:S540262_7580	0.013575	1	0.907246937	1
Gdist:S54043_5022	0.788235	1	0.374634465	0.996192022
Gdist:S54084_9356	0.113832	1	0.735823136	1
Gdist:S541107_1153	0.728021	1	0.393525297	0.996192022
Gdist:S54123_414	6.698822	1	0.009647663	0.669071895
Gdist:S543123_4907	0.164067	1	0.685439458	1
Gdist:S54358_3770	1.168498	1	0.279710005	0.972666047
Gdist:S5462_9909	0.751753	1	0.385921654	0.996192022
Gdist:S546493_916	0.065988	1	0.79727083	1
Gdist:S546605_603	2.53296	1	0.111490866	0.900862423
Gdist:S546980_1705	1.707847	1	0.191265011	0.957294737
Gdist:S547003_6840	0.445614	1	0.50442514	1
Gdist:S547283_2834	0.22287	1	0.636862108	1
Gdist:S547731_3738	0.061173	1	0.804651284	1
Gdist:S547731_7184	0.100854	1	0.750806963	1
Gdist:S548276_7541	0.641465	1	0.423180772	1
Gdist:S54834_6997	0.696532	1	0.403951417	1
Gdist:S549050_7895	0.035604	1	0.850336141	1
Gdist:S54928_3450	2.545642	1	0.110598922	0.959095066
Gdist:S549721_6289	0.046927	1	0.828500139	1
Gdist:S55012_5914	1.080373	1	0.298614153	0.996192022
Gdist:S551108_5155	8.707247	1	0.003169475	0.373061224

Gdist:S5512_7078	0.254663	1	0.613810816	1
Gdist:S552992_1902	0.035646	1	0.850249308	1
Gdist:S55507_8337	0.428664	1	0.512645146	1
Gdist:S557219_1096	2.227605	1	0.135563823	0.936024096
Gdist:S5573_6262	0.145106	1	0.703257011	1
Gdist:S55808_438	0.022569	1	0.880583457	1
Gdist:S558326_1436	0.894592	1	0.344235873	0.972155745
Gdist:S558937_4668	0.167862	1	0.682018681	1
Gdist:S55915_12399	0.014196	1	0.905160412	1
Gdist:S56030_4839	0.777406	1	0.377935649	0.996192022
Gdist:S561164_2111	0.0759	1	0.782932195	1
Gdist:S56173_1211	0.748759	1	0.386869365	1
Gdist:S562528_1768	1.898942	1	0.168196776	0.960075029
Gdist:S562528_6503	0.040967	1	0.839601586	1
Gdist:S562814_5473	0.184104	1	0.667870035	1
Gdist:S564428_20142	2.805153	1	0.093961862	0.846554707
Gdist:S565001_5794	0.059701	1	0.806968367	1
Gdist:S56520_5345	1.547521	1	0.213501743	0.991787234
Gdist:S56626_7438	1.764243	1	0.184096168	0.959095066
Gdist:S56983_2235	3.742723	1	0.053037945	0.762314894
Gdist:S571194_5965	1.838621	1	0.175112713	0.959095066
Gdist:S571608_4799	0.360036	1	0.548486172	1
Gdist:S571608_843	2.392776	1	0.12189702	0.915683241
Gdist:S57312_1819	0.183766	1	0.66815645	1
Gdist:S57312_4963	0.744666	1	0.388170337	0.996192022
Gdist:S574163_10014	0.035604	1	0.850336141	1
Gdist:S57492_3241	0.060312	1	0.806003392	1
Gdist:S575992_4710	1.707847	1	0.191265011	0.957294737
Gdist:S576229_9129	2.747707	1	0.097394029	0.873196429
Gdist:S57654_6357	4.360188	1	0.036788255	0.78448583
Gdist:S57690_8477	3.284731	1	0.069927071	0.873196429
Gdist:S57705_8304	0.140551	1	0.707733609	1
Gdist:S578094_2124	1.299781	1	0.25425331	0.968970438
Gdist:S5781_4187	0.105681	1	0.745115805	1
Gdist:S580032_7318	0.006068	1	0.937907968	1
Gdist:S58098_2927	0.528604	1	0.467194149	1
Gdist:S58119_5057	1.087091	1	0.29711672	0.996192022
Gdist:S581396_3933	0.239022	1	0.624913276	1
Gdist:S583162_7049	0.094358	1	0.75870837	1
Gdist:S583411_3893	1.579165	1	0.208880983	0.992434326
Gdist:S583414_6553	1.097748	1	0.294760882	0.996192022
Gdist:S58363_10189	0.730974	1	0.392567708	0.996192022
Gdist:S58363_4952	0.112346	1	0.73748894	1
Gdist:S58449_6751	1.884813	1	0.169788093	0.930719512
Gdist:S584933_4695	0.615524	1	0.432715592	1
Gdist:S58534_5795	1.491794	1	0.221938369	0.984691538
Gdist:S58560_1190	0.228679	1	0.632504783	1
Gdist:S58814_4848	0.553633	1	0.456836933	1

Gdist:S58860_5003	0.092793	1	0.760655663	1
Gdist:S58888_4016	0.084203	1	0.771680376	1
Gdist:S58919_3868	0.00239	1	0.961007388	1
Gdist:S58950_9765	20.20365	1	6.96E-06	0
Gdist:S58971_14711	1.907249	1	0.167269202	0.958322412
Gdist:S589859_2320	0.117653	1	0.731594504	1
Gdist:S589859_9693	0.003563	1	0.952401286	1
Gdist:S591863_1145	0.054283	1	0.815771383	1
Gdist:S592287_5141	1.188071	1	0.275719044	0.989962466
Gdist:S592675_2825	1.719127	1	0.189805434	0.961439446
Gdist:S59402_5653	1.229377	1	0.26752817	1
Gdist:S594074_9347	0.034407	1	0.852843527	1
Gdist:S59410_6978	1.902728	1	0.167773287	0.951071329
Gdist:S594889_7588	1.518727	1	0.217812172	0.960075029
Gdist:S594889_7885	0.9722	1	0.324132133	0.996192022
Gdist:S595440_3015	0.415281	1	0.519301143	1
Gdist:S598999_4368	0.153717	1	0.695008686	1
Gdist:S59908_10924	0.641465	1	0.423180772	1
Gdist:S60199_2227	1.58941	1	0.207410421	0.962317181
Gdist:S603069_6325	0.966289	1	0.325607401	0.996867696
Gdist:S604697_6378	0.155523	1	0.693312071	1
Gdist:S60540_5084	0.290251	1	0.590059822	1
Gdist:S605693_1243	1.047433	1	0.306099124	1
Gdist:S60603_1995	2.925373	1	0.087197064	0.873196429
Gdist:S60704_5495	0.175914	1	0.674908846	1
Gdist:S60715_1199	0.314091	1	0.575180331	1
Gdist:S60727_14228	0.81646	1	0.366217207	1
Gdist:S60774_9466	1.936844	1	0.164011568	0.930719512
Gdist:S60782_1920	2.566804	1	0.109127998	0.881646018
Gdist:S607923_3461	0.189846	1	0.663045217	1
Gdist:S609767_3601	2.497293	1	0.114042205	0.912272212
Gdist:S609767_4989	6.698822	1	0.009647663	0.669071895
Gdist:S61025_6654	0.909881	1	0.340145886	0.996192022
Gdist:S610843_1269	3.026875	1	0.081895631	0.837628032
Gdist:S61089_5650	1.803314	1	0.179312307	0.949004255
Gdist:S611098_8555	0.023446	1	0.878302804	1
Gdist:S61132_7845	0.296812	1	0.585888104	1
Gdist:S61145_1101	0.67591	1	0.410998542	1
Gdist:S6117_4696	0.285776	1	0.592940296	1
Gdist:S61195_134	0.827609	1	0.362964824	0.996192022
Gdist:S612164_4378	0.530882	1	0.466236179	1
Gdist:S612707_3758	0.685137	1	0.407823289	1
Gdist:S61343_3133	0.334642	1	0.562938168	1
Gdist:S61644_9984	0.489109	1	0.484325038	1
Gdist:S618113_2649	0.012268	1	0.911806154	1
Gdist:S618113_6185	0.16969	1	0.680387831	1
Gdist:S618718_1504	3.224236	1	0.072555661	0.845153247
Gdist:S620076_398	1.447593	1	0.228914584	0.97046332

Gdist:S620174_5049	0.077127	1	0.781229359	1
Gdist:S620174_7926	0.741769	1	0.389094939	1
Gdist:S622109_7026	0.221175	1	0.638146098	1
Gdist:S62212_1950	1.307963	1	0.252763812	1
Gdist:S62415_1049	0.288078	1	0.591455144	1
Gdist:S6243_5446	0.845186	1	0.357917702	1
Gdist:S6246_5781	0.004405	1	0.947085065	1
Gdist:S62465_8715	1.936844	1	0.164011568	0.930719512
Gdist:S625346_1946	2.841087	1	0.09188202	0.910498084
Gdist:S62543_5457	1.153524	1	0.282812628	1
Gdist:S626031_5038	0.302117	1	0.582558334	1
Gdist:S626117_1457	1.567091	1	0.21063003	0.968970438
Gdist:S626596_2531	0.366695	1	0.544811308	1
Gdist:S62713_5259	0.225163	1	0.635133984	1
Gdist:S6283_3963	0.193536	1	0.659990196	1
Gdist:S62832_3532	1.674189	1	0.195698389	0.948045845
Gdist:S62832_9453	0.103952	1	0.747137893	1
Gdist:S629174_10246	2.704872	1	0.100042095	0.873196429
Gdist:S630971_2254	3.268894	1	0.070605213	0.787240876
Gdist:S63101_5477	2.557901	1	0.109744209	0.910498084
Gdist:S632715_2055	0.078079	1	0.779917438	1
Gdist:S634819_2294	0.102554	1	0.748786086	1
Gdist:S635245_7636	0.589242	1	0.442712292	1
Gdist:S63697_2099	0.200027	1	0.654699262	1
Gdist:S63731_2375	0.788235	1	0.374634465	0.996192022
Gdist:S637640_3321	0.74693	1	0.387449925	0.996192022
Gdist:S638094_1350	0.7787	1	0.377538949	1
Gdist:S64145_603	2.506065	1	0.113408794	0.886540773
Gdist:S64149_15533	0.007887	1	0.929231976	1
Gdist:S64198_4811	0.003759	1	0.951109536	1
Gdist:S642317_1800	2.955572	1	0.085581993	0.873196429
Gdist:S642317_3477	0.035604	1	0.850336141	1
Gdist:S64322_9728	0.419716	1	0.517078617	1
Gdist:S64325_2625	4.655408	1	0.030955875	0.706961326
Gdist:S64325_5158	0.11936	1	0.729729282	1
Gdist:S6438_4812	0.022089	1	0.88185073	1
Gdist:S6441_5182	0.610838	1	0.434472507	1
Gdist:S64444_2675	0.461793	1	0.496787406	1
Gdist:S64444_2754	0.423439	1	0.515225794	1
Gdist:S6450_2371	2.051612	1	0.152045145	0.957294737
Gdist:S64593_7063	0.788235	1	0.374634465	0.996192022
Gdist:S64730_6850	0.020404	1	0.88641571	1
Gdist:S64750_11311	2.038815	1	0.15332903	0.961678135
Gdist:S648683_6096	0.16629	1	0.683429997	1
Gdist:S6488_4748	3.771466	1	0.052133907	0.826214689
Gdist:S6494_4711	0.556879	1	0.455520644	1
Gdist:S6494_964	0.588867	1	0.442857505	1
Gdist:S65166_7728	0.874662	1	0.34966782	0.996192022

Gdist:S65186_1884	4.937037	1	0.026287059	0.669071895
Gdist:S65245_4147	6.428879	1	0.011227941	0.669071895
Gdist:S653836_6436	0.388419	1	0.533131355	1
Gdist:S654478_7872	0.36325	1	0.546706557	1
Gdist:S655076_2933	0.106244	1	0.744461687	1
Gdist:S655608_6910	0.10349	1	0.747680926	1
Gdist:S658098_8466	1.171337	1	0.27912663	0.97046332
Gdist:S65927_7341	0.04096	1	0.839615679	1
Gdist:S660344_1826	1.032093	1	0.309667479	0.996192022
Gdist:S660344_6695	0.62547	1	0.429022015	1
Gdist:S660617_5385	1.563658	1	0.211130488	0.960075029
Gdist:S66089_6737	0.796425	1	0.372164248	0.996192022
Gdist:S663796_2369	0.717984	1	0.396806141	1
Gdist:S665335_338	0.030044	1	0.862390852	1
Gdist:S666501_4977	1.43297	1	0.231280285	0.964
Gdist:S66664_5967	0.501087	1	0.479022863	1
Gdist:S666683_1774	1.292438	1	0.255599094	0.972666047
Gdist:S666683_5670	0.026146	1	0.871544058	1
Gdist:S66714_2881	0.162823	1	0.68657099	1
Gdist:S6676_2498	0.233629	1	0.628846109	1
Gdist:S6684_7743	4.845388	1	0.027720158	0.669071895
Gdist:S66944_1366	0.868036	1	0.351499719	1
Gdist:S669911_6641	0.113687	1	0.735985125	1
Gdist:S669911_6878	0.932135	1	0.334308794	0.996192022
Gdist:S67022_483	0.025432	1	0.873296404	1
Gdist:S67077_5510	0.001271	1	0.971560648	1
Gdist:S67288_4174	0.297215	1	0.585633503	1
Gdist:S67288_5220	2.021648	1	0.155070801	0.923049505
Gdist:S67352_2624	1.735706	1	0.187683765	0.959095066
Gdist:S67374_8016	10.97676	1	0.000922617	0.152333333
Gdist:S67424_8050	1.462894	1	0.226470246	0.991787234
Gdist:S674434_415	0.006109	1	0.937698776	1
Gdist:S67501_7028	0.015458	1	0.901055389	1
Gdist:S67618_2426	0.00142	1	0.969945124	1
Gdist:S677759_774	0.071558	1	0.789081027	1
Gdist:S67978_6355	0.616112	1	0.432495691	1
Gdist:S68084_1916	0.253728	1	0.614462357	1
Gdist:S68113_3175	3.190807	1	0.074053463	0.826214689
Gdist:S68113_4309	0.973253	1	0.323870284	0.992434326
Gdist:S681213_7149	0.005392	1	0.94146452	1
Gdist:S681517_7040	0.538711	1	0.462967477	1
Gdist:S681888_6399	6.41E-05	1	0.993611253	1
Gdist:S682849_7501	1.543609	1	0.214081368	0.972666047
Gdist:S68367_2220	0.285879	1	0.592873744	1
Gdist:S68367_5660	0.525878	1	0.468344842	1
Gdist:S68367_6156	0.057958	1	0.80975224	1
Gdist:S684253_1039	0.148407	1	0.700062128	1
Gdist:S684460_5899	1.77766	1	0.182436962	0.959095066

Gdist:S68597_4677	3.458026	1	0.062945426	0.826214689
Gdist:S68807_7174	0.83388	1	0.361152936	0.999306667
Gdist:S68863_5526	5.166774	1	0.023022937	0.669071895
Gdist:S689160_923	0.301384	1	0.583016369	1
Gdist:S69319_1850	1.152949	1	0.28293267	0.972666047
Gdist:S69367_3982	6.23E-05	1	0.993702497	1
Gdist:S693953_2004	8.119386	1	0.004379439	0.322588235
Gdist:S695422_4610	0.117648	1	0.731599545	1
Gdist:S697184_8164	0.18981	1	0.663074722	1
Gdist:S70340_919	0.380393	1	0.537392973	1
Gdist:S704139_293	10.97676	1	0.000922617	0.243733333
Gdist:S704659_217	0.819936	1	0.36519898	0.996192022
Gdist:S70554_1338	10.03644	1	0.001534732	0.243733333
Gdist:S70554_8796	20.15954	1	7.12E-06	0
Gdist:S70625_3771	0.171128	1	0.679111156	1
Gdist:S70723_7943	0.111775	1	0.738132577	1
Gdist:S70723_8202	0.307866	1	0.578992582	1
Gdist:S70799_3913	2.227605	1	0.135563823	0.930719512
Gdist:S708093_5089	0.472006	1	0.492065871	1
Gdist:S70913_7507	0.420759	1	0.51655871	1
Gdist:S71018_1428	2.290767	1	0.130145594	0.923049505
Gdist:S710217_1748	0.078079	1	0.779917438	1
Gdist:S71066_4535	0.193996	1	0.659611265	1
Gdist:S71172_14636	0.167599	1	0.68225491	1
Gdist:S71188_8148	0.026901	1	0.869718911	1
Gdist:S71390_8982	3.559486	1	0.059206079	0.826214689
Gdist:S7143_5827	0.670133	1	0.41300509	0.996192022
Gdist:S7145_5306	1.140488	1	0.285549293	0.996192022
Gdist:S71493_4035	0.370227	1	0.542880822	1
Gdist:S71571_6516	1.764243	1	0.184096168	0.961678135
Gdist:S72178_6170	0.055641	1	0.813523118	1
Gdist:S72498_4996	0.857679	1	0.354389037	0.996192022
Gdist:S72514_7400	5.037536	1	0.024803746	0.671037975
Gdist:S72550_7757	0.002374	1	0.961140574	1
Gdist:S72581_7641	0.24545	1	0.620297154	1
Gdist:S72843_2519	0.117015	1	0.732294869	1
Gdist:S73077_288	0.000397	1	0.984109887	1
Gdist:S732557_6491	0.147811	1	0.700636543	1
Gdist:S734057_4564	0.000281	1	0.986623142	1
Gdist:S73431_1912	3.583958	1	0.058339994	0.787240876
Gdist:S734588_2445	0.059736	1	0.806913245	1
Gdist:S738908_4158	1.024673	1	0.311413224	0.968970438
Gdist:S73914_2266	0.66118	1	0.416143627	1
Gdist:S73922_1114	0.541643	1	0.461752767	1
Gdist:S73958_5187	2.097831	1	0.147508219	0.948045845
Gdist:S740430_4643	4.069337	1	0.043668399	0.756969163
Gdist:S74204_6794	0.003283	1	0.954305582	1
Gdist:S74445_2466	1.425677	1	0.232471232	0.968970438

Gdist:S744590_3769	0.052385	1	0.818964768	1
Gdist:S7454_7921	0.051429	1	0.820594555	1
Gdist:S74727_1161	0.265321	1	0.6064878	1
Gdist:S74727_1735	0.052826	1	0.818217156	1
Gdist:S748895_789	3.517147	1	0.060736914	0.799340502
Gdist:S74890_9507	0.597463	1	0.439547568	1
Gdist:S749541_5049	0.059263	1	0.807663992	1
Gdist:S75100_906	1.344434	1	0.246253599	0.992389036
Gdist:S7518_5696	1.020401	1	0.312423824	0.996192022
Gdist:S75237_2255	0.102554	1	0.748786086	1
Gdist:S75237_6644	0.016488	1	0.8978288	1
Gdist:S753220_6339	2.301625	1	0.129238719	0.910498084
Gdist:S75610_10060	0.025526	1	0.873064334	1
Gdist:S75685_3966	0.005614	1	0.940271754	1
Gdist:S75720_1907	0.110076	1	0.74005788	1
Gdist:S75770_7431	0.52253	1	0.469764473	1
Gdist:S758275_6493	0.279856	1	0.596795436	1
Gdist:S76057_7808	7.223881	1	0.007194002	0.562461538
Gdist:S76060_6063	0.271959	1	0.602020578	1
Gdist:S7620_3244	6.361578	1	0.011661776	0.623181818
Gdist:S7620_5752	0.057746	1	0.810093922	1
Gdist:S76453_2529	0.000272	1	0.986835668	1
Gdist:S76491_5549	0.000206	1	0.988542558	1
Gdist:S765417_6956	0.478486	1	0.489109103	1
Gdist:S766843_3656	0.035646	1	0.850249308	1
Gdist:S77596_8698	0.36646	1	0.544940002	1
Gdist:S77639_2052	0.290251	1	0.590059822	1
Gdist:S77763_6563	1.934191	1	0.164300622	0.930719512
Gdist:S777928_1666	0.037313	1	0.846830009	1
Gdist:S777928_6280	0.586167	1	0.443905252	1
Gdist:S77986_1331	1.572611	1	0.209828186	0.960075029
Gdist:S7806_9395	3.872205	1	0.049091989	0.823205298
Gdist:S781394_2998	0.112259	1	0.737587266	1
Gdist:S78363_7410	8.635953	1	0.003295928	0.448982456
Gdist:S78363_962	0.024194	1	0.876392996	1
Gdist:S78385_7201	0.001576	1	0.968331419	1
Gdist:S78393_2797	2.992406	1	0.083655805	0.838442667
Gdist:S785195_7288	0.538711	1	0.462967477	1
Gdist:S78525_622	3.626934	1	0.056851476	0.826214689
Gdist:S78690_4943	4.561623	1	0.032696078	0.787240876
Gdist:S78690_7641	0.006491	1	0.935785299	1
Gdist:S7892_6532	0.868036	1	0.351499719	1
Gdist:S78966_7269	0.798343	1	0.371589111	0.996192022
Gdist:S79020_5844	4.254913	1	0.039136958	0.747448889
Gdist:S79028_3191	1.599392	1	0.205989433	0.959095066
Gdist:S79059_1879	2.467256	1	0.11624073	0.912272212
Gdist:S79088_5202	0.820659	1	0.364987591	1
Gdist:S792208_8877	8.390771	1	0.003771308	0.332363636

Gdist:S79262_8781	5.677907	1	0.017179847	0.669071895
Gdist:S7933_3998	1.574273	1	0.209587554	0.968970438
Gdist:S79935_1053	2.174804	1	0.140287343	0.923049505
Gdist:S799448_9673	0.360036	1	0.548486172	1
Gdist:S80178_6161	0.423669	1	0.515111937	1
Gdist:S8036_3287	0.386013	1	0.534402573	1
Gdist:S80500_2065	0.000364	1	0.984787862	1
Gdist:S80512_5184	0.003283	1	0.954305582	1
Gdist:S80623_3543	0.588038	1	0.443178833	1
Gdist:S8065_3229	0.022569	1	0.880583457	1
Gdist:S80706_1302	0.834901	1	0.360859066	0.996192022
Gdist:S808919_7870	0.572842	1	0.449131796	1
Gdist:S8097_6080	1.208488	1	0.271631836	0.976510166
Gdist:S81214_2733	4.854036	1	0.027581526	0.747448889
Gdist:S81377_10143	1.661197	1	0.197441719	0.961439446
Gdist:S81377_9429	0.63961	1	0.423851911	1
Gdist:S815659_6763	4.928798	1	0.026412681	0.669071895
Gdist:S81661_2699	5.695216	1	0.017011218	0.632769231
Gdist:S82024_6472	1.461683	1	0.226662601	0.972115607
Gdist:S8211_2287	0.178355	1	0.672790584	1
Gdist:S82255_10685	0.713358	1	0.398331293	1
Gdist:S82400_4842	1.256572	1	0.262300884	0.969060241
Gdist:S82464_8432	0.388101	1	0.533299106	1
Gdist:S82536_5953	2.967429	1	0.084956778	0.826214689
Gdist:S82706_8842	0.003759	1	0.951109536	1
Gdist:S827396_1282	0.36196	1	0.547419559	1
Gdist:S827396_5437	0.438449	1	0.507871909	1
Gdist:S82769_8758	0.037687	1	0.846072577	1
Gdist:S82886_3406	0.372866	1	0.541446017	1
Gdist:S8305_15065	0.556879	1	0.455520644	1
Gdist:S8305_8664	0.761991	1	0.382706294	0.996192022
Gdist:S830668_2442	0.586167	1	0.443905252	1
Gdist:S83070_5578	1.292438	1	0.255599094	0.959095066
Gdist:S835817_646	0.093937	1	0.759230622	1
Gdist:S83600_6529	4.577207	1	0.032399977	0.738065728
Gdist:S83646_2969	1.292438	1	0.255599094	0.972666047
Gdist:S83796_6059	0.122381	1	0.726466308	1
Gdist:S84103_5394	4.754377	1	0.029223871	0.669071895
Gdist:S84344_3644	0.000213	1	0.988350975	1
Gdist:S8452_3116	1.871908	1	0.171256759	0.930719512
Gdist:S84543_6593	4.432957	1	0.035251385	0.787240876
Gdist:S8497_3287	0.966289	1	0.325607401	0.996192022
Gdist:S85175_3996	0.788235	1	0.374634465	0.996192022
Gdist:S85175_9255	2.247808	1	0.133803781	0.890957983
Gdist:S85275_7034	8.050478	1	0.004549164	0.595162791
Gdist:S85381_4939	0.228454	1	0.632672876	1
Gdist:S85381_7267	0.319694	1	0.571791316	1
Gdist:S85530_308	0.19401	1	0.659599693	1

Gdist:S85530_3792	3.241821	1	0.071780812	0.826214689
Gdist:S85572_6735	0.696468	1	0.403972929	1
Gdist:S85637_8012	2.287689	1	0.130403958	0.94994382
Gdist:S85941_9281	0.061524	1	0.80410269	1
Gdist:S86152_756	5.147401	1	0.023281193	0.706961326
Gdist:S8683_4348	0.774041	1	0.378969848	1
Gdist:S86903_9017	0.072837	1	0.787249575	1
Gdist:S86936_7429	0.886251	1	0.346495377	0.996192022
Gdist:S87311_4815	0.002055	1	0.963842268	1
Gdist:S87415_11622	0.368348	1	0.543906281	1
Gdist:S87651_2888	2.801007	1	0.094205129	0.910498084
Gdist:S87694_5787	0.002374	1	0.961140574	1
Gdist:S87765_2645	1.017756	1	0.313051855	0.996192022
Gdist:S87797_6901	0.559142	1	0.454606	1
Gdist:S87840_7742	0.450724	1	0.501991427	1
Gdist:S88105_3337	0.117648	1	0.731599545	1
Gdist:S88291_1138	2.227605	1	0.135563823	0.930719512
Gdist:S8836_997	0.18116	1	0.670378066	1
Gdist:S88467_9031	0.855215	1	0.355081201	0.996192022
Gdist:S8853_7577	0.026664	1	0.870289603	1
Gdist:S8853_8906	0.227732	1	0.633210806	1
Gdist:S8865_1286	0.042458	1	0.836748416	1
Gdist:S88922_2932	0.105288	1	0.745574071	1
Gdist:S88978_5825	0.164067	1	0.685439458	1
Gdist:S88978_7878	0.347835	1	0.555341273	1
Gdist:S89055_8031	1.60785	1	0.204794289	0.97046332
Gdist:S89159_686	1.389134	1	0.23855143	0.961439446
Gdist:S8928_3592	4.128405	1	0.042169072	0.734784314
Gdist:S8928_5360	0.787682	1	0.374802034	1
Gdist:S89304_4873	0.784897	1	0.37564767	1
Gdist:S8933_3315	0.18981	1	0.663074722	1
Gdist:S89559_2936	0.28754	1	0.591801434	1
Gdist:S89810_12533	0.994823	1	0.318566465	0.996192022
Gdist:S89810_3116	0.240368	1	0.623940336	1
Gdist:S89966_12304	0.505825	1	0.476951587	1
Gdist:S89996_5449	0.150737	1	0.697831937	1
Gdist:S9002_10672	0.183766	1	0.66815645	1
Gdist:S90455_1615	0.241987	1	0.622774507	1
Gdist:S90534_5337	1.246678	1	0.264187978	0.961678135
Gdist:S90602_2446	1.40406	1	0.236044954	0.972155745
Gdist:S90602_5989	4.919376	1	0.026557113	0.738065728
Gdist:S90746_1139	0.155735	1	0.693113873	1
Gdist:S90795_4465	0.026701	1	0.870199818	1
Gdist:S90845_14730	0.440245	1	0.507004197	1
Gdist:S90898_423	0.365006	1	0.545739273	1
Gdist:S90994_2281	0.438449	1	0.507871909	1
Gdist:S91163_7567	1.100636	1	0.294126663	0.996192022
Gdist:S9126_6370	0.10284	1	0.748447885	1

Gdist:S91493_123	0.064688	1	0.799234168	1
Gdist:S91493_4912	0.922131	1	0.336915963	0.996192022
Gdist:S91607_7236	0.002741	1	0.958247068	1
Gdist:S91895_4247	4.352034	1	0.036964765	0.738065728
Gdist:S92266_3866	0.007887	1	0.929231976	1
Gdist:S92280_8106	0.081409	1	0.775397424	1
Gdist:S92323_3399	0.046927	1	0.828500139	1
Gdist:S9249_14269	1.20949	1	0.271433114	0.996192022
Gdist:S9249_2949	1.08262	1	0.298112284	0.968970438
Gdist:S9249_8004	0.029875	1	0.862774801	1
Gdist:S92499_6804	0.363478	1	0.546581201	1
Gdist:S92508_518	0.006633	1	0.935087032	1
Gdist:S9252_10058	0.306591	1	0.579779728	1
Gdist:S92880_6893	0.217817	1	0.640708297	1
Gdist:S93061_4537	0.003352	1	0.953832145	1
Gdist:S93091_14353	0.003085	1	0.955703596	1
Gdist:S9311_6504	2.650574	1	0.103512525	0.908460606
Gdist:S93404_10483	2.468291	1	0.116164141	0.90850501
Gdist:S93637_7164	1.195874	1	0.274147862	0.996192022
Gdist:S93662_250	0.061524	1	0.80410269	1
Gdist:S93662_4606	0.401154	1	0.526493877	1
Gdist:S93760_6902	1.14066	1	0.285512977	0.996192022
Gdist:S94010_4134	2.297772	1	0.129559743	0.923049505
Gdist:S94179_6082	9.826296	1	0.001720344	0.539409836
Gdist:S9435_3306	0.069463	1	0.792120502	1
Gdist:S94519_2437	0.04255	1	0.836574228	1
Gdist:S94536_773	0.169888	1	0.680211017	1
Gdist:S94591_1508	2.727735	1	0.098619042	0.897039175
Gdist:S94599_4328	0.366695	1	0.544811308	1
Gdist:S95102_3370	5.095185	1	0.02399236	0.669071895
Gdist:S9514_7775	1.024673	1	0.311413224	0.968970438
Gdist:S9521_14044	1.279446	1	0.258002091	0.964
Gdist:S95603_3558	0.939114	1	0.332505829	1
Gdist:S95747_5341	1.653294	1	0.198511177	0.960075029
Gdist:S95767_499	2.307011	1	0.128791495	0.923049505
Gdist:S95767_7622	0.022412	1	0.880997194	1
Gdist:S95767_8921	1.152387	1	0.283049995	0.996192022
Gdist:S95941_2611	0.084203	1	0.771680376	1
Gdist:S95972_5107	0.817469	1	0.365921158	0.996192022
Gdist:S96008_3689	0.82686	1	0.363182023	0.996192022
Gdist:S96446_10551	0.893717	1	0.344471946	0.996192022
Gdist:S96522_1841	0.430965	1	0.511515725	1
Gdist:S96583_8444	0.038655	1	0.844134472	1
Gdist:S96590_11234	0.398586	1	0.527820443	1
Gdist:S96636_14046	0.121962	1	0.726915831	1
Gdist:S96636_9765	0.040085	1	0.841315297	1
Gdist:S96659_2257	2.228961	1	0.135444896	0.948045845
Gdist:S96697_4610	0.000212	1	0.988384639	1

Gdist:S96958_3517	0.388101	1	0.533299106	1
Gdist:S96958_980	3.408448	1	0.064863423	0.873196429
Gdist:S97144_4172	0.423669	1	0.515111937	1
Gdist:S97591_1821	0.150272	1	0.698275976	1
Gdist:S97591_2753	0.898015	1	0.343314619	0.98872956
Gdist:S97611_18247	0.973253	1	0.323870284	0.989962466
Gdist:S9769_694	0.124432	1	0.724276699	1
Gdist:S97704_2827	7.064558	1	0.00786237	0.58496
Gdist:S97861_2200	0.066153	1	0.797023262	1
Gdist:S97861_5521	0.032963	1	0.855930356	1
Gdist:S9805_10765	1.619727	1	0.203129917	0.97046332
Gdist:S98235_3484	1.238256	1	0.265807438	0.996192022
Gdist:S98242_7253	0.318019	1	0.572800737	1
Gdist:S98260_9233	0.265321	1	0.6064878	1
Gdist:S98358_10302	0.044126	1	0.833618622	1
Gdist:S98509_1652	1.928892	1	0.16487974	0.937295352
Gdist:S98539_10619	0.443197	1	0.505583244	1
Gdist:S99030_236	0.669525	1	0.413217236	0.996192022
Gdist:S9906_2101	0.004405	1	0.947085065	1
Gdist:S99112_5418	0.154657	1	0.694123695	1
Gdist:S99221_1923	2.544023	1	0.110712401	0.923049505
Gdist:S9963_7098	2.044963	1	0.152710695	0.930719512
Gdist:S99664_3018	0.517042	1	0.4721061	1
Gdist:S99675_8950	0.166759	1	0.683007981	1
Gdist:S99944_3542	0.004491	1	0.946568136	1
Gene:C124177984_226	0.157706	1	0.691277637	1
Gene:C124534064_203	8.806762	1	0.003001162	0.628989247
Gene:S115043_649	2.439599	1	0.118306483	0.923049505
Gene:S118286_2598	0.597463	1	0.439547568	1
Gene:S123339_1421	0.82686	1	0.363182023	0.996192022
Gene:S129840_2011	0.345674	1	0.556572522	1
Gene:S13023_338	0.682209	1	0.408826991	1
Gene:S13230_1136	1.99025	1	0.158314731	0.949004255
Gene:S13230_584	3.304179	1	0.069103863	0.826214689
Gene:S149631_906	0.178099	1	0.673011667	1
Gene:S171022_6436	0.898015	1	0.343314619	0.972155745
Gene:S171637_2011	0.351442	1	0.553297877	1
Gene:S171637_4433	0.370939	1	0.542492973	1
Gene:S171637_7022	3.355309	1	0.066988625	0.844430446
Gene:S172375_1029	1.502735	1	0.220251024	0.97046332
Gene:S175365_5658	27.11251	1	1.92E-07	0.322588235
Gene:S177672_925	0.599853	1	0.438634187	1
Gene:S1971_885	1.802688	1	0.179387842	0.960075029
Gene:S236632_813	1.268511	1	0.260046156	0.996192022
Gene:S237713_1631	0.638282	1	0.424333732	0.996192022
Gene:S237713_2650	2.386671	1	0.122374011	0.910498084
Gene:S240223_3885	2.32874	1	0.127004637	0.902898785
Gene:S268762_268	0.472022	1	0.492058618	1

Gene:S276486_3960	0.071702	1	0.788874323	1
Gene:S281001_1853	0.290701	1	0.589771596	1
Gene:S282703_1456	0.532217	1	0.465676097	1
Gene:S290628_5233	4.570704	1	0.032523199	0.768067227
Gene:S307364_1802	5.020991	1	0.025041833	0.852150376
Gene:S308708_3208	2.718422	1	0.09919601	0.897039175
Gene:S310019_2288	4.413469	1	0.035656265	0.823205298
Gene:S321905_1056	0.003085	1	0.955703596	1
Gene:S322365_1730	2.675402	1	0.101909582	0.896898129
Gene:S346962_803	0.10591	1	0.744849341	1
Gene:S346962_934	4.740015	1	0.029468809	0.734784314
Gene:S35120_486	0.9722	1	0.324132133	0.996192022
Gene:S363330_3277	0.029567	1	0.86347532	1
Gene:S402069_249	0.054362	1	0.815639457	1
Gene:S413536_297	2.342392	1	0.125896128	0.948045845
Gene:S413875_2948	0.014223	1	0.905070851	1
Gene:S445579_467	3.835033	1	0.050192005	0.826214689
Gene:S460136_553	0.200393	1	0.654403775	1
Gene:S47271_1434	0.525315	1	0.468583177	1
Gene:S47271_2967	1.321811	1	0.250267372	0.992434326
Gene:S474031_795	0.02163	1	0.883076919	1
Gene:S512153_3945	1.312549	1	0.251933651	0.968970438
Gene:S523738_1899	3.283633	1	0.069973862	0.826214689
Gene:S525873_108	0.547693	1	0.459261833	1
Gene:S640863_2054	0.048449	1	0.825785233	1
Gene:S640863_2290	0.429918	1	0.512029306	1
Gene:S642560_3839	1.97013	1	0.160434056	0.930719512
Gene:S642560_577	0.006739	1	0.934573024	1
Gene:S667291_1079	0.061524	1	0.80410269	1
Gene:S66998_3416	0.305291	1	0.58058457	1
Gene:S678020_4224	0.131662	1	0.716714837	1
Gene:S695148_1062	0.013121	1	0.90880324	1
Gene:S712373_1350	1.780881	1	0.182041168	0.957294737
Gene:S712373_1710	0.002018	1	0.964172371	1
Gene:S737940_1607	2.284217	1	0.130696079	0.920720588
Gene:S778345_741	1.627529	1	0.202045221	0.97046332
Gene:S787535_1439	0.580939	1	0.445944437	1
Gene:S789955_2465	12.3703	1	0.000436217	0.332363636
Gene:S790412_561	0.099992	1	0.751839238	1
Gene:S799671_712	0.014223	1	0.905070851	1
Gene:S82686_865	1.831691	1	0.175927929	0.996192022
Gene:S830637_471	3.169053	1	0.075045962	0.846554707
Gene:S839123_87	5.665722	1	0.017299596	0.58496
Gene:S92009_2784	1.326489	1	0.249430725	0.97046332
Gene:S93233_2108	0.490969	1	0.483495451	1
LD:S102196_408	1.734678	1	0.187814611	0.969060241
LD:S107235_2148	1.634978	1	0.201015844	0.962317181
LD:S109921_13369	2.152903	1	0.14230061	0.960075029

LD:S109921_874	0.004491	1	0.946568136	1
LD:S110750_883	0.002144	1	0.96307173	1
LD:S126259_12032	1.563658	1	0.211130488	0.959095066
LD:S126259_573	1.080373	1	0.298614153	1
LD:S139051_1298	0.008442	1	0.926792028	1
LD:S139091_1644	2.30307	1	0.129118531	0.923049505
LD:S139091_9797	0.469229	1	0.493342003	1
LD:S139093_3902	0.030275	1	0.861866769	1
LD:S143561_5364	0.227861	1	0.633114105	1
LD:S144765_9154	0.490614	1	0.48365372	1
LD:S146297_6295	0.004328	1	0.947544012	1
LD:S158022_10708	5.304027	1	0.021276183	0.700550898
LD:S158022_2718	0.08511	1	0.770488323	1
LD:S158422_2767	0.024376	1	0.875932416	1
LD:S162605_15211	2.794244	1	0.094603398	0.890762712
LD:S162605_5921	0.10349	1	0.747680926	1
LD:S166532_1813	0.818913	1	0.365498081	1
LD:S166532_9926	0.053295	1	0.817426631	1
LD:S168444_13275	0.043855	1	0.834122747	1
LD:S168444_21932	0.82374	1	0.364089027	0.996192022
LD:S182363_10553	0.395716	1	0.529310091	1
LD:S182363_1448	1.85941	1	0.172692945	0.946642857
LD:S183034_13487	1.619727	1	0.203129917	0.98872956
LD:S183034_3756	0.013906	1	0.906129143	1
LD:S185091_1642	0.18362	1	0.668280247	1
LD:S185091_6658	2.370168	1	0.123673737	0.951071329
LD:S187433_714	4.067052	1	0.043727524	0.734784314
LD:S189168_759	5.536709	1	0.018621566	0.539409836
LD:S189168_9385	1.299127	1	0.254372712	0.97046332
LD:S190550_1832	2.62631	1	0.105105712	0.873196429
LD:S192579_16897	0.067205	1	0.795450372	1
LD:S19938_1411	1.719127	1	0.189805434	0.959095066
LD:S214769_14934	0.94845	1	0.330114057	0.996192022
LD:S214769_4157	0.461793	1	0.496787406	1
LD:S224709_1027	0.279286	1	0.597169888	1
LD:S224709_10589	3.114992	1	0.077574869	0.826214689
LD:S224847_12342	0.061385	1	0.804320734	1
LD:S229656_12348	3.193427	1	0.073934876	0.826214689
LD:S229656_3723	2.901852	1	0.088477842	0.873196429
LD:S240758_5242	2.992406	1	0.083655805	0.873196429
LD:S258756_18380	2.099029	1	0.147392718	0.946642857
LD:S258756_9572	1.972267	1	0.16020739	0.964
LD:S259174_14147	0.0225	1	0.880763476	1
LD:S259174_1840	0.157706	1	0.691277637	1
LD:S269684_1562	0.051278	1	0.82085362	1
LD:S27039_17128	0.930002	1	0.334862324	1
LD:S271068_14525	0.428664	1	0.512645146	1
LD:S271068_3399	0.540382	1	0.462274623	1

LD:S30383_8588	0.001288	1	0.971372636	1
LD:S304413_9219	3.536812	1	0.060020719	0.826214689
LD:S304413_928	0.061608	1	0.803972971	1
LD:S336308_8856	2.092669	1	0.148007255	0.923049505
LD:S38447_13299	1.171945	1	0.279001871	0.989962466
LD:S410327_13451	6.335643	1	0.011833524	0.669071895
LD:S41726_11296	1.530376	1	0.216056026	0.959095066
LD:S429316_11581	0.27943	1	0.597074923	1
LD:S429316_2484	0.237218	1	0.626222779	1
LD:S434590_10049	0.625321	1	0.429076744	1
LD:S434590_1813	0.220815	1	0.638419418	1
LD:S436279_10720	0.017468	1	0.894852073	1
LD:S439680_11105	8.832592	1	0.002958978	0
LD:S45583_14264	0.964658	1	0.326016127	0.996192022
LD:S45583_5902	0.241319	1	0.623255182	1
LD:S47306_2532	0.165584	1	0.684066729	1
LD:S489312_14508	0.034968	1	0.851662118	1
LD:S489312_6068	0.485294	1	0.486033975	1
LD:S546671_1046	4.924706	1	0.026475306	0.734784314
LD:S546671_9492	1.152387	1	0.283049995	0.996192022
LD:S559071_729	0.587453	1	0.443405628	1
LD:S56055_2429	0.136866	1	0.711417194	1
LD:S571533_18083	0.249159	1	0.617667754	1
LD:S57836_13135	0.007796	1	0.929643111	1
LD:S594729_17207	1.522596	1	0.217227033	0.959095066
LD:S656103_13895	0.656109	1	0.417937098	1
LD:S71841_754	0.276866	1	0.598763055	1
LD:S81214_21664	1.142575	1	0.285108878	0.996192022
LD:S81214_3030	3.647778	1	0.056144075	0.823205298
LD:S83132_634	0.288688	1	0.591062825	1
LD:S83132_9406	0.825919	1	0.363455288	0.996192022
LD:S88985_14448	0.159588	1	0.689536188	1
LD:S88985_1626	0.10558	1	0.745233889	1
LD:S96507_10894	0.066629	1	0.796310248	1
LD:S96507_858	0.119602	1	0.72946629	1
SalarSNP:BASS15_B7_G03_7	0.636015	1	0.425157405	1
SalarSNP:EStNV_13128_133	0.237218	1	0.626222779	1
SalarSNP:EStNV_16766_463	4.137338	1	0.041947096	0.712451282
SalarSNP:EStNV_24241_102	0.741368	1	0.389222872	1
SalarSNP:EStNV_25560_563	0.323609	1	0.569446934	1
SalarSNP:EStNV_27243_587	25.78261	1	3.82E-07	0.152333333
SalarSNP:EStNV_30052_103	0.514494	1	0.473199889	1
SalarSNP:EStNV_30276_856	0.000281	1	0.986623142	1
SalarSNP:EStNV_30342_132	0.350218	1	0.553990013	1
SalarSNP:EStNV_30477_550	0.253728	1	0.614462357	1
SalarSNP:EStNV_30479_416	0.059084	1	0.807949034	1
SalarSNP:EStNV_31852_56	0.39588	1	0.529224947	1
SalarSNP:EStNV_32045_280	0.786281	1	0.375227099	0.996192022

SalarSNP:EStNV_32130_141	0.865012	1	0.352339919	0.996192022
SalarSNP:EStNV_32349_576	1.443064	1	0.229644154	0.961678135
SalarSNP:EStNV_32423_381	0.317357	1	0.573200072	1
SalarSNP:EStNV_32552_113	2.233372	1	0.135058829	0.959095066
SalarSNP:EStNV_33050_438	1.200344	1	0.273252836	0.97046332
SalarSNP:EStNV_33260_818	0.091484	1	0.762299439	1
SalarSNP:EStNV_33882_1084	0.03957	1	0.842324116	1
SalarSNP:EStNV_35308_389	0.003084	1	0.955713602	1
SalarSNP:EStNV_35477_857	0.24545	1	0.620297154	1
SalarSNP:EStNV_35796_168	0.445614	1	0.50442514	1
SalarSNP:EStNV_35961_1479	0.153717	1	0.695008686	1
SalarSNP:EStNV_36159_477	0.000238	1	0.987678623	1
SalarSNP:EStNV_36533_796	0.959035	1	0.327430094	1
SalarSNP:EStNV_36818_1287	1.009893	1	0.314928439	1
SalarSNP:EStNV_36887_452	3.201407	1	0.073574959	0.831364384
SalarSNP:EStNV_36918_790	0.489109	1	0.484325038	1
SalarSNP:EStNV_37086_867	0.824295	1	0.363927355	0.996192022
SalarSNP:EStNV_37153_794	1.772595	1	0.183061294	0.960075029
SalarSNP:EStV_13137_137	0.029875	1	0.862774801	1
SalarSNP:EStV_13981_575	1.389134	1	0.23855143	0.959095066
SalarSNP:EStV_14058_333	0.205909	1	0.649992779	1
SalarSNP:EStV_15080_392	0.012918	1	0.909509227	1
SalarSNP:EStV_15243_314	0.303532	1	0.581676721	1
SalarSNP:EStV_15513_116	0.060155	1	0.806251823	1
SalarSNP:EStV_16545_824	1.58014	1	0.208740485	0.967531453
SalarSNP:EStV_17160_114	0.052895	1	0.818100918	1
SalarSNP:EStV_17580_1116	1.520019	1	0.217616632	0.961678135
SalarSNP:EStV_20513_207	2.539132	1	0.111055794	0.897039175
SalarSNP:EStV_21132_1397	0.000717	1	0.978634568	1
SalarSNP:EStV_21544_726	0.192775	1	0.660617288	1
SalarSNP:GCR_cBin11134_C	0.332855	1	0.563983014	1
SalarSNP:GCR_cBin17378_C	0.644104	1	0.422228726	1
SalarSNP:GCR_cBin18664_C	0.00964	1	0.921786892	1
SalarSNP:GCR_cBin18774_C	0.588867	1	0.442857505	1
SalarSNP:GCR_cBin19351_C	1.838621	1	0.175112713	0.996192022
SalarSNP:GCR_cBin23273_C	0.003759	1	0.951109536	1
SalarSNP:GCR_cBin34489_C	6.679201	1	0.009754433	0.669071895
SalarSNP:GCR_cBin43704_C	0.23577	1	0.627278372	1
SalarSNP:GCR_cBin44576_C	0.696532	1	0.403951417	1
SalarSNP:GCR_cBin44942_C	0.276866	1	0.598763055	1
SalarSNP:GCR_cBin4749_Ctg	0.792922	1	0.373217975	0.996192022
SalarSNP:GCR_cBin5170_Ctg	0.930002	1	0.334862324	1
SalarSNP:GCR_cBin8189_Ctg	0.045179	1	0.83167526	1
SalarSNP:GCR_cBin8345_Ctg	0.051381	1	0.820676671	1
SalarSNP:GCR_cBin8585_Ctg	0.000419	1	0.983673494	1
SalarSNP:GCR_hBin20519_C	0.591132	1	0.441981746	0.996192022
SalarSNP:GCR_hBin7522_Ctg	2.644701	1	0.103895691	0.873196429
SalarSNP:GCR_rBin8898_Ctg	0.421523	1	0.516178056	1

SalarSNP:MHC_IA_41640:418	2.370168	1	0.123673737	0.936024096
SalarSNP:MHC_IA_42900:430	0.247498	1	0.618842683	1
SalHit:C123376875_123	1.08262	1	0.298112284	0.969060241
SalHit:C123427156_80	0.071702	1	0.788874323	1
SalHit:C123911743_415	0.860417	1	0.353622073	1
SalHit:C124022803_337	0.000123	1	0.991144634	1
SalHit:C124197598_304	2.511937	1	0.112987013	0.915683241
SalHit:C124761545_557	1.209712	1	0.271389265	0.961678135
SalHit:C124762720_287	0.051898	1	0.819793441	1
SalHit:S1027_7181	0.046122	1	0.829954797	1
SalHit:S1027_8940	0.000753	1	0.978107162	1
SalHit:S102823_1006	0.05288	1	0.81812565	1
SalHit:S102870_18665	2.762875	1	0.096474712	0.873196429
SalHit:S104654_669	0.325041	1	0.568593548	1
SalHit:S10486_5485	0.192775	1	0.660617288	1
SalHit:S105331_3668	0.342498	1	0.558391091	1
SalHit:S106841_6477	0.154657	1	0.694123695	1
SalHit:S108074_3154	2.293452	1	0.129920705	0.930719512
SalHit:S10848_3453	1.128098	1	0.288181305	0.97046332
SalHit:S10880_1341	5.97618	1	0.014500373	0.669071895
SalHit:S109150_1254	0.942055	1	0.331749902	1
SalHit:S111428_12207	4.23195	1	0.039669841	0.759688312
SalHit:S111428_3669	0.140551	1	0.707733609	1
SalHit:S111443_3771	0.013099	1	0.908879991	1
SalHit:S1132_954	0.440245	1	0.507004197	1
SalHit:S113308_970	0.19593	1	0.658026221	1
SalHit:S113858_5503	1.14066	1	0.285512977	0.996192022
SalHit:S114016_1877	0.163504	1	0.685951103	1
SalHit:S114474_2548	5.122745	1	0.023614233	0.669071895
SalHit:S114598_3165	0.240783	1	0.623641214	1
SalHit:S114598_6898	1.095772	1	0.295195793	0.996192022
SalHit:S11514_2858	2.942971	1	0.086251971	0.886540773
SalHit:S115876_228	0.054283	1	0.815771383	1
SalHit:S116298_7643	4.539836	1	0.033114769	0.712451282
SalHit:S117299_8462	0.001521	1	0.968895185	1
SalHit:S118711_4851	0.889855	1	0.34551671	0.996192022
SalHit:S118711_8963	1.473456	1	0.224801216	1
SalHit:S119227_1244	0.178234	1	0.672895583	1
SalHit:S119227_2435	0.000837	1	0.976913946	1
SalHit:S119971_1271	0.003855	1	0.950491722	1
SalHit:S123549_2712	5.837215	1	0.015690644	0.886061135
SalHit:S125768_4275	0.03484	1	0.851931462	1
SalHit:S126014_6497	0.81646	1	0.366217207	1
SalHit:S126310_347	0.001869	1	0.965513442	1
SalHit:S126835_16266	3.024062	1	0.082037745	0.873196429
SalHit:S126925_1562	0.103952	1	0.747137893	1
SalHit:S127117_1306	0.412553	1	0.520676381	1
SalHit:S129621_2871	0.727454	1	0.393709782	0.996192022

SalHit:S131311_2546	0.000929	1	0.975679869	1
SalHit:S131591_3093	0.514494	1	0.473199889	0.996192022
SalHit:S139984_3276	0.066831	1	0.796008342	1
SalHit:S140459_3613	3.966076	1	0.046425846	0.812444444
SalHit:S140659_7566	0.034006	1	0.85369466	1
SalHit:S1416_3847	1.00548	1	0.315988153	0.996192022
SalHit:S142292_1584	0.717984	1	0.396806141	1
SalHit:S142700_4079	0.094358	1	0.75870837	1
SalHit:S144233_6895	0.227815	1	0.63314882	1
SalHit:S145953_5771	0.000364	1	0.984787862	1
SalHit:S145953_9490	0.774041	1	0.378969848	1
SalHit:S146101_2536	0.033699	1	0.854347665	1
SalHit:S146115_2865	0.175455	1	0.675308734	1
SalHit:S146126_7561	0.179883	1	0.671474011	1
SalHit:S146911_10466	3.234933	1	0.072083243	0.826214689
SalHit:S147716_301	1.307963	1	0.252763812	1
SalHit:S148576_2204	6.434958	1	0.011189579	0.632769231
SalHit:S151563_1728	1.810391	1	0.178461337	0.957294737
SalHit:S154900_1539	0.0759	1	0.782932195	1
SalHit:S155290_2396	0.23577	1	0.627278372	1
SalHit:S155440_3655	1.907249	1	0.167269202	0.959095066
SalHit:S156798_3033	0.862686	1	0.352988119	1
SalHit:S159013_6091	0.003283	1	0.954305582	1
SalHit:S159124_1208	0.132682	1	0.71566685	1
SalHit:S161781_2673	0.131662	1	0.716714837	1
SalHit:S161781_3028	0.583457	1	0.444960618	1
SalHit:S161781_4577	0.395172	1	0.529593264	1
SalHit:S161886_4354	0.002144	1	0.96307173	1
SalHit:S162316_3358	0.081409	1	0.775397424	1
SalHit:S163322_1067	2.890624	1	0.089096429	0.839240106
SalHit:S164388_623	0.113717	1	0.735951842	1
SalHit:S166075_4193	0.945236	1	0.330934786	0.996192022
SalHit:S166075_5092	0.641465	1	0.423180772	1
SalHit:S166885_4145	0.094413	1	0.758640344	1
SalHit:S167224_1311	3.552857	1	0.059443009	0.812444444
SalHit:S167714_1334	0.62547	1	0.429022015	1
SalHit:S168157_991	0.15583	1	0.693025436	1
SalHit:S1706_14812	5.50E-05	1	0.994082501	1
SalHit:S171093_1659	1.463527	1	0.22636975	0.982108556
SalHit:S171769_2652	0.0759	1	0.782932195	1
SalHit:S175270_692	0.634959	1	0.425542274	1
SalHit:S175742_1533	0.232674	1	0.629547976	1
SalHit:S176657_4893	0.014903	1	0.902837459	1
SalHit:S176728_4365	0.492245	1	0.482927612	1
SalHit:S183101_2595	0.939128	1	0.332502241	0.996192022
SalHit:S185934_458	0.012173	1	0.912146246	1
SalHit:S187171_19839	5.953403	1	0.014688899	0.632769231
SalHit:S187171_8005	0.492245	1	0.482927612	1

SalHit:S188619_2201	0.253728	1	0.614462357	1
SalHit:S188619_7117	0.361291	1	0.547790068	1
SalHit:S18885_1674	0.007809	1	0.929581905	1
SalHit:S191379_6348	0.024741	1	0.875012922	1
SalHit:S19196_949	1.494932	1	0.221452757	0.97046332
SalHit:S192156_16661	1.106451	1	0.292854748	0.992751958
SalHit:S199057_2374	0.003084	1	0.955713602	1
SalHit:S202609_547	0.257989	1	0.611505071	1
SalHit:S205437_11417	4.145937	1	0.041734552	0.78448583
SalHit:S205573_1241	0.31931	1	0.572022383	1
SalHit:S208211_6081	0.314091	1	0.575180331	1
SalHit:S20943_3475	0.319694	1	0.571791316	1
SalHit:S212497_8172	0.722233	1	0.395412272	0.996192022
SalHit:S214020_2561	0.66325	1	0.415414862	1
SalHit:S214652_2227	0.687247	1	0.407102475	0.996192022
SalHit:S215479_2458	1.823323	1	0.176918235	0.959095066
SalHit:S217001_1739	5.609923	1	0.017859047	0.623181818
SalHit:S217108_4004	0.798126	1	0.371654199	1
SalHit:S217153_2544	0.121962	1	0.726915831	1
SalHit:S221112_266	8.70878	1	0.00316681	0.373061224
SalHit:S222106_10549	0.666188	1	0.414383888	1
SalHit:S22575_3588	3.108853	1	0.077867764	0.866134663
SalHit:S22575_5272	0.781907	1	0.376558527	1
SalHit:S226473_1887	0.164823	1	0.684754764	1
SalHit:S227738_2874	0.571878	1	0.449513421	1
SalHit:S227800_14465	0.51231	1	0.474140629	1
SalHit:S227800_3402	0.01397	1	0.905914468	1
SalHit:S227800_4162	1.19729	1	0.273863912	0.968970438
SalHit:S228814_2074	0.308669	1	0.578497817	1
SalHit:S233419_8892	0.442553	1	0.505892884	1
SalHit:S233811_1875	3.663432	1	0.055618938	0.787240876
SalHit:S233811_2086	0.3716	1	0.542133665	1
SalHit:S234782_2105	0.150737	1	0.697831937	1
SalHit:S234924_3667	0.567951	1	0.451074383	1
SalHit:S235382_924	0.068426	1	0.793642185	1
SalHit:S23849_2475	0.71317	1	0.398393416	0.996192022
SalHit:S240613_4416	0.085486	1	0.769995946	1
SalHit:S240648_3170	1.99025	1	0.158314731	0.930719512
SalHit:S241808_9086	0.557715	1	0.455182548	1
SalHit:S242306_1973	0.000514	1	0.98190591	1
SalHit:S242729_7709	0.172097	1	0.67825479	1
SalHit:S243747_5691	1.390543	1	0.238313377	0.983407136
SalHit:S243803_4071	0.943025	1	0.331501047	0.983407136
SalHit:S244701_3876	1.706068	1	0.191496301	0.961678135
SalHit:S245575_1093	0.010219	1	0.919478054	1
SalHit:S249108_1342	5.228856	1	0.02221514	0.669071895
SalHit:S249521_11216	0.193495	1	0.660023314	1
SalHit:S253158_3206	4.039862	1	0.044437447	0.734784314

SalHit:S253707_4432	3.437258	1	0.063741413	0.846554707
SalHit:S254544_1255	0.3716	1	0.542133665	1
SalHit:S25721_5836	0.015151	1	0.902037404	1
SalHit:S25721_7918	0.038474	1	0.844495343	1
SalHit:S257951_2189	0.106244	1	0.744461687	1
SalHit:S258084_579	3.458026	1	0.062945426	0.826214689
SalHit:S258364_2087	0.003759	1	0.951109536	1
SalHit:S259229_3718	0.323609	1	0.569446934	1
SalHit:S260262_255	0.000753	1	0.978107162	1
SalHit:S260584_10644	1.108884	1	0.292324699	0.996192022
SalHit:S260584_2458	0.012173	1	0.912146246	1
SalHit:S261274_20497	0.234783	1	0.627999504	1
SalHit:S261274_7888	0.059802	1	0.806809128	1
SalHit:S261633_28093	1.808125	1	0.17873336	0.959095066
SalHit:S261794_1054	2.016249	1	0.155623197	0.930719512
SalHit:S262166_3433	0.472006	1	0.492065871	1
SalHit:S262497_3516	0.110076	1	0.74005788	1
SalHit:S263117_5869	0.908874	1	0.340413251	0.996192022
SalHit:S267277_747	1.844146	1	0.174465821	0.946642857
SalHit:S267372_2757	8.039849	1	0.004575935	0.332363636
SalHit:S268906_11932	0.865012	1	0.352339919	0.999306667
SalHit:S269412_918	0.751753	1	0.385921654	0.996192022
SalHit:S273164_1801	0.3716	1	0.542133665	1
SalHit:S274116_5651	0.860417	1	0.353622073	1
SalHit:S279268_1478	0.445614	1	0.50442514	1
SalHit:S279771_1404	0.955224	1	0.328393158	0.996192022
SalHit:S279771_2947	0.360106	1	0.548447496	1
SalHit:S280657_1162	2.16896	1	0.140821435	0.930719512
SalHit:S282118_3930	0.173646	1	0.676891367	1
SalHit:S28329_3550	0.057798	1	0.810010481	1
SalHit:S285971_2992	1.025769	1	0.311154523	0.996192022
SalHit:S286928_2598	4.169872	1	0.041148905	0.826214689
SalHit:S288563_5173	0.121962	1	0.726915831	1
SalHit:S288563_5644	0.057798	1	0.810010481	1
SalHit:S288563_7916	0.024194	1	0.876392996	1
SalHit:S29463_5965	0.28754	1	0.591801434	1
SalHit:S295324_1768	0.228679	1	0.632504783	1
SalHit:S297381_2880	3.448104	1	0.063324379	0.826214689
SalHit:S300505_4118	0.126474	1	0.722116607	1
SalHit:S304891_1949	0.285416	1	0.593173237	1
SalHit:S304891_767	1.292438	1	0.255599094	0.962317181
SalHit:S306722_1825	0.04509	1	0.831837905	1
SalHit:S311726_3277	0.111966	1	0.737916839	1
SalHit:S312971_3029	0.029331	1	0.864017157	1
SalHit:S315074_1366	0.648529	1	0.420639053	1
SalHit:S3164_1583	0.004405	1	0.947085065	1
SalHit:S3164_6418	0.857028	1	0.354571708	1
SalHit:S316727_1311	8.31E-07	1	0.999272518	1

SalHit:S317662_1985	0.086219	1	0.76904005	1
SalHit:S318001_4665	0.085924	1	0.769424988	1
SalHit:S31821_9847	1.318964	1	0.250778037	0.983407136
SalHit:S318567_1701	0.032963	1	0.855930356	1
SalHit:S323319_843	0.138634	1	0.709642598	1
SalHit:S324937_1374	3.083379	1	0.079096054	0.839240106
SalHit:S328044_4638	1.087091	1	0.29711672	0.996192022
SalHit:S32936_4225	0.066153	1	0.797023262	1
SalHit:S330374_228	0.147811	1	0.700636543	1
SalHit:S331166_5798	0.545991	1	0.459960225	1
SalHit:S335584_5764	11.31074	1	0.000770601	0
SalHit:S337125_1011	0.000414	1	0.983775114	1
SalHit:S33900_1460	0.690725	1	0.405918048	1
SalHit:S339911_3339	0.128933	1	0.719541024	1
SalHit:S339911_6409	0.175455	1	0.675308734	1
SalHit:S341408_5153	0.032106	1	0.857794913	1
SalHit:S341443_3903	0.027297	1	0.868771806	1
SalHit:S341443_5355	0.067745	1	0.79464872	1
SalHit:S341846_8220	1.193306	1	0.274663648	0.991787234
SalHit:S341887_2153	0.677207	1	0.410550036	0.996192022
SalHit:S341987_3098	0.492245	1	0.482927612	1
SalHit:S343921_1954	0.001271	1	0.971560648	1
SalHit:S344034_1491	0.100854	1	0.750806963	1
SalHit:S34679_17551	0.04509	1	0.831837905	1
SalHit:S34679_800	3.609305	1	0.05745712	0.812444444
SalHit:S34693_1686	0.904426	1	0.341597712	0.996192022
SalHit:S349460_2626	0.007774	1	0.92974164	1
SalHit:S353786_532	0.24925	1	0.617603633	1
SalHit:S355030_2257	0.18981	1	0.663074722	1
SalHit:S355279_1207	0.139482	1	0.708796359	1
SalHit:S35637_2122	0.241319	1	0.623255182	1
SalHit:S356513_2505	2.078372	1	0.149399448	0.923049505
SalHit:S357188_2413	1.288659	1	0.256295175	0.984691538
SalHit:S35917_3484	2.916757	1	0.087663904	0.886540773
SalHit:S361210_2144	0.343359	1	0.557896847	1
SalHit:S365449_7529	0.943025	1	0.331501047	0.996192022
SalHit:S371829_3660	0.588867	1	0.442857505	1
SalHit:S372646_3054	0.004948	1	0.94392169	1
SalHit:S372646_403	0.512054	1	0.474251062	1
SalHit:S372965_4460	0.498577	1	0.480125871	1
SalHit:S373646_810	0.022871	1	0.879792108	1
SalHit:S37375_7111	0.075362	1	0.783683471	1
SalHit:S375996_4447	0.094334	1	0.758737629	1
SalHit:S37673_4072	0.326464	1	0.56774886	1
SalHit:S37979_3170	1.799832	1	0.179732785	0.957294737
SalHit:S38141_6780	0.023446	1	0.878302804	1
SalHit:S38409_6737	1.121061	1	0.28969003	0.996192022
SalHit:S386453_3287	4.568363	1	0.032567676	0.787240876

SalHit:S386453_5112	0.180619	1	0.670842247	1
SalHit:S389388_6314	0.696532	1	0.403951417	1
SalHit:S39061_10111	0.171128	1	0.679111156	1
SalHit:S392460_1601	1.131048	1	0.287551838	0.996192022
SalHit:S393517_1929	1.351328	1	0.24504615	0.968970438
SalHit:S394519_1767	2.051612	1	0.152045145	0.957294737
SalHit:S395278_4852	9.26422	1	0.00233674	0.421846154
SalHit:S396585_2965	0.897647	1	0.343413299	0.996192022
SalHit:S401259_10429	0.558735	1	0.454770415	1
SalHit:S402209_5711	0.061524	1	0.80410269	1
SalHit:S402232_1651	7.076875	1	0.007808507	0.421846154
SalHit:S406713_5055	0.425027	1	0.514439406	1
SalHit:S406986_4178	0.16969	1	0.680387831	1
SalHit:S407026_7260	1.60785	1	0.204794289	0.97046332
SalHit:S407342_880	4.786565	1	0.028682567	0.677037037
SalHit:S409379_746	0.022412	1	0.880997194	1
SalHit:S409665_1998	1.1144	1	0.291127499	0.959095066
SalHit:S413354_2932	0.154544	1	0.694230013	1
SalHit:S413688_3464	6.74E-05	1	0.99344982	1
SalHit:S414332_763	0.038744	1	0.843956889	1
SalHit:S417941_755	2.203489	1	0.137698802	0.923049505
SalHit:S420806_11496	1.689637	1	0.193648822	0.959095066
SalHit:S420806_8290	0.183083	1	0.668736789	1
SalHit:S42917_9951	0.297215	1	0.585633503	1
SalHit:S4303_9108	0.66325	1	0.415414862	1
SalHit:S43071_3808	0.065933	1	0.797353309	1
SalHit:S432900_3804	0.458438	1	0.498354959	1
SalHit:S434130_330	1.515168	1	0.218352154	0.959095066
SalHit:S43861_1436	0.004405	1	0.947085065	1
SalHit:S43861_2337	0.146884	1	0.701530691	1
SalHit:S440663_362	0.824719	1	0.363804019	0.996192022
SalHit:S440995_2172	0.299358	1	0.584285487	1
SalHit:S44456_2602	0.007501	1	0.930983192	1
SalHit:S44456_5540	10.20186	1	0.001402989	0.322588235
SalHit:S447810_3696	2.284099	1	0.130706068	0.951071329
SalHit:S452627_2679	0.780489	1	0.376991579	1
SalHit:S453385_2176	3.072494	1	0.079627231	0.886540773
SalHit:S453385_428	1.005211	1	0.31605292	1
SalHit:S453385_786	3.267155	1	0.070680112	0.794782609
SalHit:S453677_1818	0.016083	1	0.899084487	1
SalHit:S45460_2763	1.684875	1	0.194278003	0.968970438
SalHit:S45460_891	2.18136	1	0.139690964	0.923049505
SalHit:S460384_3598	0.263612	1	0.60764975	1
SalHit:S463531_4086	3.344732	1	0.067420441	0.823205298
SalHit:S46568_10274	0.086219	1	0.76904005	1
SalHit:S466797_6775	0.336095	1	0.562092272	1
SalHit:S468867_1710	0.207001	1	0.649128223	1
SalHit:S468867_988	1.922631	1	0.165566876	0.968970438

SalHit:S468935_401	0.165837	1	0.683837982	1
SalHit:S469071_2535	3.865774	1	0.049280457	0.734784314
SalHit:S469071_971	0.896794	1	0.343642881	1
SalHit:S47024_3427	1.326617	1	0.249407964	0.968970438
SalHit:S47024_3725	0.911037	1	0.339839472	0.996192022
SalHit:S481938_1027	0.191748	1	0.66146592	1
SalHit:S482017_6928	1.038337	1	0.308208451	0.996192022
SalHit:S48341_1363	7.075675	1	0.007813737	0.595162791
SalHit:S483775_2814	0.061385	1	0.804320734	1
SalHit:S483775_3667	0.735716	1	0.391036782	0.996192022
SalHit:S490385_4491	0.9722	1	0.324132133	0.996192022
SalHit:S49076_2139	0.678553	1	0.410085429	0.98872956
SalHit:S49076_2694	1.08262	1	0.298112284	0.969060241
SalHit:S49076_7001	2.841087	1	0.09188202	0.923049505
SalHit:S49076_9602	0.038655	1	0.844134472	1
SalHit:S49089_986	0.110076	1	0.74005788	1
SalHit:S49115_8466	0.18293	1	0.66886728	1
SalHit:S492378_5464	0.109869	1	0.740293007	1
SalHit:S492378_7434	0.28754	1	0.591801434	1
SalHit:S49260_1709	0.234883	1	0.627926856	1
SalHit:S494492_5892	3.094172	1	0.078573117	0.845861183
SalHit:S500022_1016	0.485784	1	0.48581396	1
SalHit:S502826_4965	1.948187	1	0.16278231	0.930719512
SalHit:S503742_5592	1.108884	1	0.292324699	0.996192022
SalHit:S50453_1549	9.165362	1	0.002466388	0.152333333
SalHit:S505394_4239	1.080373	1	0.298614153	1
SalHit:S508212_1407	0.007438	1	0.931272344	1
SalHit:S509135_6353	0.179459	1	0.671838162	1
SalHit:S510473_1387	0.001271	1	0.971560648	1
SalHit:S51494_11690	0.212923	1	0.644486199	1
SalHit:S51494_16577	1.217531	1	0.269845834	0.984691538
SalHit:S51494_16759	1.640944	1	0.200196003	0.968970438
SalHit:S516356_4851	0.791524	1	0.37363976	1
SalHit:S517254_2461	0.588867	1	0.442857505	1
SalHit:S518988_677	0.10349	1	0.747680926	1
SalHit:S519253_1139	4.192672	1	0.04059906	0.787240876
SalHit:S519253_4343	1.462525	1	0.226528868	0.972155745
SalHit:S51948_2505	0.220364	1	0.63876254	1
SalHit:S51948_4671	0.013473	1	0.907593396	1
SalHit:S52170_270	18.1041	1	2.09E-05	0
SalHit:S525071_6038	1.59E-05	1	0.99682218	1
SalHit:S525071_6983	0.191748	1	0.66146592	1
SalHit:S525370_259	0.630749	1	0.427080765	1
SalHit:S525583_945	0.010219	1	0.919478054	1
SalHit:S527948_252	0.389608	1	0.532505476	1
SalHit:S530137_1949	0.112346	1	0.73748894	1
SalHit:S531204_1936	0.784897	1	0.37564767	1
SalHit:S53219_2845	0.83388	1	0.361152936	0.996192022

SalHit:S534814_2714	1.016822	1	0.313274085	0.999306667
SalHit:S5395_4782	0.000753	1	0.978107162	1
SalHit:S5395_7944	1.948187	1	0.16278231	0.930719512
SalHit:S540769_959	0.014196	1	0.905160412	1
SalHit:S542331_3847	1.032093	1	0.309667479	0.996192022
SalHit:S54277_3619	0.845186	1	0.357917702	0.996192022
SalHit:S543389_5919	0.912745	1	0.339387158	1
SalHit:S54501_5201	0.04096	1	0.839615679	1
SalHit:S54868_1385	0.117653	1	0.731594504	1
SalHit:S550185_614	0.790276	1	0.374016657	1
SalHit:S55224_232	0.332855	1	0.563983014	1
SalHit:S552727_1630	1.073205	1	0.300222671	0.972666047
SalHit:S558206_4087	6.41E-05	1	0.993611253	1
SalHit:S559536_6183	0.401548	1	0.526290665	1
SalHit:S56217_940	0.314091	1	0.575180331	1
SalHit:S563209_3485	0.033638	1	0.854478967	1
SalHit:S567449_927	0.796425	1	0.372164248	0.996192022
SalHit:S568137_774	2.413775	1	0.120272056	0.923049505
SalHit:S575105_1693	0.319289	1	0.57203518	1
SalHit:S576605_3402	0.069349	1	0.792286314	1
SalHit:S57706_1989	4.145937	1	0.041734552	0.734784314
SalHit:S577812_3658	0.583963	1	0.444762951	0.996192022
SalHit:S578279_333	3.132173	1	0.076761351	0.894878661
SalHit:S581751_1502	0.16969	1	0.680387831	1
SalHit:S58313_4910	1.515661	1	0.218277323	0.972155745
SalHit:S584677_840	0.578037	1	0.447082783	1
SalHit:S585005_1586	0.098222	1	0.753974394	1
SalHit:S592297_1046	2.810509	1	0.093648631	0.873196429
SalHit:S592297_2656	2.391055	1	0.12203122	0.910498084
SalHit:S594047_1310	0.001869	1	0.965513442	1
SalHit:S594636_3547	1.077091	1	0.299349334	0.98872956
SalHit:S597042_1961	0.037463	1	0.846525668	1
SalHit:S597256_2140	0.240368	1	0.623940336	1
SalHit:S600261_600	0.132682	1	0.71566685	1
SalHit:S600699_4332	2.379658	1	0.122924452	0.930719512
SalHit:S602336_6773	0.107584	1	0.742911378	1
SalHit:S604501_4136	0.825919	1	0.363455288	0.996192022
SalHit:S605138_705	1.312317	1	0.251975563	0.97046332
SalHit:S609634_1036	0.000742	1	0.978267909	1
SalHit:S612302_2168	1.501836	1	0.220389009	0.959095066
SalHit:S612302_7117	3.903947	1	0.048172793	0.831364384
SalHit:S612770_3168	0.113717	1	0.735951842	1
SalHit:S614561_3987	0.072648	1	0.787519923	1
SalHit:S618840_2758	0.013656	1	0.906971274	1
SalHit:S619952_1799	6.335643	1	0.011833524	0.706272727
SalHit:S621605_4762	2.253409	1	0.133320374	0.930719512
SalHit:S62224_1665	1.88721	1	0.169516974	0.959095066
SalHit:S62399_1028	1.791935	1	0.180690625	0.957294737

SalHit:S626715_3624	1.009893	1	0.314928439	1
SalHit:S627974_2057	2.078372	1	0.149399448	0.923049505
SalHit:S63194_3846	0.532217	1	0.465676097	1
SalHit:S634821_498	0.007416	1	0.931372413	1
SalHit:S636915_947	0.012988	1	0.909264519	1
SalHit:S640273_2950	0.00283	1	0.957577477	1
SalHit:S641000_5592	0.640505	1	0.423527934	1
SalHit:S642671_1637	0.000753	1	0.978107162	1
SalHit:S643886_6448	1.100636	1	0.294126663	0.996192022
SalHit:S645775_422	2.049426	1	0.152263602	0.948045845
SalHit:S64808_1923	0.205909	1	0.649992779	1
SalHit:S64808_2593	0.556877	1	0.455521276	1
SalHit:S648440_1926	0.023762	1	0.877491647	1
SalHit:S649731_1232	0.485043	1	0.486146971	1
SalHit:S653212_1140	1.99523	1	0.157795103	0.923049505
SalHit:S653212_179	0.37543	1	0.540059155	1
SalHit:S653524_6455	0.102364	1	0.749011155	1
SalHit:S65493_4081	0.117653	1	0.731594504	1
SalHit:S65799_1161	0.508928	1	0.475603097	1
SalHit:S661159_285	0.372866	1	0.541446017	1
SalHit:S661277_5664	0.189846	1	0.663045217	1
SalHit:S673707_838	1.86299	1	0.172280118	0.94994382
SalHit:S675517_922	0.818913	1	0.365498081	1
SalHit:S678411_577	0.577471	1	0.447305447	1
SalHit:S679571_2134	0.007438	1	0.931272344	1
SalHit:S681882_2890	1.318964	1	0.250778037	0.995067826
SalHit:S681882_3013	0.089898	1	0.764307005	1
SalHit:S683685_828	0.002007	1	0.964269798	1
SalHit:S687744_7975	0.623659	1	0.42969065	1
SalHit:S68852_10003	1.478137	1	0.224066292	0.962317181
SalHit:S688722_1069	0.041504	1	0.838568924	1
SalHit:S694964_1144	0.143344	1	0.704978554	1
SalHit:S697750_3426	1.121042	1	0.289694141	0.996192022
SalHit:S699749_1912	0.453698	1	0.500584052	1
SalHit:S70344_7682	1.601123	1	0.205744124	0.957294737
SalHit:S704716_2081	1.648841	1	0.199116743	0.959095066
SalHit:S706284_4420	0.094363	1	0.758702666	1
SalHit:S707709_1535	1.15E-05	1	0.997292869	1
SalHit:S718588_3898	1.934191	1	0.164300622	0.923049505
SalHit:S720489_1453	0.711525	1	0.398937849	1
SalHit:S726481_118	0.057746	1	0.810093922	1
SalHit:S73487_2332	0.192775	1	0.660617288	1
SalHit:S735493_1940	0.694347	1	0.404689761	1
SalHit:S736134_5529	0.006491	1	0.935785299	1
SalHit:S736134_7346	1.763489	1	0.184189952	0.94994382
SalHit:S737061_1987	0.073085	1	0.786897213	1
SalHit:S744526_2175	0.134499	1	0.713811947	1
SalHit:S74545_5079	0.232674	1	0.629547976	1

SalHit:S747242_398	0.020404	1	0.88641571	1
SalHit:S74979_874	5.392601	1	0.020222308	0.595162791
SalHit:S752338_5854	0.237393	1	0.626095542	1
SalHit:S75583_3748	0.16694	1	0.682845931	1
SalHit:S758078_2162	0.023762	1	0.877491647	1
SalHit:S76032_10732	1.122674	1	0.289343425	0.996192022
SalHit:S762083_2146	0.638282	1	0.424333732	1
SalHit:S76349_7595	0.332855	1	0.563983014	1
SalHit:S76374_963	3.255501	1	0.071184164	0.826214689
SalHit:S768178_1923	0.227732	1	0.633210806	1
SalHit:S768213_1908	0.293506	1	0.587982512	1
SalHit:S768218_2293	1.364462	1	0.242765656	0.961678135
SalHit:S776102_2112	0.344197	1	0.55741667	1
SalHit:S781410_2479	0.25402	1	0.614258688	1
SalHit:S78704_4164	0.512054	1	0.474251062	1
SalHit:S78760_103	3.108393	1	0.07788977	0.837628032
SalHit:S787651_531	2.097831	1	0.147508219	0.923049505
SalHit:S795010_298	2.529542	1	0.111732636	0.923049505
SalHit:S797343_7313	1.329518	1	0.248891021	0.97046332
SalHit:S797477_1052	6.74E-05	1	0.99344982	1
SalHit:S797477_1574	6.876617	1	0.008733069	0.669071895
SalHit:S798853_175	0.005056	1	0.943311493	1
SalHit:S799945_710	0.140551	1	0.707733609	1
SalHit:S801501_1444	0.019822	1	0.888035531	1
SalHit:S808420_898	0.188466	1	0.664196692	1
SalHit:S81217_10509	0.794143	1	0.372850164	0.996192022
SalHit:S81217_8404	0.741769	1	0.389094939	1
SalHit:S81886_3734	0.042224	1	0.837193781	1
SalHit:S820211_536	1.356574	1	0.244132165	0.972666047
SalHit:S820702_1332	0.014419	1	0.904420019	1
SalHit:S82254_6678	2.208528	1	0.137249545	0.930719512
SalHit:S826387_869	0.366695	1	0.544811308	1
SalHit:S828444_1201	0.146884	1	0.701530691	1
SalHit:S833101_575	0.175914	1	0.674908846	1
SalHit:S83418_6389	0.027569	1	0.868125	1
SalHit:S834419_990	2.84929	1	0.091414299	0.873196429
SalHit:S838387_395	4.077236	1	0.043464691	0.747448889
SalHit:S838387_712	0.490969	1	0.483495451	1
SalHit:S83919_2393	0.001135	1	0.973126044	1
SalHit:S839660_1318	0.680194	1	0.409520035	1
SalHit:S85177_10121	0.072648	1	0.787519923	1
SalHit:S85907_571	0.275047	1	0.599966362	1
SalHit:S88367_11998	0.010779	1	0.917308735	1
SalHit:S88367_2537	2.203489	1	0.137698802	0.910498084
SalHit:S88367_9559	0.081871	1	0.774777259	1
SalHit:S90355_10517	1.069475	1	0.301063973	0.996192022
SalHit:S90355_11358	0.121808	1	0.727081743	1
SalHit:S90996_1668	0.500717	1	0.479185106	1

SalHit:S91307_10080	3.455141	1	0.063055357	0.890957983
SalHit:S91307_4577	0.436827	1	0.508657779	1
SalHit:S91955_5816	0.001253	1	0.971768001	1
SalHit:S92841_633	0.514494	1	0.473199889	1
SalHit:S93239_12662	0.117653	1	0.731594504	1
SalHit:S95866_2343	2.857204	1	0.09096551	0.873196429
SalHit:S97503_354	1.95231	1	0.162338116	0.957294737
SalHit:S98604_475	0.583963	1	0.444762951	0.996192022
SalHit:S98813_6560	0.647942	1	0.420849361	1

Supplementary Table S3. Results from *structure* clustering analyses showing probabilities for K 1-5 and derived Delta K estimation.

K	Repe-titions	Mean LnP(K)	SD LnP(K)	Ln'(K)	|Ln''(K)|	Delta K
1	5	-1078059.8	7.7	NA	NA	NA
2	5	-1069056.7	41.0	9003.1	5986.9	146.0
3	5	-1066040.5	38.4	3016.2	2257.7	58.9
4	5	-1065281.9	2352.4	758.5	257.6	0.1
5	5	-1064781	3807.0	500.9	NA	NA
